# Decarbonylative organoboron cross-coupling of esters by nickel catalysis

**DOI:** 10.1038/ncomms8508

**Published:** 2015-06-29

**Authors:** Kei Muto, Junichiro Yamaguchi, Djamaladdin G. Musaev, Kenichiro Itami

**Affiliations:** 1 Institute of Transformative Bio-Molecules (WPI-ITbM) and Graduate School of Science, Nagoya University, Chikusa, Nagoya 464-8602, Japan; 2 Emory College's Research Center and Department of Chemistry, Emory University, 1515 Dickey Drive, Atlanta, Georgia 30322, US; 3 JST-ERATO, Itami Molecular Nanocarbon Project, Nagoya University, Chikusa, Nagoya 464-8602, Japan

## Abstract

The Suzuki–Miyaura cross-coupling is a metal-catalysed reaction in which boron-based nucleophiles and halide-based electrophiles are reacted to form a single molecule. This is one of the most reliable tools in synthetic chemistry, and is extensively used in the synthesis of pharmaceuticals, agrochemicals and organic materials. Herein, we report a significant advance in the choice of electrophilic coupling partner in this reaction. With a user-friendly and inexpensive nickel catalyst, a range of phenyl esters of aromatic, heteroaromatic and aliphatic carboxylic acids react with boronic acids in a decarbonylative manner. Overall, phenyl ester moieties function as leaving groups. Theoretical calculations uncovered key mechanistic features of this unusual decarbonylative coupling. Since extraordinary numbers of ester-containing molecules are available both commercially and synthetically, this new ‘ester' cross-coupling should find significant use in synthetic chemistry as an alternative to the standard halide-based Suzuki–Miyaura coupling.

## Abstract (web summary)

The Suzuki-Miyaura cross-coupling is a mainstay of organic synthesis, allowing carbon-carbon bond formation between a variety of coupling partners. Here, the authors report a decarbonylative process, whereby alkyl or aryl esters can be coupled with organoboron compounds using nickel catalysts.

## Graphical abstract

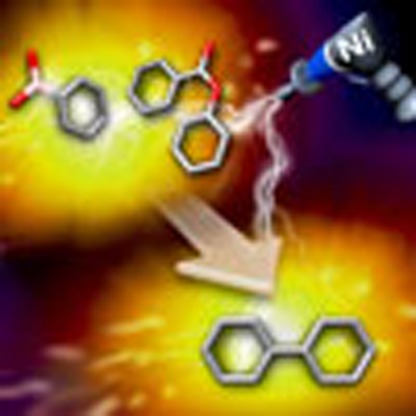

The Suzuki–Miyaura coupling, a palladium- or nickel-catalysed cross-coupling reaction of boron-based nucleophiles and organic electrophiles^1^,^2^, is one of the most reliable reactions in synthetic organic chemistry, indispensable in the synthesis of a range of functional organic materials ranging from pharmaceuticals, agrochemicals, organic electronic devices and liquid crystals (Fig. 1a). Conventionally, the Suzuki–Miyaura coupling uses organic halides as the electrophilic component^3^. The development of a new mode of bond/group activation is expected to offer great opportunities, particularly when it leads to unconventional, streamlined organic syntheses. Recent advances include the successful utilization of aniline derivatives such as diazonium and ammonium salts^4^,^5^, anisole derivatives^6^ and many other compounds^7^,^8^,^9^,^10^,^11^ as coupling partners for boronic acids. Here we describe a rare example of the unconventional use of esters as the electrophilic component in a nickel-catalysed Suzuki–Miyaura cross-coupling.

The use of esters in the Suzuki–Miyaura coupling is important not only because this avoids the production of corrosive halide-containing wastes, but also because it opens access to a vast number of commercially and synthetically available ester-containing molecules (Fig. 1b). Moreover, ester groups are often associated with heterocycle synthesis, for example, Knorr pyrrole synthesis, Feist–Bénary furan synthesis and Hantzsch dihydropyridine synthesis, where these functionalities are important components of the heterocycle cores (Fig. 1b). Thus, the successful implementation of this simple ‘ester-based' Suzuki–Miyaura coupling would permit these ester groups to be used directly as leaving groups. Given the recent rapid progress in C–H borylation chemistry^12^, which has enormously increased the accessibility of boron-based reagents, the target ‘ester-based' Suzuki–Miyaura coupling is expected to significantly advance organic synthesis. Furthermore, the use of earth-abundant first-row metal nickel as a catalyst in the target coupling makes this reaction commercially more appealing.

In recent years, there is a growing interest in the use of aroyl compounds in metal-catalysed decarboxylative or decarbonylative coupling reactions^13^,^14^. Gooßen *et al.*^15^ reported that aromatic carboxylic acids and haloarenes could be cross-coupled in a decarboxylative manner employing a palladium-copper bimetallic catalytic system. Arising from this groundbreaking discovery, a number of decarboxylative or decarbonylative cross-coupling reactions with aroyl compounds have been reported^13^,^14^. Gooßen and Paetzoid^16^ reported that arylcarboxylic anhydrides can be cross-coupled with arylboroxines in a decarbonylative manner in the presence of rhodium catalyst to give the corresponding biaryls. A chelation-assisted decarbonylative coupling of ethyl benzo[*h*]quinoline-10-carboxylate and arylboronic acids is also possible with rhodium catalyst^17^. The decarbonylative transformations of carboxylic anhydrides are also reported in nickel-based system^18^,^19^,^20^. For example, Rovis and co-workers^19^ reported that cyclic anhydrides react with diphenylzinc in the presence of a stoichiometric amount of nickel complex to provide the corresponding decarbonylative phenylation products. Phthalimides also react in a similar manner with diorganozinc reagents, but this reaction also requires a stoichiometric amount of nickel complex^20^.

Although ester groups have remained elusive electrophiles in the Suzuki–Miyaura coupling, we devised a decarbonylative cross-coupling manifold employing Ni^0^/Ni^II^ redox catalysis (Fig. 1c). Prior experimental and theoretical investigations^18^,^19^,^20^,^21^,^22^,^23^,^24^,^25^ have shown that oxidative addition of the C(acyl)–O bond of certain carboxylic acid esters to Ni^0^ complexes leads to the formation of an acylnickel(II) intermediate that could then undergo (i) transmetalation reaction with a boronic acid and (ii) decarbonylation to produce a diorganonickel(II) intermediate. Subsequent reductive elimination releases the decarbonylative cross-coupling product and regenerates Ni^0^ species. Armed by these knowledge and the discovered beneficial impact of a phenyl substituent on the cleavage of C(acyl)–O bonds by nickel catalysts^22^,^25^, we primarily focused on the use of phenyl esters of arenecarboxylic acids (ArCO_2_Ph) in this study.

## Results

### Discovery of decarbonylative cross-coupling of esters

We initiated this study by finding suitable catalytic conditions for the decarbonylative cross-coupling of esters and boronic acids, using phenyl 3-pyridinecarboxylate (**1A**) and *p*-anisylboronic acid (**2a**) as model substrates. We focused on the use of nickel catalysts due to their low cost and our extensive experience^26^,^27^,^28^,^29^,^30^,^31^,^32^,^33^,^34^. Representative results are shown in Table 1 (see Supplementary Tables 1–3 for full details). We found that, in the presence of a catalytic amount of Ni(cod)_2_ (10 mol%; cod (1,5-cyclooctadiene)) and Na_2_CO_3_ as base, the decarbonylative cross-coupling did not proceed even at 150 °C (entry 1). The use of previously successful bidentate phosphines^22^,^25^,^31^,^32^,^33^,^34^, such as dcype (1,2-bis(dicyclohexylphosphino)ethane) and dppe (1,2-bis(diphenylphosphino)ethane) as ligands for nickel, did not produce coupling product **3Aa** either (entries 2 and 3). Gratifyingly, further screening showed that the use of dpph (1,6-bis(diphenylphosphino)hexane) furnished **3Aa** in 51% yield (entry 4). With the assumption that dpph is acting as a monodentate ligand, we turned our attention to monodentate phosphine ligands, and we were delighted to discover that commercially available and inexpensive P(*n*-Bu)_3_ performed well in the decarbonylative cross-coupling (PCy_3_, 26%; PPh_2_Me, 47% and P(*n*-Bu)_3_, 65%). We determined Na_2_CO_3_ to be the optimal base due to its relatively low cost, but an equally important finding is the successful coupling in the absence of an exogenous base (entry 8) as is usually required in typical halide-based Suzuki–Miyaura couplings^1^,^2^. The coupling can also be conducted in various solvents (ethers, hydrocarbons and alcohols). Fortunately, the use of toluene, which is also inexpensive and a preferred solvent in process chemistry, provided 95% yield of **3Aa** (entry 9). Even more pleasingly, we found that Ni(OAc)_2_, which is much more stable and less expensive than Ni(cod)_2_ and typical palladium complexes (Ni(OAc)_2_·4H_2_O, $108 per kg from Sigma-Aldrich), functioned as a pre-catalyst yielding **3Aa** in 88% yield at 5 mol% catalyst loading (entry 10). Temperature effects were also examined, and 150 °C was determined to be optimal. Esters prepared from phenol and its derivatives are effective substrates in this particular coupling, and ethyl esters are virtually unreactive under otherwise identical conditions. In almost all cases, we did not observe the formation of carbonylated coupling product (diarylketone)^13^, which indicates the high capability of nickel to facilitate the decarbonylation process. Taken together, we have established an operationally simple, economical/ecological sound, user-friendly, nickel-based catalytic system affecting organoboron-based, decarbonylative cross-coupling reactions using esters as electrophilic coupling partners.

### Mechanistic considerations

**Figure 2:**
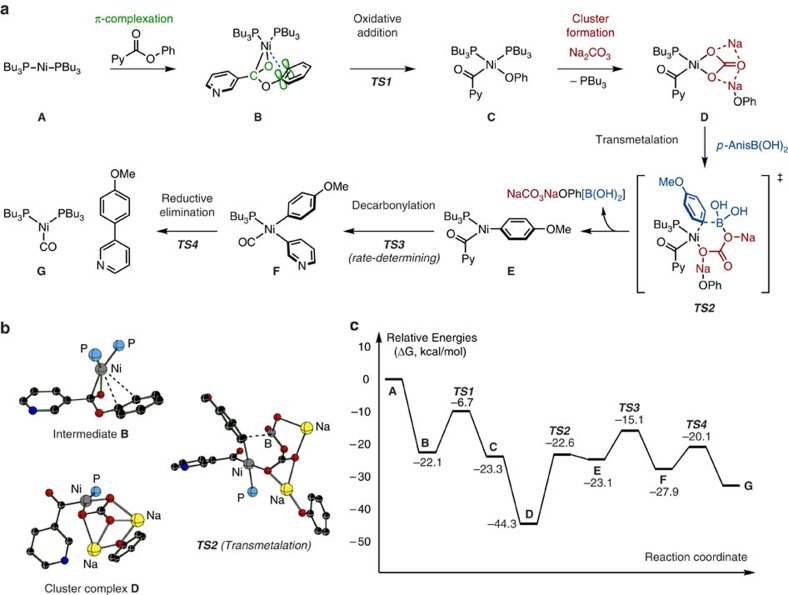
Calculation of nickel-catalysed reaction. (**a**) Schematic presentation of the elementary steps of the decarbonylative coupling of phenyl 3-pyridinecarboxylate and *p*-anisylboronic acid in the presence of Ni-P(*n*Bu)_3_ and Na_2_CO_3_. (**b**) The important reactive intermediates **B** and **D**, and transmetalation transition state ***TS2***. Hydrogen atoms and *n*-butyl groups on phosphorous atoms are omitted for clarity. (**c**) Relative energies of representative intermediates and transition states. The presented Gibbs free relative energies (Δ*G*) are obtained at the M06/{Lanl2dz_Ni_+[6–31G(d,p)]} level of theory, in toluene solution (by using the polarizable continuum model (PCM) solvation method), and at the experimentally reported temperature (423.15 K) and pressure (1 atm).

Having established a new coupling protocol, we then turned our attention to elucidate key mechanistic features of this decarbonylative Suzuki–Miyaura coupling by comprehensive theoretical calculation. Shown in Fig. 2a is the operative mechanism of the reaction in the presence of base (Na_2_CO_3_). The performed density functional theory calculations (see Supplementary Information and caption of Fig. 2 for details of the used computational methods) show that the initial step of the reaction, that is, C(acyl)–O oxidative addition of phenyl 3-pyridinecarboxylate to Ni[P(*n*-Bu)_3_]_2_ (**A**), proceeds through an intermediate π-complex **B** (Fig. 2b). The existing donor–acceptor interactions between π-bonds of phenyl and pyridyl rings with empty d-orbitals of nickel exist (see Supplementary Fig. 159 for details) in the π-complex **B** is predicted to play a key role in C(acyl)–O bond activation since ethyl esters are virtually unreactive. Computation has shown that the addition of Na_2_CO_3_ to the reaction mixture has almost no impact on the C–O oxidative addition step. In contrast, it has profound effect on the mechanism of the subsequent steps of the reaction (Fig. 2a). At first, Na_2_CO_3_ reacts with the oxidative addition intermediate **C** with almost no energy barrier, and leads to the formation of the unprecedented Ni-[NaCO_3_NaOPh] cluster complex **D** (Fig. 2b)^24^,^25^,^35^,^36^,^37^. Overall reaction, **C**+Na_2_CO_3_→**D**, is found to be exergonic by 22.2 kcal mol^−1^.

Although there was an important question from the outset of this work whether the subsequent step is transmetalation or decarbonylation, the calculations uncovered that the former is the case. Transmetalation of **D** with *p*-anisylboronic acid occurs to give complex **E** with an energy barrier of 22.3 kcal mol^−1^ (Fig. 2c). The following decarbonylation occurs with higher barrier of 29.2 kcal mol^−1^ to give intermediate **F**, which then undergoes smooth reductive elimination to furnish the decarbonylative cross-coupling product and nickel(0) species. Overall, the decarbonylation is a rate-determining step of the present coupling under the optimized conditions with Na_2_CO_3_. It should be noted that the calculations have also determined a slight high-energy reaction pathway in the absence of base, in which the rate-determining step is transmetalation from **C** (barrier 31.9 kcal mol^−1^). Thus, the calculations shed light on why this reaction occurs without base, yet is somewhat accelerated on the addition of Na_2_CO_3_ (Table 1).

### Scope of decarbonylative organoboron cross-coupling of esters

After establishing optimal catalytic conditions [Ni(OAc)_2_, P(*n*-Bu)_3_, Na_2_CO_3_ and toluene, 150 °C, 24 h] and elucidating mechanistic details, we performed the decarbonylative cross-coupling with a wide range of substrates. As shown in Table 2, the scope is very broad with regard to both partners. Various electronically and sterically diverse arenecarboxylic acid esters were found to cross-couple with *p*-anisylboronic acid (**2a**) in high to excellent yields. Esters of heterocycles such as thiophenes, furans, benzothiophenes, oxazoles, thiazoles, pyridines and quinolines reacted smoothly to provide the corresponding heterobiaryls. Apart from 2-pyridyl, 2-pyrazyl and 2-quinolinyl groups, most heteroaryl groups were transferred into the product in high to excellent yields. Easy access to heteroarene coupling partners is worth mentioning. For example, esters of oxazoles and thiazole **1Q**–**1S** could be prepared easily, while several steps are needed for the synthesis of halogen analogues of these coupling partners. The present decarbonylative cross-coupling is also applicable to the synthesis of flavone and its derivatives (**3ABh** and **3ABa**), which are of significant scientific and public interest. Halide-containing substrates, except for organofluorines, are problematic as aryl–halogen bonds are also activated and arylated by the Ni(OAc)_2_/P(*n*-Bu)_3_ catalyst. By competitive experiments, we estimated the reactivity order as Ar–I≥Ar–Br>Ar–Cl≥Ar–CO_2_Ph⩾Ar–F (see Supplementary Table 5). The scope of this reaction with respect to arylboronic acids is also very broad. In addition to various substituted phenyl groups including sterically hindered *o*-disubstituted groups (**3Am**), heteroarene and polycyclic aromatic hydrocarbon moieties (**3Ao** and **3Ap**) could also be coupled. Moreover, alkenylboronic acids also participated well in the decarbonylative cross-coupling to afford the corresponding substituted alkenes in good yields (**3Wq** and **3Aq**), when NaCl was employed as an additive (see Supplementary Table 6). The effect of NaCl or related salt remains unclear, but one possibility might be that it works as a dehydrating agent for the water that is generated from arylboronic acids. As mentioned above, the present coupling is selective for phenol-derived esters. Thus, methyl esters in either of the coupling partners are left intact (**3Fa**, **3Za** and **3Ai**). It should also be highlighted that the present coupling has been conducted on a gram scale in high yield (**3Aa**: 1.44 g, 78%).

In this study, we primarily focused on the development of biaryl- and heterobiaryl-forming reactions in the context of accessing privileged structures in a useful and unconventional way. Broadening the scope of present catalysis to an aliphatic system (*sp*^3^-*sp*^2^ cross-coupling) would have significant impact in synthetic chemistry. Though still preliminary, we found that this coupling is feasible (Table 2). When arylacetic acid phenyl esters **1AF**–**1AH** were treated with **2a** under the standard conditions, the corresponding decarbonylative coupling products (**3AFa**, **3AGa** and **3AHa**) were produced. The addition of a catalytic amount of DMAP (*N*,*N*-dimethylaminopyridine) to the reaction system had a positive effect on the coupling reaction (see Supplementary Table 7). Although there is room for further optimization of this particular coupling partner, this successful alkyl–aryl cross-coupling speaks well for the potential of our present nickel catalysis with regard to the development of unconventional yet useful synthetic chemistry.

## Discussion

**Figure 3:**
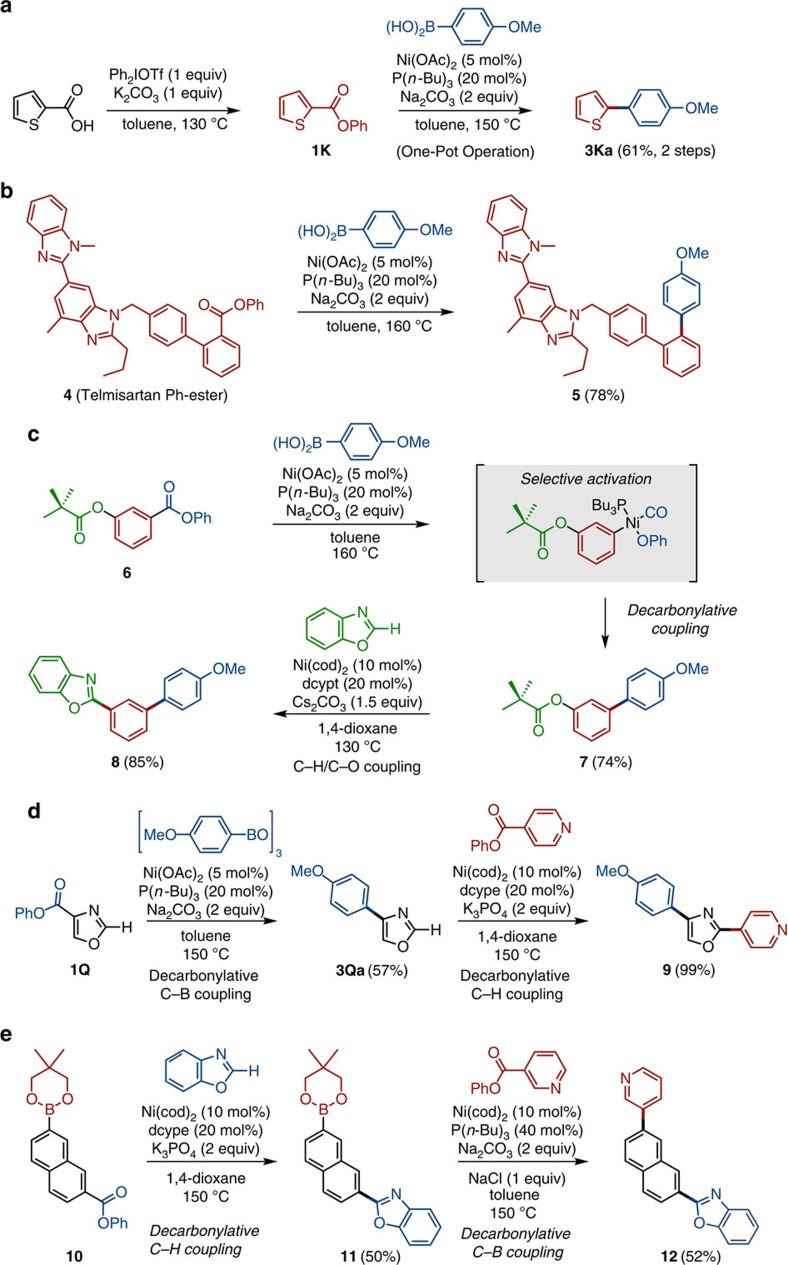
Further applications of ‘ester' Suzuki–Miyaura coupling. (**a**) One-pot transformation of thiophene-2-carboxylic acid to biaryl **3Ka**. (**b**) Application to the synthesis of telmisartan derivatives. (**c**) Orthogonal coupling of **6**; decarbonylative cross-coupling catalysed by Ni(OAc)_2_/P(*n*-Bu)_3_ (first step) and C–H/C–O coupling catalysed by Ni(cod)_2_/dcypt (second step). dcypt, 3,4-bis(dicyclohexylphosphino)thiophene. (**d**) Sequential coupling of **1Q**; decarbonylative C–B coupling catalysed by Ni(OAc)_2_/P(*n*-Bu)_3_ (first step) and decarbonylative C–H coupling catalysed by Ni(cod)_2_/dcype (second step). (**e**) Orthogonal coupling of **10**: decarbonylative C–H coupling catalysed by Ni(cod)_2_/dcype (first step) and decarbonylative C–B coupling catalysed by Ni(OAc)_2_/P(*n*-Bu)_3_ (second step).

While highly chemoselective coupling at the phenol-derived ester moiety is an advantage of the present protocol, there is a need to transform a molecule of interest into an activated phenyl ester form. To simplify this pre-activation step, we have also established a one-pot protocol starting directly from carboxylic acids (Fig. 3a). For example, 2-thiophenecarboxylic acid could be converted to the corresponding phenyl ester **1K** by the treatment with diphenyl iodonium triflate (Ph_2_IOTf) and K_2_CO_3_ in toluene at 130 °C for 2 h^38^. After removing iodobenzene (co-product of esterification) under reduced pressure, **2a**, nickel catalyst and Na_2_CO_3_ were added to the same reaction flask, and the corresponding decarbonylative cross-coupling product **3Ka** was obtained in 61% yield (two steps) after stirring the mixture at 150 °C.

The nickel-catalysed decarbonylative cross-coupling was also viable with complex molecular precursors. For example, telmisartan, an angiotensin blocker that is under use for treatment of hypertension^39^, could be subjected to decarbonylative cross-coupling with **2a** (after esterification) to produce aryl analogue **5** in 78% yield (Fig. 3b). The successful coupling of heterocycle-rich compound **4** bodes well for the high versatility of present catalysis in many molecular transformations particularly in complex molecule synthesis.

The molecular recognition ability and chemoselectivity of the Ni(OAc)_2_/P(*n*-Bu)_3_ catalyst is exceptionally high (Fig. 3c). 3-Hydroxybenzoic acid derivative **6**, in which the phenol end and the acid end are protected as a pivalate and phenyl ester, respectively, provides an interesting example. This compound underwent selective activation of the latter phenyl ester moiety (highlighted in blue in Fig. 3c) by the Ni(OAc)_2_/P(*n*-Bu)_3_ catalyst, leading to the selective coupling with **2a** to produce **7** in 74% yield. There are a number of reports that nickel complexes catalyse the Suzuki–Miyaura coupling of a range of aryl pivalates and related aryl C–O bonds with arylboronic acids^6^,^40^,^41^,^42^,^43^. However, under the present conditions, aryl pivalate moiety (highlighted in green) remained intact. This result underscores the capability of the Ni(OAc)_2_/P(*n*-Bu)_3_ catalyst to distinguish subtle difference in the steric environment of the two aryl ester moieties. Moreover, we found that the remaining phenyl ester moiety of **7** could be activated by another recently developed nickel catalyst Ni(cod)_2_/dcypt to effect the C–H/C–O coupling reaction with benzoxazole^31^,^34^, yielding teraryl **8** in 85% yield (Fig. 3c).

A sequence of decarbonylative C–B coupling and decarbonylative C–H coupling^22^ is also possible. For example, by the action of Ni(OAc)_2_/P(*n*-Bu)_3_ catalyst, phenyl oxazole-4-carboxylate (**1Q**) reacted with (4-methoxyphenyl)boroxine in a decarbonylative C–B coupling manner to afford aryloxazole **3Qa** in 57% yield (Fig. 3d). The oxazole C–H bond of **3Qa** can be further arylated by decarbonylative C–H coupling with phenyl isonicotinate in the presence of Ni(cod)_2_/dcype catalyst to afford diaryloxazole **9** in 99% yield (Fig. 3d). A sequential coupling of bifunctional aromatic **10** having boron and phenyl ester moieties is also possible (Fig. 3e). In the presence of Ni(cod)_2_/dcype catalyst, **10** underwent decarbonylative C–H coupling with benzoxazole to give **11** in 50% yield. Note that the boronate group of compound **10** was tolerated under these conditions. Subsequently, the boronate group of **11** can be arylated by decarbonylative C–B coupling with phenyl nicotinate by Ni(OAc)_2_/P(*n*-Bu)_3_ catalyst to afford triaryl **12** in 52% yield.

In conclusion, we have developed a user-friendly nickel-based catalytic system (Ni(OAc)_2_/P(*n*-Bu)_3_) for the decarbonylative organoboron cross-coupling using esters as coupling partners. In this report, we also described (1) the elucidation of key mechanistic features of this newly developed reaction by comprehensive theoretical calculation, (2) the broad scope with regard to both coupling partners (esters and boron), (3) successful aliphatic cross-coupling, (4) a gram-scale cross-coupling, (5) one-pot cross-coupling protocol starting directly from carboxylic acids, (6) application to complex molecular settings and (7) exceptionally high molecular recognition ability of Ni(OAc)_2_/P(*n*-Bu)_3_ catalyst that allows unconventional orthogonal cross-coupling. Overall, the new ‘ester' Suzuki–Miyaura coupling described herein not only is useful as an alternative to the standard halide-based cross-coupling, but also allows strategic and unconventional utilization of ubiquitous ester functionalities in chemical synthesis. Further optimization of catalyst and reaction conditions to achieve broader scope and allow lower temperature coupling is ongoing in our laboratory.

## Methods

### General procedure for Ni-catalysed decarbonylative coupling

A 20-ml glass vessel equipped with J. Young O-ring tap containing a magnetic stirring bar and Ni(OAc)_2_·4H_2_O (5.0 mg, 0.020 mmol, 5 mol%) was dried with a heatgun under reduced pressure and filled with N_2_ gas after cooling to room temperature. To this vessel was added phenyl arenecarboxylic acid phenyl ester **1** (0.40 mmol, 1.0 equiv.), arylboronic acid **2** (0.60 mmol, 1.5 equiv.) and Na_2_CO_3_ (84.8 mg, 0.8 mmol, 2.0 equiv.). The vessel was vacuumed and refilled with N_2_ gas three times. To this was added P(*n*-Bu)_3_ (19.0 μl, 0.08 mmol, 20 mol%) and toluene (1.6 ml). The vessel was sealed with O-ring tap and then heated at 150 °C for 24 h in an eight-well reaction block with stirring. After cooling the reaction mixture to room temperature, the mixture was passed through a short silica-gel pad with EtOAc. The filtrate was concentrated and the residue was purified by silica-gel chromatography to afford the corresponding cross-coupling product **3**.

## Additional information

**How to cite this article**: Muto, K. *et al.* Decarbonylative organoboron cross-coupling of esters by nickel catalysis. *Nat. Commun.* 6:7508 doi: 10.1038/ncomms8508 (2015).

## Supplementary Material

Supplementary Information Supplementary Figures 1-158, Supplementary Tables 1-13, Supplementary Methods and Supplementary References

## Figures and Tables

**Figure 1:**
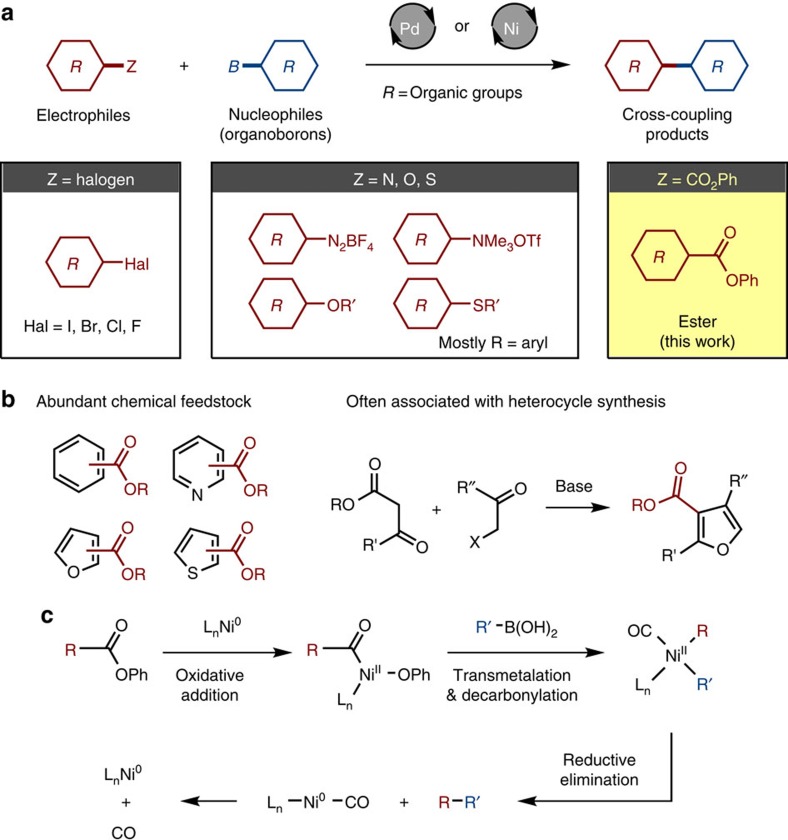
Esters as electrophiles in the Suzuki–Miyaura coupling. (**a**) Electrophiles (R–Z) in the Suzuki–Miyaura coupling with organoboron compounds catalysed by palladium or nickel; *Z*=halogen (standard), *Z*=N, O, S (emerging), *Z*=CO_2_Ph (esters; this work). (**b**) Advantages of using esters as electrophilic coupling partners in Suzuki–Miyaura coupling. (**c**) Our mechanistic blueprint of decarbonylative cross-coupling of esters and boronic acids catalysed by nickel.

**Table 1:**
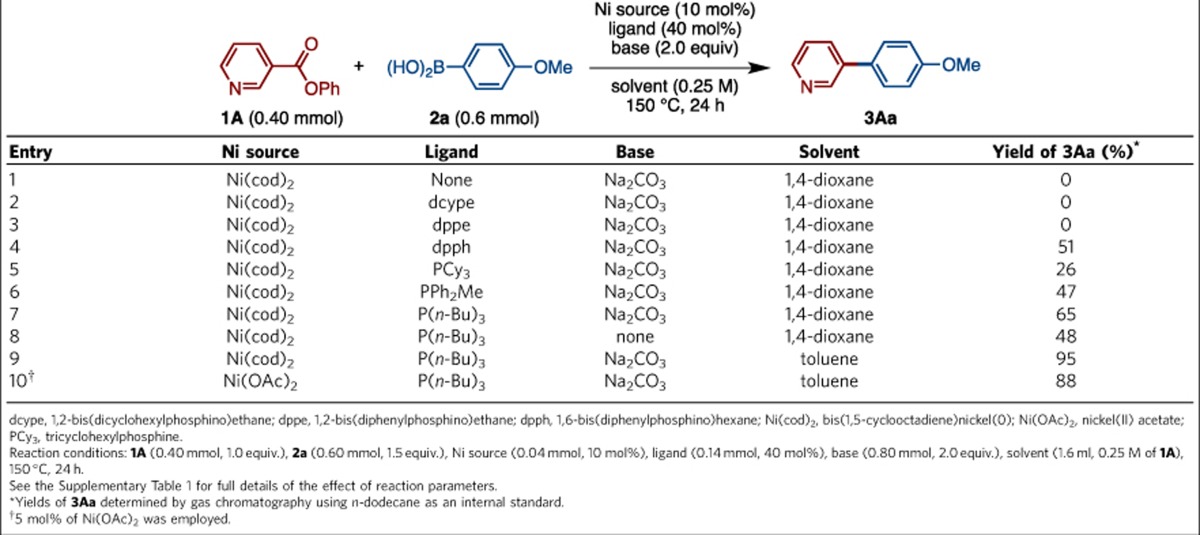
Effect of parameters in the nickel-catalysed decarbonylative cross-coupling of **1A** and **2a**.

**Table 2:**
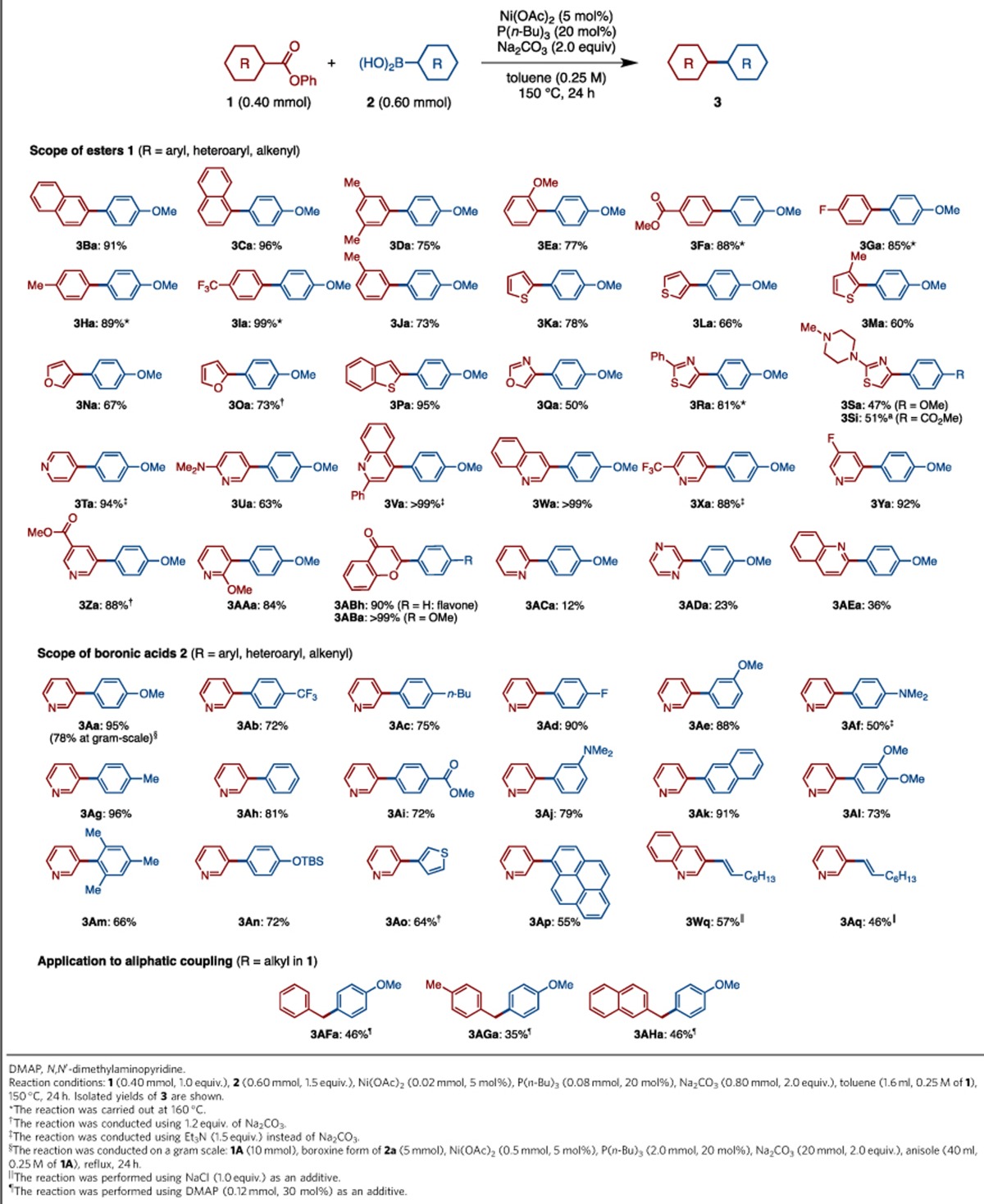
Scope of decarbonylative cross-coupling catalysed by Ni(OAc)_2_/P(*n*-Bu)_3_.
