# Supplementary material for: Decarbonylative organoboron cross-coupling of esters by nickel catalysis
Source: Nat Commun. 2015 Jun 29;6:7508. doi: 10.1038/ncomms8508 (PMC4491840; doi:10.1038/ncomms8508)
Supplement: Supplementary Information — Supplementary Figures 1-158, Supplementary Tables 1-13, Supplementary Methods and Supplementary References [file ncomms8508-s1.pdf]

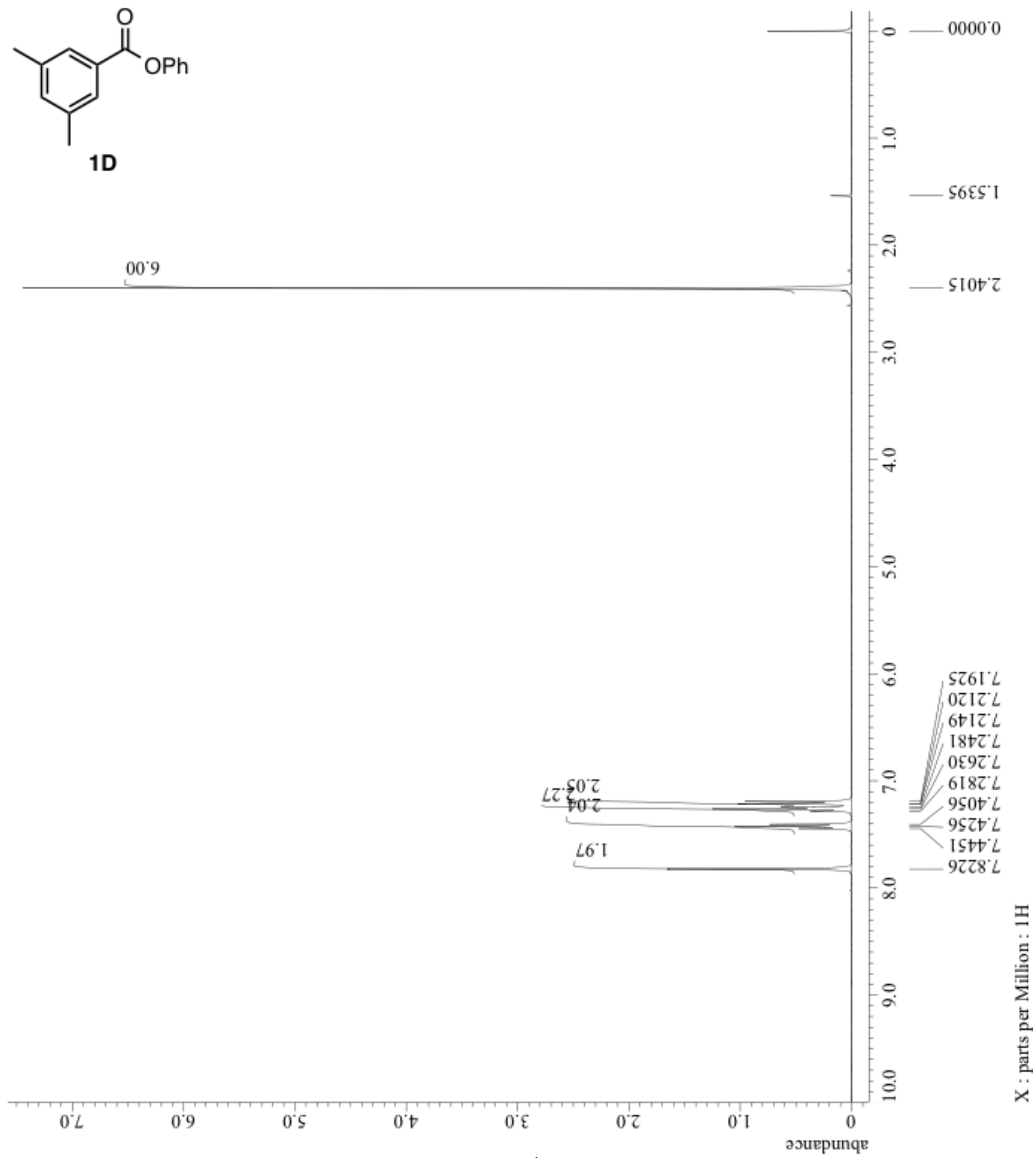


**Supplementary Figure 1. $^{1}$H NMR (400 MHz, $CDCl_3$) of 1D**

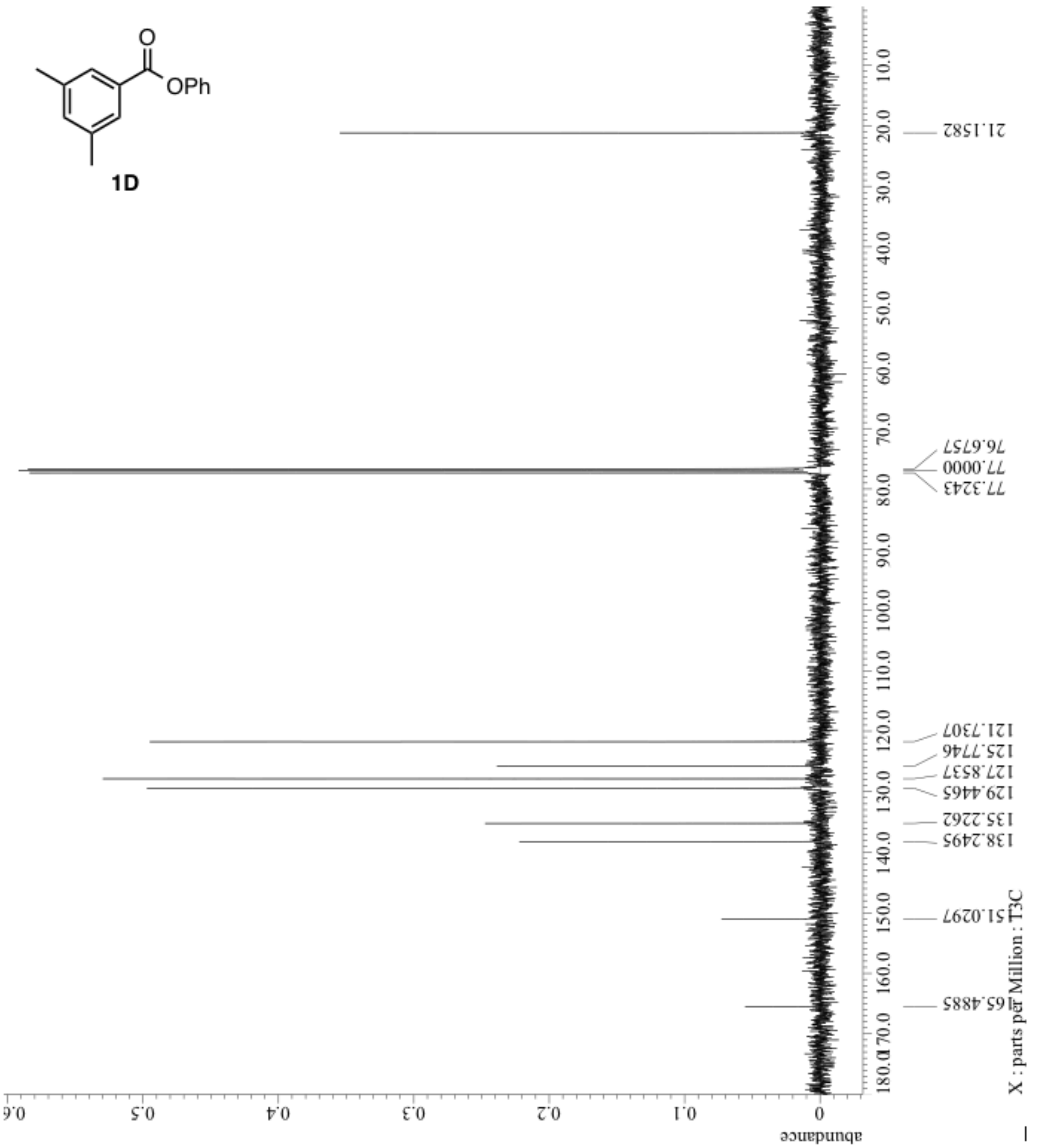


**Supplementary Figure 2. $^{13}$C NMR (100 MHz, $CDCl_3$) of 1D**

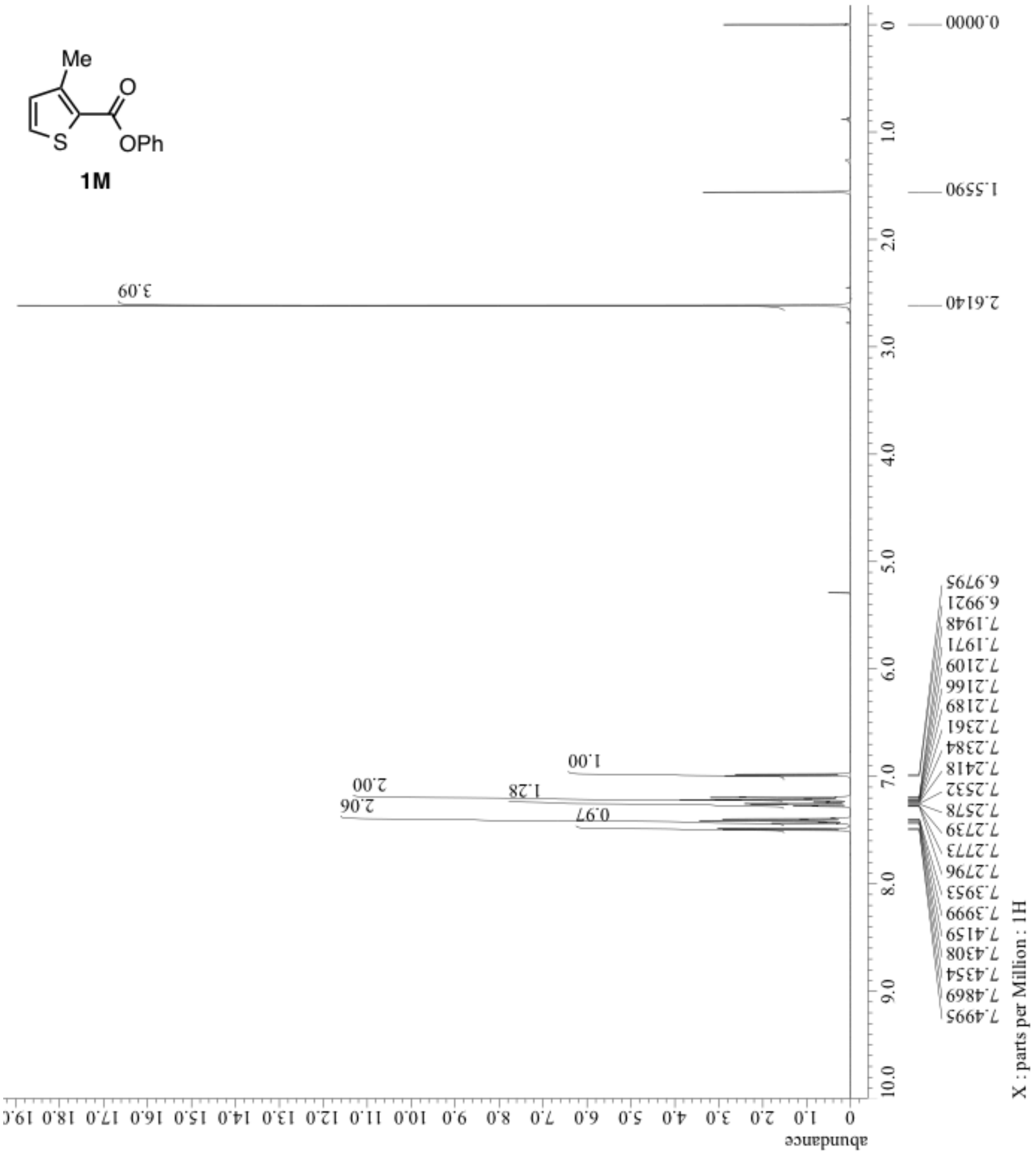


**Supplementary Figure 3. $^{1}$H NMR (400 MHz, $CDCl_3$) of 1M**

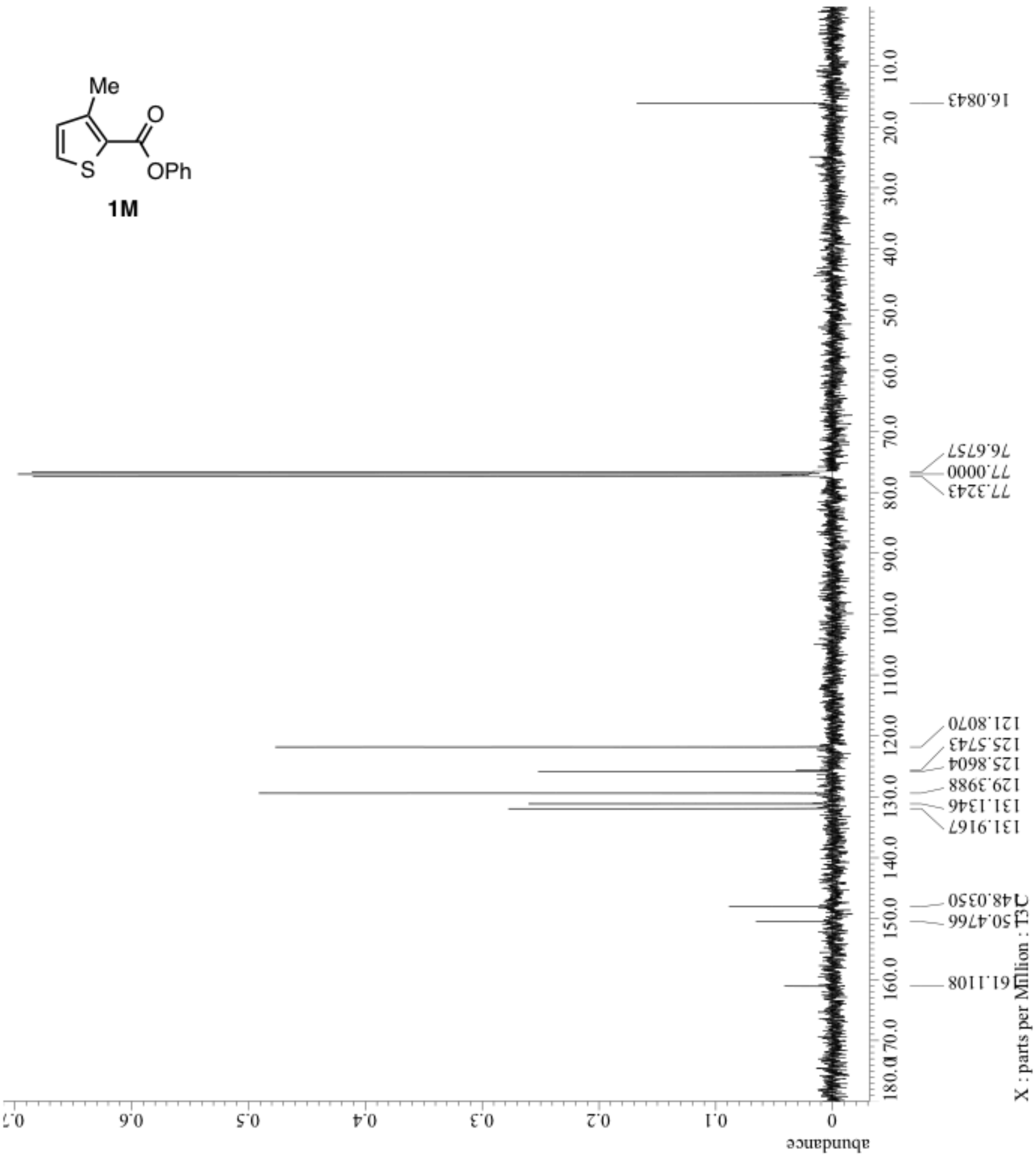


**Supplementary Figure 4. $^{13}$C NMR (100 MHz, $CDCl_3$) of 1M**

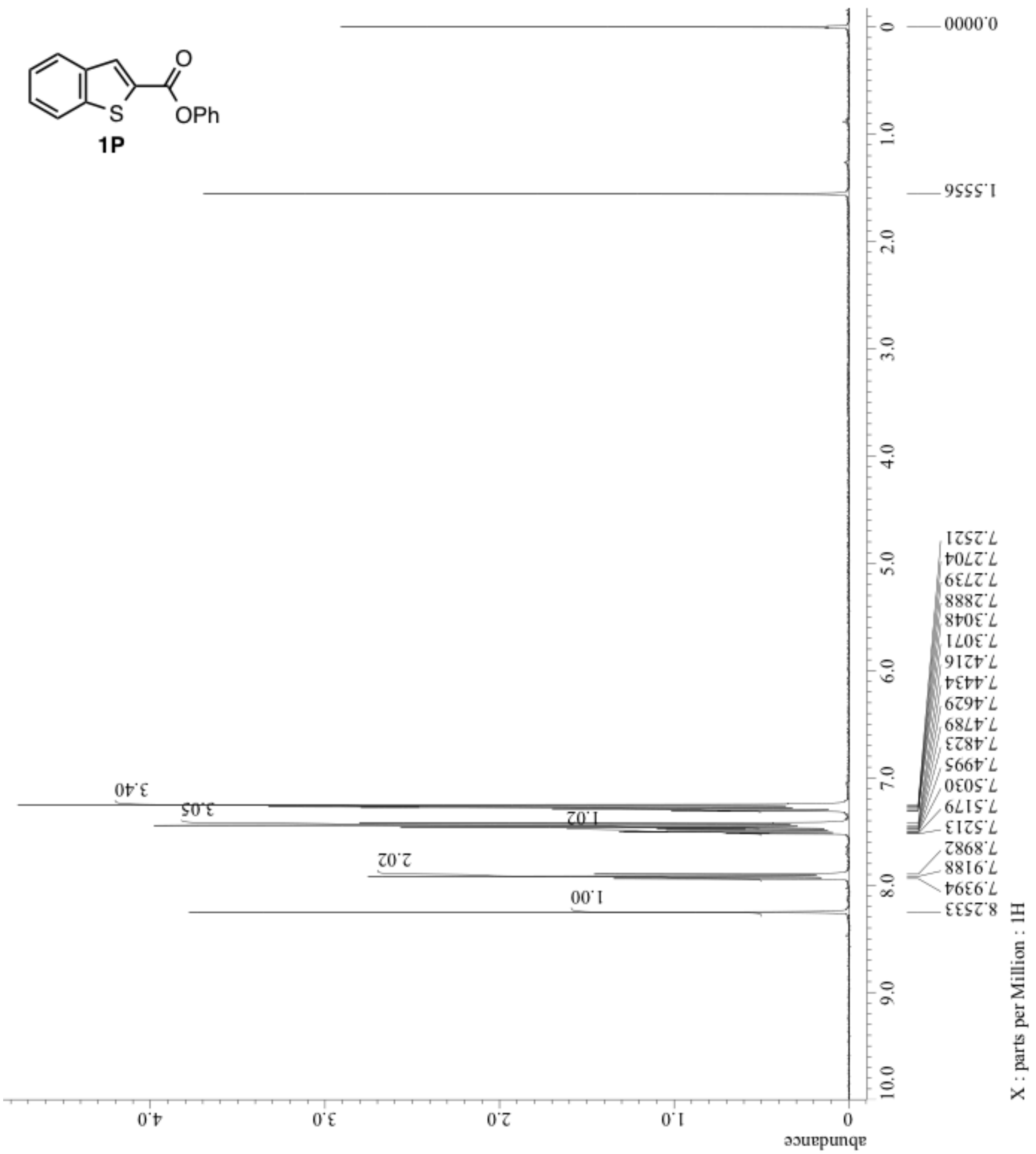


**Supplementary Figure 5.** $^{1}$**H NMR (400 MHz,** $CDCl_3$**) of 1P**

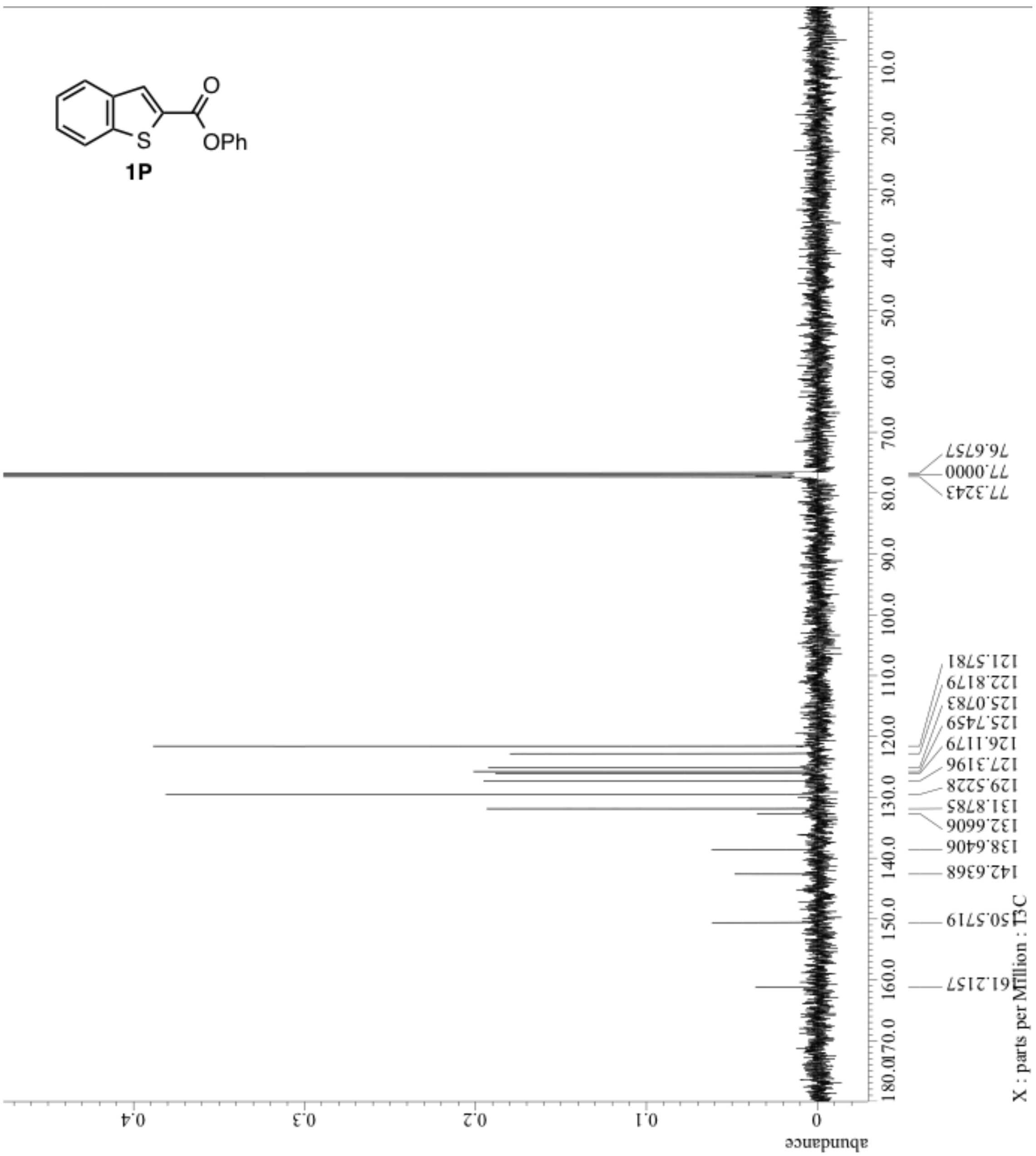


**Supplementary Figure 6. $^{13}$C NMR (100 MHz, $CDCl_3$) of 1P**

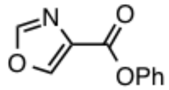

**1Q**

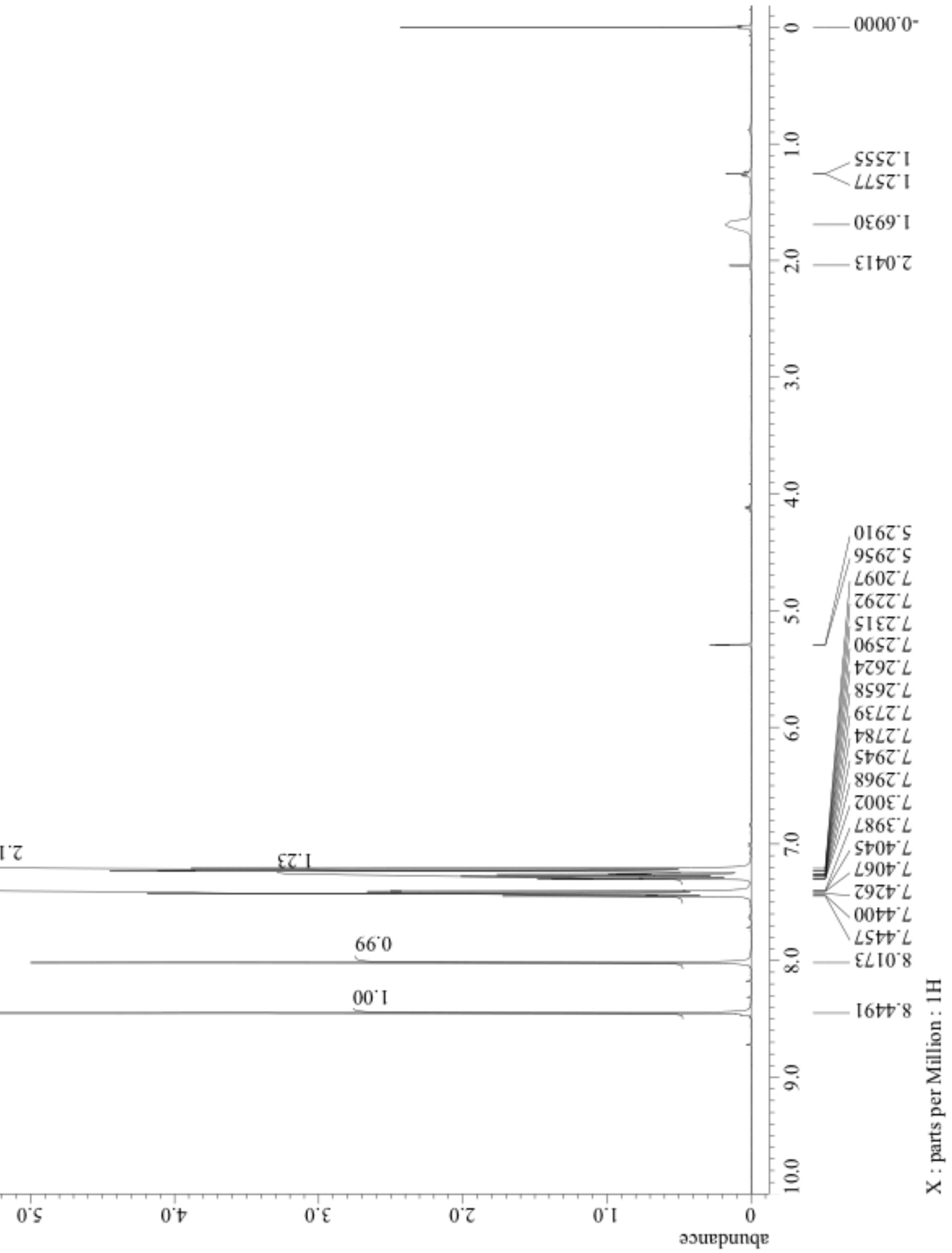


**Supplementary Figure 7.** $^1$**H NMR (400 MHz, CDCl$_3$) of 1Q**

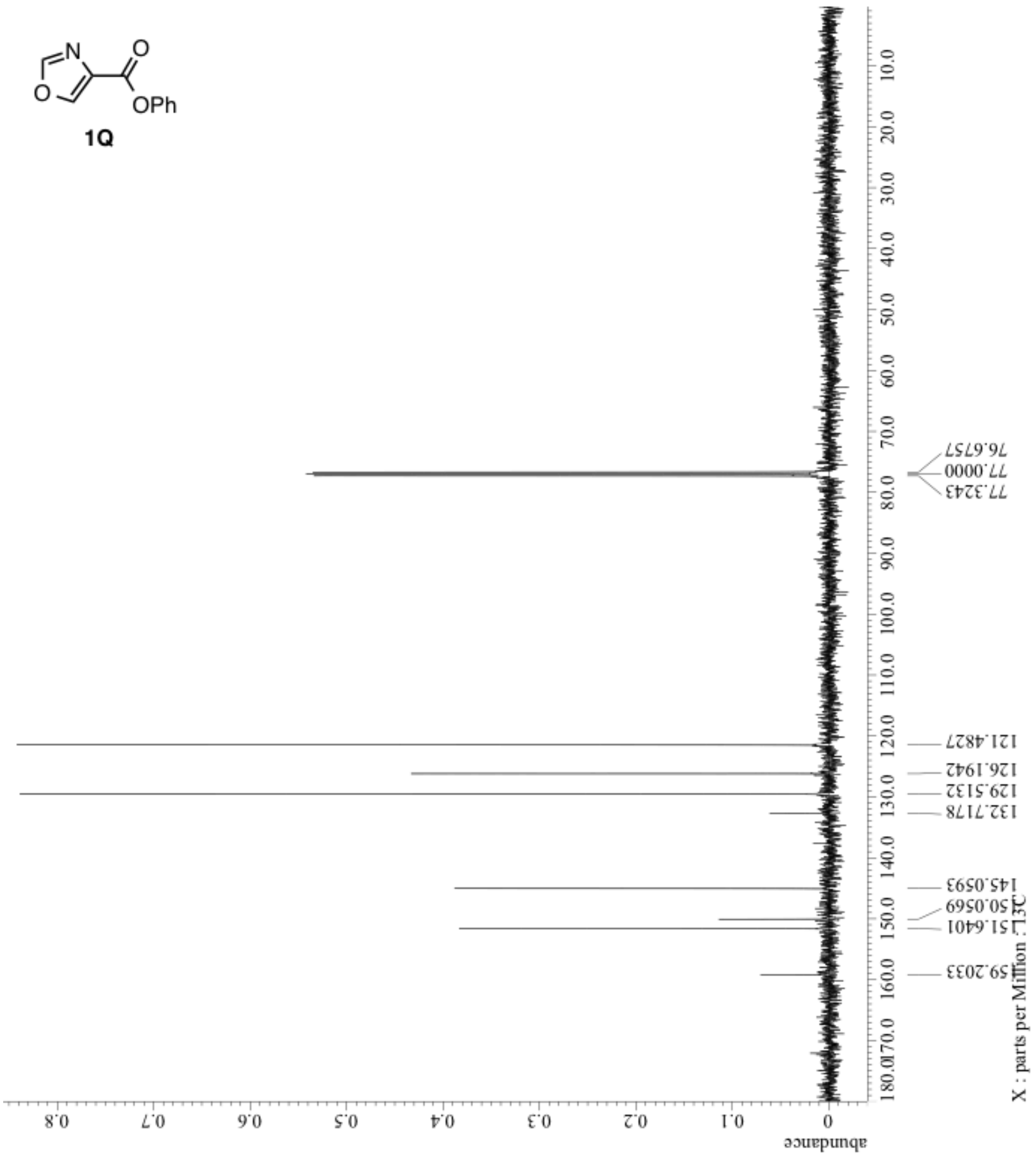


**Supplementary Figure 8. $^{13}C$ NMR (100 MHz, $CDCl_3$) of 1Q**

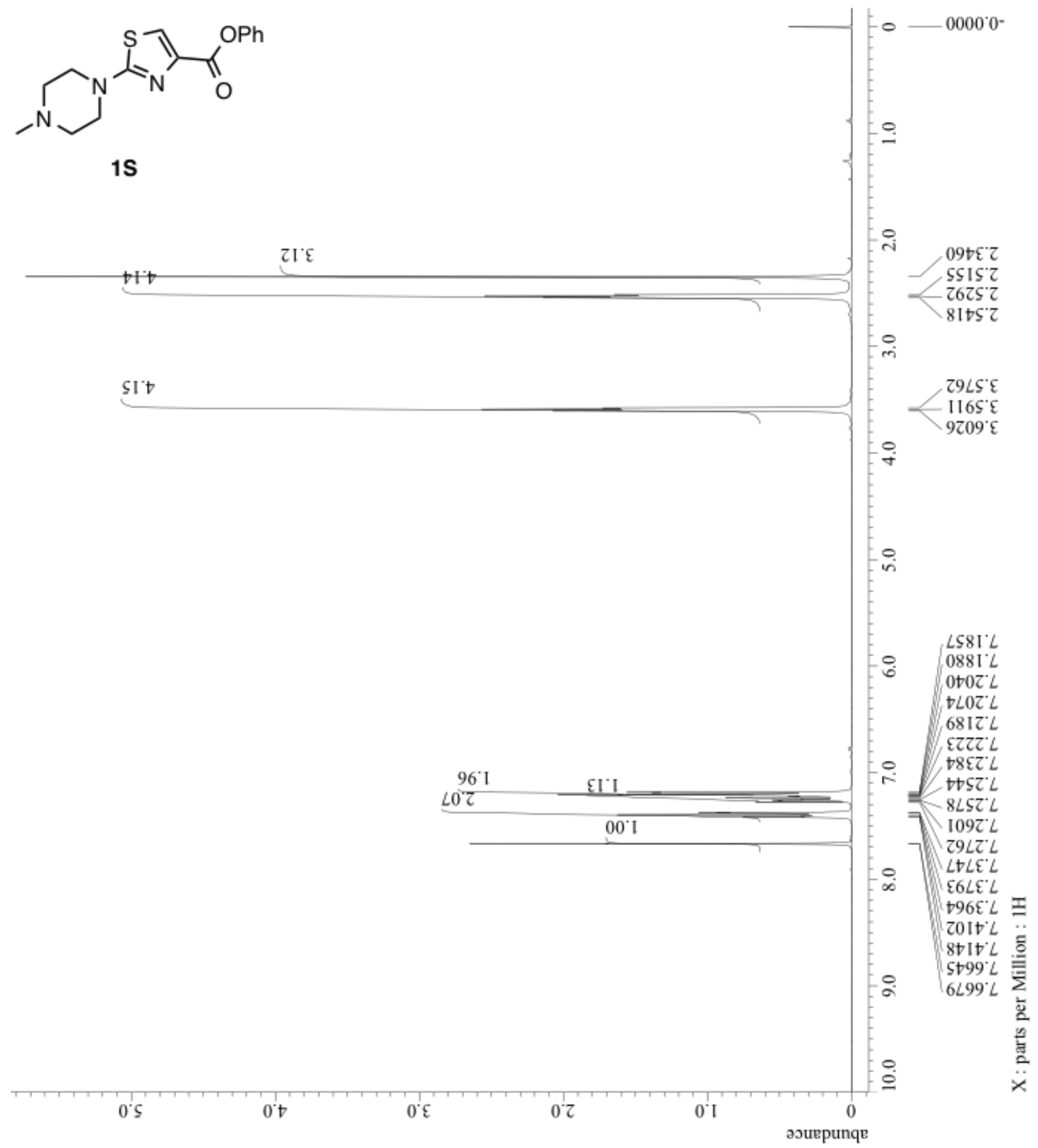


**Supplementary Figure 9. $^{1}H$ NMR (400 MHz, $CDCl_3$) of 1S**

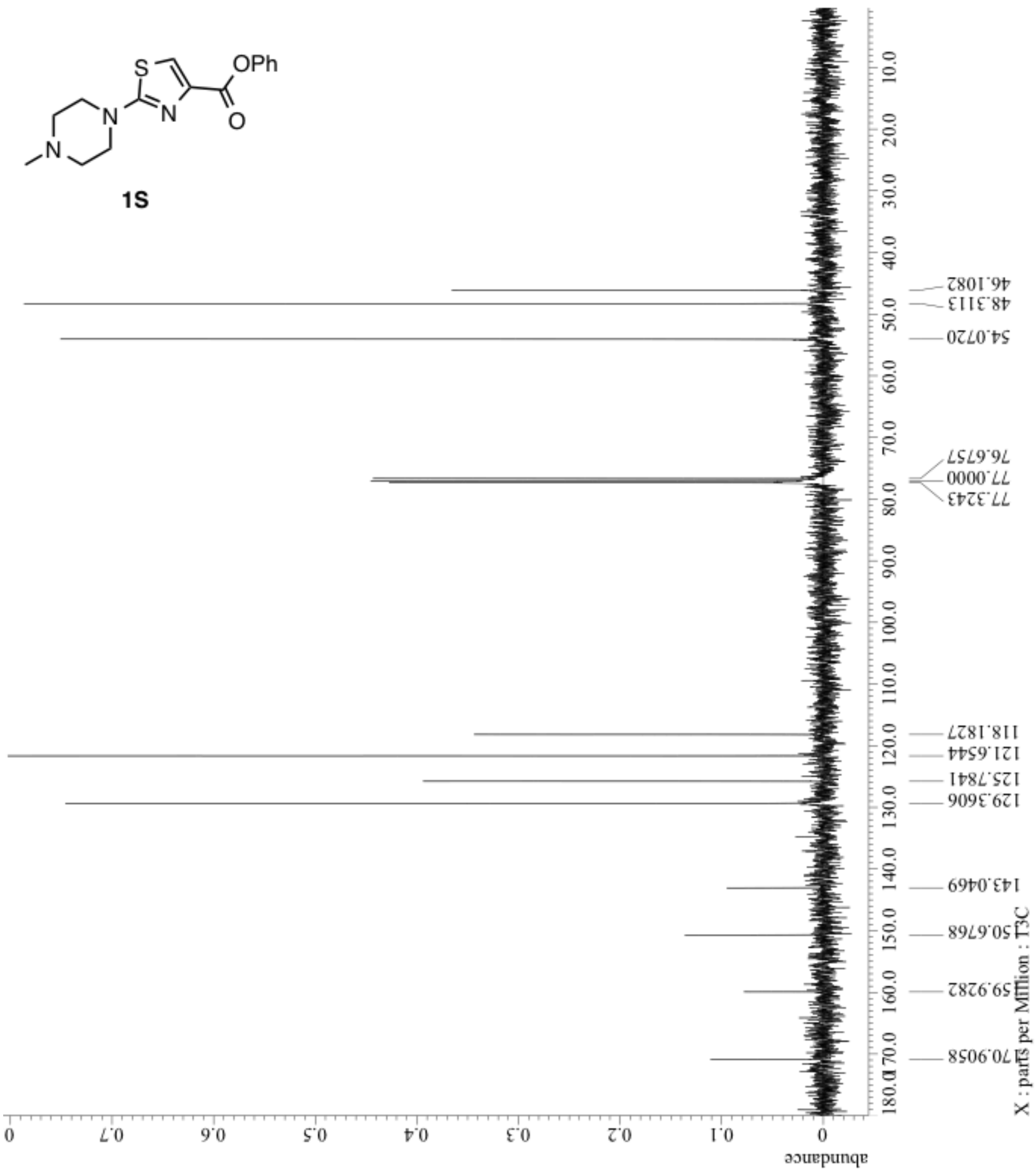


**Supplementary Figure 10. $^{13}C$ NMR (100 MHz, $CDCl_3$) of 1S**

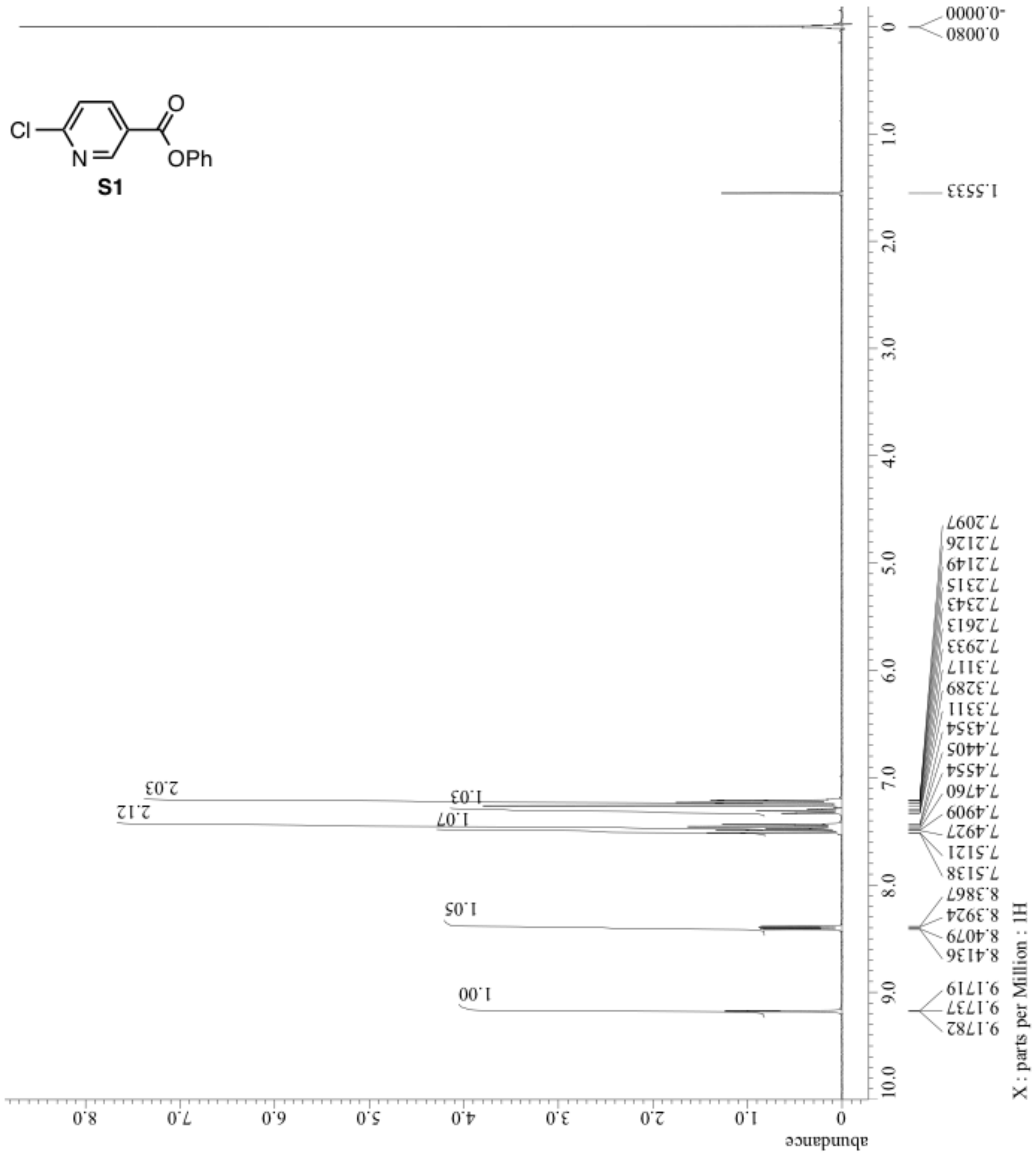


**Supplementary Figure 11. $^1$H NMR (400 MHz, $CDCl_3$) of S1**

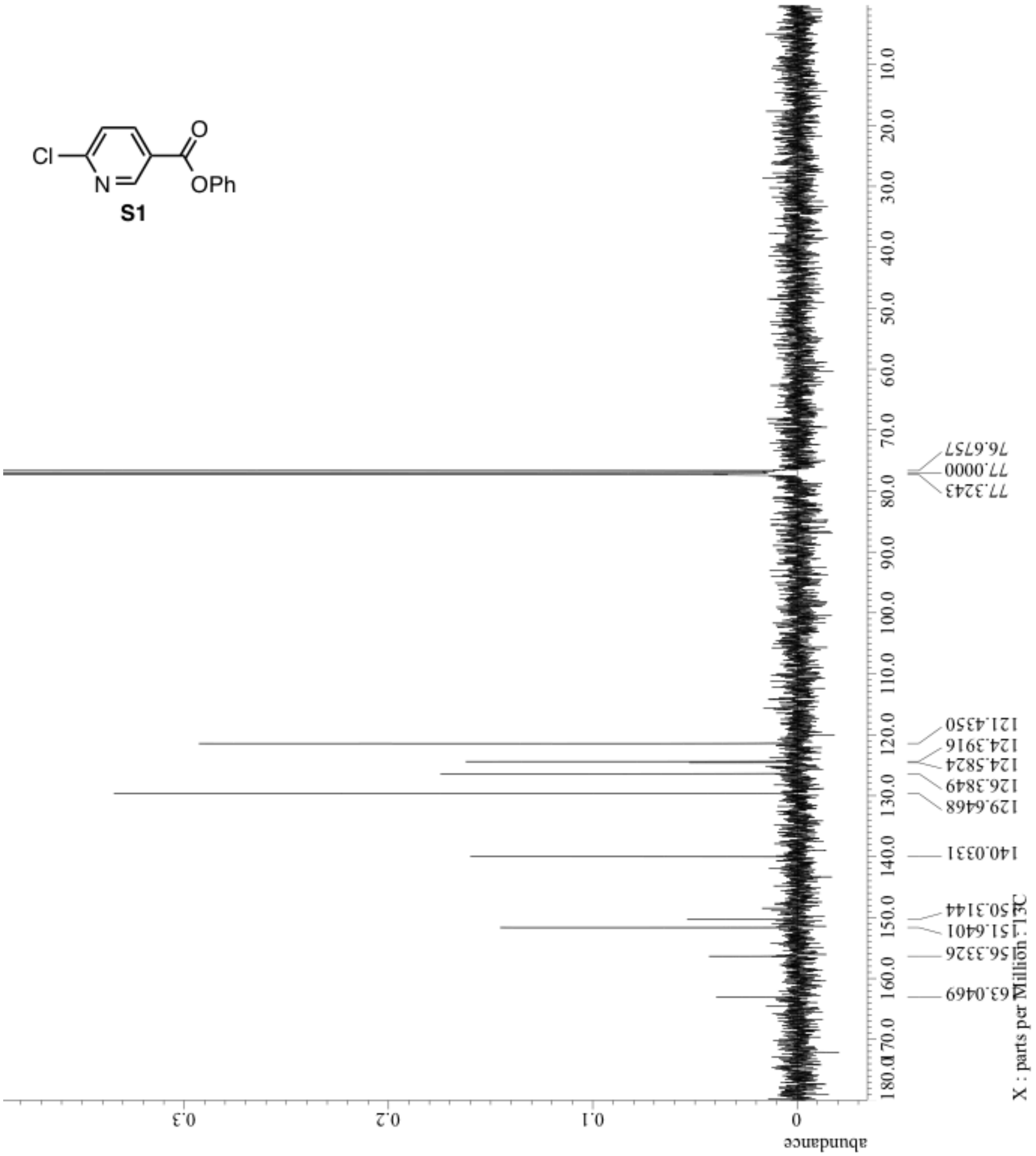


**Supplementary Figure 12. $^{1}$H NMR (100 MHz, $CDCl_3$) of S1**

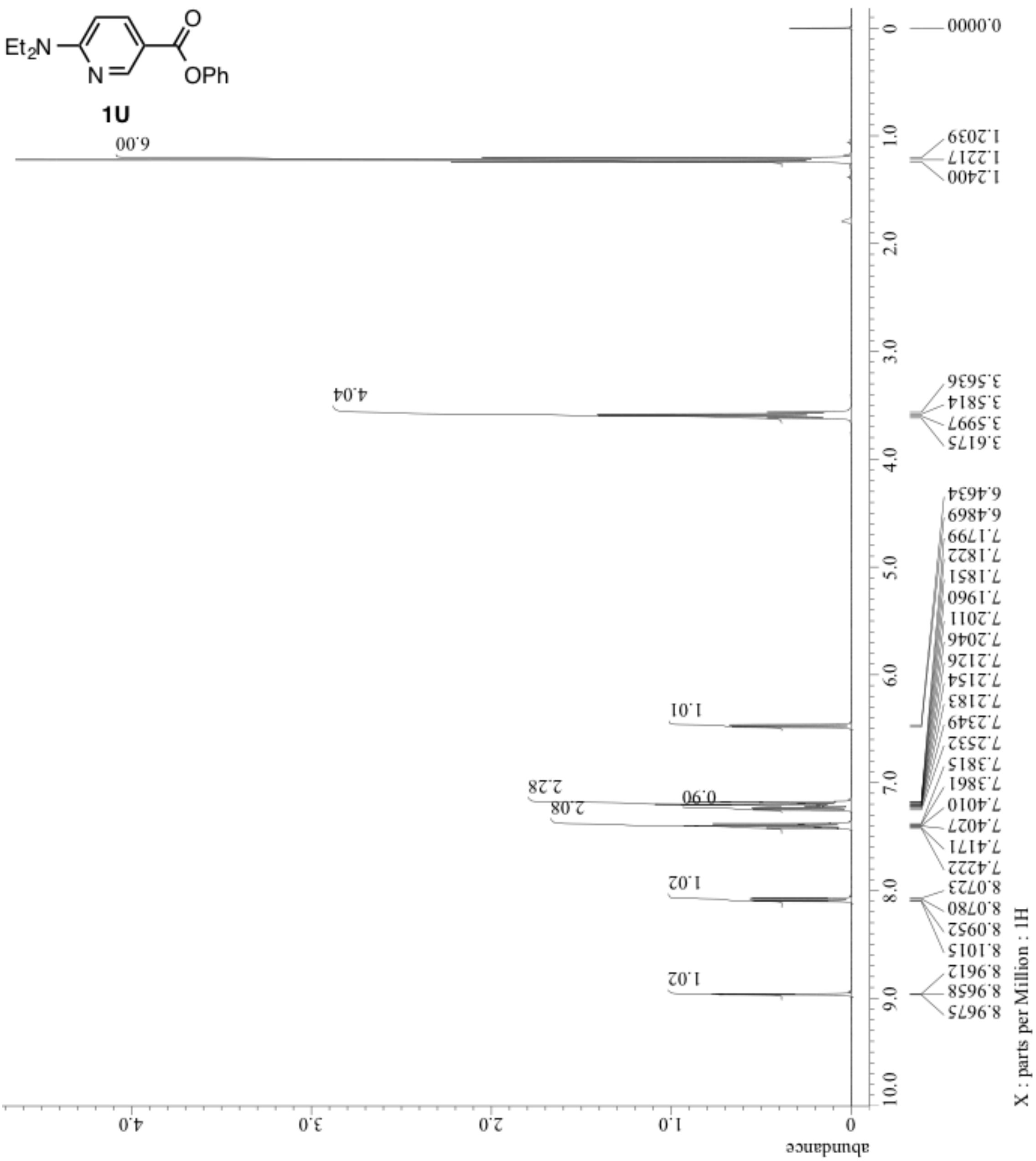


**Supplementary Figure 13. $^{1}$H NMR (400 MHz, $CDCl_3$) of 1U**

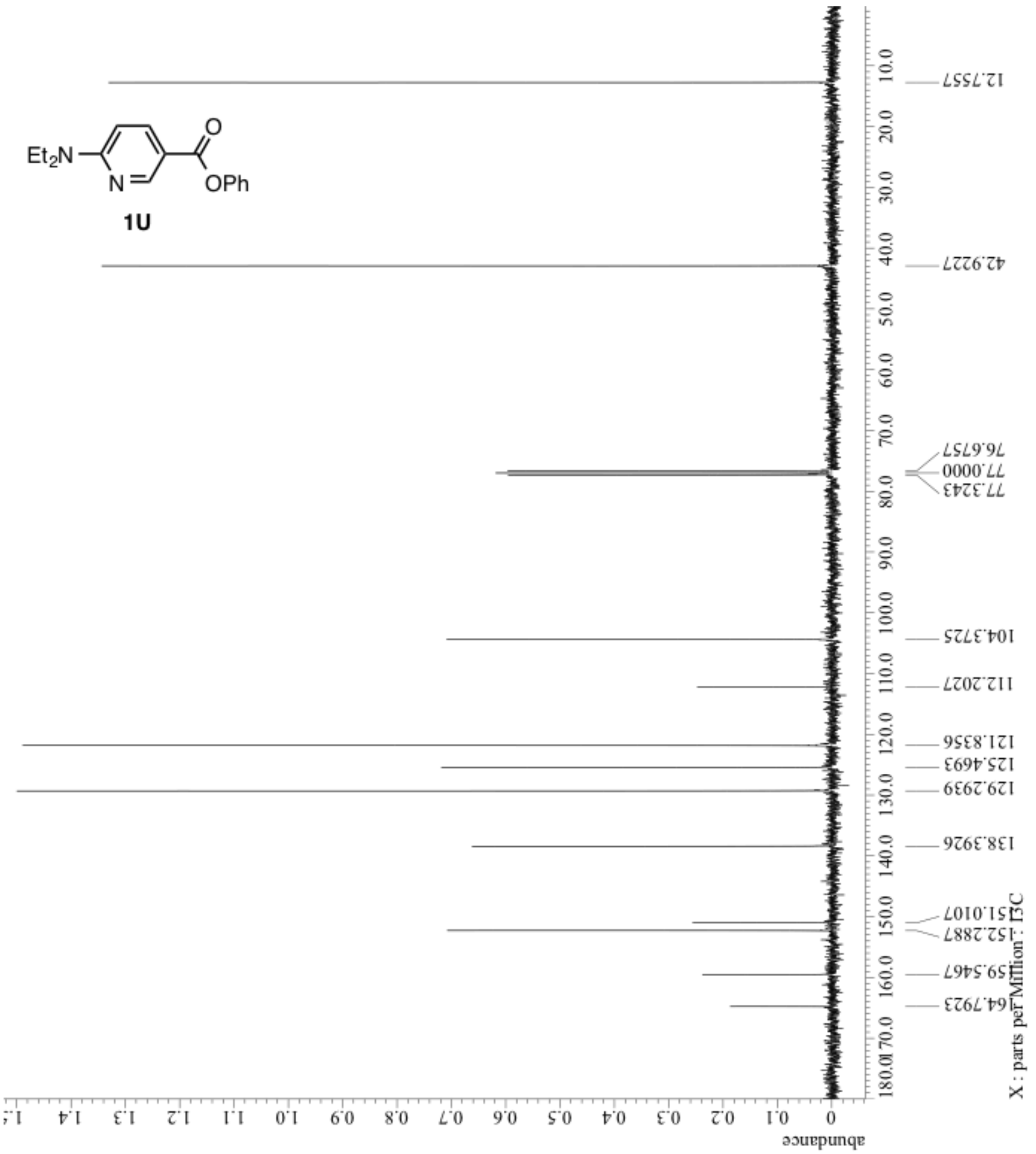


**Supplementary Figure 14. $^{13}$C NMR (100 MHz, $CDCl_3$) of 1U**

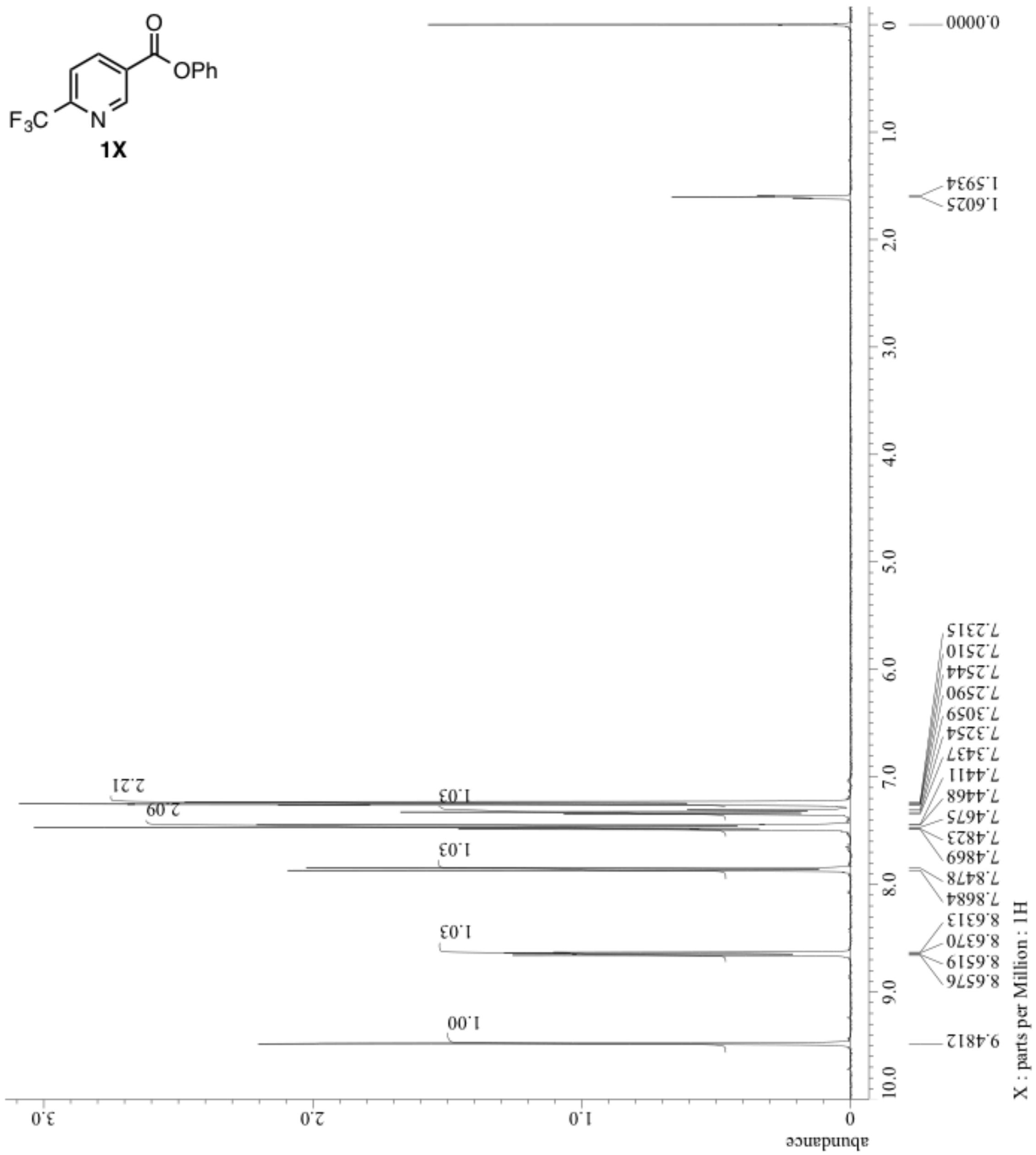


**Supplementary Figure 15. $^{1}H$ NMR (400 MHz, $CDCl_3$) of 1X**

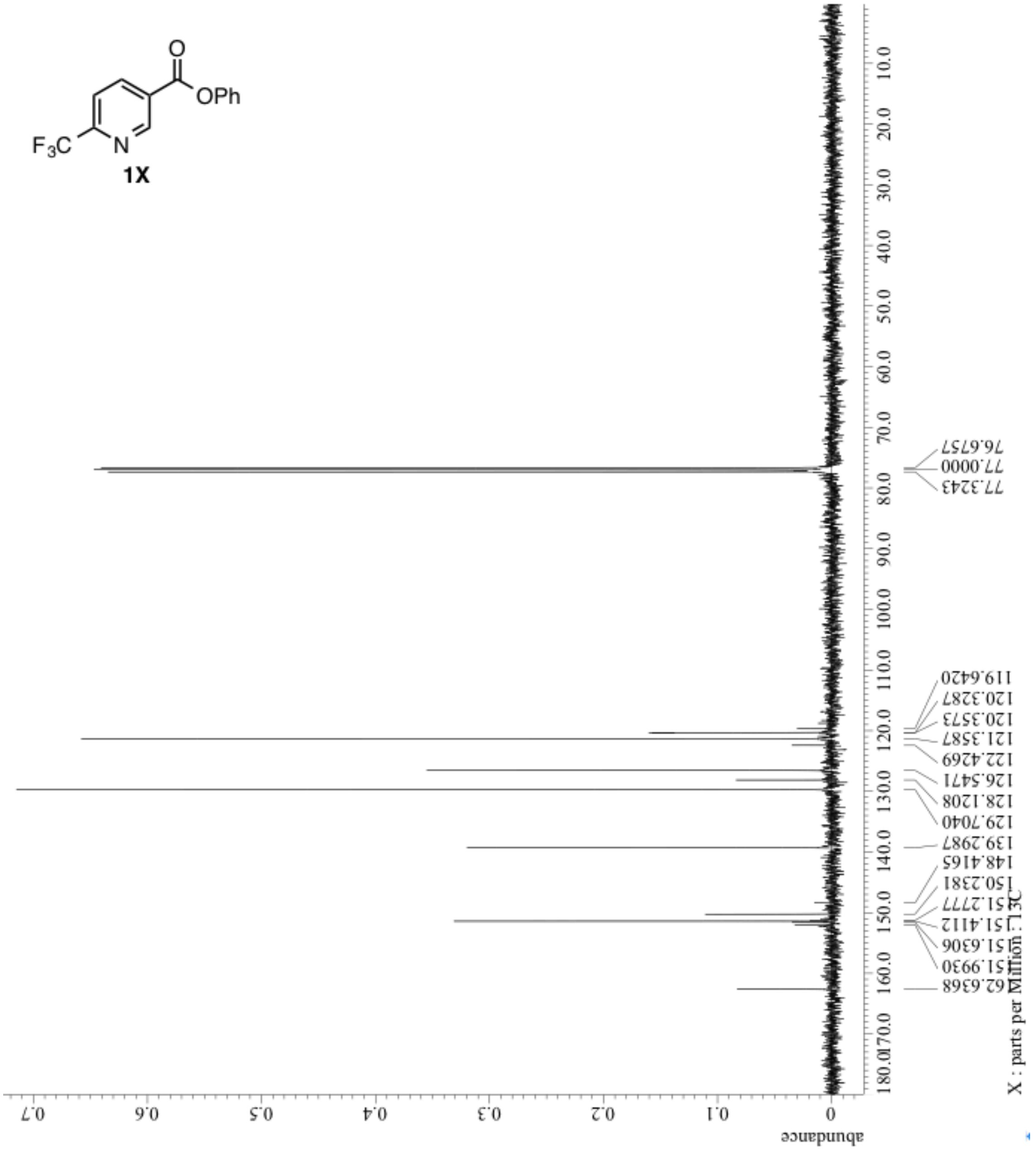


**Supplementary Figure 16. $^{13}C$ NMR (100 MHz, $CDCl_3$) of 1X**

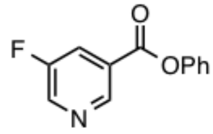


**1Y**

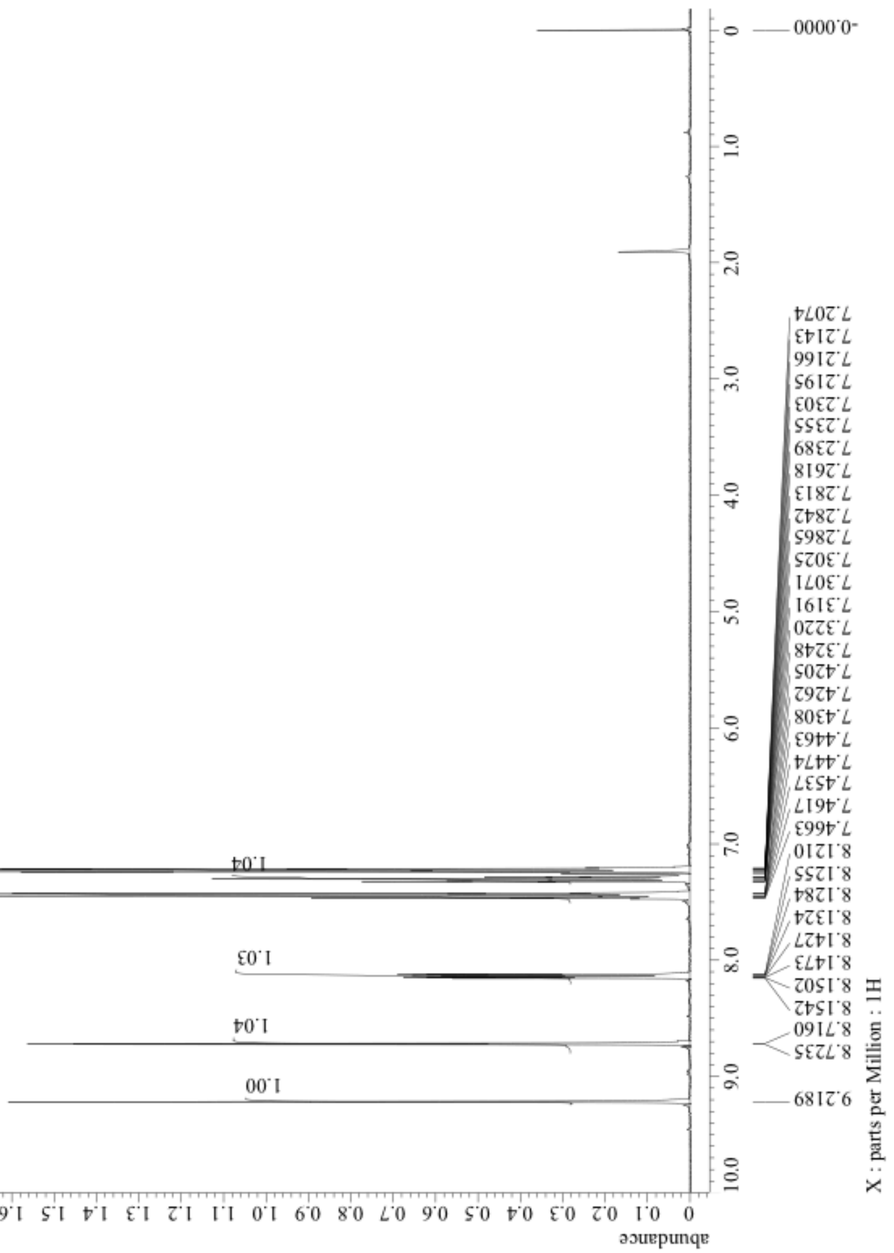


**Supplementary Figure 17. $^{1}$H NMR (400 MHz, $CDCl_3$) of 1Y**

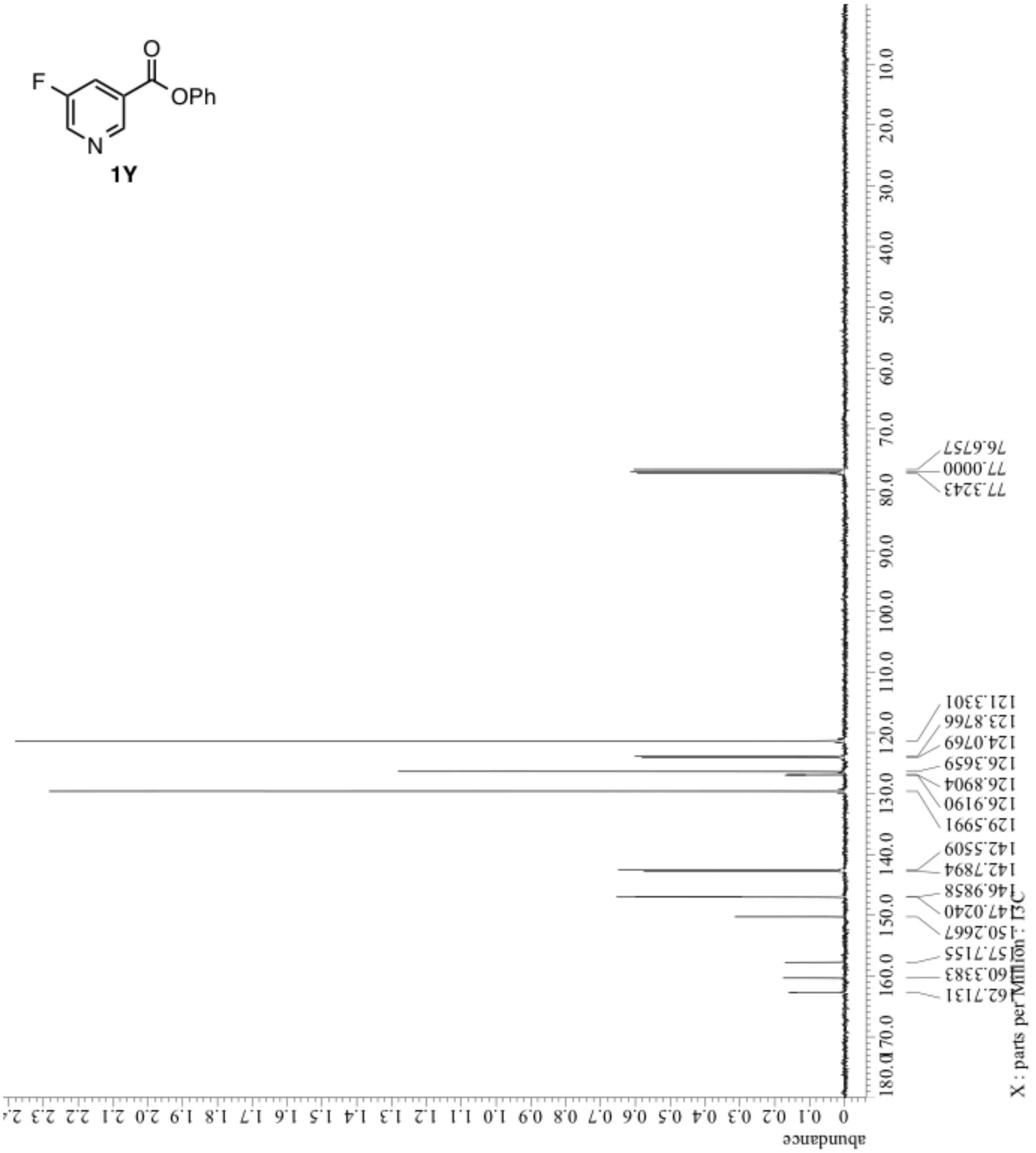


**Supplementary Figure 18.** $^{13}$**C NMR (100 MHz, CDCl**$_3$**) of 1Y**

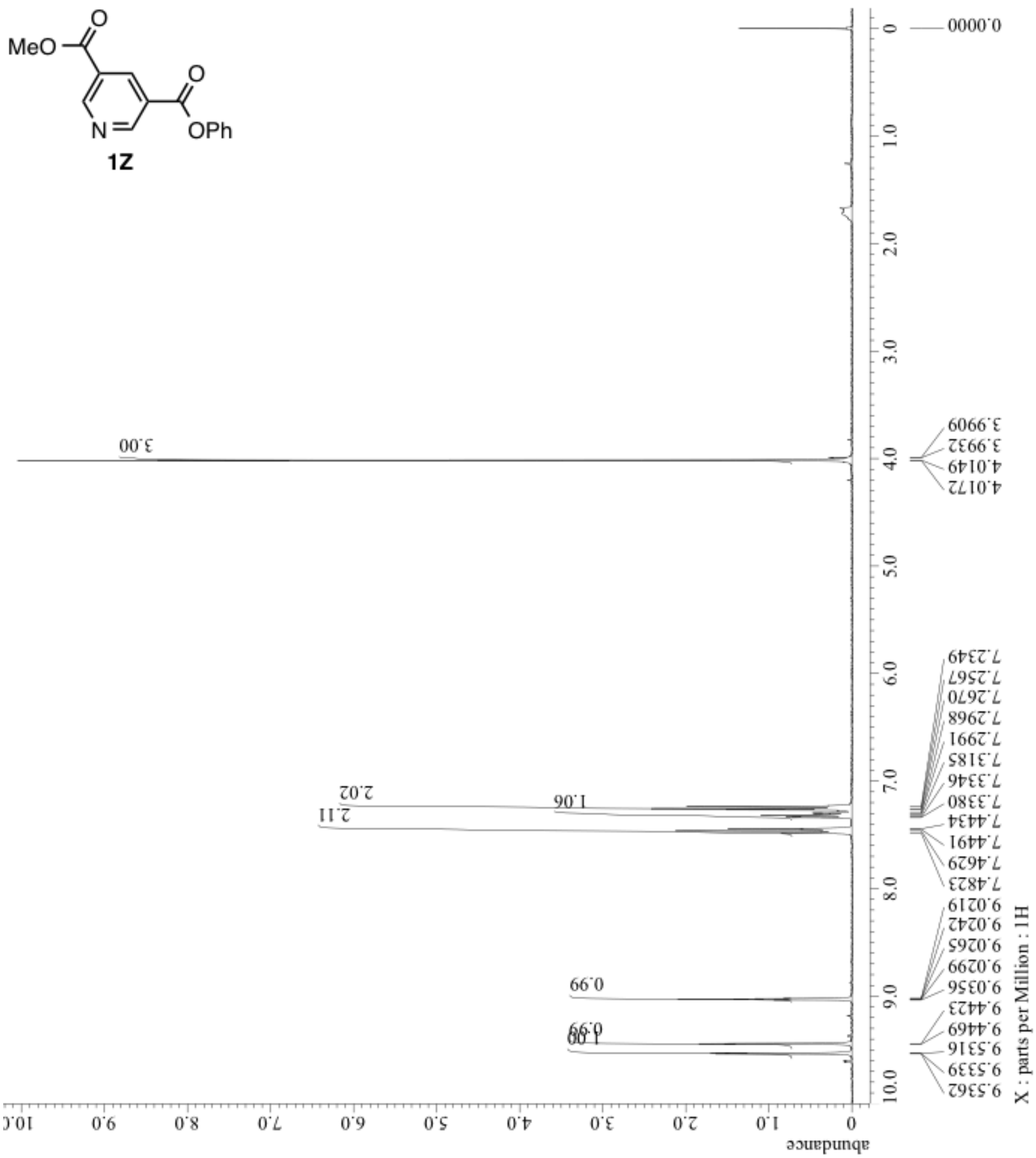


**Supplementary Figure 19. $^{1}$H NMR (400 MHz, $CDCl_3$) of 1X**

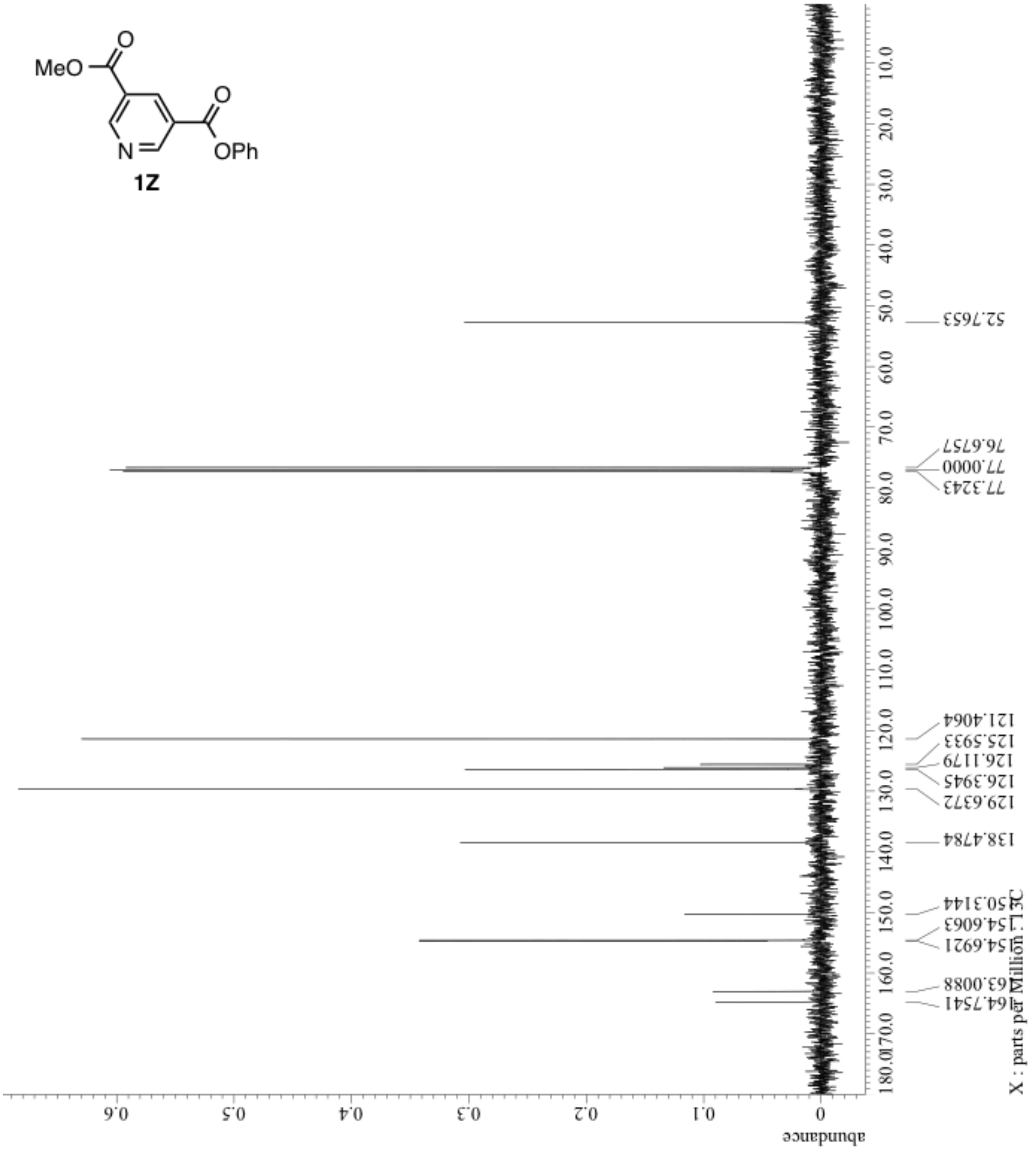


**Supplementary Figure 20. $^{13}C$ NMR (100 MHz, $CDCl_3$) of 1Z**

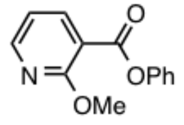


**1AA**

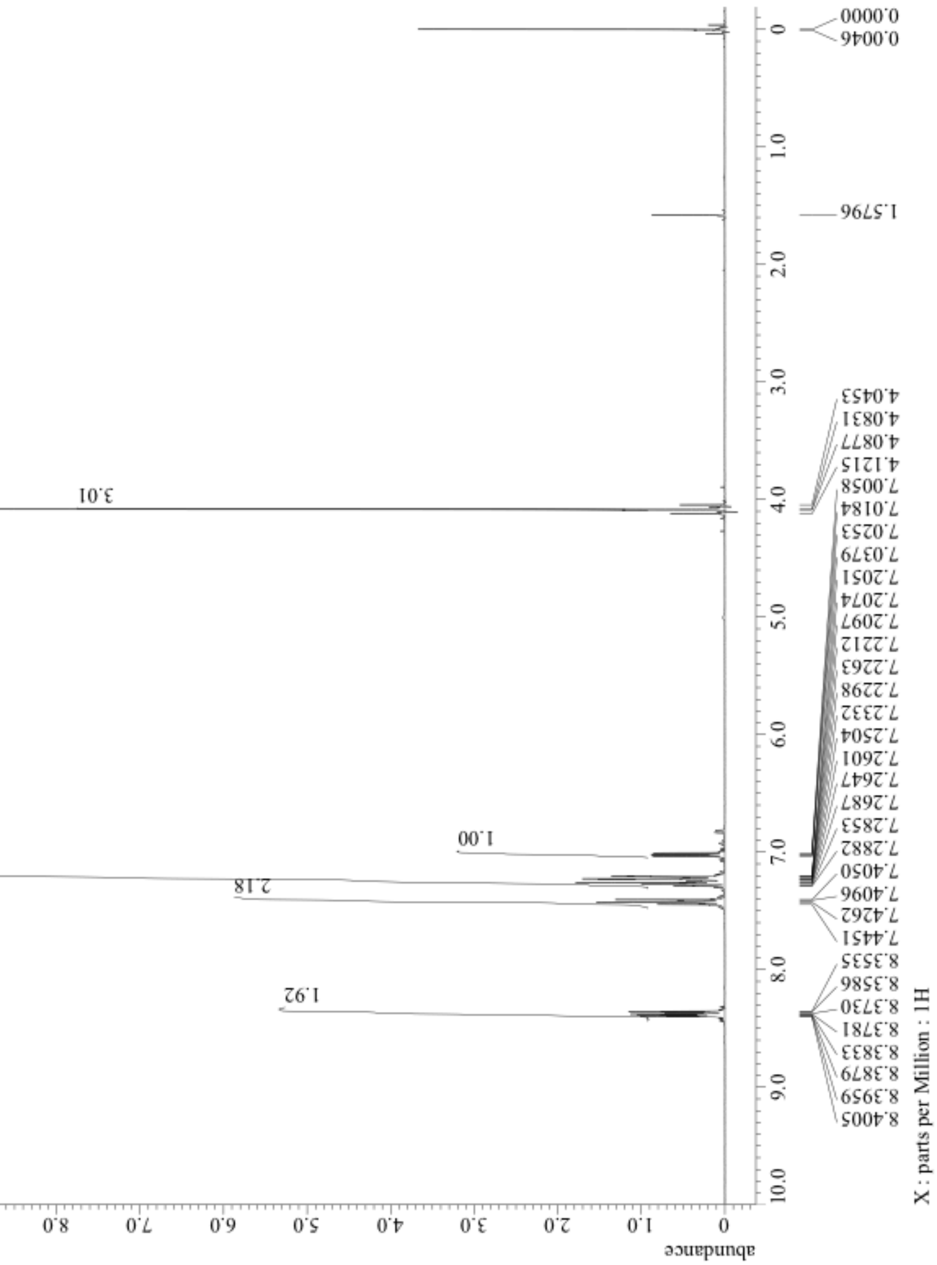


**Supplementary Figure 21.** $^1$**H NMR (400 MHz, CDCl$_3$) of 1AA**

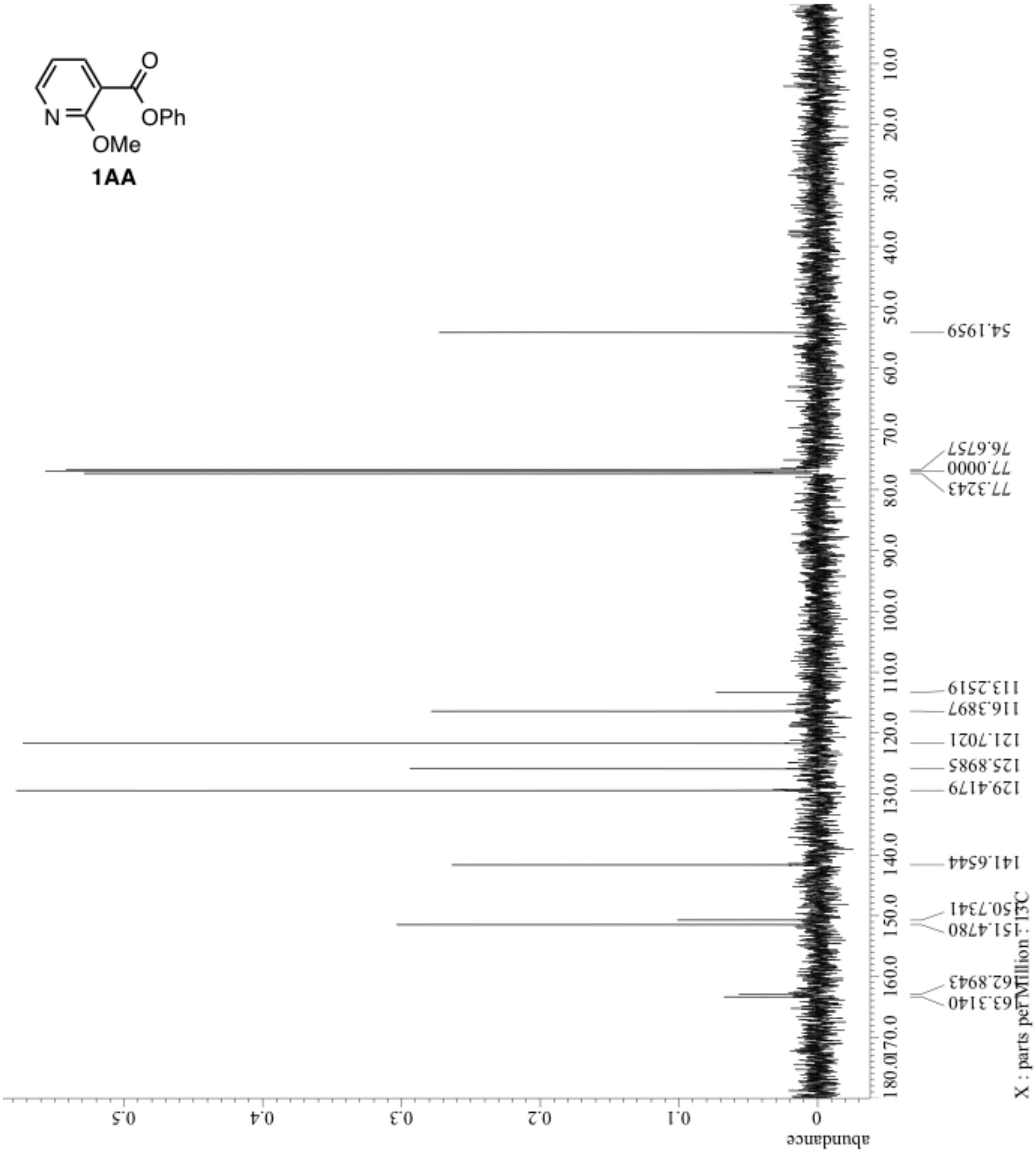


**Supplementary Figure 22. $^{13}$C NMR (100 MHz, $CDCl_3$) of 1AA**

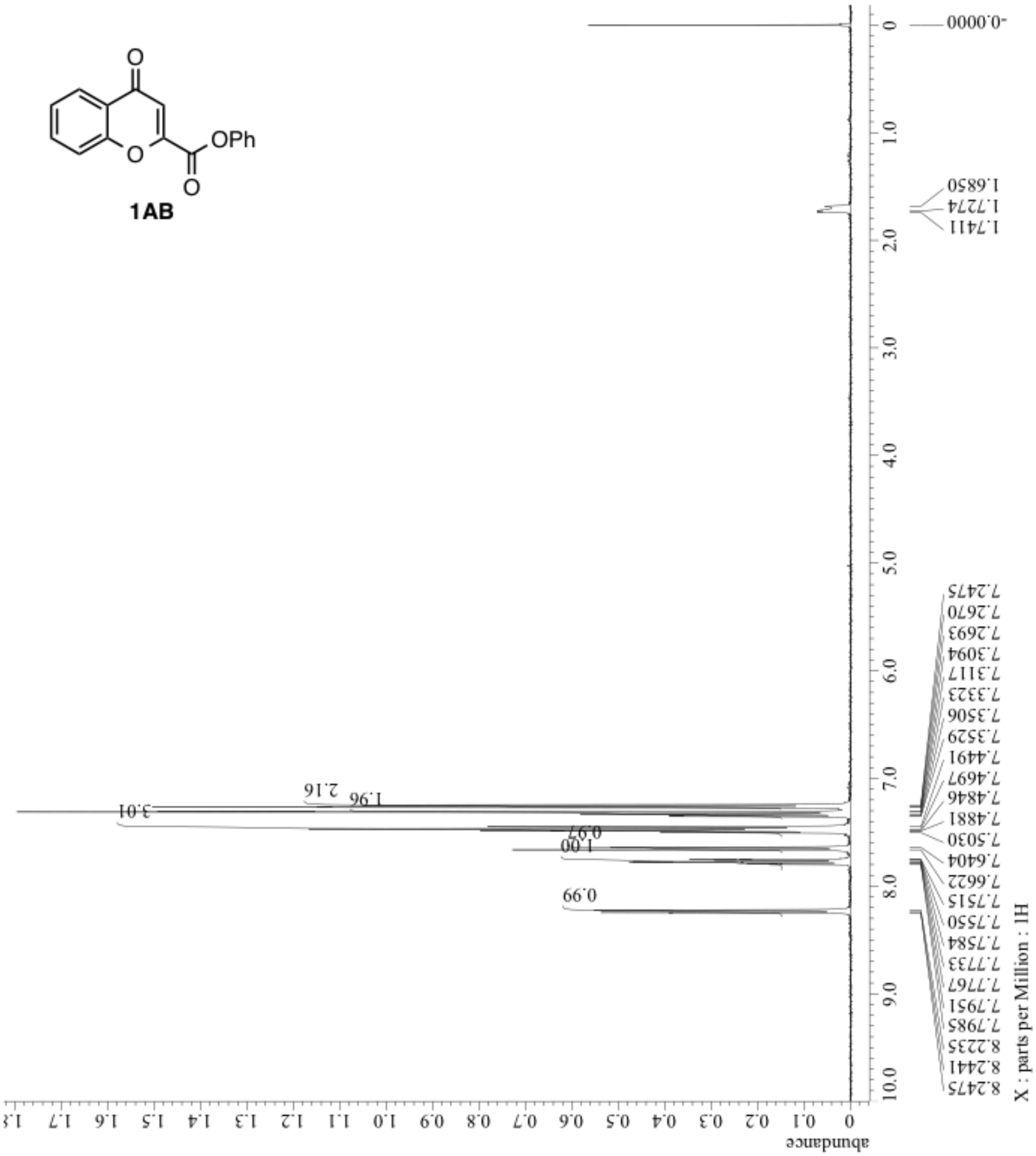


**Supplementary Figure 23. $^{1}$H NMR (400 MHz, $CDCl_3$) of 1AB**

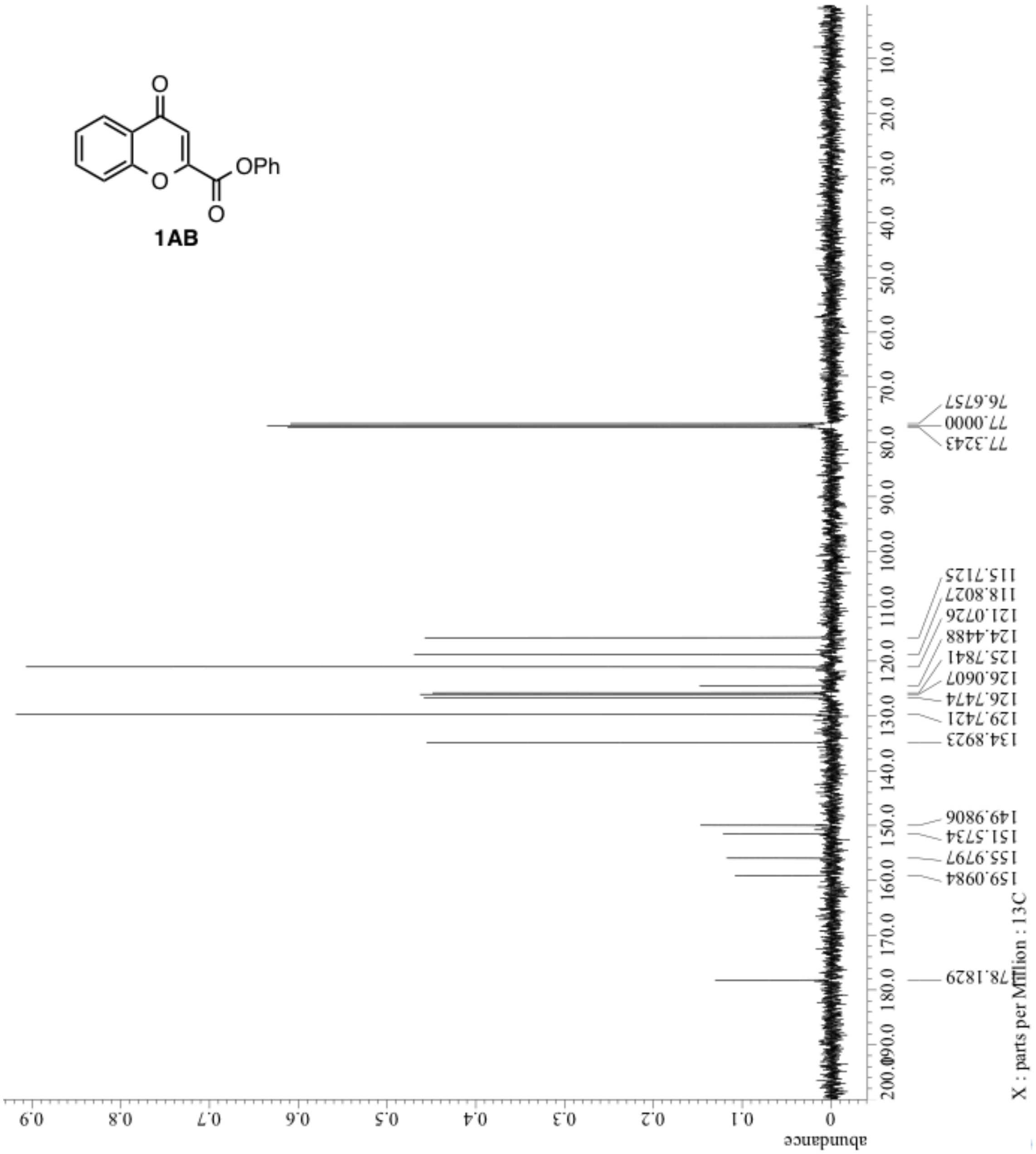



**Supplementary Figure 24. $^{13}$C NMR (100 MHz, CDCl$_3$) of 1AB**

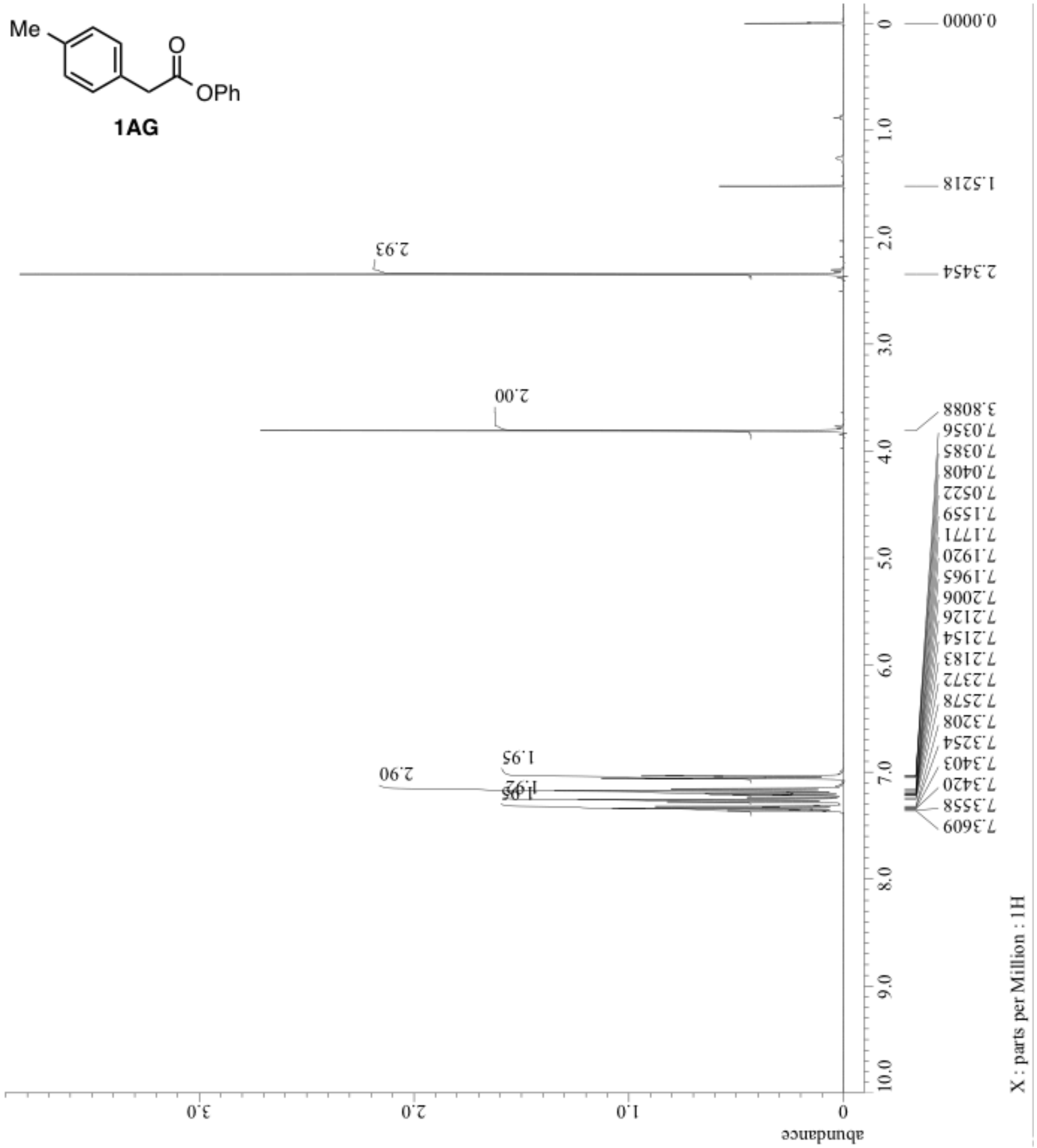


**Supplementary Figure 25. $^{1}$H NMR (400 MHz, $CDCl_3$) of 1AG**

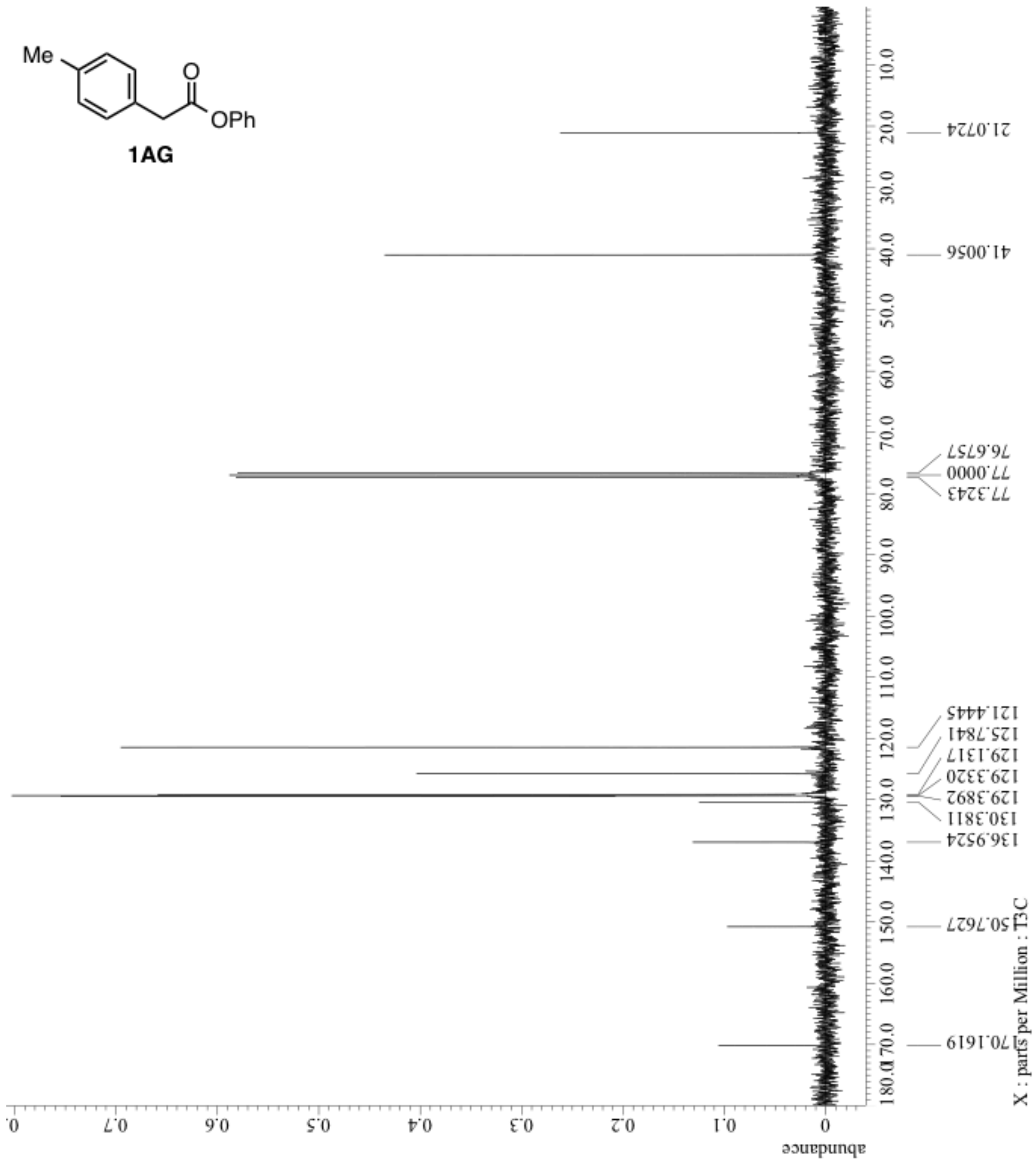

Supplementary Figure 26. $^{13}$C NMR (100 MHz, $CDCl_3$) of 1AG

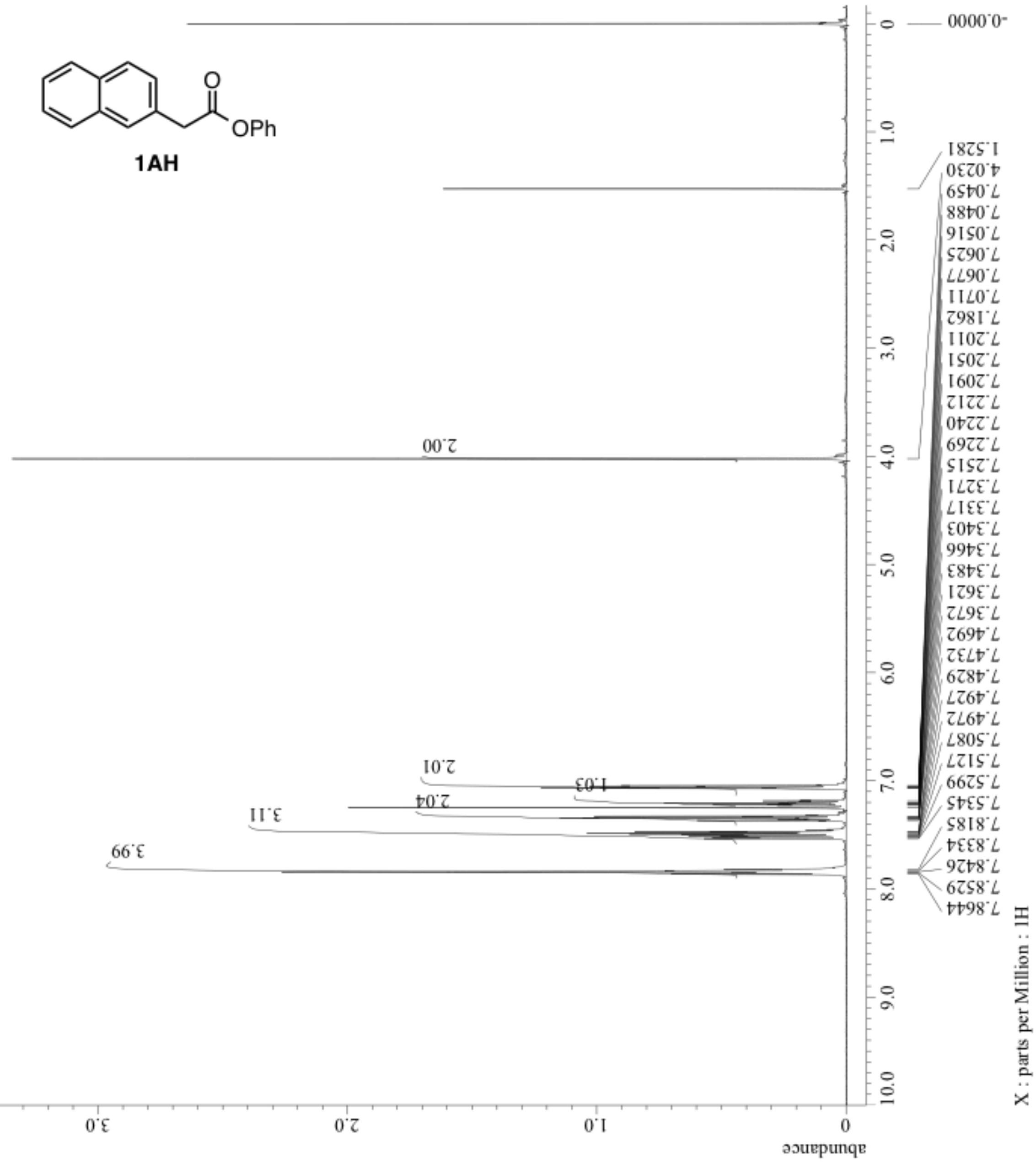


**Supplementary Figure 27. $^{1}$H NMR (400 MHz, $CDCl_3$) of 1AH**

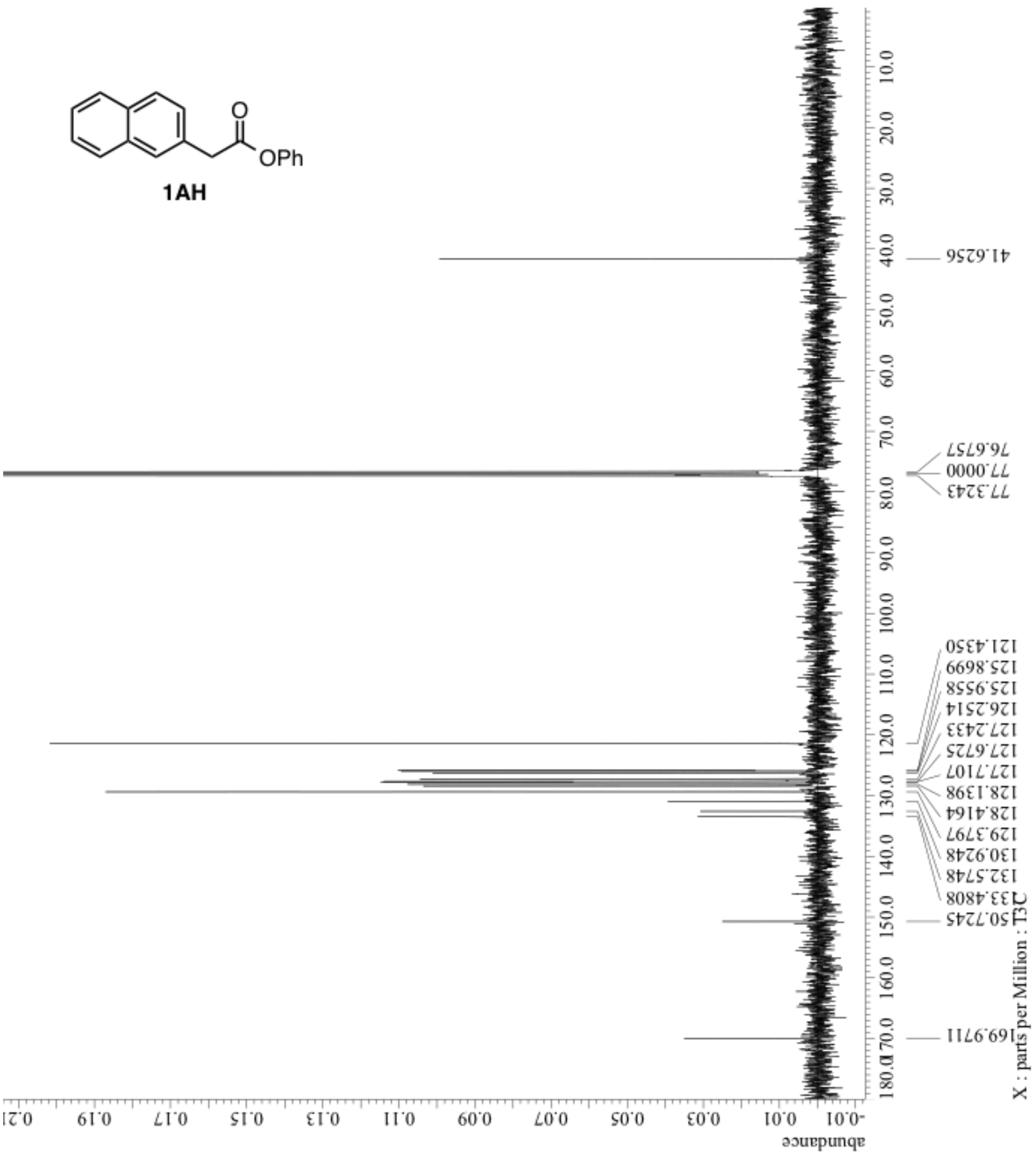


**Supplementary Figure 28. $^{13}C$ NMR (100 MHz, $CDCl_3$) of 1AH**

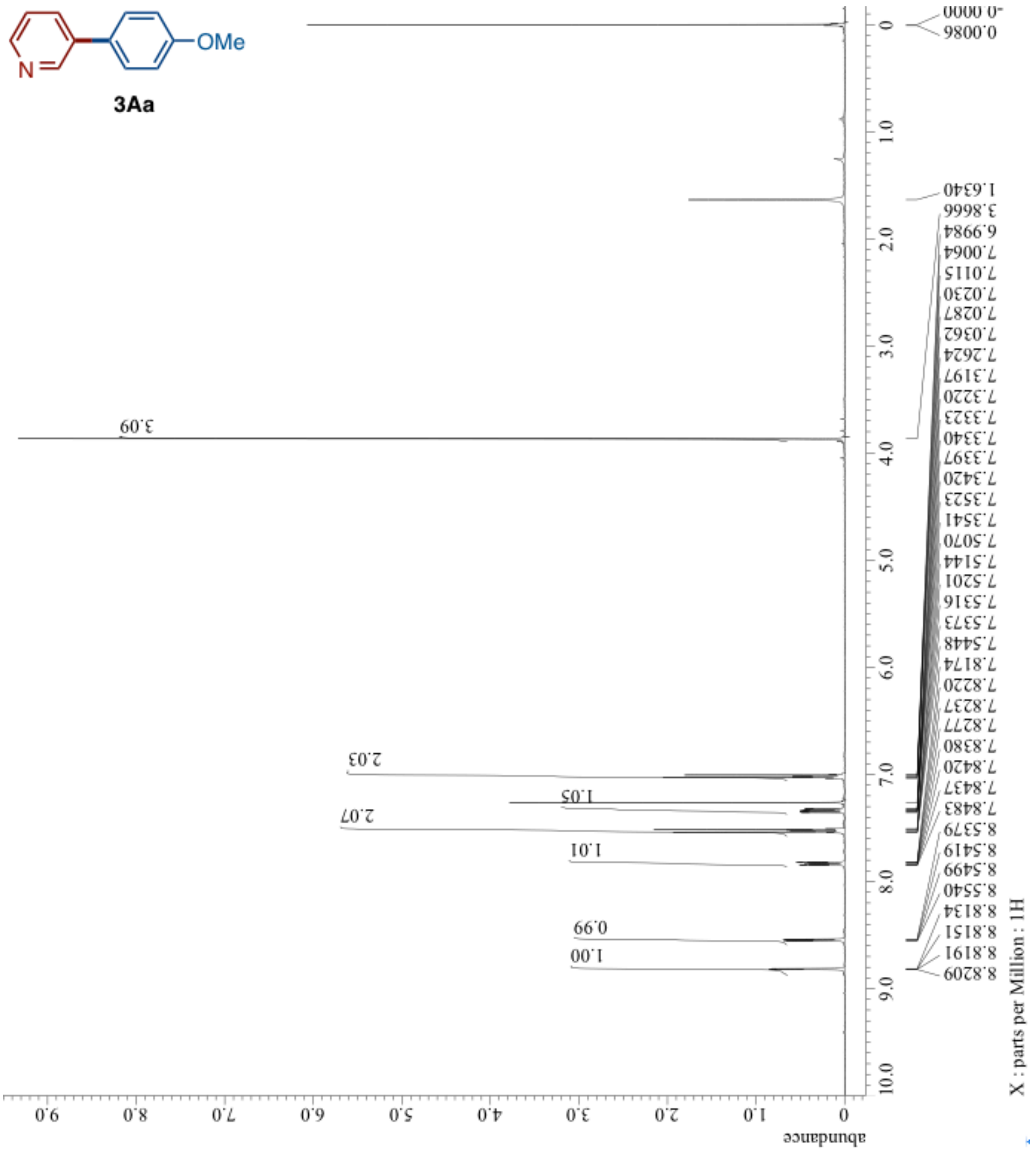


**Supplementary Figure 29.** $^1$H NMR (400 MHz, $CDCl_3$) of 3Aa

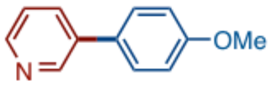


**3Aa**

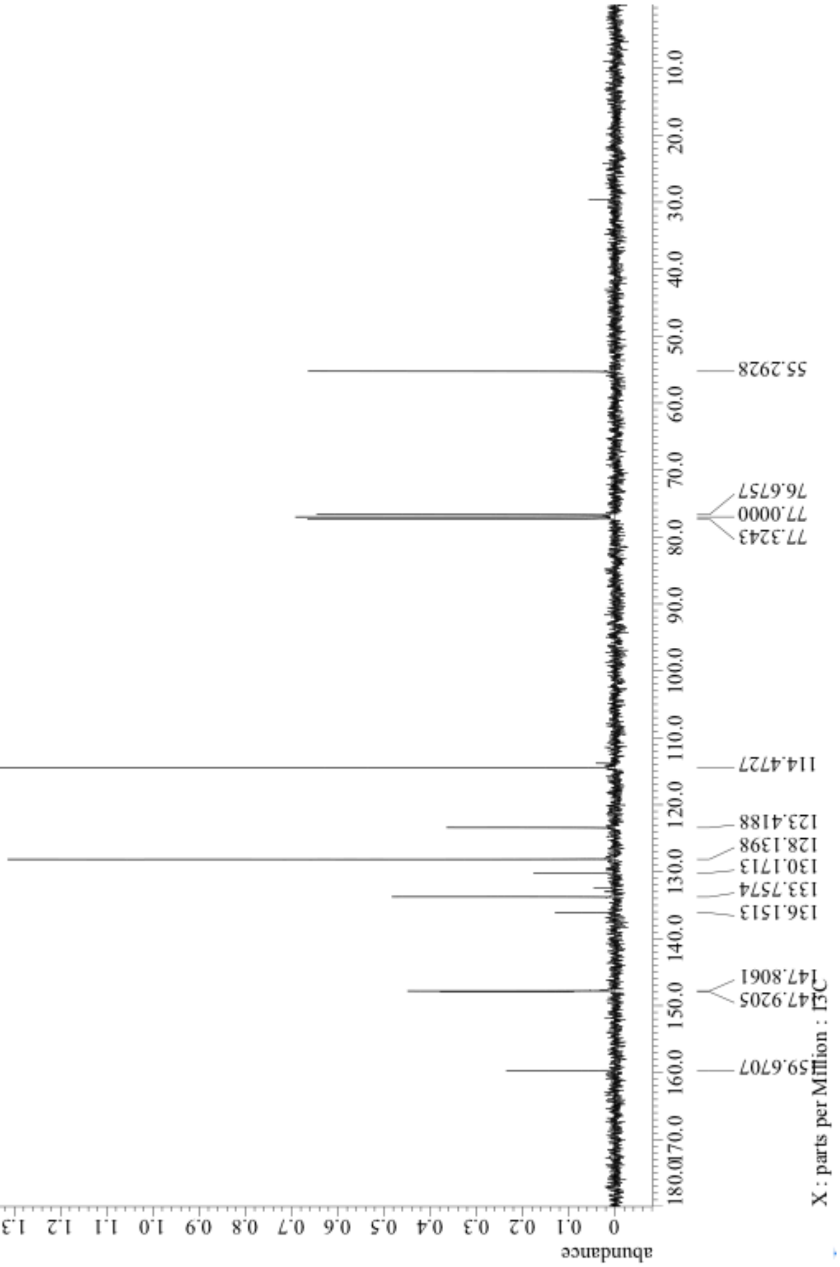


**Supplementary Figure 30.** $^{13}$**C NMR (100 MHz,** $CDCl_3$**) of 3Aa**

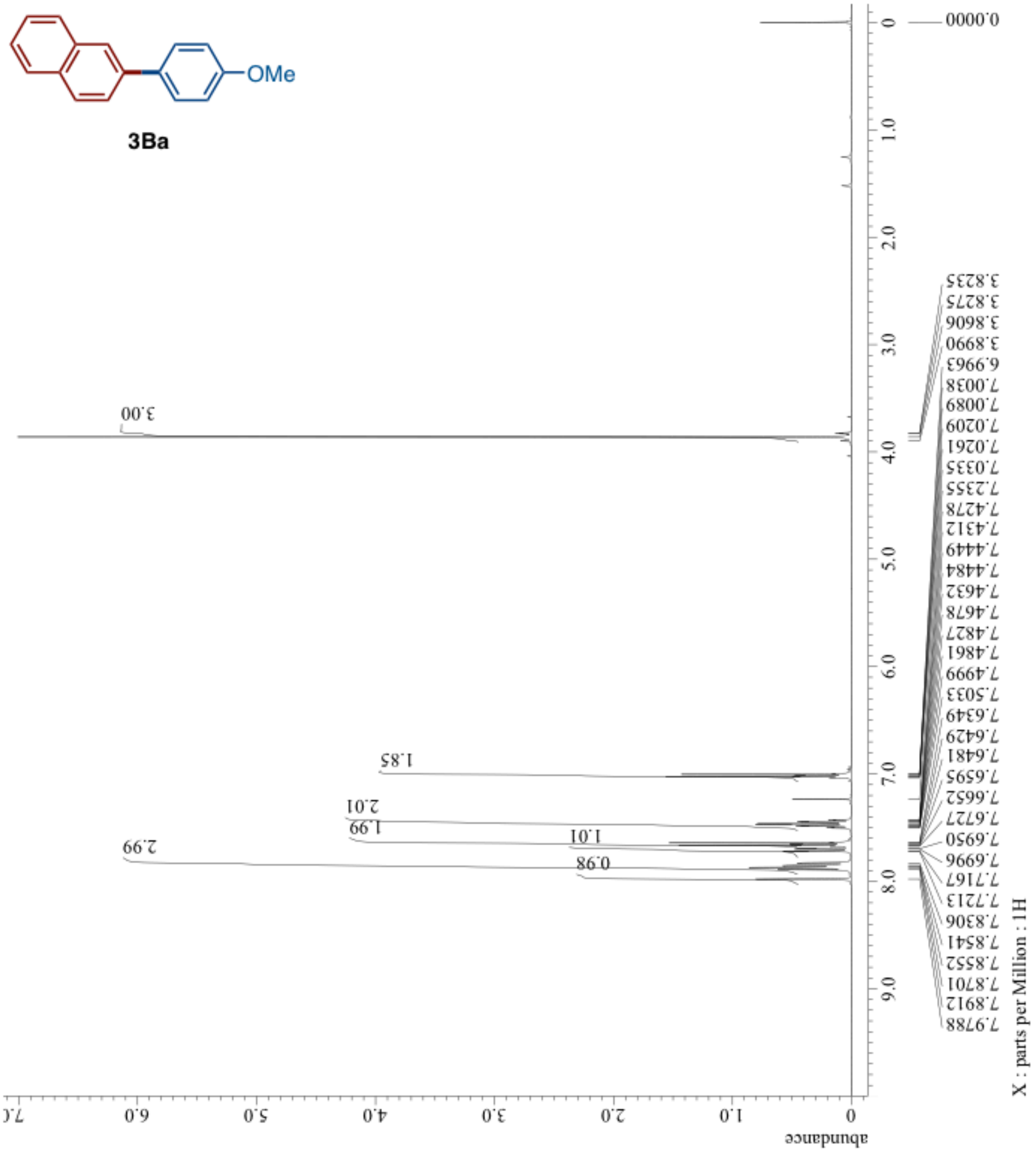


**Supplementary Figure 31.** $^{1}H$ **NMR (400 MHz,** $CDCl_3$**) of 3Ba**

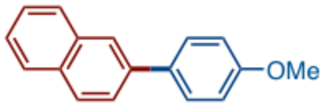


**3Ba**

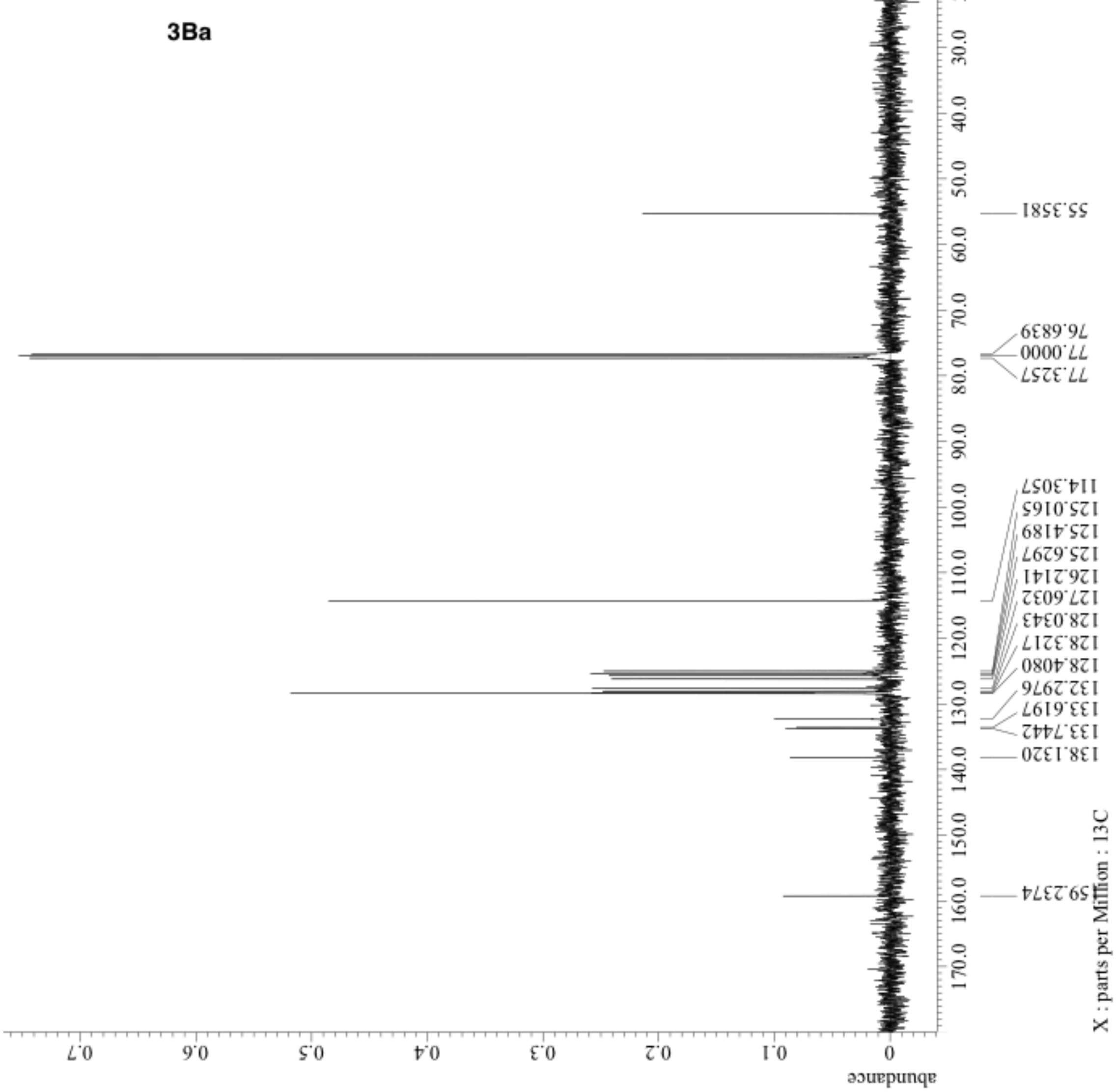


**Supplementary Figure 32. $^{13}$C NMR (100 MHz, $CDCl_3$) of 3Ba**

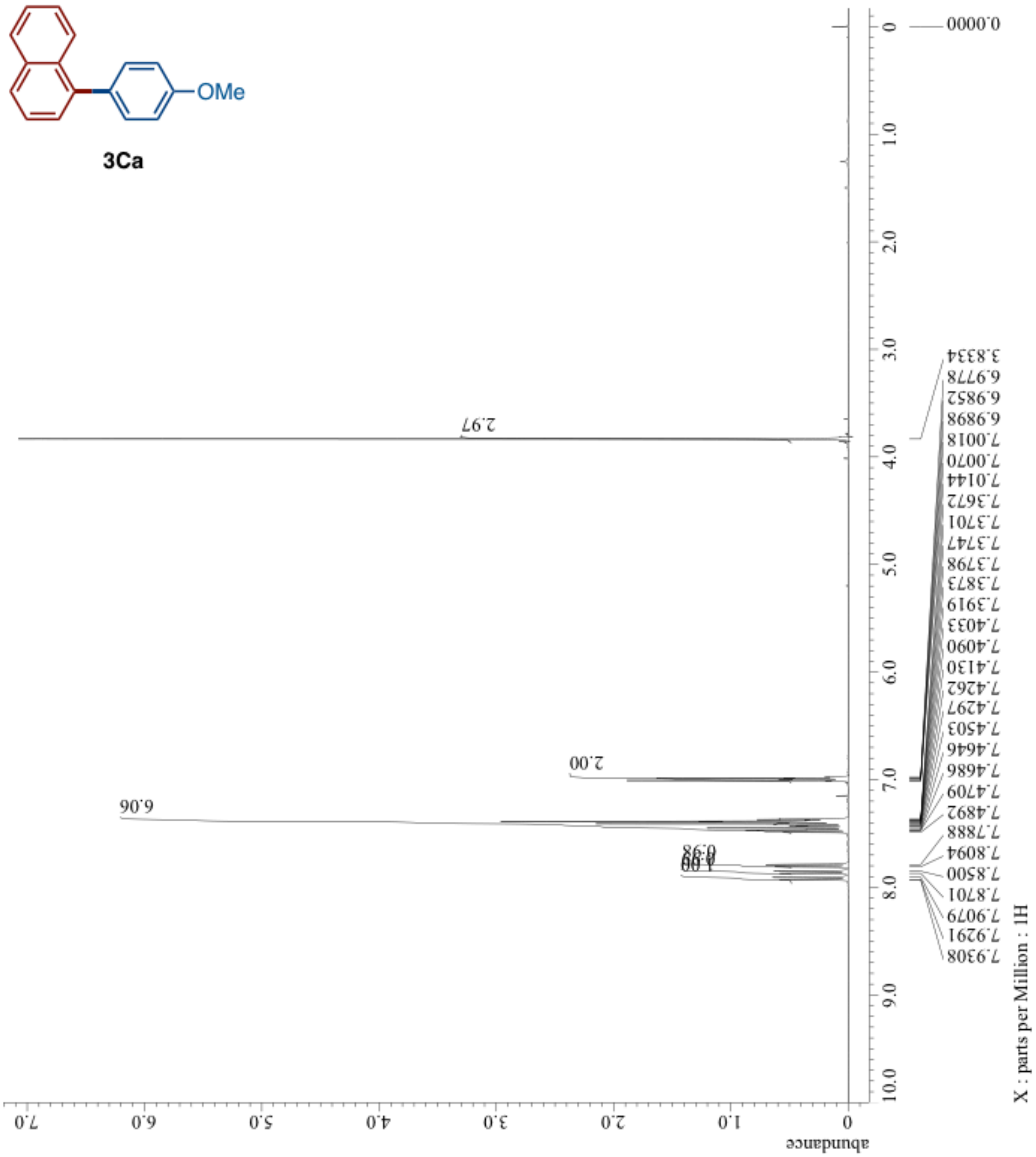


**Supplementary Figure 33.** $^{1}$**H NMR (400 MHz, CDCl**$_3$**) of 3Ca**

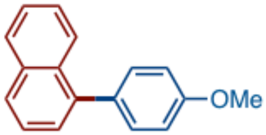


3Ca

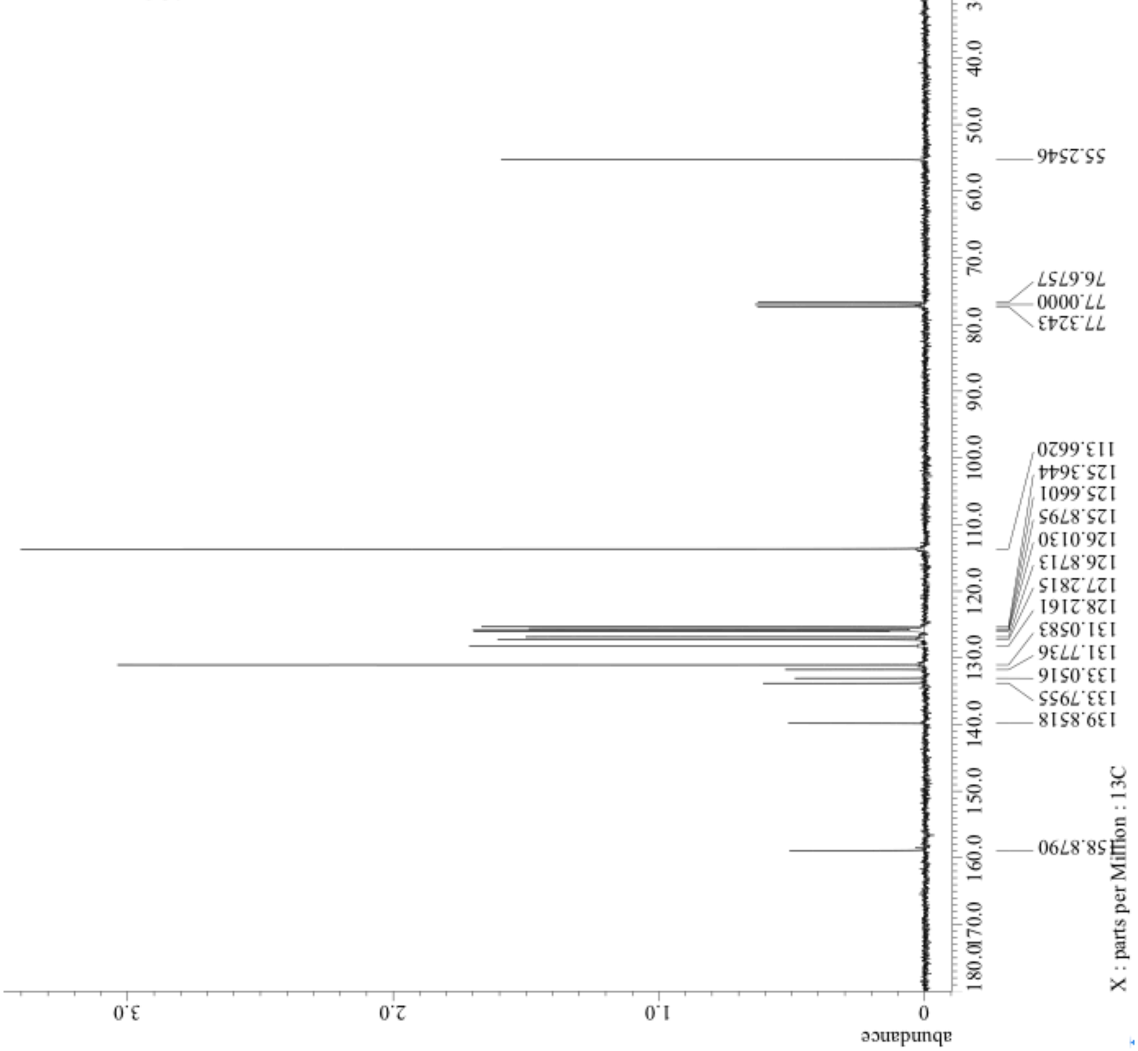


**Supplementary Figure 34. $^{13}$C NMR (100 MHz, $CDCl_3$) of 3Ca**

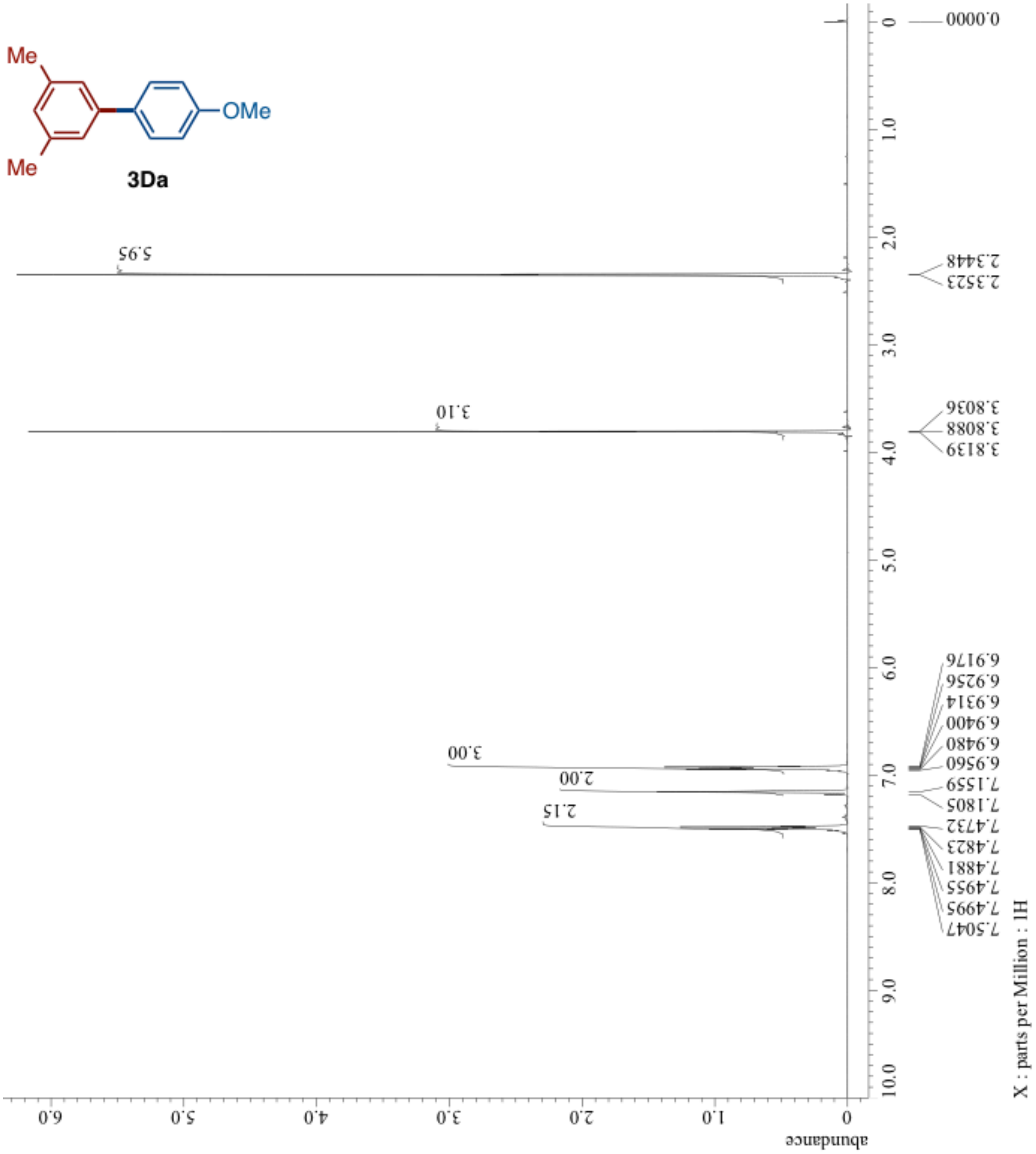


**Supplementary Figure 35.** $^{1}$**H NMR (400 MHz, CDCl$_3$) of 3Da**

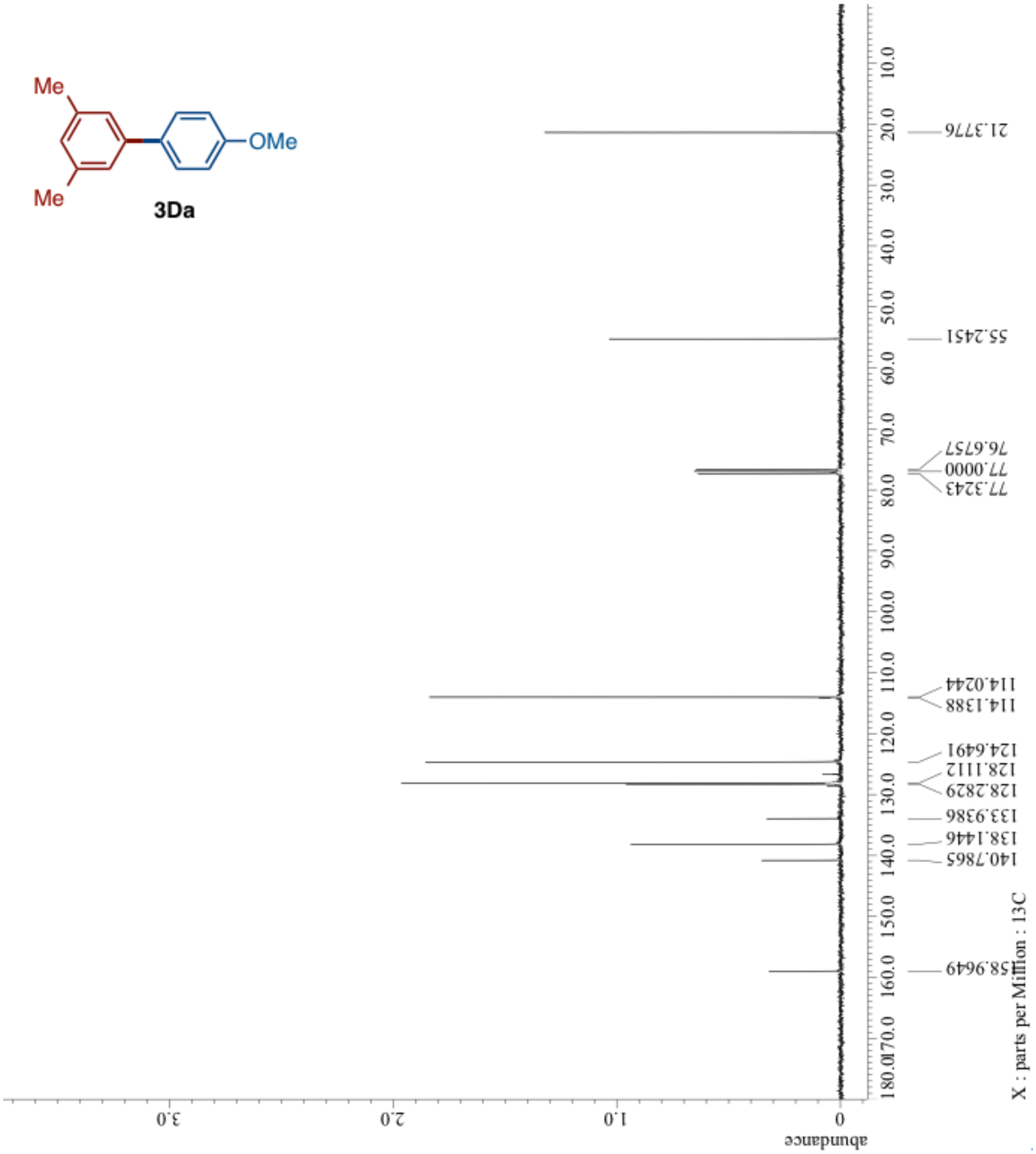


**Supplementary Figure 36. $^{13}C$ NMR (100 MHz, $CDCl_3$) of 3Da**

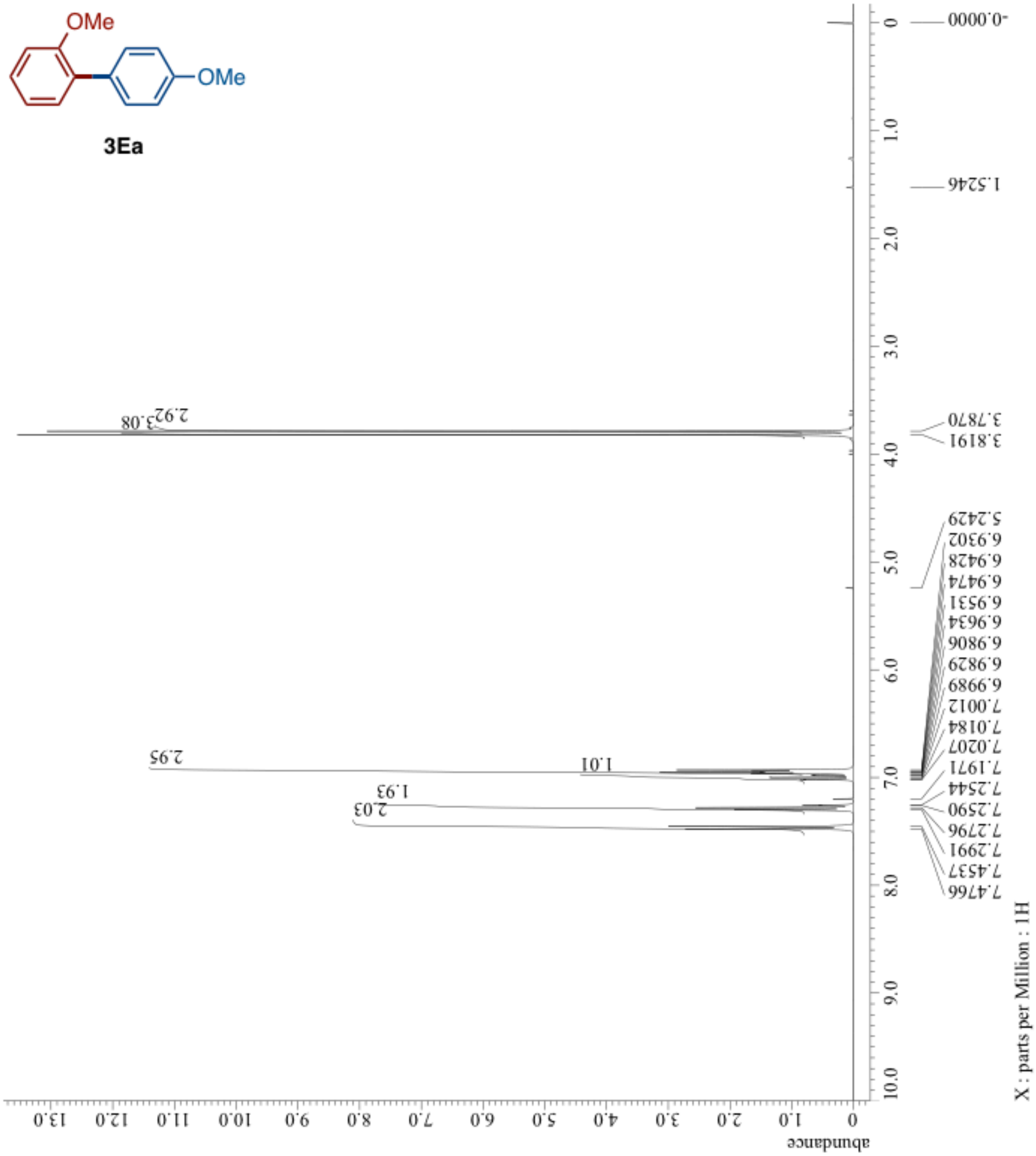


**Supplementary Figure 37.** $^{1}$**H NMR (400 MHz, CDCl$_3$) of 3Ea**

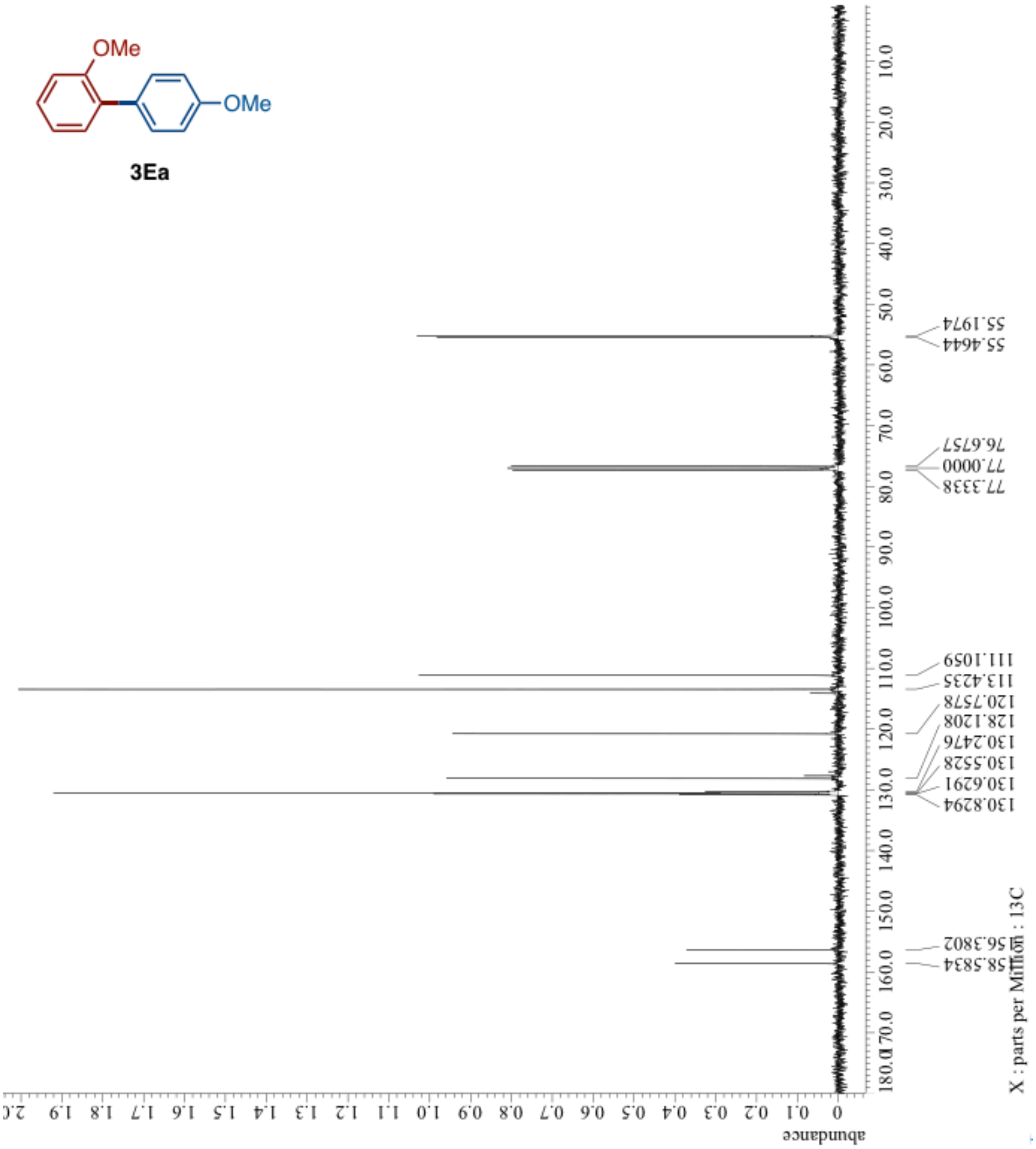


**Supplementary Figure 38. $^{13}$C NMR (100 MHz, $CDCl_3$) of 3Ea**

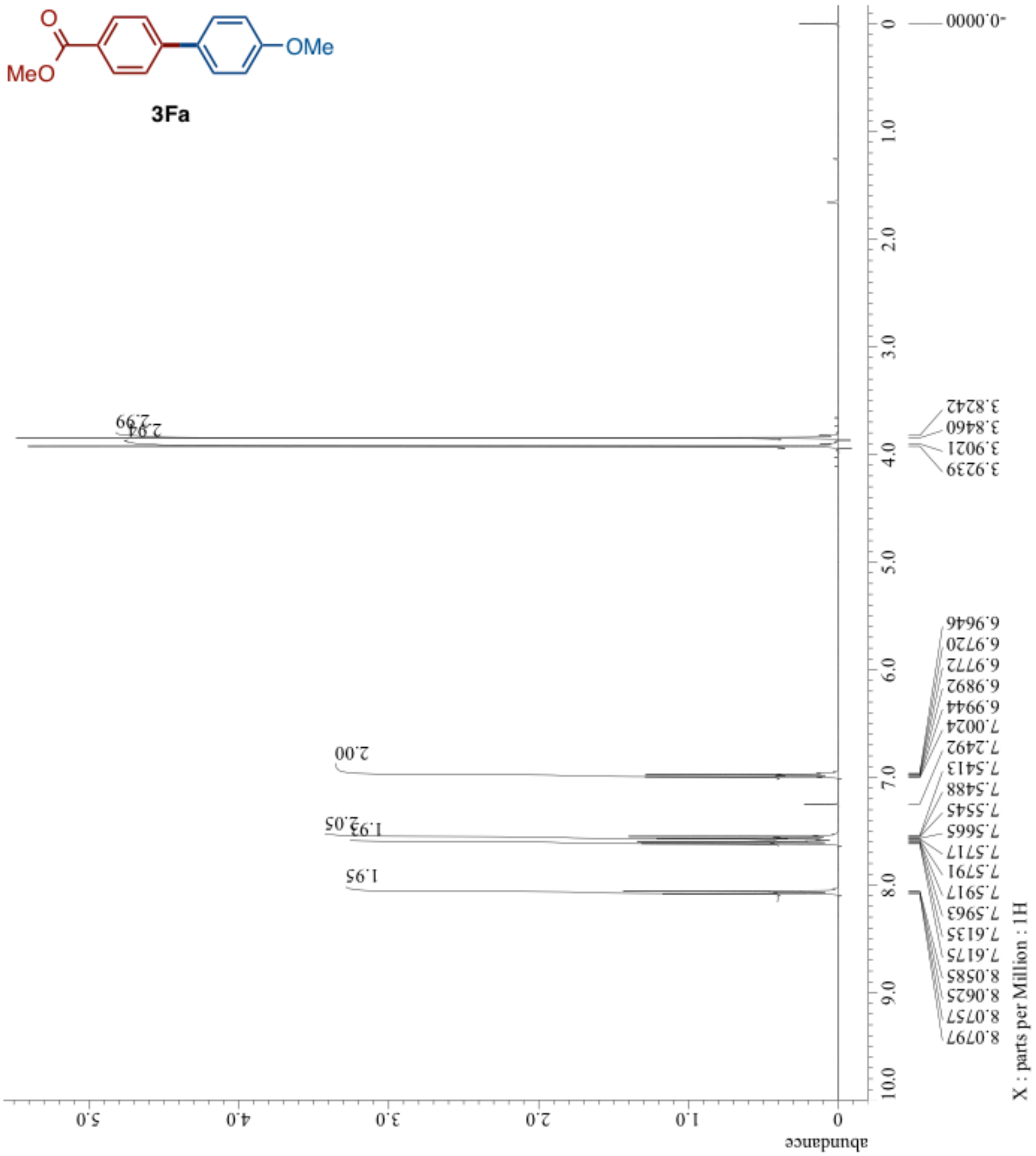


**Supplementary Figure 39. $^{1}$H NMR (400 MHz, $CDCl_3$) of 3Fa**

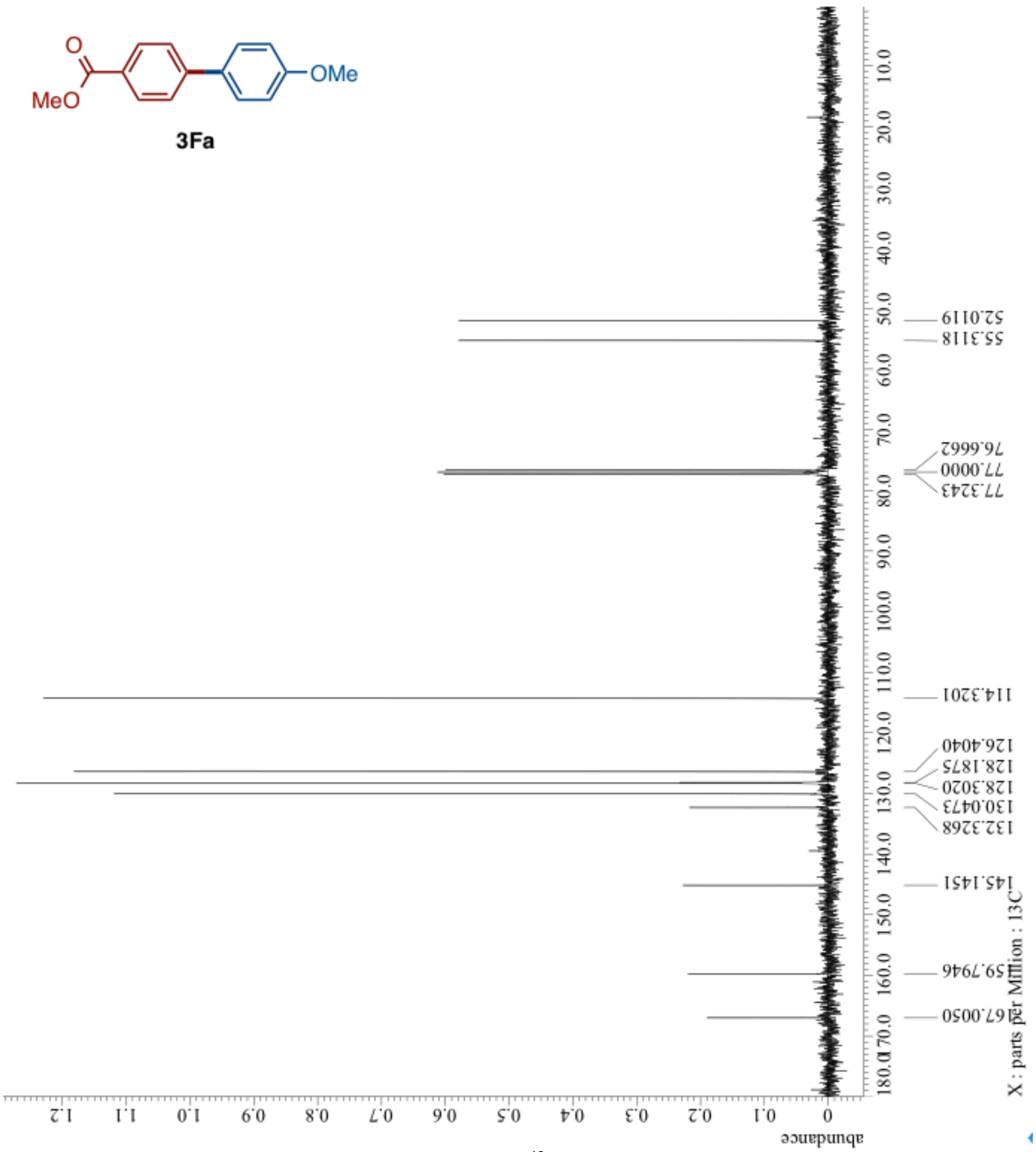


**Supplementary Figure 40.** $^{13}$**C NMR (100 MHz,** $CDCl_3$**) of 3Fa**

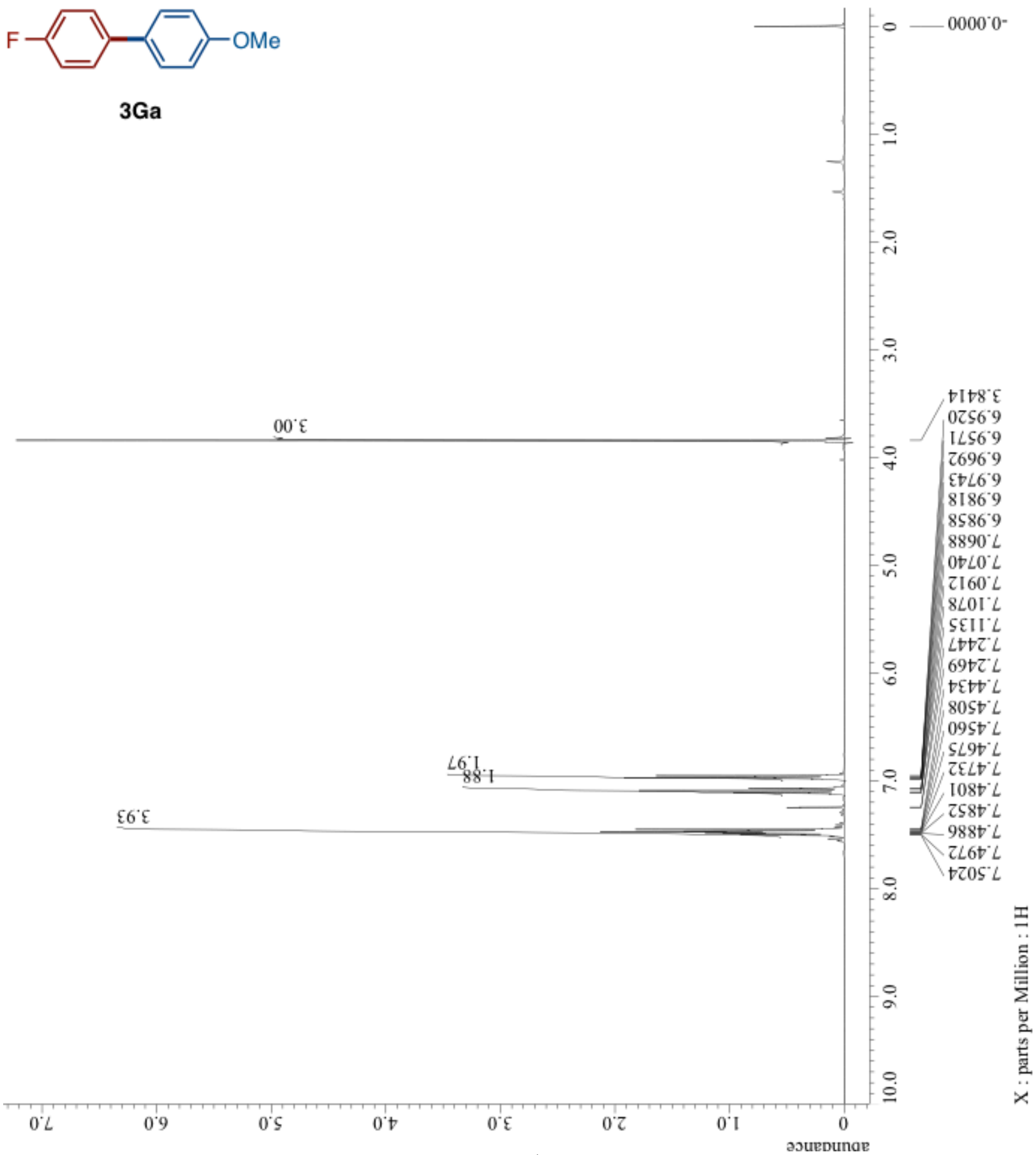


**Supplementary Figure 41.** **$^{1}H$ NMR (400 MHz, $CDCl_3$) of 3Ga**

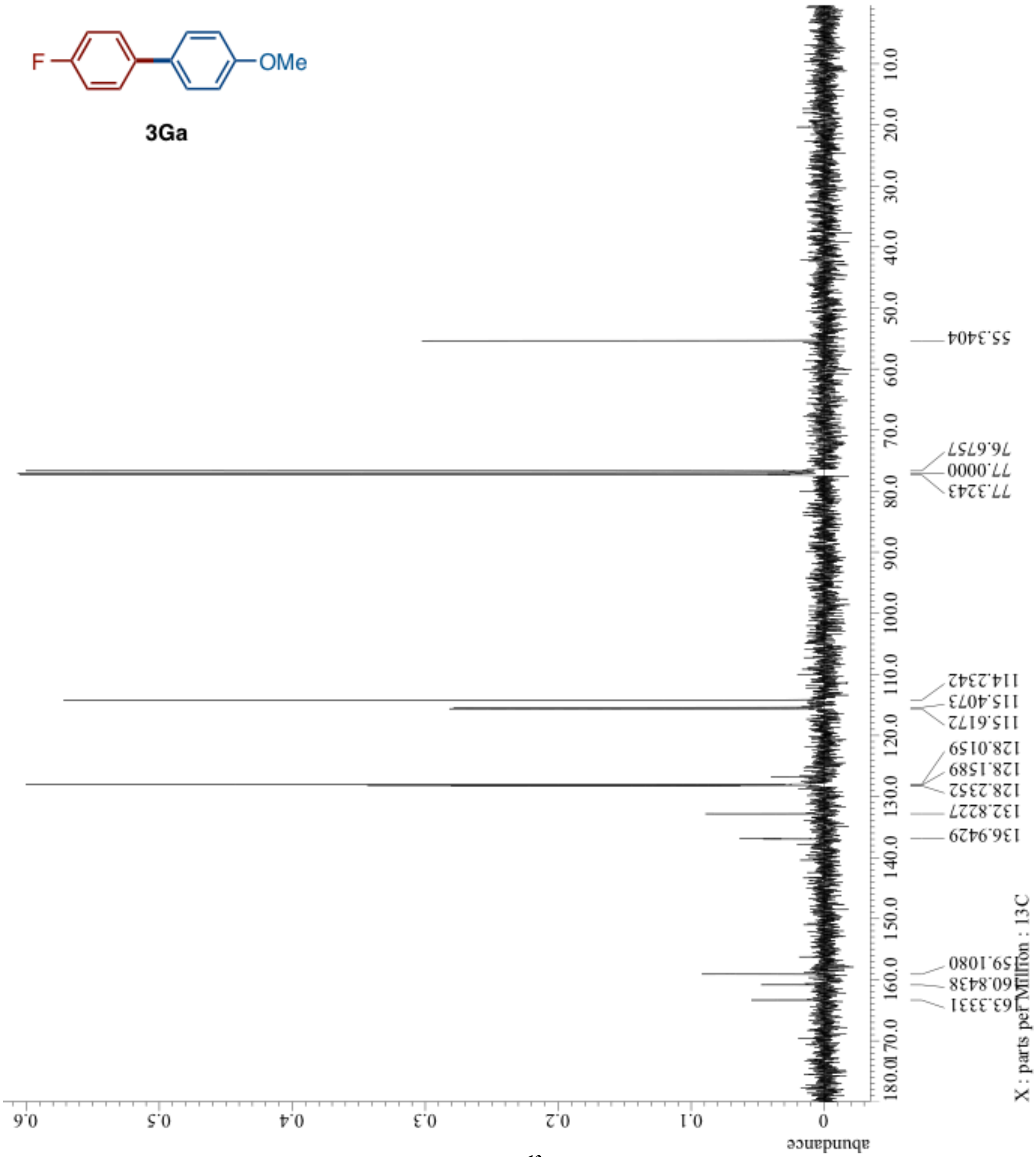


**Supplementary Figure 42. $^{13}C$ NMR (100 MHz, $CDCl_3$) of 3Ga**

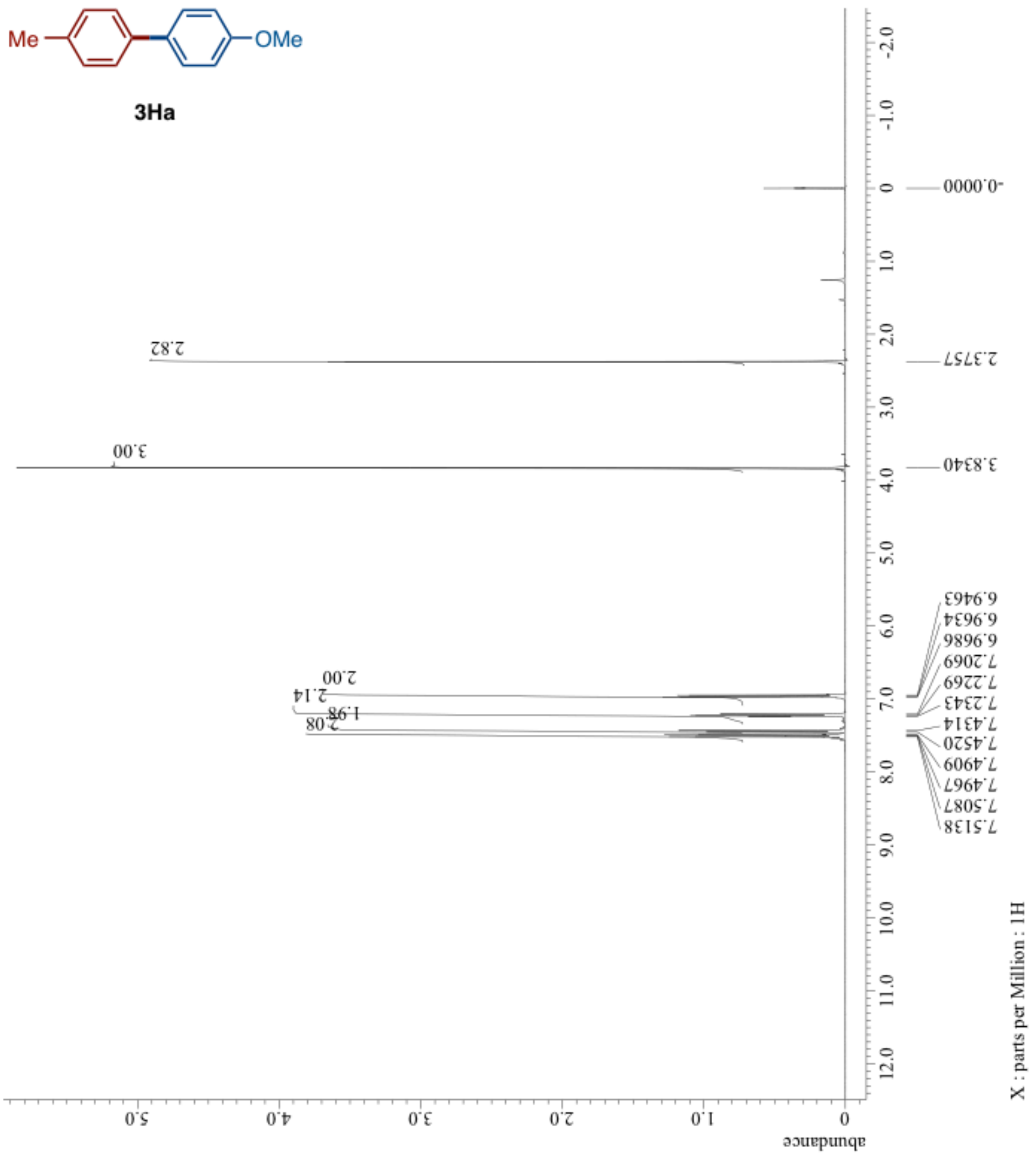


**Supplementary Figure 43. $^1$H NMR (400 MHz, $CDCl_3$) of 3Ha**

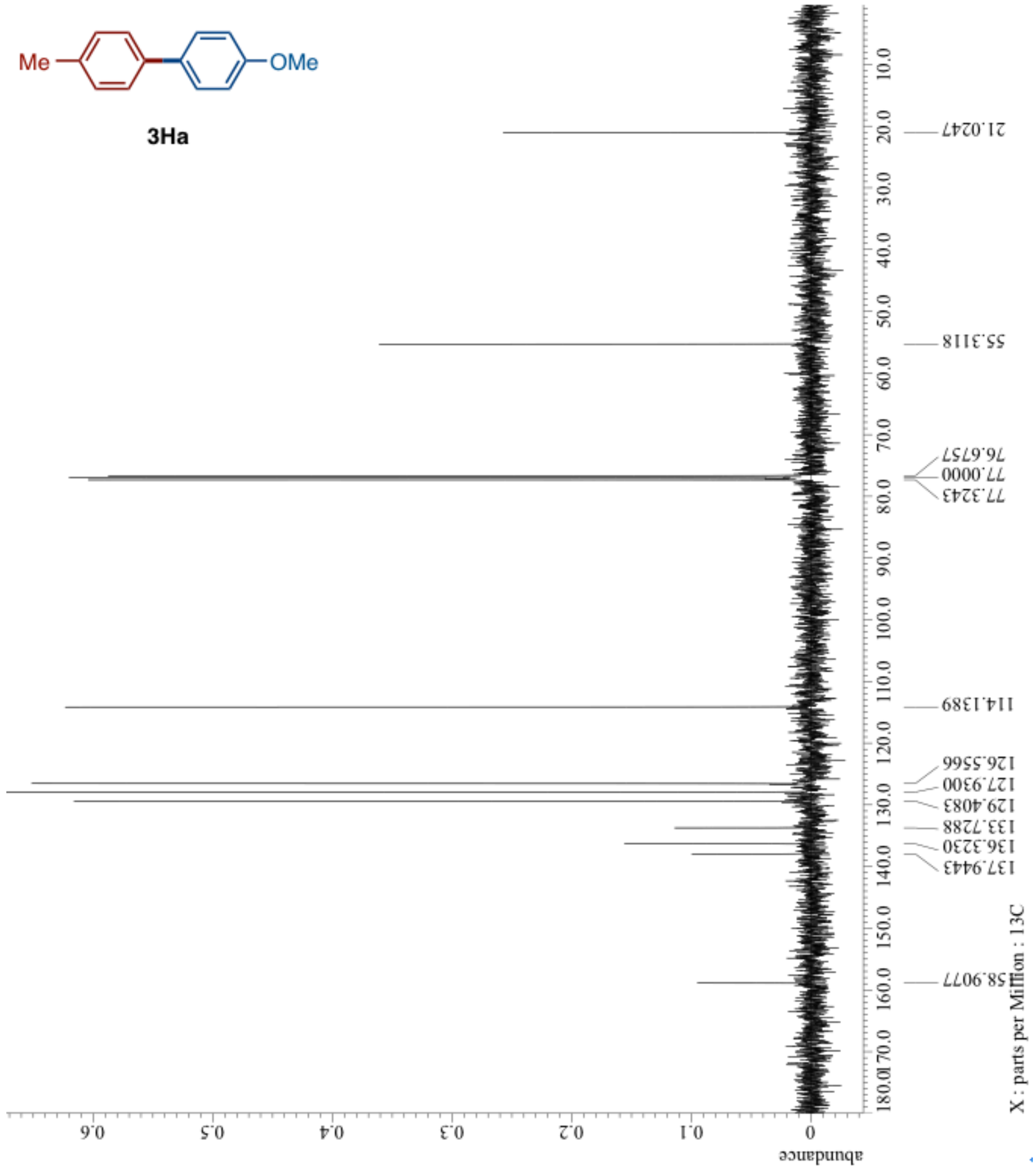


**Supplementary Figure 44.** $^{13}$C NMR (100 MHz, $CDCl_3$) of 3Ha

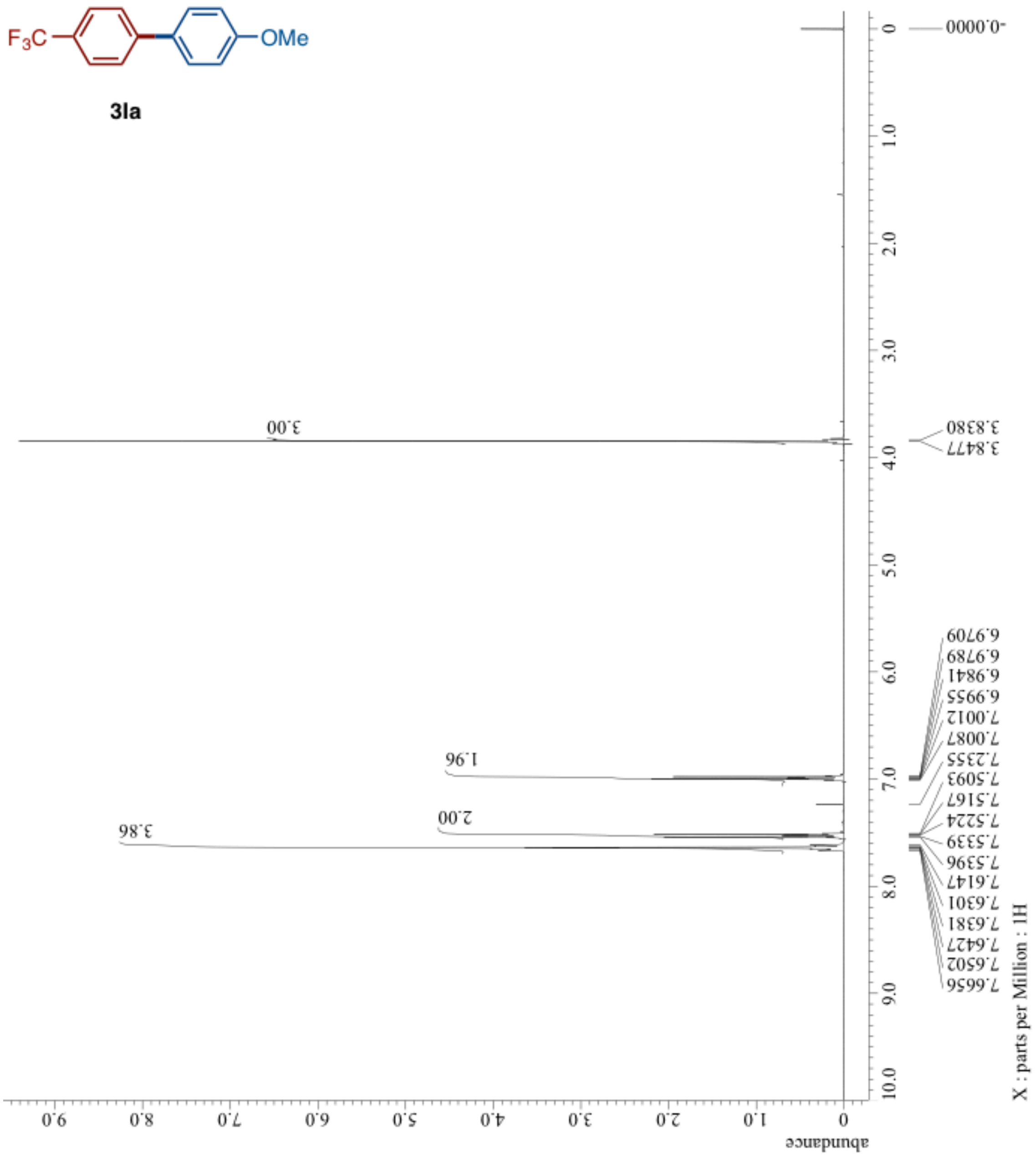

Supplementary Figure 45. $^{1}$H NMR (400 MHz, $CDCl_3$) of 3Ia

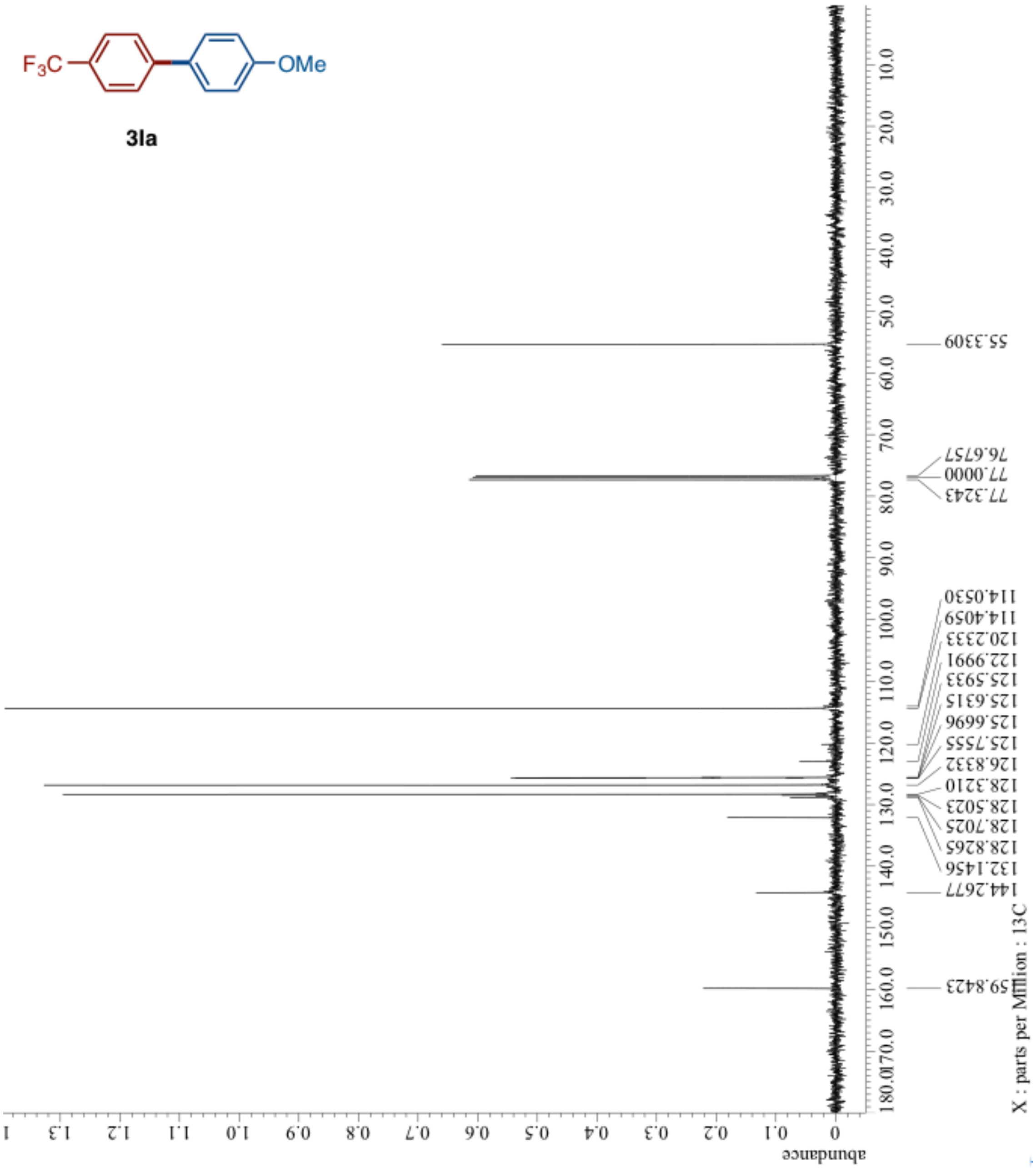


Supplementary Figure 46. $^{13}C$ NMR (100 MHz, $CDCl_3$) of 3Ia

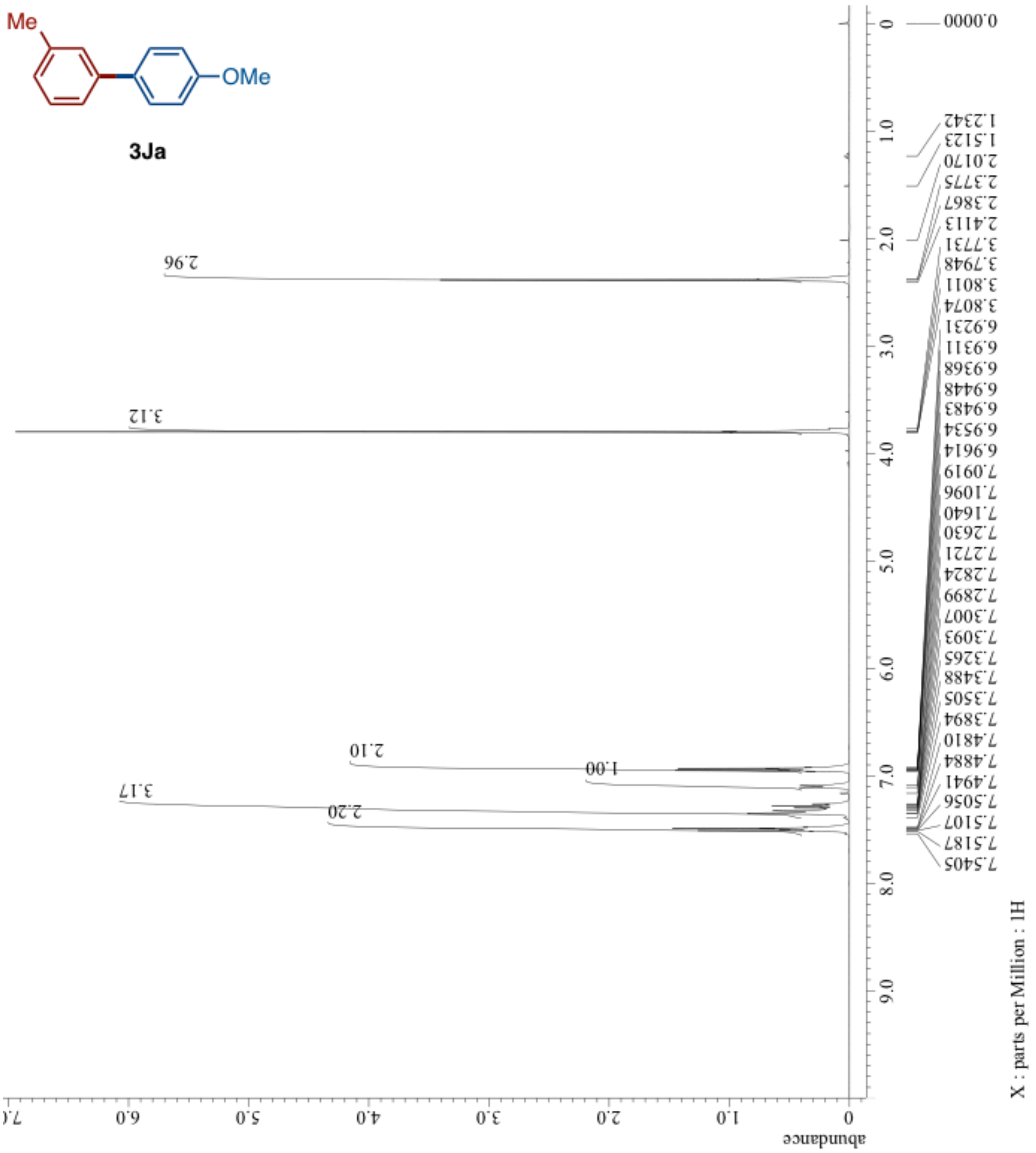


**Supplementary Figure 47.** **$^{1}$H NMR (400 MHz, $CDCl_3$) of 3Ja**

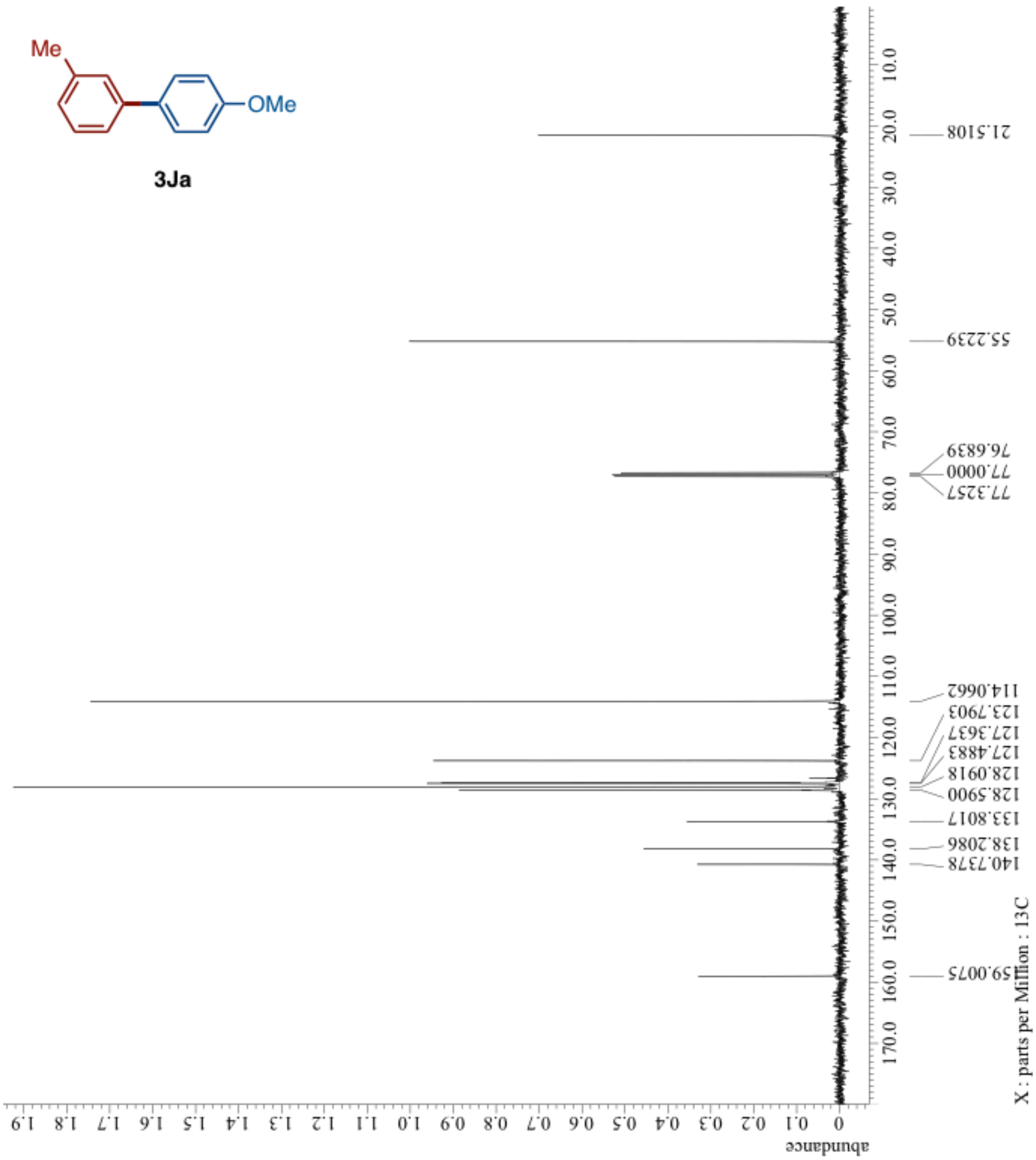


**Supplementary Figure 48.** $^{13}C$ **NMR (100 MHz,** $CDCl_3$**) of 3Ja**

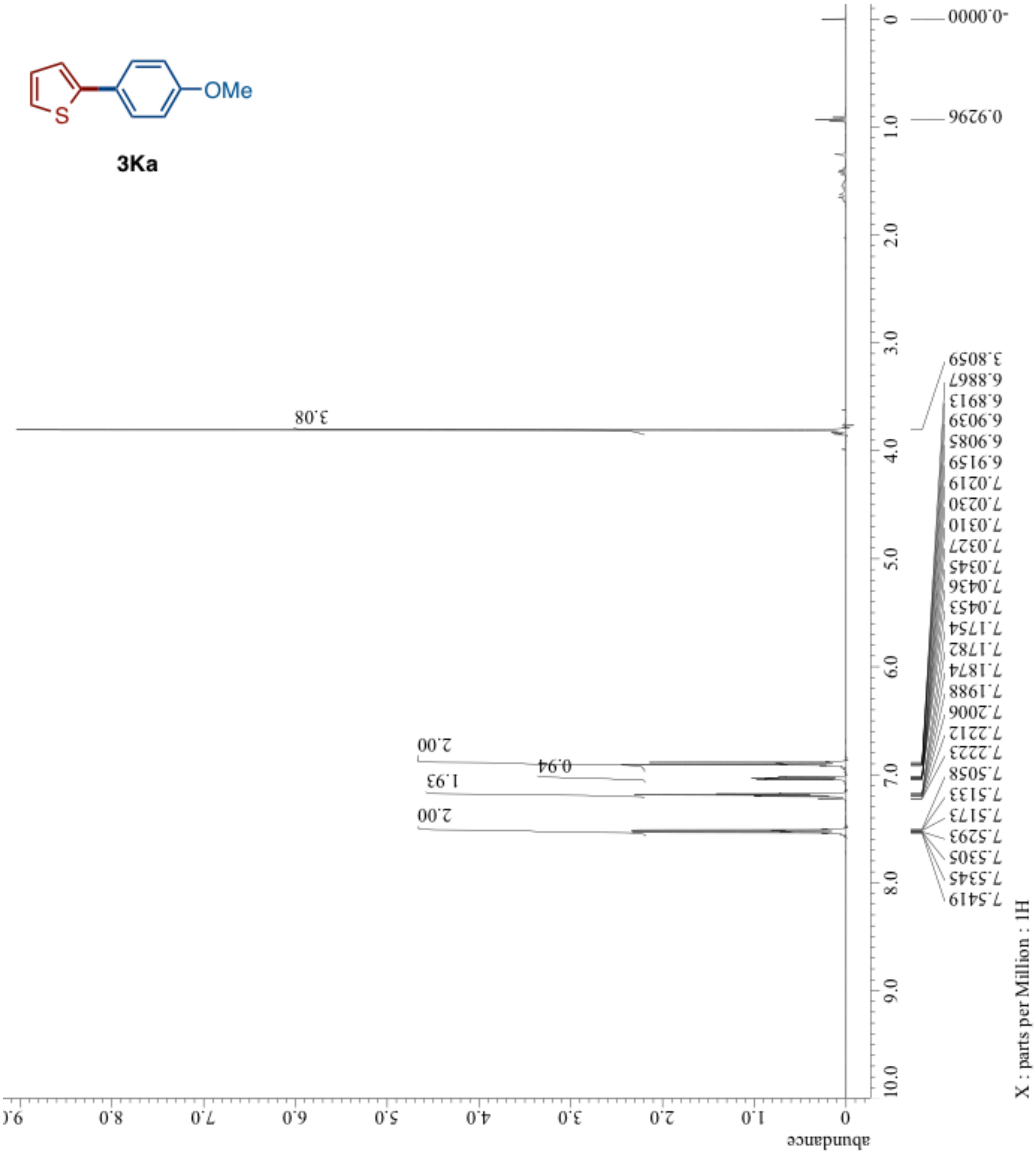


**Supplementary Figure 49.** $^1$**H NMR (400 MHz, CDCl$_3$) of 3Ka**

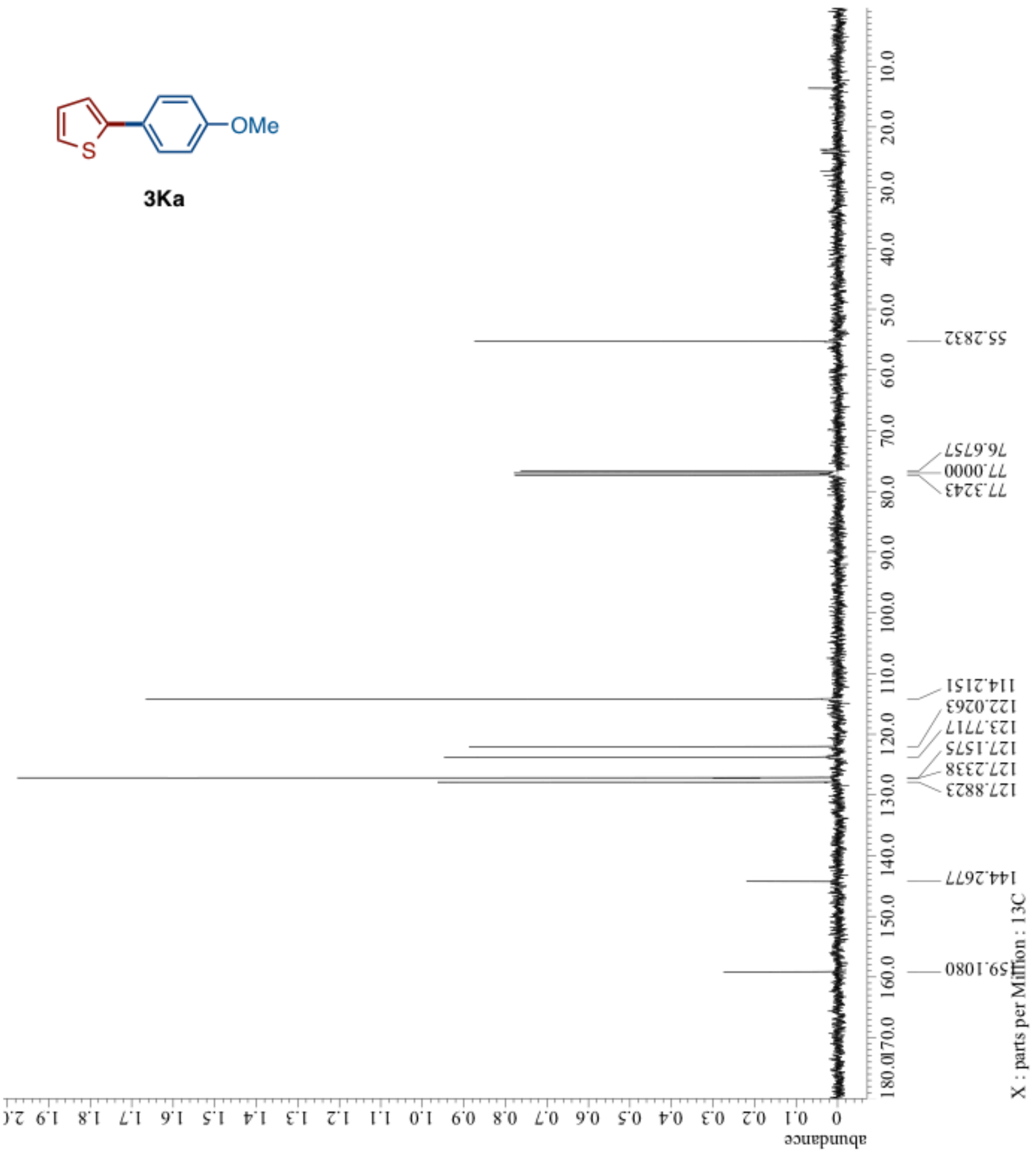


**Supplementary Figure 50. $^{13}$C NMR (100 MHz, $CDCl_3$) of 3Ka**

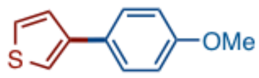


**3La**

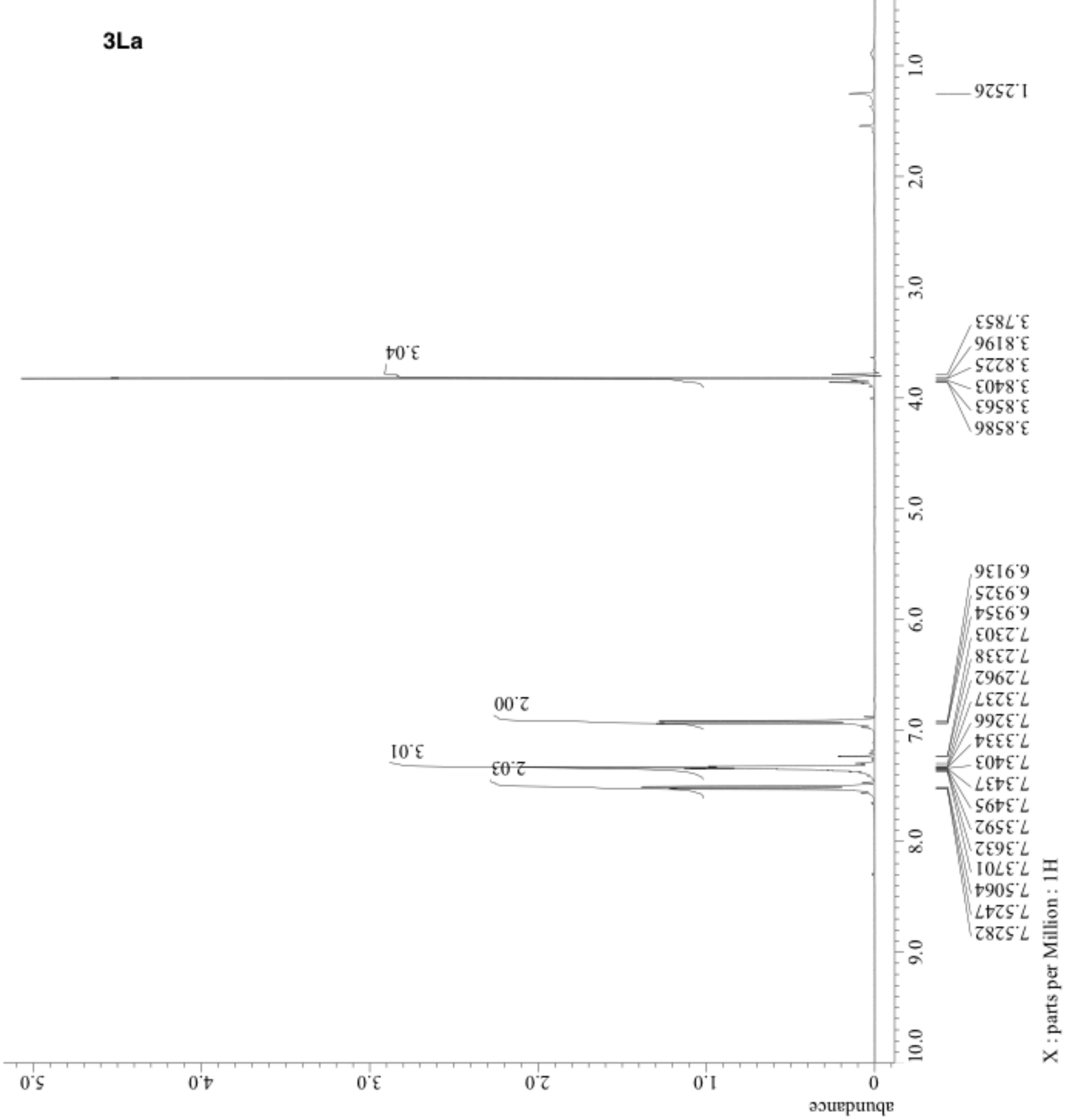


**Supplementary Figure 51.** **$^{1}$H NMR (400 MHz, $CDCl_3$) of 3La**

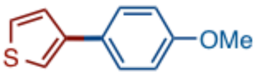


**3La**

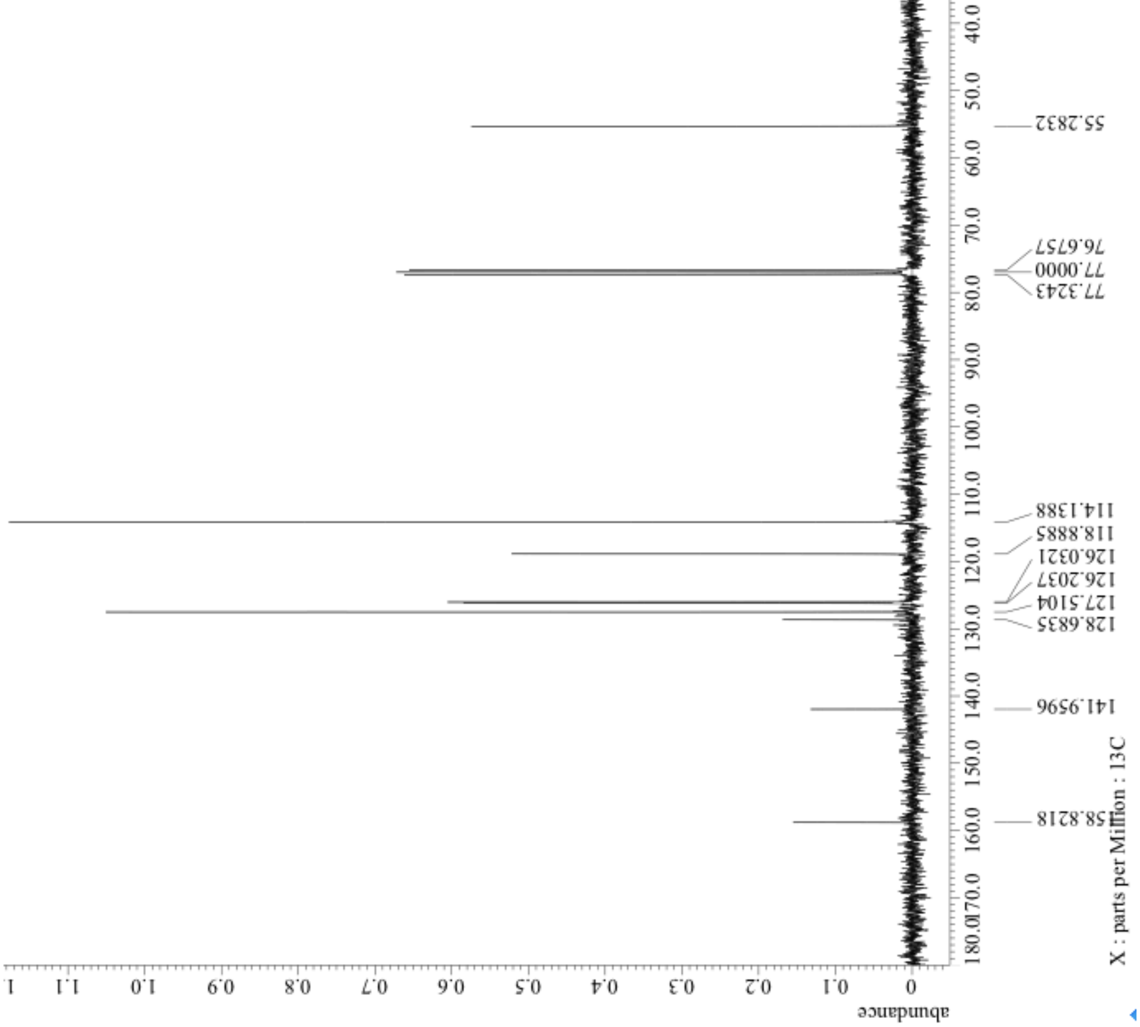


**Supplementary Figure 52. $^{13}C$ NMR (100 MHz, $CDCl_3$) of 3La**

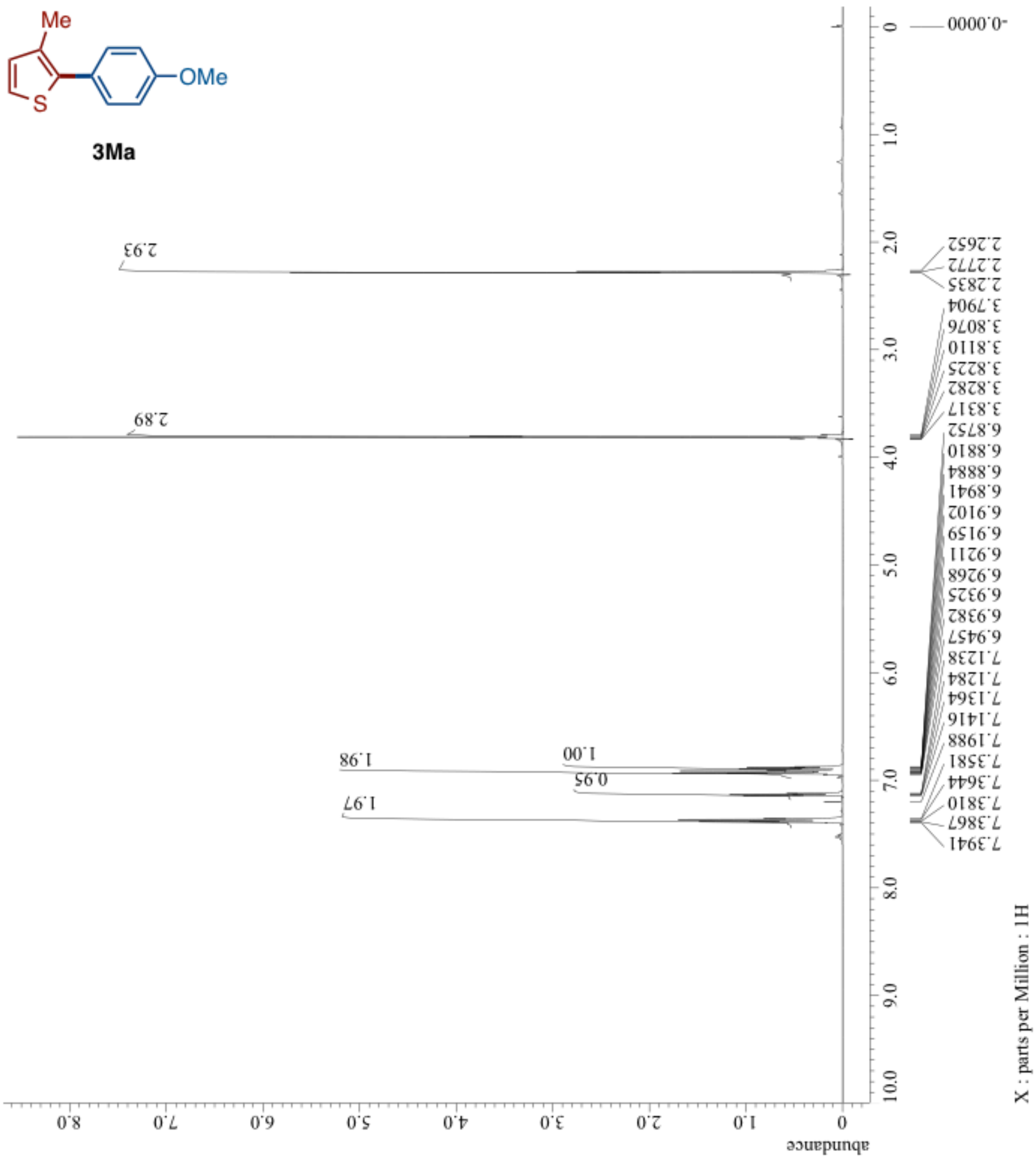


**Supplementary Figure 53. $^{1}$H NMR (400 MHz, $CDCl_3$) of 3Ma**

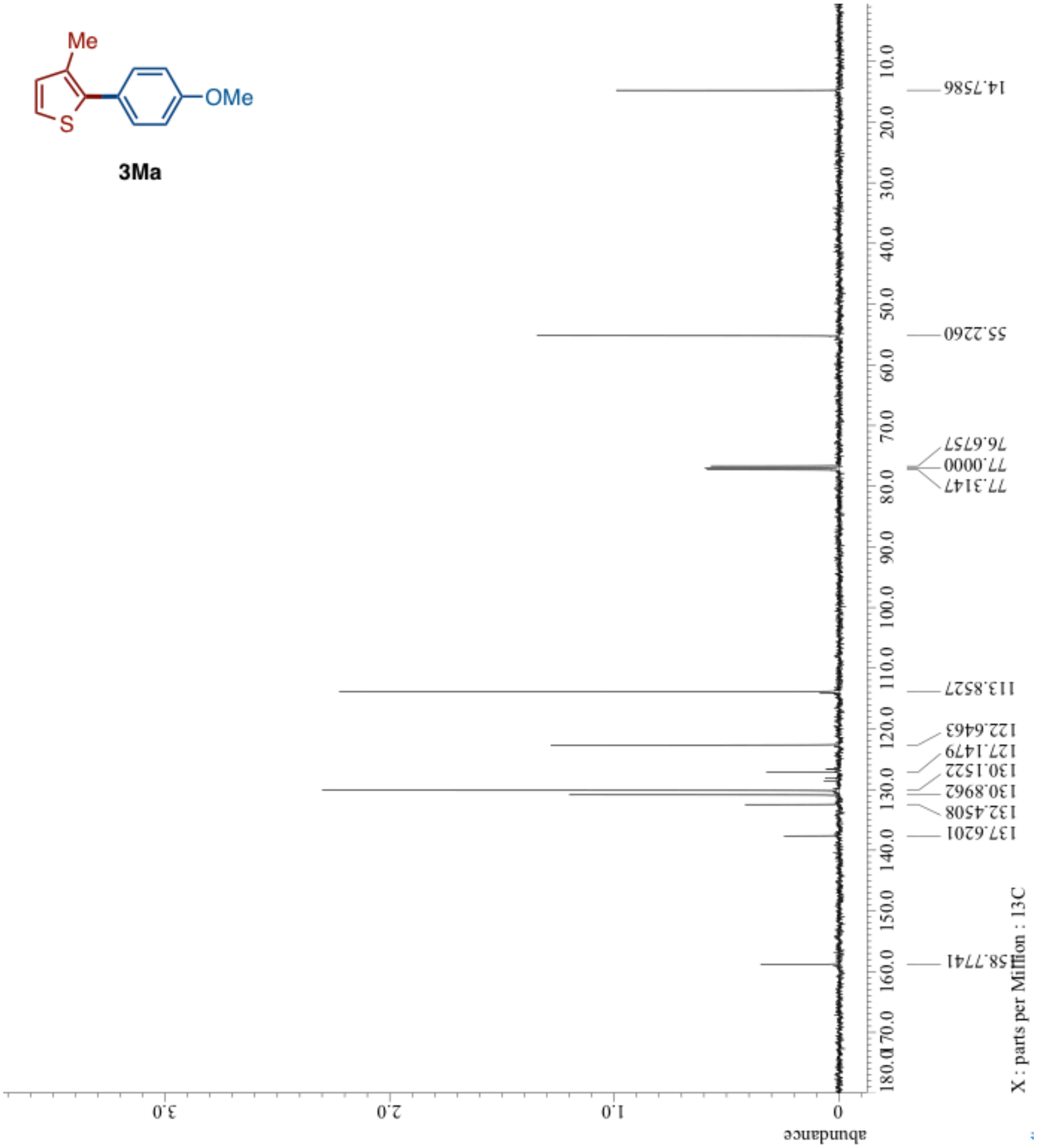


**Supplementary Figure 54.** $^{13}$**C NMR (100 MHz, CDCl$_3$) of 3Ma**

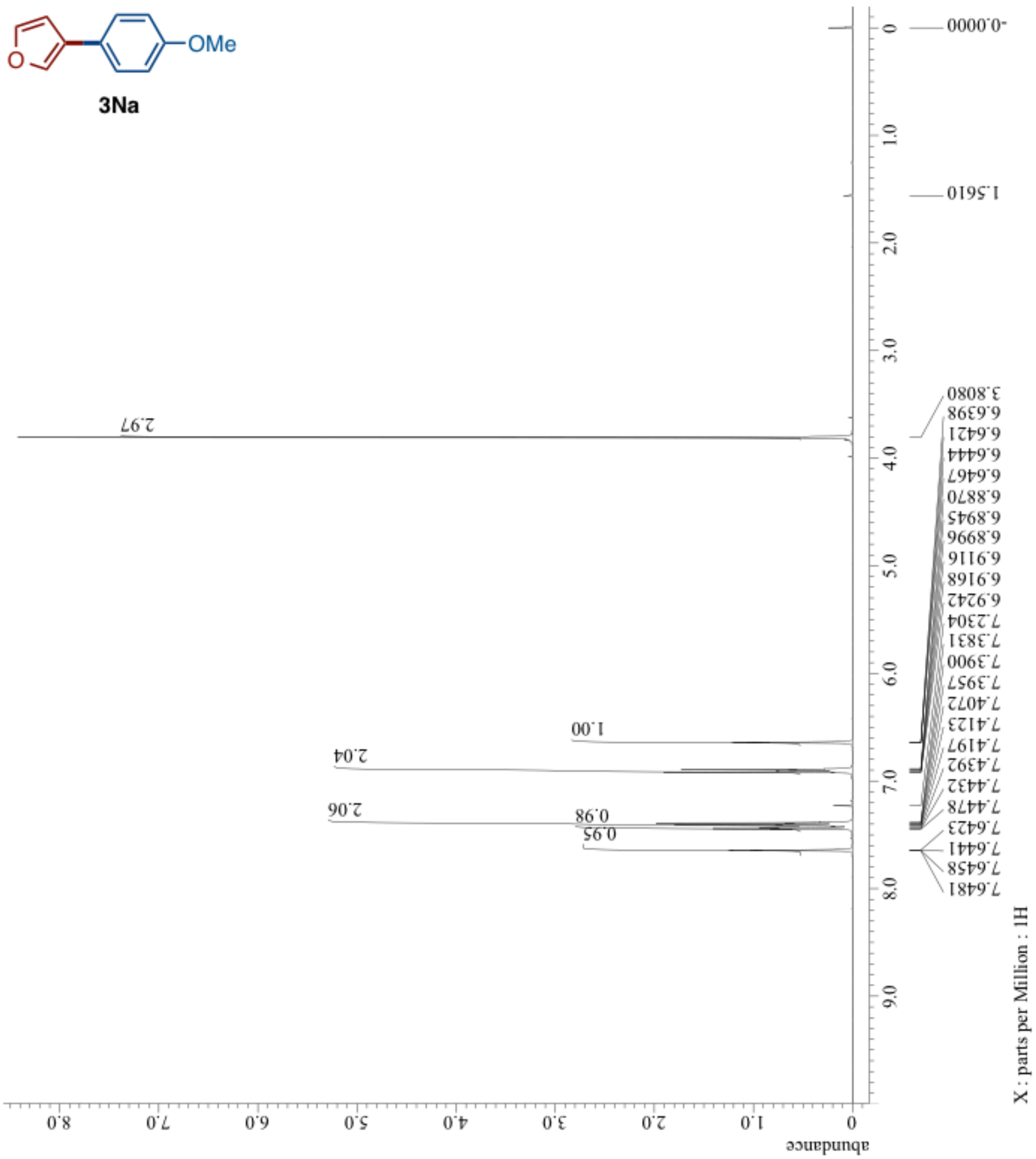


**Supplementary Figure 55.** $^{1}$**H NMR (400 MHz,** $CDCl_3$**) of 3Na**

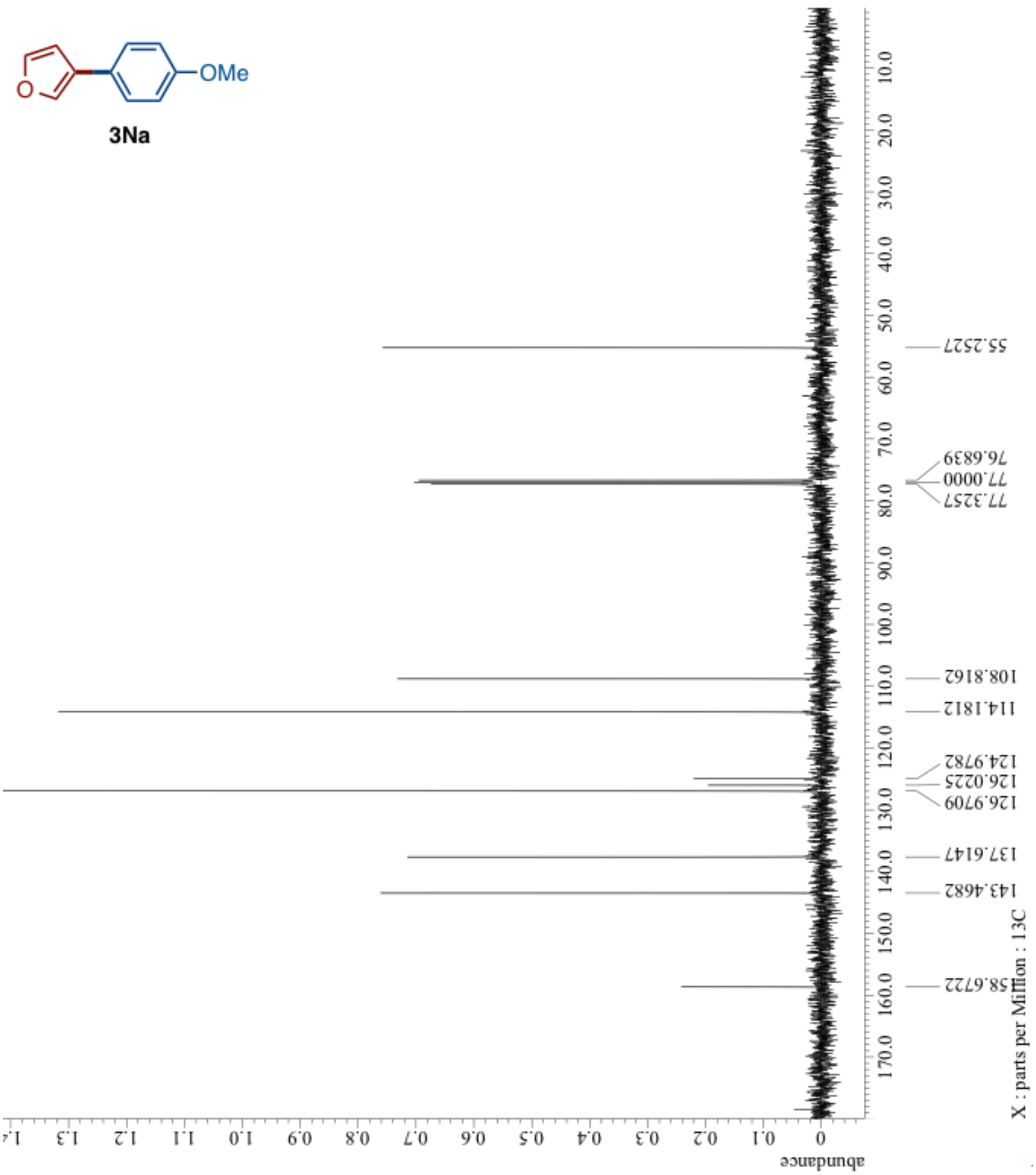


**Supplementary Figure 56. $^{13}$C NMR (100 MHz, $CDCl_3$) of 3Na**

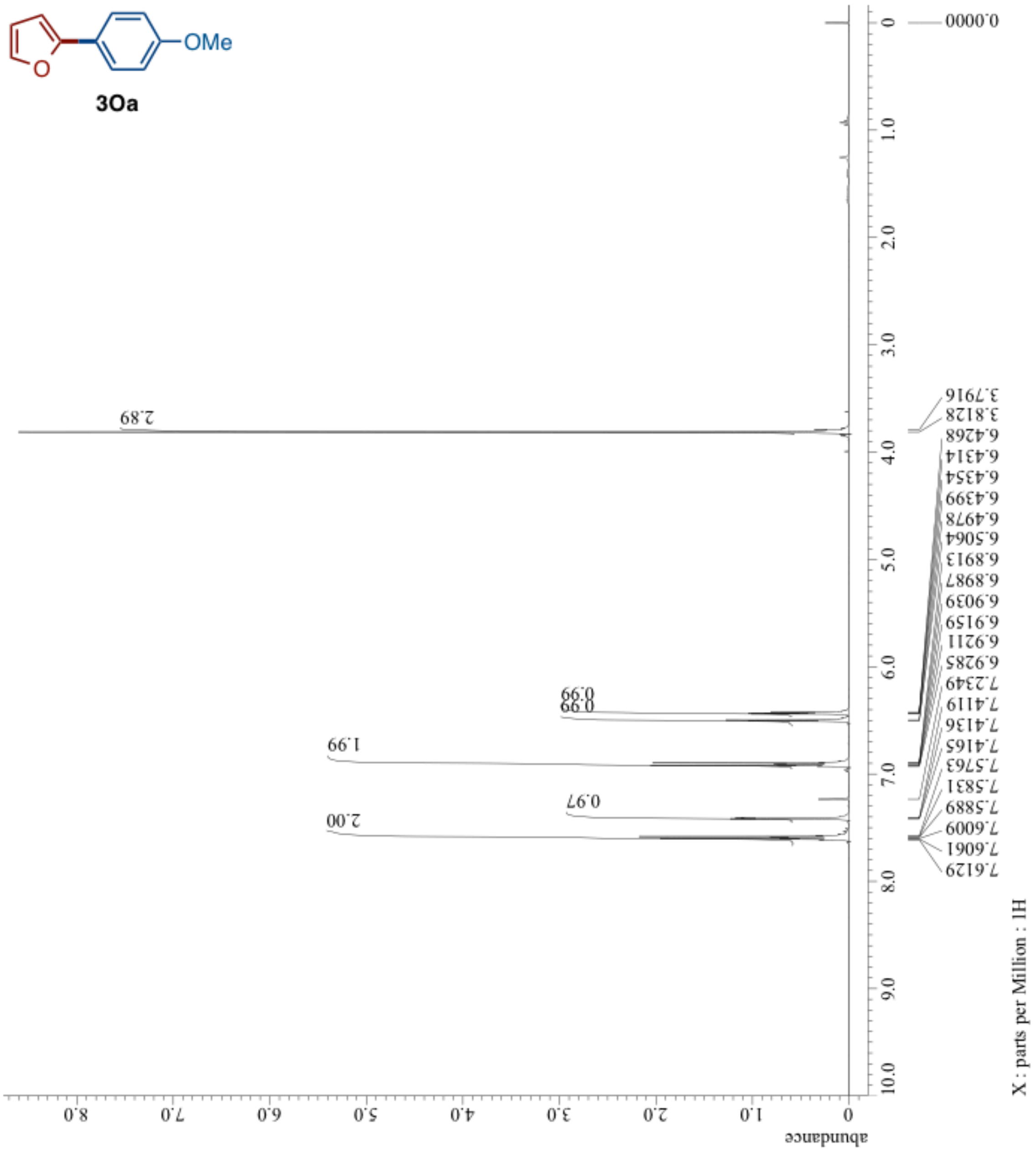


**Supplementary Figure 57. $^{1}H$ NMR (400 MHz, $CDCl_3$) of 3Oa**

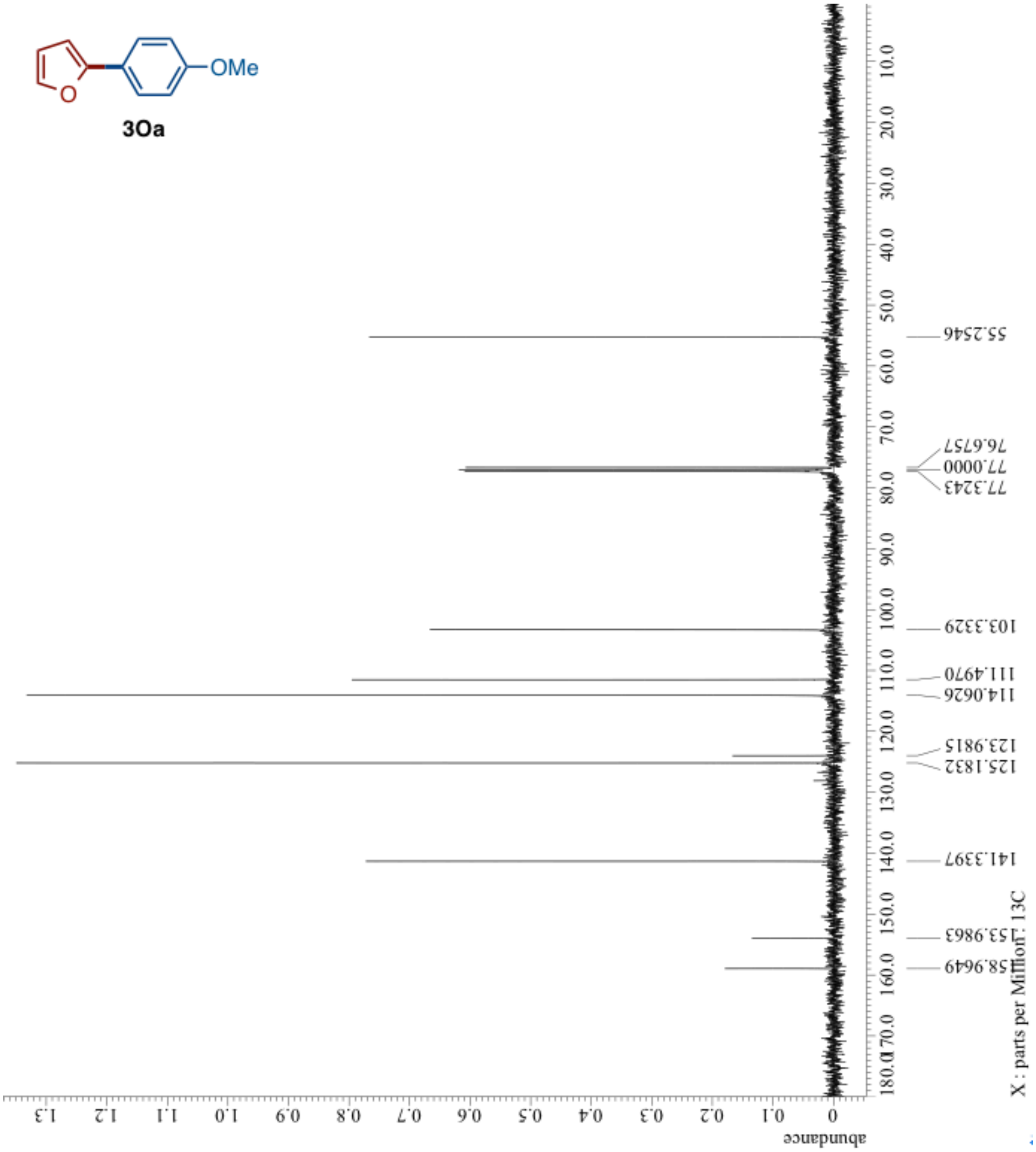


**Supplementary Figure 58.** $^{13}$**C NMR (100 MHz,** $CDCl_3$**) of 3Oa**

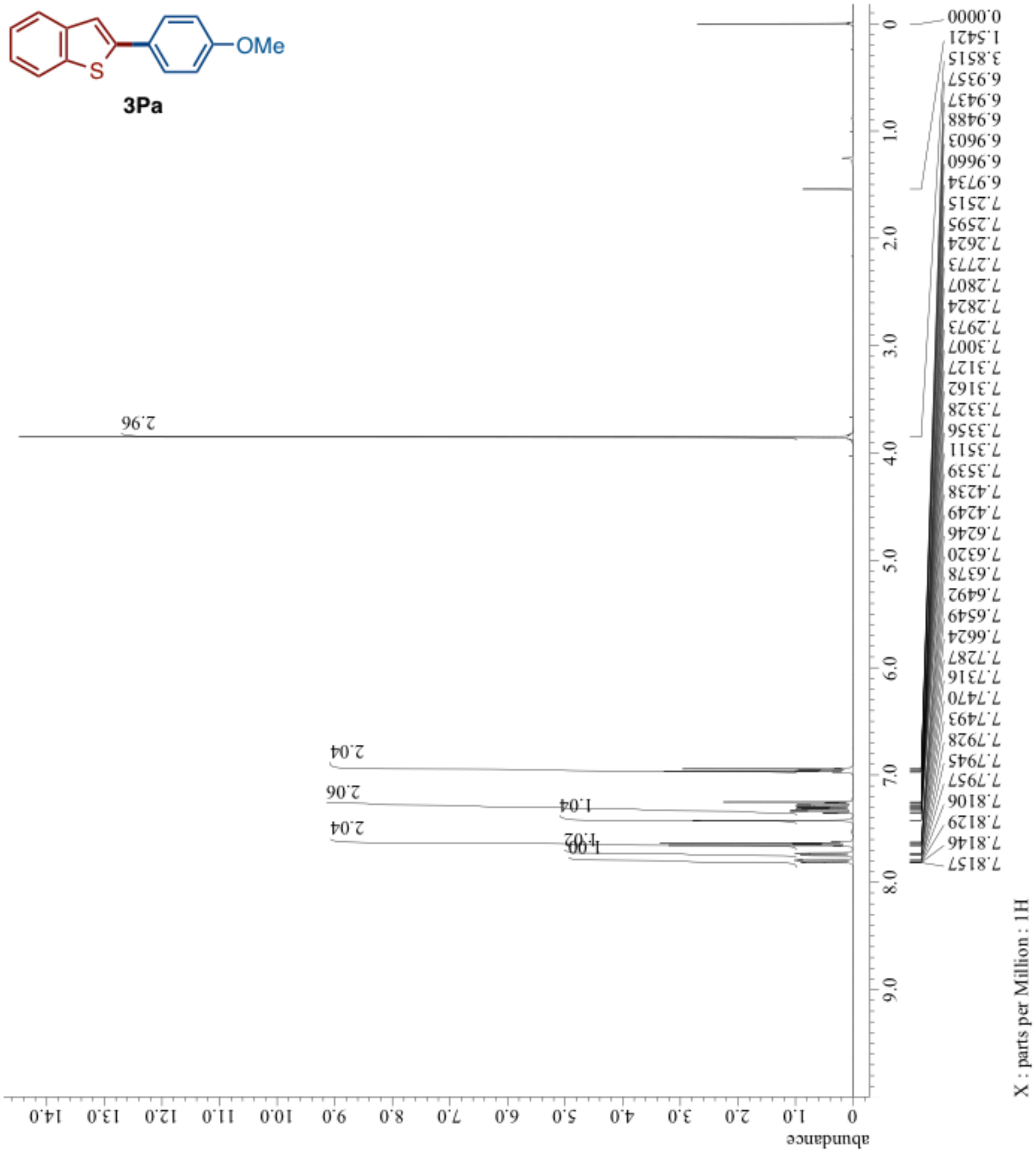


**3Pa**



**Supplementary Figure 59. $^{1}$H NMR (400 MHz, $CDCl_3$) of 3Pa**

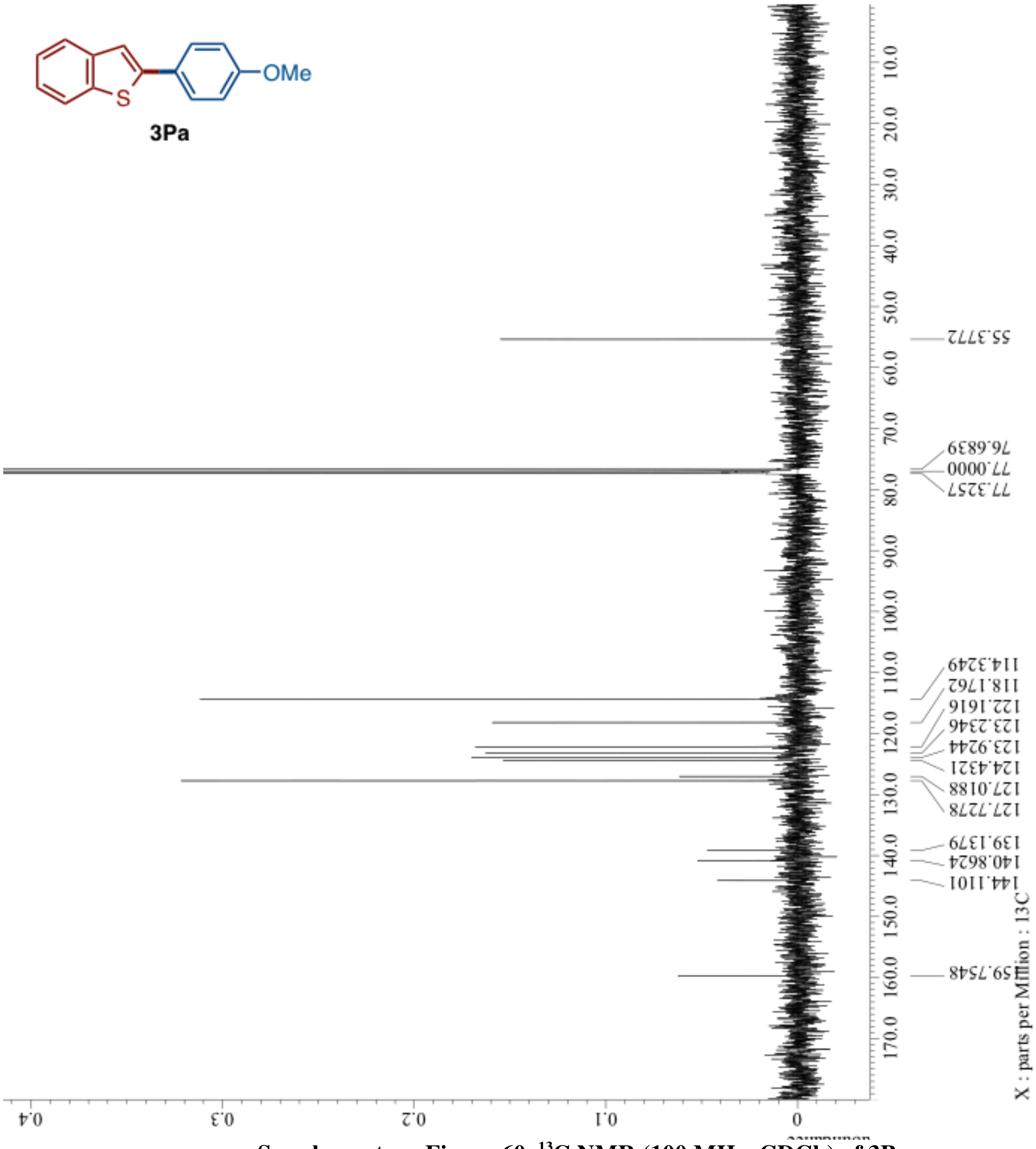


**Supplementary Figure 60. $^{13}C$ NMR (100 MHz, $CDCl_3$) of 3Pa**

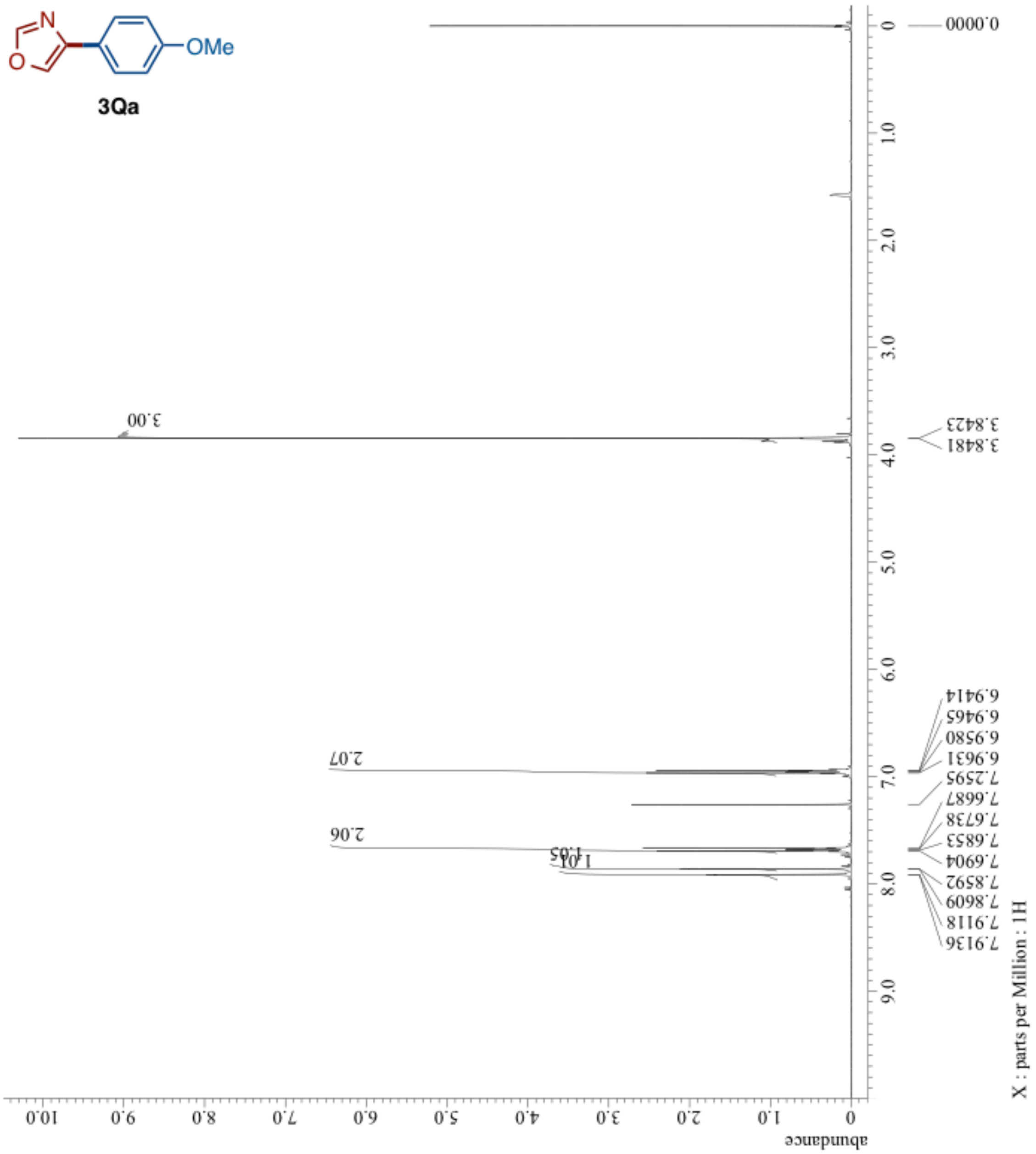


**Supplementary Figure 61.** $^{1}$**H NMR (400 MHz, CDCl$_3$) of 3Qa**

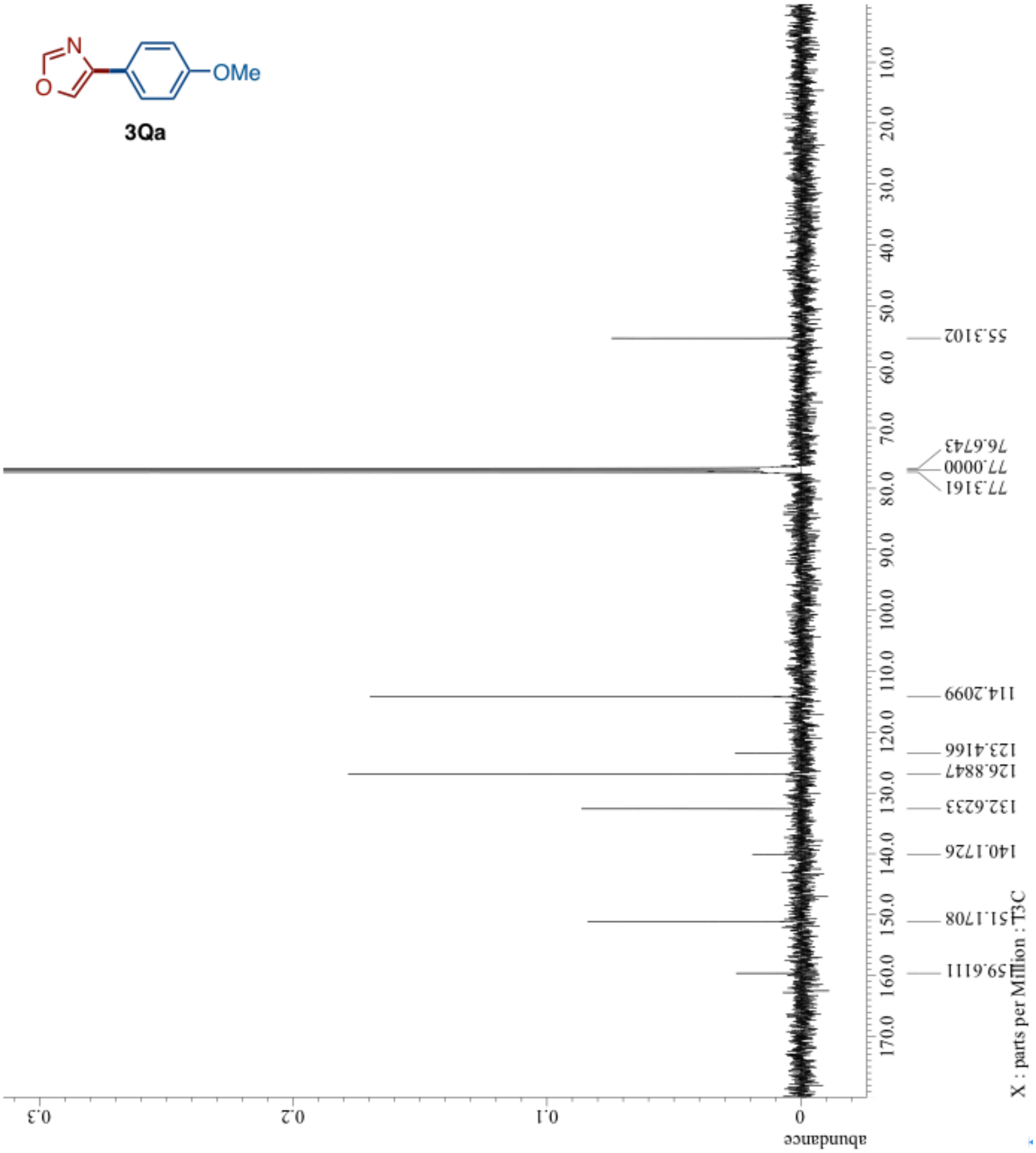


**Supplementary Figure 62.** $^{13}$**C NMR (100 MHz, CDCl$_3$) of 3Qa**

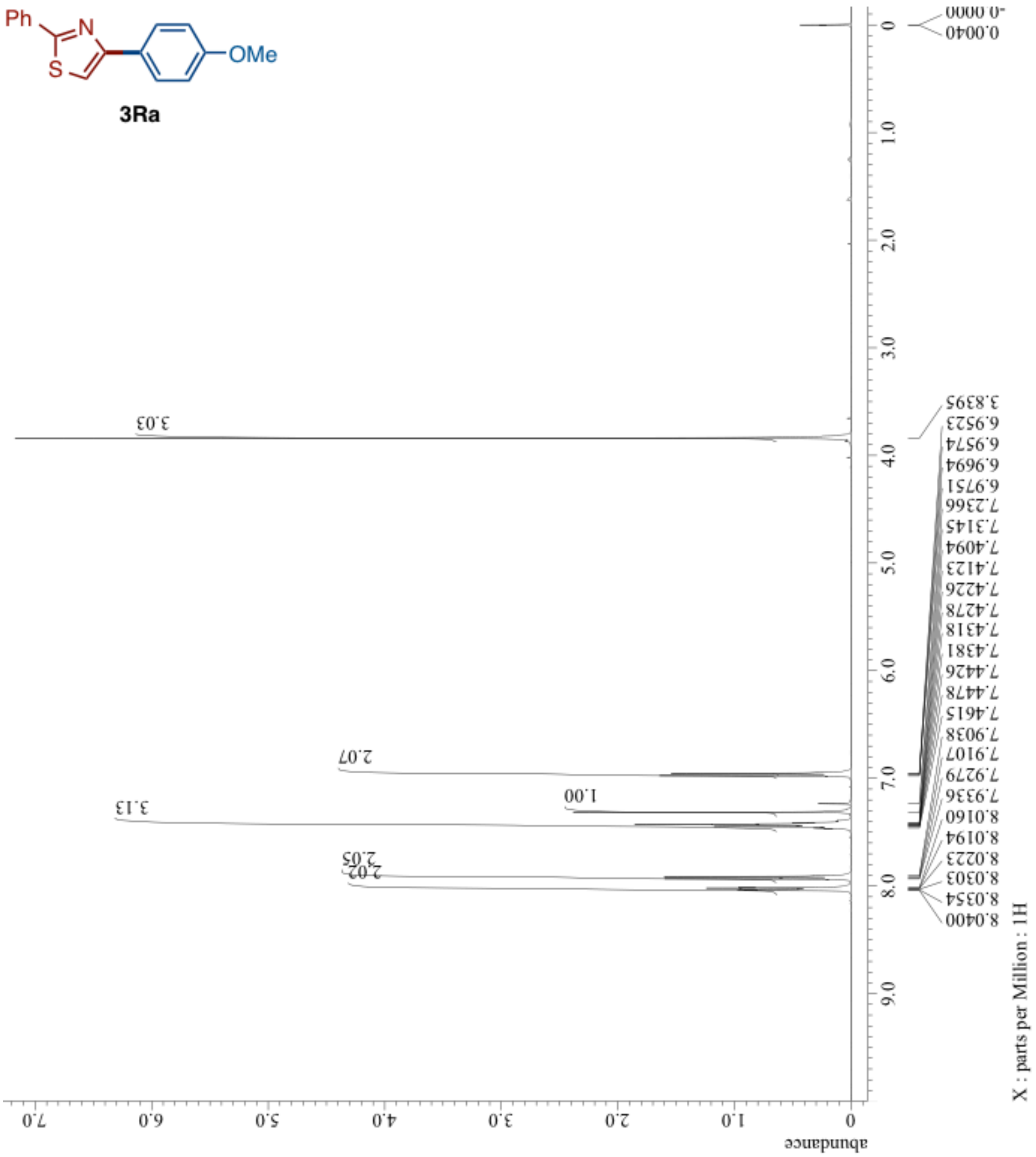


**Supplementary Figure 63.** $^{1}$**H NMR (400 MHz, CDCl$_3$) of 3Ra**

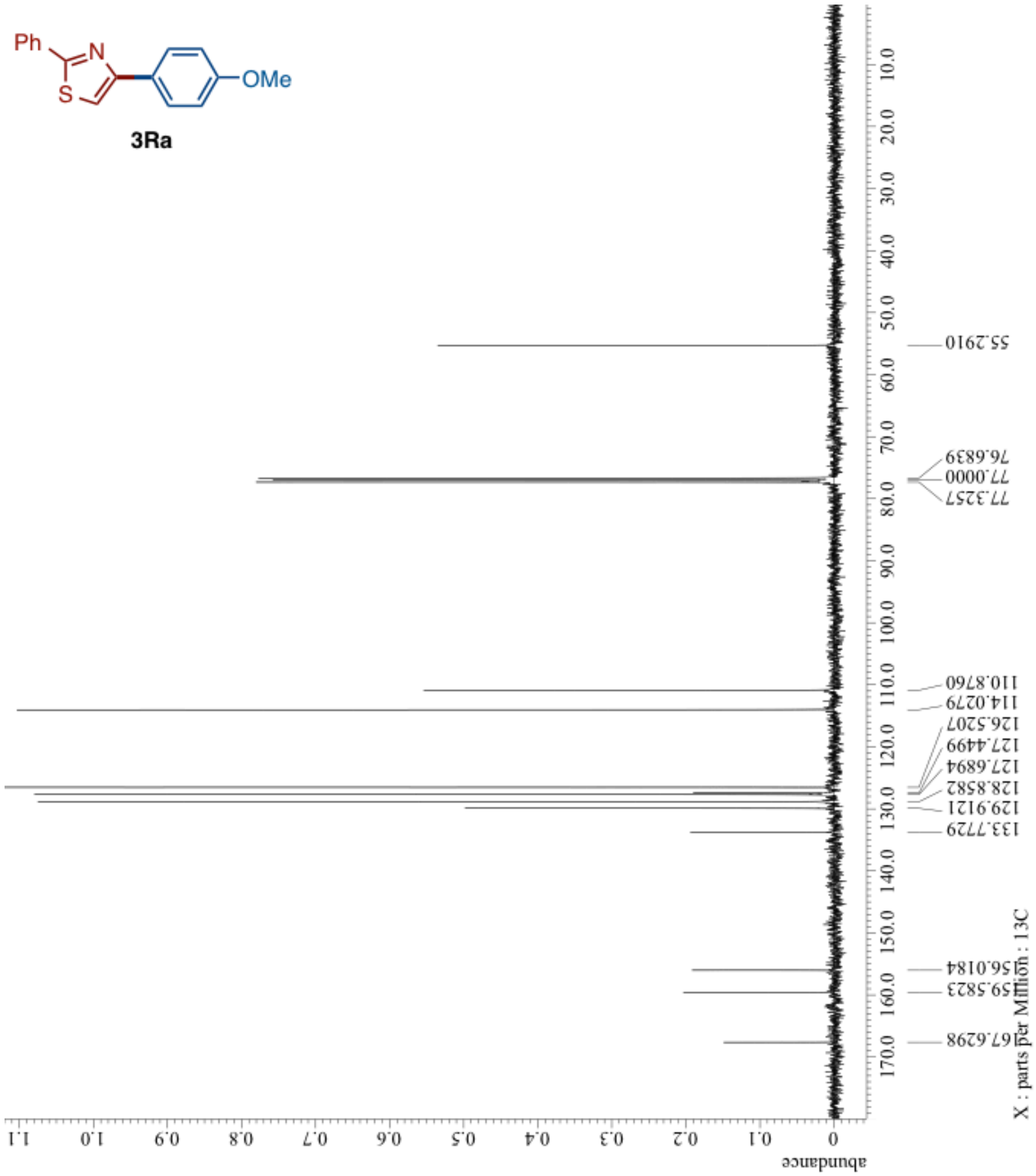


**Supplementary Figure 64. $^{13}C$ NMR (100 MHz, $CDCl_3$) of 3Ra**

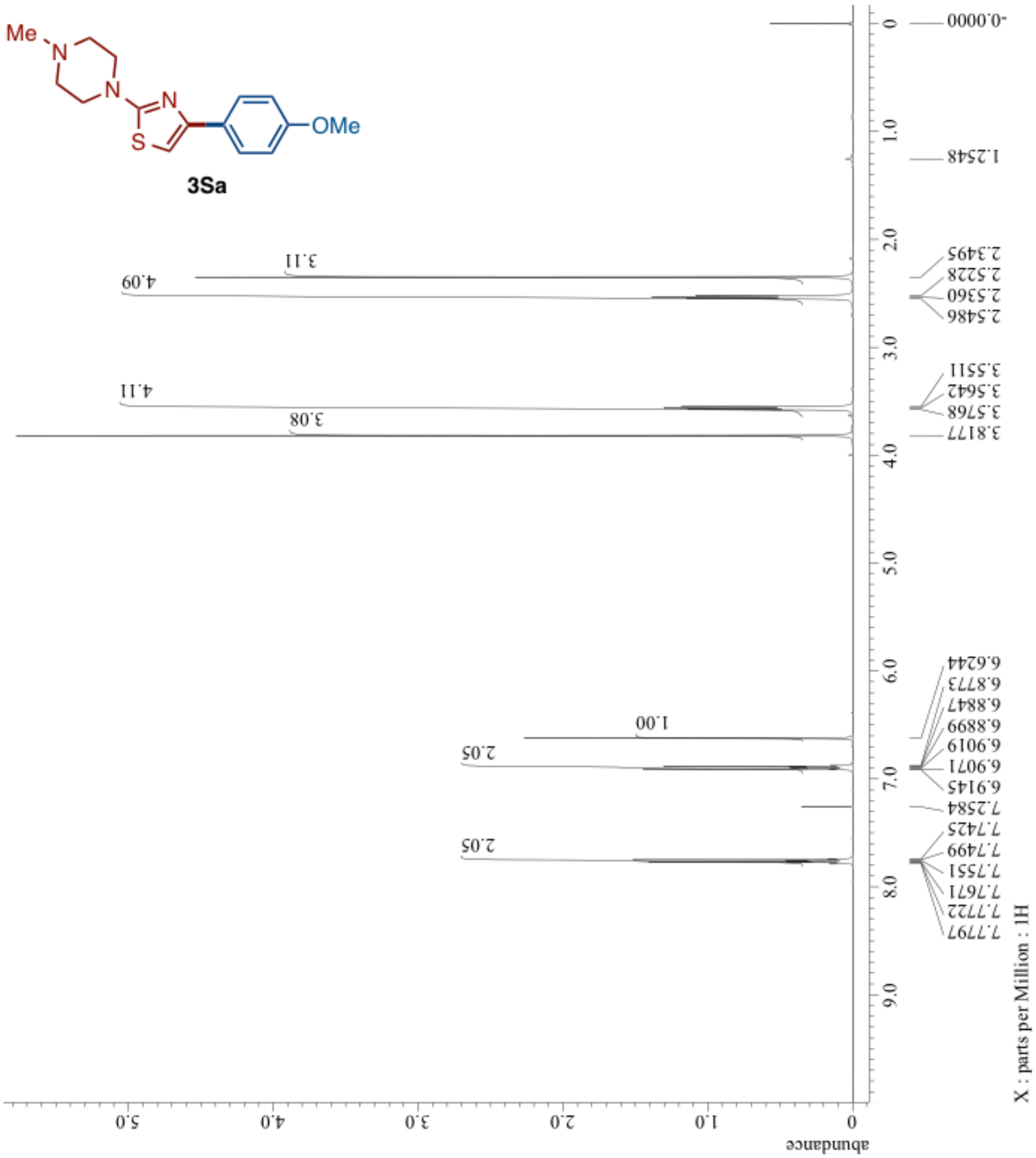


Supplementary Figure 65. $^{1}$H NMR (400 MHz, $CDCl_3$) of 3Sa

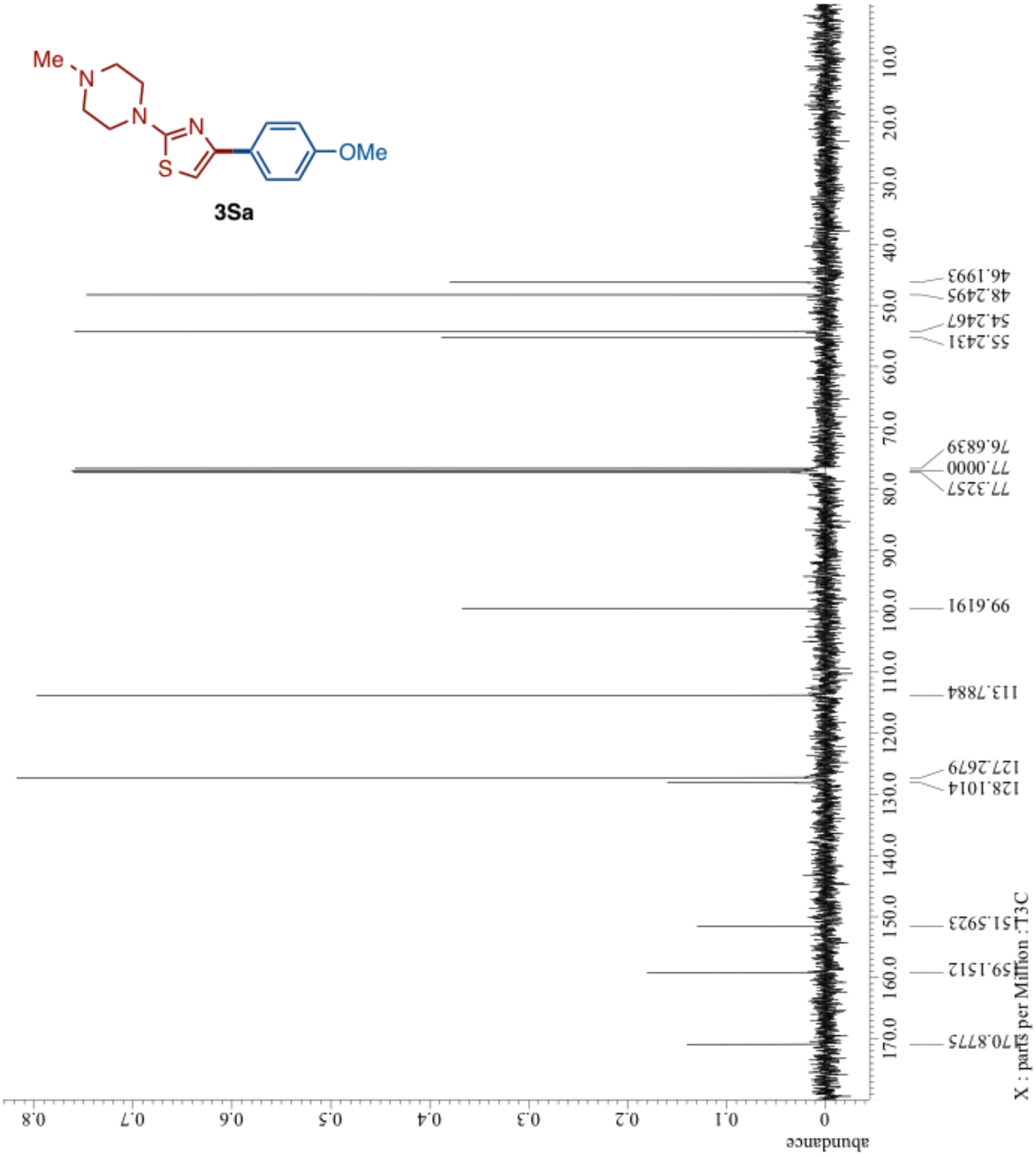


**Supplementary Figure 66. $^{13}$C NMR (100 MHz, $CDCl_3$) of 3Sa**

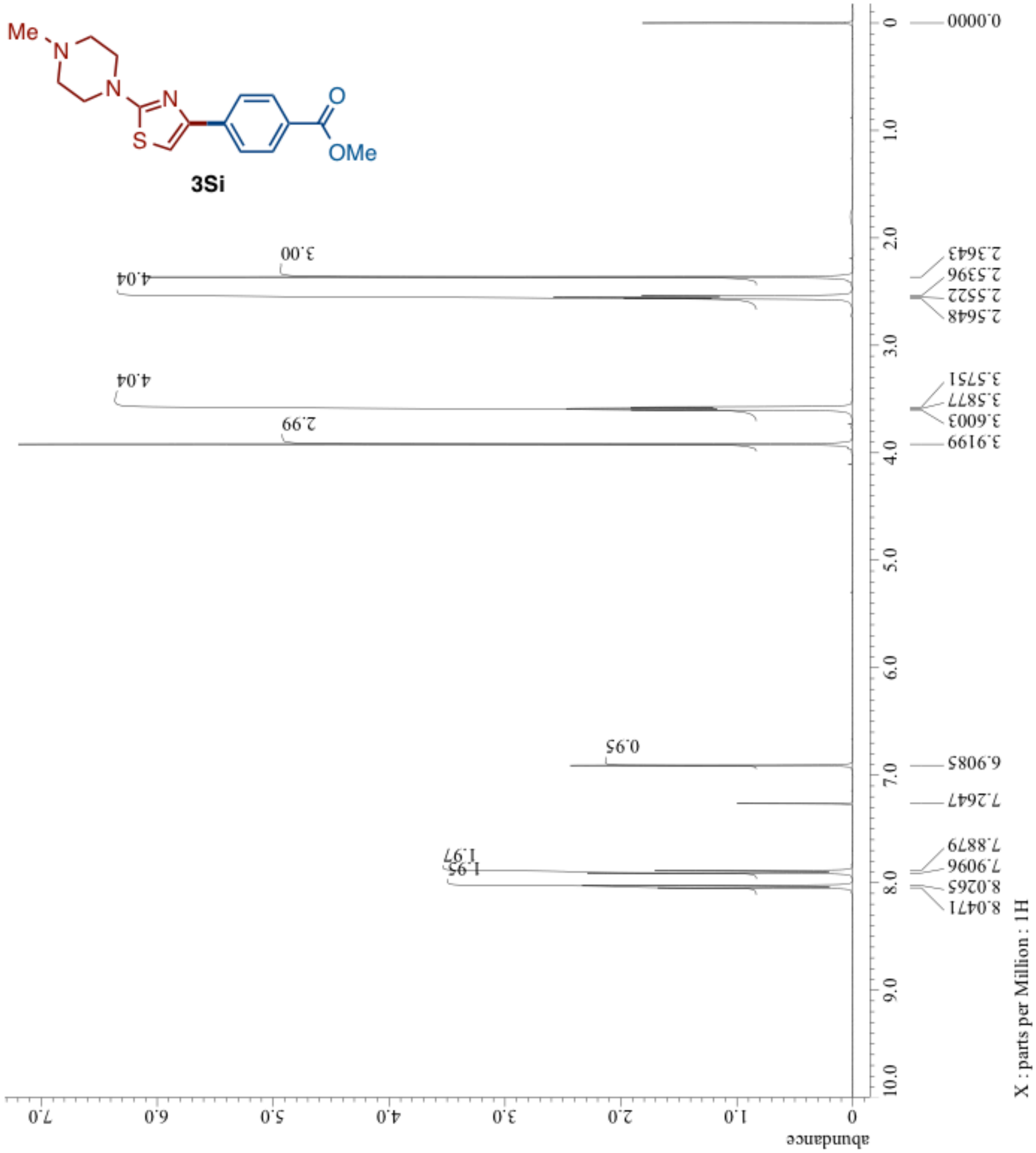


**Supplementary Figure 67. $^{1}$H NMR (400 MHz, $CDCl_3$) of 3Si**

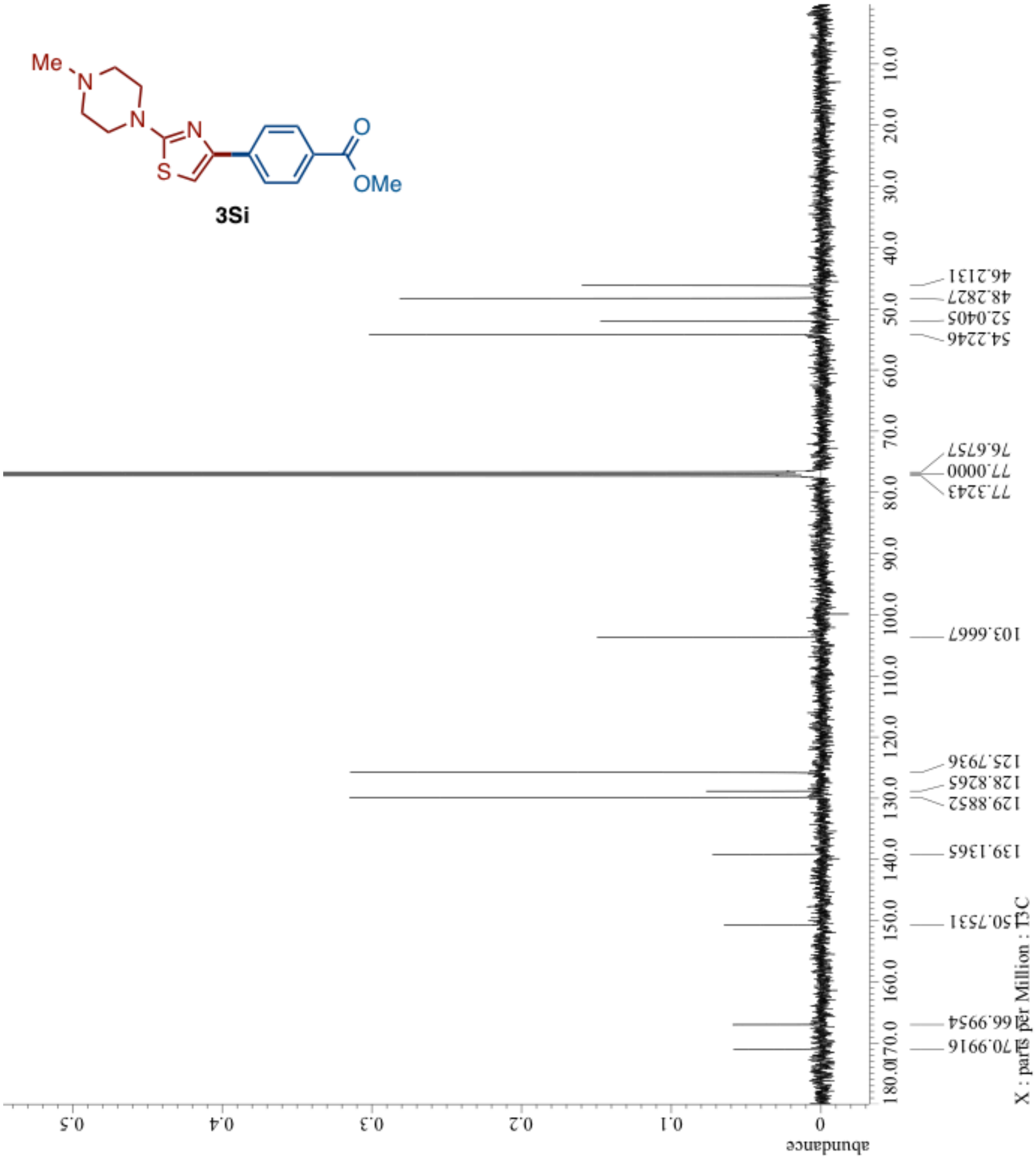


**Supplementary Figure 68.** **$^{13}$C NMR (100 MHz, $CDCl_3$) of 3Si**

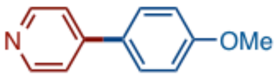


3Ta

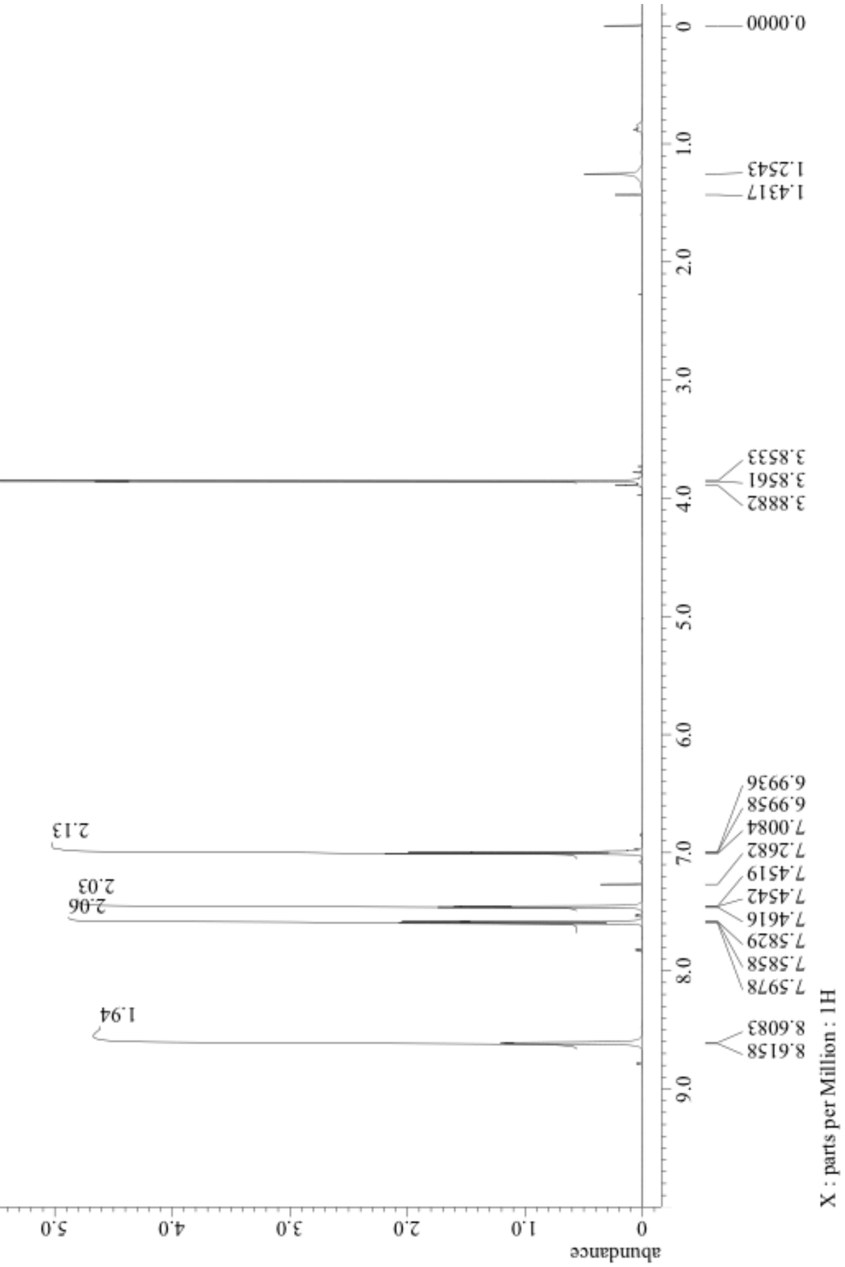

**Supplementary Figure 69. $^1$H NMR (600 MHz, $CDCl_3$) of 3Ta**

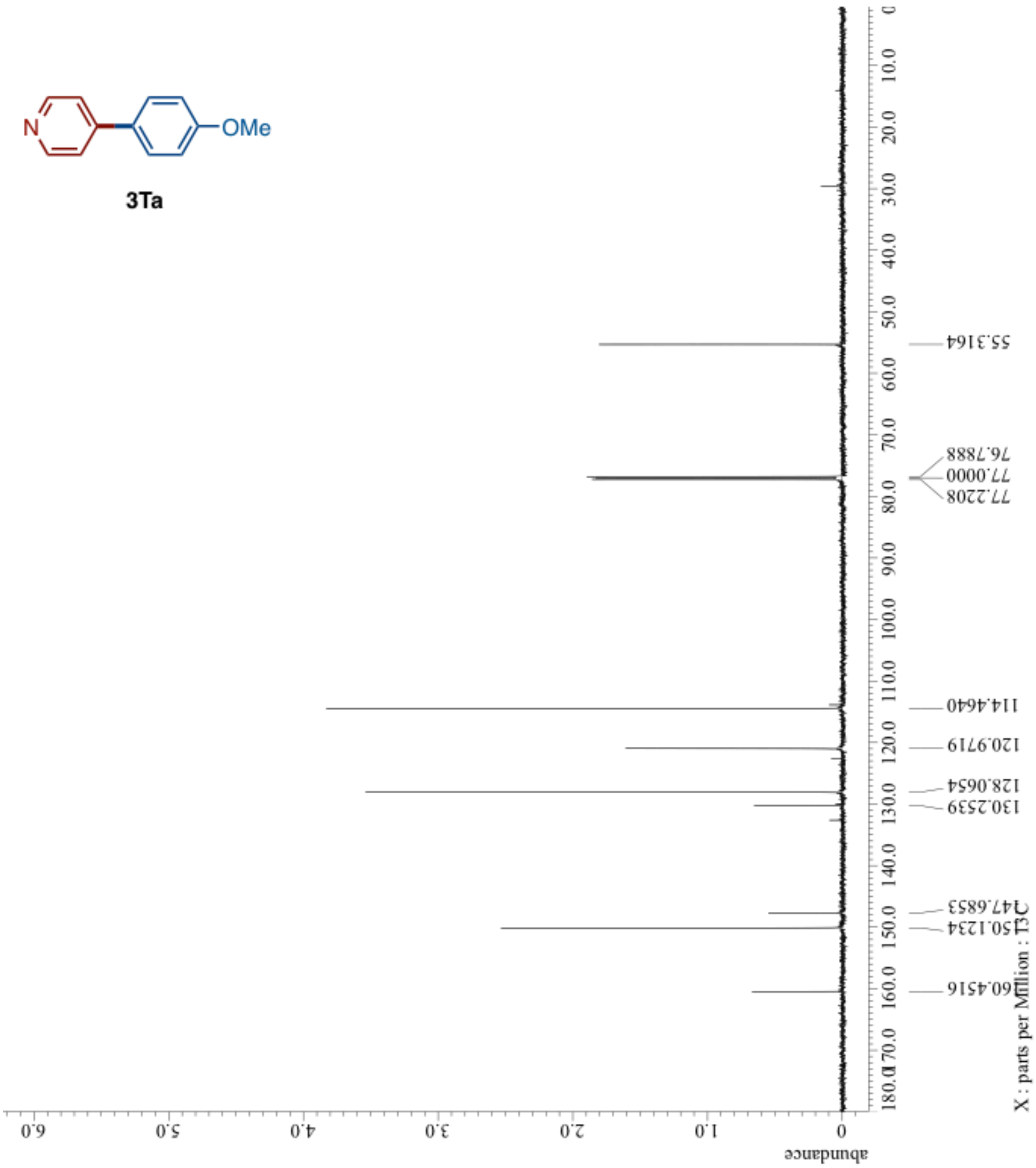


**Supplementary Figure 70.** $^{13}C$ **NMR (150 MHz,** $CDCl_3$**) of 3Ta**

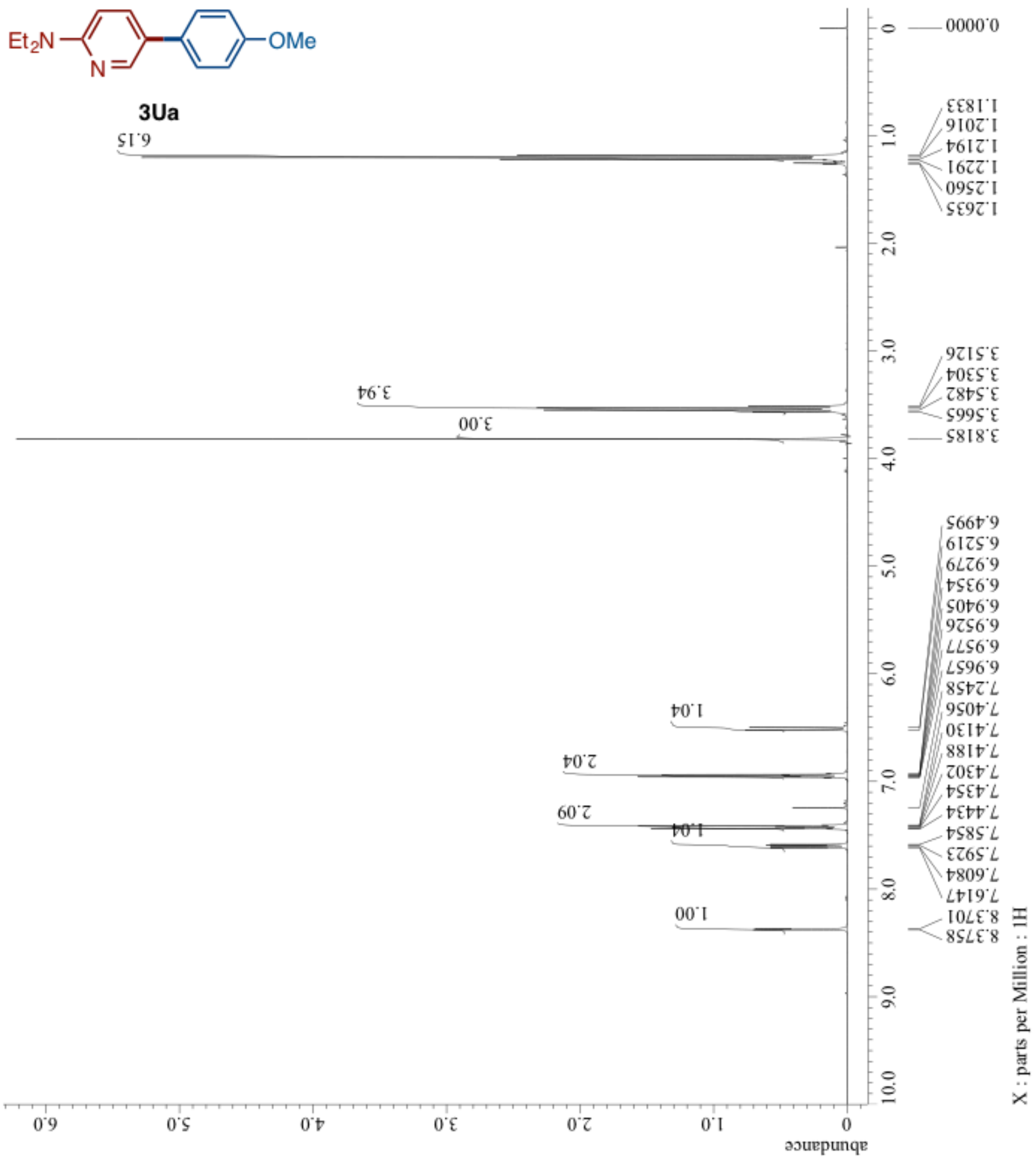

**Supplementary Figure 71.** **$^{1}$H NMR (400 MHz, $CDCl_3$) of 3Ua**

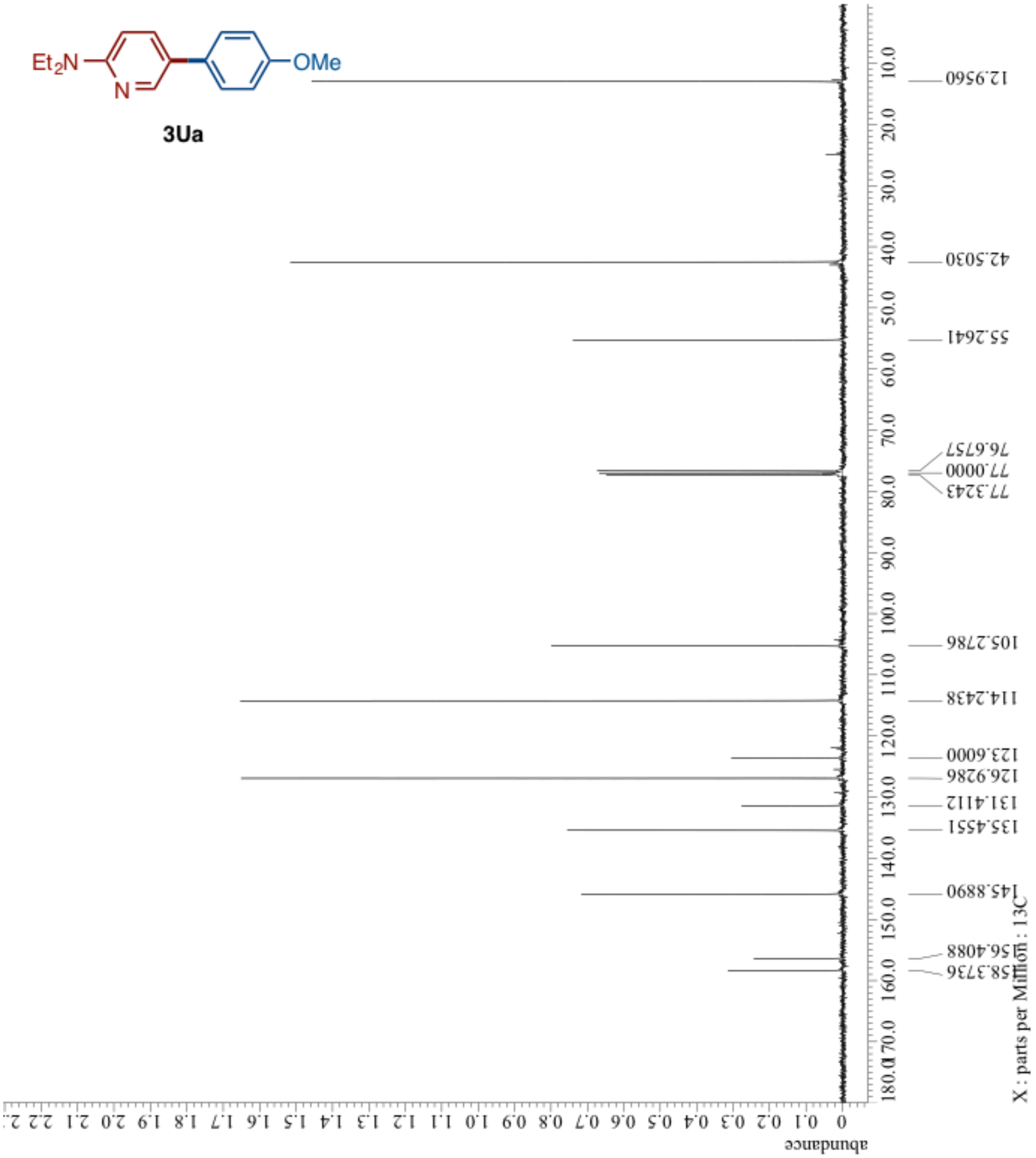


**Supplementary Figure 72.** $^{13}$**C NMR (100 MHz,** $CDCl_3$**) of 3Ua**

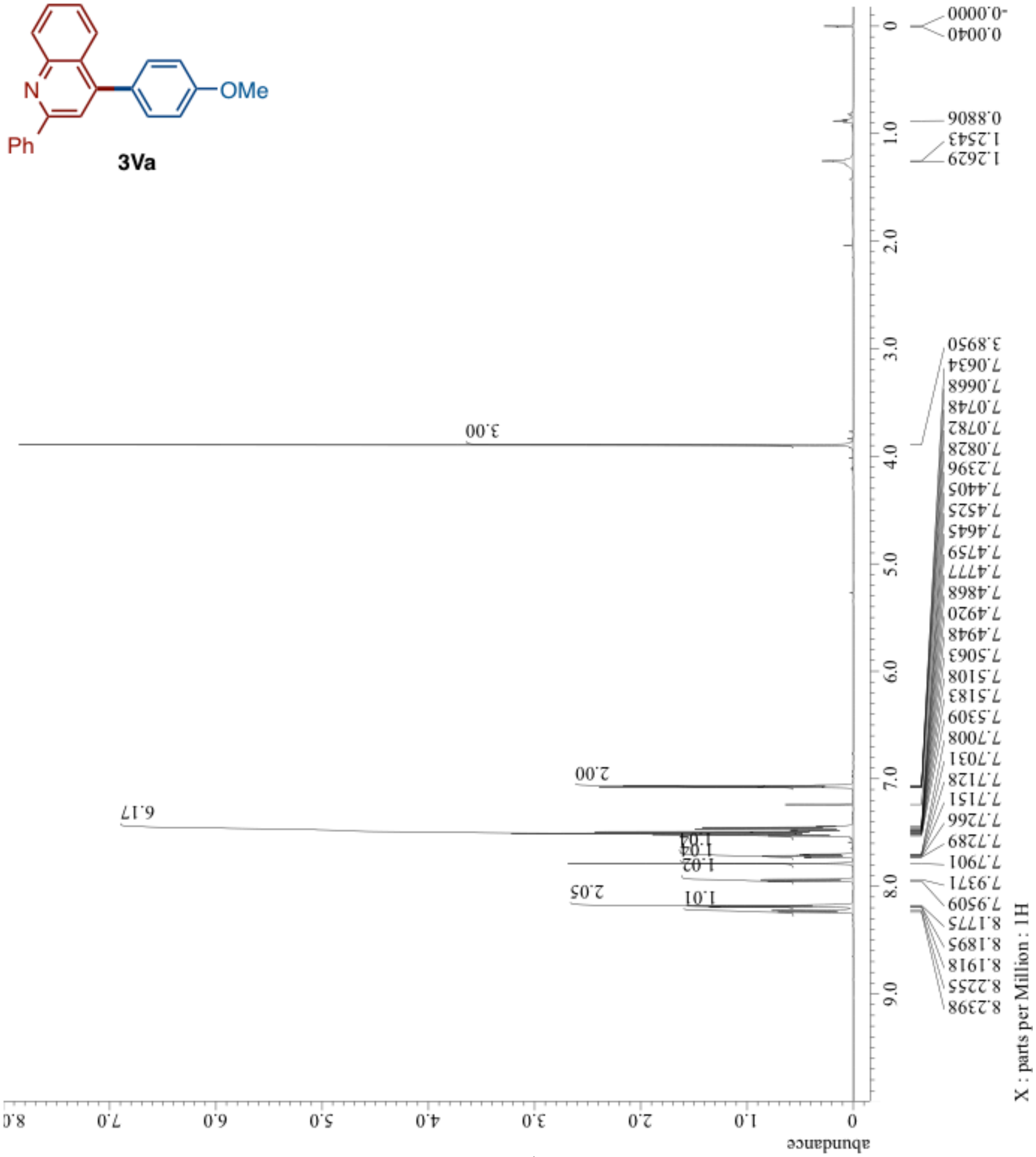


**Supplementary Figure 73.** $^{1}$**H NMR (600 MHz, CDCl$_3$) of 3Va**

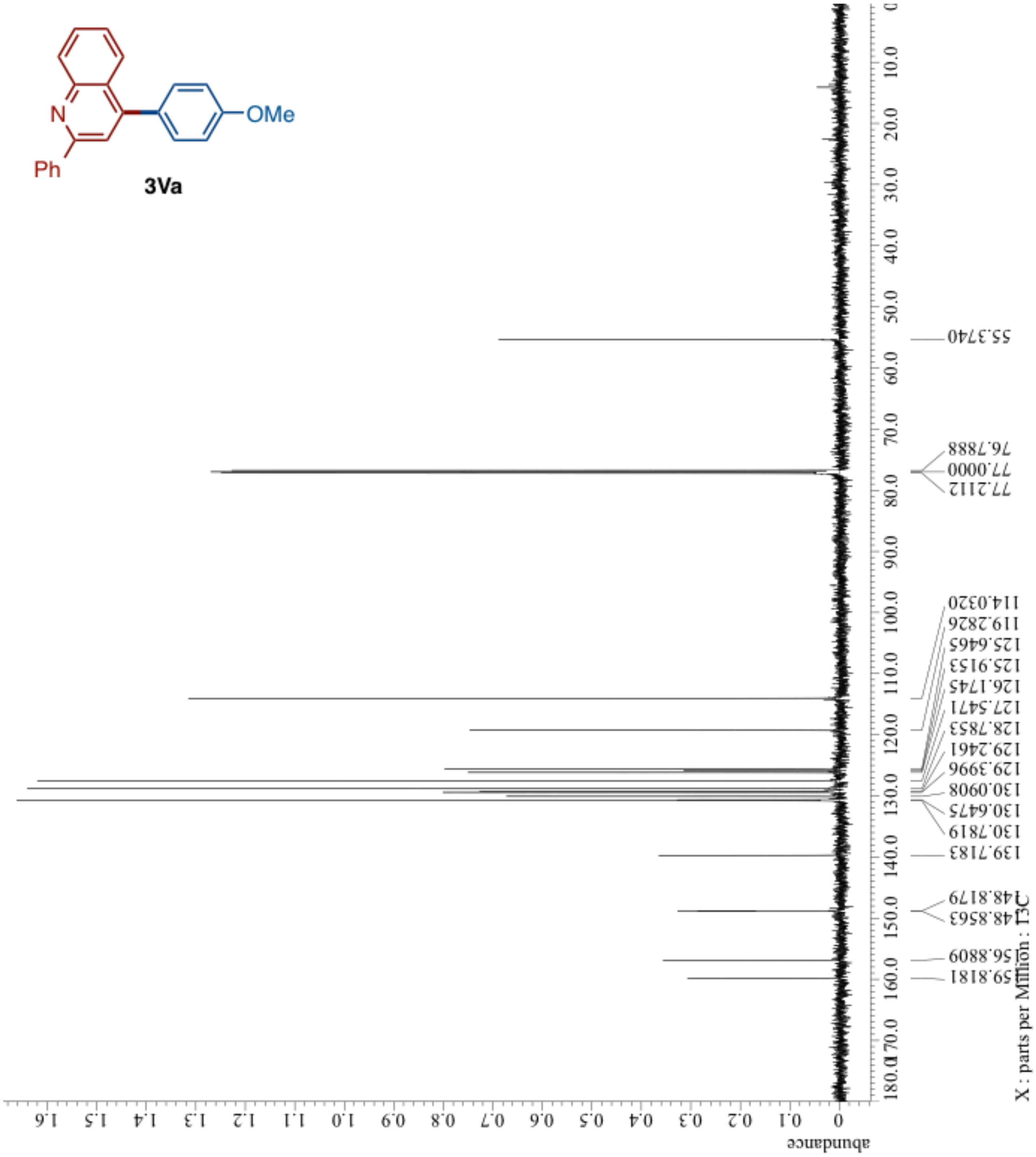


**Supplementary Figure 74. $^{13}C$ NMR (150 MHz, $CDCl_3$) of 3Va**

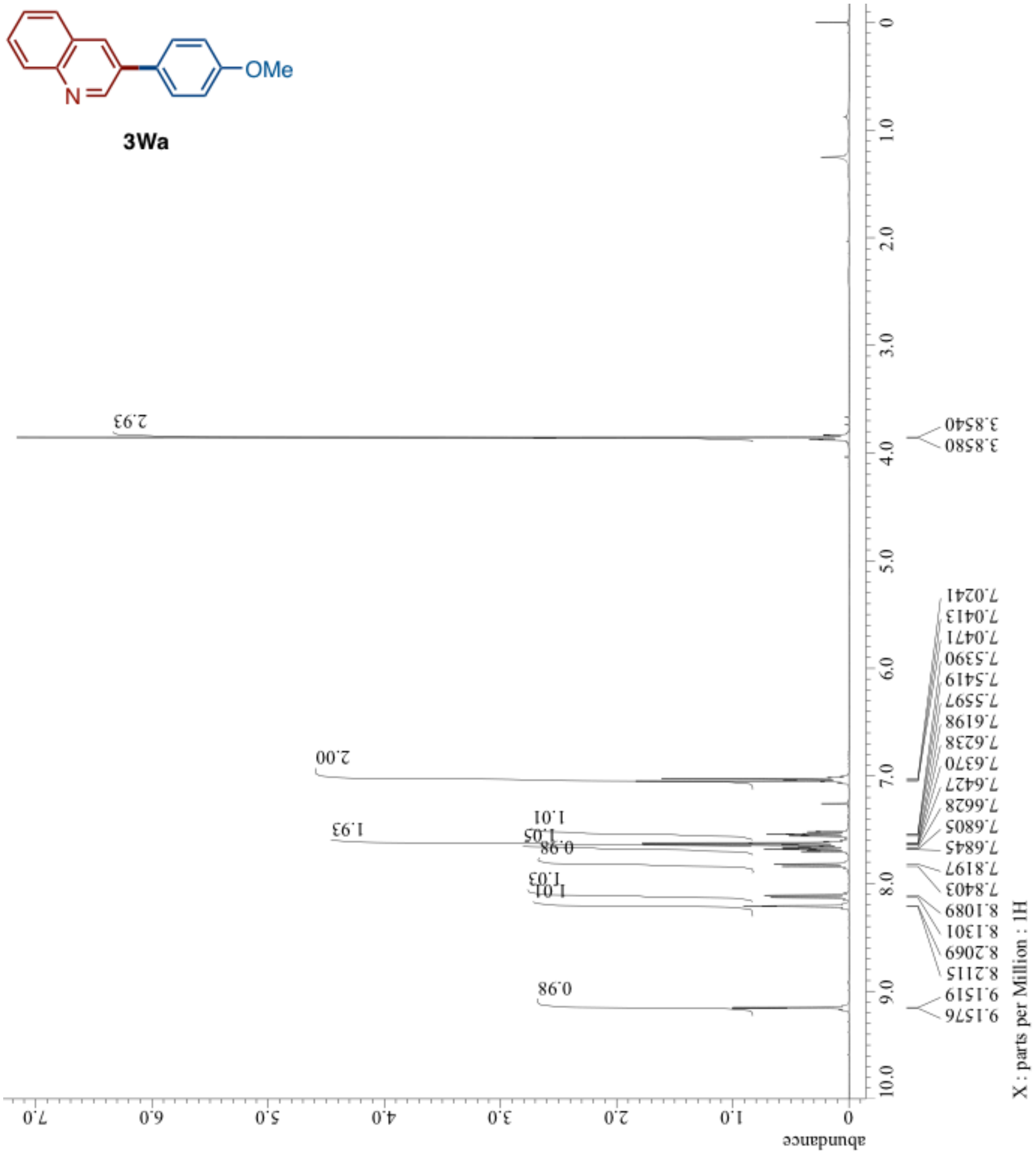


**Supplementary Figure 75. $^{1}$H NMR (400 MHz, $CDCl_3$) of 3Wa**

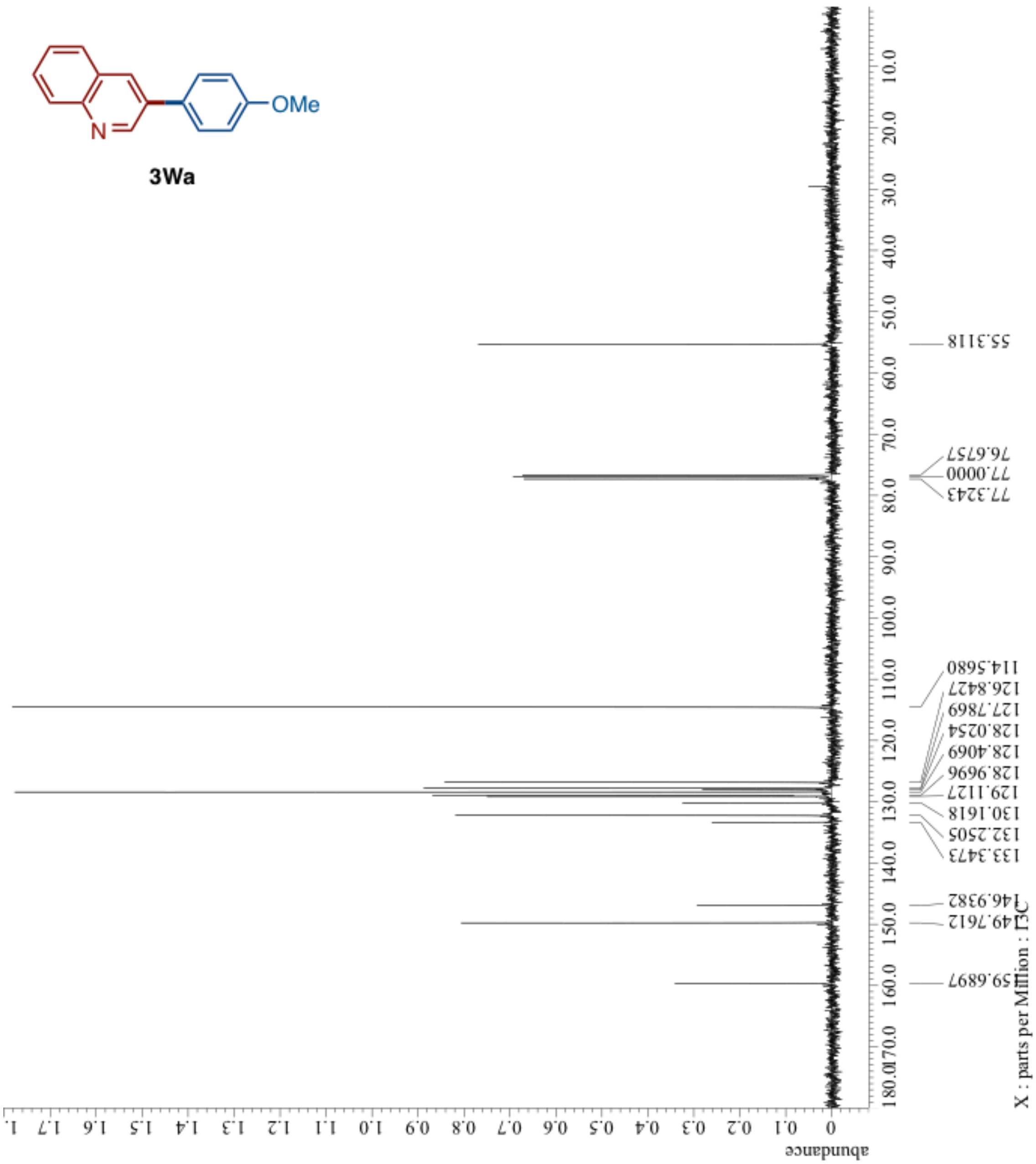


**Supplementary Figure 76.** $^{13}$**C NMR (100 MHz, CDCl$_3$) of 3Wa**

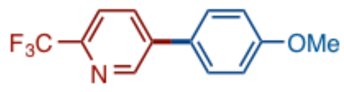


**3Xa**

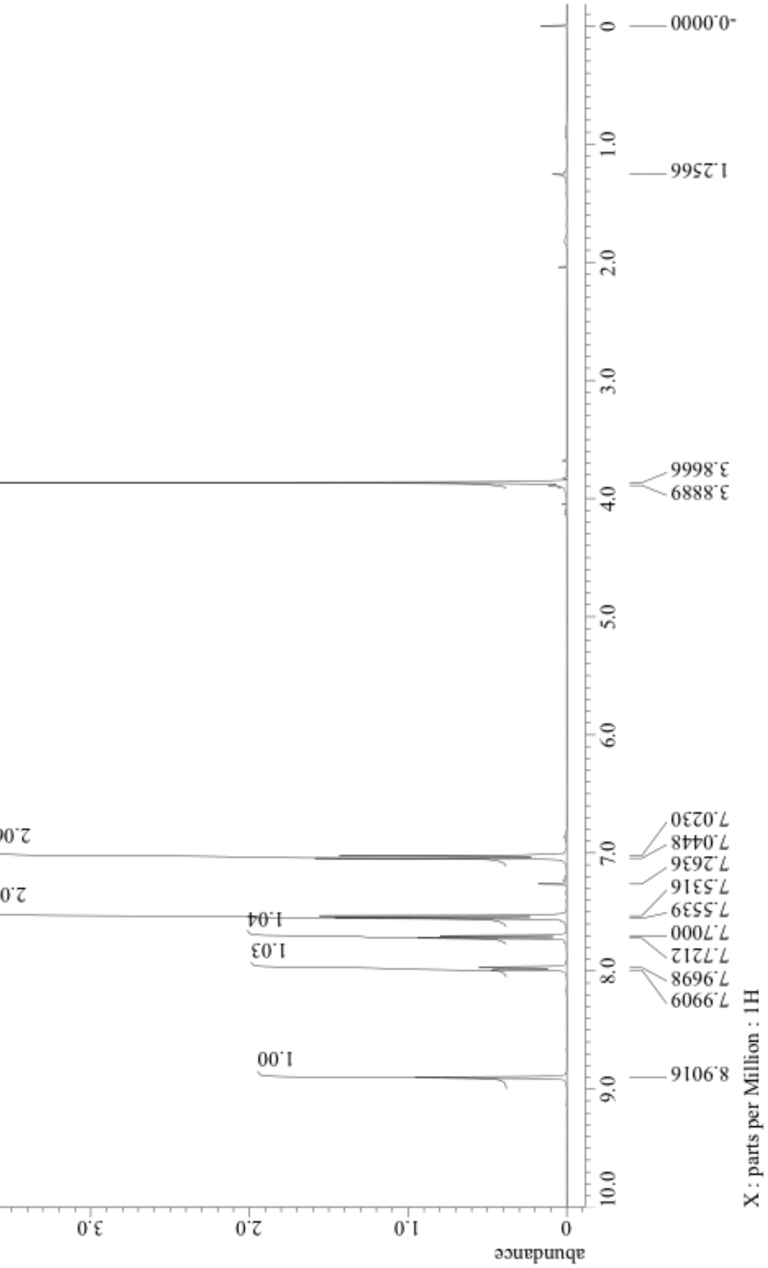


**Supplementary Figure 77.** $^{1}$**H NMR (400 MHz, CDCl$_3$) of 3Xa**

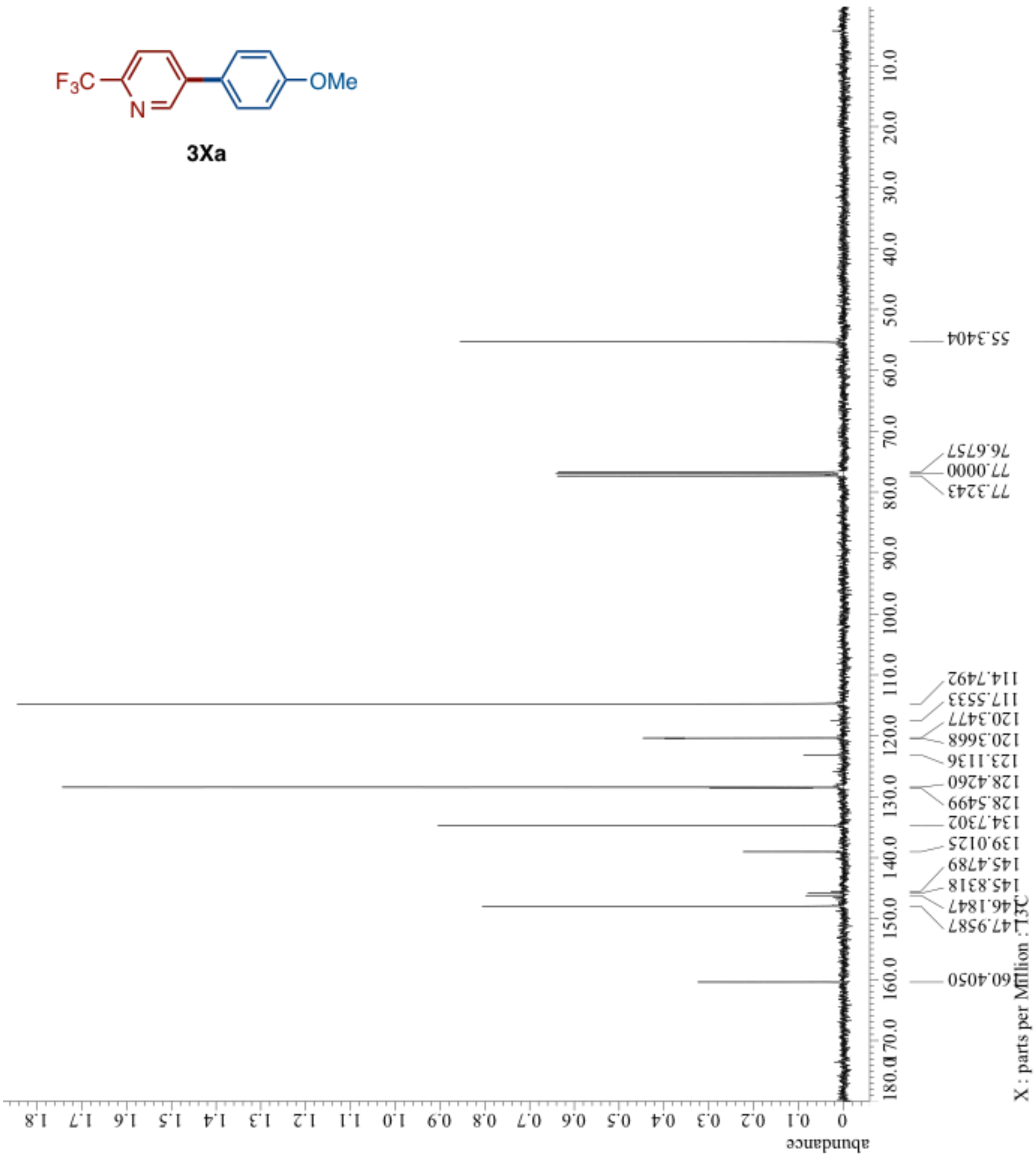


**Supplementary Figure 78.** $^{13}$**C NMR (100 MHz,** $CDCl_3$**) of 3Xa**

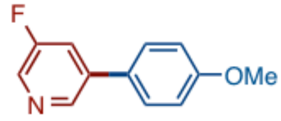


**3Ya**

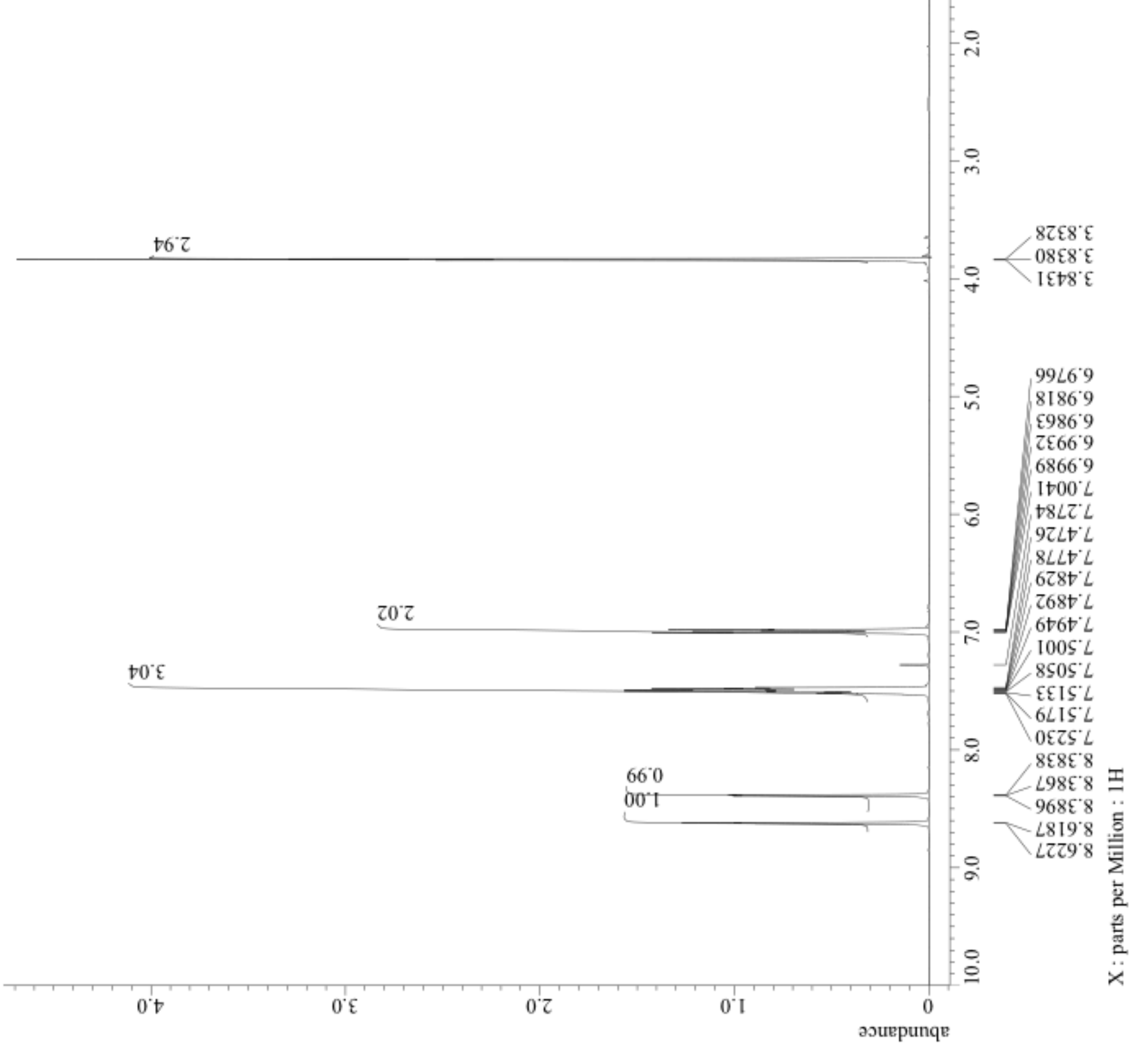


**Supplementary Figure 79.** $^{1}$**H NMR (400 MHz, CDCl$_3$) of 3Ya**

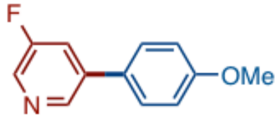


**3Ya**

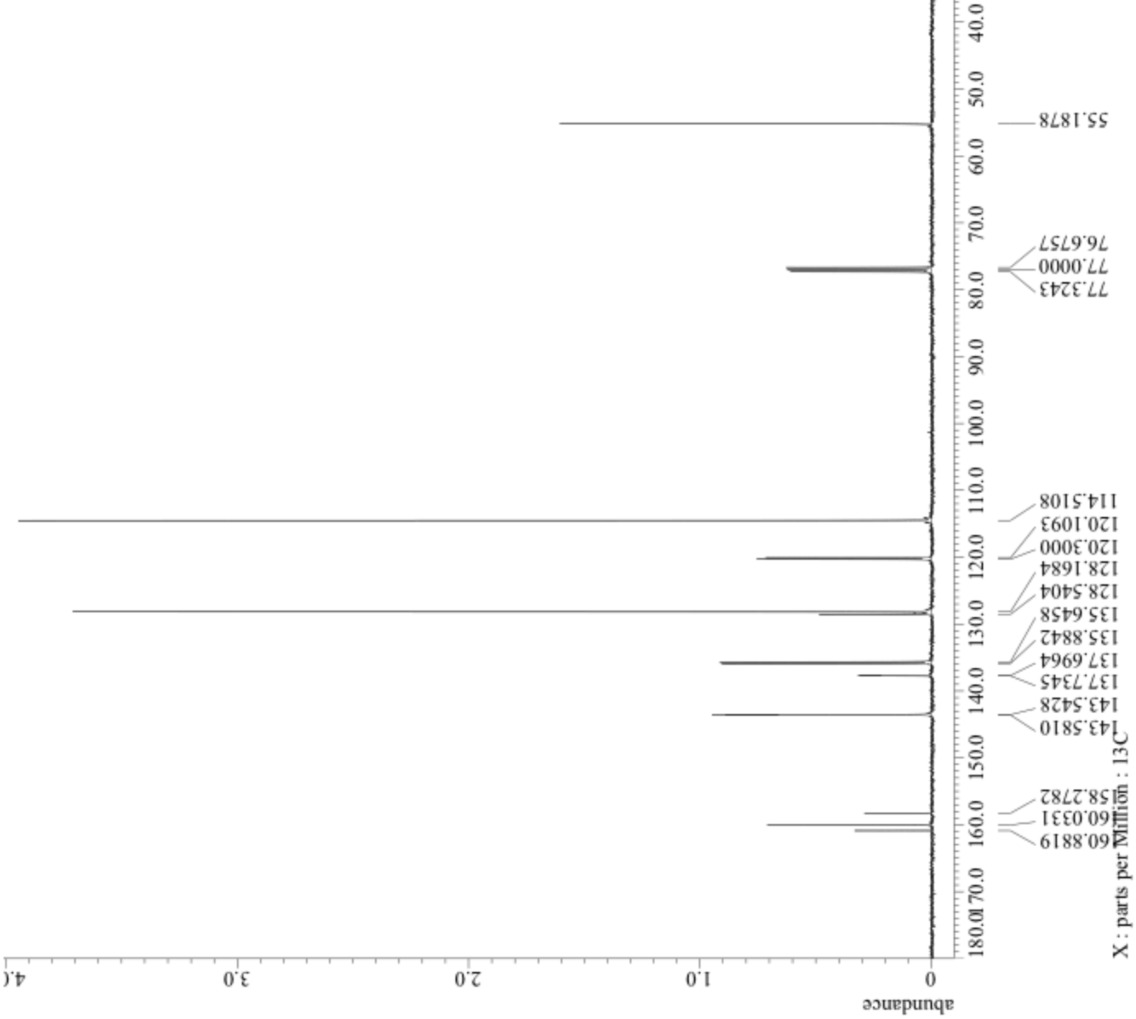


**Supplementary Figure 80.** $^{13}C$ **NMR (100 MHz,** $CDCl_3$**) of 3Ya**

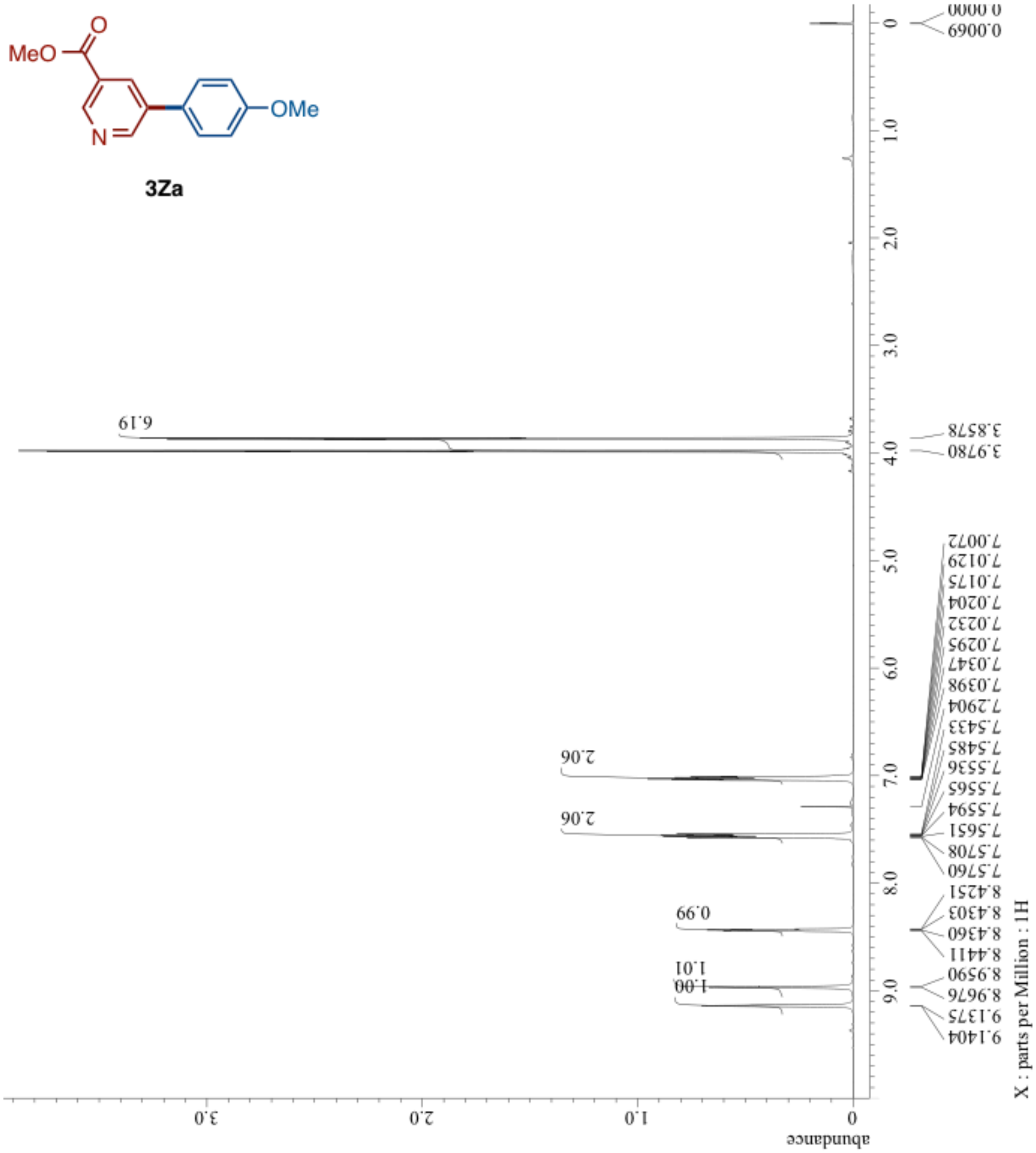


**Supplementary Figure 81.** $^{1}$H NMR (400 MHz, $CDCl_3$) of 3Za

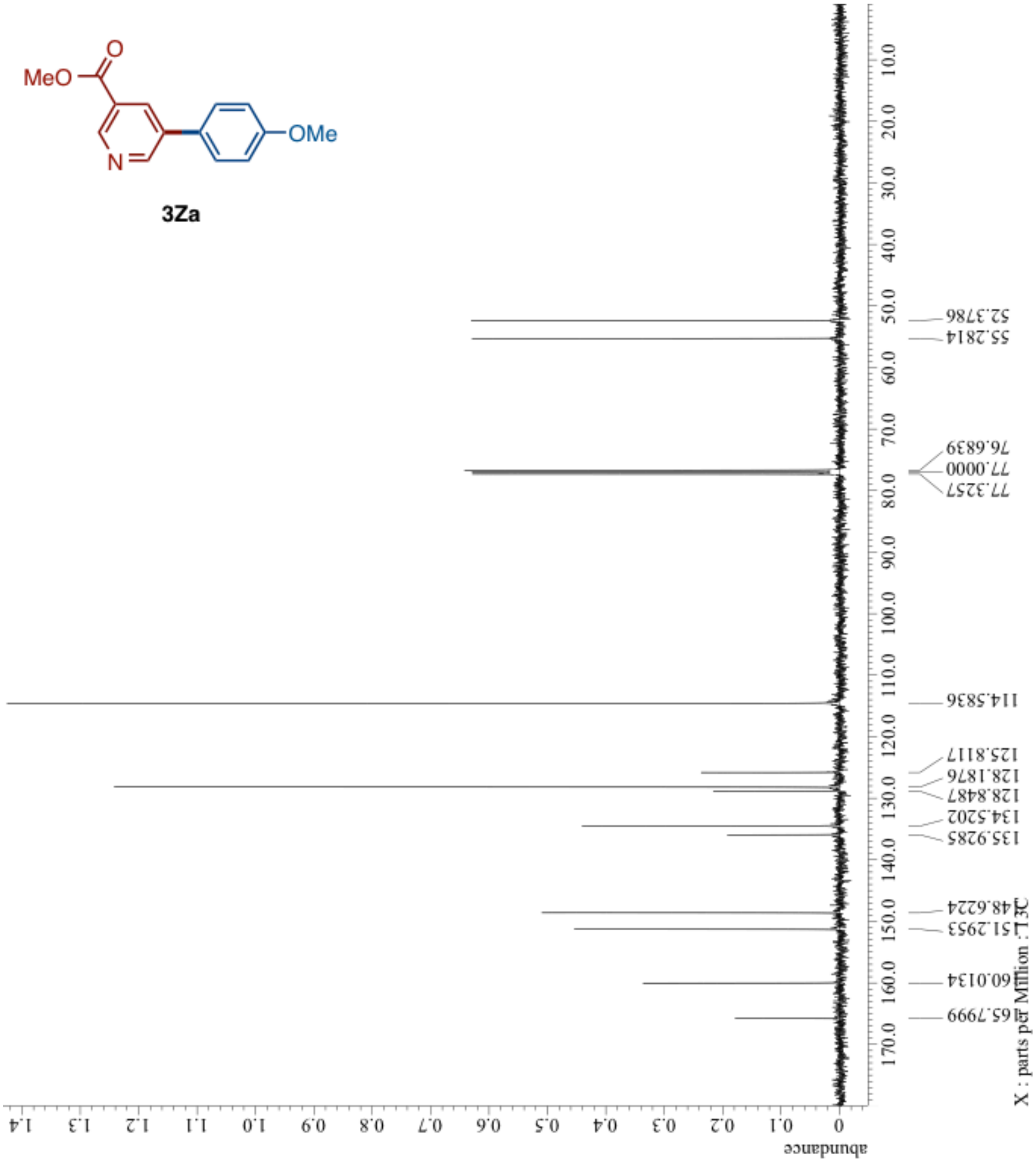


**Supplementary Figure 82.** $^{13}$**C NMR (100 MHz, CDCl$_3$) of 3Za**

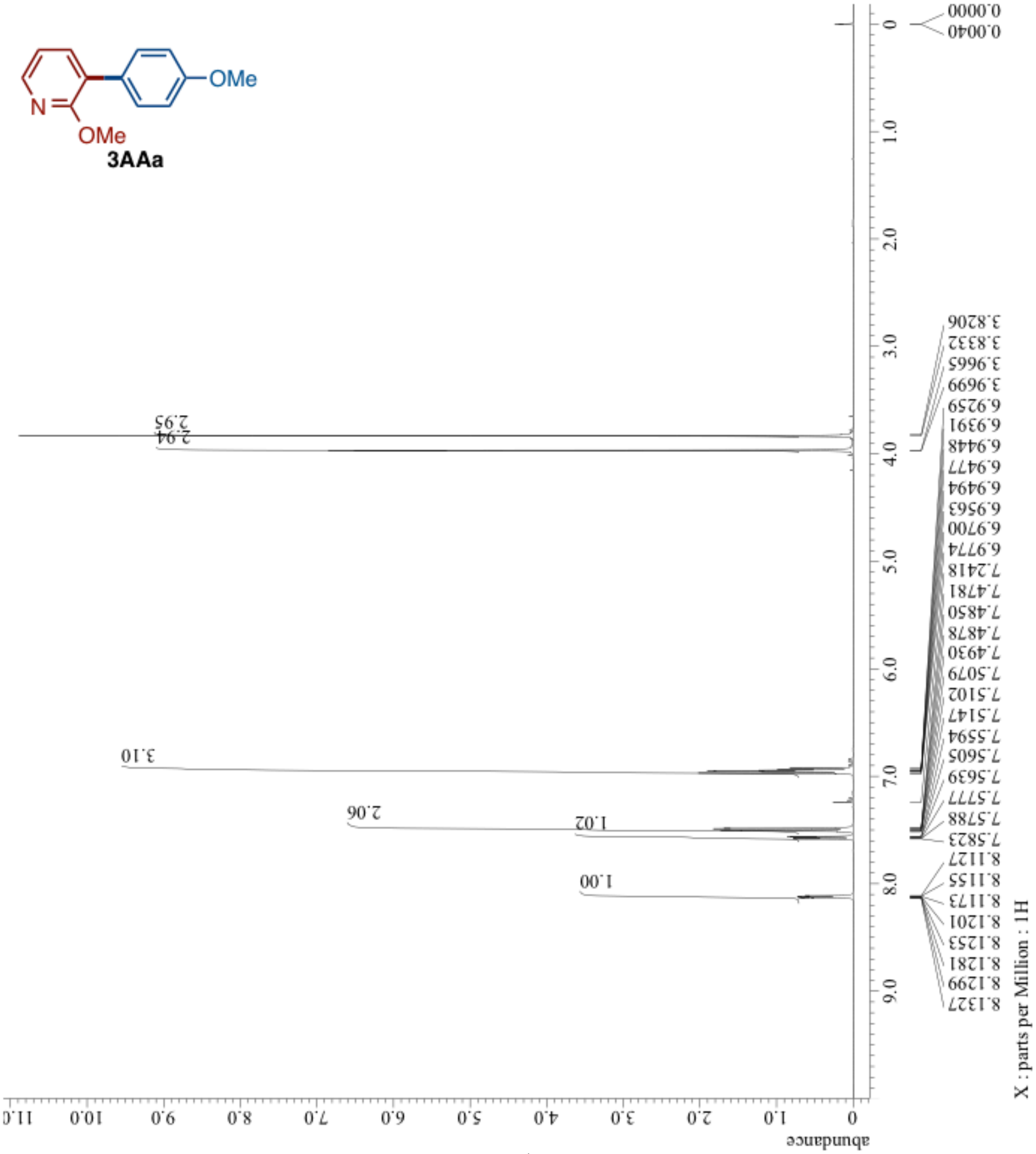


**Supplementary Figure 83.** $^1$**H NMR (400 MHz, $CDCl_3$) of 3AAa**

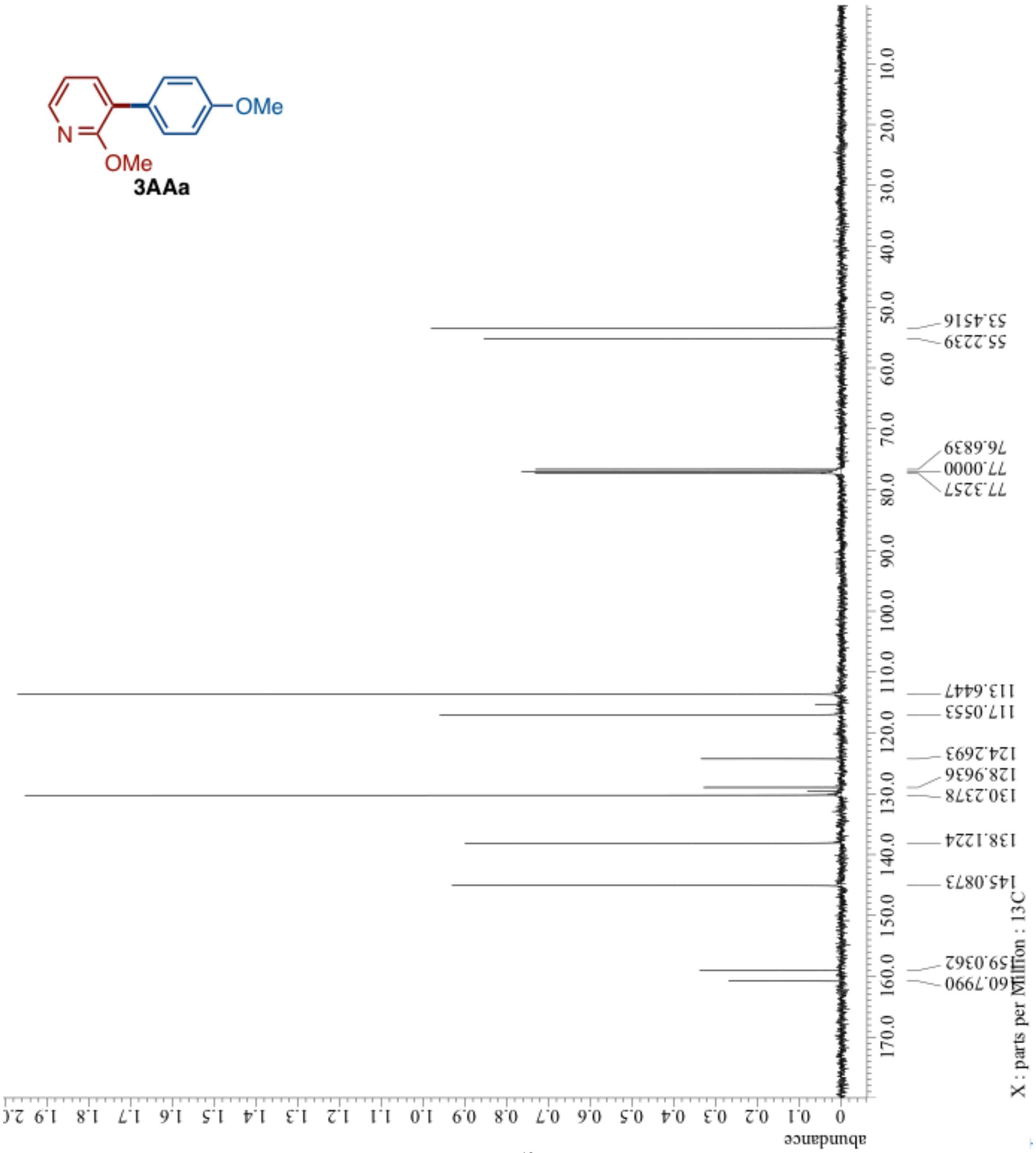


**Supplementary Figure 84.** $^{13}$**C NMR (100 MHz, $CDCl_3$) of 3AAa**

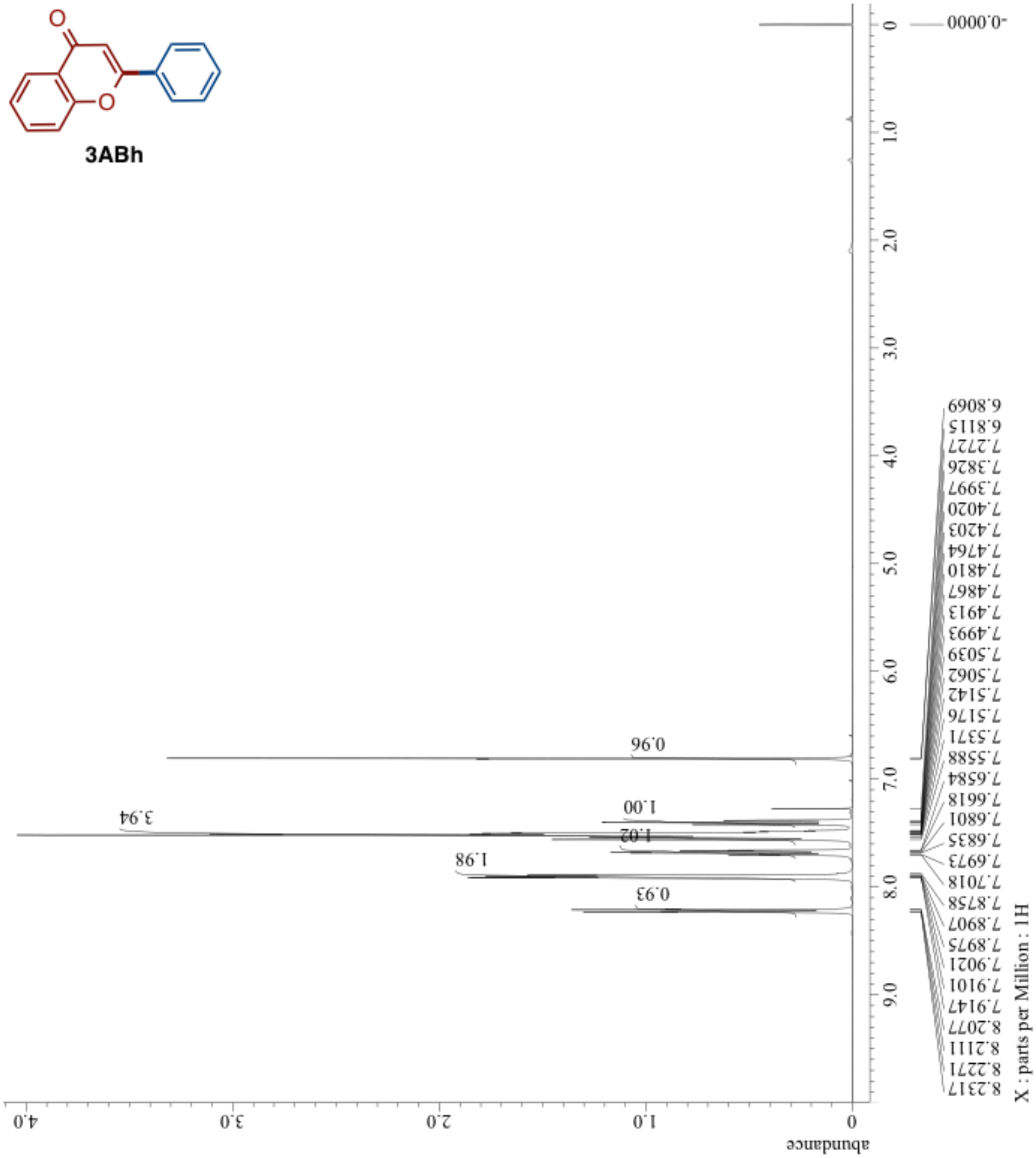

**3ABh**

**Supplementary Figure 85.** $^{1}H$ **NMR (400 MHz,** $CDCl_3$**) of 3ABh**

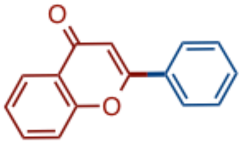

**3ABh**

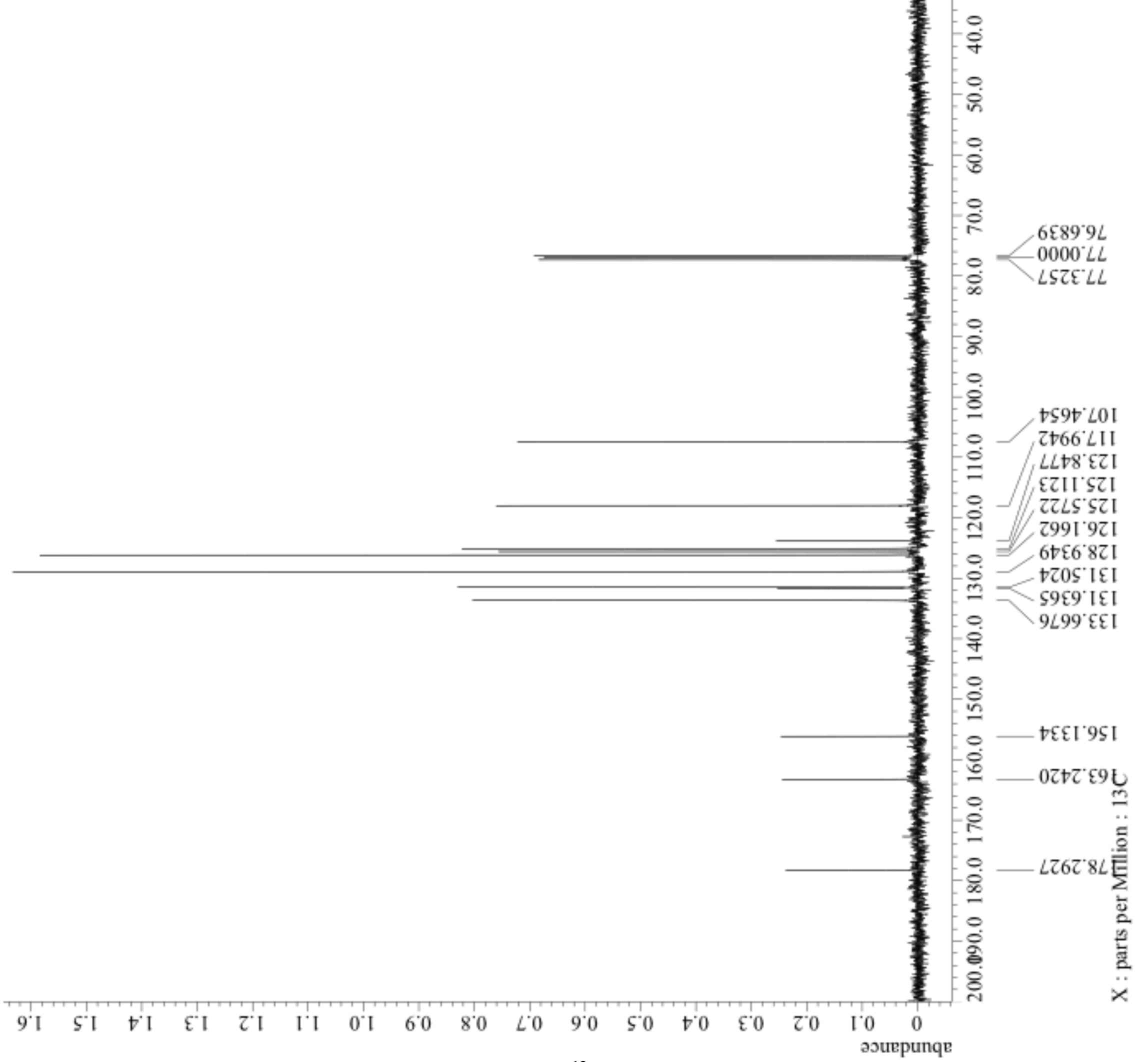


**Supplementary Figure 86.** $^{13}$**C NMR (100 MHz,** $CDCl_3$**) of 3ABh**

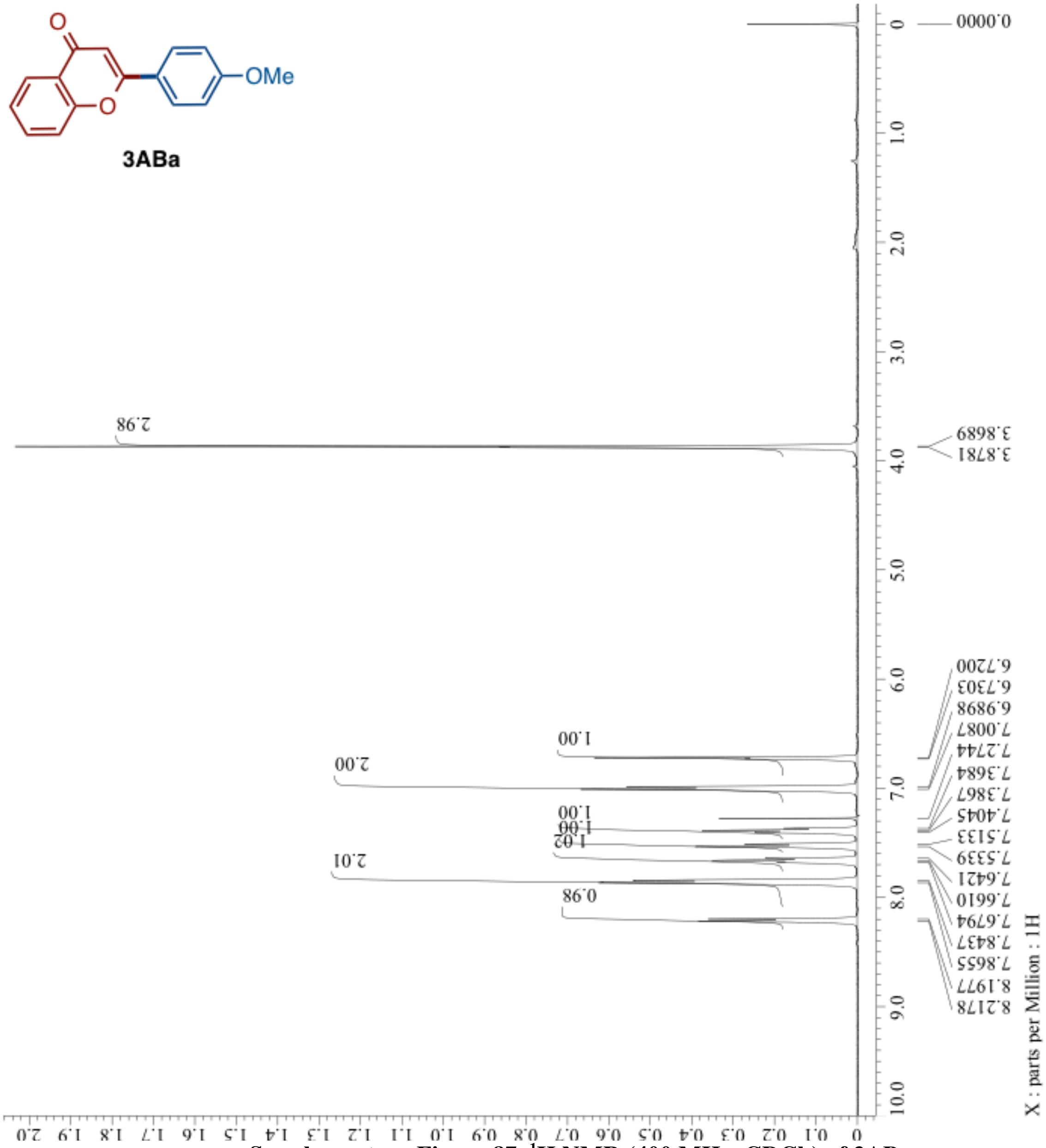


**Supplementary Figure 87. $^{1}H$ NMR (400 MHz, $CDCl_3$) of 3ABa**

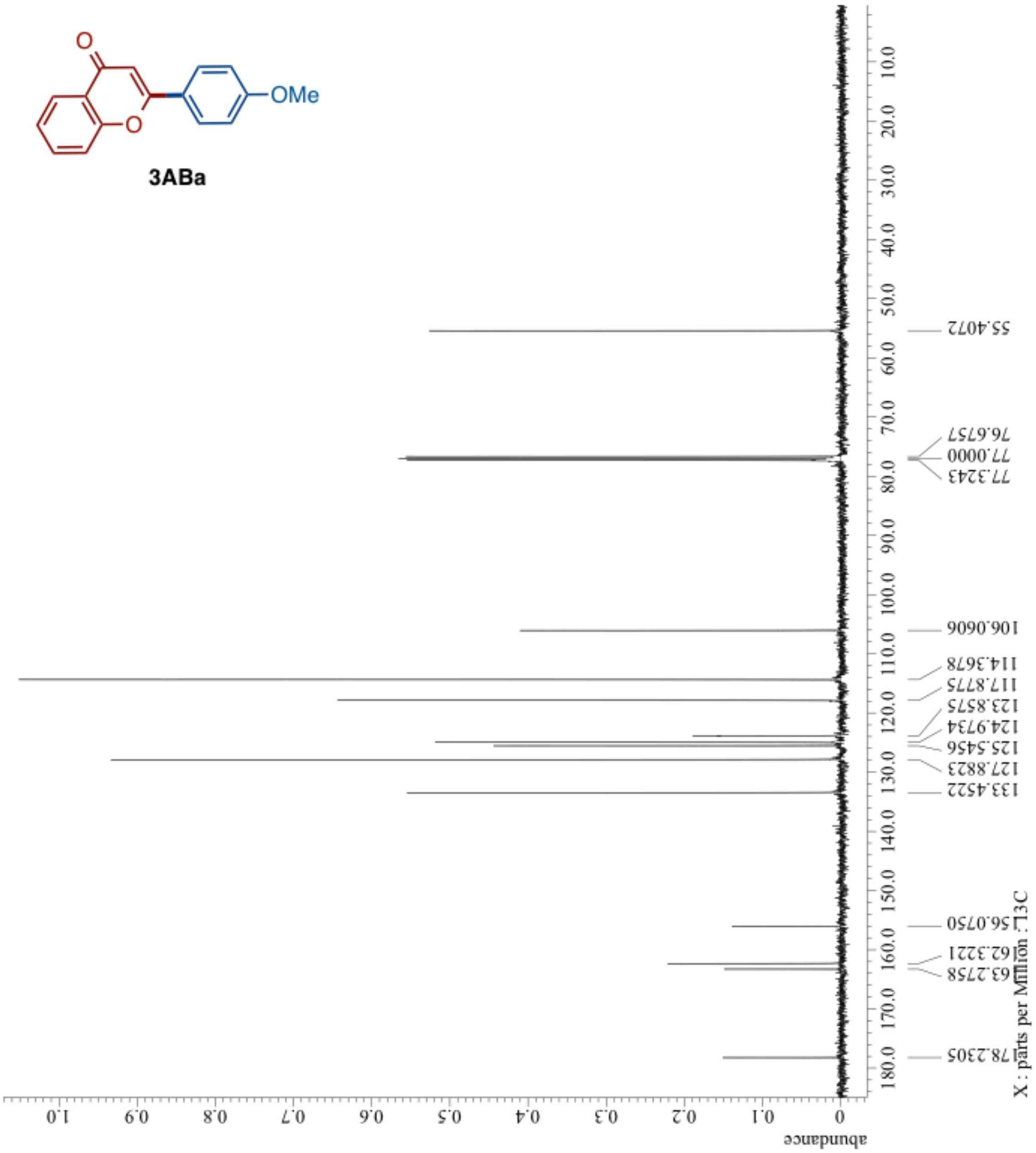


**Supplementary Figure 88.** $^{13}$**C NMR (100 MHz, CDCl$_3$) of 3ABa**

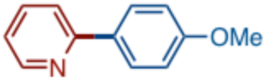


**3ACa**

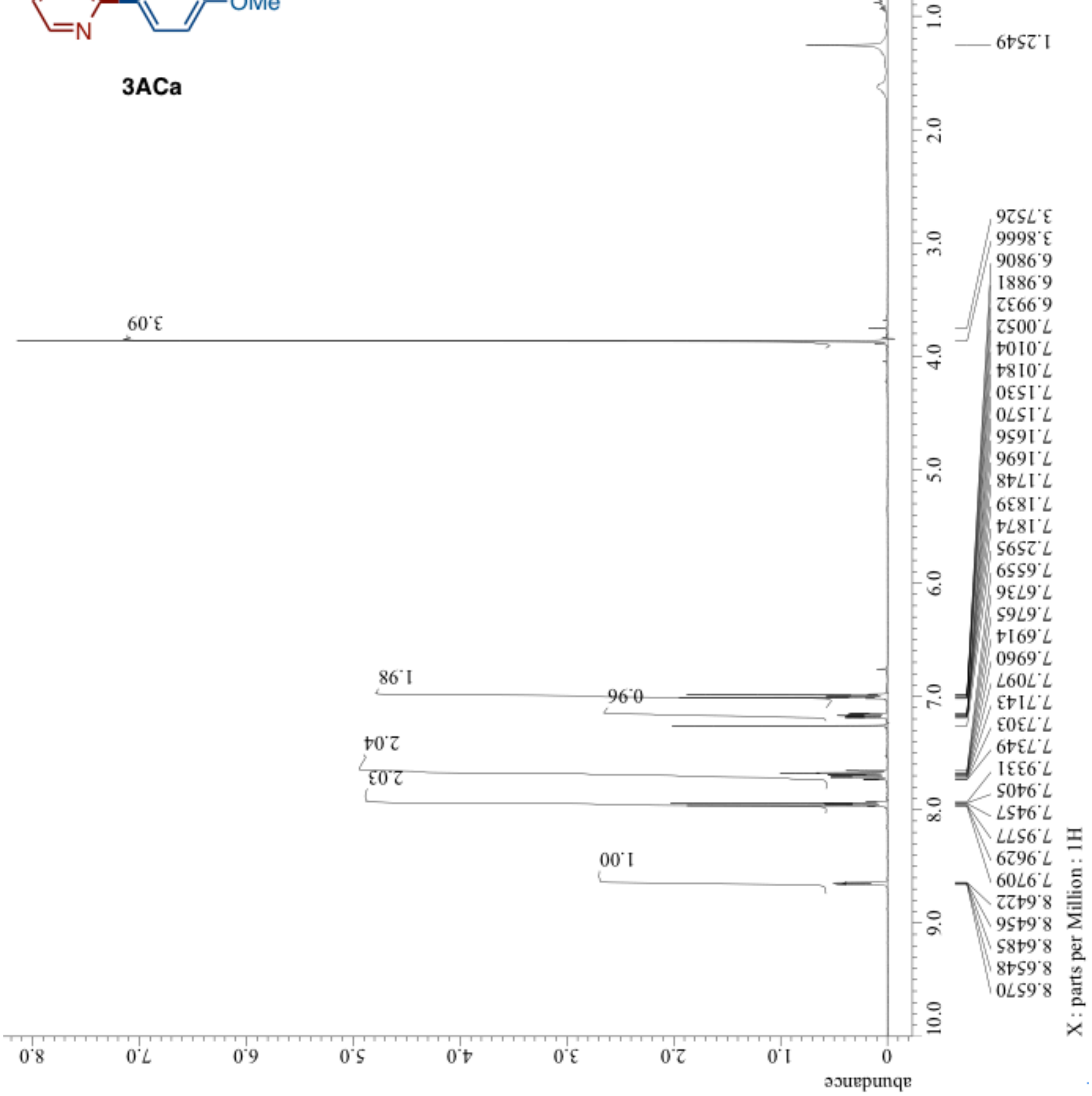


**Supplementary Figure 89. $^1$H NMR (400 MHz, $CDCl_3$) of 3ACa**

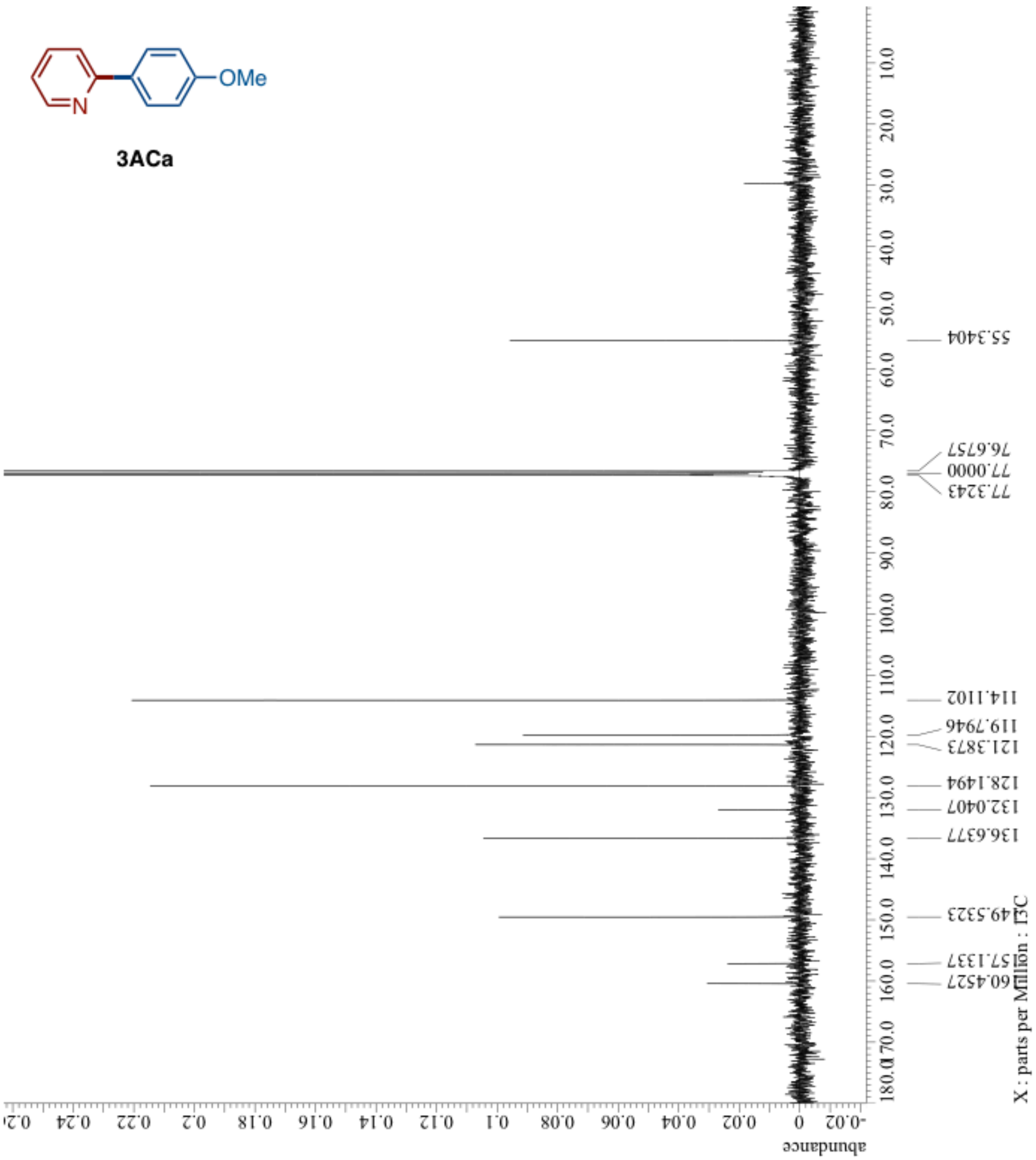


**Supplementary Figure 90.** $^{13}$**C NMR (100 MHz,** $CDCl_3$**) of 3ACa**

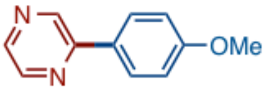


**3ADa**

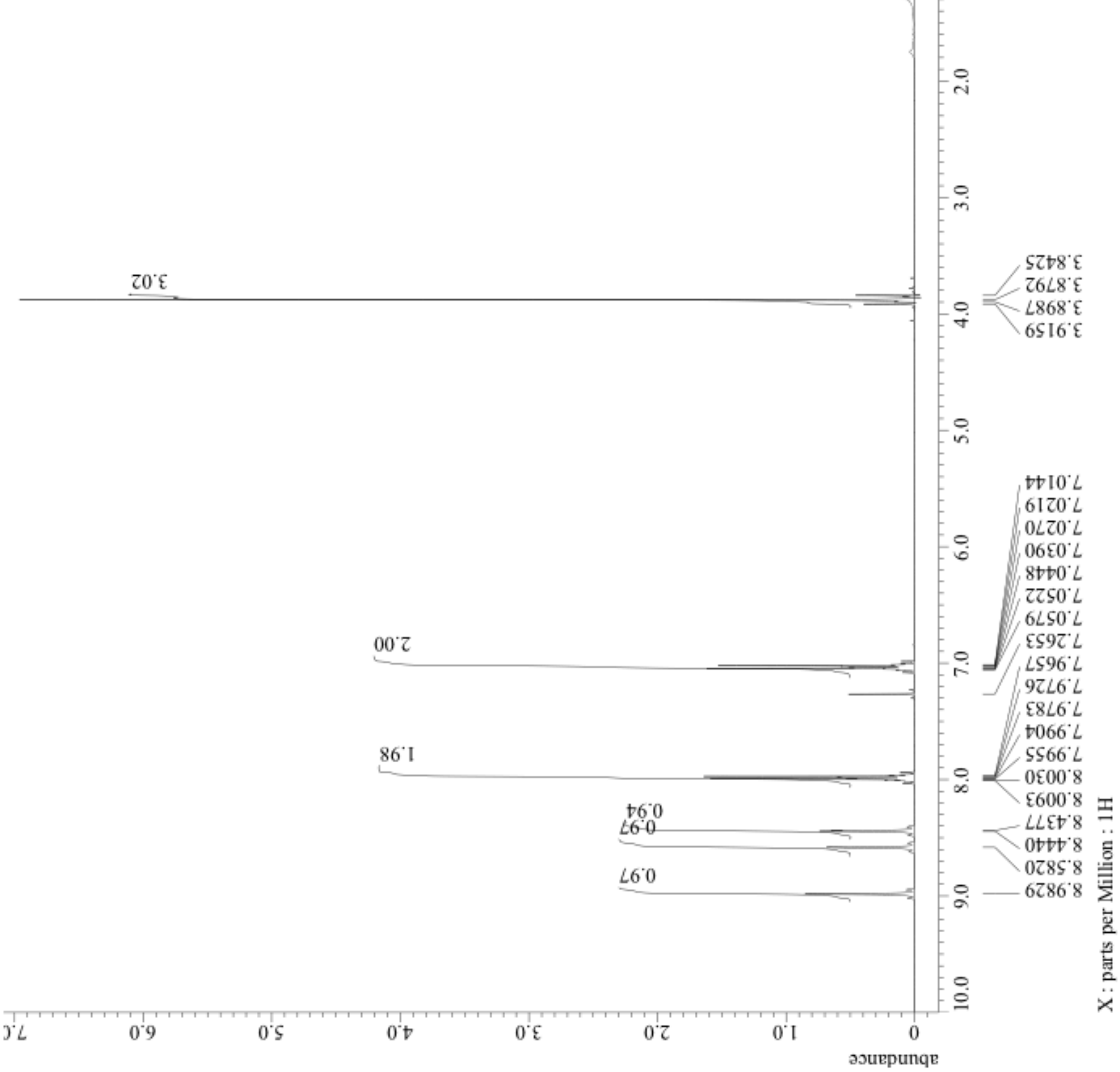

**Supplementary Figure 91. $^1$H NMR (400 MHz, $CDCl_3$) of 3ADa**

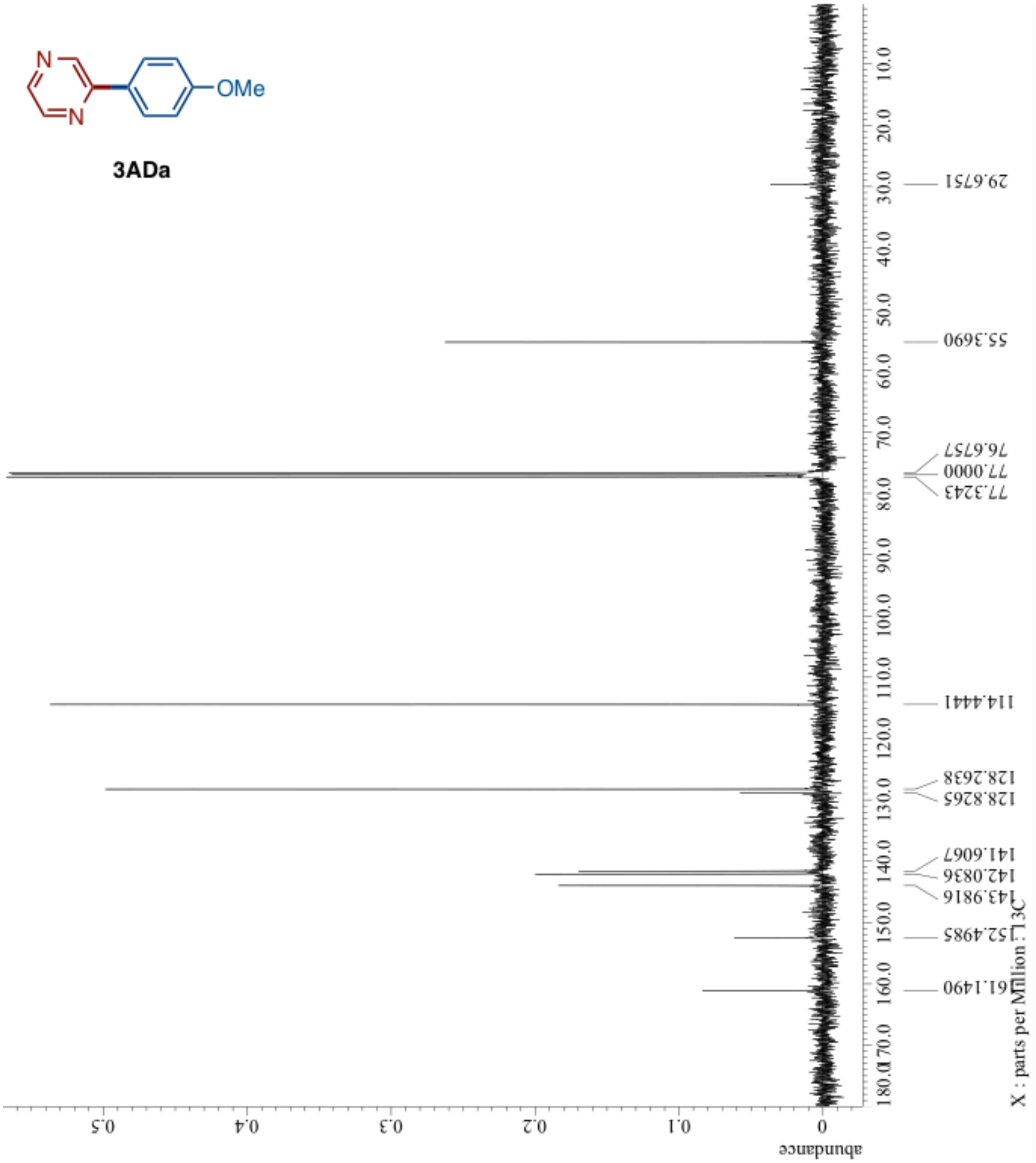


**Supplementary Figure 92.** $^{13}C$ **NMR (100 MHz,** $CDCl_3$**) of 3ADa**

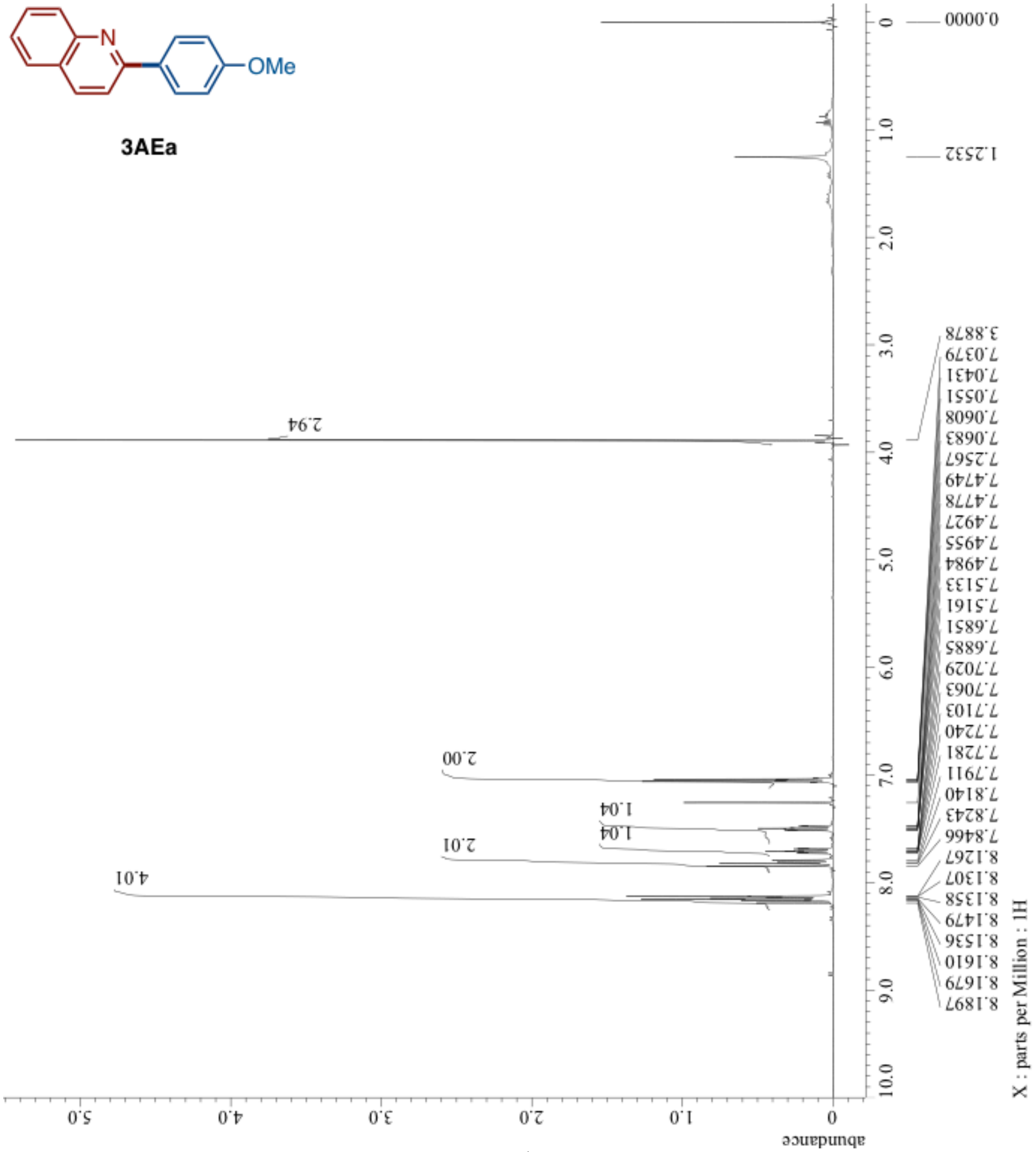


**Supplementary Figure 93.** **$^{1}$H NMR (400 MHz, $CDCl_3$) of 3AEa**

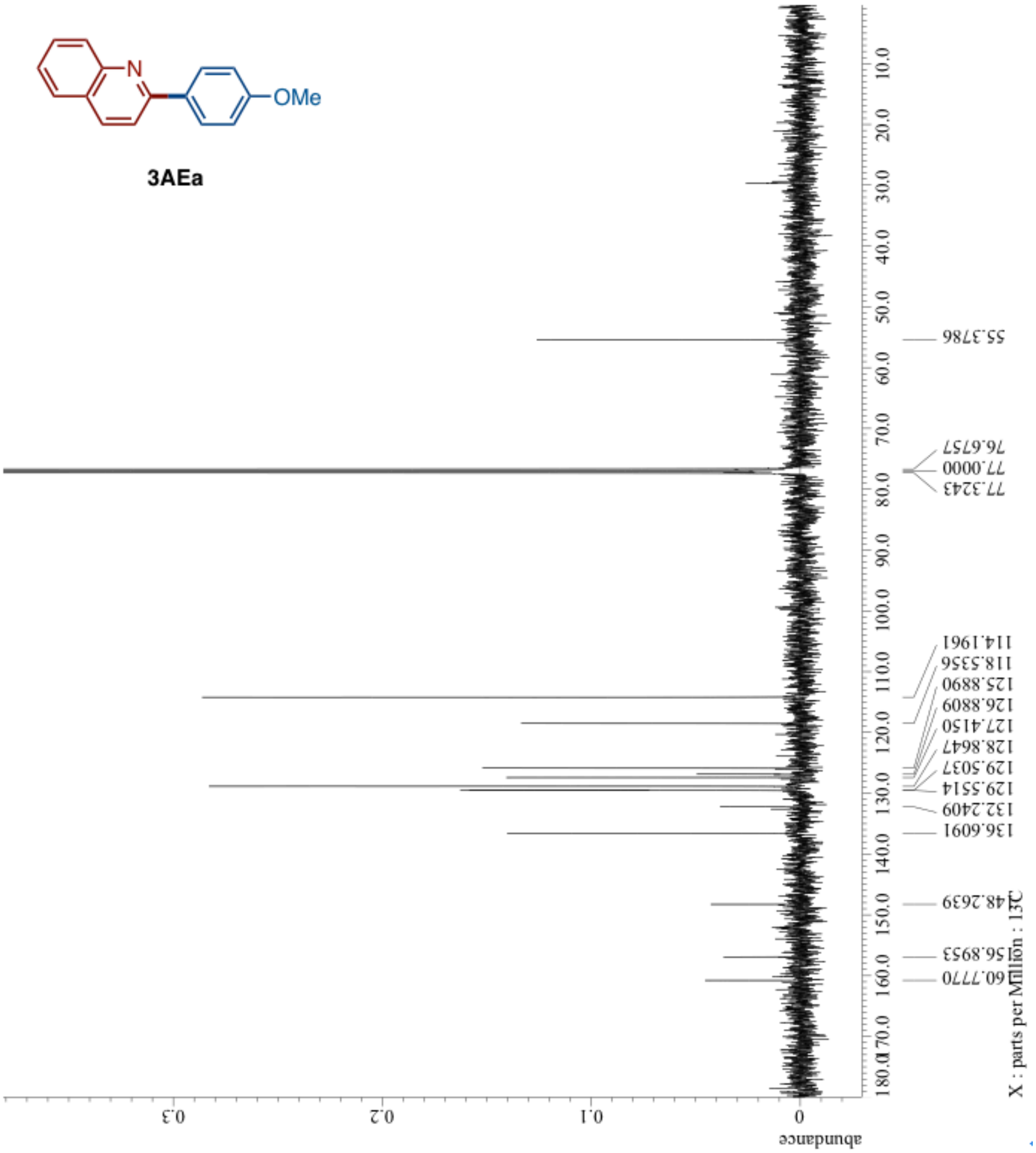


**Supplementary Figure 94. $^{13}$C NMR (100 MHz, $CDCl_3$) of 3AEa**

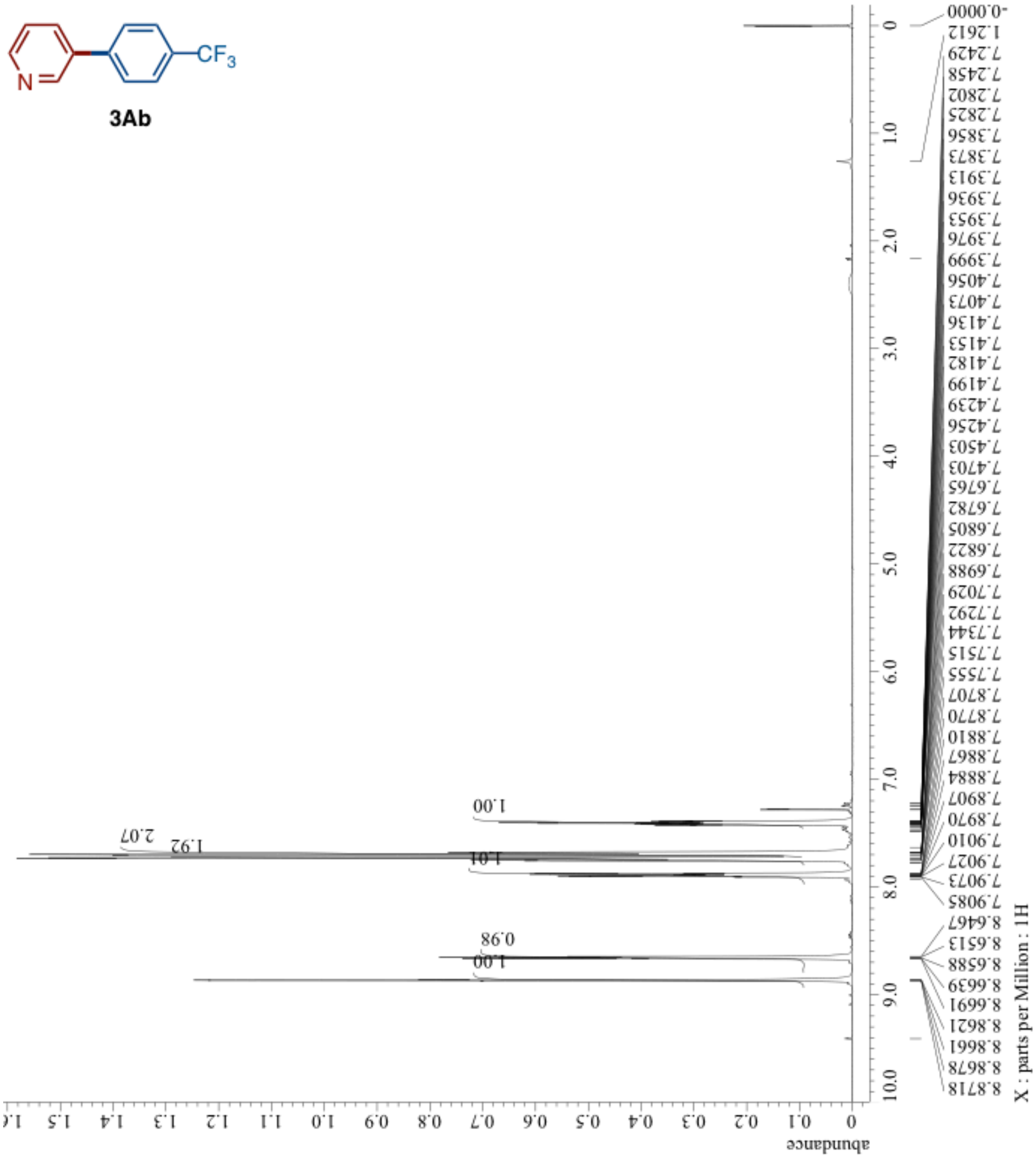


**Supplementary Figure 95.** $^{1}$**H NMR (400 MHz, CDCl$_3$) of 3Ab**

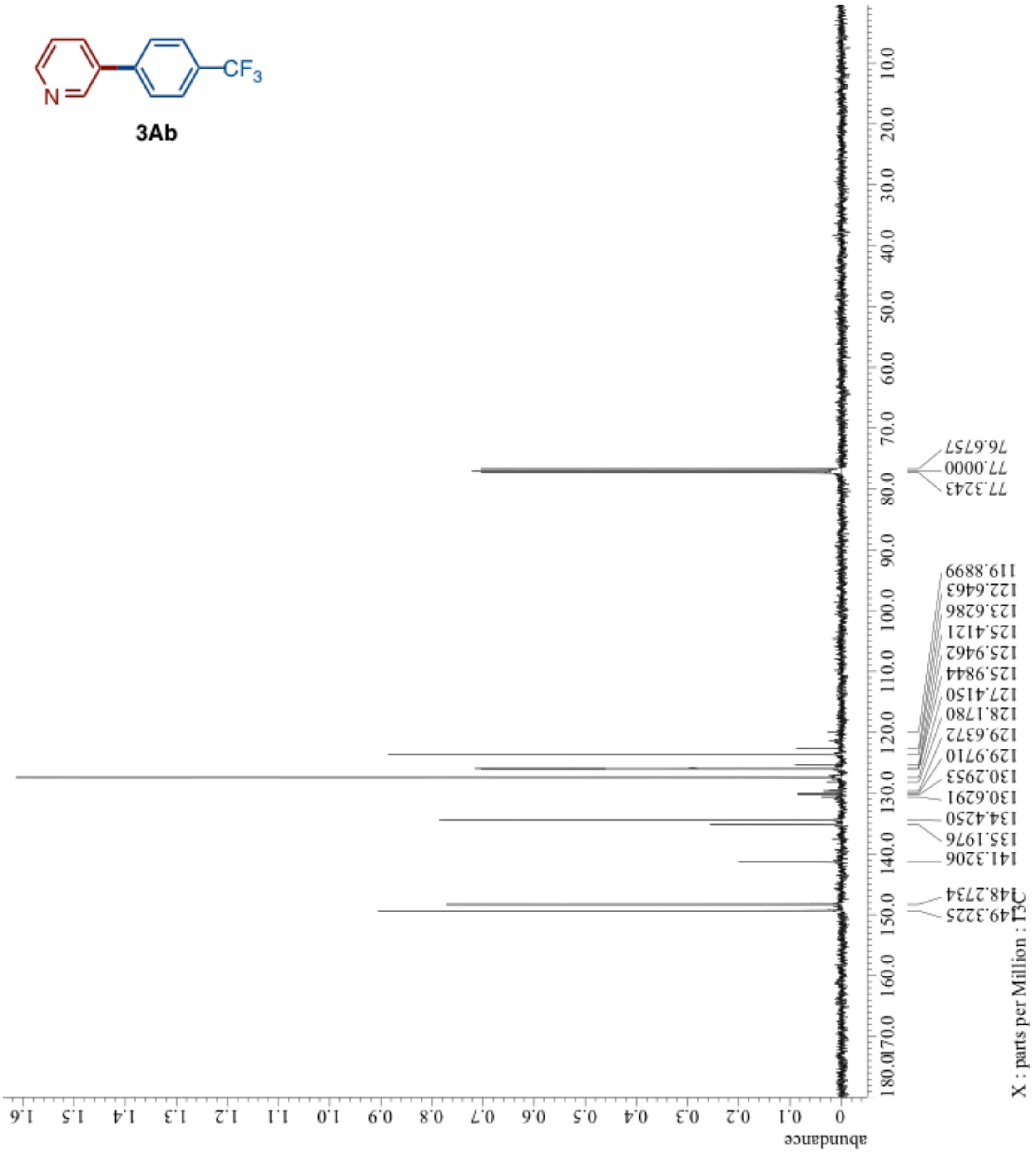


**Supplementary Figure 96. $^{13}C$ NMR (100 MHz, $CDCl_3$) of 3Ab**

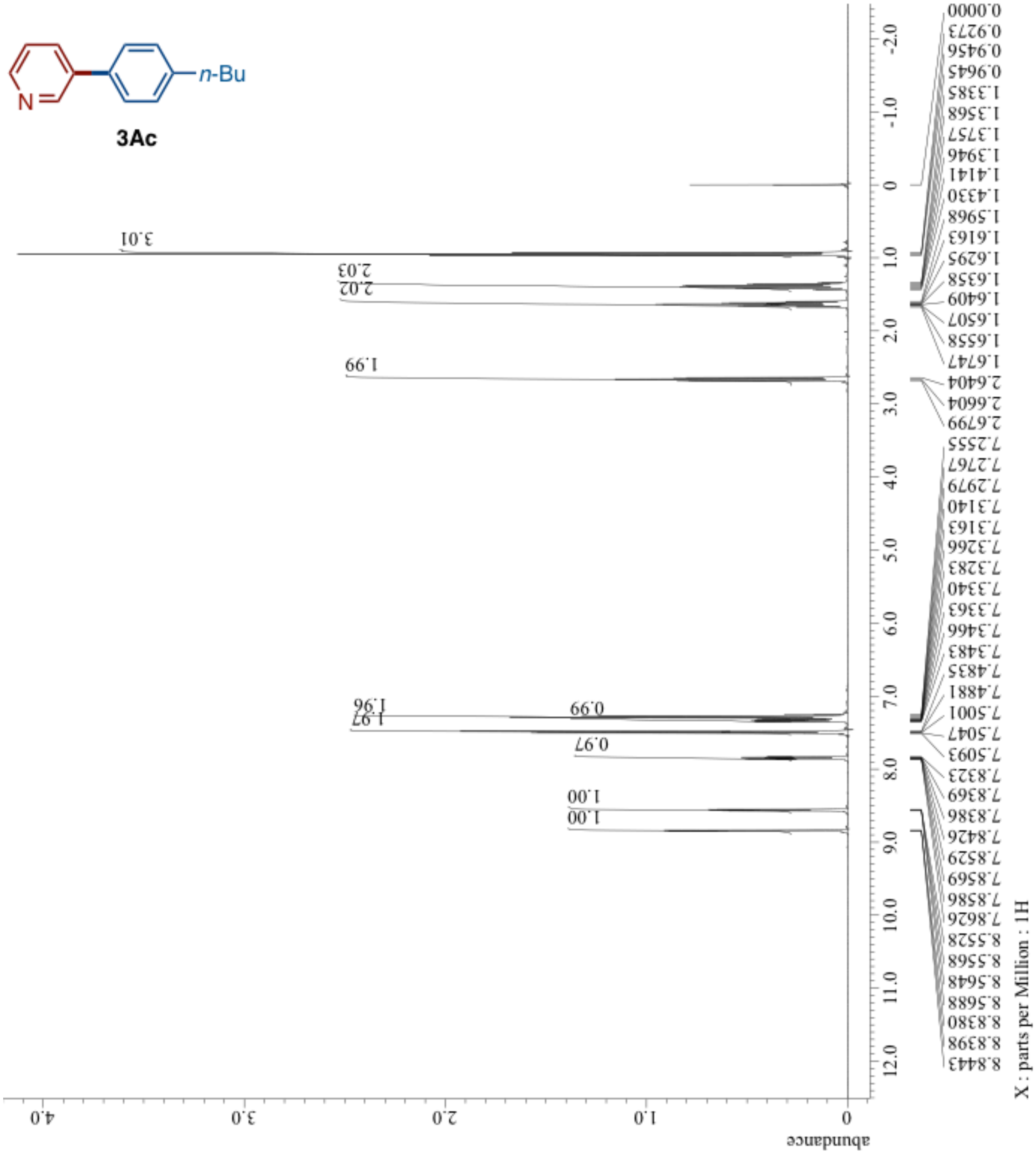


**Supplementary Figure 97.** $^{1}$**H NMR (400 MHz,** $CDCl_3$**) of 3Ac**

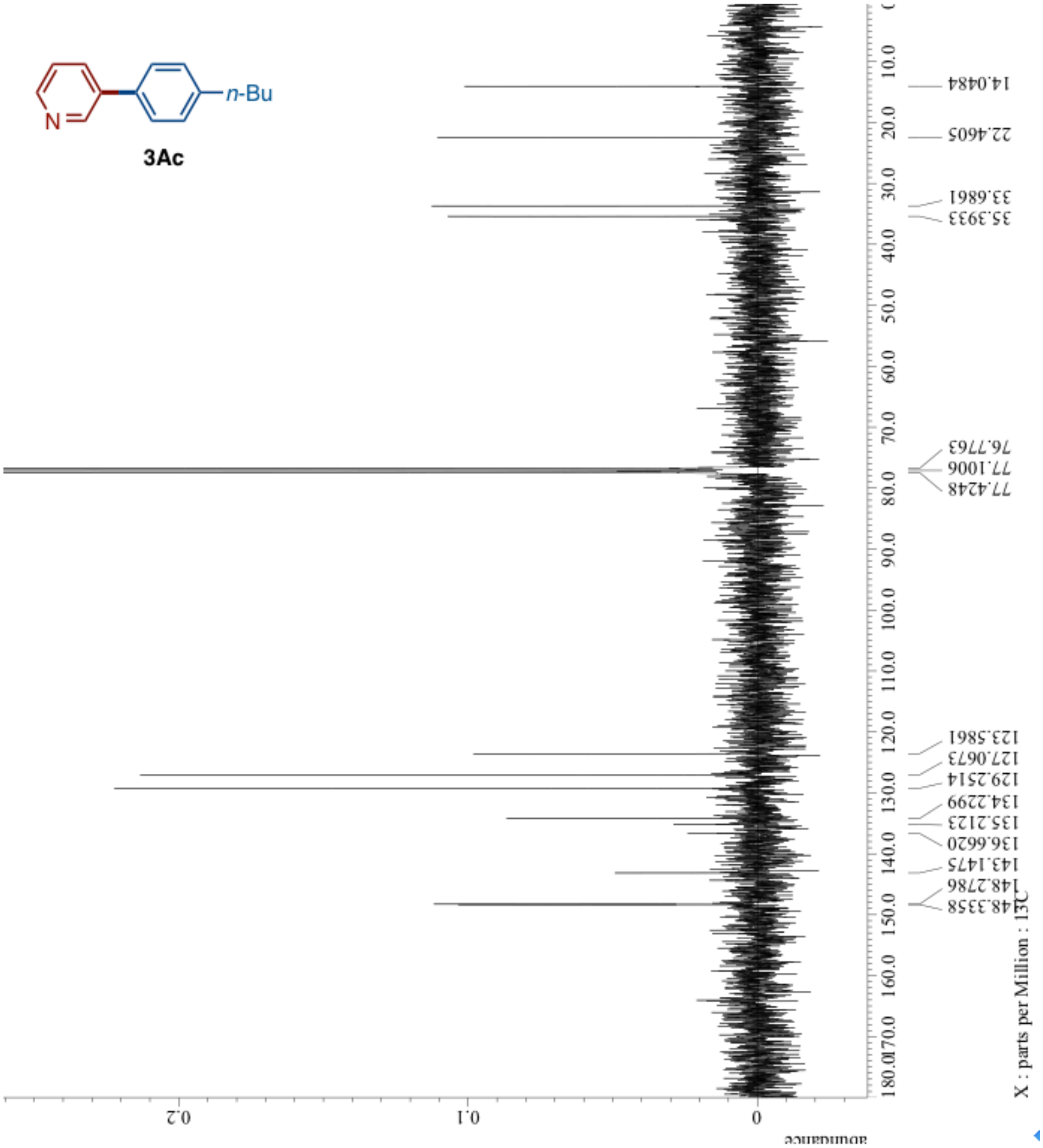


Supplementary Figure 98. $^{13}C$ NMR (100 MHz, $CDCl_3$) of 3Ac

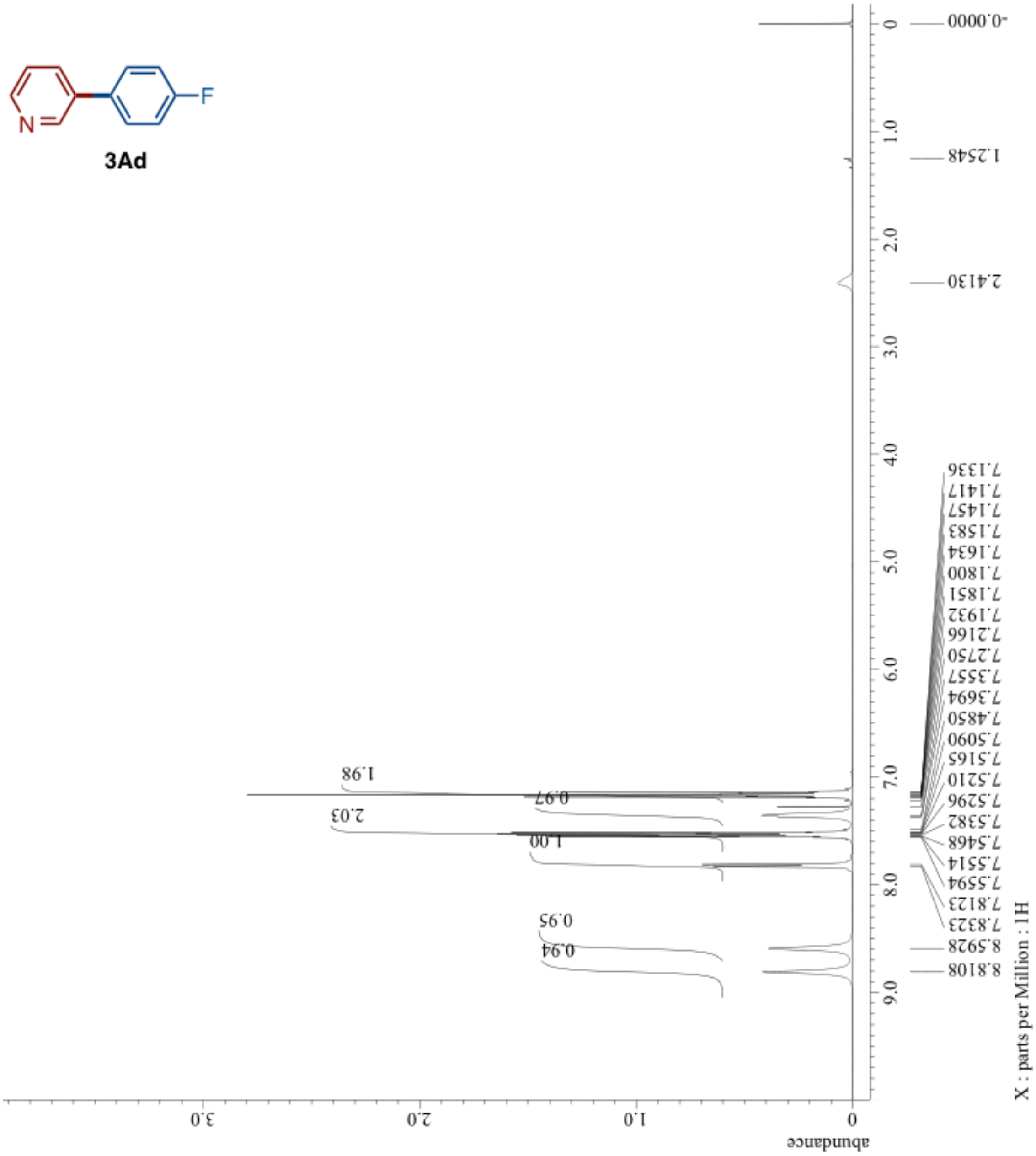


**Supplementary Figure 99. $^{1}$H NMR (400 MHz, $CDCl_3$) of 3Ad**

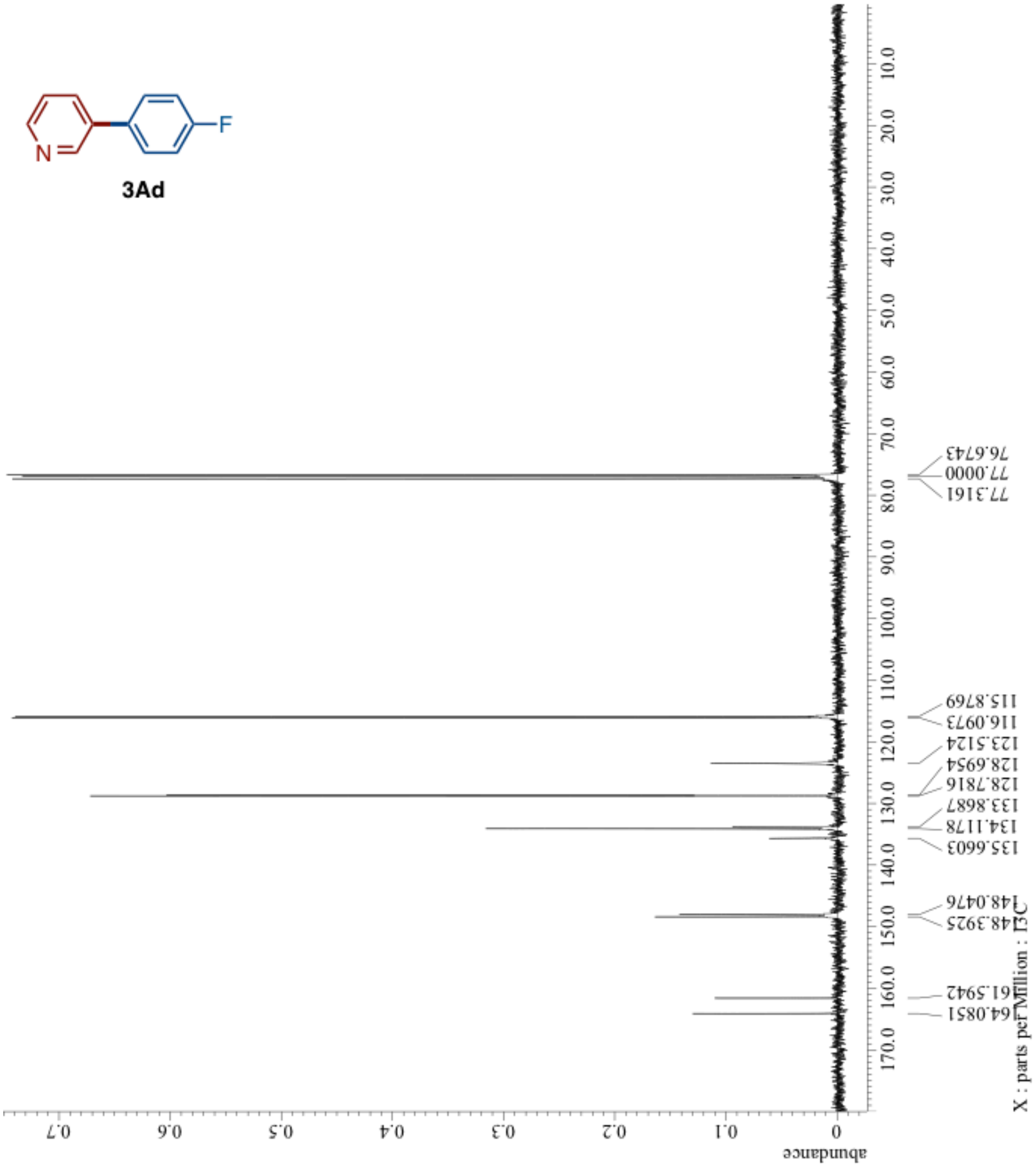


**Supplementary Figure 100.** $^{13}$**C NMR (100 MHz,** $CDCl_3$**) of 3Ad**

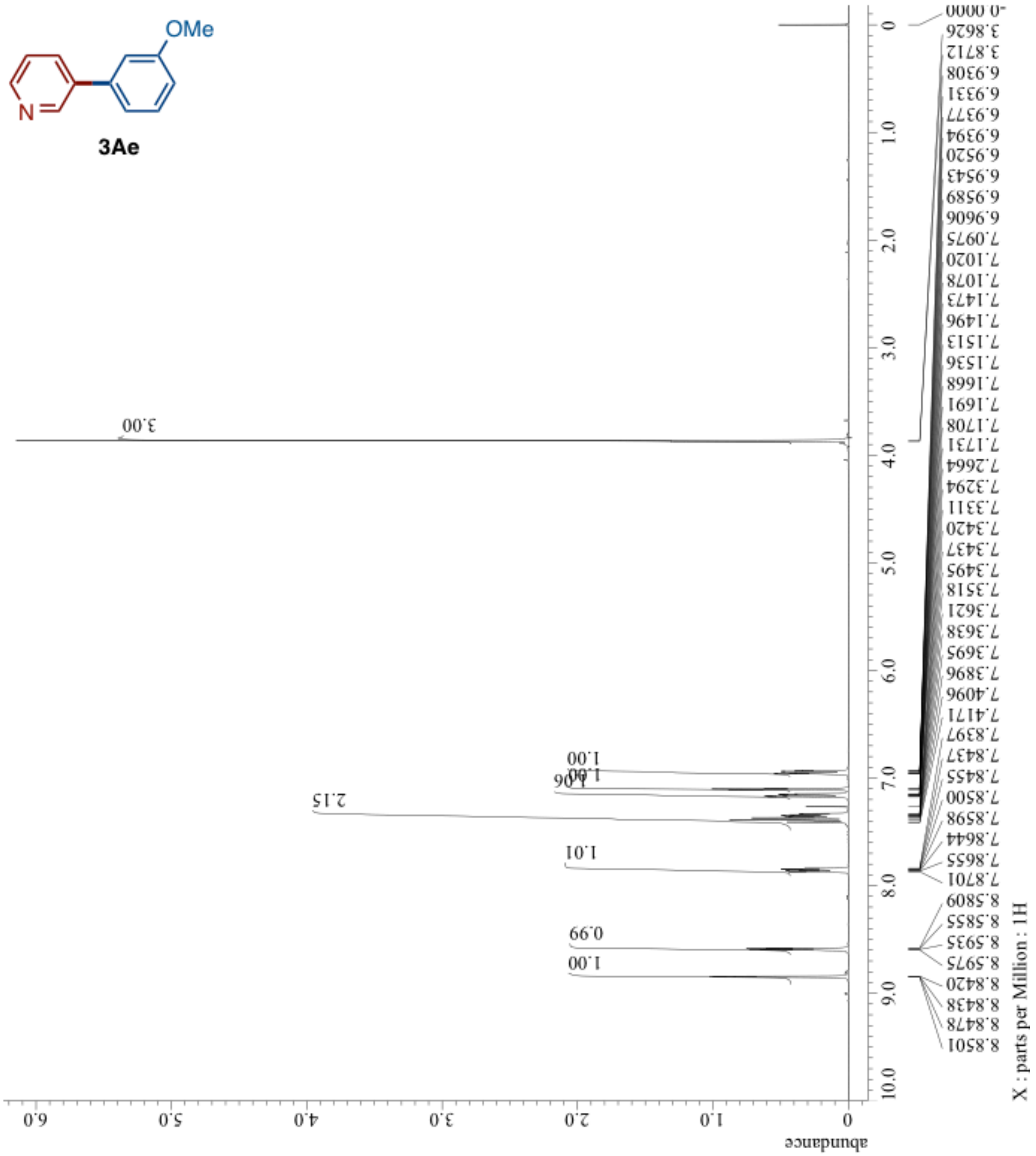


**Supplementary Figure 101.** **$^{1}$H NMR (400 MHz, $CDCl_3$) of 3Ae**

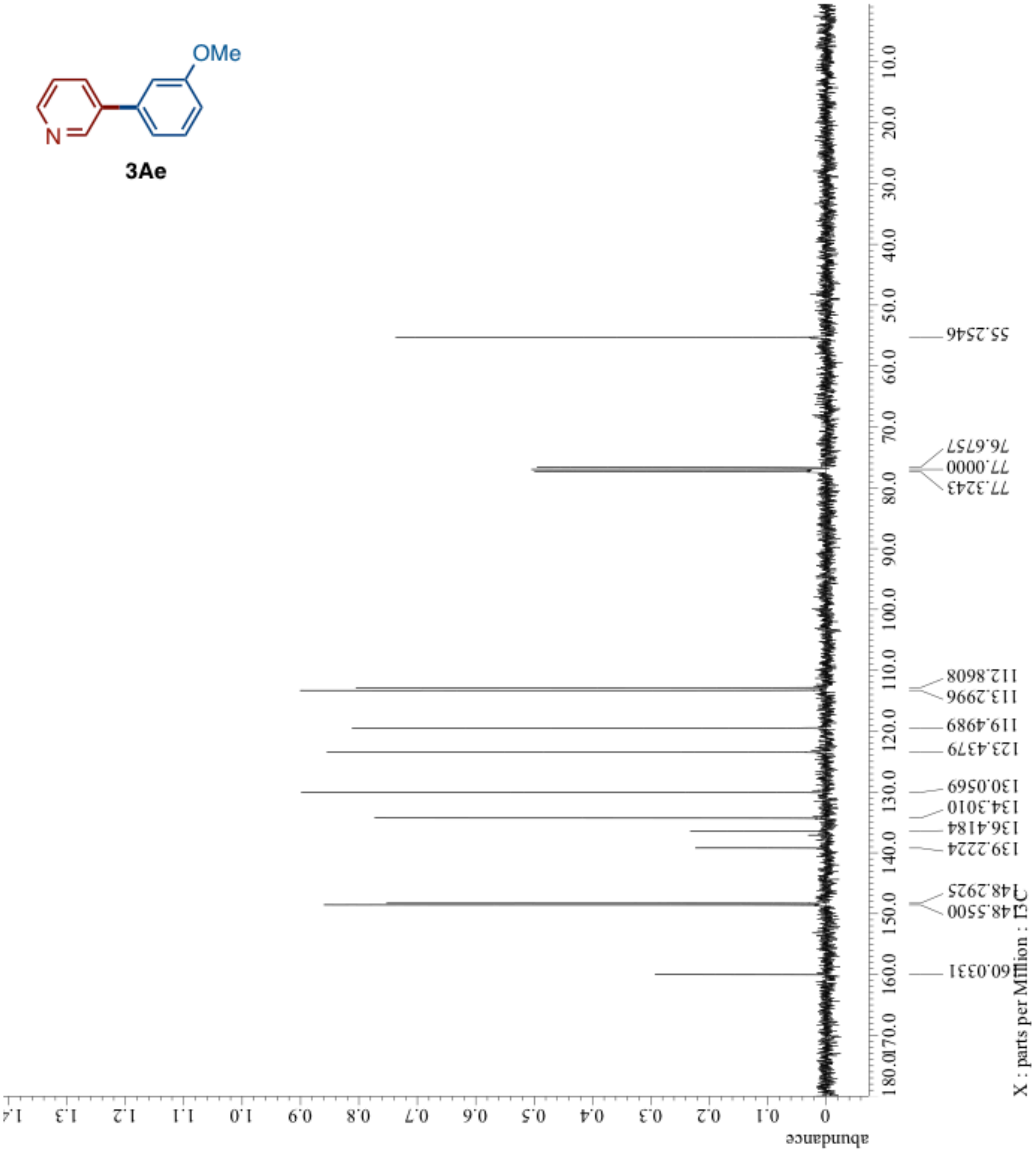


**Supplementary Figure 102.** **$^{13}C$ NMR (100 MHz, $CDCl_3$) of 3Ae**

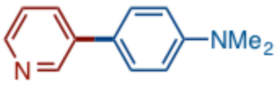


**3Af**

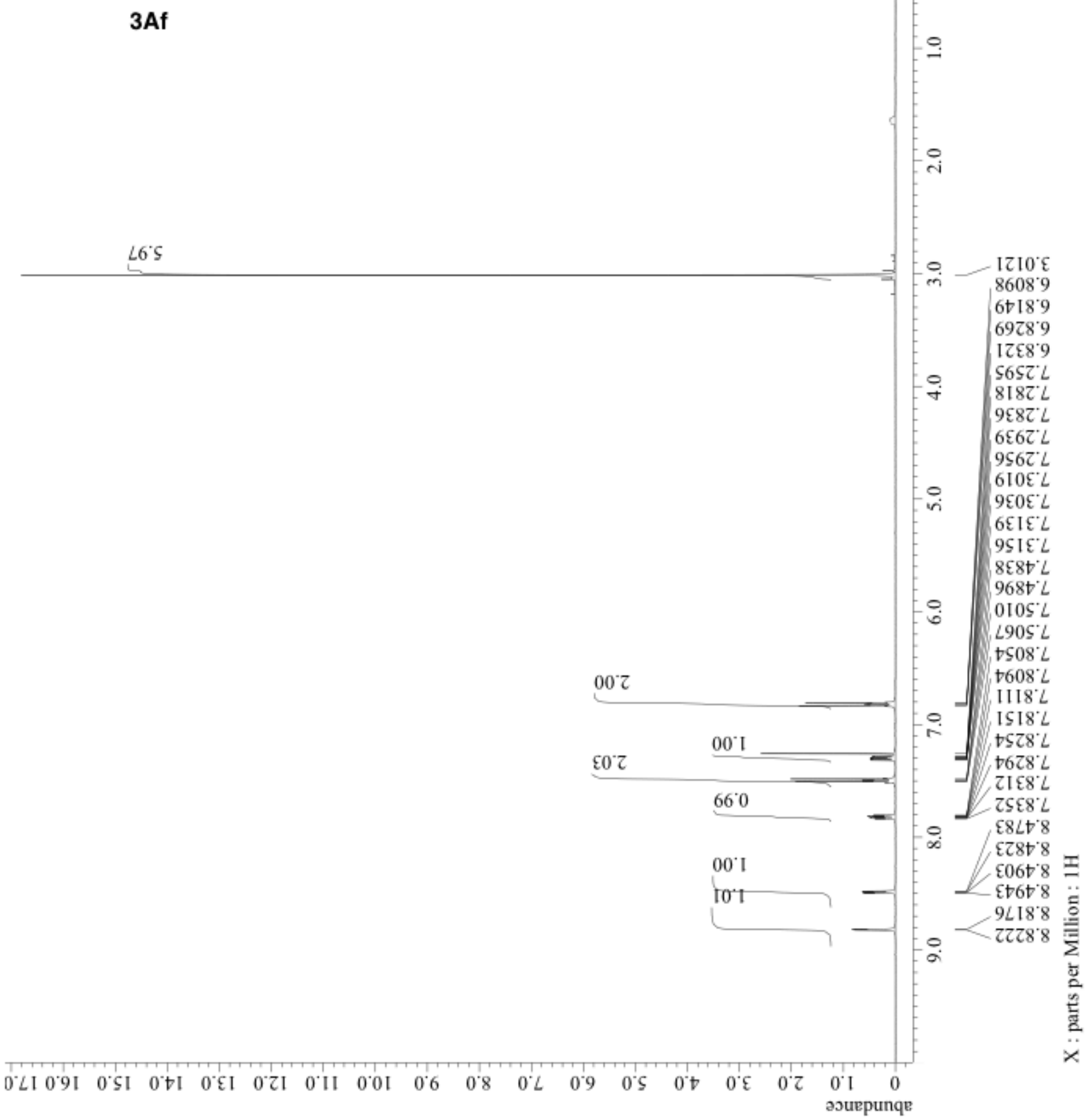


**Supplementary Figure 103.** $^{1}$**H NMR (400 MHz,** $CDCl_3$**) of 3Af**

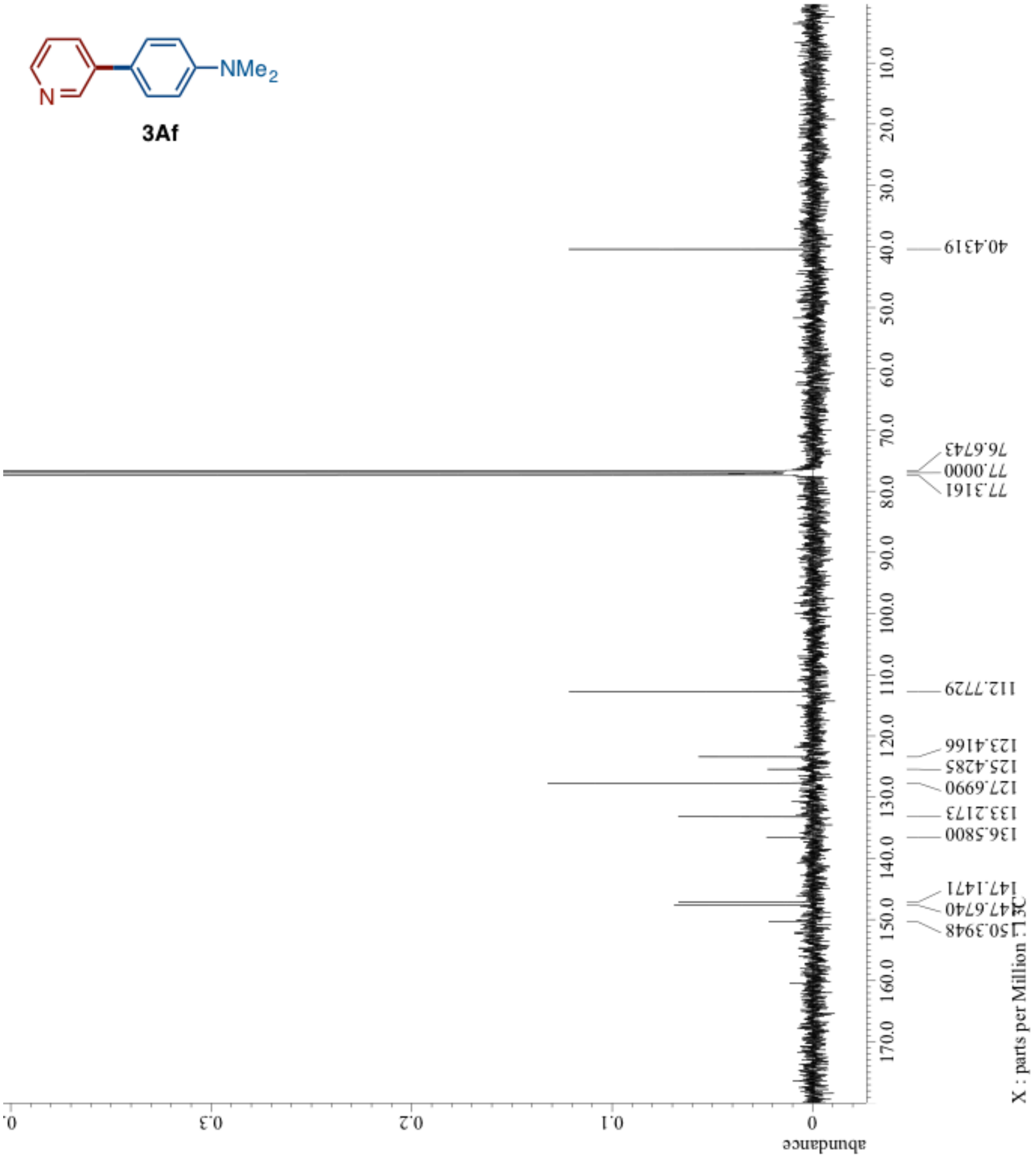


**Supplementary Figure 104. $^{13}C$ NMR (100 MHz, $CDCl_3$) of 3Af**

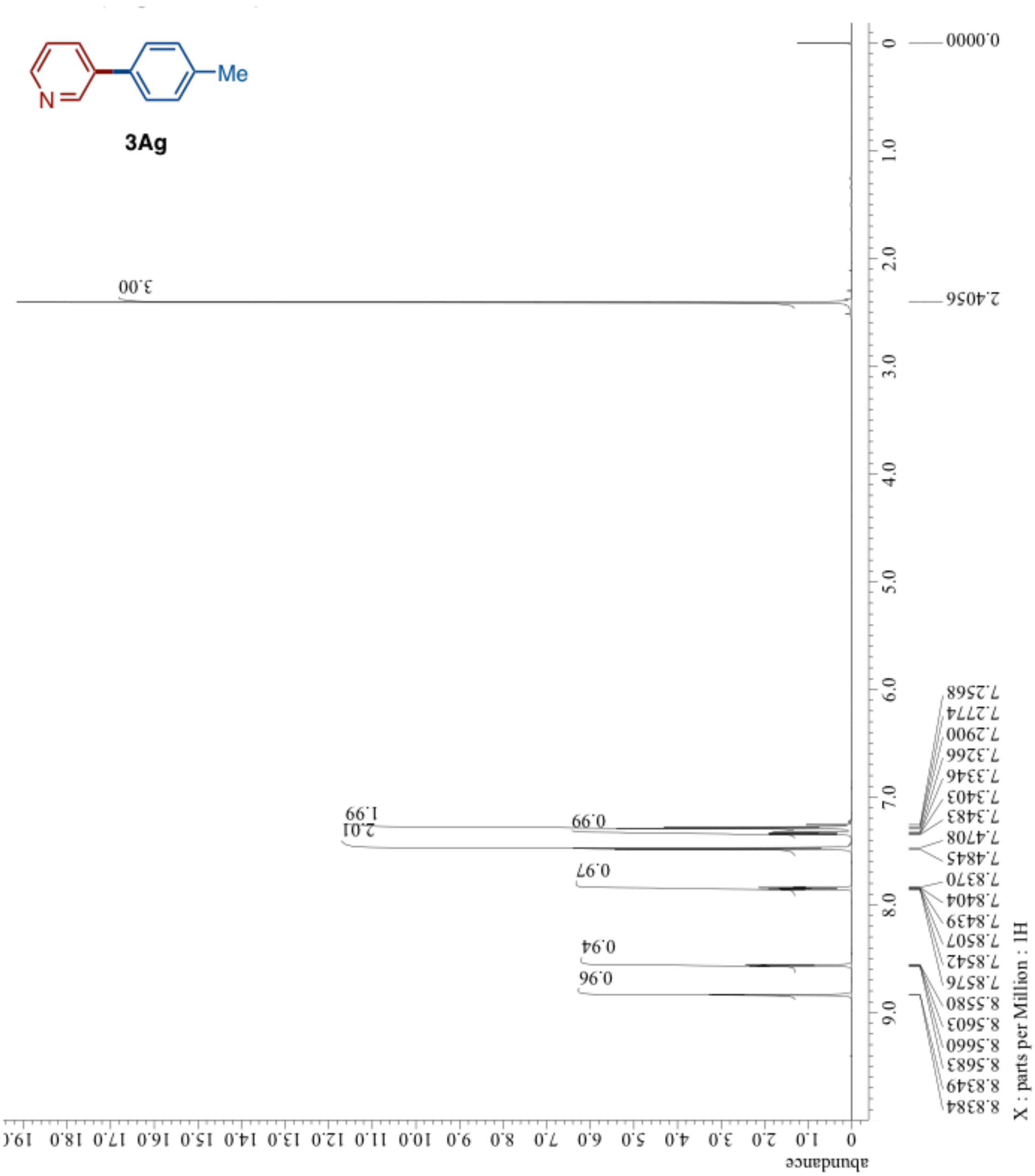


**Supplementary Figure 105. $^{1}H$ NMR (600 MHz, $CDCl_3$) of 3Ag**

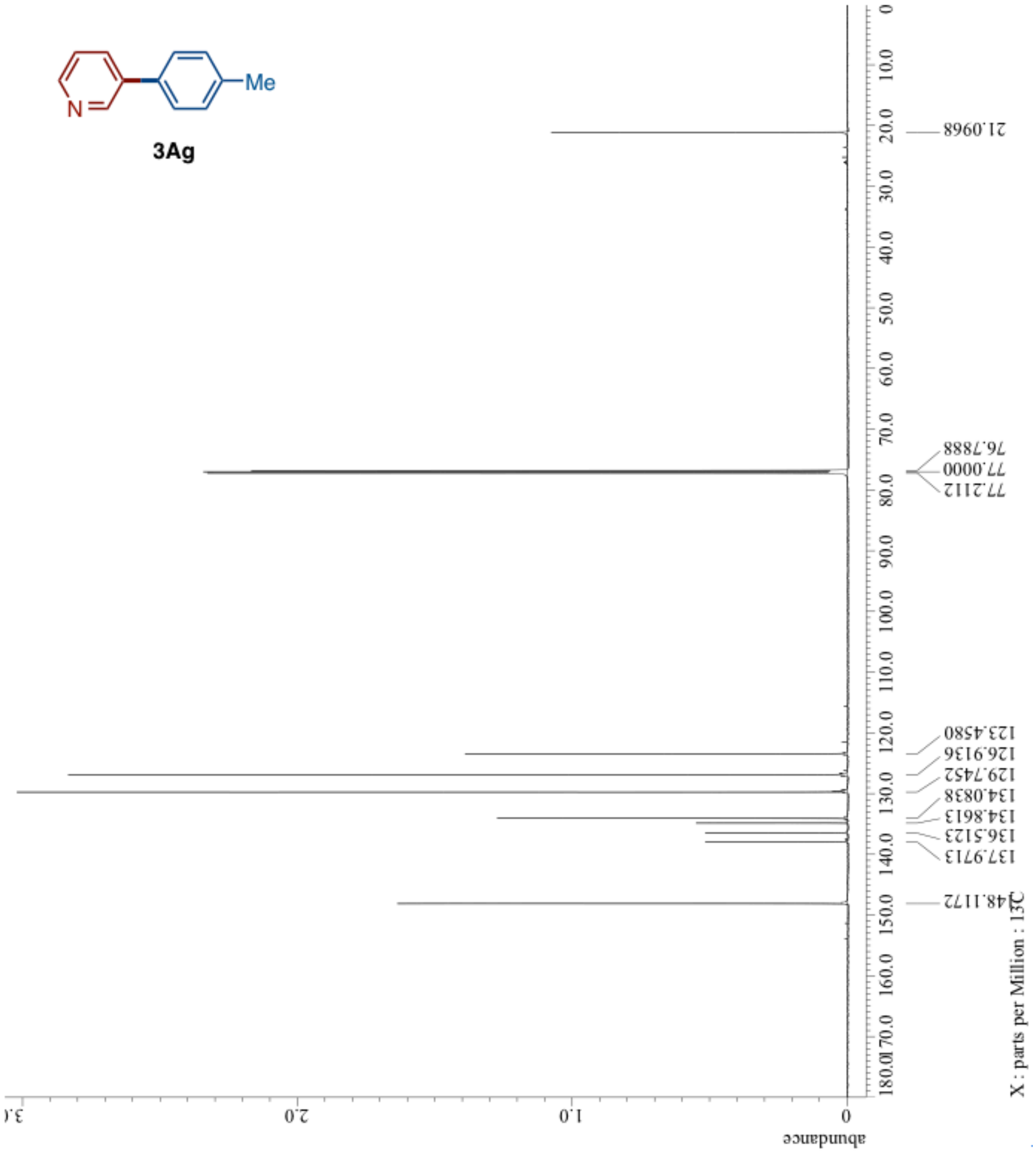


**Supplementary Figure 106.** $^{13}$**C NMR (150 MHz,** $CDCl_3$**) of 3Ag**

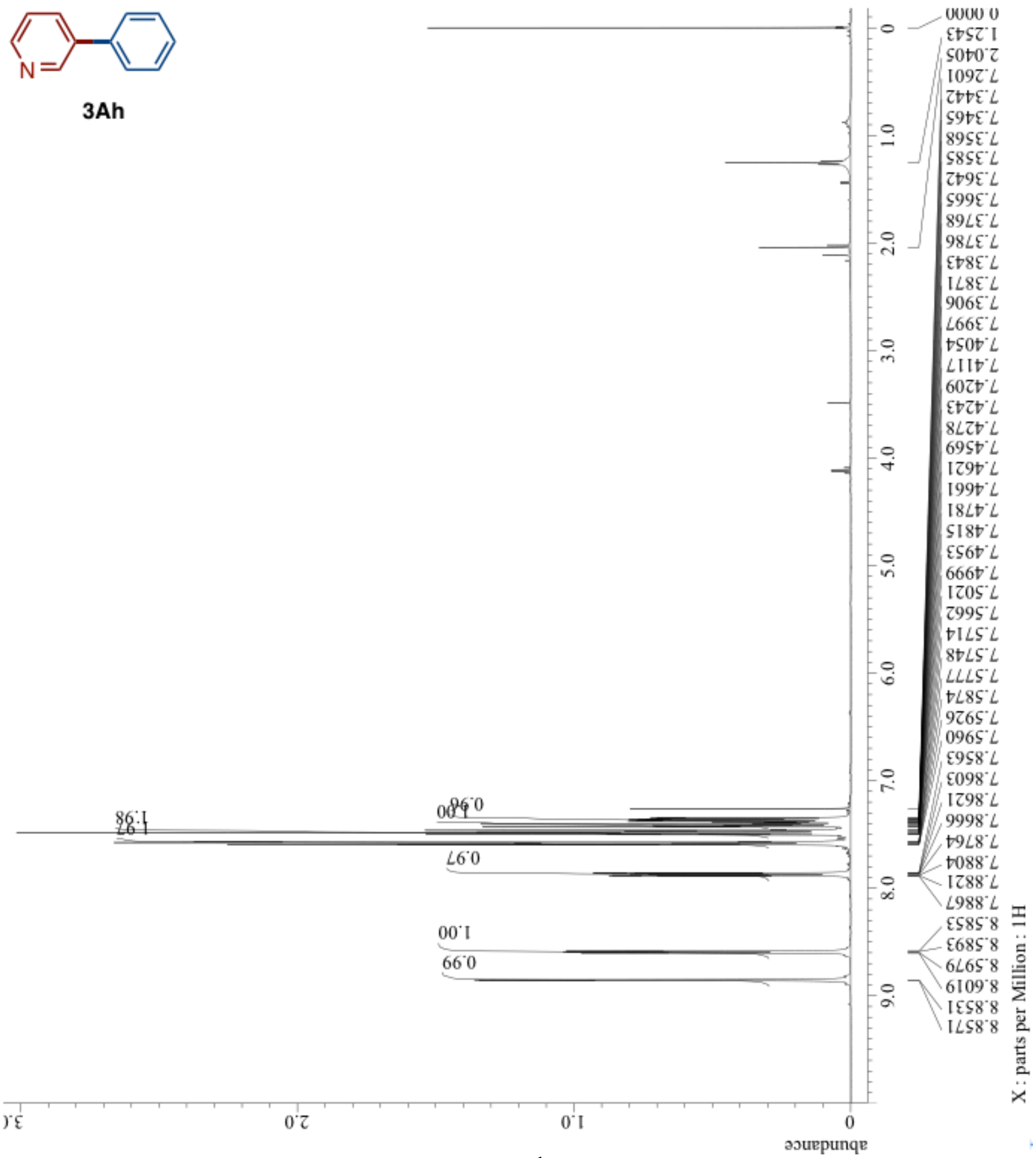


**Supplementary Figure 107. $^1$H NMR (400 MHz, $CDCl_3$) of 3Ah**

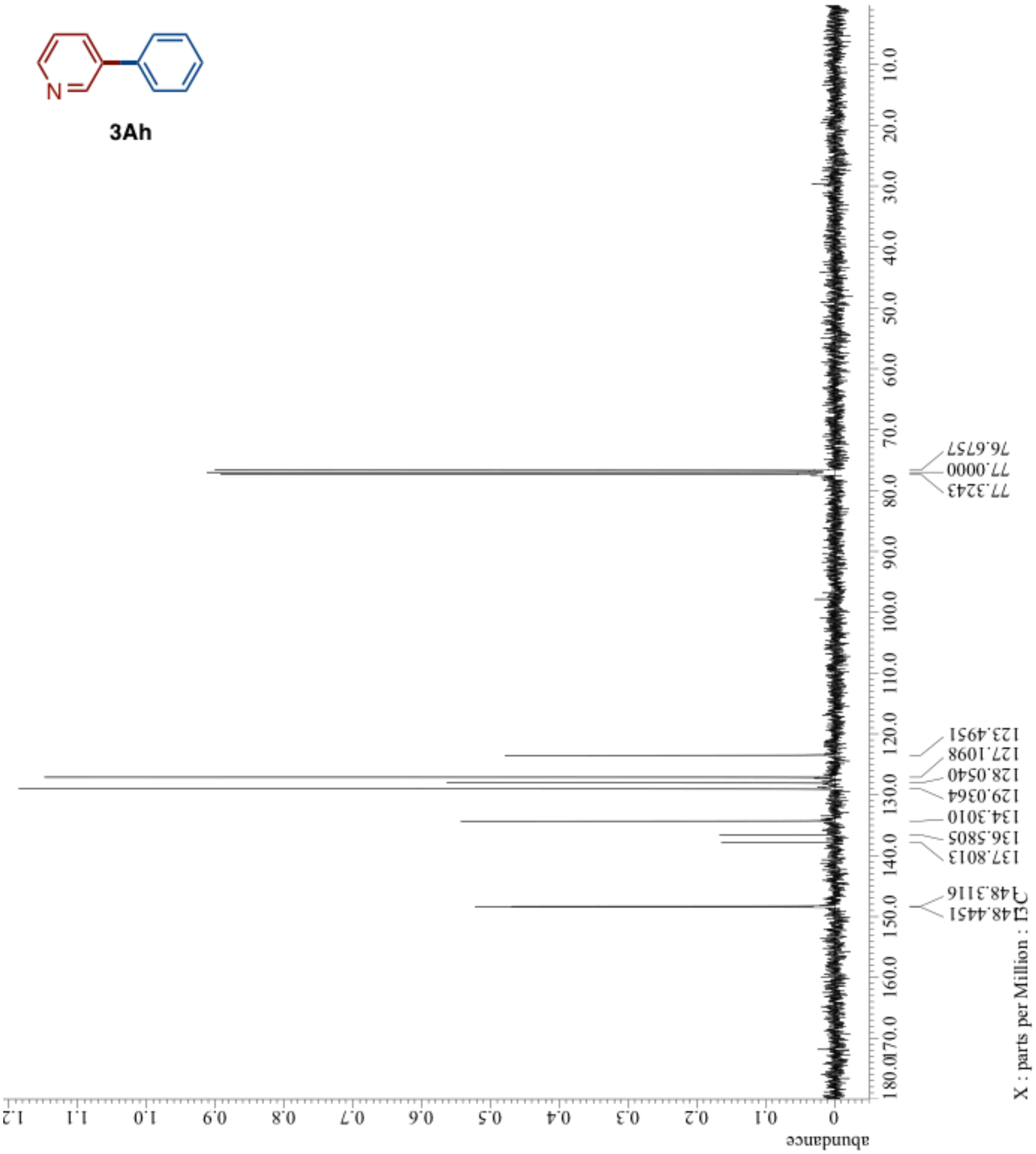


**Supplementary Figure 108.** $^{13}$**C NMR (100 MHz,** $CDCl_3$**) of 3Ah**

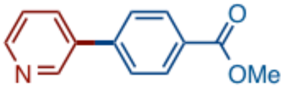


**3Ai**

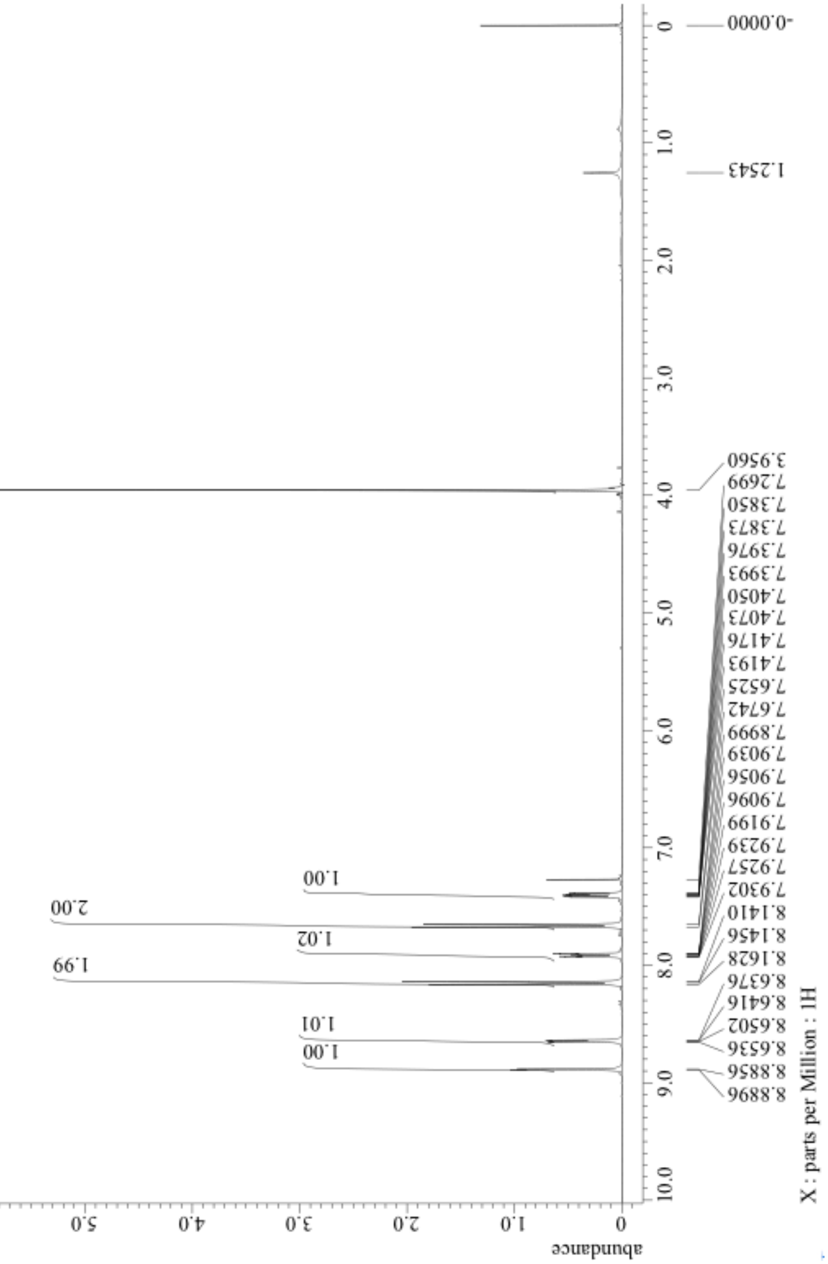


**Supplementary Figure 109. $^1$H NMR (400 MHz, $CDCl_3$) of 3Ai**

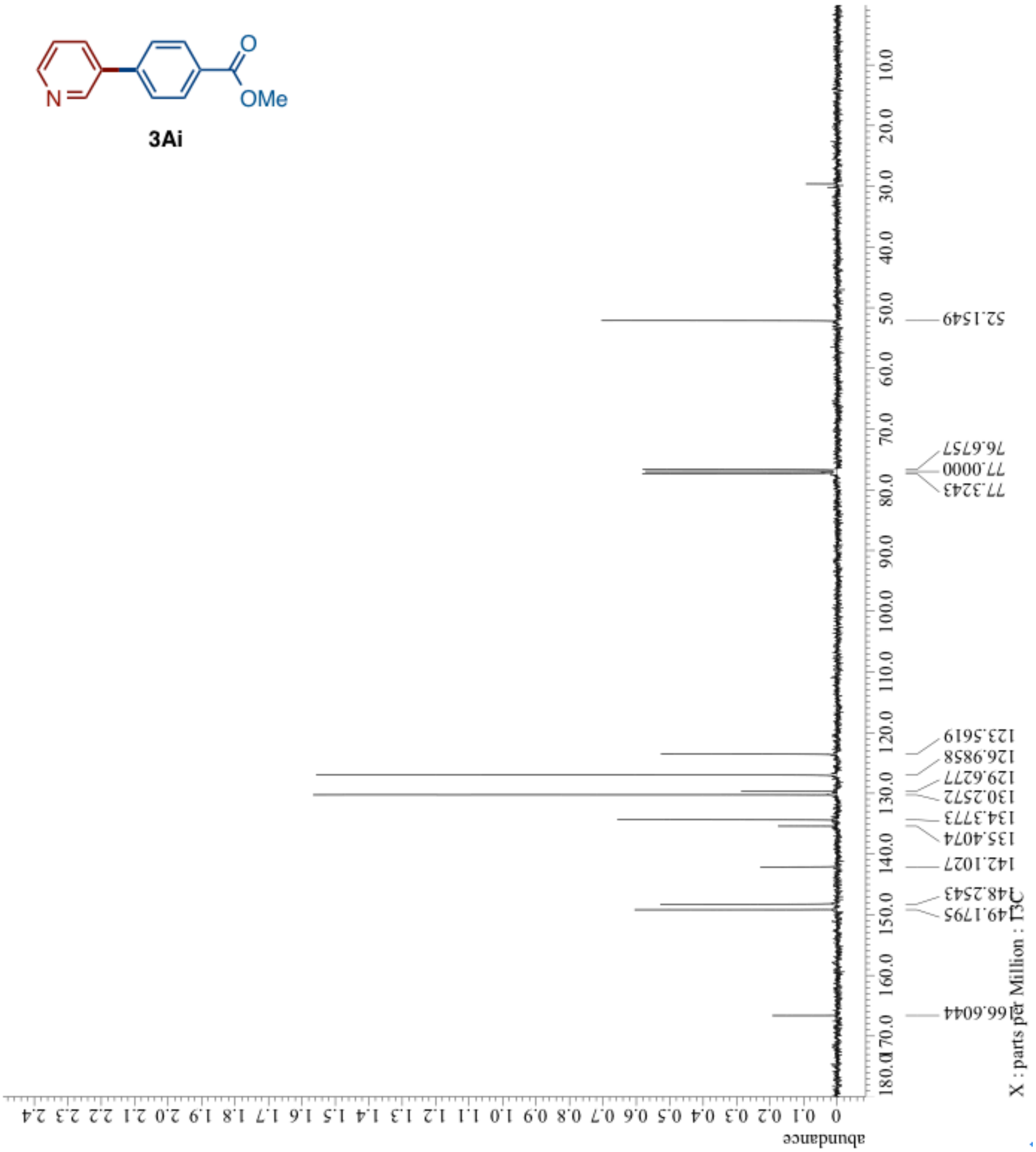


**3Ai**

**Supplementary Figure 110.** $^{13}$**C NMR (100 MHz,** $CDCl_3$**) of 3Ai**

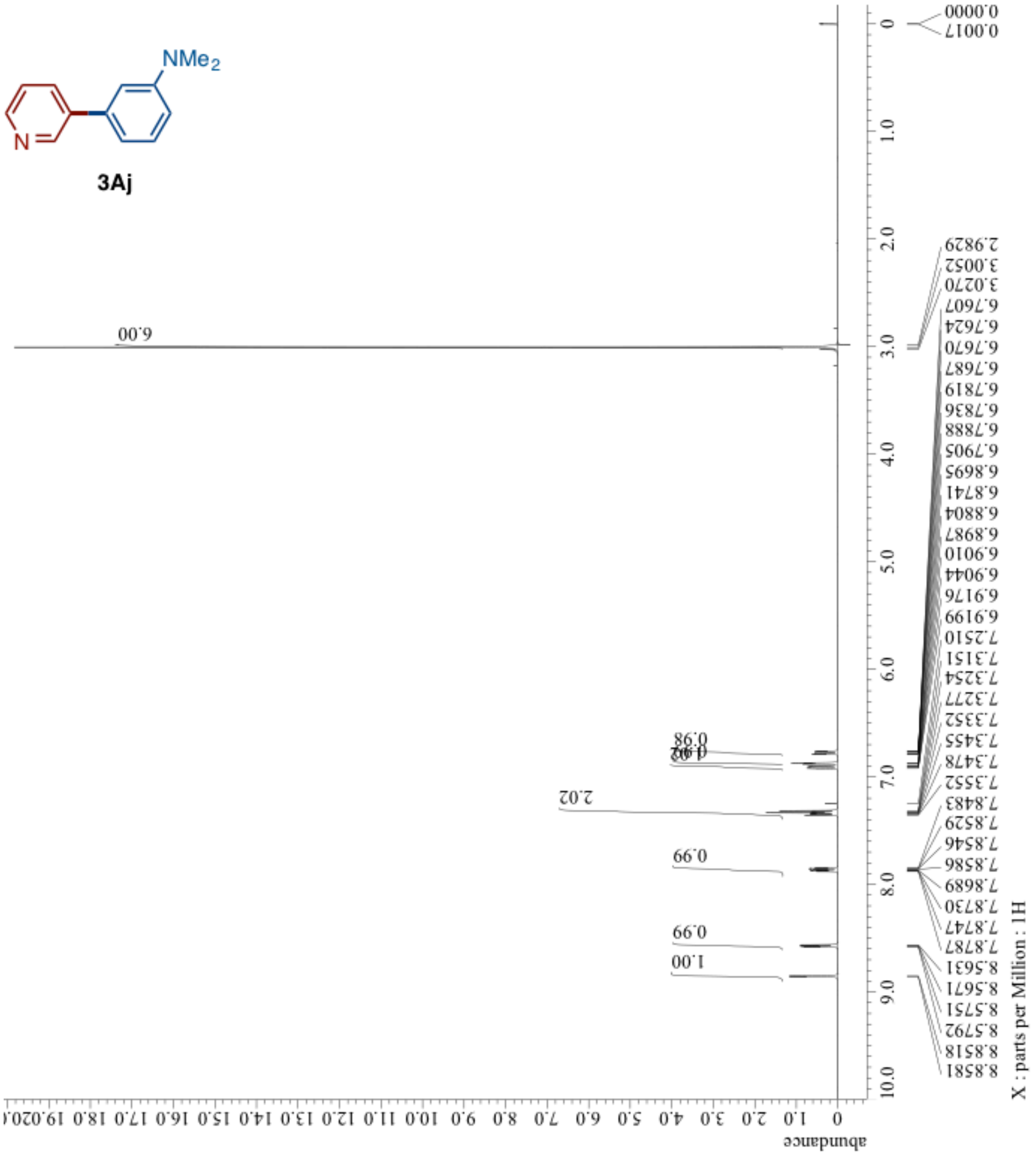


**3Aj**

**Supplementary Figure 111. $^{1}$H NMR (400 MHz, $CDCl_3$) of 3Aj**

$^{13}$C NMR (**3Aj**: 100 MHz)

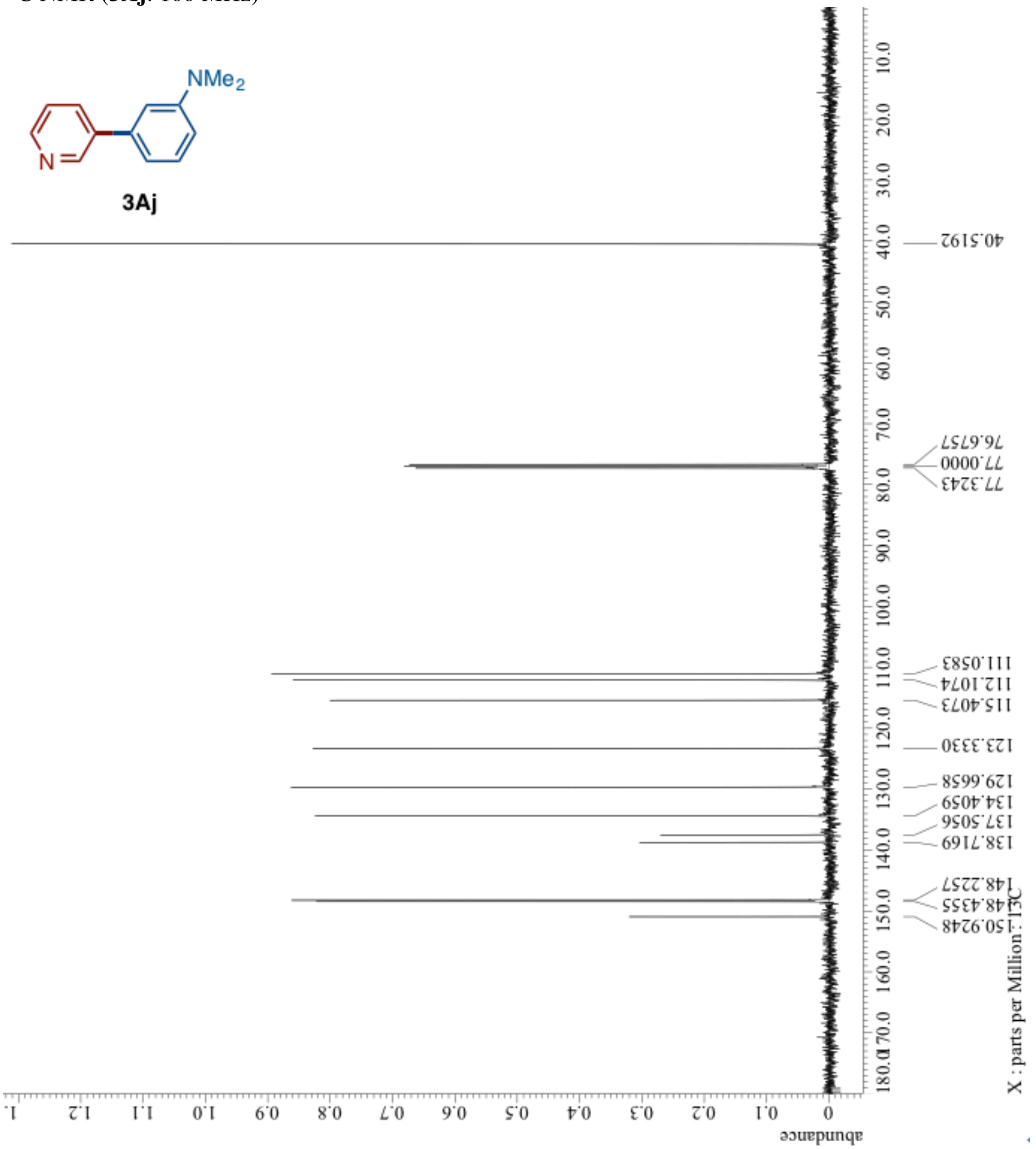


**Supplementary Figure 112.** $^{13}$**C NMR (100 MHz,** $CDCl_3$**) of 3Aj**

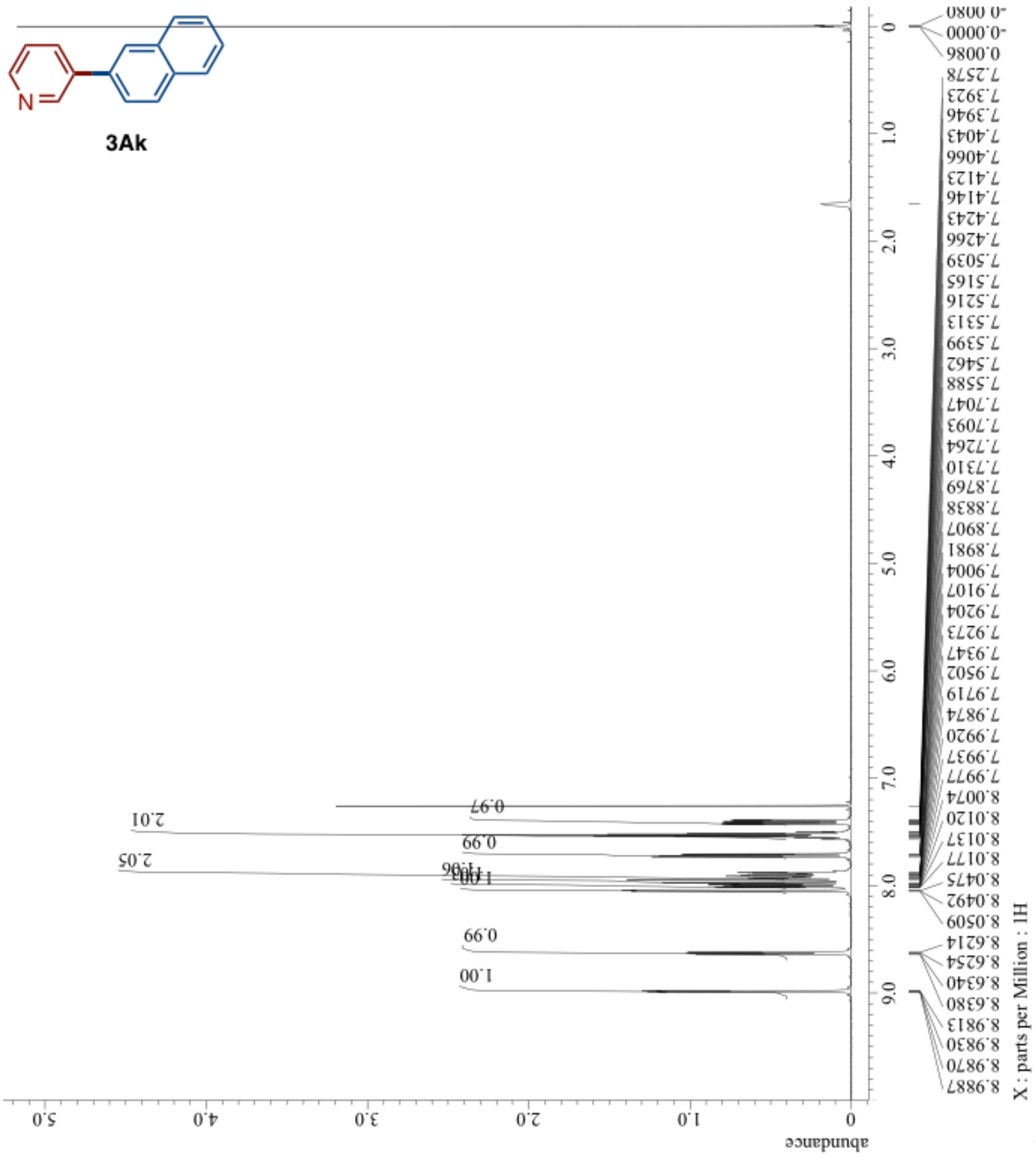


**Supplementary Figure 113. $^{1}$H NMR (400 MHz, $CDCl_3$) of 3Ak**

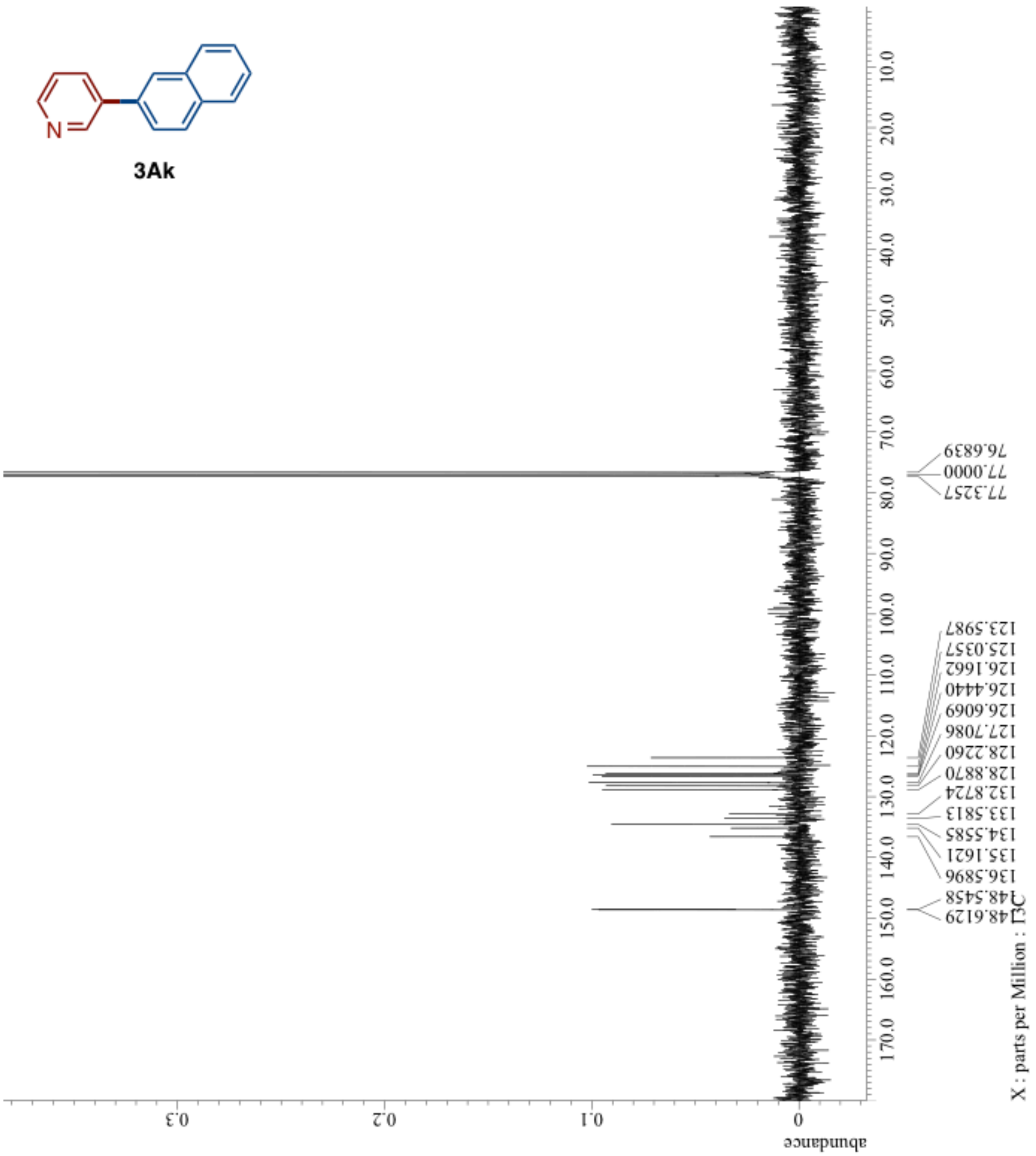


**Supplementary Figure 114.** $^{13}$**C NMR (100 MHz,** $CDCl_3$**) of 3Ak**

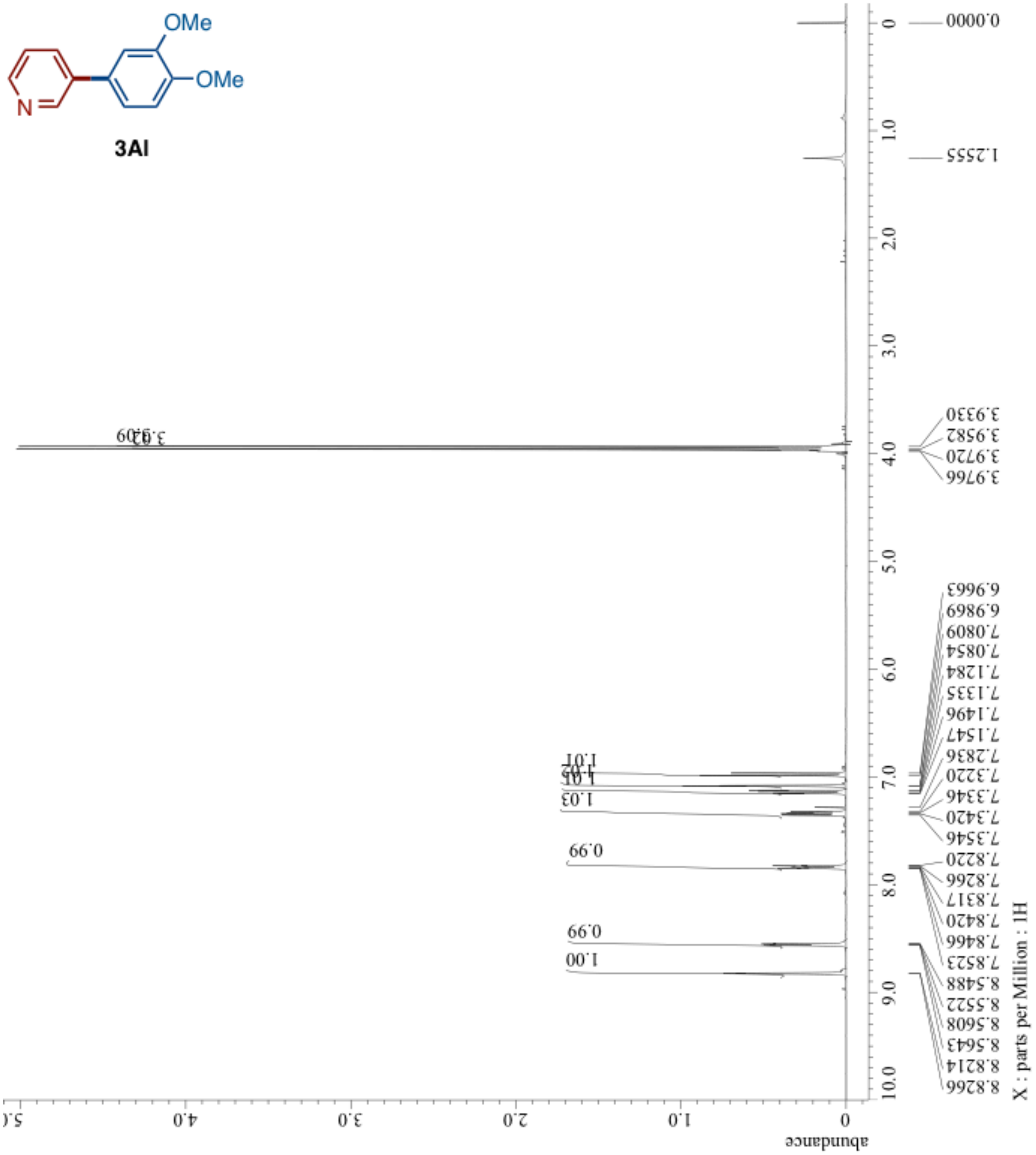


**Supplementary Figure 115.** $^{1}$**H NMR (400 MHz,** $CDCl_3$**) of 3Al**

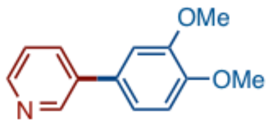


**3Al**

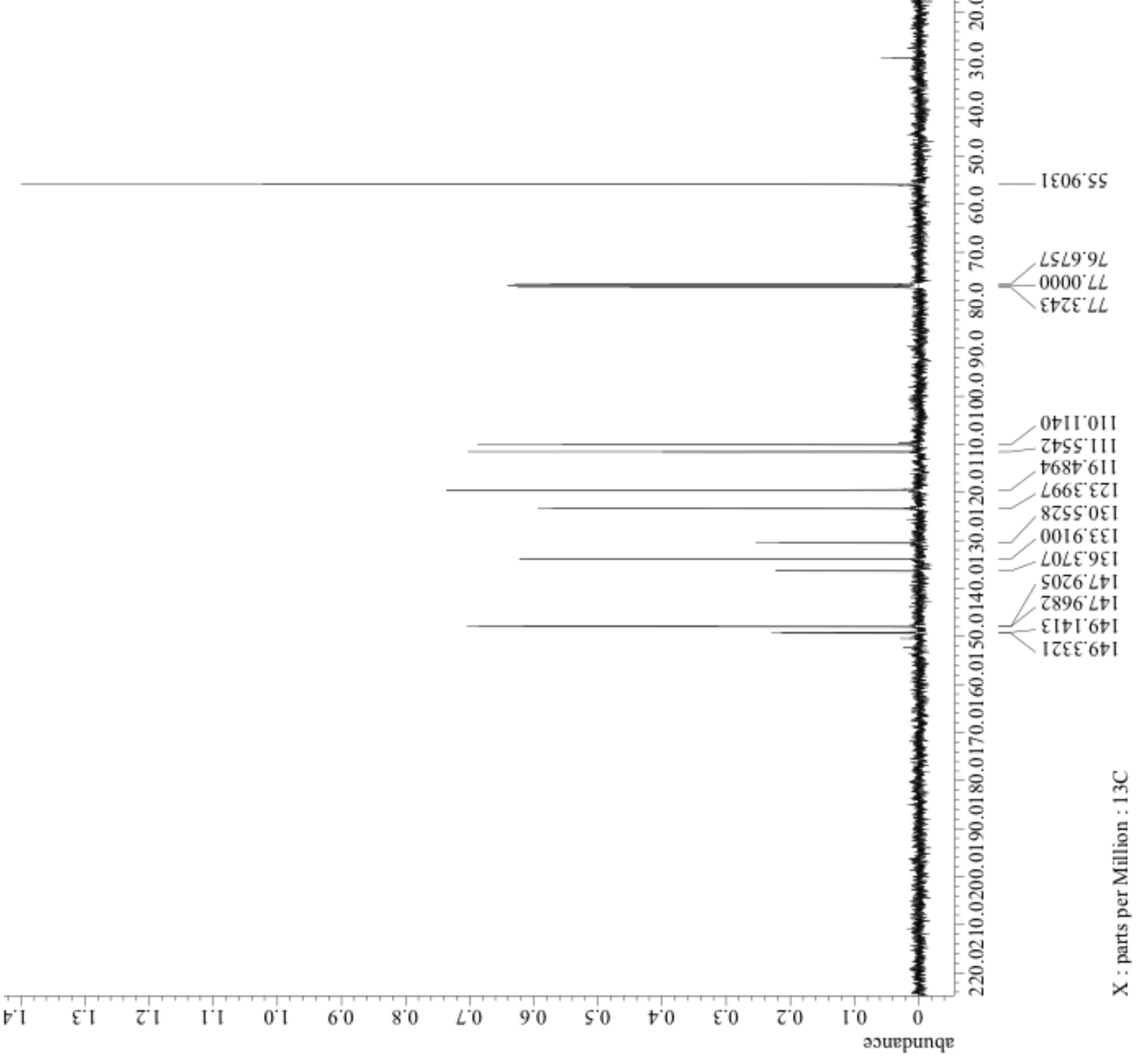


**Supplementary Figure 116. $^{13}C$ NMR (100 MHz, $CDCl_3$) of 3Al**

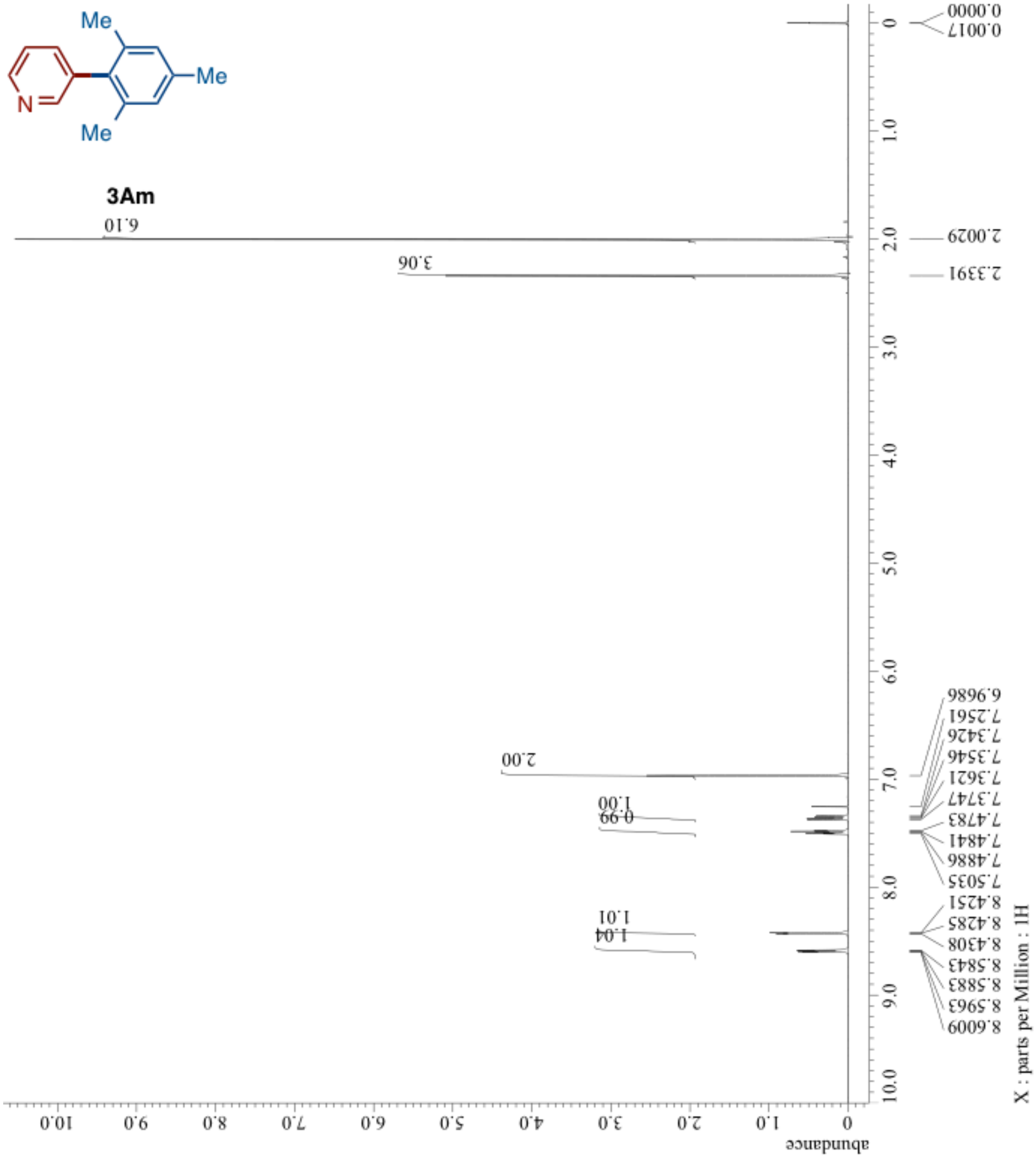


**Supplementary Figure 117.** $^{1}$**H NMR (400 MHz,** $CDCl_3$**) of 3Am**

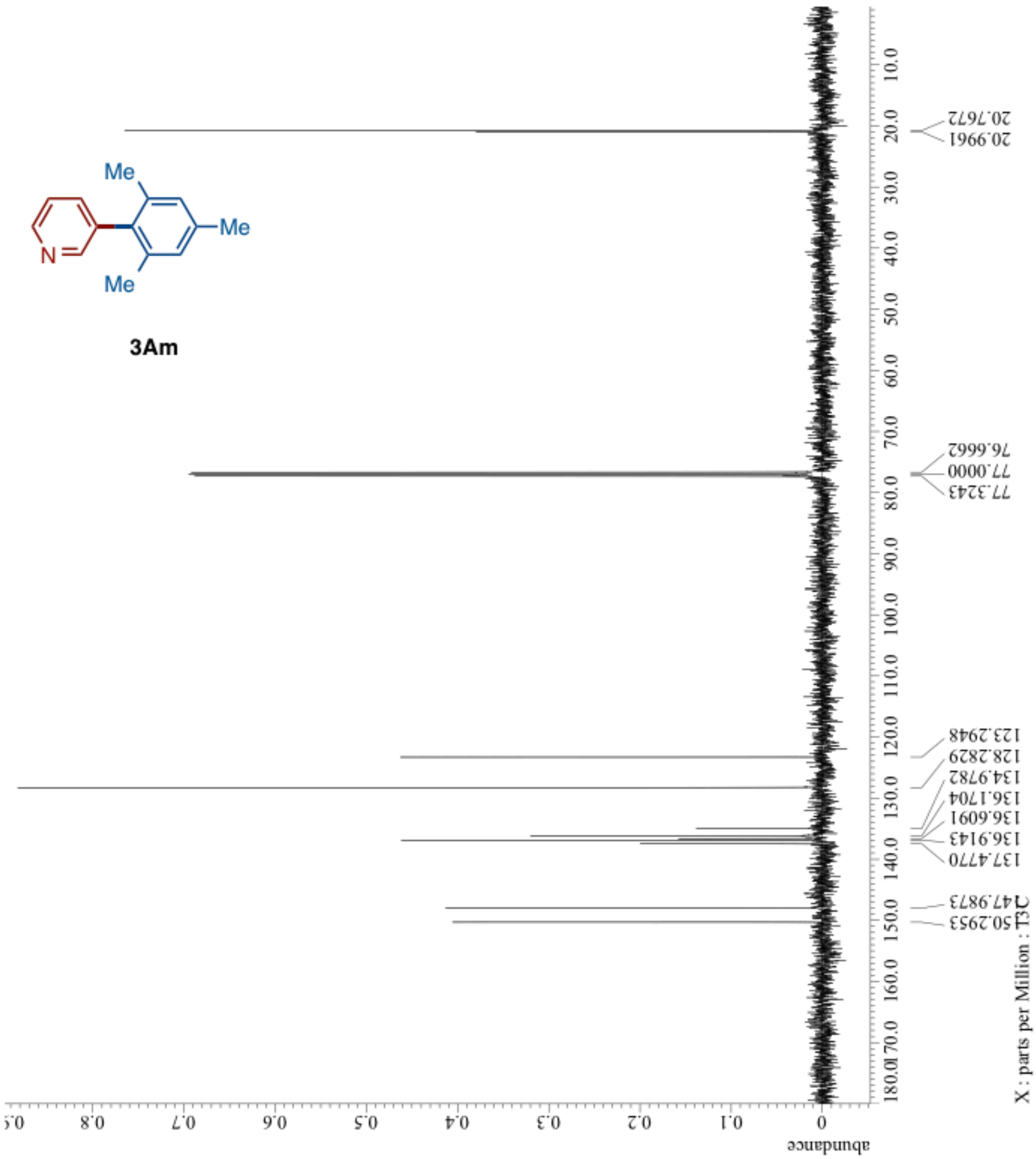


**Supplementary Figure 118.** $^{13}$**C NMR (100 MHz,** $CDCl_3$**) of 3Am**

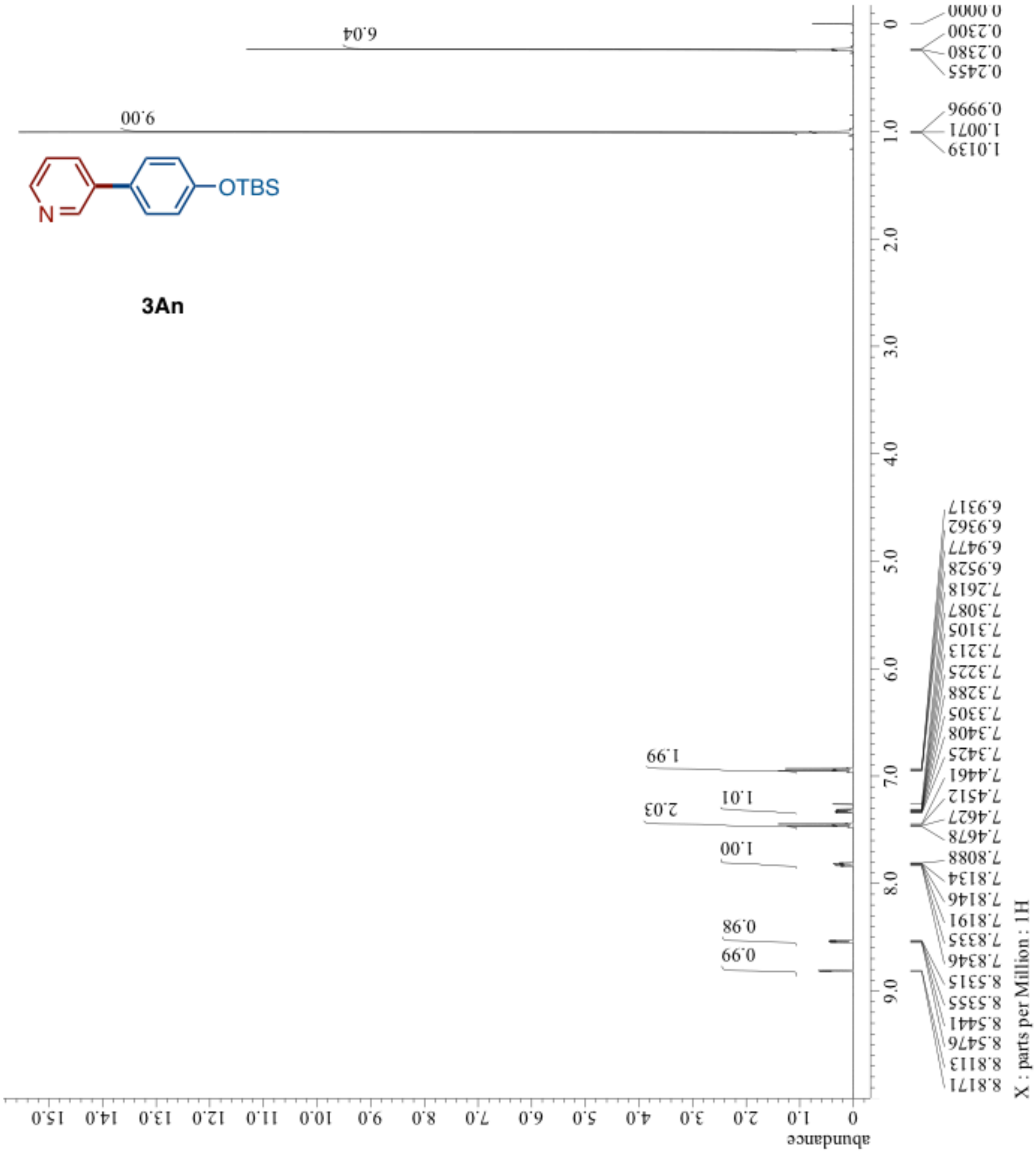


**Supplementary Figure 119.** $^{1}$**H NMR (400 MHz,** $CDCl_3$**) of 3An**

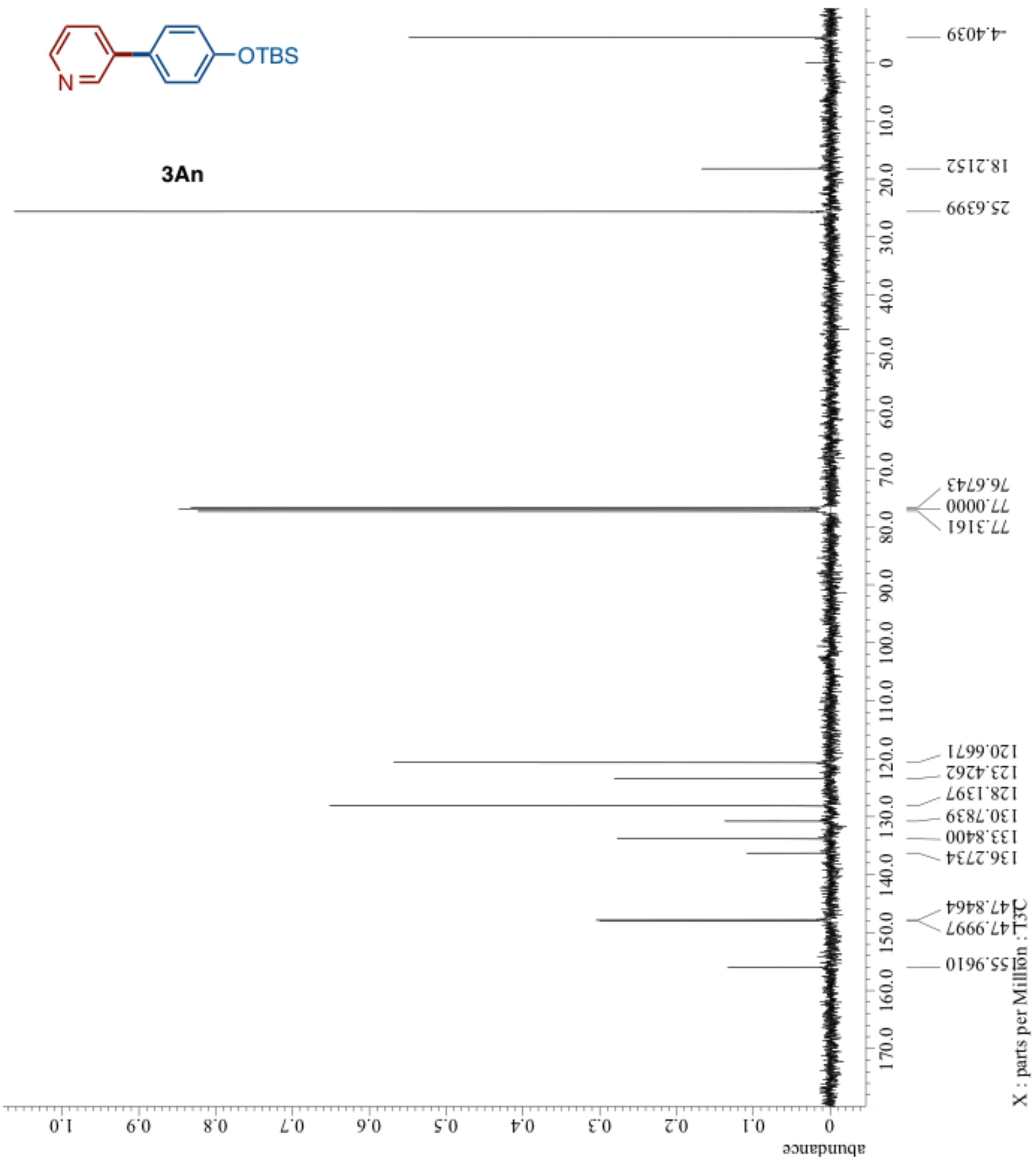


**Supplementary Figure 120.** $^{13}$**C NMR (100 MHz,** $CDCl_3$**) of 3An**

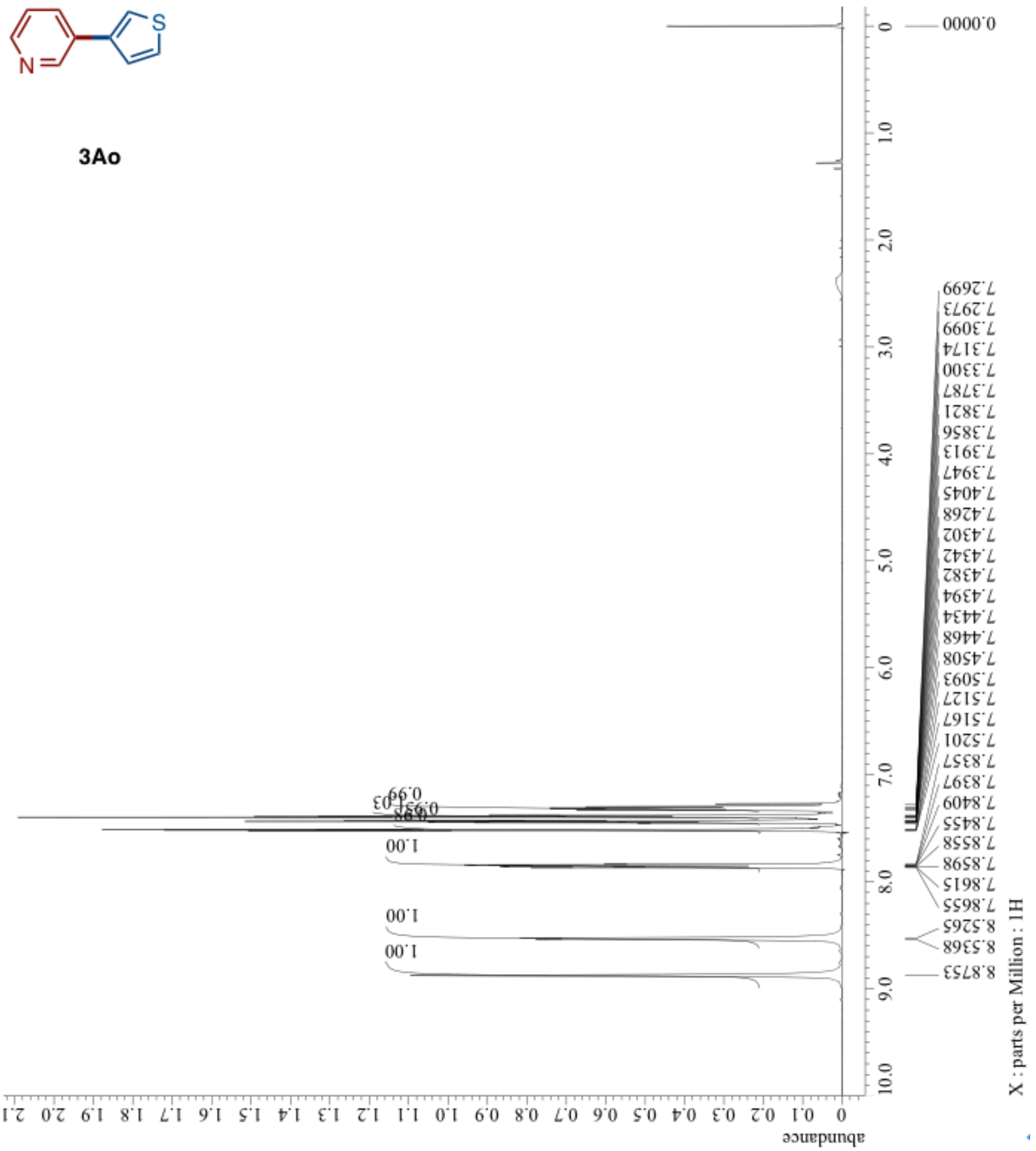


**Supplementary Figure 121. $^1$H NMR (400 MHz, $CDCl_3$) of 3Ao**

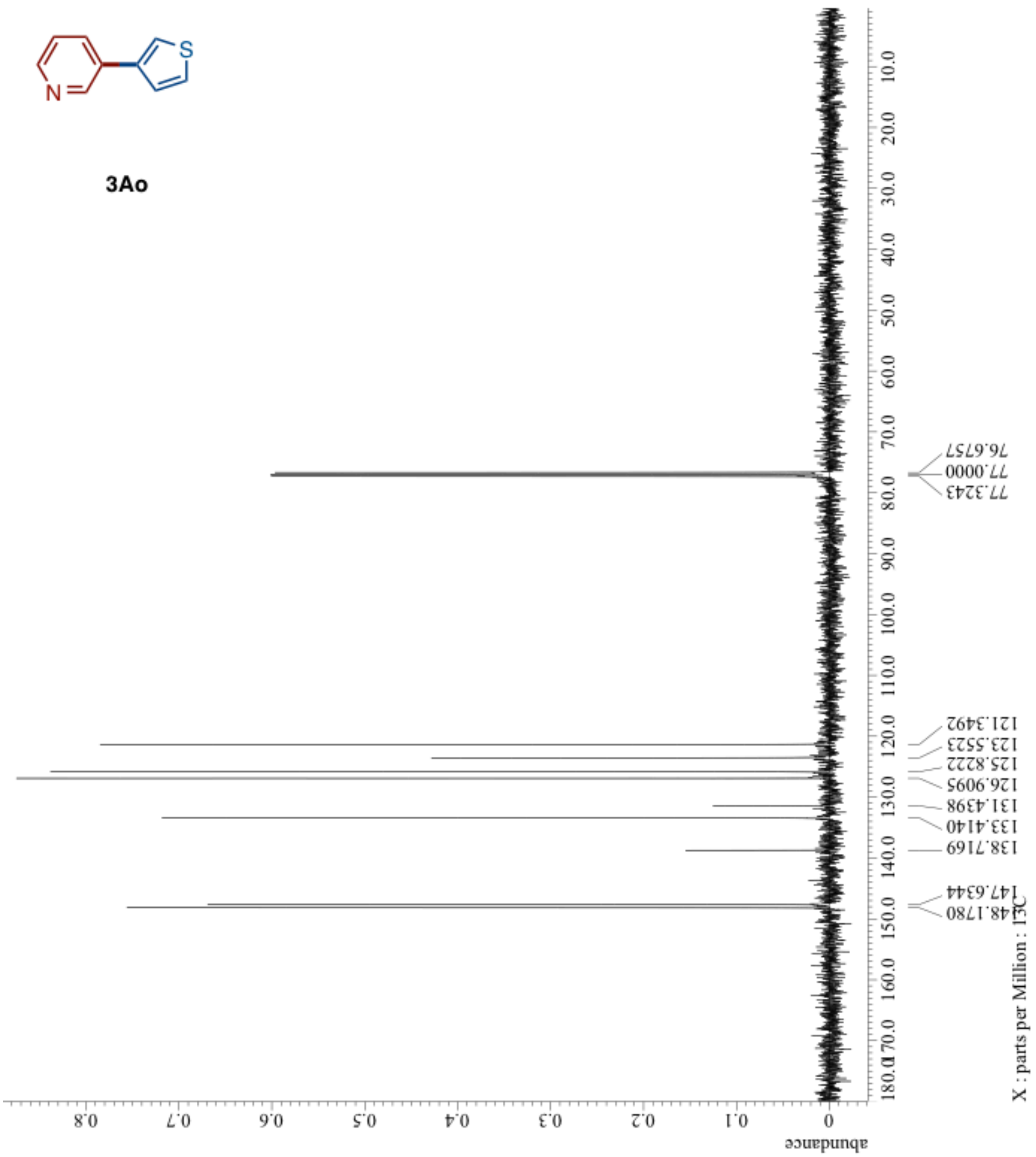


**Supplementary Figure 122.** $^{13}$**C NMR (100 MHz,** $CDCl_3$**) of 3Ao**

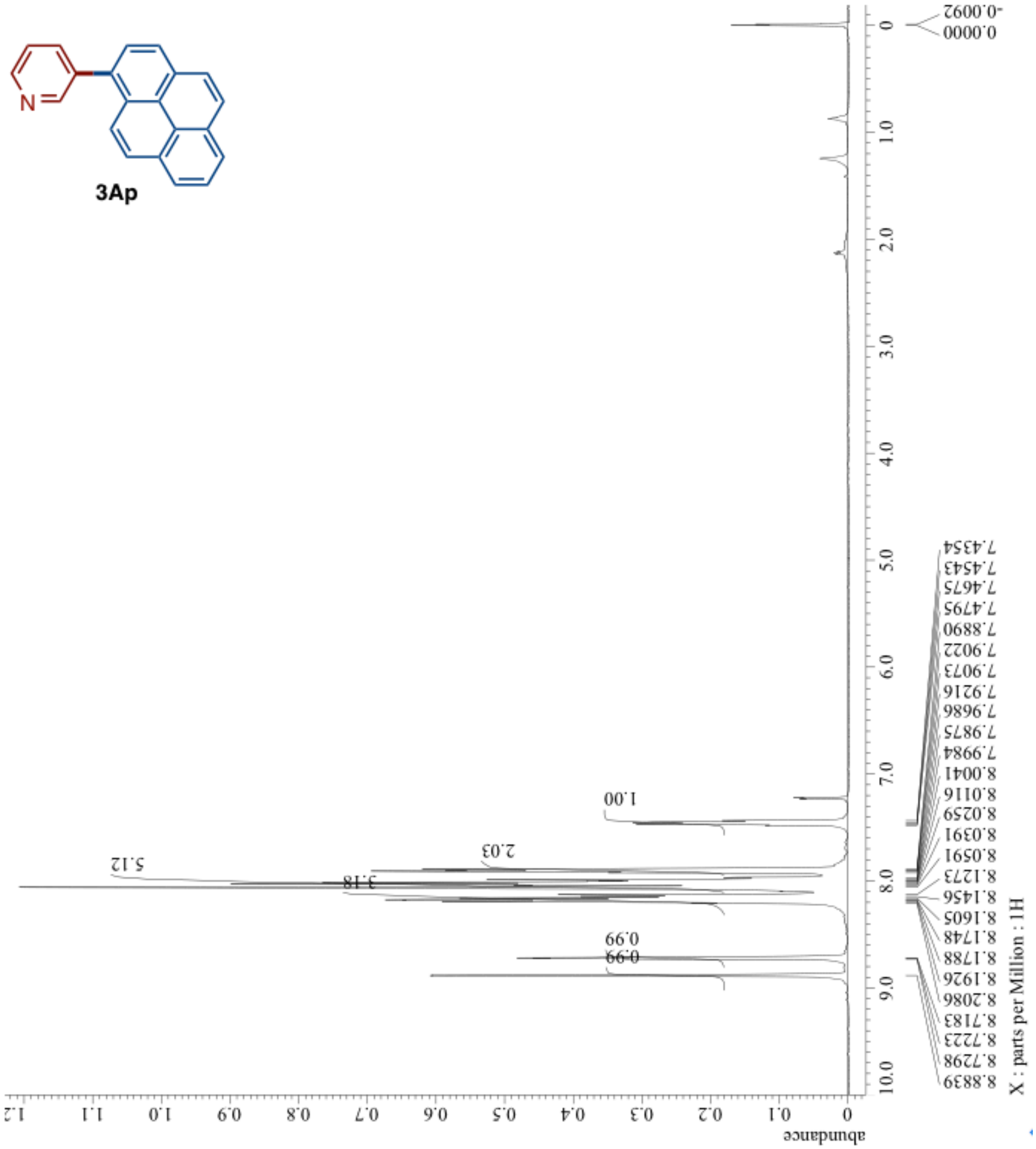


**Supplementary Figure 123.** $^{1}$**H NMR (400 MHz, CDCl$_3$) of 3Ap**

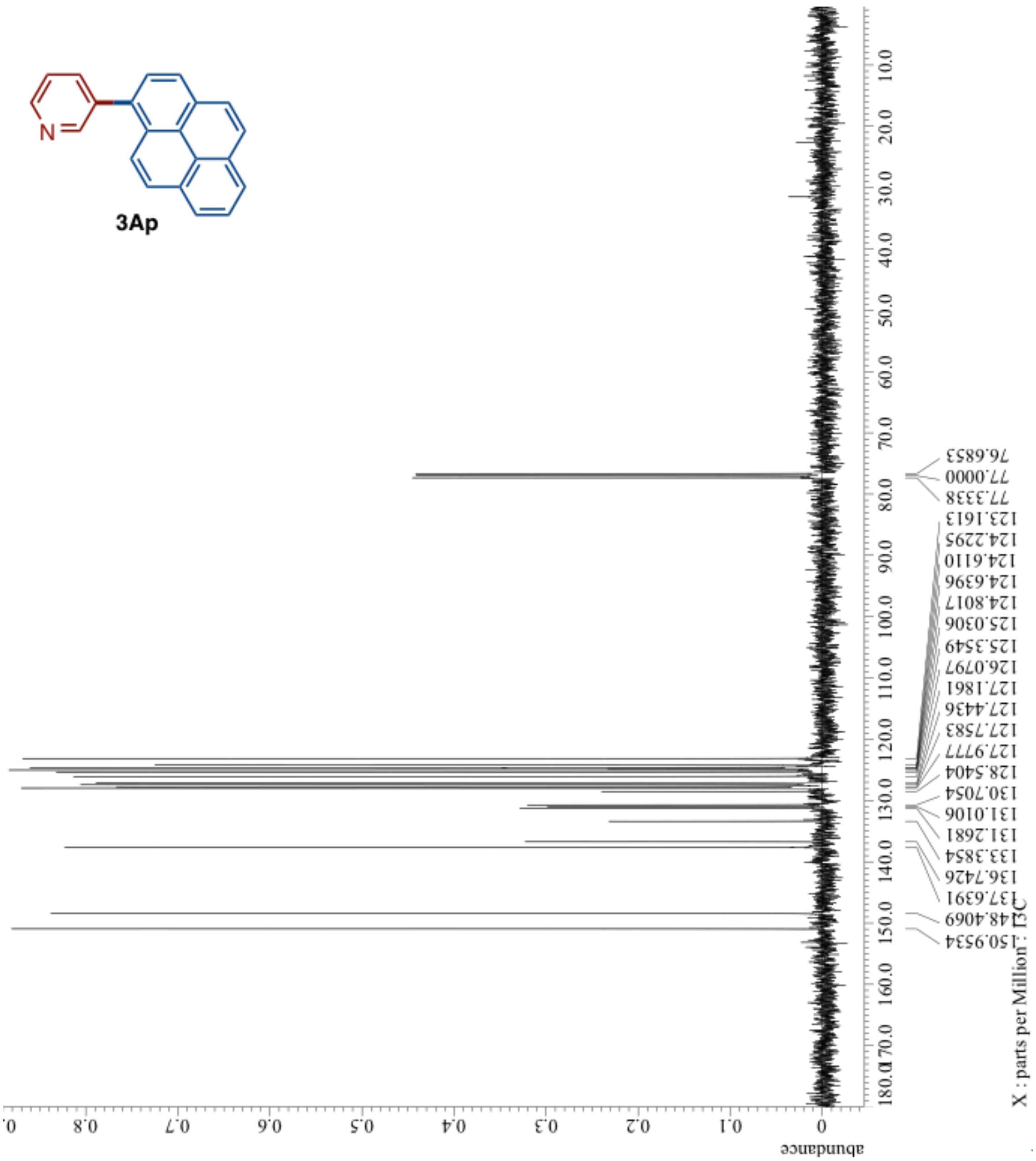


**Supplementary Figure 124.** $^{13}$**C NMR (100 MHz, CDCl$_3$) of 3Ap**

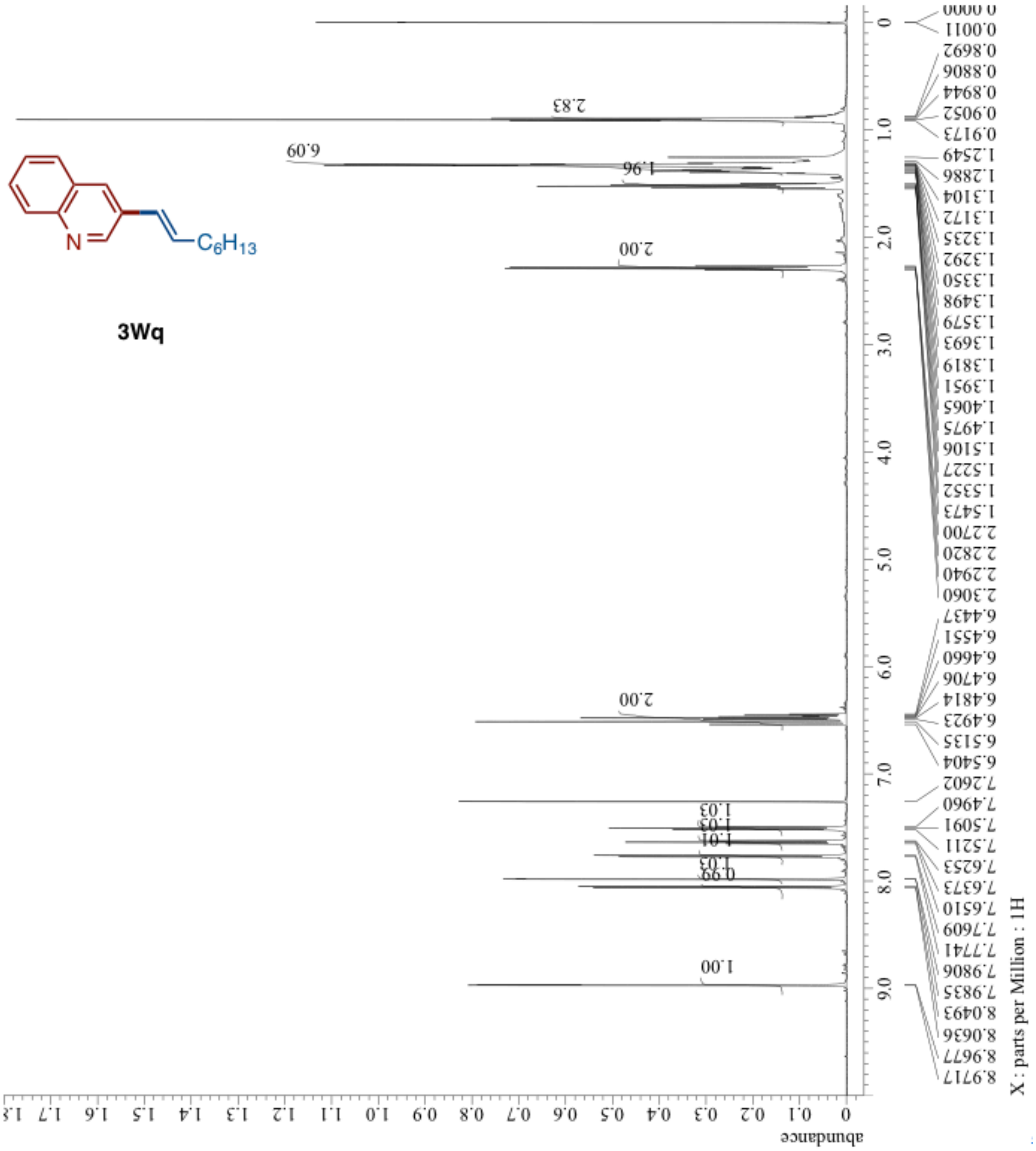


**Supplementary Figure 125. $^1$H NMR (600 MHz, $CDCl_3$) of 3Wq**

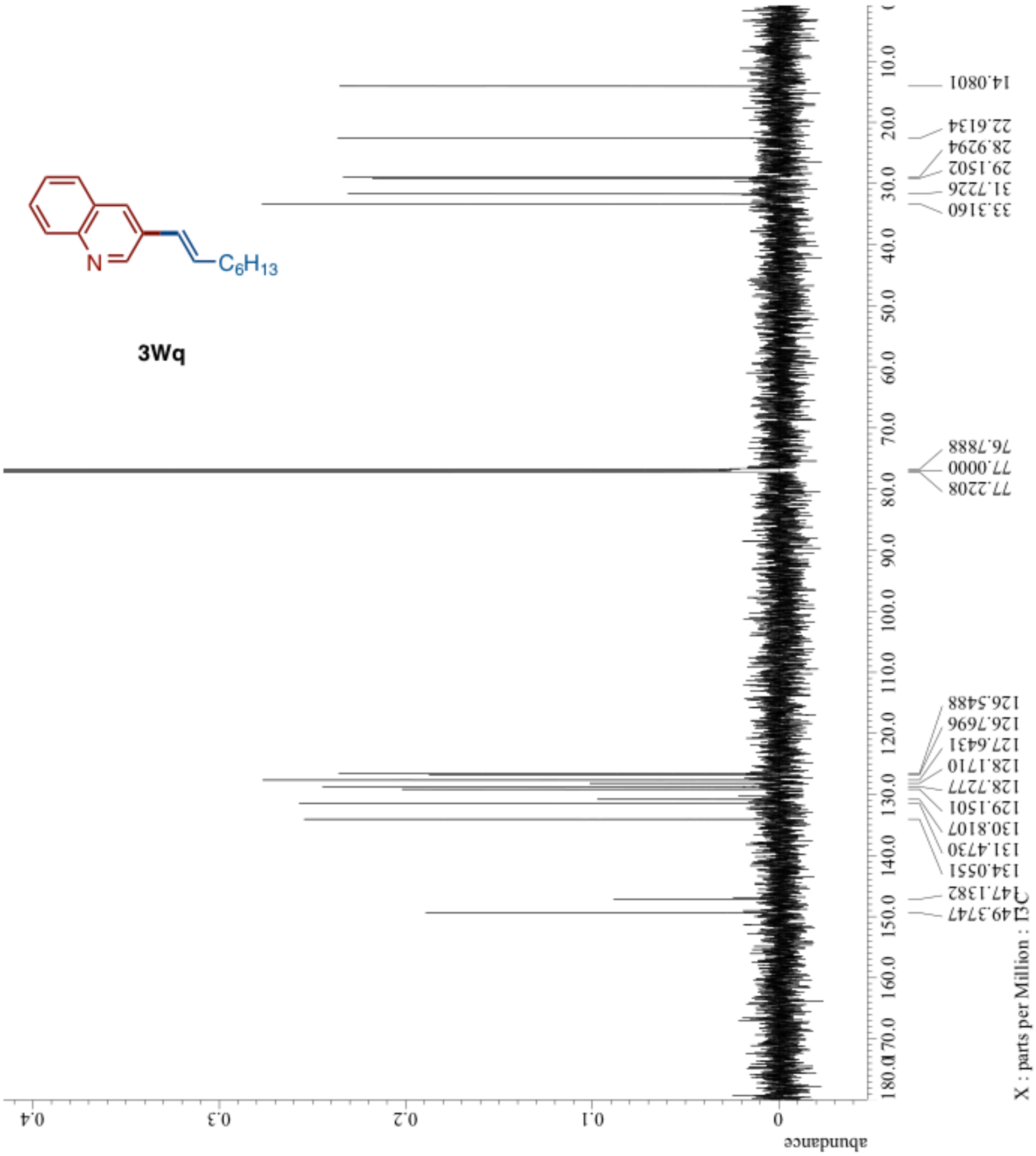


**Supplementary Figure 126.** $^{13}$**C NMR (150 MHz,** $CDCl_3$**) of 3Wq**

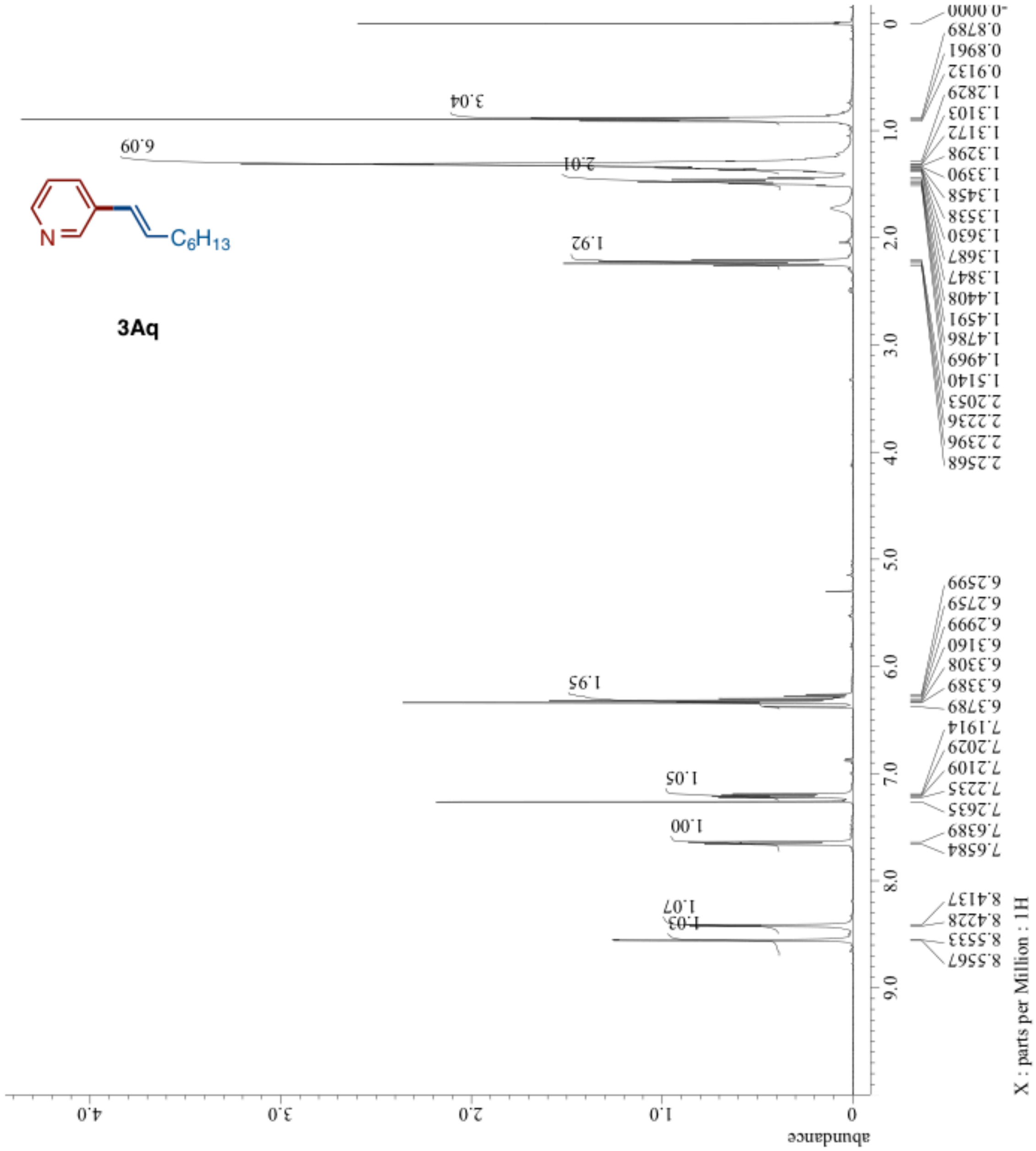

**Supplementary Figure 127.** $^{1}$**H NMR (400 MHz, CDCl$_3$) of 3Aq**

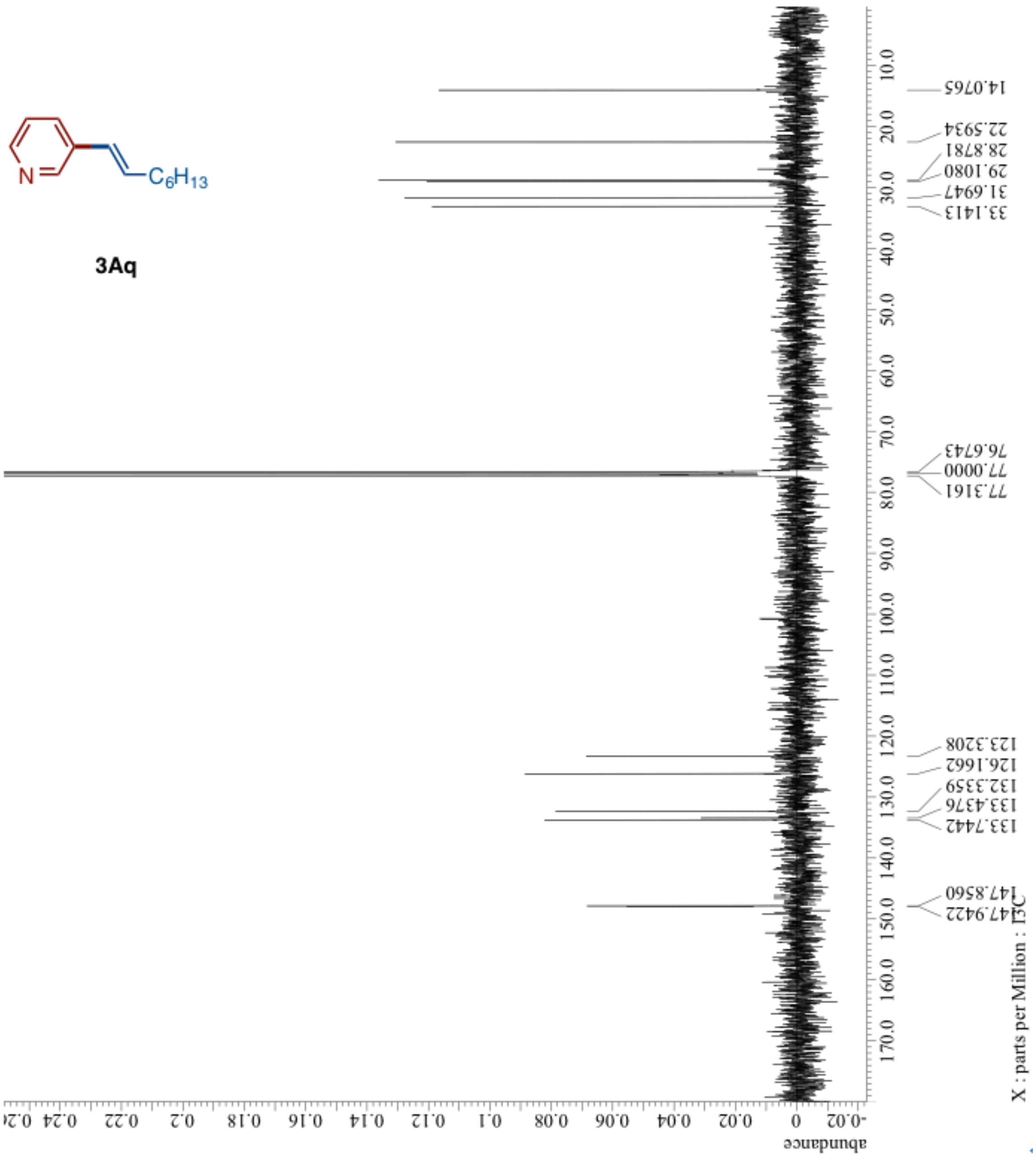

**Supplementary Figure 128.** $^{13}$**C NMR (100 MHz,** $CDCl_3$**) of 3Aq**

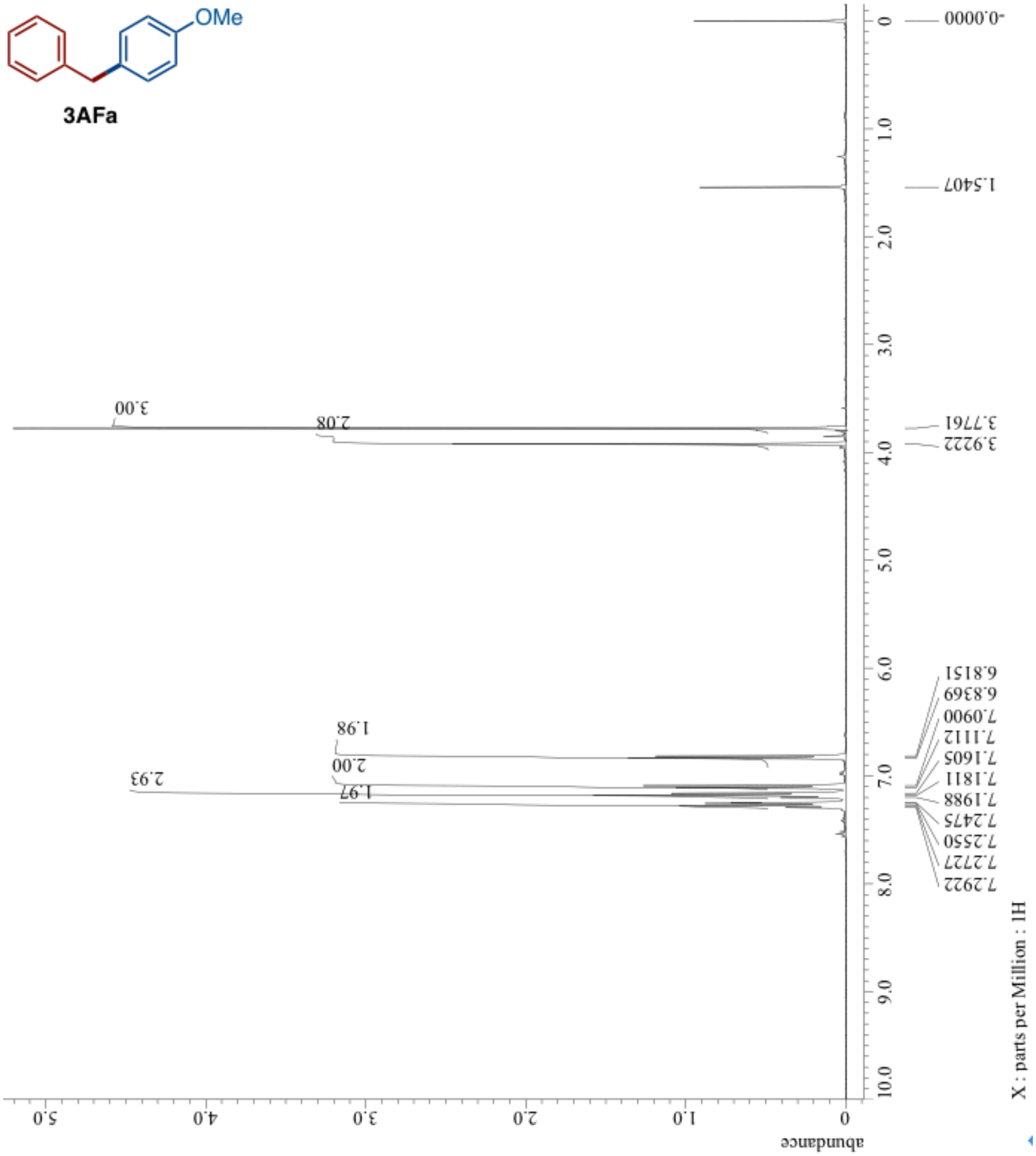


**Supplementary Figure 129.** $^{1}$**H NMR (400 MHz, CDCl$_3$) of 3AFa**

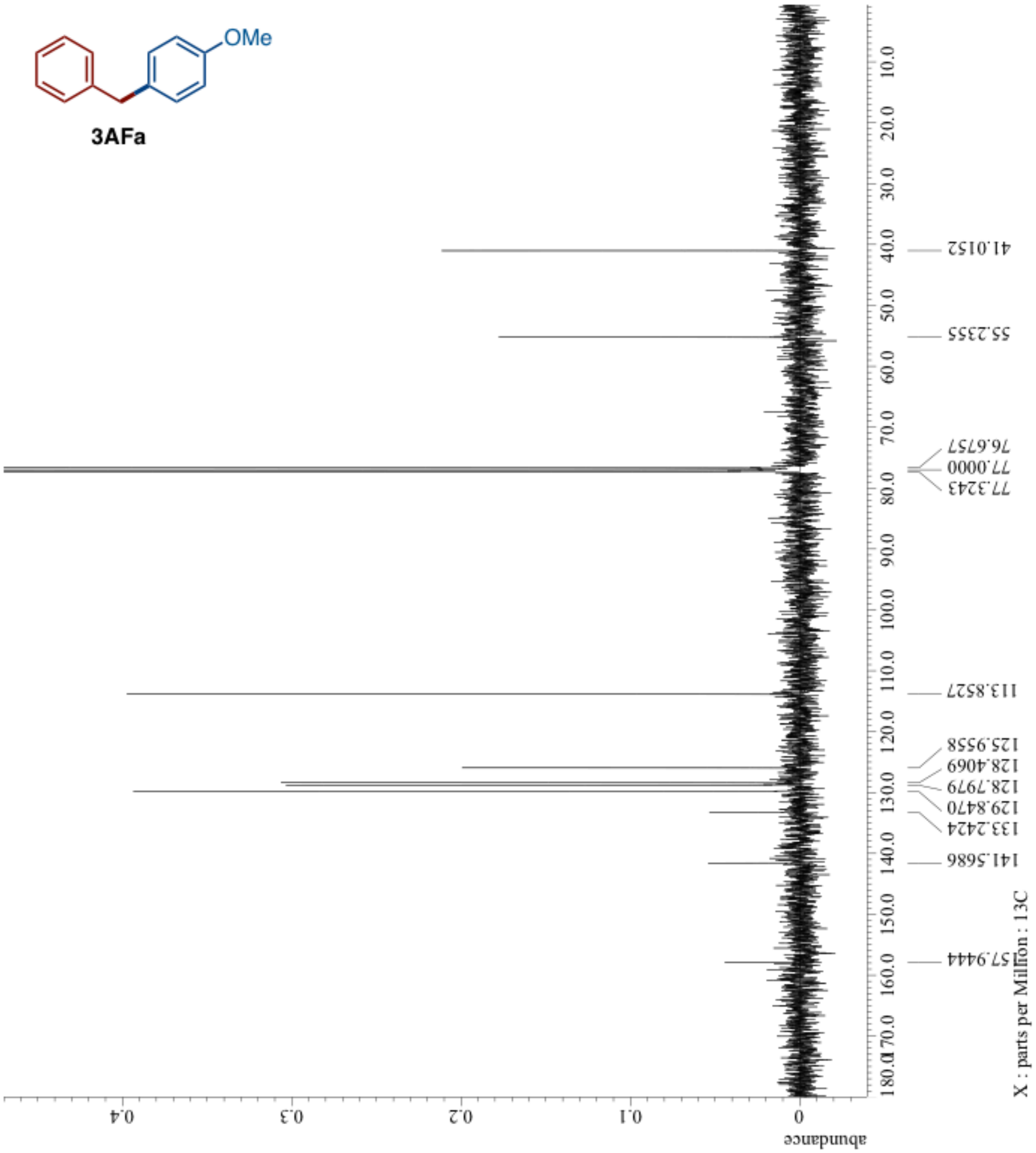


**Supplementary Figure 130. $^{13}$C NMR (100 MHz, $CDCl_3$) of 3AFa**

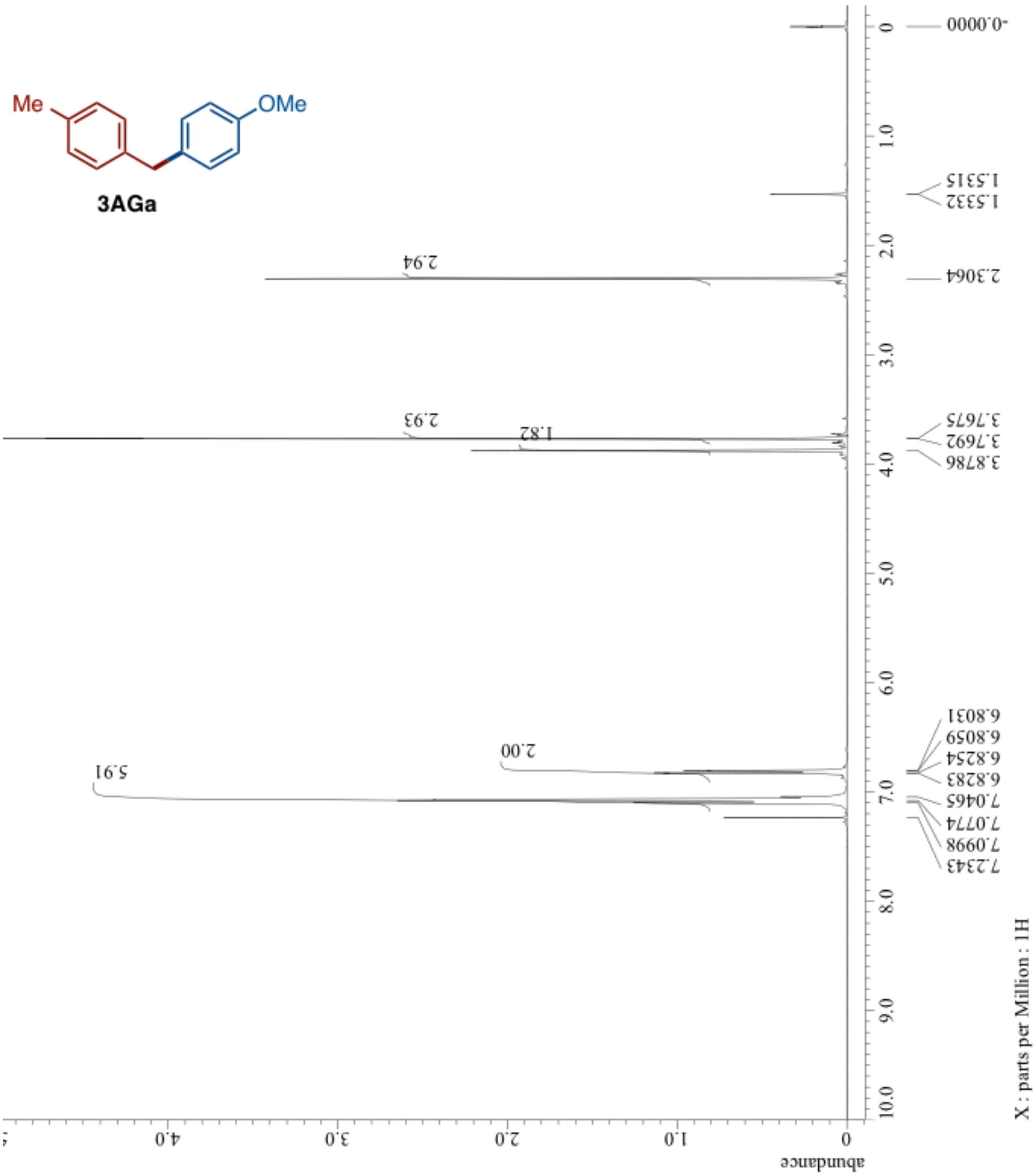


**Supplementary Figure 131. $^{1}H$ NMR (400 MHz, $CDCl_3$) of 3AGa**

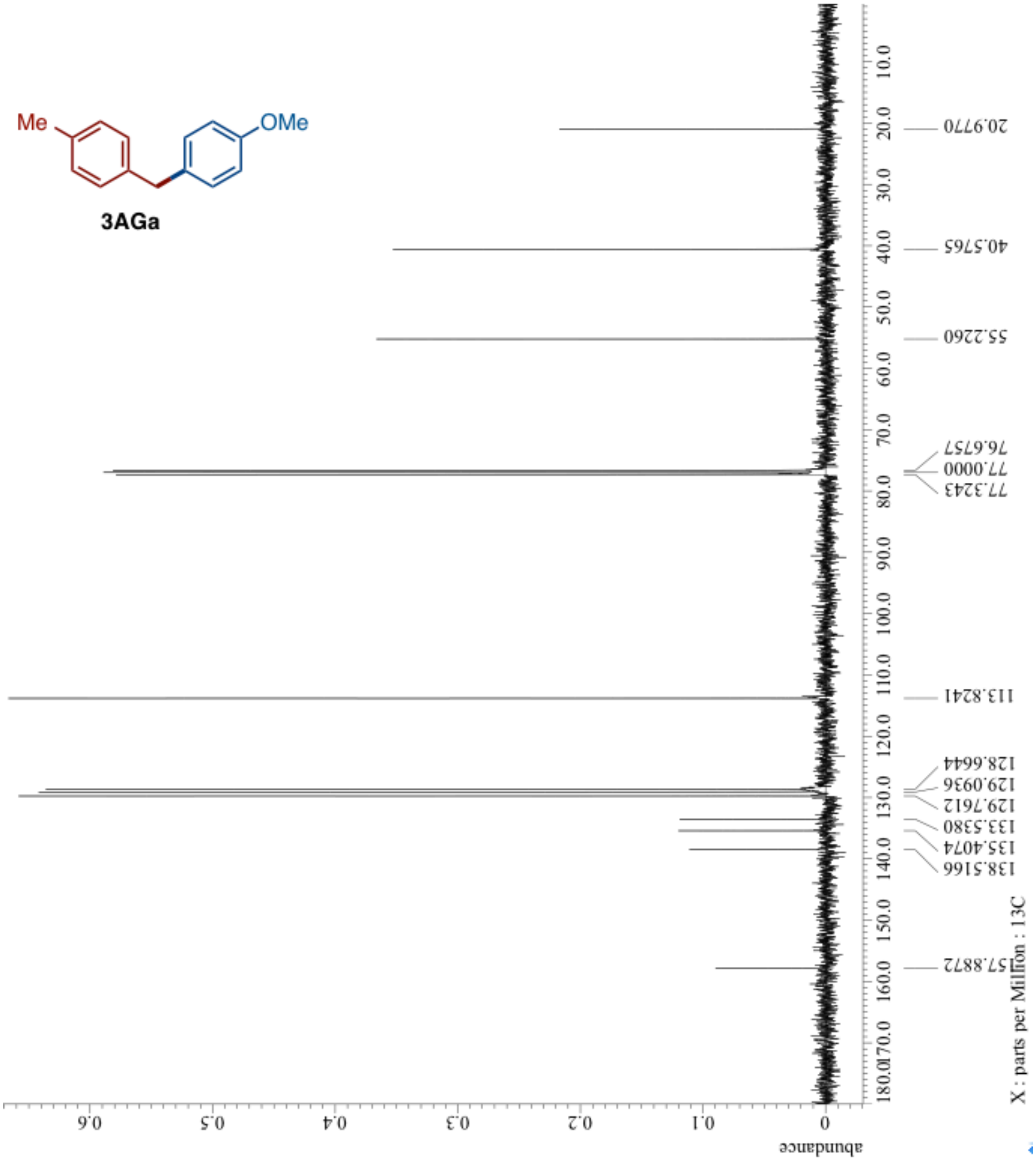


**Supplementary Figure 132.** $^{13}$**C NMR (100 MHz, CDCl$_3$) of 3AGa**

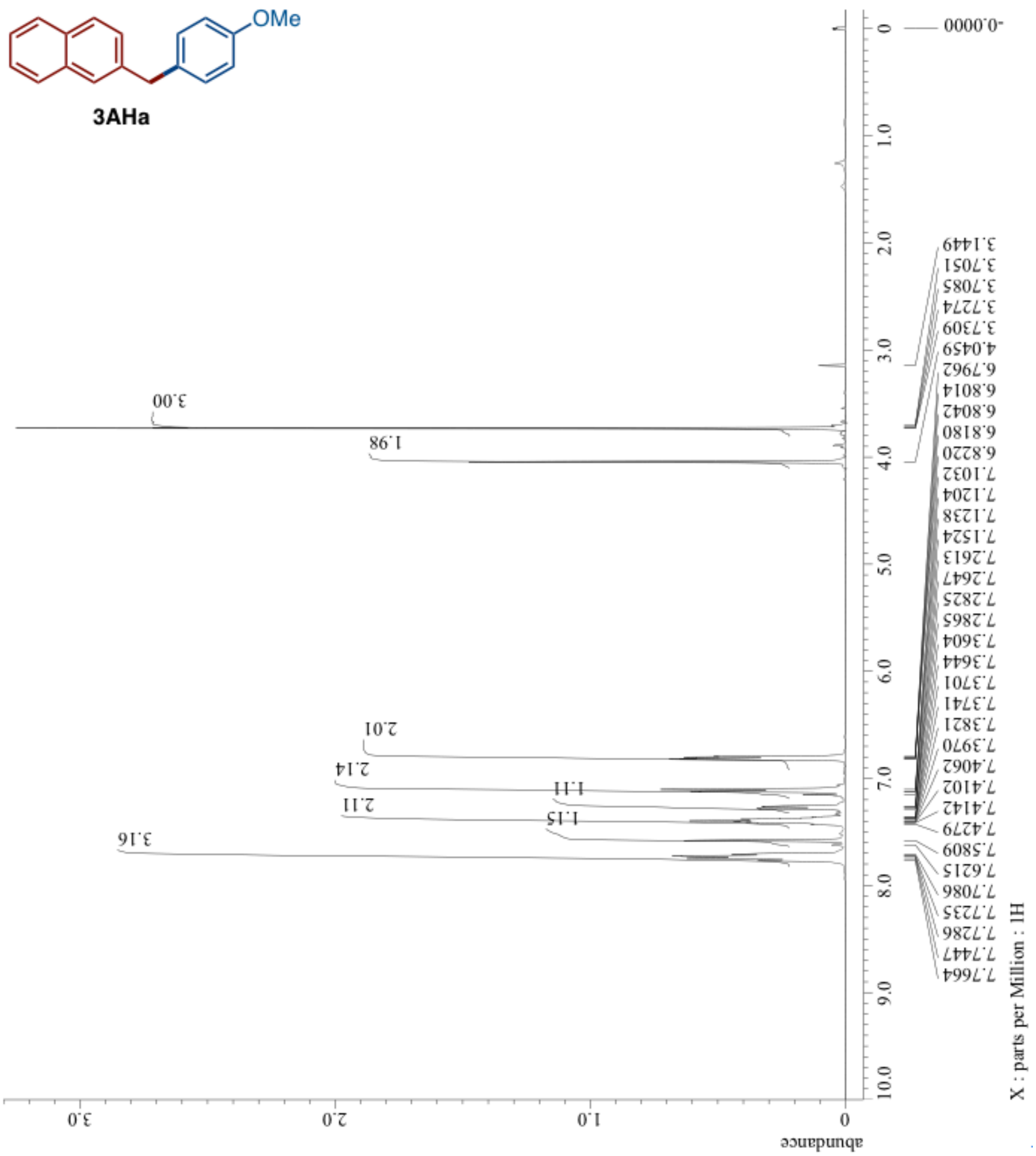


**Supplementary Figure 133. $^{1}$H NMR (400 MHz, $CDCl_3$) of 3AHa**

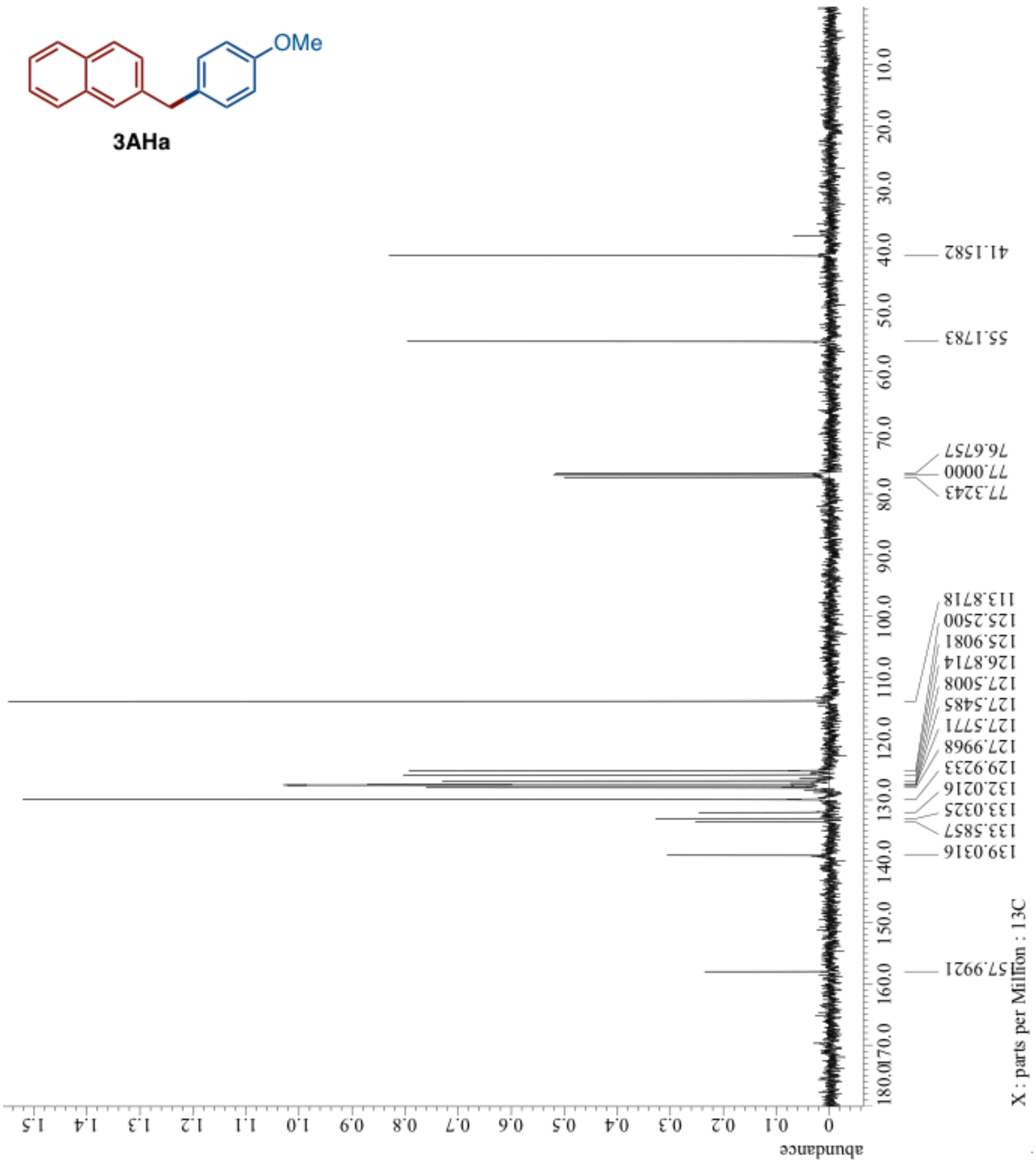


**Supplementary Figure 134.** $^{13}$**C NMR (100 MHz, CDCl$_3$) of 3AHa**

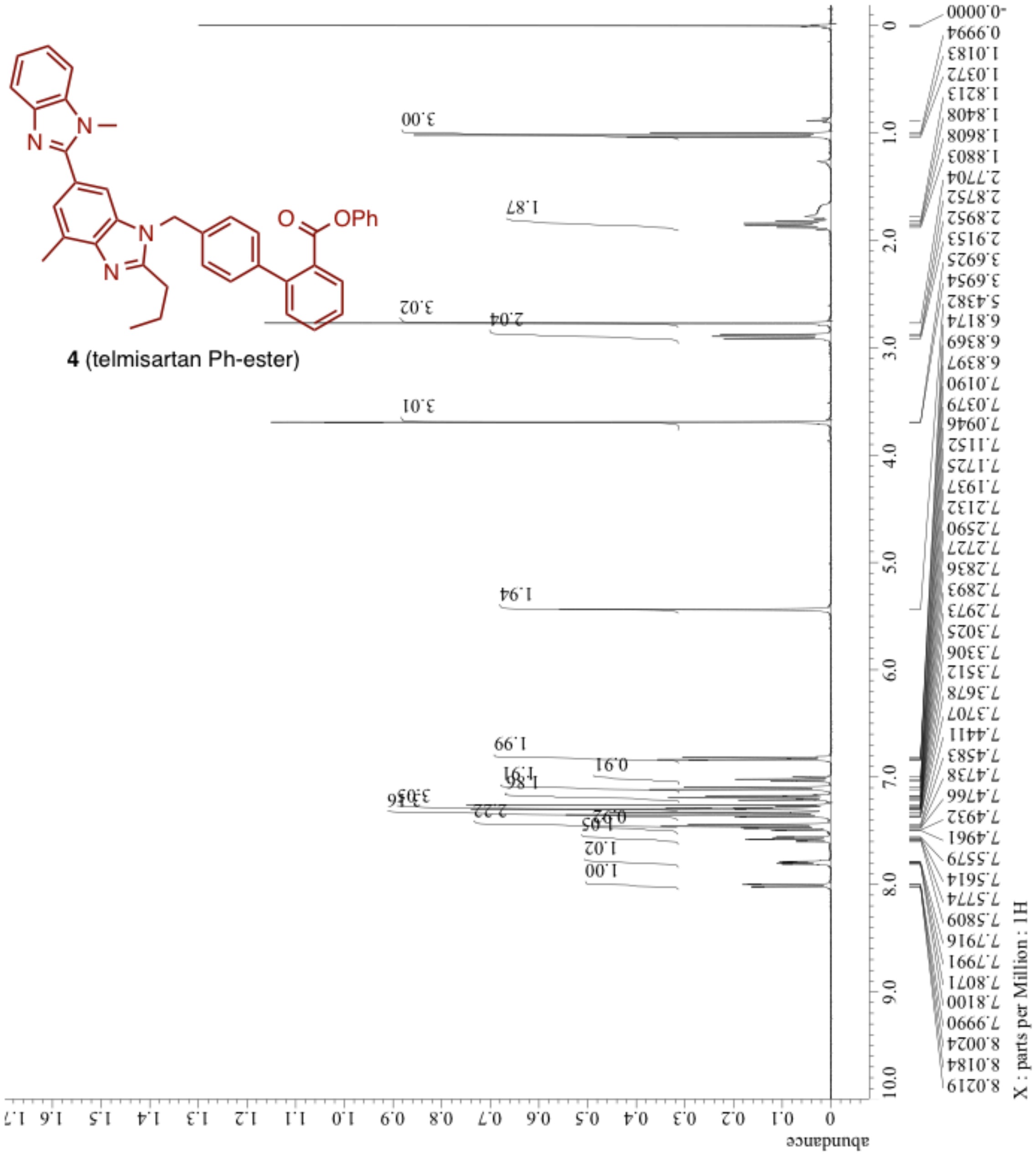


**Supplementary Figure 135.** **$^{1}$H NMR (400 MHz, $CDCl_3$) of 4**

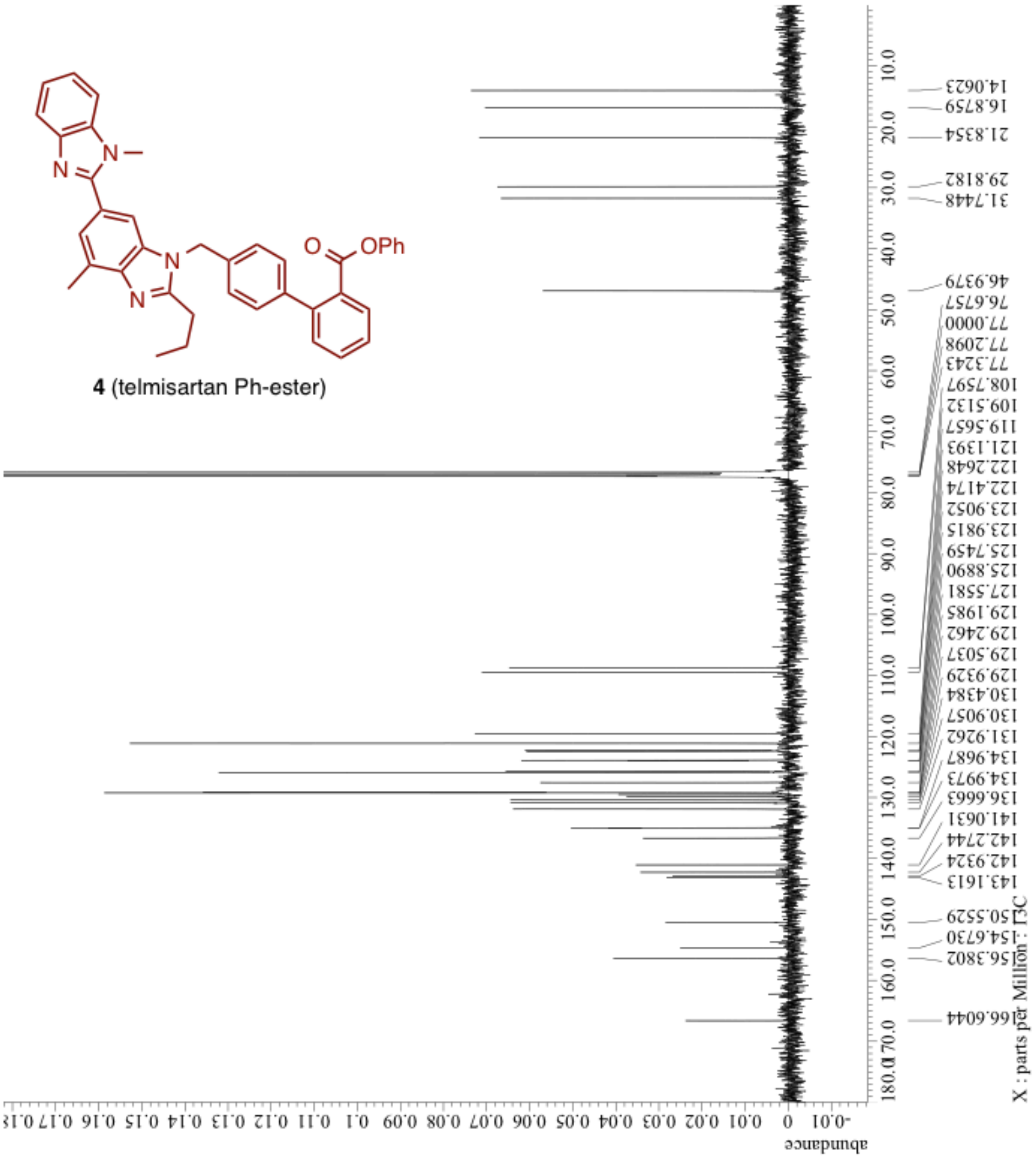


**Supplementary Figure 136.** $^{13}$**C NMR (100 MHz, CDCl$_3$) of 4**

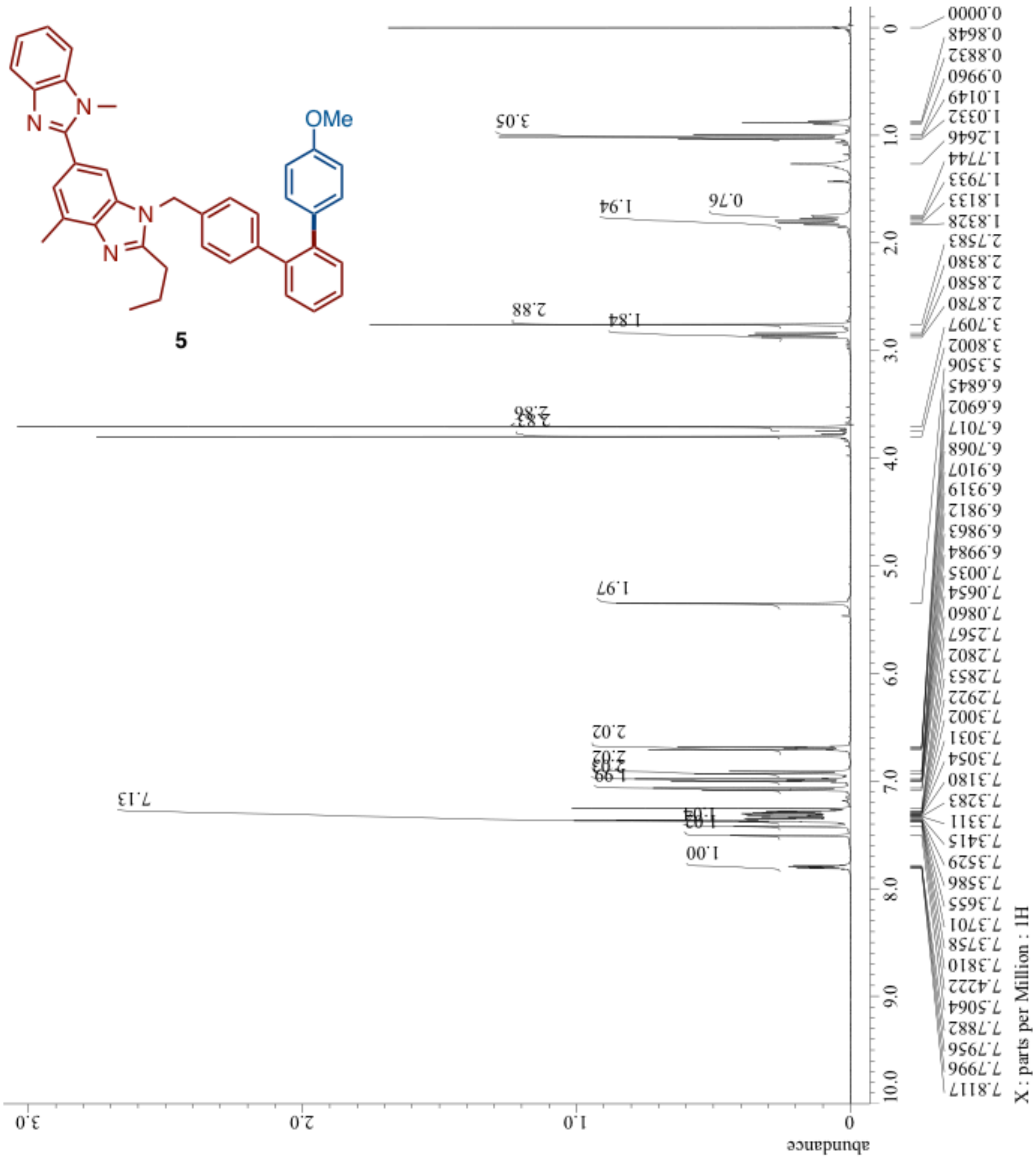


**Supplementary Figure 137. $^{1}$H NMR (400 MHz, $CDCl_3$) of 5**

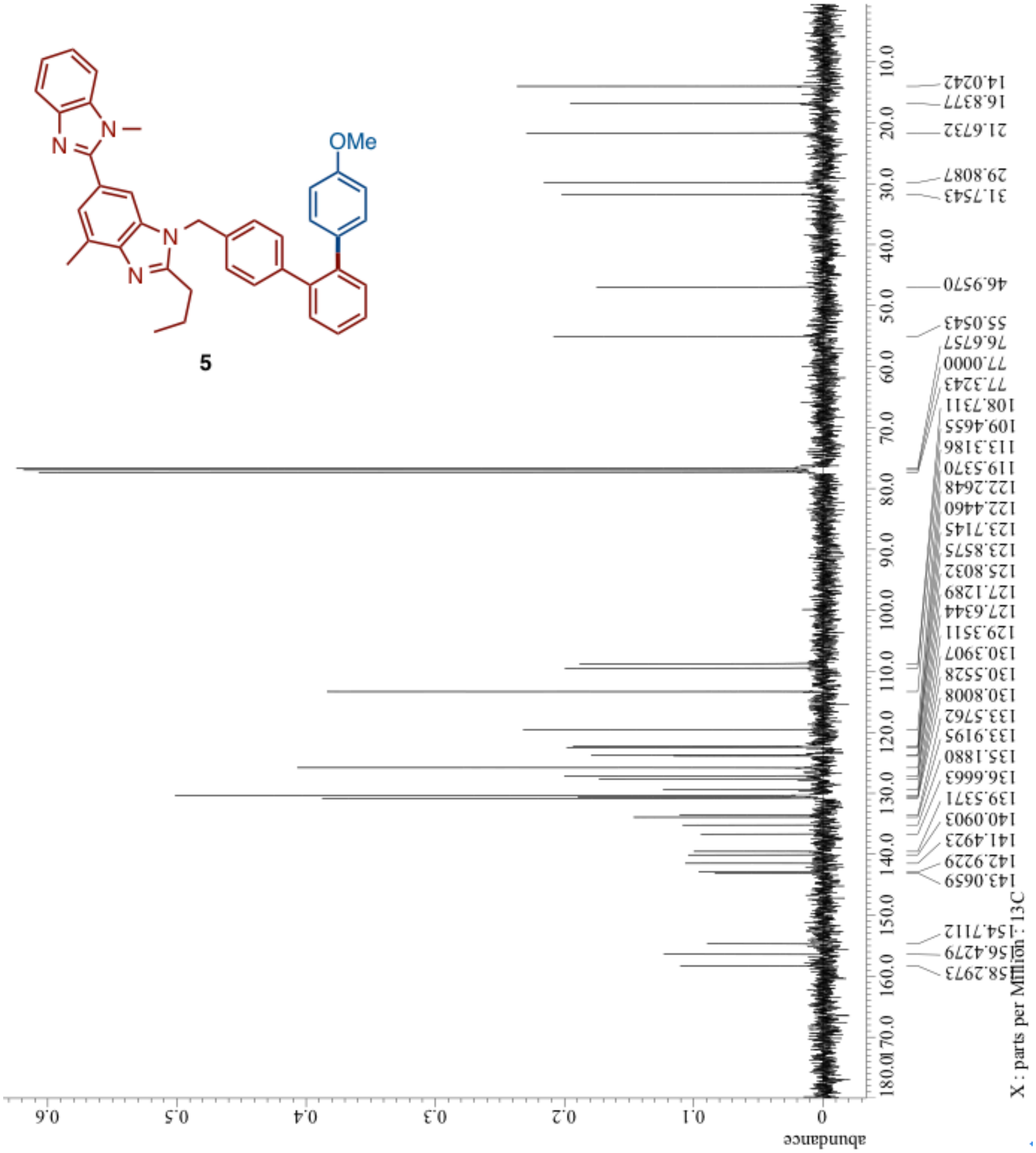


**Supplementary Figure 138.** $^{13}$C NMR (100 MHz, $CDCl_3$) of **5**

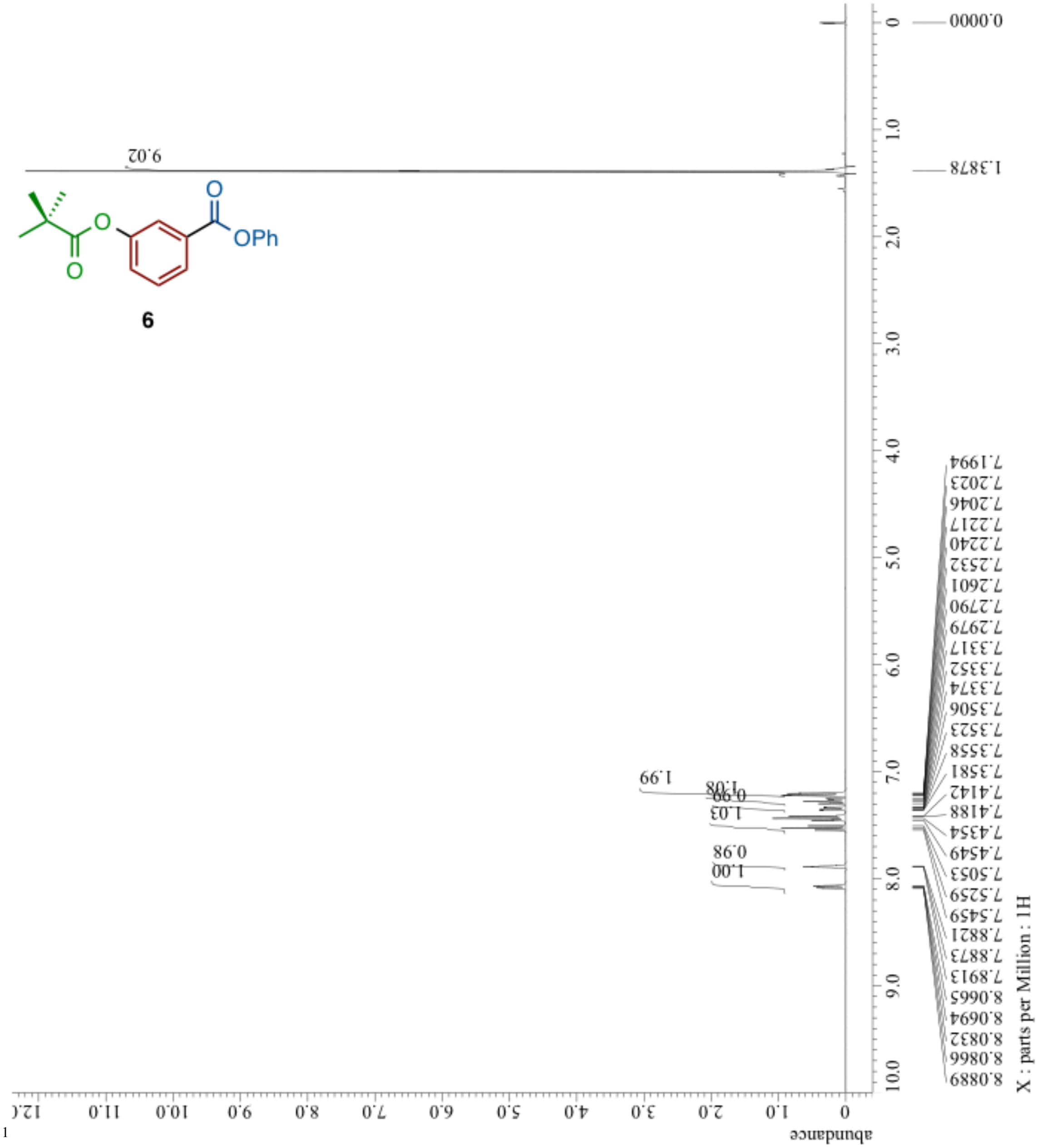


**Supplementary Figure 139. $^{1}$H NMR (400 MHz, $CDCl_3$) of 6**

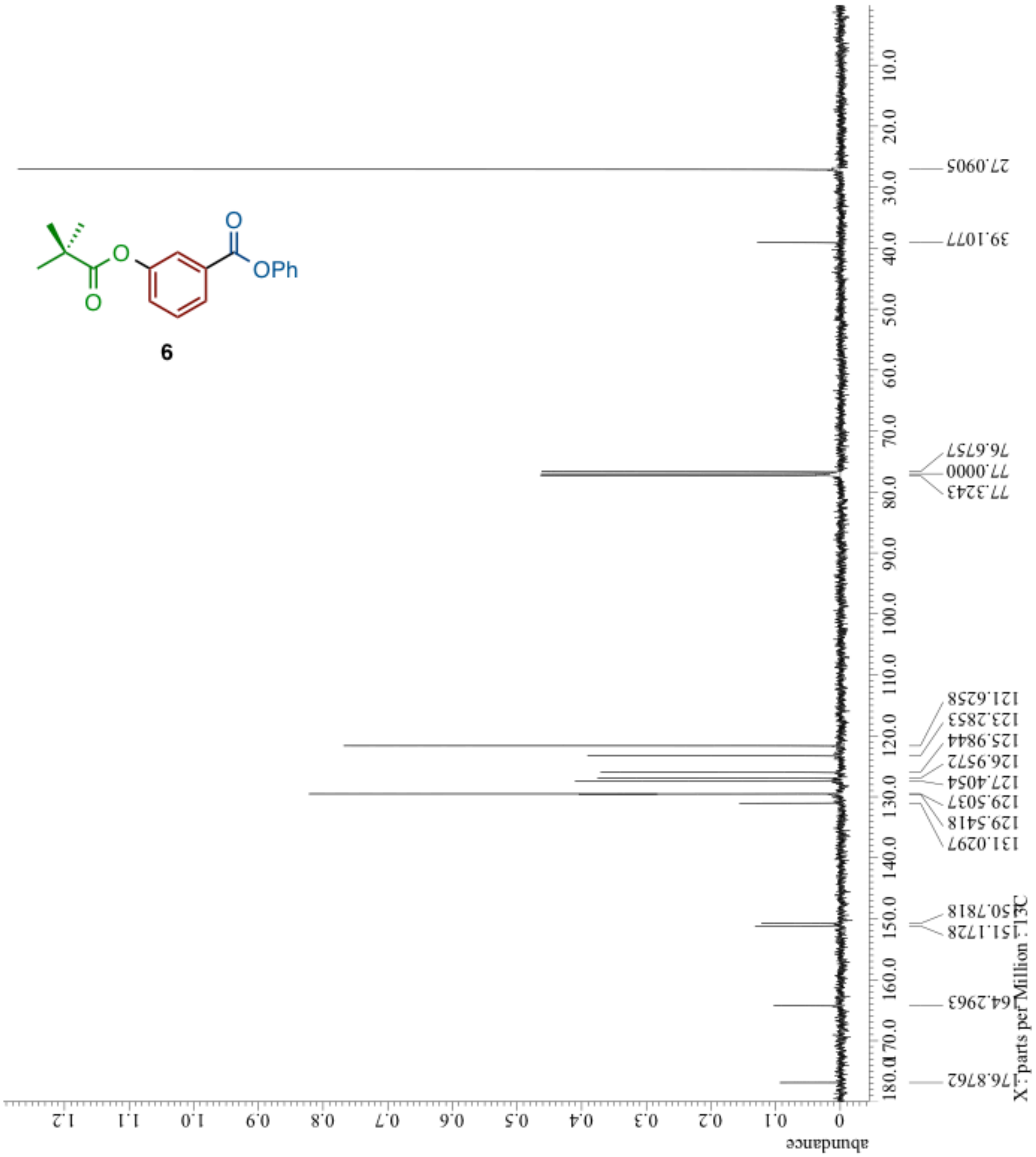


**Supplementary Figure 140.** $^{13}$C NMR (100 MHz, $CDCl_3$) of 6

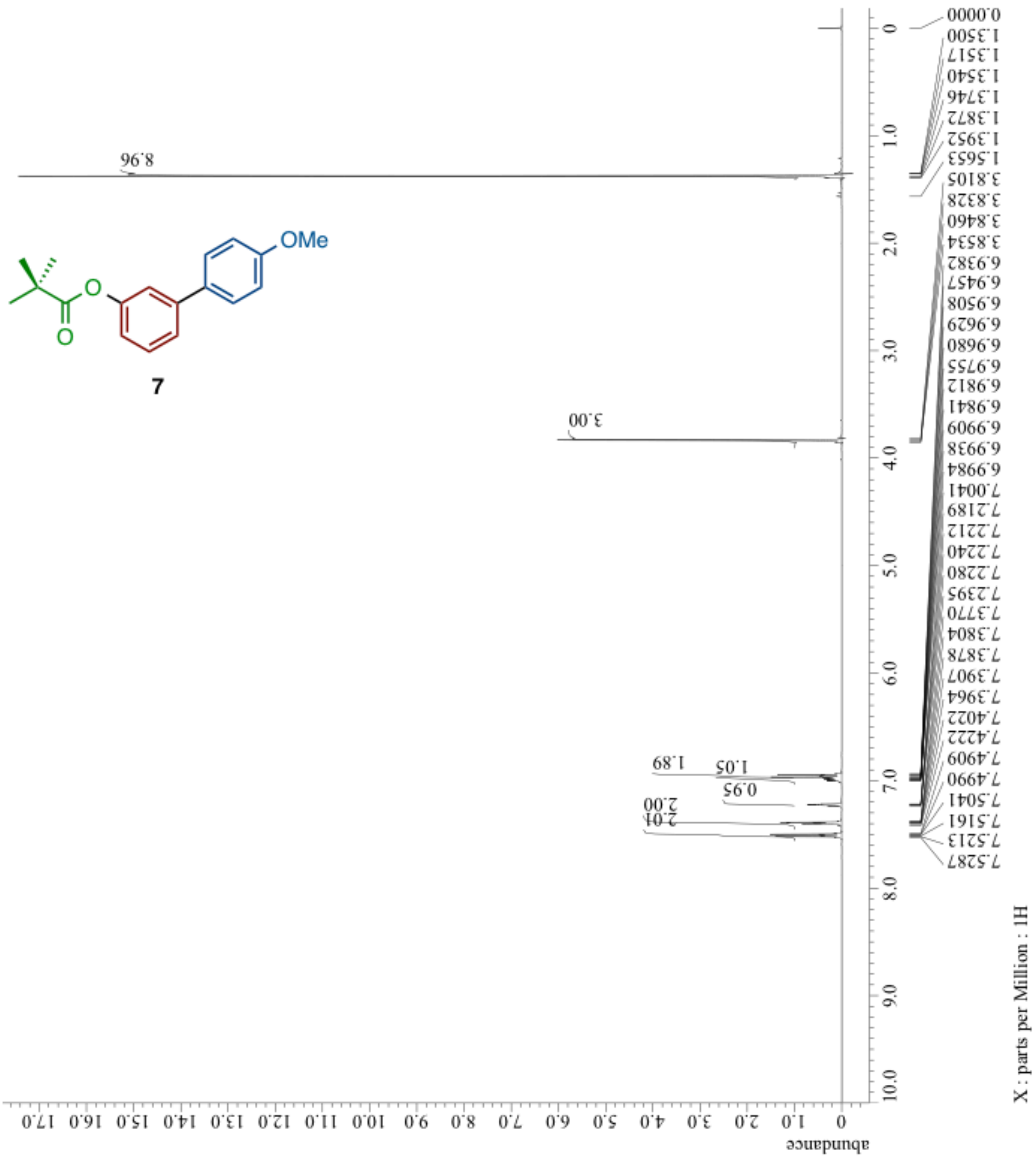


**Supplementary Figure 141.** $^{1}$**H NMR (400 MHz, CDCl$_3$) of 7**

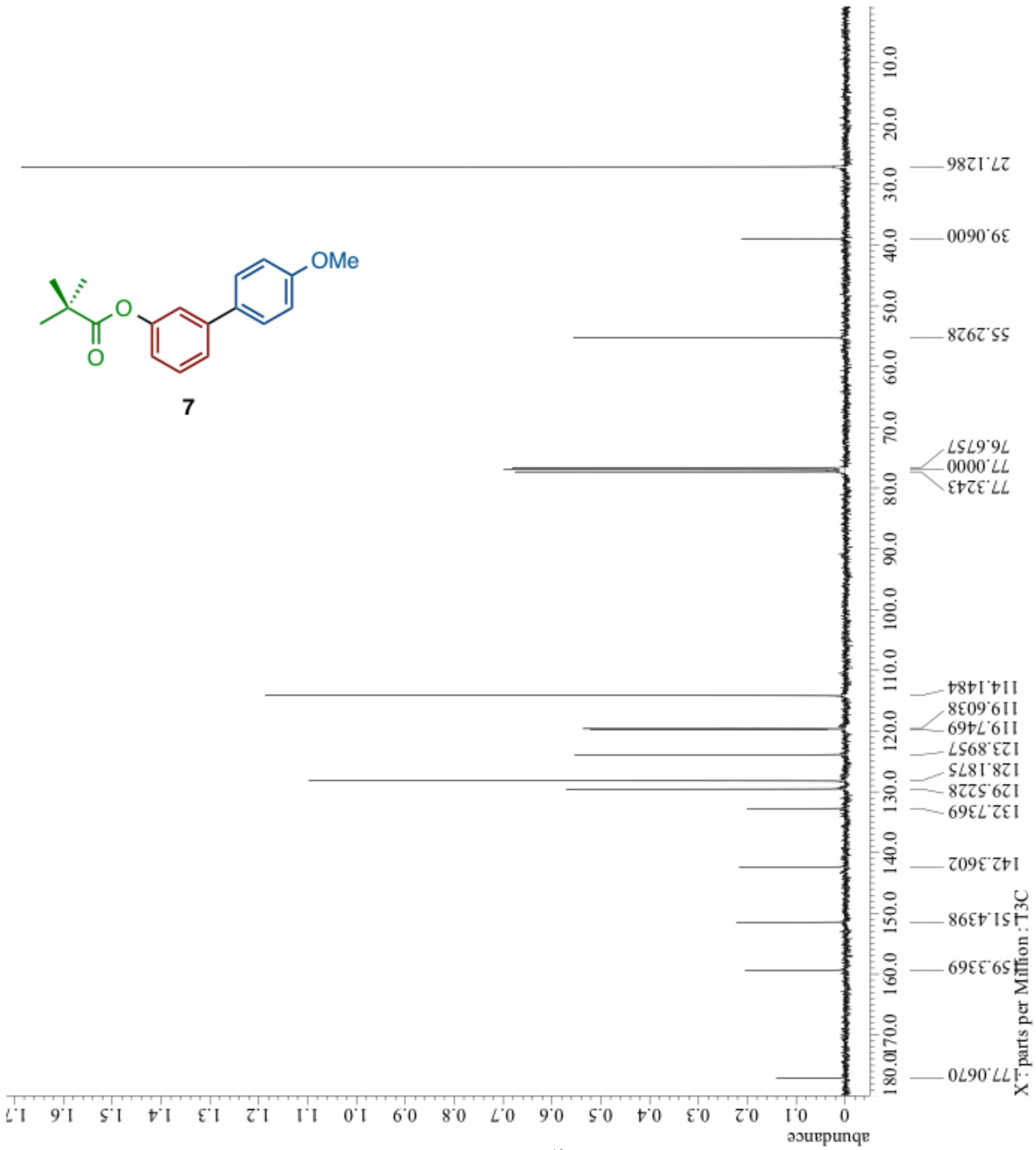


**Supplementary Figure 142.** $^{13}C$ **NMR (100 MHz,** $CDCl_3$**) of 7**

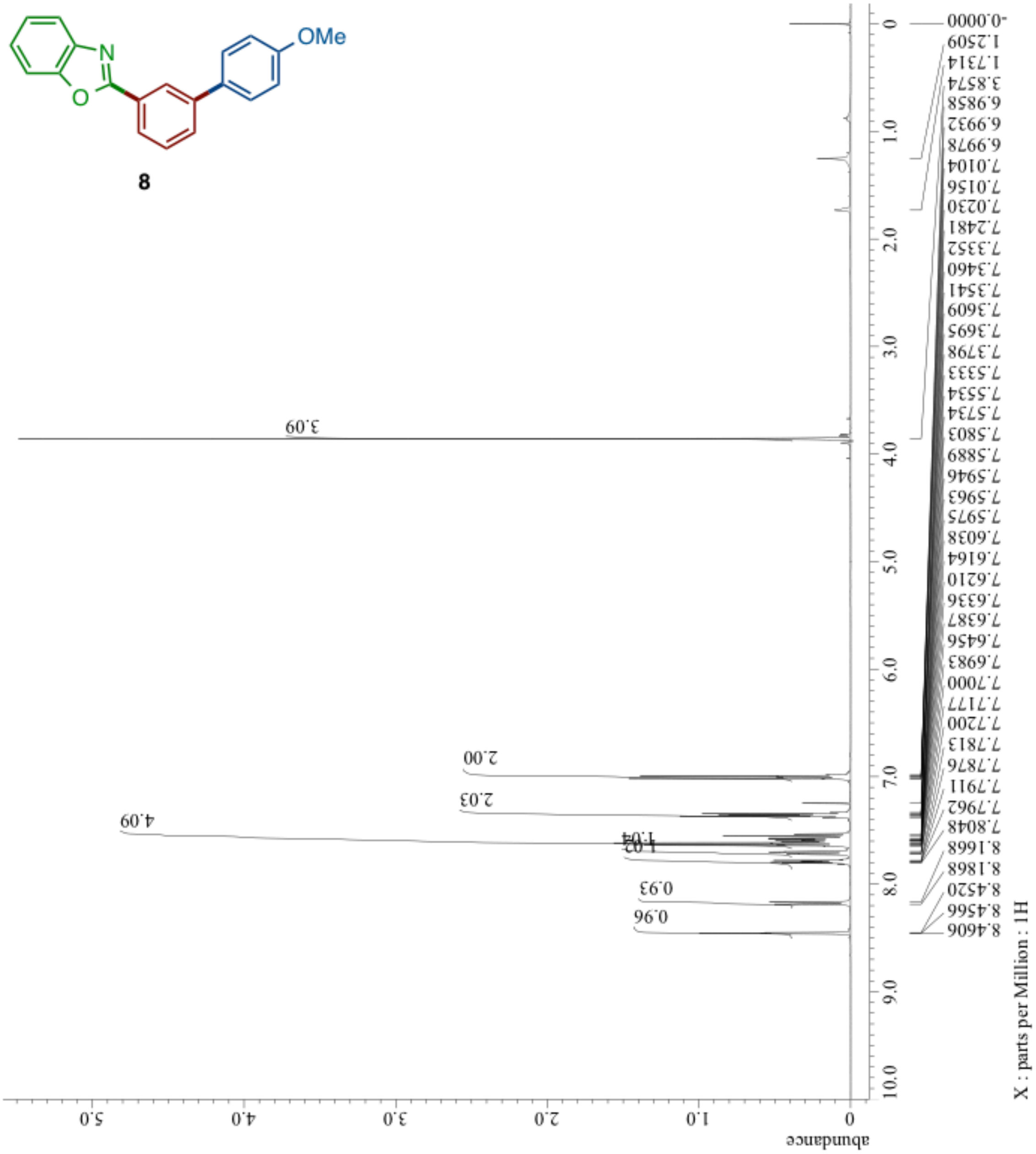


**Supplementary Figure 143.** $^{1}$**H NMR (400 MHz, CDCl$_3$) of 8**

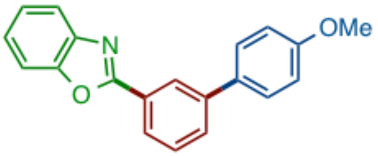


8

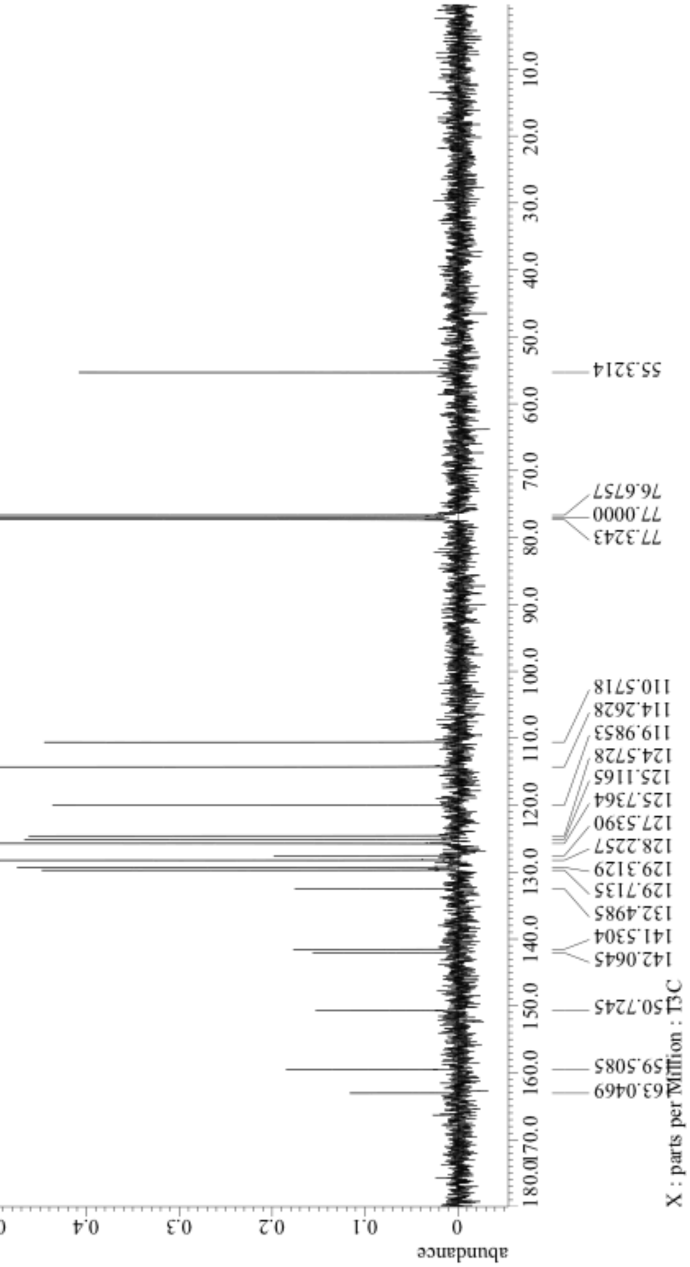

**Supplementary Figure 144.** $^{13}$**C NMR (100 MHz, CDCl$_3$) of 8**

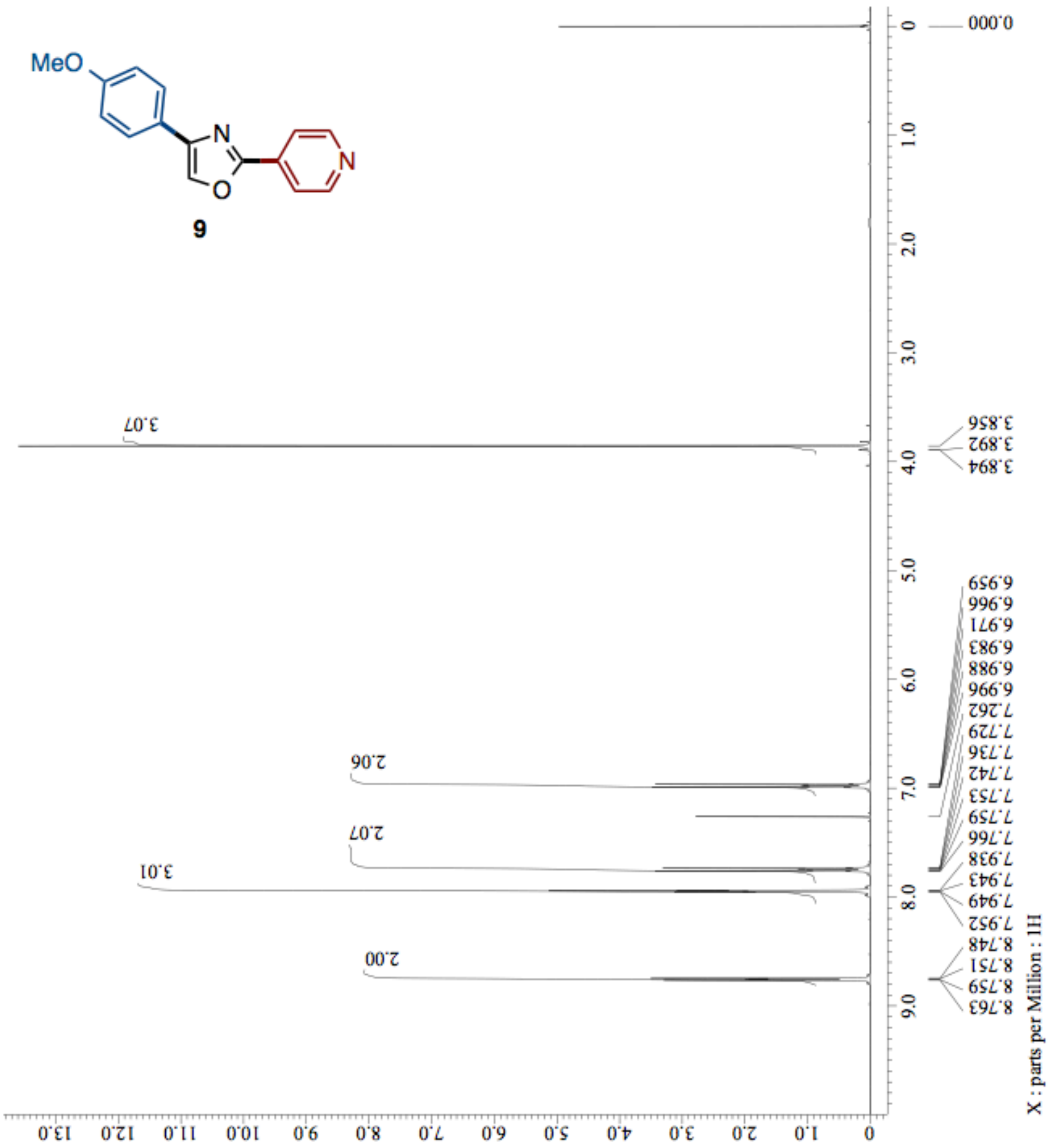


**Supplementary Figure 145.** $^1$**H NMR (400 MHz,** $CDCl_3$**) of 9**

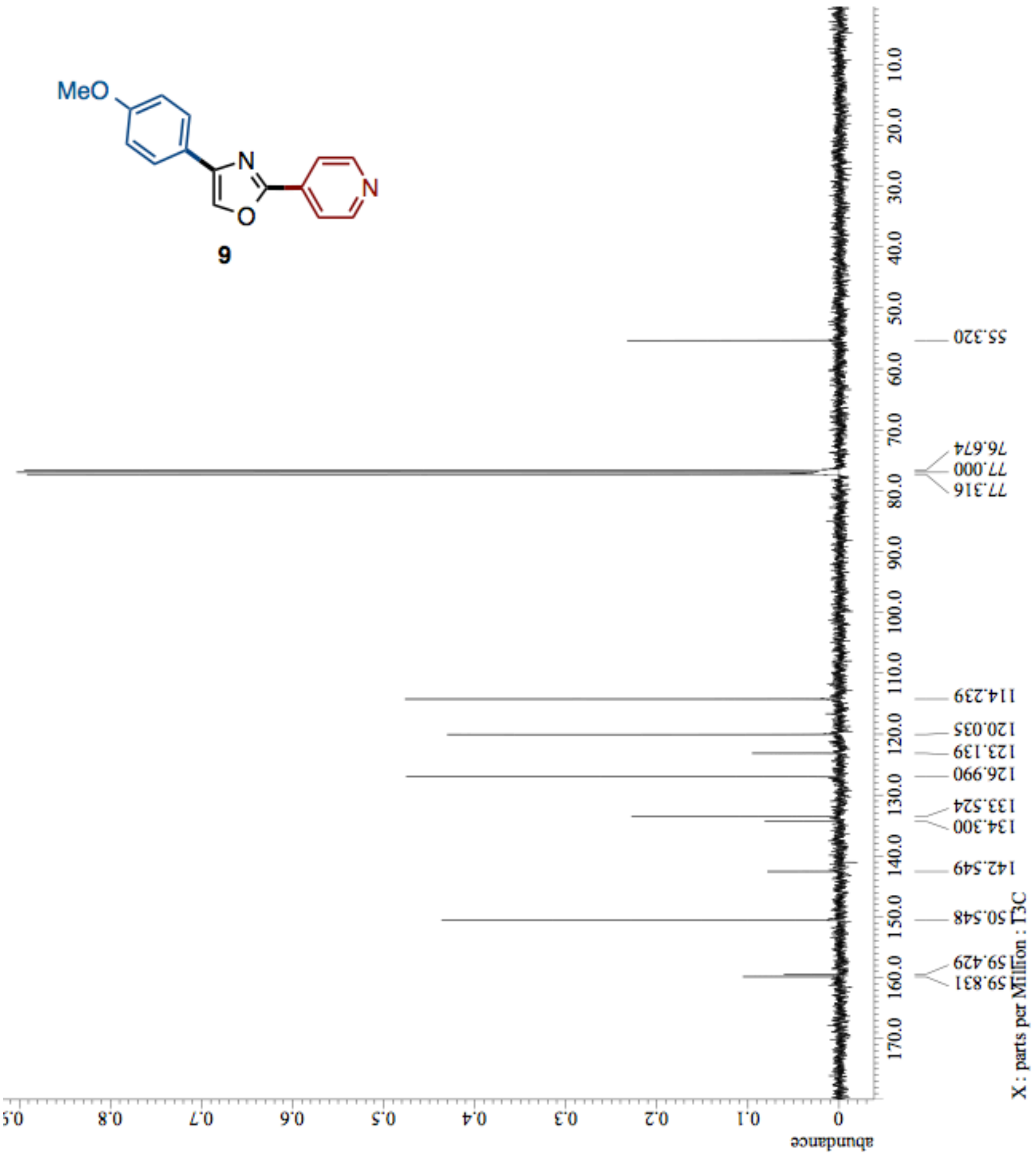


**Supplementary Figure 146.** $^{13}$**C NMR (100 MHz, CDCl$_3$) of 9**

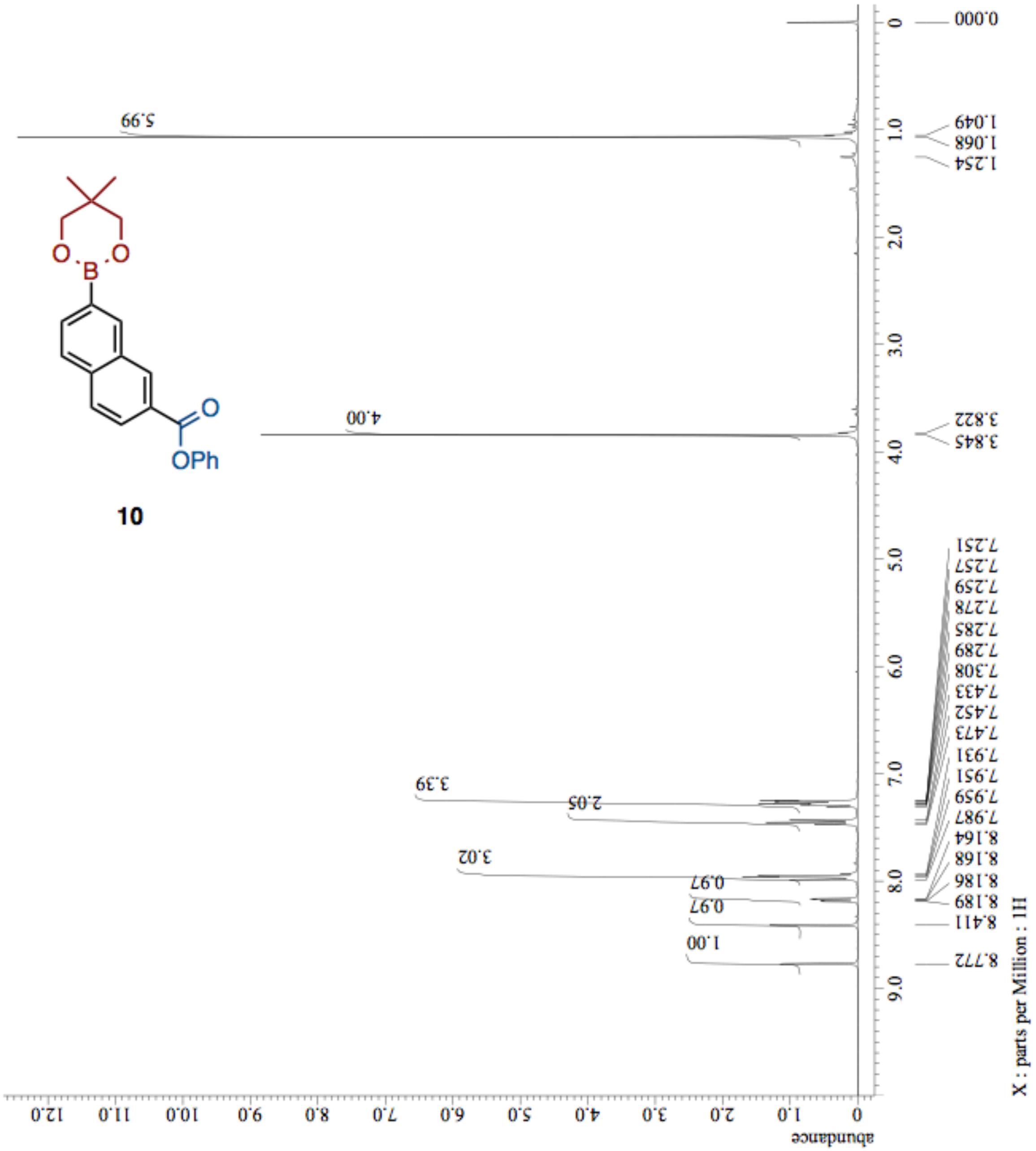


**Supplementary Figure 147. $^1$H NMR (400 MHz, $CDCl_3$) of 10**

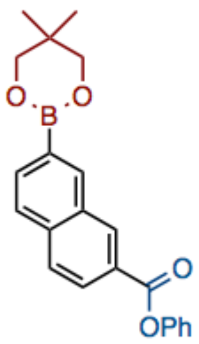


**10**

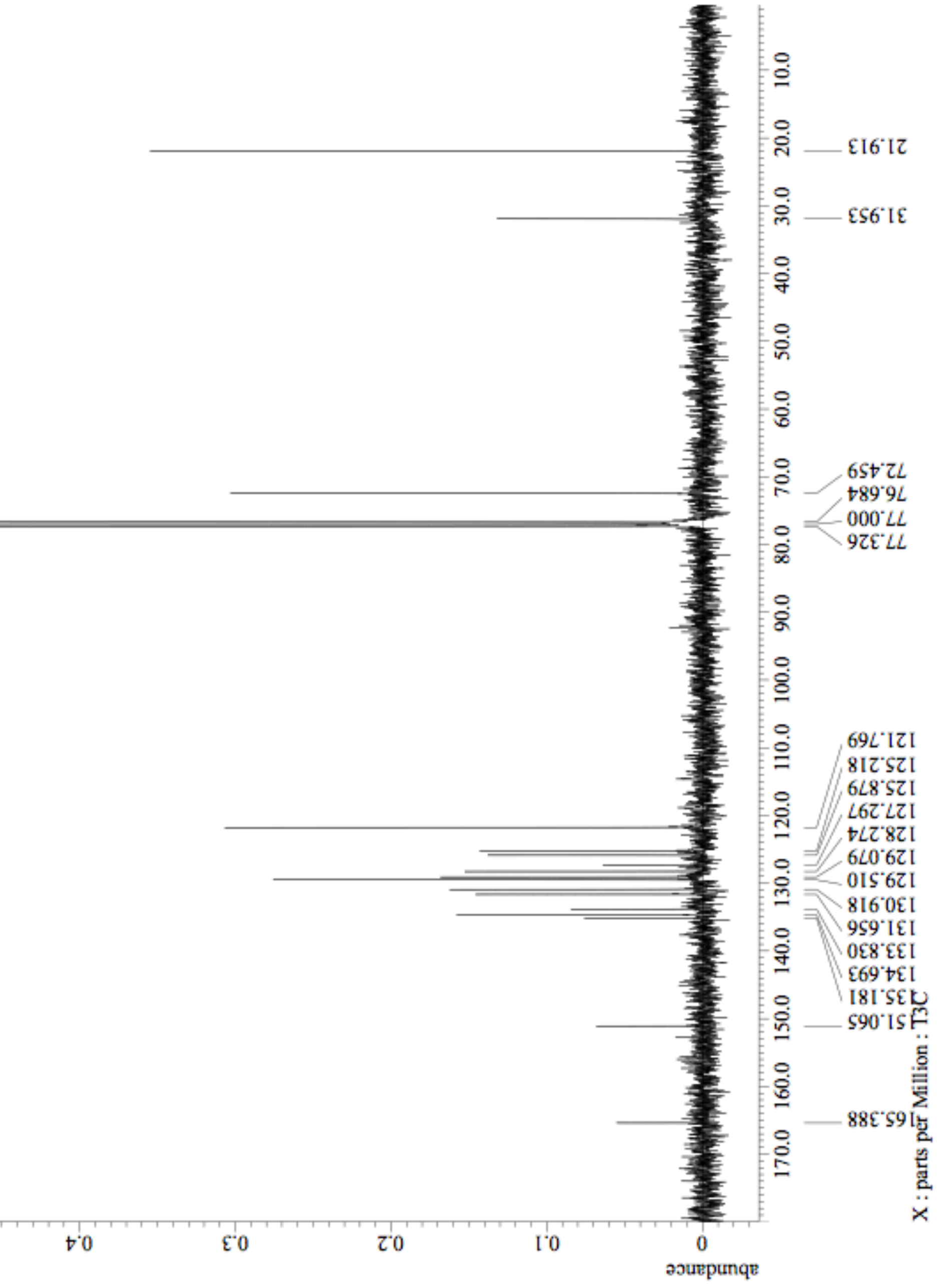


**Supplementary Figure 148. $^{13}$C NMR (100 MHz, $CDCl_3$) of 10**

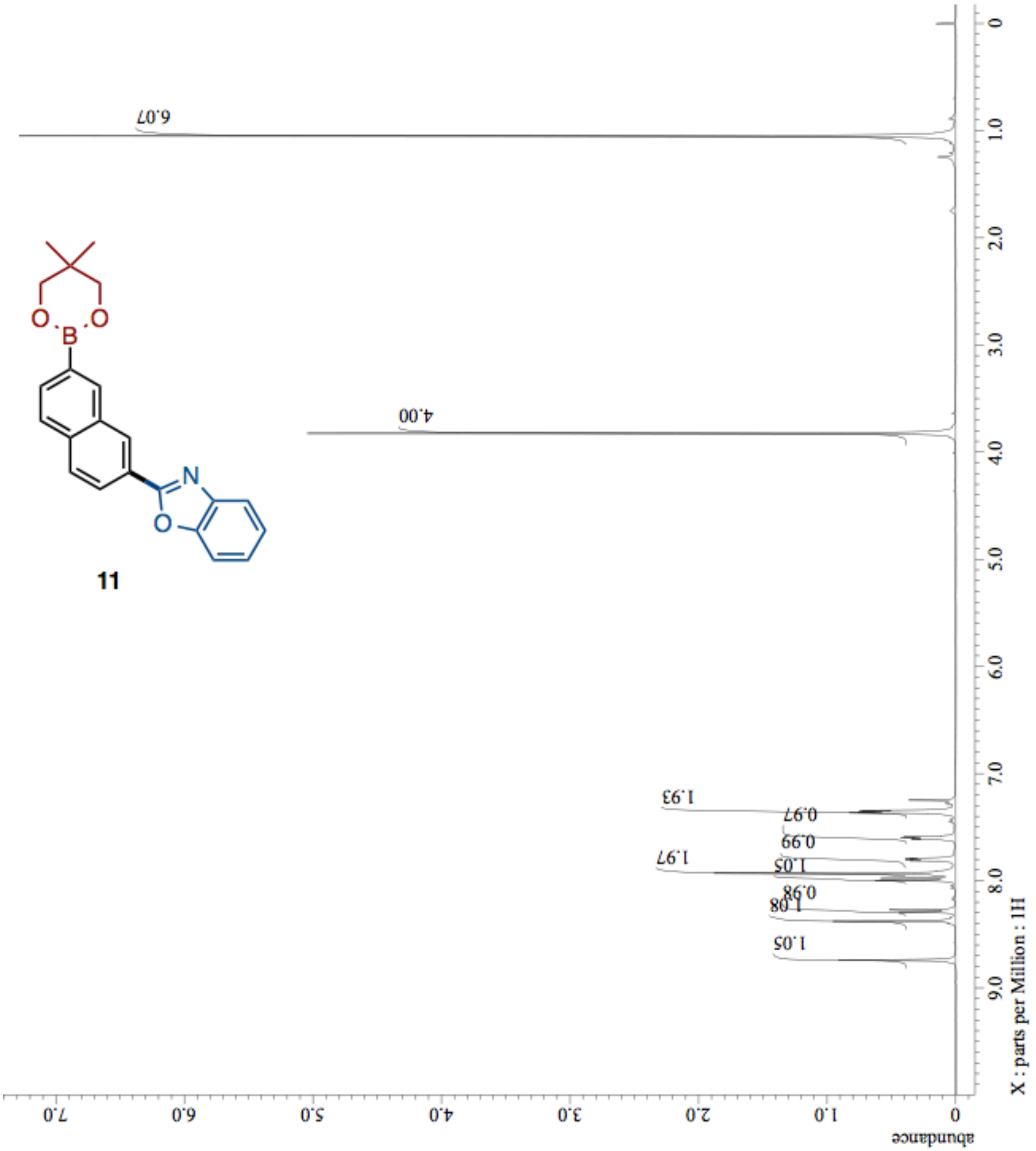


**Supplementary Figure 149. $^{1}H$ NMR (400 MHz, $CDCl_3$) of 11**

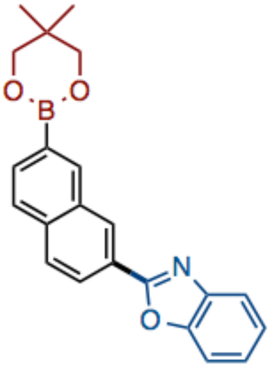

**11**

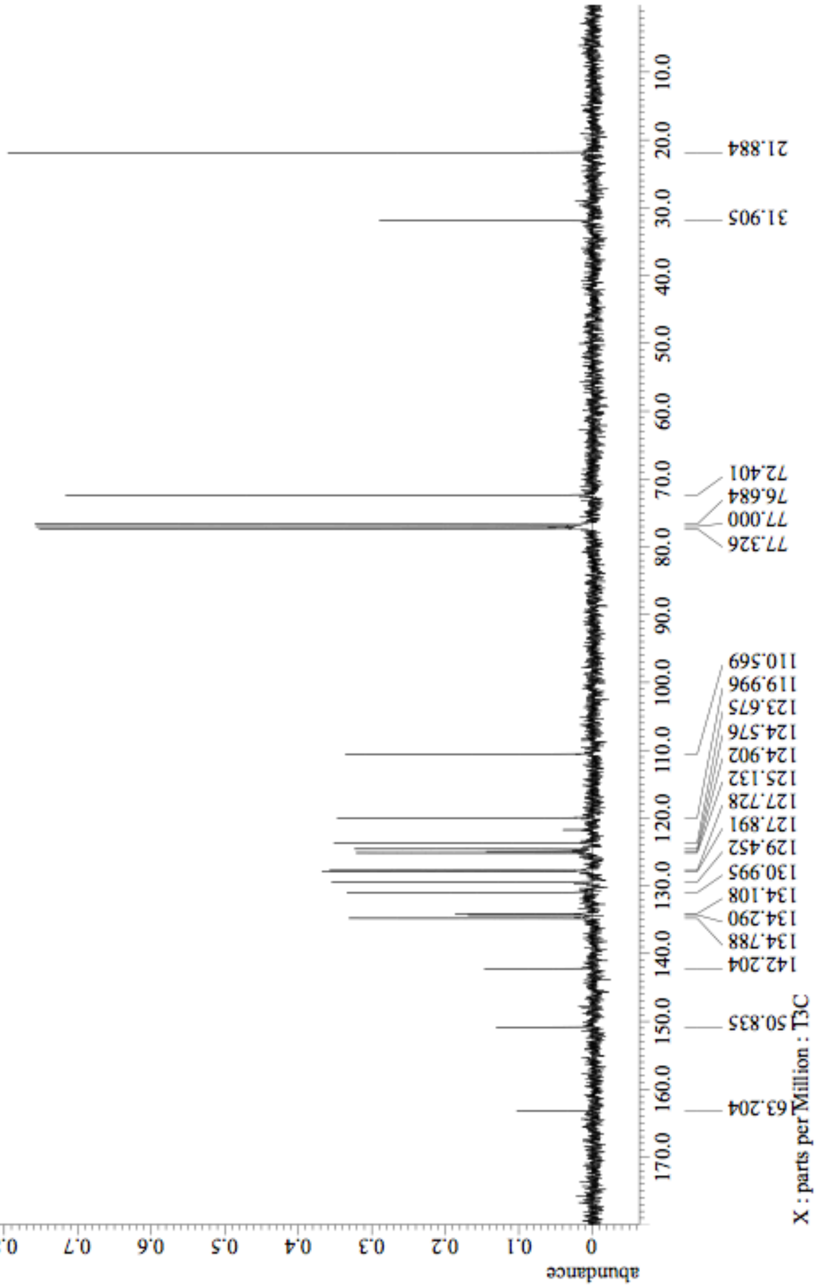


**Supplementary Figure 150.** $^{13}$**C NMR (100 MHz,** $CDCl_3$**) of 11**

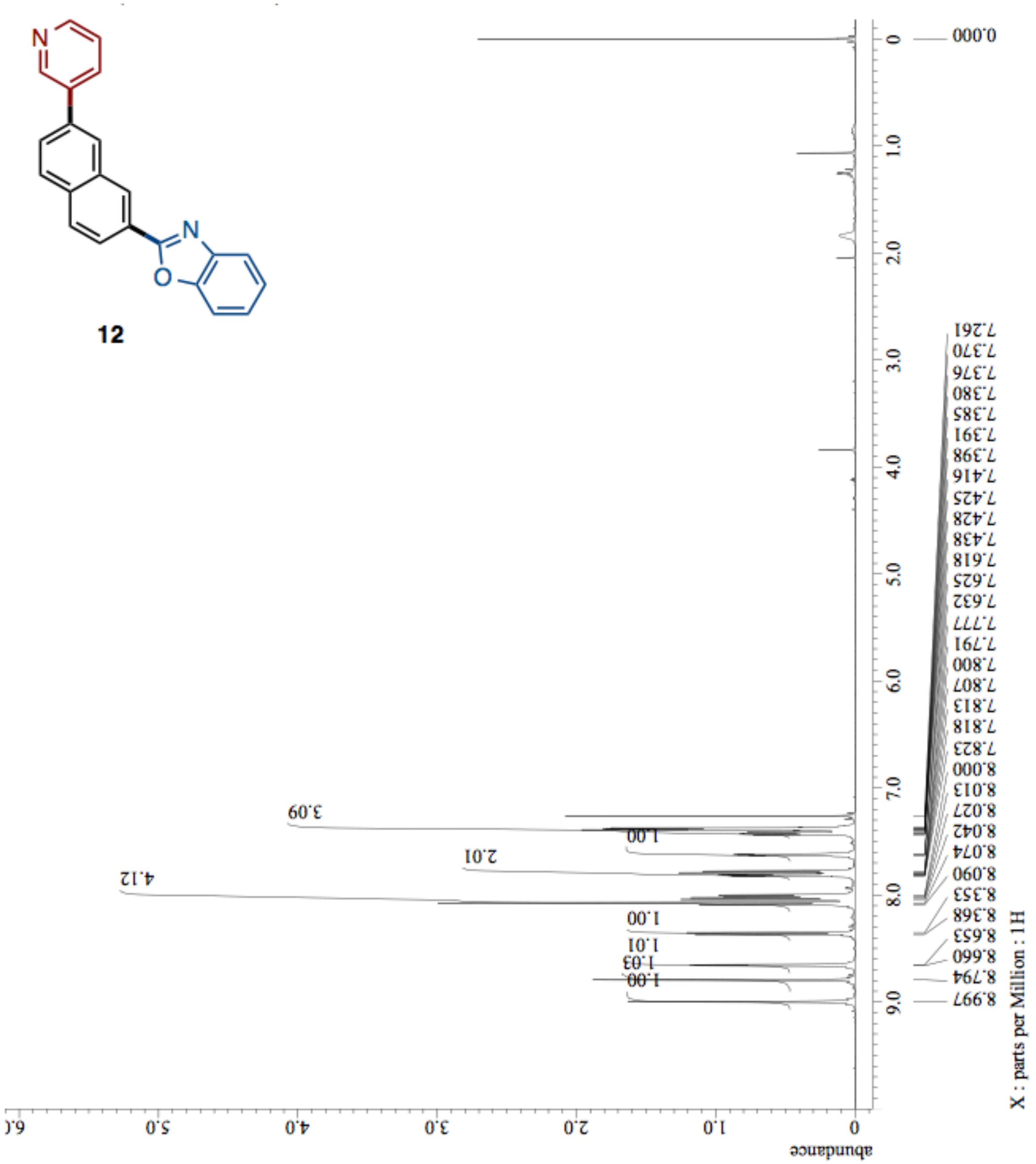


**Supplementary Figure 151.** $^1$**H NMR (600 MHz, CDCl$_3$) of 12**

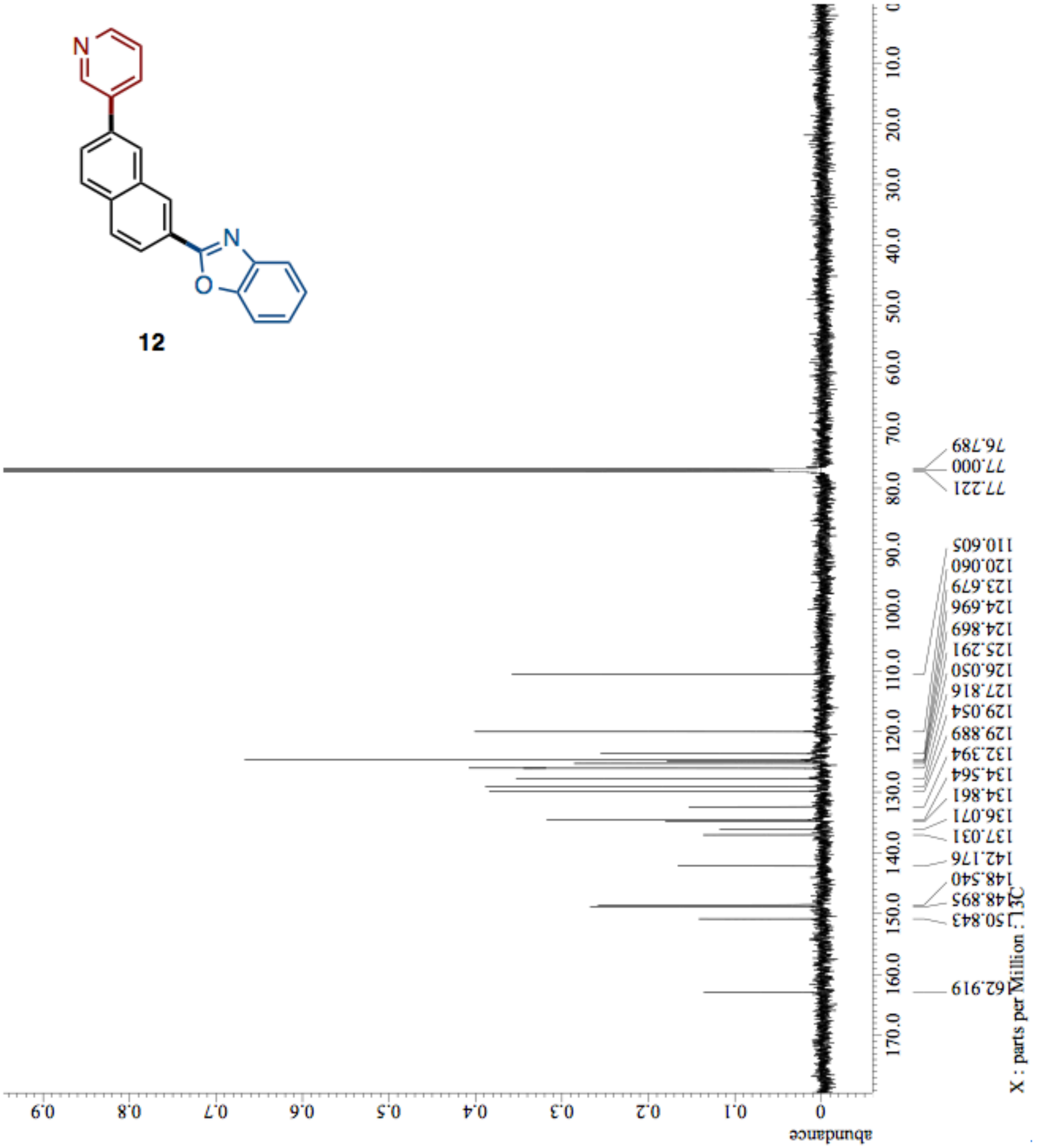


**Supplementary Figure 152.** $^{13}$**C NMR (150 MHz,** $CDCl_3$**) of 12**

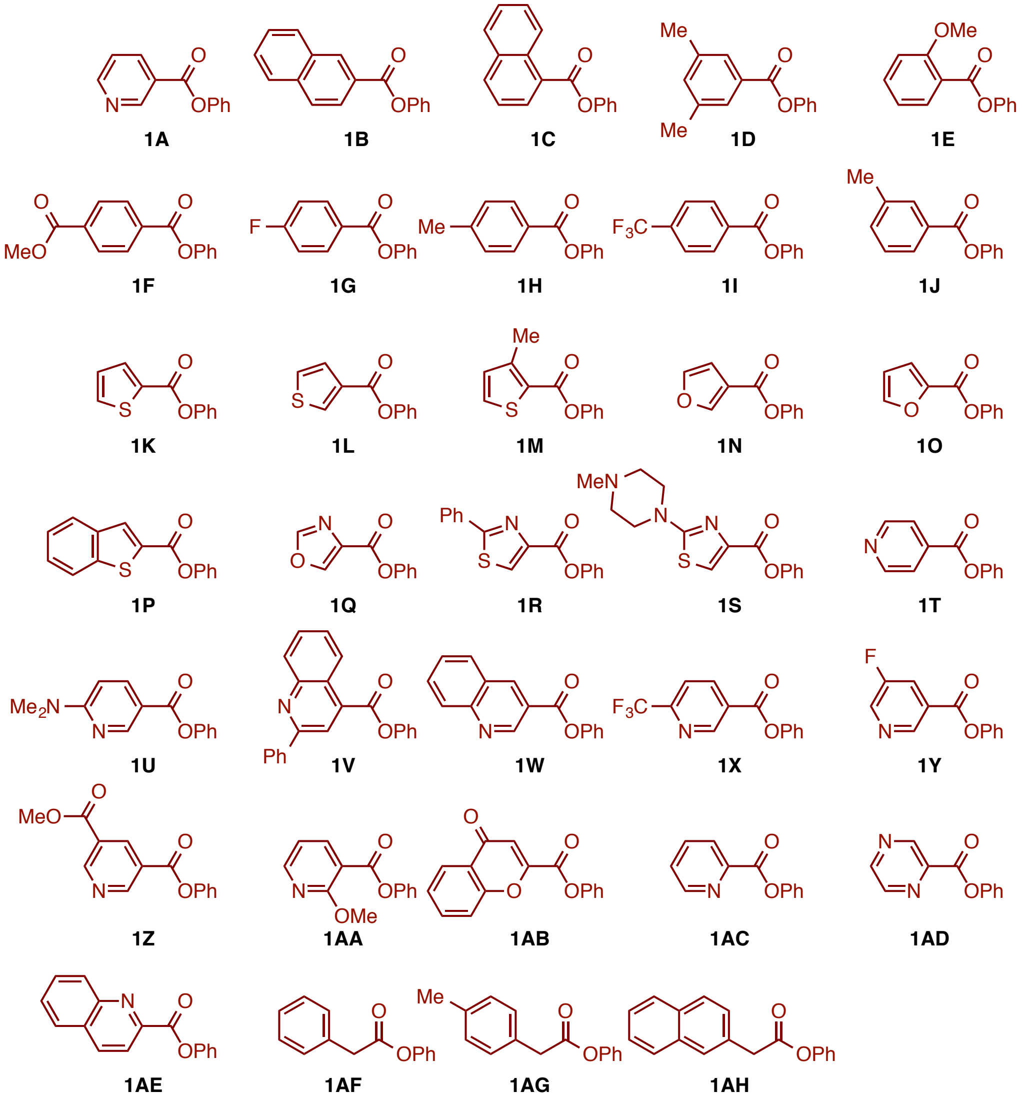


**Supplementary Figure 153.** Substrate structure of carboxylic acid esters **1**

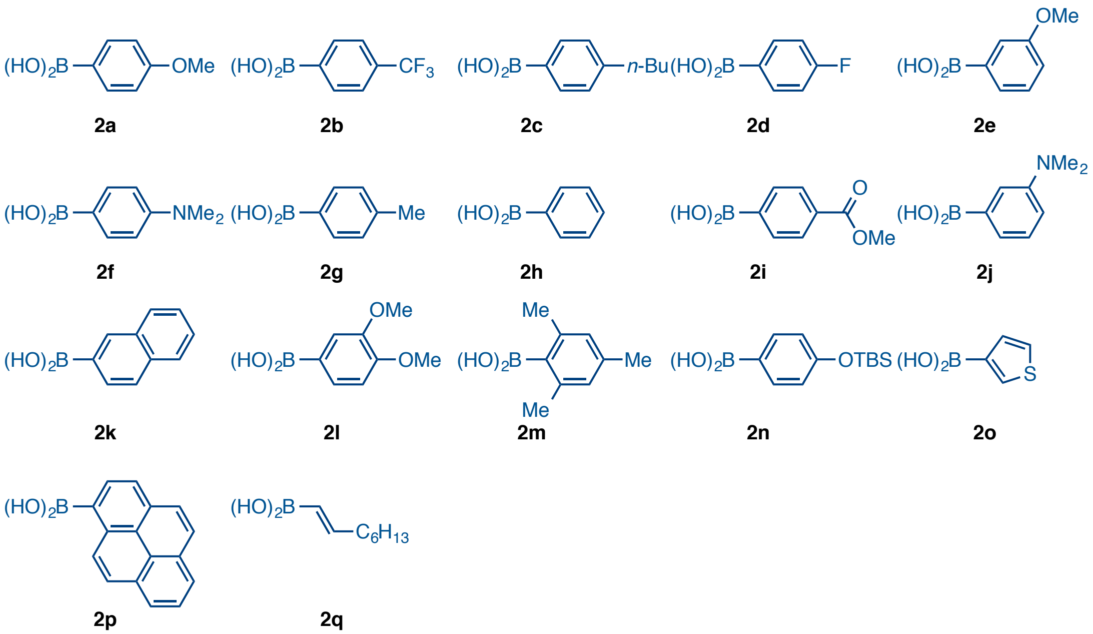


**Supplementary Figure 154.** Substrate structure of boronic acids **2**

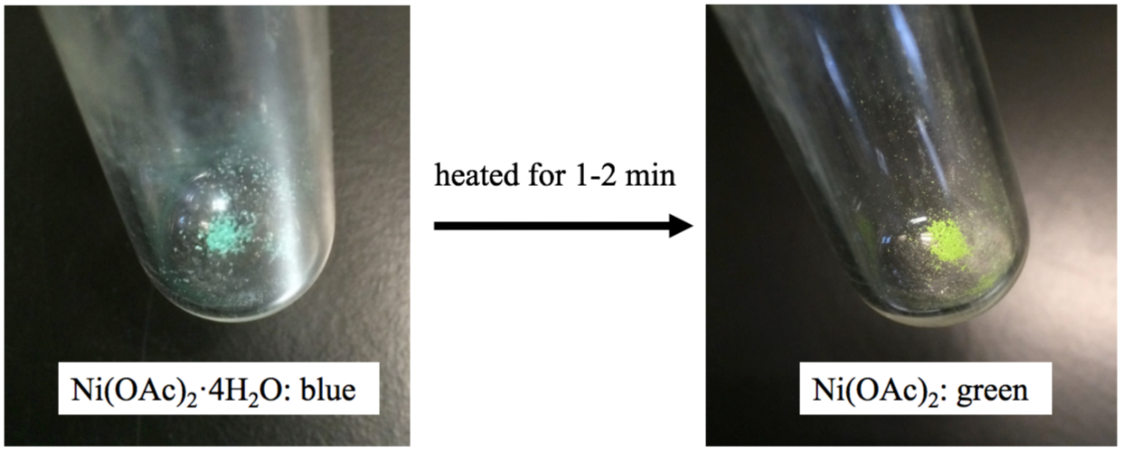


**Supplementary Figure 155.** How the color of the nickel salt changes

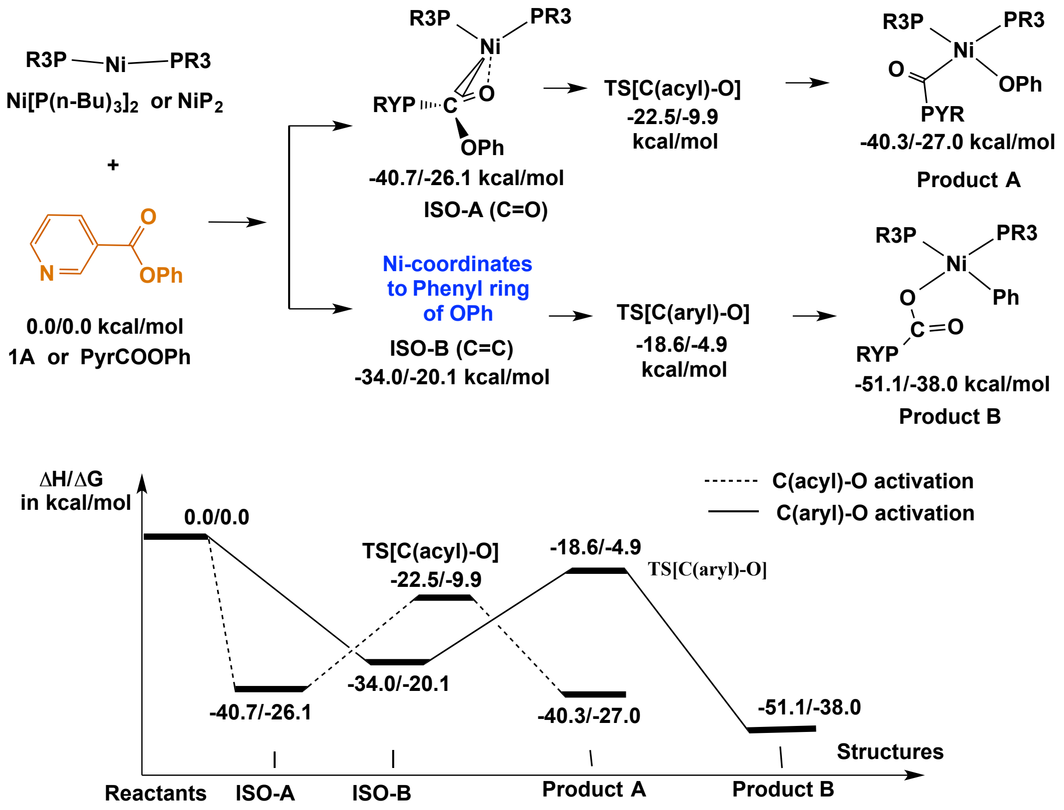


**Supplementary Figure 156.** Oxidative Addition of Phenyl 3-Pyridinecarboxylate to Ni[P(*n*-Bu)$_3$]$_2$ Catalyst. Schematic presentation of possible reactants, intermediates, transition states and products of the C(acyl)-O and C(aryl)-O oxidative addition of phenyl 3-pyridinecarboxylate to Ni[P(*n*-Bu)$_3$]$_2$ in the no-base condition and at the M06L/BS1 level of theory. For structural parameters, see Cartesian coordinates given in Supplementary Table 13. For total energies, see Supplementary Table 8.

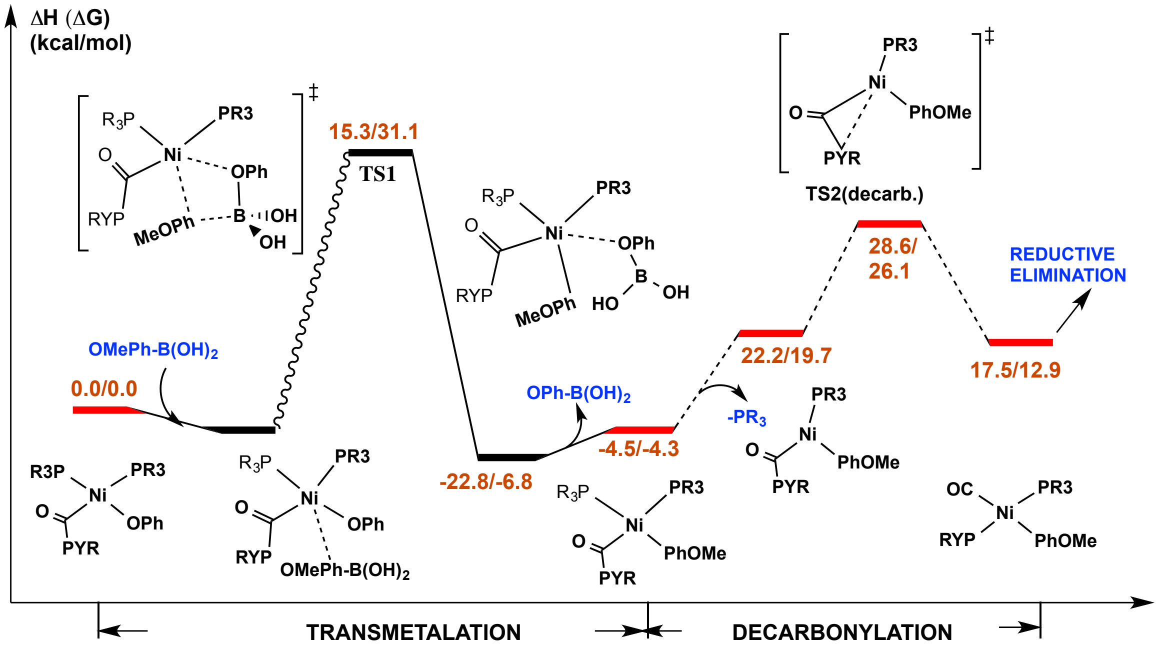


**Supplementary Figure 157.** Schematic presentation of the favorable transmetalation-then-decarbonylation pathway of the phenyl 3-pyridinecarboxylate and boronic acid cross-coupling catalyzed by Ni[P($n$-Bu)$_3$]$_2$ complex in the no-base condition. Presented energies are obtained at the M06L/BS1 level of theory.

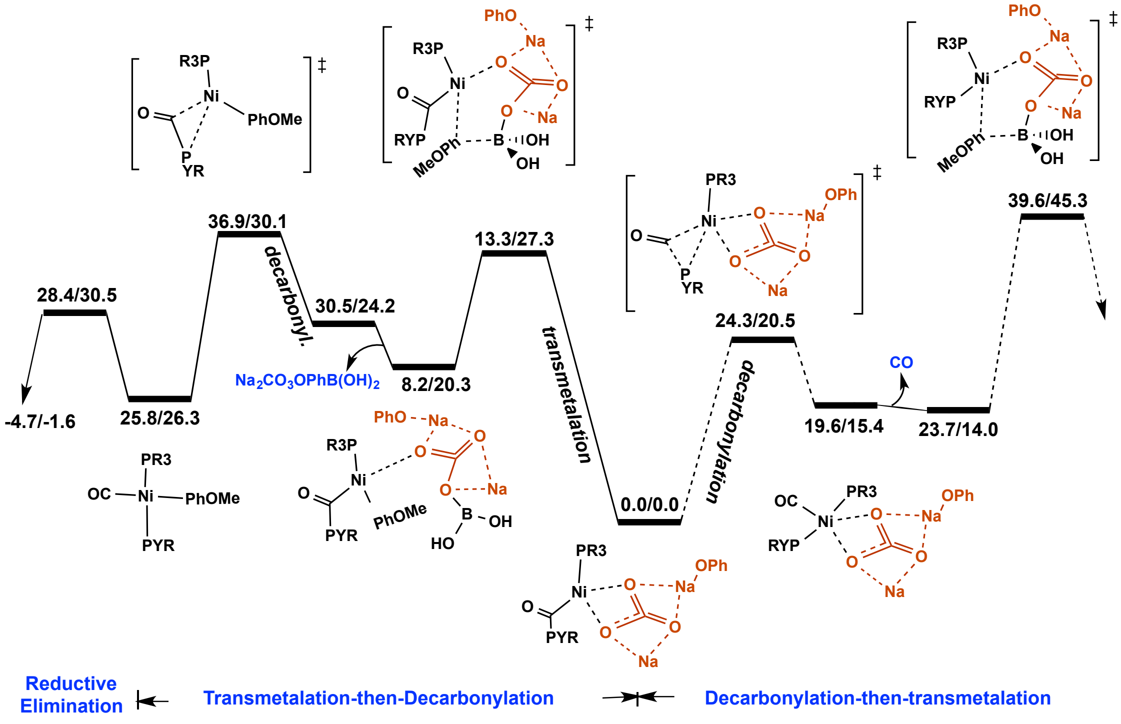


**Supplementary Figure 158.** Schematic presentation of the favorable transmetalation-then-decarbonylation pathway of the phenyl 3-pyridinecarboxylate and boronic acid cross-coupling catalyzed by Ni[P($n$-Bu)$_3$]$_2$ complex in the presence of $Na_2CO_3$ condition. Presented energies are obtained at the M06L/BS1 level of theory.

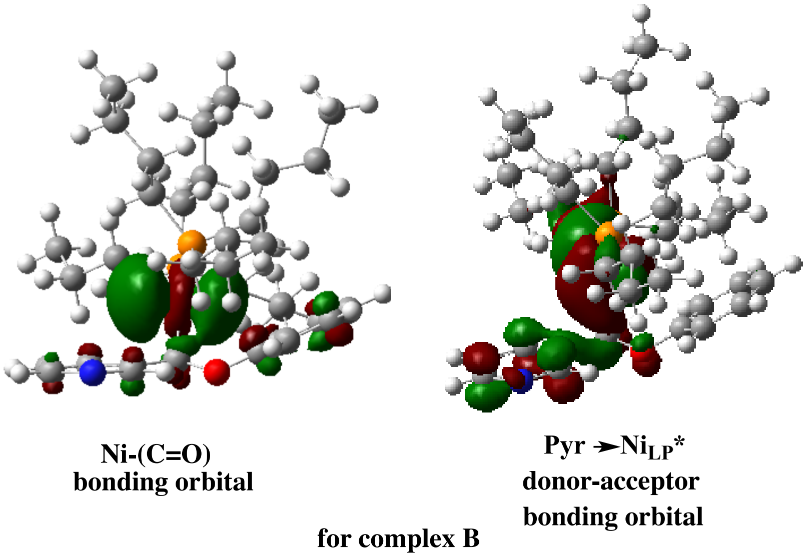


**Supplementary Figure 159.** Important NBO orbitals of the complex B

**Supplementary Table 1.** Investigation of the reaction parameters

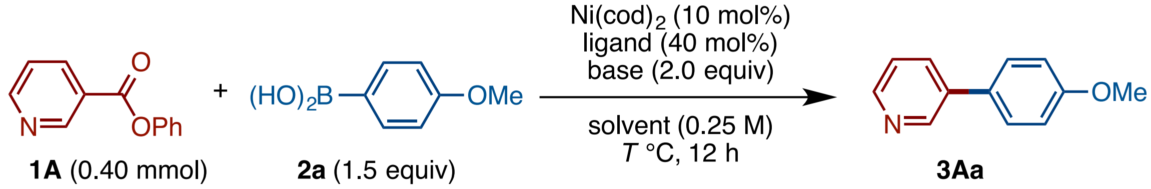


| entry | ligand | base | solvent | $T$ | yield of **3Aa** (%)[a] |
|---|---|---|---|---|---|
| 1 | none | $Na_2CO_3$ | 1,4-dioxane | 150 | 0 |
| 2 | $PCy_3$ | $Na_2CO_3$ | 1,4-dioxane | 150 | 26 |
| 3 | dppe | $Na_2CO_3$ | 1,4-dioxane | 150 | 0 |
| 4 | dpph | $Na_2CO_3$ | 1,4-dioxane | 150 | 51 |
| 5 | $PPh_2Me$ | $Na_2CO_3$ | 1,4-dioxane | 150 | 47 |
| 6 | $P(n\text{-}Bu)_3$ | $Na_2CO_3$ | 1,4-dioxane | 150 | 65 |
| 7 | $P(n\text{-}Bu)_3$ | $Li_2CO_3$ | 1,4-dioxane | 150 | 64 |
| 8 | $P(n\text{-}Bu)_3$ | $K_2CO_3$ | 1,4-dioxane | 150 | 23 |
| 9 | $P(n\text{-}Bu)_3$ | $Cs_2CO_3$ | 1,4-dioxane | 150 | 15 |
| 10 | $P(n\text{-}Bu)_3$ | $Et_3N$ | 1,4-dioxane | 150 | 68 |
| 11 | $P(n\text{-}Bu)_3$ | none | 1,4-dioxane | 150 | 49 |
| 12 | $P(n\text{-}Bu)_3$ | $Na_2CO_3$ | toluene | 150 | 95 |
| 13 | $P(n\text{-}Bu)_3$ | $Na_2CO_3$ | *m*-xylene | 150 | 38 |
| 14 | $P(n\text{-}Bu)_3$ | $Na_2CO_3$ | DME | 150 | 56 |
| 15 | $P(n\text{-}Bu)_3$ | $Na_2CO_3$ | *t*-AmylOH | 150 | 53 |
| 16 | $P(n\text{-}Bu)_3$ | $Na_2CO_3$ | toluene | 150 | 88[b] |
| 17 | $P(n\text{-}Bu)_3$ | $Na_2CO_3$ | toluene | 140 | 75 |
| 18 | $P(n\text{-}Bu)_3$ | $Na_2CO_3$ | toluene | 130 | 60 |
| 19 | $P(n\text{-}Bu)_3$ | $Na_2CO_3$ | toluene | 120 | 18 |

a. GC yield using dodecane as an internal standard. b. The reaction was conducted for 24 h using $Ni(OAc)_2$ (5 mol%) and $P(n\text{-}Bu)_3$ (20 mol%) as catalyst.

**Supplementary Table 2.** Investigation of the phenylboronic acids derivatives

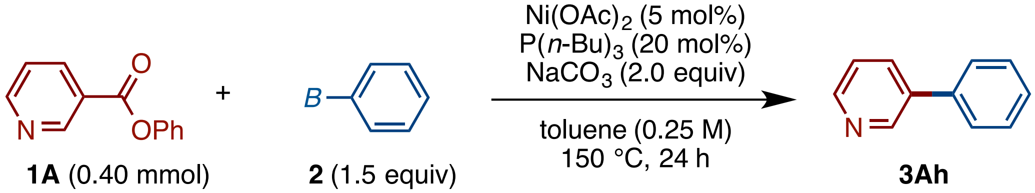


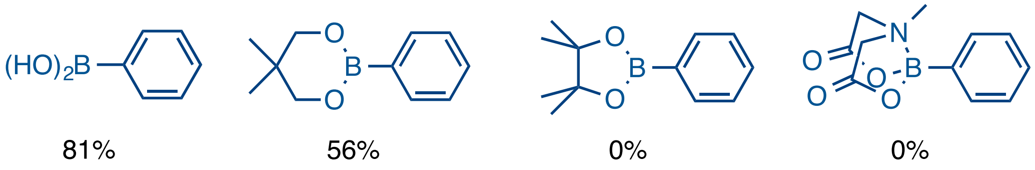

**Supplementary Table 3.** Investigation of alternative protocols

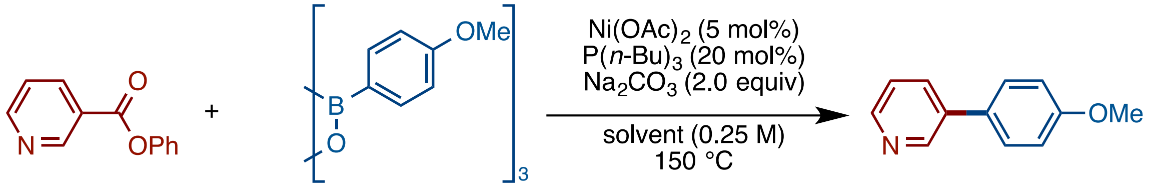


**1A** (0.40 mmol) **2a** (0.5 equiv) **3Aa**

| entry | solvent | conditions | GC yield (%) |
|---|---|---|---|
| 1 | toluene | close system (J-Young) | 84 |
| 2 | *m*-xylene | close system (J-Young) | 57 |
| 3 | anisole | close system (J-Young) | 23 |
| 4 | anisole | open system (Schlenk + condenser) | 89 (82) |

The number in the parenthesis shows the isolated yield.

**Supplementary Table 4.** The effect of the aryl-substituent of carboxylic acids

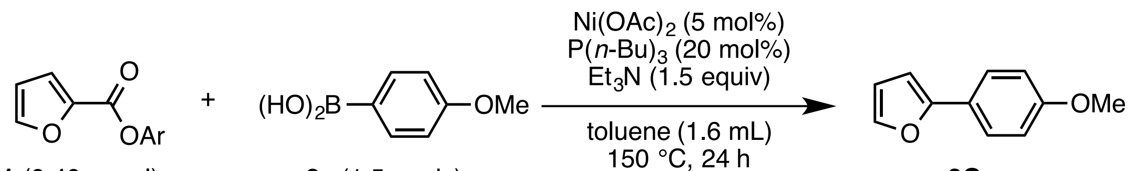


**1** (0.40 mmol) **2a** (1.5 equiv) **3Oa**

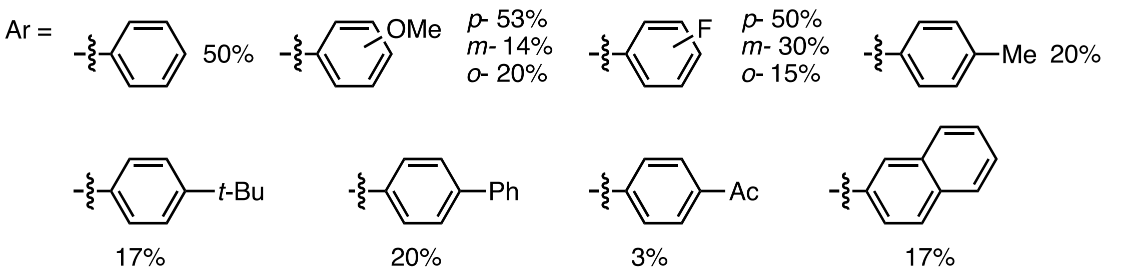

**Supplementary Table 5.** Competitive experiments between phenyl esters and haloarenes

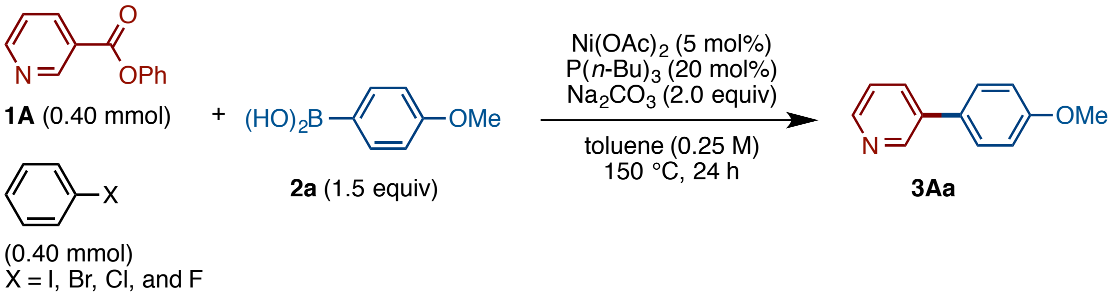


GC yield

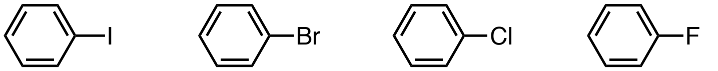


| | Ph–I | Ph–Br | Ph–Cl | Ph–F |
|---|---|---|---|---|
| | **3Aa:** 0%<br>**1A:** 78% | **3Aa:** 0%<br>**1A:** 99% | **3Aa:** 43%<br>**1A:** 28% | **3Aa:** 66%<br>**1A:** 1% |
| 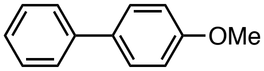 | 14% | 5% | 23% | 0% |

**Supplementary Table 6.** The effect of salts for the reaction of alkenylboronic acid **2q**

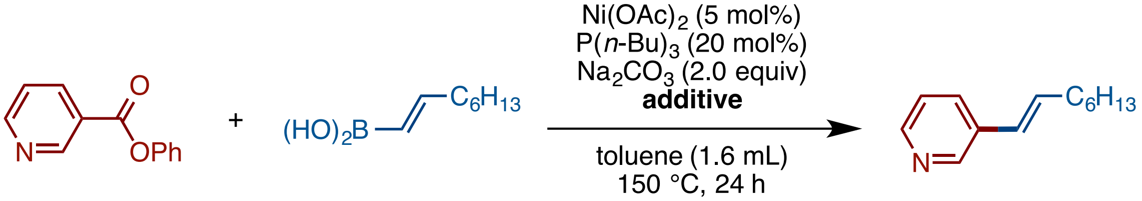


**1A** (0.40 mmol) **2q** (0.60 mmol) **3Aq**

| entry | additive | isolated yield of **3Aq** |
|---|---|---|
| 1 | none | 24% |
| 2 | LiCl: 10 mol% | <20% |
| 3 | LiCl: 1 equiv | 46% |
| 4 | NaCl: 1 equiv | 46% |
| 5 | LiF: 1 equiv | 42% |
| 6 | NaF: 1 equiv | 46% |
| 7 | KF: 1 equiv | 38% |

**Supplementary Table 7.** The effect of DMAP for the reaction of **1AF**

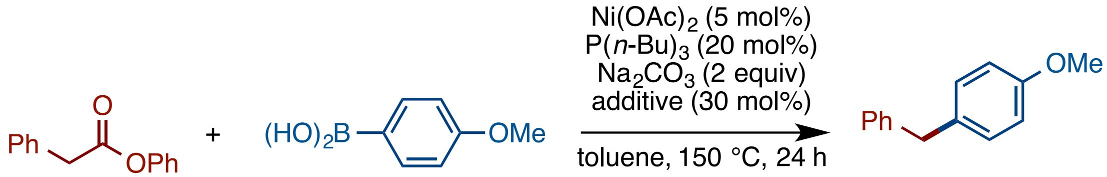


**1AF** (0.40 mmol) **2a** **3AFa**

| entry | additive | Isolated yield of **3** |
|---|---|---|
| 1 | none | 27% |
| 2 | none | 43%[a] |
| 3 | DMAP (30 mol%) | 46% |
| 4 | DMAP (1 equiv) | 26% |

a. The reaction was 48 h.

**Supplementary Table 8.** Calculated total (in a.u., at the M06L/BS1 level of theory) and relative energies (in kcal/mol) of the reactants, transition states, intermediates and products of the studied C(acyl)-O and C(aryl)-O oxidative addition of phenyl 3-pyridinecarboxylate to $Ni[P(n\text{-}Bu)_3]_2$ complex. For cartesian coordinates of all reported structures see Supplementary Table 13).

| Structure | | Energies | | | | Relative Energies | | | |
|---|---|---|---|---|---|---|---|---|---|
| | | $-E_{tot}$ | -(E +ZPC) | -H | -G | ΔE | Δ(E+ZPC) | ΔH | ΔG |
| **$Ni[P(n\text{-}Bu)_3]_2$ → $Ni[P(n\text{-}Bu)_3]$ + $[P(n\text{-}Bu)_3]$** | | | | | | | | | |
| **$Ni[P(n\text{-}Bu)_3]_2$** | | 1799.047953 | 1798.302214 | 1798.262328 | 1798.375858 | -42.1 | -41.5 | -41.1 | -28 |
| **$Ni[P(n\text{-}Bu)_3]$** | | 984.149528 | 983.776269 | 983.755037 | 983.826269 | | | | |
| **$[P(n\text{-}Bu)_3]$** | | 814.831310 | 814.459779 | 814.441825 | 814.504941 | | | | |
| **$Ni[P(n\text{-}Bu)_3]$ + $[P(n\text{-}Bu)_3]$** | | 1798.980838 | 1798.236048 | 1798.196862 | 1798.33121 | 0.0 | 0.0 | 0.0 | 0.0 |
| Oxidative addition of phenyl 3-pyridinecarboxylate (1A) to $Ni[P(n\text{-}Bu)_3]_2$ catalyst | | | | | | | | | |
| **$Ni[P(n\text{-}Bu)_3]_2$ + (PyrCOOPh)** | | 2466.886649 | 2466.956609 | 2465.904096 | 2466.070012 | 0 | 0 | 0 | 0 |
| **$Ni[P(n\text{-}Bu)_3]_2$(PyrCOOPh)** | **ISO-A(C=O)** | 2466.955448 | 2466.022313 | 2465.969010 | 2466.111613 | -43.2/0 | -41.2/0 | -40.7/0 | -26.1/0 |
| | **ISO-B(C=C)** | 2466.943787 | 2466.012035 | 2465.958304 | 2466.102092 | -35.9 | -34.8 | -34 | -20.1 |
| | **TS[C(acyl)-O]** | 2466.924214 | 2465.994081 | 2465.940003 | 2466.085737 | -23.6/19.6 | -23.5/17.7 | -22.5/18.2 | -9.9/16.2 |
| | **TS[C(aryl)-O]** | 2466.918790 | 2465.987373 | 2465.933787 | 2466.077801 | -20.2 | -19.3 | -18.6 | -4.9 |
| **$Ni[P(n\text{-}Bu)_3]_2$(PyrCO)(OPh), from TS[C(acyl)-O]** | | 2466.954329 | 2466.022239 | 2465.968331 | 2466.112966 | -42.5 | -41.2 | -40.3 | -27 |
| **$Ni[P(n\text{-}Bu)_3]_2$(PyrCOO)(Ph), from TS[C(aryl)-O]** | | 2466.973059 | 2466.039236 | 2465.985560 | 2466.130675 | -54.2 | -51.9 | -51.1 | -38.1 |
| **$Ni[P(n\text{-}Bu)_3]_2$ + $Na_2CO_3$ → $Ni[P(n\text{-}Bu)_3]_2[Na_2CO_3]$** | | | | | | | | | |
| **$NiP_2[Na_2CO_3]$** | **iso2** | 2387.576020 | 2386.809538 | 2386.761115 | 2386.894495 | -21.0 | -19.0 | -18.2 | -6.4 |
| | **Iso1** | 2387.580192 | 2386.814613 | 2386.766031 | 2386.899542 | -23.6 | -22.2 | -21.3 | -9.6 |
| **$Na_2CO_3$** | | 588.494676 | 588.4770470 | 588.469801 | 588.508414 | | | | |
| **$Ni[P(n\text{-}Bu)_3]_2$** | | 1799.047953 | 1798.302214 | 1798.262328 | 1798.375858 | | | | |
| **$Ni[P(n\text{-}Bu)_3]_2$ + $Na_2CO_3$** | | 2387.542629 | 2386.779261 | 2386.732129 | 2386.884272 | 0.0 | 0.0 | 0.0 | 0.0 |
| **$Ni[P(n\text{-}Bu)_3]_2[Na_2CO_3]$ → $Ni[P(n\text{-}Bu)_3][Na_2CO_3]$ + $[P(n\text{-}Bu)_3]$** | | | | | | | | | |
| **$NiP_2[Na_2CO_3]$, Iso1** | | 2387.580192 | 2386.814613 | 2386.766031 | 2386.899542 | 0.0 | 0.0 | 0.0 | 0.0 |
| **$NiP[Na_2CO_3]$, iso1** | | 1572.707774 | 1572.316435 | 1572.287108 | 1572.380429 | | | | |
| **$[P(n\text{-}Bu)_3]$** | | 814.831310 | 814.459779 | 814.441825 | 814.504940 | | | | |
| **$NiP[Na_2CO_3]$ + $[P(n\text{-}Bu)_3]$** | | 2387.539084 | 2386.776214 | 2386.728933 | 2386.885369 | 25.8 | 24.1 | 23.2 | 8.9 |

**Supplementary Table 9.** Decarbonylative transmetalation in the absence of $Na_2CO_3$ base. Calculated total (in a.u., at the M06L/BS1 level of theory) and relative energies (in kcal/mol) of the reactants, transition states, intermediates and products of the studied phenyl 3-pyridinecarboxylate and *p*-anisylboronic acid [*i.e.* MeOPh-B(OH)$_2$] cross-coupling catalyzed by Ni[P(*n*-Bu)$_3$]$_2$ catalyst in the absence of base (*i.e.* $Na_2CO_3$). For cartesian coordinates of all reported structure see Supplementary Table 13.

| Structure | | Energies | | | | Relative Energies | | | |
|---|---|---|---|---|---|---|---|---|---|
| | | -$E_{tot}$ | -(E +ZPC) | -H | -G | ΔE | Δ(E+ZPC) | ΔH | ΔG |
| I. Decarbonylation-then-transmetalation from the oxidative addition product Ni[P(n-Bu)$_3$]$_2$(PyrCO)(OPh), after one of the ligands [*i.e.* P(n-Bu)$_3$ dissociation]. | | | | | | | | | |
| I.A.1. P(n-Bu)$_3$ - dissociation | | | | | | | | | |
| **Ni[P(n-Bu)$_3$]$_2$(PyrCO)(OPh)** | | 2466.954329 | 2466.022239 | 2465.968331 | 2466.112966 | [0.0] | [0.0] | [0.0] | [0.0] |
| **[P(n-Bu)$_3$]** | | 814.831310 | 814.459779 | 814.441825 | 814.504941 | | | | |
| **[Ni[P(n-Bu)$_3$](PyrCO)(OPh)+[P(n-Bu)$_3$]** | | 2466.905400 | 2466.976871 | 2466.924291 | 2466.090237 | [30.7] | [28.5] | [27.6] | [14.3] |
| I.A.2. Decarbonylation | | | | | | | | | |
| **Ni[P(n-Bu)$_3$](PyrCO)(OPh)** | | 1652.074090 | 1651.517092 | 1651.482466 | 1651.585296 | 0.0 | 0.0 | 0.0 | 0.0 |
| **TS [(OC-C(Pyr))** | | 1652.062105 | 1651.506432 | 1651.472146 | 1651.573609 | 7.5 | 6.7 | 6.5 | 7.3 |
| **Ni[P(n-Bu)$_3$](CO)(OPh)(Pyr)** | | 1652.071579 | 1651.515716 | 1651.480567 | 1651.584590 | 1.6 | 0.9 | 1.2 | 0.4 |
| I.B. Transmetalation from the Ni[P(n-Bu)$_3$](CO)(OPh)(Pyr) complex | | | | | | | | | |
| **Ni[P(n-Bu)$_3$](CO)(OPh)(Pyr)** | | 1652.071579 | 1651.515716 | 1651.480567 | 1651.584590 | | | | |
| **MeOPh_B(OH)$_2$** | | 522.743995 | 522.585484 | 522.574127 | 522.622372 | | | | |
| **Ni[P(n-Bu)$_3$](CO)(OPh)(Pyr) +MeOPh_B(OH)$_2$** | | 2174.825574 | 2174.101200 | 2174.054694 | 2174.206962 | 0.0 | 0.0 | 0.0 | 0.0 |
| **Ni[P(n-Bu)$_3$](CO)(Pyr)(OPh)** | **iso1** | 2174.846852 | 2174.129090 | 2174.082738 | 2174.211641 | -13.4 | -17.5 | -17.6 | -2.9 |
| **[MeOPh_B(OH)$_2$], B-C, pre-reaction complex** | **Iso2** | 2174.845932 | 2174.129063 | 2174.082710 | 2174.208173 | | | | |
| **TS(B-C act.)** | **iso1** | 2174.804872 | 2174.091779 | 2174.046422 | 2174.169626 | | | | |
| | **iso2** | 2174.805385 | 2174.091461 | 2174.045258 | 2174.170968 | 12.7 | 6.1 | 5.9 | 22.6 |
| **Ni[P(n-Bu)$_3$](Pyr)(CO)(PhOMe)[(HO)$_2$B-OPh]** | **CO-N trans, Iso1** | 2174.832348 | 2174.116384 | 2174.069275 | 2174.200053 | -4.3 | -9.5 | -9.2 | 4.3 |
| | **CO-N trans, Iso2** | 2174.831376 | 2174.115632 | 2174.068231 | 2174.201346 | | | | |
| **Ni[P(n-Bu)$_3$](Pyr)(CO)(PhOMe)** | **N-CO trans** | 1691.334065 | 1690.750683 | 1690.71358 | 1690.822796 | | | | |
| | **P-CO trans** | 1691.334516 | 1690.750869 | 1690.71376 | 1690.823534 | | | | |
| **[(HO)$_2$B-OPh], iso2** | | 483.482975 | 483.352792 | 483.343067 | 483.387441 | | | | |
| **Ni[P(n-Bu)$_3$](Pyr)(CO)(PhOMe), N-CO trans + [(HO)$_2$B-OPh]** | | 2174.817040 | 2174.103475 | 2174.056647 | 2174.210237 | 5.4 | -1.4 | -1.2 | -2.1 |
| I.C. Reductive Elimination | | | | | | | | | |
| **Ni[P(n-Bu)$_3$]** | **N-CO trans** | 1691.334065 | 1690.750683 | 1690.713580 | 1690.822796 | 0.0 | 0.0 | 0.0 | 0.0 |

| | | | | | | | | | | |
|---|---|---|---|---|---|---|---|---|---|---|
| **(Pyr)(CO)(PhOMe)** | **TS (RE)** | | 1691.327295 | 1690.745196 | 1690.708668 | 1690.816121 | 4.3 | 3.4 | 3.1 | 4.2 |
| | **PROD** | | 1691.383292 | 1690.798020 | 1690.762157 | 1690.867216 | -30.9 | -29.7 | -30.5 | -27.9 |
| **Ni[P(n-Bu)$_3$](CO)** | | | 1097.533889 | 1097.152363 | 1097.129157 | 1097.206373 | | | | |
| **(PyrPhOMe)** | | | 593.806680 | 593.603521 | 593.591395 | 593.641012 | | | | |
| **Ni[P(n-Bu)$_3$](CO) + (PyrPhOMe)** | | | 1691.340569 | 1690.755884 | 1690.720552 | 1690.847385 | -4.1 | -3.3 | -4.4 | -15.4 |

II. Transmetalation-then-decarbonylation from the complex Ni[P(n-Bu)$_3$](PyrCO)(OPh)
II.A. Transmetalation

| | | | | | | | | | | |
|---|---|---|---|---|---|---|---|---|---|---|
| **Ni[P(n-Bu)$_3$](PyrCO)(OPh)** | | | 1652.074090 | 1651.517092 | 1651.482466 | 1651.585296 | | | | |
| **MeOPh_B(OH)$_2$** | | | 522.743995 | 522.585484 | 522.574127 | 522.622372 | | | | |
| **Ni[P(n-Bu)$_3$](PyrCO)(OPh)+MeOPh_B(OH)$_2$** | | | 2174.818085 | 2174.102576 | 2174.056593 | 2174.207668 | 0.0 | 0.0 | 0.0 | 0.0 |
| **B-OPh product** | | **Iso1** | 2174.837801 | 2174.120636 | 2174.074629 | 2174.199528 | | | | |
| | | **Iso2** | 2174.840580 | 2174.123170 | 2174.077233 | 2174.202028 | | | | |
| **TS33 (B-C act.)** | | **iso2** | 2174.805385 | 2174.091461 | 2174.045258 | 2174.170968 | | | | |
| **CO-N_tr,** | | **iso1** | 2174.811068 | 2174.095061 | 2174.049299 | 2174.174807 | 4.4 | 4.7 | 4.6 | 20.6 |
| | | **iso2** | 2174.815520 | 2174.098756 | 2174.053294 | 2174.177336 | 1.6 | 2.4 | 2.1 | 19.0 |
| **Ni[P(n-Bu)$_3$](PyrCO)(PhOMe)(OPh_B(OH)$_2$** | | **Iso_1a** | 2174.823735 | 2174.107507 | 2174.060940 | 2174.190835 | | | | |
| | | **Iso-1b** | 2174.823578 | 2174.106501 | 2174.060355 | 2174.187237 | | | | |
| **Ni[P(n-Bu)$_3$](PyrCO)(PhOMe)** | | | 1691.327612 | 1690.742389 | 1690.706162 | 1690.811668 | | | | |
| **[(HO)$_2$B-OPh], iso2** | | | 483.482975 | 483.352792 | 483.343067 | 483.387441 | | | | |
| **Ni[P(n-Bu)$_3$](PyrCO)(PhOMe) + PhO-B(OH)$_2$** | | | 2174.810587 | 2174.095182 | 2174.049229 | 2174.199109 | 4.7 | 4.6 | 4.6 | 5.4 |

II.B. Decarbonylation

| | | | | | | | | |
|---|---|---|---|---|---|---|---|---|
| **Ni[P(n-Bu)$_3$](PyrCO)(PhOMe)** | 1691.327612 | 1690.742389 | 1690.706162 | 1690.811668 | 0.0 | 0.0 | 0.0 | 0.0 |
| **TS (decarb.)** | 1691.315774 | 1690.731822 | 1690.695944 | 1690.801493 | 7.4 | 6.6 | 6.4 | 6.4 |
| **Ni[P(n-Bu)$_3$](Pyr)(CO)(PhOMe)** | 1691.334534 | 1690.750651 | 1690.713580 | 1690.822512 | -4.4 | -5.2 | -4.7 | -6.8 |

II.C. For reductive elimination see Section I.C

III. Transmetalation-then-decarbonylation from the oxidative addition product Ni[P(n-Bu)$_3$]$_2$(PyrCO)(OPh), i.e. without the ligand dissociation.
III.A. Transmetalation

| | | | | | | | | | |
|---|---|---|---|---|---|---|---|---|---|
| **Ni[P(n-Bu)$_3$]$_2$(PyrCO)(OPh)** | | 2466.954329 | 2466.022239 | 2465.968331 | 2466.112966 | | | | |
| **MeOPh_B(OH)$_2$** | | 522.743995 | 522.585484 | 522.574127 | 522.622372 | | | | |
| **Ni[P(n-Bu)$_3$]$_2$(PyrCO)(OPh) + MeOPh_B(OH)$_2$** | | 2989.698324 | 2988.607723 | 2988.542458 | 2988.735338 | 0.0 | 0.0 | 0.0 | 0.0 |
| **TS (B-C act.),** | **iso-1_up** | 2989.673968 | 2988.582911 | 2988.518073 | 2988.685725 | 15.3 | 15.7 | 15.3 | 31.1 |
| | **iso-2_up** | 2989.675404 | 2988.585118 | 2988.520179 | 2988.685227 | | | | |
| **Ni[P(n-Bu)$_3$]$_2$(PyrCO)(PhOMe)[PhO-B(OH)$_2$]** | | 2989.737961 | 2988.643899 | 2988.578853 | 2988.746093 | -24.9 | -22.7 | -22.8 | -6.8 |
| **Ni[P(n-Bu)$_3$]$_2$(PyrCO)(PhOMe)** | | 2506.221645 | 2505.262347 | 2505.206564 | 2505.354794 | | | | |

| | | | | | | | | |
|---|---|---|---|---|---|---|---|---|
| **[(HO)$_2$B-OPh], iso2** | 483.482975 | 483.352792 | 483.343067 | 483.387441 | | | | |
| **Ni[P(n-Bu)$_3$]$_2$(PyrCO)(PhOMe) + PhO-B(OH)$_2$** | 2989.704620 | 2988.615139 | 2988.549631 | 2988.742235 | -4.0 | -4.7 | -4.5 | -4.3 |
| III.B. Decarbonylation | | | | | | | | |
| **Ni[P(n-Bu)$_3$]$_2$(PyrCO)(PhOMe)** | 2506.221645 | 2505.262347 | 2505.206564 | 2505.354794 | [0.0] | [0.0] | [0.0] | [0.0] |
| **[P(n-Bu)$_3$]** | 814.831310 | 814.459779 | 814.441825 | 814.504941 | | | | |
| **Ni[P(n-Bu)$_3$](PyrCO)(PhOMe)** | 1691.327612 | 1690.742389 | 1690.706162 | 1690.811668 | | | | |
| **Ni[P(n-Bu)$_3$](PyrCO)(PhOMe) + [P(n-Bu)$_3$]** | 2506.158922 | 2505.202168 | 2505.147987 | 2505.316609 | [39.4] | [37.8] | [26.7] | [24.0] |
| **Ni[P(n-Bu)$_3$](PyrCO)(PhOMe)** | 1691.327612 | 1690.742389 | 1690.706162 | 1690.811668 | 0.0 | 0.0 | 0.0 | 0.0 |
| **TS (decarb.)** | 1691.315774 | 1690.731822 | 1690.695944 | 1690.801493 | 7.4 | 6.6 | 6.4 | 6.4 |
| **Ni[P(n-Bu)$_3$](Pyr)(CO)(PhOMe)** | 1691.334534 | 1690.750651 | 1690.713580 | 1690.822512 | -4.4 | -5.2 | -4.7 | -6.8 |
| III.C. For reductive elimination see Section I.C | | | | | | | | |

**Supplementary Table 10.** Decarbonylative transmetalation in the *presence* of the $Na_2CO_3$ base. $Na_2CO_3$ coordination to the C(acyl)-O oxidative addition product Ni[P(n-Bu)$_3$]$_2$(PyrCO)(OPh) and rearrangement. Calculated total (in a.u., at the M06L/BS1 level of theory) and relative energies (in kcal/mol) of the reactants, transition states, intermediates and products of the studied $Na_2CO_3$ coordination to the phenyl 3-pyridinecarboxylate oxidative addition product Ni[P(*n*-Bu)$_3$]$_2$(PyrCO)(OPh) and following multi-step rearrangement of the resulted intermediates. For cartesian coordinates of all reported structure see Supplementary Table 13.

| Structure | | Energies | | | | Relative Energies | | | |
|---|---|---|---|---|---|---|---|---|---|
| | | $-E_{tot}$ | -(E +ZPC) | -H | -G | ΔE | Δ(E+ZPC) | ΔH | ΔG |
| A. Reaction Ni[P(n-Bu)$_3$]$_2$(PyrCO)(OPh) + $Na_2CO_3$ → Ni[P(n-Bu)$_3$]$_2$(PyrCO)($Na_2CO_3$)(OPh) | | | | | | | | | |
| I.A.1. P(n-Bu)$_3$ - dissociation | | | | | | | | | |
| **Ni[P(n-Bu)$_3$]$_2$(PyrCO)(OPh)** | | 2466.954329 | 2466.022239 | 2465.968331 | 2466.112966 | | | | |
| **$Na_2CO_3$** | | 588.494676 | 588.477047 | 588.469801 | 588.508414 | | | | |
| **Ni[P(n-Bu)$_3$]$_2$(PyrCO)(OPh) + $Na_2CO_3$** | | 3055.449005 | 3054.499286 | 3054.438132 | 3054.621380 | 0.0 | 0.0 | 0.0 | 0.0 |
| **Ni[P(n-Bu)$_3$]$_2$(PyrCO)($Na_2CO_3$)(OPh)** | **Iso-1a** | 3055.488680 | 3054.537664 | 3054.476076 | 3054.636281 | -24.9 | -24.1 | -23.8 | -9.4 |
| | **Iso-1b** | 3055.488591 | 3054.536383 | 3054.475062 | 3054.634673 | | | | |
| B. Ni[P(n-Bu)$_3$]$_2$(PyrCO)($Na_2CO_3$)(OPh) → Ni[P(n-Bu)$_3$]$_2$(PyrCO)($Na_2CO_3$OPh) isomerization | | | | | | | | | |
| **Ni[P(n-Bu)$_3$]$_2$(PyrCO)($Na_2CO_3$)(OPh)** | **Iso-1a** | 3055.488680 | 3054.537664 | 3054.476076 | 3054.636281 | 0.0 | 0.0 | 0.0 | 0.0 |
| | **Iso-1b** | 3055.488591 | 3054.536383 | 3054.475062 | 3054.634673 | | | | |
| **Ni[P(n-Bu)$_3$]$_2$(PyrCO)($Na_2CO_3$OPh)** | **Iso-3a** | 3055.505151 | 3054.555050 | 3054.492800 | 3054.656882 | -10.3 | -10.9 | -10.5 | -12.9 |

| | | | | | | | | | |
|---|---|---|---|---|---|---|---|---|---|
| | **Iso-3b** | 3055.499666 | 3054.549503 | 3054.487244 | 3054.650785 | | | | |
| C. Ligand Exchange: $Ni[P(n\text{-}Bu)_3]_2(PyrCO)(Na_2CO_3OPh) \rightarrow Ni[P(n\text{-}Bu)_3]_2(PyrCO)(NaCO_3)$ + NaOPh | | | | | | | | | |
| **$Ni[P(n\text{-}Bu)_3]_2(PyrCO)(Na_2CO_3OPh)$** | **Iso-3a** | 3055.505151 | 3054.555050 | 3054.492800 | 3054.656882 | 0.0 | 0.0 | 0.0 | 0.0 |
| | **Iso-3b** | 3055.499666 | 3054.549503 | 3054.487244 | 3054.650785 | | | | |
| **$Ni[P(n\text{-}Bu)_3]_2(PyrCO)(NaCO_3)$** | **Down Iso-3a** | 2586.260084 | 2585.404600 | 2585.350746 | 2585.497264 | | | | |
| | **UP iso-3b** | 2586.262707 | 2585.407175 | 2585.353485 | 2585.498639 | | | | |
| **Na_OPh** | | 469.175242 | 469.082344 | 469.074299 | 469.114741 | | | | |
| **$Ni[P(n\text{-}Bu)_3]_2(PyrCO)(NaCO_3)$, iso3b_UP + NaOPh** | | 3055.437949 | 3054.489519 | 3054.427784 | 3054.613380 | 42.2 | 41.1 | 40.8 | 27.3 |
| D. Reaction: $Ni[P(n\text{-}Bu)_3]_2(PyrCO)(Na_2CO_3OPh) \rightarrow Ni[P(n\text{-}Bu)_3](PyrCO)(Na_2CO_3OPh)$ + $[P(n\text{-}Bu)_3]$ | | | | | | | | | |
| **$Ni[P(n\text{-}Bu)_3]_2(PyrCO)(Na_2CO_3OPh)$, Iso-3a** | | 3055.505151 | 3054.555050 | 3054.492800 | 3054.656882 | 0.0 | 0.0 | 0.0 | 0.0 |
| **$Ni[P(n\text{-}Bu)_3](PyrCO)(Na_2CO_3OPh)$** | **Iso1** | 2240.650977 | 2240.074859 | 2240.032693 | 2240.151469 | | | | |
| | **Iso2** | 2240.653829 | 2240.076958 | 2240.035154 | 2240.150825 | | | | |
| **$[P(n\text{-}Bu)_3]$** | | 814.831310 | 814.459779 | 814.441825 | 814.504941 | | | | |
| **$Ni[P(n\text{-}Bu)_3](PyrCO)(Na_2CO_3OPh)$, iso2 + $[P(n\text{-}Bu)_3]$** | | 3055.485139 | 3054.536737 | 3054.476979 | 3054.655766 | 12.6 | 11.5 | 9.9 | 0.7 |

**Supplementary Table 11.** Calculated total (in a.u., at the M06L/BS1 level of theory) and relative energies (in kcal/mol) of the reactants, transition states, intermediates and products of the studied phenyl 3-pyridinecarboxylate and *p*-anisylboronic acid [*i.e.* MeOPh-B$(OH)_2$] cross-coupling catalyzed by Ni[P(*n*-Bu)$_3$]$_2$ catalyst in the *presence* of base (*i.e.* $Na_2CO_3$). For cartesian coordinates of all reported structure, see Supplementary Table 13.

| Structure | | Energies | | | | Relative Energies | | | |
|---|---|---|---|---|---|---|---|---|---|
| | | $-E_{tot}$ | -(E +ZPC) | -H | -G | ΔE | Δ(E+ZPC) | ΔH | ΔG |
| I. Decarbonylation-then-transmetalation from the $Ni[P(n\text{-}Bu)_3](PyrCO)(Na_2CO_3OPh)$ cluster complex | | | | | | | | | |
| I.A. Decarbonylation: $Ni[P(n\text{-}Bu)_3](PyrCO)(Na_2CO_3OPh) \rightarrow Ni[P(n\text{-}Bu)_3](CO)(Pyr)(Na_2CO_3OPh)$ | | | | | | | | | |
| **$Ni[P(n\text{-}Bu)_3](PyrCO)(Na_2CO_3OPh)$** | **Iso-2** | 2240.653829 | 2240.076958 | 2240.035154 | 2240.150825 | 0.0 | 0.0 | 0.0 | 0.0 |
| | **TS** | 2240.612500 | 2240.039326 | 2239.996498 | 2240.118211 | 25.9 | 23.6 | 24.3 | 20.5 |
| **$Ni[P(n\text{-}Bu)_3](CO)(Pyr)(Na_2CO_3OPh)$,Trans-O** | | 2240.621459 | 2240.047162 | 2240.003860 | 2240.126299 | 20.3 | 18.7 | 19.6 | 15.4 |
| I.A.1. CO-dissociation: $Ni[P(n\text{-}Bu)_3](Pyr)(CO)(Na_2CO_3OPh) \rightarrow Ni[P(n\text{-}Bu)_3](Pyr)(Na_2CO_3OPh)$ + CO | | | | | | | | | |
| **$Ni[P(n\text{-}Bu)_3](PyrCO)(Na_2CO_3OPh)$** | **Trans-O** | 2240.621459 | 2240.047162 | 2240.003860 | 2240.126299 | 0.0 | 0.0 | 0.0 | 0.0 |
| | **Trans-N** | 2240.614635 | 2240.040420 | 2239.997244 | 2240.119472 | | | | |
| **CO** | | 113.296662 | 113.291641 | 113.288337 | 113.3107850 | | | | |
| **$Ni[P(n\text{-}Bu)_3](Pyr)(Na_2CO_3OPh)$,** | | 2127.308317 | 2126.742004 | 2126.701406 | 2126.817765 | | | | |

| | | | | | | | | | |
|---|---|---|---|---|---|---|---|---|---|
| **Ni[P(n-Bu)$_3$](Pyr)(Na$_2$CO$_3$OPh) + CO** | | 2240.604979 | 2240.033645 | 2239.989743 | 2240.128550 | 10.3 | 8.5 | 8.9 | -1.4 |
| I.B. Transmetalation from the Ni[P(n-Bu)$_3$](Pyr)(Na$_2$CO$_3$OPh) complex | | | | | | | | | |
| **Ni[P(n-Bu)$_3$](Pyr)(Na$_2$CO$_3$OPh),** | | 2127.308317 | 2126.742004 | 2126.701406 | 2126.817765 | | | | |
| **MeOPh_B(OH)$_2$** | | 522.743995 | 522.585484 | 522.574127 | 522.622372 | | | | |
| **Ni[P(n-Bu)$_3$](Pyr)(Na$_2$CO$_3$OPh) + MeOPh_B(OH)$_2$** | | 2650.052312 | 2649.327488 | 2649.275533 | 2649.440137 | 0.0 | 0.0 | 0.0 | 0.0 |
| **COMPLEX,** | | 2650.054169 | 2649.328488 | 2649.276691 | 2649.413974 | -1.2 | -0.6 | -0.7 | 16.4 |
| **TS1, B-O formation** | | | | no barrier was located | | | | | |
| **B-O complex** | | 2650.053240 | 2649.327730 | 2649.275783 | 2649.414957 | -0.6 | -0.2 | -0.2 | 15.8 |
| **TS2, B-C active.** | | 2650.025711 | 2649.302276 | 2649.250183 | 2649.390267 | 16.7 | 15.8 | 15.9 | 31.3 |
| **B-C complex: Ni[P(n-Bu)$_3$](Pyr)(MeOPh)(Na$_2$CO$_3$(OPh)B(OH)$_2$)** | **iso1** | 2650.040433 | 2649.315553 | 2649.263193 | 2649.403507 | 7.5 | 7.5 | 7.7 | 23 |
| | **iso2** | 2650.031954 | 2649.307755 | 2649.255311 | 2649.396236 | | | | |
| **Ni[P(n-Bu)$_3$](Pyr)(MeOPh), O-P trans, Iso-1** | | 1577.980792 | 1577.406174 | 1577.371479 | 1577.476373 | | | | |
| **Na$_2$CO$_3$(OPh)B(OH)$_2$** | | 1072.020568 | 1071.872413 | 1071.854530 | 1071.919027 | | | | |
| **Ni[P(n-Bu)$_3$](Pyr)(MeOPh) + Na$_2$CO$_3$(OPh)B(OH)$_2$** | | 2650.001360 | 2649.278587 | 2649.226009 | 2649.395400 | 32.0 | 30.7 | 31.1 | 28.1 |
| I.C. Reductive elimination | | | | | | | | | |
| **Ni[P(n-Bu)$_3$](Pyr)(MeOPh)(Na$_2$CO$_3$(OPh)B(OH)$_2$)** | **iso1** | 2650.040433 | 2649.315553 | 2649.263193 | 2649.403507 | 0.0 | 0.0 | 0.0 | 0.0 |
| | **iso2** | 2650.031954 | 2649.307755 | 2649.255311 | 2649.396236 | | | | |
| **TS, iso2** | | 2650.027094 | 2649.303384 | 2649.251479 | 2649.390739 | 8.4 | 7.6 | 7.4 | 8.0 |
| **COMPLEX_prod,** | **iso-1** | 2650.065887 | 2649.340579 | 2649.288578 | 2649.427124 | | | | |
| | **iso-2** | 2650.083236 | 2649.357099 | 2649.305590 | 2649.442512 | -26.9 | -26.1 | -26.6 | -24.5 |
| **Ni[P(n-Bu)$_3$](Na$_2$CO$_3$(OPh)B(OH)$_2$)** | | 2056.249575 | 2055.728396 | 2055.689485 | 2055.802417 | | | | |
| **MeOPh_Pyr** | | 593.806680 | 593.603521 | 593.591395 | 593.641012 | | | | |
| **Ni[P(n-Bu)$_3$](Na$_2$CO$_3$(OPh)B(OH)$_2$) + MeOPh_Pyr** | | 2650.056255 | 2649.331917 | 2649.280880 | 2649.443429 | -9.9 | -10.3 | -11.1 | -25.1 |
| II. Transmetalation-then-decarbonylation from the Ni[P(n-Bu)$_3$](PyrCO)(Na$_2$CO$_3$OPh) cluster complex | | | | | | | | | |
| II.A. Transmetalation from Ni[P(n-Bu)$_3$](PyrCO)(Na$_2$CO$_3$OPh) | | | | | | | | | |
| **Ni[P(n-Bu)$_3$](PyrCO)(Na$_2$CO$_3$OPh), Iso-1** | | 2240.653829 | 2240.076958 | 2240.035154 | 2240.150825 | | | | |
| **MeOPh_B(OH)$_2$** | | 522.743995 | 522.585484 | 522.574127 | 522.622372 | | | | |
| **Ni[P(n-Bu)$_3$](PyrCO)(Na$_2$CO$_3$OPh) + MeOPh_B(OH)$_2$** | | 2763.397824 | 2762.662442 | 2762.609281 | 2762.773197 | 0.0 | 0.0 | 0.0 | 0.0 |
| **TS(B-C active.)** | **Iso2_down** | 2763.364975 | 2762.630124 | 2762.576682 | 2762.718606 | 20.6 | 20.3 | 20.5 | 34.3 |
| | **Iso2_up** | 2763.375548 | 2762.641315 | 2762.588054 | 2762.729615 | 14.0 | 13.3 | 13.3 | 27.3 |
| **Ni[P(n-Bu)$_3$](PyrCO)(PhOMe)(Na$_2$CO$_3$OPhB(OH)$_2$)** | **Iso2_down** | 2763.384351 | 2762.648868 | 2762.594958 | 2762.738319 | | | | |
| | **Iso2_up** | 2763.385586 | 2762.650096 | 2762.596291 | 2762.740707 | 7.7 | 7.8 | 8.2 | 20.3 |
| II.B. Ni[P(n-Bu)$_3$](PyrCO)(PhOMe)(Na$_2$CO$_3$OPhB(OH)$_2$) → Ni[P(n-Bu)$_3$](PyrCO)(PhOMe) + (Na$_2$CO$_3$OPhB(OH)$_2$) | | | | | | | | | |

| | | | | | | | | |
|---|---|---|---|---|---|---|---|---|
| **Ni[P(n-Bu)$_3$](PyrCO)(PhOMe)(Na$_2$CO$_3$OPhB(OH)$_2$], Iso2_up** | 2763.385586 | 2762.650096 | 2762.596291 | 2762.739707 | 0.0 | 0.0 | 0.0 | 0.0 |
| **Ni[P(n-Bu)$_3$](PyrCO)(PhOMe)** | 1691.327612 | 1690.742389 | 1690.706162 | 1690.811668 | | | | |
| **Na$_2$CO$_3$OPhB(OH)$_2$** | 1072.020568 | 1071.872413 | 1071.854530 | 1071.919027 | | | | |
| **Ni[P(n-Bu)$_3$](PyrCO)(PhOMe) + [Na$_2$CO$_3$OPhB(OH)$_2$]** | 2763.348180 | 2762.614802 | 2762.560692 | 2762.733495 | 23.5 | 22.1 | 22.3 | 3.8 |

II.C. Decarbonylation: Ni[P(n-Bu)$_3$](PyrCO)(PhOMe) → Ni[P(n-Bu)$_3$](Pyr)(CO)(PhOMe)
See Supplementary Table 9, section II.B
II.D. Reduction Elimination from the decarbonylated product Ni[P(n-Bu)$_3$](Pyr)(CO)(PhOMe)
See Supplementary Table 9, section I.C

**Supplementary Table 12.** Calculated total (in a.u., at the M06/BS1 level of theory) and relative energies (in kcal/mol) of the reactants, important transition states, intermediates and products of the studied phenyl 3-pyridinecarboxylate and *p*-anisylboronic acid [*i.e.* MeOPh-B(OH)$_2$] cross-coupling catalyzed by Ni[P(*n*-Bu)$_3$]$_2$ catalyst in the absence and presence of base (*i.e.* Na$_2$CO$_3$) conditions

| Structure | Energies | | | | Relative Energies | | | |
|---|---|---|---|---|---|---|---|---|
| | $-E_{tot}$ | -(E +ZPC) | -H | -G | ΔE | Δ(E+ZPC) | ΔH | ΔG |
| Ni[P(n-Bu)$_3$]$_2$ → Ni[P(n-Bu)$_3$] + [P(n-Bu)$_3$] | | | | | | | | |
| **[P(n-Bu)$_3$]** | 814.490909 | 814.122914 | 814.103415 | 814.170490 | | | | |
| **Na$_2$CO$_3$** | 588.394747 | 588.377485 | 588.369946 | 588.409119 | | | | |
| **MeOPh-B(OH)$_2$** | 522.478119 | 522.320396 | 522.308972 | 522.357204 | | | | |
| **Na$_2$CO$_3$(OPh)[B(OH)$_2$]** | 1071.684273 | 1071.535321 | 1071.517884 | 1071.581034 | | | | |
| **NaOPh** | 468.986985 | 468.894407 | 468.886389 | 468.926895 | | | | |
| **PyrCOOPh** | 667.449129 | 667.265396 | 667.252733 | 667.305218 | | | | |
| **Transmetalation from the Ni[P(n-Bu)$_3$]$_2$(OPh)(PyrCO) oxidative addition complex (i.e. in the absence of Na$_2$CO$_3$)** | | | | | | | | |
| **Ni[P(n-Bu)$_3$]$_2$(PyrCO)(OPh)** | 2465.849470 | 2464.923877 | 2464.870386 | 2465.012720 | | | | |
| **Ni[P(n-Bu)$_3$]$_2$(PyrCO)(OPh) + MeOPh-B(OH)$_2$** | 2988.327589 | 2987.244273 | 2987.179358 | 2987.369924 | 0.0 | 0.0 | 0.0 | 0.0 |
| **TS(TRME, no base)** | 2988.310009 | 2987.223803 | 2987.159129 | 2987.323873 | 11.1 | 12.8 | 12.7 | 28.9 |
| **Prod., iso1** | 2988.378488 | 2987.292071 | 2987.227559 | 2987.392273 | -31.9 | -30.0 | -30.2 | -14.0 |
| Reaction Ni[P(n-Bu)$_3$]$_2$(PyrCO)(OPh) + Na$_2$CO$_3$ → Ni[P(n-Bu)$_3$]$_2$(PyrCO)(Na$_2$CO$_3$OPh) | | | | | | | | |
| **Ni[P(n-Bu)$_3$]$_2$(PyrCO)(OPh) + Na$_2$CO$_3$** | 3054.244217 | 3053.301362 | 3053.240332 | 3053.421839 | 0.0 | 0.0 | 0.0 | 0.0 |
| **Ni[P(n-Bu)$_3$]$_2$(PyrCO)(Na$_2$CO$_3$OPh)** | 3054.299150 | 3053.355753 | 3053.293809 | 3053.455389 | -34.5 | -34.1 | -33.6 | -21.1 |
| Reaction Ni[P(n-Bu)$_3$]$_2$(PyrCO)(Na$_2$CO$_3$OPh) → Ni[P(n-Bu)$_3$](PyrCO)(Na$_2$CO$_3$OPh) + [P(n-Bu)$_3$] | | | | | | | | |
| **Ni[P(n-Bu)$_3$]$_2$(PyrCO)(Na$_2$CO$_3$OPh)** | 3054.29915 | 3053.355753 | 3053.293809 | 3053.455389 | 0.0 | 0.0 | 0.0 | 0.0 |

| | | | | | | | | |
|---|---|---|---|---|---|---|---|---|
| **Ni[P(n-Bu)$_3$](PyrCO)(Na$_2$CO$_3$OPh)** | 2239.79129 | 2239.217874 | 2239.176036 | 2239.292649 | | | | |
| **Ni[P(n-Bu)$_3$](PyrCO)(Na$_2$CO$_3$OPh) + [P(n-Bu)$_3$]** | 3054.282199 | 3053.340788 | 3053.279451 | 3053.463139 | 10.6 | 9.4 | 9.0 | -4.9 |
| Transmetalation from the Ni[P(n-Bu)$_3$](Pyr)(Na$_2$CO$_3$OPh) complex | | | | | | | | |
| **Ni[P(n-Bu)$_3$](PyrCO)(Na$_2$CO$_3$OPh) + MeOPh-B(OH)$_2$** | 2762.269409 | 2761.538270 | 2761.485008 | 2761.649853 | 0.0 | 0.0 | 0.0 | 0.0 |
| **B-O complex** | 2762.273253 | 2761.541162 | 2761.488113 | 2761.627769 | -2.4 | -1.8 | -2.0 | 13.9 |
| **TS(TRME, Na$_2$CO$_3$OPh)** | 2762.260184 | 2761.529096 | 2761.476239 | 2761.617153 | 5.8 | 5.8 | 5.5 | 20.5 |
| **Product** | 2762.270430 | 2761.537494 | 2761.484054 | 2761.626846 | -0.7 | 0.5 | 0.6 | 14.4 |
| **Ni[P(n-Bu)$_3$](PyrCO)(MeOPh) + Na$_2$CO$_3$(OPh)[B(OH)$_2$]** | 2762.232157 | 2761.502610 | 2761.448586 | 2761.619122 | 23.4 | 22.4 | 22.9 | 19.3 |
| Decarbonylation: Ni[P(n-Bu)$_3$](PyrCO)(PhOMe) $\rightarrow$ Ni[P(n-Bu)$_3$](Pyr)(CO)(PhOMe) | | | | | | | | |
| **Ni[P(n-Bu)$_3$](PyrCO)(MeOPh)** | 1690.547884 | 1689.967289 | 1689.930702 | 1690.038088 | 0.0 | 0.0 | 0.0 | 0.0 |
| **TS (decarbonylation** | 1690.534837 | 1689.955043 | 1689.919302 | 1690.024305 | 8.2 | 7.7 | 7.2 | 8.7 |
| **Ni[P(n-Bu)$_3$](Pyr)(CO)(MeOPh)** | 1690.553265 | 1689.974446 | 1689.937195 | 1690.047382 | -3.4 | -4.5 | -4.1 | -5.8 |
| Reductive Elimination | | | | | | | | |
| **Ni[P(n-Bu)$_3$](Pyr)(CO)(MeOPh)** | 1690.553265 | 1689.974446 | 1689.937195 | 1690.047382 | 0.0 | 0.0 | 0.0 | 0.0 |
| **TS** | 1690.546591 | 1689.968420 | 1689.932022 | 1690.038307 | 4.2 | 3.8 | 3.3 | 5.7 |

**Supplementary Table 13.** Cartersian Coordinates (in Å) of all structures reported in Supplementary Tables 8–12.

**P(n-Bu)$_3$**

| | | | |
|---|---|---|---|
| P | -0.22309200 | 0.05246700 | -2.07745000 |
| C | 1.08468400 | -1.12802400 | -1.44307700 |
| C | 0.35351500 | 1.63631300 | -1.26900500 |
| C | -1.75929200 | -0.48077700 | -1.14262200 |
| C | 0.89197000 | 1.69646100 | 0.16815800 |
| C | 2.00432600 | 2.72551600 | 0.33896600 |
| C | -2.18886600 | 0.24325000 | 0.12669600 |
| C | -3.56506300 | -0.19756900 | 0.60942500 |
| C | 0.82714700 | -1.91461400 | -0.15952500 |
| C | 2.08879500 | -2.55818200 | 0.40234500 |
| C | 2.63777300 | 2.68819200 | 1.71951000 |
| C | 1.87505800 | -3.18757400 | 1.76718800 |
| C | -3.94158800 | 0.39784000 | 1.95477500 |
| H | -0.45215800 | 2.36704200 | -1.40917600 |
| H | 1.15001200 | 1.97381400 | -1.94683400 |
| H | 0.08175200 | 1.92818500 | 0.86248600 |
| H | 1.27776100 | 0.72545600 | 0.48514700 |
| H | -1.63818200 | -1.54601300 | -0.91549000 |
| H | -2.58137700 | -0.43380100 | -1.86699400 |
| H | -1.45701400 | 0.07075400 | 0.92173500 |
| H | -2.19594600 | 1.32802400 | -0.03797300 |
| H | 1.99115200 | -0.51696000 | -1.32588400 |
| H | 1.31757300 | -1.83100600 | -2.25091000 |
| H | 0.37808300 | -1.27261400 | 0.60810600 |
| H | 0.08070200 | -2.69729900 | -0.35139600 |
| H | 1.60519700 | 3.72932500 | 0.13822900 |
| H | 2.76947300 | 2.55355000 | -0.43104000 |
| H | 1.94779800 | 3.05310400 | 2.48796100 |
| H | 2.91629000 | 1.66302100 | 1.99515900 |
| H | 2.88259100 | -1.79889900 | 0.46028500 |
| H | 2.45510900 | -3.31468500 | -0.30436500 |
| H | 2.79853800 | -3.61237500 | 2.17122800 |
| H | -3.59408400 | -1.29454500 | 0.66332400 |
| H | -4.31440100 | 0.08060100 | -0.14417100 |
| H | -4.92048700 | 0.05011300 | 2.29819900 |
| H | -3.20858800 | 0.13172500 | 2.72403900 |
| H | -3.97891200 | 1.49206300 | 1.90840800 |
| H | 1.50807600 | -2.44815000 | 2.48819800 |
| H | 1.13378400 | -3.99283400 | 1.72335200 |
| H | 3.54288300 | 3.30039900 | 1.77311800 |

**Na$_2$CO$_3$**

| | | | |
|---|---|---|---|
| C | -0.00000300 | 0.55668100 | 0.00051800 |
| O | 1.12681600 | 1.17058900 | 0.00007500 |
| O | -1.12684800 | 1.17054400 | -0.00032300 |
| O | -0.00000500 | -0.77801900 | 0.00046000 |
| Na | -2.21369100 | -0.72022900 | -0.0001450 |
| Na | 2.21371900 | -0.72022600 | -0.0002920 |

**PyrCOOPh,** phenyl 3-pyridinecarboxylate (**1A**)

| | | | |
|---|---|---|---|
| C | -0.43246400 | 0.58772500 | -0.20462800 |
| O | 0.41039400 | -0.44292700 | 0.08791300 |
| O | -0.07175000 | 1.69467300 | -0.54090700 |
| C | -1.84428700 | 0.17326800 | -0.06762500 |
| C | -2.23591700 | -1.10144100 | 0.35708400 |
| N | -3.50934700 | -1.47871600 | 0.48980300 |
| C | -4.44658200 | -0.56961200 | 0.19245800 |
| C | -2.84071700 | 1.10423100 | -0.37052900 |
| C | -4.16757500 | 0.72555600 | -0.23943000 |
| H | -1.48219700 | -1.84690900 | 0.60029100 |
| H | -5.47898800 | -0.89745900 | 0.30609300 |
| H | -2.55368200 | 2.09868400 | -0.69966700 |
| H | -4.97442700 | 1.41541400 | -0.46430700 |
| C | 1.78919900 | -0.24072900 | 0.05683500 |
| C | 2.39501800 | 0.83390700 | 0.70030200 |
| C | 3.78422000 | 0.91862500 | 0.69273400 |
| C | 4.55241100 | -0.05540900 | 0.05848600 |
| C | 2.53816200 | -1.22421400 | -0.57813100 |
| C | 3.92647300 | -1.12697700 | -0.57406100 |
| H | 1.79082200 | 1.58706700 | 1.19153900 |
| H | 4.26717800 | 1.75382600 | 1.19138900 |
| H | 5.63554700 | 0.01966600 | 0.05960800 |
| H | 2.02726900 | -2.04985300 | -1.06277100 |
| H | 4.51710200 | -1.89297600 | -1.06782100 |

**Ni[P(n-Bu)]**

| | | | |
|---|---|---|---|
| Ni | -3.33216300 | -0.00258600 | 0.01209700 |
| P | -1.31795900 | 0.00144100 | 0.00622400 |
| C | -0.54147400 | -1.11410200 | -1.27370100 |
| C | -0.54130500 | 1.66573600 | -0.32802400 |
| C | -0.52906500 | -0.54230400 | 1.60795800 |
| C | 0.86514900 | 1.76378500 | -0.90813600 |
| C | 1.29590000 | 3.20692000 | -1.14079900 |
| C | 0.88125600 | -0.09396700 | 1.97270600 |
| C | 1.32123200 | -0.61865100 | 3.33411700 |
| C | 0.86581100 | -1.66536200 | -1.07449200 |
| C | 1.29217500 | -2.58763000 | -2.21002700 |
| C | 2.68633100 | 3.32092300 | -1.74001500 |
| C | 2.68111500 | -3.16818900 | -2.01228000 |
| C | 2.71526700 | -0.16020000 | 3.72314300 |
| H | -0.60087000 | 2.22394400 | 0.61702300 |
| H | -1.23971700 | 2.17431900 | -1.00401100 |
| H | 1.59444500 | 1.27674200 | -0.24830200 |
| H | 0.91374500 | 1.21732800 | -1.86108600 |
| H | -0.59323400 | -1.63949300 | 1.61942600 |
| H | -1.21998800 | -0.20888300 | 2.39215400 |
| H | 1.60314300 | -0.42220700 | 1.21434200 |
| H | 0.93330700 | 1.00442500 | 1.97793800 |
| H | -0.60387500 | -0.57015200 | -2.22685400 |
| H | -1.23911600 | -1.95407200 | -1.37880300 |
| H | 1.59522300 | -0.85018000 | -0.98524200 |
| H | 0.91860600 | -2.21771500 | -0.12483100 |
| H | 1.25347000 | 3.75110400 | -0.18715900 |
| H | 0.56386100 | 3.70000900 | -1.79500300 |
| H | 3.43758600 | 2.86172900 | -1.08826700 |
| H | 2.74315400 | 2.81144400 | -2.70813100 |
| H | 1.24982600 | -2.03233900 | -3.15733400 |
| H | 0.55765900 | -3.39848900 | -2.31036000 |
| H | 2.97179100 | -3.82512000 | -2.83695800 |
| H | 1.27698900 | -1.71661500 | 3.32595600 |
| H | 0.59557500 | -0.29970000 | 4.09488400 |

| | | | |
|---|---|---|---|
| H | 3.01572300 | -0.54629100 | 4.70133700 |
| H | 3.46054200 | -0.49513100 | 2.99347600 |
| H | 2.77468900 | 0.93272600 | 3.76783700 |
| H | 3.43511300 | -2.37677500 | -1.94004300 |
| H | 2.73751000 | -3.75404200 | -1.08835700 |
| H | 2.98046100 | 4.36247900 | -1.89839300 |

**$B(OH)_2$-PhOMe, iso2:** *p*-anisylboronic acid (**2a**)

| | | | |
|---|---|---|---|
| B | -2.63762800 | 0.11709300 | -0.00125400 |
| O | -3.37722300 | -1.03426900 | -0.02947300 |
| O | -3.32219500 | 1.30734700 | 0.02881900 |
| C | -1.08737600 | -0.00629500 | -0.00305300 |
| C | -0.47267600 | -1.27351500 | 0.01094900 |
| C | 0.90248000 | -1.42128600 | 0.01444000 |
| C | 1.72642400 | -0.28814400 | 0.00275300 |
| C | -0.23490400 | 1.10432700 | -0.01629200 |
| C | 1.15220100 | 0.98670900 | -0.01403100 |
| H | -1.10170600 | -2.16011700 | 0.01960900 |
| O | 3.06279400 | -0.52998100 | 0.00824600 |
| H | -0.64912200 | 2.11313400 | -0.03264200 |
| H | 1.77012500 | 1.87778100 | -0.02509700 |
| H | 1.37026100 | -2.40140500 | 0.02638000 |
| H | -2.74450800 | 2.07282200 | 0.05920700 |
| C | 3.92394900 | 0.59389500 | -0.00443700 |
| H | 4.93909900 | 0.19832300 | 0.00175000 |
| H | 3.77826100 | 1.20385500 | -0.90422600 |
| H | 3.77600000 | 1.22583900 | 0.87970300 |
| H | -4.31786500 | -0.83461300 | -0.02111800 |

**$(OH)_2$B-OPh, lowest isomer, iso2**

| | | | |
|---|---|---|---|
| B | -2.14015000 | 0.01066500 | 0.06296400 |
| O | -3.38394300 | -0.46015600 | -0.24778400 |
| O | -1.99299900 | 1.16385000 | 0.77184500 |
| O | -1.04630900 | -0.72513200 | -0.34158100 |
| C | 0.26011500 | -0.33943500 | -0.17883900 |
| C | 1.17588800 | -1.31746500 | 0.20718400 |
| C | 2.52218200 | -0.98992100 | 0.33424200 |
| C | 2.95962500 | 0.30737300 | 0.07665400 |
| C | 0.68932500 | 0.96004300 | -0.44949200 |
| C | 2.03807500 | 1.27554300 | -0.31621400 |
| H | 0.81772200 | -2.32383900 | 0.40228400 |
| H | 3.23115600 | -1.75546200 | 0.63767100 |
| H | 4.01083100 | 0.56081300 | 0.17646900 |
| H | -0.03198700 | 1.71091200 | -0.75319300 |
| H | 2.36911400 | 2.28921900 | -0.52544200 |
| H | -3.33211700 | -1.27709100 | -0.75031900 |
| H | -2.84922400 | 1.53680500 | 0.99665500 |

**NaOPh**

| | | | |
|---|---|---|---|
| O | -1.54748200 | 0.84553800 | -0.58549700 |
| C | -0.34077000 | 0.43494100 | -0.34636700 |
| C | 0.70867400 | 1.32339200 | 0.03954700 |
| C | 1.98737300 | 0.86925000 | 0.32565100 |
| C | 2.31369000 | -0.48911800 | 0.24737700 |
| C | 0.03088400 | -0.94363300 | -0.42960600 |
| C | 1.31764100 | -1.38555500 | -0.13831600 |
| H | 0.47294100 | 2.38460800 | 0.10175100 |
| H | 2.75227600 | 1.58800200 | 0.61840100 |
| H | 3.31814900 | -0.83526900 | 0.47364300 |
| H | -0.72039500 | -1.65785300 | -0.77715700 |
| H | 1.54813600 | -2.44735200 | -0.22159000 |
| Na | -2.82692800 | -0.42291900 | 0.5726560 |

**$NaCO_3$-$B(OH)_2$, lowest isomer, iso3**

| | | | |
|---|---|---|---|
| B | -1.79750000 | 0.08179800 | -0.00029700 |
| C | 0.61654000 | -0.35804000 | 0.00142600 |
| O | -0.69394100 | -0.78395100 | 0.00152600 |
| O | 0.85343200 | 0.88950600 | 0.00400900 |
| Na | 3.07154100 | 0.31507000 | -0.00168800 |
| O | 1.47507200 | -1.26330200 | -0.00130000 |
| O | -1.70583300 | 1.42950800 | -0.00210200 |
| O | -3.03033900 | -0.50802700 | -0.00090500 |
| H | -2.93972200 | -1.46380600 | 0.00175200 |
| H | -0.74611500 | 1.62741900 | -0.00008600 |

**$NaCO_3$[$B(OH)_2$][OPh], iso3**

| | | | |
|---|---|---|---|
| C | 2.98535300 | -0.20026700 | 0.00783700 |
| O | 4.01770300 | 0.49125600 | 0.02505000 |
| O | 1.73382000 | 0.37667200 | -0.00599400 |
| O | 2.92109200 | -1.47062200 | 0.00035600 |
| Na | 0.51822900 | -1.58152600 | -0.0344030 |
| Na | 5.22396900 | -1.42516800 | 0.02604400 |
| O | -1.25611500 | -0.32818600 | -0.04203300 |
| C | -2.56356600 | -0.27826100 | -0.01758000 |
| C | -3.35791600 | -1.45469800 | -0.00414000 |
| C | -4.74585100 | -1.39882700 | 0.02251500 |
| C | -5.41627500 | -0.17474000 | 0.03669600 |
| C | -3.26873100 | 0.95447200 | -0.00306200 |
| C | -4.65660600 | 0.99659700 | 0.02364600 |
| H | -2.84806700 | -2.41821600 | -0.01530000 |
| H | -5.31542800 | -2.32723700 | 0.03217700 |
| H | -6.50163200 | -0.13447100 | 0.05759000 |
| H | -2.69181600 | 1.87727800 | -0.01282600 |
| H | -5.15681100 | 1.96416300 | 0.03434600 |
| B | 1.33742600 | 1.72964900 | -0.00787400 |
| O | 0.01391300 | 1.96209200 | -0.02734100 |
| O | 2.26139300 | 2.73341500 | 0.00884800 |
| H | 1.79469200 | 3.57380900 | 0.00459300 |
| H | -0.52513400 | 1.10740000 | -0.03580700 |

**Ni[P(n-Bu)$_3$]$_2$**

| | | | |
|---|---|---|---|
| Ni | 0.00272800 | -0.52526600 | -1.30802200 |
| P | 2.04048600 | -0.29380400 | -0.73301600 |
| P | -2.02029200 | -0.29292600 | -0.69795800 |
| C | -1.98730700 | 0.39185800 | 1.03606400 |
| C | 3.49734100 | -0.52871500 | -1.87614400 |
| C | 2.27740300 | 1.42600200 | -0.05559600 |
| C | 2.43055500 | -1.40861300 | 0.70821200 |
| C | -3.07618400 | 0.92181400 | -1.64062000 |
| C | -3.09697500 | -1.81108500 | -0.56873600 |
| C | -3.17234500 | 0.21054600 | 1.97713300 |
| C | -2.93197500 | 0.85121200 | 3.33839800 |
| C | -4.09675300 | 0.66463700 | 4.29421000 |
| C | -4.18024000 | 1.70190200 | -0.93742600 |
| C | -4.61557900 | -1.69548400 | -0.50296100 |
| C | -5.29656000 | -3.05751000 | -0.44544600 |
| C | 3.67257100 | 2.01659600 | 0.11032500 |
| C | 3.63651500 | 3.44474800 | 0.63912400 |
| C | 3.41896800 | -0.96781100 | 1.78146900 |
| C | 3.54633100 | -1.98590500 | 2.90805700 |
| C | 4.88085800 | -0.85526500 | -1.32659600 |
| C | 5.92103600 | -1.02104900 | -2.42782800 |
| C | -6.81115100 | -2.96181600 | -0.40299500 |
| C | 5.01810100 | 4.05601800 | 0.78973400 |

| | | | |
|---|---|---|---|
| C | 7.29631600 | -1.37873000 | -1.89348800 |
| C | 4.50807600 | -1.54518400 | 3.99680900 |
| H | -1.74835000 | 1.45965500 | 0.92750100 |
| H | -1.09304600 | -0.05020100 | 1.49507800 |
| H | -4.08551000 | 0.63378900 | 1.54052700 |
| H | -3.37685100 | -0.86059400 | 2.11682200 |
| H | -2.73095100 | 1.92262200 | 3.19903300 |
| H | -2.01557900 | 0.43336500 | 3.77780600 |
| H | -5.01491900 | 1.10470200 | 3.88988400 |
| H | -4.29775000 | -0.39748300 | 4.47167800 |
| H | -3.48826200 | 0.36812500 | -2.49599900 |
| H | -2.36737900 | 1.63502100 | -2.08025500 |
| C | -4.89811300 | 2.66751300 | -1.87268200 |
| H | -4.91738100 | 1.02083200 | -0.49361000 |
| H | -3.75487200 | 2.26845200 | -0.09631800 |
| H | -2.72175400 | -2.37136400 | 0.29915000 |
| H | -2.83000300 | -2.42674900 | -1.43701900 |
| H | -4.92443500 | -1.10535900 | 0.36922000 |
| H | -4.98628300 | -1.14763800 | -1.38153700 |
| H | 1.73849800 | 1.44162200 | 0.90316100 |
| H | 1.69029500 | 2.07576400 | -0.71717800 |
| H | 4.27848200 | 1.40006700 | 0.78659500 |
| H | 4.19786800 | 2.00407900 | -0.85574000 |
| H | 2.74133300 | -2.37069100 | 0.27668600 |
| H | 1.45995700 | -1.60700200 | 1.18103700 |
| H | 4.41088800 | -0.78965000 | 1.34761700 |
| H | 3.10177600 | -0.00406600 | 2.20639700 |
| H | 3.54019300 | 0.37849800 | -2.49552200 |
| H | 3.19719200 | -1.32822000 | -2.56569100 |
| H | 5.21905200 | -0.07636300 | -0.63125700 |
| H | 4.83533700 | -1.78197800 | -0.73573100 |
| H | -4.93133400 | -3.60370900 | 0.43548100 |
| H | -4.98317200 | -3.65404900 | -1.31330600 |
| H | -7.14859300 | -2.39925100 | 0.47436400 |
| H | -7.20054800 | -2.44607100 | -1.28768800 |
| H | 3.11553100 | 3.45508900 | 1.60658100 |
| H | 3.02454600 | 4.06249400 | -0.03259300 |
| H | 5.63677700 | 3.47138700 | 1.47946600 |
| H | 5.54482500 | 4.08729400 | -0.17047000 |
| H | 5.97660200 | -0.09118100 | -3.01094500 |
| H | 5.58050500 | -1.79180600 | -3.13294300 |
| H | 8.03137800 | -1.49067800 | -2.69538800 |
| H | 3.87212600 | -2.94787400 | 2.48848000 |
| H | 2.55289800 | -2.17305000 | 3.33862800 |
| H | 4.58927400 | -2.28695400 | 4.79626500 |
| H | 5.51408200 | -1.38254600 | 3.59485900 |
| H | 4.18567400 | -0.60281200 | 4.45319300 |
| H | -3.90843000 | 1.13087500 | 5.26549200 |
| H | 7.66893100 | -0.60818300 | -1.20953100 |
| H | 7.27127400 | -2.32126200 | -1.33551300 |
| H | -7.28254300 | -3.94790400 | -0.36280800 |
| C | -5.97993700 | 3.47235900 | -1.17511300 |
| H | -5.33256900 | 2.10079800 | -2.70798100 |
| H | -4.16122500 | 3.34444900 | -2.32643900 |
| H | -6.48499000 | 4.15968000 | -1.85980400 |
| H | -6.74406700 | 2.81789600 | -0.74173100 |
| H | -5.56301300 | 4.06943800 | -0.35664200 |
| H | 4.97349300 | 5.07889300 | 1.17441100 |

**Ni[P(n-Bu)$_3$]$_2$-[PyrCOOPh], iso-A**

| | | | |
|---|---|---|---|
| Ni | 0.06151300 | -1.16131100 | 0.18964600 |
| P | 2.14565600 | -0.41510300 | -0.07803600 |
| P | -1.27450700 | 0.38828500 | -0.54831000 |
| C | -0.37972200 | 0.58085000 | 3.82203400 |
| C | -1.51448900 | 1.31286100 | 4.16612400 |
| C | -0.48146900 | -0.60373200 | 3.09424200 |
| C | -2.77064100 | 0.84435100 | 3.78053300 |
| C | -2.88682600 | -0.33353000 | 3.04787500 |
| C | -1.74468700 | -1.05914200 | 2.69558100 |
| O | -1.95379700 | -2.21028100 | 1.98741000 |
| C | -1.04744900 | -2.56802700 | 0.93327600 |
| O | 0.20657400 | -2.79575200 | 1.18815600 |
| C | -1.75921800 | -3.37733800 | -0.07561900 |
| H | 0.39457600 | -1.19433000 | 2.85773400 |
| H | -3.66749300 | 1.40111100 | 4.04065800 |
| H | -3.85774200 | -0.70703400 | 2.73054100 |
| H | 0.60513100 | 0.92569800 | 4.12982600 |
| H | -1.42299500 | 2.23463000 | 4.73346200 |
| C | -3.15792500 | -3.45639200 | -0.16692400 |
| N | -3.81407300 | -4.12051000 | -1.12356500 |
| C | -3.07909100 | -4.75988000 | -2.04056500 |
| C | -1.01777600 | -4.07545300 | -1.03874000 |
| C | -1.68480600 | -4.77450800 | -2.03262000 |
| H | -3.77157300 | -2.95286600 | 0.57873000 |
| H | -3.63159200 | -5.29046300 | -2.81510100 |
| H | 0.06792700 | -4.05111000 | -0.98527500 |
| H | -1.13760600 | -5.32520500 | -2.79228600 |
| C | -3.07782300 | 0.00776000 | -0.40693300 |
| C | -1.01272400 | 1.86880700 | 0.52763200 |
| C | -1.06769100 | 0.97812600 | -2.29285400 |
| C | 3.22094100 | -1.68619700 | -0.88369600 |
| C | 2.40433300 | 1.13634100 | -1.05461400 |
| C | 2.92922900 | -0.07631900 | 1.56379300 |
| C | -4.06108300 | 1.12030000 | -0.06957900 |
| H | -3.14045800 | -0.75807000 | 0.37404700 |
| H | -3.36865500 | -0.50604400 | -1.33392900 |
| C | -5.47122800 | 0.58507000 | 0.14578500 |
| H | -3.73307300 | 1.63016900 | 0.84884900 |
| H | -4.07529500 | 1.88808400 | -0.85469400 |
| C | -2.20947300 | 1.74279200 | -2.95286400 |
| H | -0.83910400 | 0.08185400 | -2.88463400 |
| H | -0.14914600 | 1.57959400 | -2.31787400 |
| C | -1.88491600 | 2.14102800 | -4.38706700 |
| H | -3.12187600 | 1.13040900 | -2.94545600 |
| H | -2.45187100 | 2.64517900 | -2.37635100 |
| C | -1.36179100 | 3.26569100 | 0.03346900 |
| H | 0.04690400 | 1.84342500 | 0.81843300 |
| H | -1.55488900 | 1.64414600 | 1.45572300 |
| C | -1.04813300 | 4.32240500 | 1.08511100 |
| H | -0.80554000 | 3.49252400 | -0.88912200 |
| H | -2.42449200 | 3.32457600 | -0.23536200 |
| C | 3.62753800 | 2.01613300 | -0.82491400 |
| H | 1.50815900 | 1.74326100 | -0.86799600 |
| H | 2.33358500 | 0.84671400 | -2.11288500 |
| C | 3.57571600 | 3.28210300 | -1.67055400 |
| H | 3.68706200 | 2.29681800 | 0.23642900 |
| H | 4.55304200 | 1.46666100 | -1.04314700 |
| C | 4.44328100 | -0.13296500 | 1.72710200 |
| H | 2.47399200 | -0.79950400 | 2.25343200 |
| H | 2.54337800 | 0.90362200 | 1.88174900 |
| C | 4.87582200 | 0.19717000 | 3.15017400 |
| H | 4.80337400 | -1.13779700 | 1.46465400 |
| H | 4.93840900 | 0.55466200 | 1.03000100 |
| C | 4.53264400 | -1.26557900 | -1.53504000 |

| | | | |
|---|---|---|---|
| H | 2.58488000 | -2.17212700 | -1.63519600 |
| H | 3.39322600 | -2.45606700 | -0.11859400 |
| C | 5.26269900 | -2.43934800 | -2.17534500 |
| H | 4.33541000 | -0.50275400 | -2.30191600 |
| H | 5.19322200 | -0.78642600 | -0.80109500 |
| C | -6.43824400 | 1.65048900 | 0.62846400 |
| H | -5.43118800 | -0.23865700 | 0.87333000 |
| H | -5.83570800 | 0.13383200 | -0.78684100 |
| H | -7.44658500 | 1.25306100 | 0.77295000 |
| H | -6.10927100 | 2.07323700 | 1.58468500 |
| H | -6.50865100 | 2.47858200 | -0.08523500 |
| C | 6.56103000 | -2.03123200 | -2.84776300 |
| H | 4.59746700 | -2.92151900 | -2.90446500 |
| H | 5.46093300 | -3.19949900 | -1.40754100 |
| H | 7.07251400 | -2.88524600 | -3.30029400 |
| H | 6.38386500 | -1.29594500 | -3.64008300 |
| H | 7.25257000 | -1.57517400 | -2.13122100 |
| C | 6.37814000 | 0.10497100 | 3.34785900 |
| H | 4.36345700 | -0.47927000 | 3.84780300 |
| H | 4.52498400 | 1.20694200 | 3.40519400 |
| H | 6.67050200 | 0.34806200 | 4.37306500 |
| H | 6.74445500 | -0.90427800 | 3.13111700 |
| H | 6.90909700 | 0.79308300 | 2.68115400 |
| C | 4.76338600 | 4.19926600 | -1.44161700 |
| H | 2.63991900 | 3.81744800 | -1.45232500 |
| H | 3.51577600 | 3.00314300 | -2.73146000 |
| H | 4.70641300 | 5.10199100 | -2.05585800 |
| H | 4.82326800 | 4.51580100 | -0.39478500 |
| H | 5.70556700 | 3.69540100 | -1.68279500 |
| C | -1.37841500 | 5.73232700 | 0.63103200 |
| H | 0.01585400 | 4.25271800 | 1.35383200 |
| H | -1.60065100 | 4.08064500 | 2.00444500 |
| H | -1.14770600 | 6.47423100 | 1.40056300 |
| H | -0.81275800 | 6.00228400 | -0.26738700 |
| H | -2.44152100 | 5.83073600 | 0.38644700 |
| C | -3.02150000 | 2.88584900 | -5.06377600 |
| H | -1.63115100 | 1.24032000 | -4.96242000 |
| H | -0.97688500 | 2.75983500 | -4.39031800 |
| H | -2.77139100 | 3.16738200 | -6.09029800 |
| H | -3.92900500 | 2.27399000 | -5.10144900 |
| H | -3.27217100 | 3.80405100 | -4.52165200 |

**$Ni[P(n\text{-}Bu)_3]_2$-[PyrCOOPh], iso-B**

| | | | |
|---|---|---|---|
| Ni | -0.20515700 | -1.21877000 | -0.31505100 |
| P | -2.21261100 | -0.49785800 | 0.24066600 |
| P | 1.02723800 | 0.58970600 | -0.57366800 |
| C | -1.33588500 | -3.78347800 | -1.08970400 |
| C | -1.24940200 | -3.79144800 | -2.46070500 |
| C | -0.30490100 | -3.23201600 | -0.26803000 |
| C | -0.07570000 | -3.30338200 | -3.09619300 |
| C | 0.97736800 | -2.83326300 | -2.35295900 |
| C | 0.89004300 | -2.73871900 | -0.92837200 |
| O | 2.20035200 | -2.79807500 | -0.31956500 |
| C | 2.37953300 | -2.23173600 | 0.88107600 |
| O | 1.47251300 | -1.84076400 | 1.60351600 |
| C | 3.80809000 | -2.03766500 | 1.20757000 |
| H | -0.27860100 | -3.48669700 | 0.78852300 |
| H | 0.00101200 | -3.32372600 | -4.18004500 |
| H | 1.90382100 | -2.51453300 | -2.82628400 |
| H | -2.20434700 | -4.21839300 | -0.59658100 |
| H | -2.05806100 | -4.20181100 | -3.05932500 |
| C | 4.84642400 | -2.36016100 | 0.32392900 |
| N | 6.13529200 | -2.11459500 | 0.57092000 |
| C | 6.43012900 | -1.52805500 | 1.73881400 |
| C | 4.14323300 | -1.42810000 | 2.42020100 |
| C | 5.47801500 | -1.16792900 | 2.69090800 |
| H | 4.61531600 | -2.82672000 | -0.63227300 |
| H | 7.48627700 | -1.33245700 | 1.91966200 |
| H | 3.35097800 | -1.16402600 | 3.11552600 |
| H | 5.78160100 | -0.69281300 | 3.61838900 |
| C | 2.74559700 | 0.42535800 | -1.26930300 |
| C | 0.32760300 | 1.97128700 | -1.59581700 |
| C | 1.26467100 | 1.29534800 | 1.13449800 |
| C | -3.25009000 | -1.62647000 | 1.28463500 |
| C | -2.34752400 | 1.14724900 | 1.09663500 |
| C | -3.16846500 | -0.32545100 | -1.33707100 |
| C | 3.86710000 | 1.27899700 | -0.69193700 |
| H | 2.65028700 | 0.59862100 | -2.35030300 |
| H | 3.01950400 | -0.63260700 | -1.17845700 |
| C | 5.19203200 | 1.05780800 | -1.41087300 |
| H | 3.60406200 | 2.34564400 | -0.72706300 |
| H | 4.00058400 | 1.03892700 | 0.37381300 |
| C | 1.24524100 | 2.80301400 | 1.35496600 |
| H | 2.18865600 | 0.85827100 | 1.53345100 |
| H | 0.46674300 | 0.83370100 | 1.72981800 |
| C | 1.07893100 | 3.16663800 | 2.82463500 |
| H | 2.16384300 | 3.26200100 | 0.96257800 |
| H | 0.41906300 | 3.26053200 | 0.78882800 |
| C | 1.22089700 | 3.12952900 | -2.02500400 |
| H | -0.55183200 | 2.35178500 | -1.05571400 |
| H | -0.08412600 | 1.48401500 | -2.49039500 |
| C | 0.45693700 | 4.18741100 | -2.81161800 |
| H | 1.68960800 | 3.60256600 | -1.15289300 |
| H | 2.05140000 | 2.75119600 | -2.63797200 |
| C | -3.52657300 | 2.07494700 | 0.83539900 |
| H | -1.42902500 | 1.68724500 | 0.83331400 |
| H | -2.24938800 | 0.94523200 | 2.17324900 |
| C | -3.32073900 | 3.44200100 | 1.47770700 |
| H | -3.66094200 | 2.20963900 | -0.24803200 |
| H | -4.46255600 | 1.63154100 | 1.20066800 |
| C | -4.69134200 | -0.37230700 | -1.33314600 |
| H | -2.78510200 | -1.13772100 | -1.97191500 |
| H | -2.81375100 | 0.60119200 | -1.81059000 |
| C | -5.27602800 | -0.24392400 | -2.73433700 |
| H | -5.03089400 | -1.31917600 | -0.88917200 |
| H | -5.10399900 | 0.42126100 | -0.69615000 |
| C | -4.46462300 | -1.07732600 | 2.02230200 |
| H | -2.55789400 | -2.07632000 | 2.00878500 |
| H | -3.54230000 | -2.45333800 | 0.62182000 |
| C | -5.19671800 | -2.15101900 | 2.81754600 |
| H | -4.15100800 | -0.27532100 | 2.70631600 |
| H | -5.16607300 | -0.60864500 | 1.32006600 |
| C | 6.34486500 | 1.80927400 | -0.77025900 |
| H | 5.08498300 | 1.35575700 | -2.46329100 |
| H | 5.41418400 | -0.01825800 | -1.42671800 |
| H | 7.28679500 | 1.64156600 | -1.30001900 |
| H | 6.15968100 | 2.88908800 | -0.75832500 |
| H | 6.49080800 | 1.49358100 | 0.26911900 |
| C | -6.38367000 | -1.60810000 | 3.59273300 |
| H | -4.48921900 | -2.63446200 | 3.50495200 |
| H | -5.52825100 | -2.94190200 | 2.13049300 |
| H | -6.89948900 | -2.39248200 | 4.15345100 |
| H | -6.07087200 | -0.84136400 | 4.30990800 |
| H | -7.11677000 | -1.14548000 | 2.92303000 |

| | | | |
|---|---|---|---|
| C | 1.34079400 | 5.32927000 | -3.27973300 |
| H | -0.35824600 | 4.57611000 | -2.18463900 |
| H | -0.03292900 | 3.71429000 | -3.67381800 |
| H | 0.77597800 | 6.08182500 | -3.83695800 |
| H | 1.81768000 | 5.83463800 | -2.43293100 |
| H | 2.14155100 | 4.96691500 | -3.93332200 |
| C | -6.79187900 | -0.32272600 | -2.75158600 |
| H | -4.85159900 | -1.03008500 | -3.37365400 |
| H | -4.94462800 | 0.70649400 | -3.17489000 |
| H | -7.19545700 | -0.22849100 | -3.76359600 |
| H | -7.14314000 | -1.27678000 | -2.34397000 |
| H | -7.23723100 | 0.47245300 | -2.14373600 |
| C | 1.04885100 | 4.66531500 | 3.06393200 |
| H | 1.88880700 | 2.70696700 | 3.40723300 |
| H | 0.14979900 | 2.70959500 | 3.19525000 |
| H | 0.92338800 | 4.90872400 | 4.12270200 |
| H | 1.97527000 | 5.14053300 | 2.72360700 |
| H | 0.22370700 | 5.13586900 | 2.51737200 |
| C | -4.47574200 | 4.39581000 | 1.23325800 |
| H | -2.38628700 | 3.87595200 | 1.09035600 |
| H | -3.15968200 | 3.31420600 | 2.55750400 |
| H | -4.30467700 | 5.37235200 | 1.69452500 |
| H | -4.63322400 | 4.56024500 | 0.16177400 |
| H | -5.41120100 | 3.99790000 | 1.64077400 |

**TS[C(acyl)-O], iso1**

| | | | |
|---|---|---|---|
| Ni | -0.32032500 | -0.97549500 | -0.78476800 |
| P | 1.83339500 | -0.25007600 | -0.66633700 |
| P | -1.52711000 | 0.80101900 | -0.44945700 |
| C | 1.90022500 | -2.58150700 | 2.70985600 |
| C | 1.97762800 | -1.76148000 | 3.83924900 |
| C | 0.77073900 | -2.59260500 | 1.89963700 |
| C | 0.87980000 | -0.95160600 | 4.14411600 |
| C | -0.25609300 | -0.94827900 | 3.34281900 |
| C | -0.35863500 | -1.76519300 | 2.17719100 |
| O | -1.42015400 | -1.73258300 | 1.42710800 |
| C | -1.41299300 | -2.42048600 | -0.90517300 |
| O | -0.31705500 | -2.93688500 | -1.18435700 |
| C | -2.70600900 | -3.09084600 | -0.96214100 |
| H | 0.72756000 | -3.24445100 | 1.03018500 |
| H | 0.91186900 | -0.30663800 | 5.02202700 |
| H | -1.10887400 | -0.31703000 | 3.59252600 |
| H | 2.74017800 | -3.22949200 | 2.45648400 |
| H | 2.86456100 | -1.75794700 | 4.46709100 |
| C | -3.74944200 | -2.81550300 | -0.06494000 |
| N | -4.95798600 | -3.37403500 | -0.15187300 |
| C | -5.16696500 | -4.24026100 | -1.15235900 |
| C | -2.93571400 | -4.02841700 | -1.97760500 |
| C | -4.19147300 | -4.60928100 | -2.07521400 |
| H | -3.56033000 | -2.15172500 | 0.77368400 |
| H | -6.16854600 | -4.66336500 | -1.21534800 |
| H | -2.13987800 | -4.27702700 | -2.67415300 |
| H | -4.41281200 | -5.33527700 | -2.85107200 |
| C | -3.27496900 | 0.51736800 | 0.02414400 |
| C | -0.73849700 | 1.67868500 | 0.96003600 |
| C | -1.58897500 | 2.00377300 | -1.85117900 |
| C | 2.88006500 | -1.73729300 | -0.98392700 |
| C | 2.32208100 | 1.02025800 | -1.91482900 |
| C | 2.39304400 | 0.44301700 | 0.95148600 |
| C | -4.26175800 | 1.67716000 | 0.00024700 |
| H | -3.21973400 | 0.04920400 | 1.01596400 |
| H | -3.63793400 | -0.27024400 | -0.64736900 |
| C | -5.63811100 | 1.24240900 | 0.48930900 |
| H | -3.90590300 | 2.51235800 | 0.61655100 |
| H | -4.34797100 | 2.07396000 | -1.02177200 |
| C | -1.68892800 | 3.48675300 | -1.51003800 |
| H | -2.42780800 | 1.69660300 | -2.48993000 |
| H | -0.68934100 | 1.82917000 | -2.45482400 |
| C | -1.67306100 | 4.36796600 | -2.75206400 |
| H | -2.59998300 | 3.68625700 | -0.93004400 |
| H | -0.85180900 | 3.77207600 | -0.85656500 |
| C | -1.53172400 | 2.58352800 | 1.89302000 |
| H | 0.11191900 | 2.22924600 | 0.53422900 |
| H | -0.29905300 | 0.87240900 | 1.56070100 |
| C | -0.64511600 | 3.08530200 | 3.02749800 |
| H | -1.96161200 | 3.43873600 | 1.35379400 |
| H | -2.38118700 | 2.02774000 | 2.31513300 |
| C | 3.61652200 | 1.80081900 | -1.71628700 |
| H | 1.48410500 | 1.73115100 | -1.95471500 |
| H | 2.31786900 | 0.51815800 | -2.89207600 |
| C | 3.82906100 | 2.83857800 | -2.81101900 |
| H | 3.59869200 | 2.30469600 | -0.73939400 |
| H | 4.47808200 | 1.12203700 | -1.68493400 |
| C | 3.82982300 | 0.21111100 | 1.40051800 |
| H | 1.72111200 | 0.02842000 | 1.71320000 |
| H | 2.17986800 | 1.52074000 | 0.91784400 |
| C | 4.11072900 | 0.92101400 | 2.71826900 |
| H | 3.99838800 | -0.86838900 | 1.52636100 |
| H | 4.54784700 | 0.55180500 | 0.64193100 |
| C | 4.29606500 | -1.58026400 | -1.52061300 |
| H | 2.29772500 | -2.35982400 | -1.67429000 |
| H | 2.87853300 | -2.28916200 | -0.03400600 |
| C | 4.97810500 | -2.92732900 | -1.72656100 |
| H | 4.27275400 | -1.03845400 | -2.47719800 |
| H | 4.90484400 | -0.96920000 | -0.84272600 |
| C | -6.66953900 | 2.35377800 | 0.42084900 |
| H | -5.54843300 | 0.87583400 | 1.52086100 |
| H | -5.97254800 | 0.37767700 | -0.10063400 |
| H | -7.64703900 | 2.02447400 | 0.78347600 |
| H | -6.36611900 | 3.21434300 | 1.02687000 |
| H | -6.80161600 | 2.71105000 | -0.60629300 |
| C | 6.37785900 | -2.79817100 | -2.29903100 |
| H | 4.35792400 | -3.54757700 | -2.38782700 |
| H | 5.01211000 | -3.46069200 | -0.76675000 |
| H | 6.85331500 | -3.77321500 | -2.43591800 |
| H | 6.36284000 | -2.29892300 | -3.27406800 |
| H | 7.02300400 | -2.20599900 | -1.64124000 |
| C | -1.39244800 | 3.93437600 | 4.03893900 |
| H | 0.19131100 | 3.65656700 | 2.60017000 |
| H | -0.18513700 | 2.21809900 | 3.52384200 |
| H | -0.73584100 | 4.28658500 | 4.83930100 |
| H | -1.83855000 | 4.81643600 | 3.56649400 |
| H | -2.20622000 | 3.36886400 | 4.50519300 |
| C | 5.47578900 | 0.58684900 | 3.29066800 |
| H | 3.32410200 | 0.64657600 | 3.43435400 |
| H | 4.01760800 | 2.00585800 | 2.56619300 |
| H | 5.66929500 | 1.11904100 | 4.22638900 |
| H | 5.56101200 | -0.48589800 | 3.49787200 |
| H | 6.27838700 | 0.84667000 | 2.59131000 |
| C | -1.73800300 | 5.84894500 | -2.42485000 |
| H | -2.51348200 | 4.08975000 | -3.40217800 |
| H | -0.76489300 | 4.15109600 | -3.33183900 |
| H | -1.72638100 | 6.46634000 | -3.32699000 |
| H | -2.64997100 | 6.09319000 | -1.86979600 |

| | | | |
|---|---|---|---|
| H | -0.88891700 | 6.15512300 | -1.80449300 |
| C | 5.10212400 | 3.64380300 | -2.62375100 |
| H | 2.95995600 | 3.51075100 | -2.84072800 |
| H | 3.84562100 | 2.33327400 | -3.78643500 |
| H | 5.23775900 | 4.38303700 | -3.41786500 |
| H | 5.09450800 | 4.18189300 | -1.66971900 |
| H | 5.98442000 | 2.99509200 | -2.62295500 |

**TS[C(aryl)-O], iso1**

| | | | |
|---|---|---|---|
| Ni | -0.04146800 | -0.83268100 | -1.11912700 |
| P | -2.04552300 | -0.42327500 | -0.19829800 |
| P | 1.08532800 | 1.00922500 | -0.69060500 |
| C | -0.54706000 | -3.15657200 | -3.44560400 |
| C | 0.02248400 | -2.76974900 | -4.64435800 |
| C | -0.01774300 | -2.69345200 | -2.21956300 |
| C | 1.16147500 | -1.93961500 | -4.64658400 |
| C | 1.71017500 | -1.46897700 | -3.46690800 |
| C | 1.06275100 | -1.77161900 | -2.25598600 |
| O | 2.50680500 | -2.23558500 | -0.93764300 |
| C | 2.00758300 | -2.32449100 | 0.23586500 |
| O | 0.81746800 | -2.08835500 | 0.56249000 |
| C | 2.96492100 | -2.71734600 | 1.31877900 |
| H | -0.31324700 | -3.16654700 | -1.28715900 |
| H | 1.61025800 | -1.64627500 | -5.59318200 |
| H | 2.58639100 | -0.82660600 | -3.47840800 |
| H | -1.36989800 | -3.86930300 | -3.43309100 |
| H | -0.37647000 | -3.13876700 | -5.58472500 |
| C | 4.23881200 | -3.21532700 | 1.02932100 |
| N | 5.13066400 | -3.57875400 | 1.95646000 |
| C | 4.76376200 | -3.43551100 | 3.23619900 |
| C | 2.60751000 | -2.58268100 | 2.66118800 |
| C | 3.52463600 | -2.94233700 | 3.63931000 |
| H | 4.53873200 | -3.32646100 | -0.01145600 |
| H | 5.50293300 | -3.73164300 | 3.97997200 |
| H | 1.62111100 | -2.19787000 | 2.90784600 |
| H | 3.28877600 | -2.84848700 | 4.69516100 |
| C | 2.69251200 | 1.29716400 | -1.54981200 |
| C | 0.10204600 | 2.56182500 | -0.92393700 |
| C | 1.56072600 | 0.94497700 | 1.10288100 |
| C | -2.92836000 | -2.01495700 | 0.13460700 |
| C | -1.98331000 | 0.41908400 | 1.45064500 |
| C | -3.19885700 | 0.59453900 | -1.22555400 |
| C | 3.73652100 | 2.20326700 | -0.90613200 |
| H | 2.45635800 | 1.63506500 | -2.56841000 |
| H | 3.11541500 | 0.28999700 | -1.66168300 |
| C | 5.02608900 | 2.25976400 | -1.71606100 |
| H | 3.35024400 | 3.22156000 | -0.77459900 |
| H | 3.96630100 | 1.83968800 | 0.10536700 |
| C | 1.55515500 | 2.22022300 | 1.93463700 |
| H | 2.55048800 | 0.46821400 | 1.13799200 |
| H | 0.88643500 | 0.20825700 | 1.55388100 |
| C | 1.70570100 | 1.92372900 | 3.42196800 |
| H | 2.35497600 | 2.90034300 | 1.60792000 |
| H | 0.61415000 | 2.77134100 | 1.77795300 |
| C | 0.78331500 | 3.92396000 | -0.96635100 |
| H | -0.67070800 | 2.55637300 | -0.14018500 |
| H | -0.44946200 | 2.40624600 | -1.86080900 |
| C | -0.22257600 | 5.05971900 | -1.10649500 |
| H | 1.38526000 | 4.08633000 | -0.06296100 |
| H | 1.48823600 | 3.95681800 | -1.80854700 |
| C | -3.18359500 | 1.20618100 | 1.96058700 |
| H | -1.12437700 | 1.10075300 | 1.40611900 |
| H | -1.68589600 | -0.35390200 | 2.17457700 |
| C | -2.86949300 | 1.93719200 | 3.26030000 |
| H | -3.49082000 | 1.94305800 | 1.20374000 |
| H | -4.05027900 | 0.54833600 | 2.10770300 |
| C | -4.70502100 | 0.44554500 | -1.04985200 |
| H | -2.93086100 | 0.37285100 | -2.26675100 |
| H | -2.89791000 | 1.64104700 | -1.07242700 |
| C | -5.48826400 | 1.35953600 | -1.98338700 |
| H | -4.99613800 | -0.59794200 | -1.23634200 |
| H | -4.99606700 | 0.65551700 | -0.01251500 |
| C | -4.02559400 | -2.07826600 | 1.19017500 |
| H | -2.13075700 | -2.71965000 | 0.40530300 |
| H | -3.30304400 | -2.36399400 | -0.83827000 |
| C | -4.60524400 | -3.48001400 | 1.33396600 |
| H | -3.62144300 | -1.75651700 | 2.16061500 |
| H | -4.83621800 | -1.37634200 | 0.95607100 |
| C | 6.08638800 | 3.13626000 | -1.07420100 |
| H | 4.79975500 | 2.62540400 | -2.72713000 |
| H | 5.41165500 | 1.23994000 | -1.84890800 |
| H | 7.00428300 | 3.16755800 | -1.66758900 |
| H | 5.73266300 | 4.16668900 | -0.95896300 |
| H | 6.35141400 | 2.77050200 | -0.07624200 |
| C | -5.67466000 | -3.56883900 | 2.40764700 |
| H | -3.79194600 | -4.18446000 | 1.55500800 |
| H | -5.01765400 | -3.79830200 | 0.36673800 |
| H | -6.08168600 | -4.57990700 | 2.49566500 |
| H | -5.27579600 | -3.28754800 | 3.38830400 |
| H | -6.51071400 | -2.89458600 | 2.19224300 |
| C | 0.43501100 | 6.42558600 | -1.18638500 |
| H | -0.91693200 | 5.02657900 | -0.25495300 |
| H | -0.83969200 | 4.88700000 | -1.99882200 |
| H | -0.30153500 | 7.22843800 | -1.27890200 |
| H | 1.03508500 | 6.62939900 | -0.29307400 |
| H | 1.10563300 | 6.49204400 | -2.04969800 |
| C | 1.70865800 | 3.17460100 | 4.28147300 |
| H | 2.62925400 | 1.35098600 | 3.58419300 |
| H | 0.88707900 | 1.25607400 | 3.73024800 |
| H | 1.80785300 | 2.93941100 | 5.34477500 |
| H | 2.53671500 | 3.83888200 | 4.01219600 |
| H | 0.78136700 | 3.74415700 | 4.15466200 |
| C | -4.04689900 | 2.73259300 | 3.79362300 |
| H | -2.01075000 | 2.60406200 | 3.09224600 |
| H | -2.53459200 | 1.20919000 | 4.01210400 |
| H | -3.79987900 | 3.25457800 | 4.72217700 |
| H | -4.37713200 | 3.48535400 | 3.06963500 |
| H | -4.90380500 | 2.08211800 | 3.99864400 |
| C | -6.99120400 | 1.20146800 | -1.83899700 |
| H | -5.18743700 | 1.15883800 | -3.02055600 |
| H | -5.20059200 | 2.40183400 | -1.78783500 |
| H | -7.53837200 | 1.86641800 | -2.51286000 |
| H | -7.30458400 | 0.17575500 | -2.06135100 |
| H | -7.31703000 | 1.42595200 | -0.81753800 |

**Ni[P(n-Bu)$_3$]$_2$[PyrCO][OPh], Product A**

| | | | |
|---|---|---|---|
| Ni | 0.03695800 | -0.83413600 | -0.59701200 |
| P | 1.81288700 | 0.25929700 | 0.06114500 |
| P | -1.60915400 | 0.82392300 | -0.28659100 |
| C | -0.95392600 | 2.53863300 | -0.62088300 |
| C | 3.21835300 | -0.78957500 | 0.63838600 |
| C | 2.48987500 | 1.21300500 | -1.36402900 |
| C | 1.51064100 | 1.42061200 | 1.47533600 |
| C | -2.84766000 | 0.61821200 | -1.63309600 |

| | | | |
|---|---|---|---|
| C | -2.66285700 | 0.97558600 | 1.24352100 |
| C | -1.50831300 | 3.72706900 | 0.15034900 |
| C | -0.78332700 | 5.01913800 | -0.20351800 |
| C | -1.23648500 | 6.20398900 | 0.62968000 |
| C | -3.77224700 | 1.80224700 | -1.89115400 |
| C | -4.00175400 | 0.24035500 | 1.20955800 |
| C | -4.65861000 | 0.18777900 | 2.58226300 |
| C | 3.94431900 | 1.66914500 | -1.37019100 |
| C | 4.29857400 | 2.40576400 | -2.65638100 |
| C | 2.22234500 | 2.76862500 | 1.45717900 |
| C | 1.68478000 | 3.72379300 | 2.51480000 |
| C | 4.29115000 | -0.14462300 | 1.50875400 |
| C | 5.43741600 | -1.10170100 | 1.81099400 |
| C | -6.01707700 | -0.48750200 | 2.54462800 |
| C | 5.75087500 | 2.84493500 | -2.70019500 |
| C | 6.48810000 | -0.49160700 | 2.72054300 |
| C | 2.35538100 | 5.08477300 | 2.47657500 |
| H | -1.07281300 | 2.69950200 | -1.70053200 |
| H | 0.12882600 | 2.50905300 | -0.46161000 |
| H | -2.58708500 | 3.83939800 | -0.02783800 |
| H | -1.40250900 | 3.55112900 | 1.23134000 |
| H | -0.91976000 | 5.23234200 | -1.27226800 |
| H | 0.29803400 | 4.86136700 | -0.07026800 |
| H | -2.30290600 | 6.40729900 | 0.48604500 |
| H | -1.08304700 | 6.01773900 | 1.69888200 |
| H | -3.41924700 | -0.28384700 | -1.38048600 |
| H | -2.28792400 | 0.35411800 | -2.53830000 |
| C | -4.94803900 | 1.44061400 | -2.78923000 |
| H | -4.15434100 | 2.20367300 | -0.94106400 |
| H | -3.20751800 | 2.62748600 | -2.34798100 |
| H | -2.84078300 | 2.03412200 | 1.46650800 |
| H | -2.06001100 | 0.58182300 | 2.07289100 |
| H | -4.68260400 | 0.73626400 | 0.50405900 |
| H | -3.87833900 | -0.78549500 | 0.84258000 |
| H | 1.82380500 | 2.07154800 | -1.52138300 |
| H | 2.30796400 | 0.54669100 | -2.21842400 |
| H | 4.15370900 | 2.32375100 | -0.51352100 |
| H | 4.60947300 | 0.80136100 | -1.25951700 |
| H | 1.75400200 | 0.86602900 | 2.39042700 |
| H | 0.42664600 | 1.58300900 | 1.52429400 |
| H | 3.30284400 | 2.62840900 | 1.59732700 |
| H | 2.11087000 | 3.23764100 | 0.46725400 |
| H | 3.66262300 | -1.22376300 | -0.26867200 |
| H | 2.77427100 | -1.63839600 | 1.17399100 |
| H | 4.69270000 | 0.75770100 | 1.03075900 |
| H | 3.84776800 | 0.19377800 | 2.45564200 |
| H | -4.75104300 | 1.20483300 | 2.98956500 |
| H | -3.99273000 | -0.35485200 | 3.26787600 |
| H | -6.71288600 | 0.05464500 | 1.89459200 |
| H | -5.92745800 | -1.50715500 | 2.15323800 |
| H | 3.63900100 | 3.27789600 | -2.76229000 |
| H | 4.07491900 | 1.75823400 | -3.51443000 |
| H | 5.98846900 | 3.51566100 | -1.86742800 |
| H | 6.42651000 | 1.98595600 | -2.62814400 |
| H | 5.89749800 | -1.41734000 | 0.86463800 |
| H | 5.03291900 | -2.01685000 | 2.26398300 |
| H | 7.30587600 | -1.18894300 | 2.92095900 |
| H | 1.80199300 | 3.27134500 | 3.50844700 |
| H | 0.60062600 | 3.83836400 | 2.36764000 |
| H | 1.94400300 | 5.76139400 | 3.23052100 |
| H | 3.43254800 | 5.00348100 | 2.65621900 |
| H | 2.22414400 | 5.56324700 | 1.49914800 |
| H | -0.68890300 | 7.11496500 | 0.37252500 |
| H | 6.92438500 | 0.40932400 | 2.27556600 |
| H | 6.05790900 | -0.20190500 | 3.68529600 |
| H | -6.47211900 | -0.54886500 | 3.53743600 |
| C | -5.85676000 | 2.62205800 | -3.07598400 |
| H | -5.52024700 | 0.63244200 | -2.31351900 |
| H | -4.56753500 | 1.02039100 | -3.72978500 |
| H | -6.69893300 | 2.34388000 | -3.71551800 |
| H | -6.26919300 | 3.03741400 | -2.14995400 |
| H | -5.31270200 | 3.42864200 | -3.57920600 |
| H | 5.98863600 | 3.37393100 | -3.62712300 |
| O | -1.35032100 | -2.10333100 | -1.08888200 |
| C | -2.09623300 | -2.52361900 | -0.09024400 |
| C | -3.29915000 | -3.22494500 | -0.35451600 |
| C | -4.12126900 | -3.65510500 | 0.67829100 |
| C | -3.79006700 | -3.41684400 | 2.01567900 |
| C | -1.77913800 | -2.29186200 | 1.27404400 |
| C | -2.60957600 | -2.73176400 | 2.29920500 |
| H | -3.56389000 | -3.40941800 | -1.39309700 |
| H | -5.04070500 | -4.18557000 | 0.43730400 |
| H | -4.43999300 | -3.75762900 | 2.81667500 |
| H | -0.87264200 | -1.72593700 | 1.50201400 |
| H | -2.33304500 | -2.52853800 | 3.33241100 |
| C | 1.17911400 | -2.01812400 | -1.52724600 |
| C | 1.47220100 | -3.39211700 | -1.03752100 |
| O | 1.54020100 | -1.66359600 | -2.64357700 |
| C | 1.19074600 | -3.77238600 | 0.27648500 |
| N | 1.45366300 | -4.97910100 | 0.78382300 |
| C | 2.01379300 | -5.86866500 | -0.04613700 |
| C | 2.06399800 | -4.33779400 | -1.88012100 |
| C | 2.33492200 | -5.60102600 | -1.37705600 |
| H | 0.72188500 | -3.04897200 | 0.94643300 |
| H | 2.21742900 | -6.85130500 | 0.37781700 |
| H | 2.29330700 | -4.06182700 | -2.90612600 |
| H | 2.78726400 | -6.37046200 | -1.99551300 |

**Ni[P(n-Bu)$_3$]$_2$[PyrCOO][Ph], Product B**

| | | | |
|---|---|---|---|
| Ni | -0.07321600 | -1.00421400 | -0.81864400 |
| P | -0.95153600 | 1.15454900 | -0.70773900 |
| P | 1.99618900 | -0.51443500 | -0.31690100 |
| C | 0.97535100 | -4.95548100 | 0.17400100 |
| C | 1.26898800 | -5.52870400 | -1.06145200 |
| C | 0.59278900 | -3.61444600 | 0.25555900 |
| C | 1.16645000 | -4.75410400 | -2.21643500 |
| C | 0.79507500 | -3.41140500 | -2.13110700 |
| C | 0.51086600 | -2.81960600 | -0.89418400 |
| O | -1.76002900 | -1.24688400 | 1.25200200 |
| C | -2.38089300 | -1.65434100 | 0.24758100 |
| O | -1.89605300 | -1.71289500 | -0.94280800 |
| C | -3.81970500 | -2.04592300 | 0.36891400 |
| H | 0.34696700 | -3.18741500 | 1.22750300 |
| H | 1.38153700 | -5.19557500 | -3.18788100 |
| H | 0.74113600 | -2.81857300 | -3.04392400 |
| H | 1.03831800 | -5.55534800 | 1.07999000 |
| H | 1.56966600 | -6.57134000 | -1.12490600 |
| C | -4.47008400 | -1.95315600 | 1.60273400 |
| N | -5.76751600 | -2.21426600 | 1.79179700 |
| C | -6.46891800 | -2.59115600 | 0.71481600 |
| C | -4.57299800 | -2.44943400 | -0.73484300 |
| C | -5.92232600 | -2.72790400 | -0.55979700 |
| H | -3.89539000 | -1.63812700 | 2.47329100 |
| H | -7.52617000 | -2.79528400 | 0.88239800 |

| | | | |
|---|---|---|---|
| H | -4.09178700 | -2.52648800 | -1.70553200 |
| H | -6.54509700 | -3.04034000 | -1.39257200 |
| C | 3.40235400 | -1.61056000 | -0.80038500 |
| C | 2.63565000 | 1.16045900 | -0.77505200 |
| C | 1.88015300 | -0.57285700 | 1.53178800 |
| C | -2.80432400 | 1.25548400 | -0.80189200 |
| C | -0.52551900 | 1.99980800 | 0.87902100 |
| C | -0.37138200 | 2.33178900 | -2.02452400 |
| C | 4.51649700 | -1.80372800 | 0.22227500 |
| H | 3.80672900 | -1.19738400 | -1.73432500 |
| H | 2.98051500 | -2.58453400 | -1.06172400 |
| C | 5.60984200 | -2.72825100 | -0.29705200 |
| H | 4.96024400 | -0.84118900 | 0.50931900 |
| H | 4.09819400 | -2.22882400 | 1.14542200 |
| C | 2.67900000 | 0.42756300 | 2.36017200 |
| H | 2.12077000 | -1.60165900 | 1.82541900 |
| H | 0.80754900 | -0.46732100 | 1.74735000 |
| C | 2.20157600 | 0.48443200 | 3.80542700 |
| H | 3.74940600 | 0.18010700 | 2.33083700 |
| H | 2.59651200 | 1.43665100 | 1.92681300 |
| C | 4.10780200 | 1.49419600 | -0.56681600 |
| H | 2.01756400 | 1.90206800 | -0.25328600 |
| H | 2.38759000 | 1.27703500 | -1.83827700 |
| C | 4.40480700 | 2.94563000 | -0.92563100 |
| H | 4.40484600 | 1.31691700 | 0.47410400 |
| H | 4.73537800 | 0.82933500 | -1.17602900 |
| C | -0.61242500 | 3.51637700 | 0.97062000 |
| H | 0.49608200 | 1.69125800 | 1.12861800 |
| H | -1.15150800 | 1.51767100 | 1.64110700 |
| C | -0.07772800 | 4.03542800 | 2.29996900 |
| H | -0.03567100 | 3.97741100 | 0.15443000 |
| H | -1.64973300 | 3.85329500 | 0.83044900 |
| C | -1.27100200 | 3.49683400 | -2.42467000 |
| H | -0.12849000 | 1.73085900 | -2.90929100 |
| H | 0.59105000 | 2.72404900 | -1.67270900 |
| C | -0.52386300 | 4.54704000 | -3.23677000 |
| H | -2.12092800 | 3.12737600 | -3.01282700 |
| H | -1.70963600 | 3.97081700 | -1.53560500 |
| C | -3.55749300 | 1.41209800 | 0.51726600 |
| H | -3.13169700 | 0.33769000 | -1.30455000 |
| H | -3.07390800 | 2.08377000 | -1.46502300 |
| C | -5.06606300 | 1.33064100 | 0.31992200 |
| H | -3.24238800 | 0.64154800 | 1.23293800 |
| H | -3.30674300 | 2.37848600 | 0.97887300 |
| C | 6.71775900 | -2.95606400 | 0.71525600 |
| H | 6.02790700 | -2.30766400 | -1.22214900 |
| H | 5.15802700 | -3.68777700 | -0.58389900 |
| H | 7.49344800 | -3.62133600 | 0.32653400 |
| H | 7.20078600 | -2.01305700 | 0.99378200 |
| H | 6.32823300 | -3.40552500 | 1.63496900 |
| C | -5.83679000 | 1.47168600 | 1.62004800 |
| H | -5.31169600 | 0.37001300 | -0.15467500 |
| H | -5.38317900 | 2.10467100 | -0.39307700 |
| H | -6.91652300 | 1.39562000 | 1.46210300 |
| H | -5.55584500 | 0.68669900 | 2.33045900 |
| H | -5.63904400 | 2.43720600 | 2.09908900 |
| C | 5.86877800 | 3.30714700 | -0.75522200 |
| H | 3.77923600 | 3.59857600 | -0.29934200 |
| H | 4.08700600 | 3.13526700 | -1.96014200 |
| H | 6.06198300 | 4.35370500 | -1.00539600 |
| H | 6.19797800 | 3.14779300 | 0.27708700 |
| H | 6.50612200 | 2.69101500 | -1.39808200 |
| C | 2.97794000 | 1.48568000 | 4.64118200 |
| H | 2.27117800 | -0.51611300 | 4.25212300 |
| H | 1.13189600 | 0.73893200 | 3.81181500 |
| H | 2.62138800 | 1.51730100 | 5.67418300 |
| H | 4.04479200 | 1.23920000 | 4.66764700 |
| H | 2.88817700 | 2.49757200 | 4.23006700 |
| C | -0.15871100 | 5.54597400 | 2.42208600 |
| H | 0.96572300 | 3.70366500 | 2.41202800 |
| H | -0.62835000 | 3.55952300 | 3.12285600 |
| H | 0.24121000 | 5.90062300 | 3.37579500 |
| H | 0.40577000 | 6.03900900 | 1.62326500 |
| H | -1.19468600 | 5.89348700 | 2.34925900 |
| C | -1.41786000 | 5.68708600 | -3.68944100 |
| H | -0.05789900 | 4.06645800 | -4.10790700 |
| H | 0.30821500 | 4.93786700 | -2.63405000 |
| H | -0.86559600 | 6.43438100 | -4.26573600 |
| H | -2.23564800 | 5.32213000 | -4.31984200 |
| H | -1.87156000 | 6.19880800 | -2.83385900 |

**Ni[P(n-Bu)$_3$]$_2$[Na$_2$CO$_3$], lowest isomer, iso1**

| | | | |
|---|---|---|---|
| Ni | 0.04713300 | -1.37872100 | -1.01945400 |
| P | -1.44217100 | 0.13319900 | -0.60644100 |
| P | 1.98953400 | -0.71812300 | -0.55904300 |
| O | -0.94385000 | -3.16468200 | -1.42273300 |
| C | -1.90103400 | -3.31016100 | -0.54255500 |
| O | -3.09339500 | -2.90939500 | -0.75730500 |
| O | -1.60207300 | -3.91357500 | 0.58764800 |
| Na | -3.73013400 | -3.69047500 | 1.2156570 |
| Na | 0.56195500 | -4.06492600 | -0.0386550 |
| C | -2.98401200 | 0.29028700 | -1.63937000 |
| C | -2.08456400 | -0.22229400 | 1.11142700 |
| C | -0.86330700 | 1.90687500 | -0.49263800 |
| C | 3.65023700 | -1.61488200 | -0.58889600 |
| C | 2.41163200 | 0.79511400 | -1.59043900 |
| C | 1.95465900 | -0.08810500 | 1.20989300 |
| C | -1.45708000 | 2.86870400 | 0.52873900 |
| C | -0.70145300 | 4.19180400 | 0.57511300 |
| C | -3.84516000 | 1.54548100 | -1.58234200 |
| C | -5.05644500 | 1.46923900 | -2.50452600 |
| C | 3.21849600 | 0.23886100 | 1.99568800 |
| C | 2.90738400 | 0.72597300 | 3.40603500 |
| C | 3.02834800 | 2.02247300 | -0.92958500 |
| C | 3.00412600 | 3.24790100 | -1.83478100 |
| C | 4.97057000 | -0.85554000 | -0.67702900 |
| C | 6.18236900 | -1.77988600 | -0.68268000 |
| C | -5.90598400 | 2.72729600 | -2.47402700 |
| C | 7.50017600 | -1.03499600 | -0.80245100 |
| C | 4.15230000 | 1.04151900 | 4.21605400 |
| C | -1.26046900 | 5.16612200 | 1.59626600 |
| C | 3.60338900 | 4.48084900 | -1.18246500 |
| C | -3.47639700 | 0.23182600 | 1.52771600 |
| C | -3.84898900 | -0.21802900 | 2.93519200 |
| C | -5.25485400 | 0.18808200 | 3.33985800 |
| H | -0.95623500 | 2.32751600 | -1.50515000 |
| H | 0.21778200 | 1.84601700 | -0.31498900 |
| H | -2.51834800 | 3.05864100 | 0.31720500 |
| H | -1.42948500 | 2.41333500 | 1.53033200 |
| H | -3.55992000 | -0.61611100 | -1.40990600 |
| H | -2.64319500 | 0.14472300 | -2.67346400 |
| H | -4.19220800 | 1.73777200 | -0.55874600 |
| H | -3.24343500 | 2.42481500 | -1.85743800 |
| H | 1.38637100 | -0.85335700 | 1.75768400 |

| | | | |
|---|---|---|---|
| H | 1.28988100 | 0.78837500 | 1.20475600 |
| H | 3.86808300 | -0.64703100 | 2.05615300 |
| H | 3.80923200 | 1.00663400 | 1.47957400 |
| H | 1.46319300 | 1.08582700 | -2.05883700 |
| H | 3.04865600 | 0.45271900 | -2.41802500 |
| H | 2.48536500 | 2.26738500 | -0.00339000 |
| H | 4.06205600 | 1.81455700 | -0.61861800 |
| H | 3.58888400 | -2.30113900 | -1.44493400 |
| H | 3.65257000 | -2.26635800 | 0.29985400 |
| H | 4.98305300 | -0.24413100 | -1.59075300 |
| H | 5.07050100 | -0.14457800 | 0.15253700 |
| H | -5.66538400 | 0.59882000 | -2.22290700 |
| H | -4.71736400 | 1.27037800 | -3.53053200 |
| H | -6.77426000 | 2.65452400 | -3.13556700 |
| H | -6.27799800 | 2.92943600 | -1.46327300 |
| H | -5.32721000 | 3.60380500 | -2.78565300 |
| H | 6.08356900 | -2.50004900 | -1.50707600 |
| H | 6.17656100 | -2.38217600 | 0.23711200 |
| H | 8.35815600 | -1.71360100 | -0.80220500 |
| H | 7.54198400 | -0.45140300 | -1.72856200 |
| H | 7.63507000 | -0.33289000 | 0.02776800 |
| H | 2.30352300 | -0.03155200 | 3.92521400 |
| H | 2.26745500 | 1.61774300 | 3.34007500 |
| H | 3.91004400 | 1.39150300 | 5.22378700 |
| H | 4.79199700 | 0.15803200 | 4.31857600 |
| H | 4.75187400 | 1.82001300 | 3.73191400 |
| H | -0.71119800 | 4.64892000 | -0.42460400 |
| H | 0.35758400 | 3.98550000 | 0.79288100 |
| H | -0.69980100 | 6.10509700 | 1.61838400 |
| H | -2.30566300 | 5.41152200 | 1.37881200 |
| H | -1.23146000 | 4.74103500 | 2.60571200 |
| H | 1.96248800 | 3.44851400 | -2.12569100 |
| H | 3.53450000 | 3.02055200 | -2.76987600 |
| H | 3.57368300 | 5.35163900 | -1.84374200 |
| H | 3.06254700 | 4.74430600 | -0.26637200 |
| H | 4.64971300 | 4.31392900 | -0.90364200 |
| H | -1.33126300 | 0.17100200 | 1.80958700 |
| H | -2.01788600 | -1.31582500 | 1.20010900 |
| H | -3.55818100 | 1.32569400 | 1.47416000 |
| H | -4.21682400 | -0.15848200 | 0.81312700 |
| H | -3.12051300 | 0.19044900 | 3.64922300 |
| H | -3.73770700 | -1.31131000 | 3.00914800 |
| H | -5.50596100 | -0.14216100 | 4.35217200 |
| H | -5.37257200 | 1.27645300 | 3.31024600 |
| H | -6.00158400 | -0.23342700 | 2.65771400 |

**Decarbonylative transmetalation in the absence of $Na_2CO3$**

For Product-A and $P(n\text{-}Bu)_3$, see above

**Ni[P(n-Bu)$_3$][PyrCO][OPh]**

| | | | |
|---|---|---|---|
| Ni | -1.21202600 | 0.83229700 | -1.12799600 |
| P | 0.59344500 | -0.19655900 | -0.50374500 |
| C | 0.63229700 | -2.03615000 | -0.49643600 |
| C | 2.06245800 | 0.36058700 | -1.46750500 |
| C | 0.84704800 | 0.34153800 | 1.24325700 |
| C | 1.58917600 | -2.73348100 | 0.46434100 |
| C | 1.50723800 | -4.25032500 | 0.34945100 |
| C | 2.44139300 | -4.96348100 | 1.30990100 |
| C | 3.36421800 | -0.42650300 | -1.37815800 |
| C | 2.26470200 | 0.46003300 | 1.78802500 |
| C | 2.27763300 | 0.98189800 | 3.21924900 |
| C | 3.68009100 | 1.12859200 | 3.78059500 |
| H | 0.81992900 | -2.34821800 | -1.53266100 |
| H | -0.39424000 | -2.35936800 | -0.27900700 |
| H | 2.62351900 | -2.41372600 | 0.28577900 |
| H | 1.35748900 | -2.43506600 | 1.49663100 |
| H | 1.73610600 | -4.54337700 | -0.68412000 |
| H | 0.47105900 | -4.56862500 | 0.52571800 |
| H | 3.48483400 | -4.68384400 | 1.12910100 |
| H | 2.21179700 | -4.70828200 | 2.34985500 |
| H | 2.22034300 | 1.40723800 | -1.17429600 |
| H | 1.73068700 | 0.40206400 | -2.51250900 |
| C | 4.47669500 | 0.22239200 | -2.19211800 |
| H | 3.68931200 | -0.51805200 | -0.33412000 |
| H | 3.20210700 | -1.45300400 | -1.73569200 |
| H | 0.24885800 | -0.34202500 | 1.86180400 |
| H | 0.34772100 | 1.31766500 | 1.32061800 |
| H | 2.77769800 | -0.51053600 | 1.74717900 |
| H | 2.85097500 | 1.13936100 | 1.15146600 |
| H | 1.68797100 | 0.30570900 | 3.85283200 |
| H | 1.75481000 | 1.94795900 | 3.25000600 |
| H | 4.20769100 | 0.16872800 | 3.78531800 |
| H | 4.27690100 | 1.82429500 | 3.18108300 |
| H | 2.37057100 | -6.05017200 | 1.21403600 |
| H | 3.66964200 | 1.50339700 | 4.80759300 |
| C | 5.78003000 | -0.55296900 | -2.12965000 |
| H | 4.63269300 | 1.24718200 | -1.82793400 |
| H | 4.14856600 | 0.32441600 | -3.23522700 |
| H | 6.56841800 | -0.07042900 | -2.71347200 |
| H | 6.13990000 | -0.64064000 | -1.09888000 |
| H | 5.65659400 | -1.56925800 | -2.51858800 |
| O | -2.64422400 | 2.12467200 | -1.19054500 |
| C | -1.73914900 | 2.82415700 | -0.53698700 |
| C | -0.44092600 | 2.99664500 | -1.11708500 |
| C | 0.59542200 | 3.60440700 | -0.38958400 |
| C | 0.37559200 | 4.05673800 | 0.89995200 |
| C | -1.93691100 | 3.31072900 | 0.78682400 |
| C | -0.90329000 | 3.91653100 | 1.47286000 |
| H | -0.31182000 | 2.76013200 | -2.17371500 |
| H | 1.56800200 | 3.74075200 | -0.85808500 |
| H | 1.17474600 | 4.53114100 | 1.46168600 |
| H | -2.91787800 | 3.18531200 | 1.23675000 |
| H | -1.07696600 | 4.28178000 | 2.48258700 |
| C | -2.19329700 | -0.70247100 | -1.48168600 |
| C | -3.03540700 | -1.21247900 | -0.35736700 |
| O | -2.19786200 | -1.24863300 | -2.57267400 |
| C | -2.96149300 | -0.64743300 | 0.91921100 |
| N | -3.67725400 | -1.06116900 | 1.96714100 |
| C | -4.52221500 | -2.07796400 | 1.75159300 |
| C | -3.92132300 | -2.27615600 | -0.55336500 |
| C | -4.68208500 | -2.71389400 | 0.52046200 |
| H | -2.27765000 | 0.18899300 | 1.09013200 |
| H | -5.10513700 | -2.40214500 | 2.61259000 |
| H | -3.99263300 | -2.72847200 | -1.53906900 |
| H | -5.38748300 | -3.53259800 | 0.41573600 |

**TS[(OC-C(Pyr)], Decarbonylation TS**

| | | | |
|---|---|---|---|
| Ni | 1.35632800 | -0.71440400 | 0.05261100 |
| P | -0.85115600 | -0.63908200 | -0.15920300 |
| C | -1.55658700 | -2.30126100 | -0.53028900 |
| C | -1.54964700 | -0.16117300 | 1.46810900 |
| C | -1.57361500 | 0.52671000 | -1.40131700 |

| | | | |
|---|---|---|---|
| C | -2.96111900 | -2.38553700 | -1.12063000 |
| C | -3.52645700 | -3.79747700 | -1.04206100 |
| C | -4.89891900 | -3.91895700 | -1.67879700 |
| C | -3.03084200 | -0.41783400 | 1.71320000 |
| C | -2.05111200 | 1.87185600 | -0.86090600 |
| C | -2.45690300 | 2.81824900 | -1.98211900 |
| C | -2.98403400 | 4.14082400 | -1.45699900 |
| H | -1.51084100 | -2.84547100 | 0.42366000 |
| H | -0.84646400 | -2.81571800 | -1.18744500 |
| H | -3.64499500 | -1.69343800 | -0.61199100 |
| H | -2.93865600 | -2.06387200 | -2.17002900 |
| H | -3.57290200 | -4.10322900 | 0.01237400 |
| H | -2.82591800 | -4.49394700 | -1.52191200 |
| H | -5.61959700 | -3.24867300 | -1.19822500 |
| H | -4.86834500 | -3.65511700 | -2.74114900 |
| H | -1.29809600 | 0.90070800 | 1.59311800 |
| H | -0.94571600 | -0.68513700 | 2.21886000 |
| C | -3.49608300 | 0.13959100 | 3.05191100 |
| H | -3.63727300 | 0.02193200 | 0.90885700 |
| H | -3.23150600 | -1.49871500 | 1.67866600 |
| H | -2.40166200 | 0.01835200 | -1.90964000 |
| H | -0.79961600 | 0.69436600 | -2.15997300 |
| H | -2.90447800 | 1.72374200 | -0.18427100 |
| H | -1.26516100 | 2.34961700 | -0.26382500 |
| H | -3.21027400 | 2.33504800 | -2.62029400 |
| H | -1.58220900 | 2.99495900 | -2.62317600 |
| H | -3.87821400 | 3.99693000 | -0.84044300 |
| H | -2.22987000 | 4.63446600 | -0.83405700 |
| H | -5.29332200 | -4.93578500 | -1.60313200 |
| H | -3.24871400 | 4.82563500 | -2.26771700 |
| C | -4.96112900 | -0.14367300 | 3.32971300 |
| H | -3.31183900 | 1.22219700 | 3.06991000 |
| H | -2.87292300 | -0.28115800 | 3.85235900 |
| H | -5.27837000 | 0.26329100 | 4.29353900 |
| H | -5.60334000 | 0.29596600 | 2.55898600 |
| H | -5.16124500 | -1.22049600 | 3.34400300 |
| O | 1.48096200 | 0.85347200 | 1.12714500 |
| C | 1.23956200 | 2.01316800 | 0.55274600 |
| C | 0.82843000 | 3.12784100 | 1.32167600 |
| C | 0.53493500 | 4.34350200 | 0.71605200 |
| C | 0.64012300 | 4.50637700 | -0.66787900 |
| C | 1.36198400 | 2.20417600 | -0.84603900 |
| C | 1.06024200 | 3.42355700 | -1.44074200 |
| H | 0.73540500 | 3.00207300 | 2.39791700 |
| H | 0.21153200 | 5.17935900 | 1.33344000 |
| H | 0.40383800 | 5.45948200 | -1.13250600 |
| H | 1.69853800 | 1.35966900 | -1.45103300 |
| H | 1.15665100 | 3.53061000 | -2.51964400 |
| C | 3.28759700 | -1.29463200 | 0.03820600 |
| C | 1.86239000 | -2.17704800 | -0.68819300 |
| O | 1.98802700 | -3.20358300 | -1.25322900 |
| C | 3.81950400 | -1.76865900 | 1.23994200 |
| C | 5.17007100 | -1.56705800 | 1.49471900 |
| C | 5.93529200 | -0.91102000 | 0.53264500 |
| C | 4.15160900 | -0.66780100 | -0.86687500 |
| N | 5.45134800 | -0.46666700 | -0.63420600 |
| H | 3.18696200 | -2.27741100 | 1.96334800 |
| H | 5.62429200 | -1.90861600 | 2.41981200 |
| H | 6.99616900 | -0.73522500 | 0.70434200 |
| H | 3.77522500 | -0.31162200 | -1.82613100 |

**Ni[P(n-Bu)$_3$](Pyr)(CO)(OPh),**

**Decarb. Product**

| | | | |
|---|---|---|---|
| Ni | 1.35756800 | -0.98887000 | 0.07472000 |
| P | -0.94698200 | -0.69069400 | -0.13422900 |
| C | -1.90862700 | -2.25229100 | -0.34407200 |
| C | -1.54666900 | 0.03709200 | 1.43900600 |
| C | -1.48948300 | 0.44502800 | -1.49431700 |
| C | -3.31865100 | -2.16485800 | -0.92062500 |
| C | -4.10336200 | -3.45332800 | -0.71111900 |
| C | -5.48754400 | -3.40587900 | -1.33208000 |
| C | -3.04009300 | -0.00969800 | 1.73686200 |
| C | -1.84146200 | 1.87149700 | -1.08147300 |
| C | -2.11448500 | 2.75840600 | -2.28838300 |
| C | -2.50139100 | 4.17043200 | -1.88921000 |
| H | -1.93291400 | -2.70622800 | 0.65630700 |
| H | -1.30908300 | -2.93651800 | -0.95520100 |
| H | -3.87331500 | -1.32747100 | -0.47848300 |
| H | -3.26298900 | -1.94898000 | -1.99557700 |
| H | -4.18137700 | -3.65106900 | 0.36703100 |
| H | -3.53338900 | -4.29508800 | -1.12673900 |
| H | -6.08334400 | -2.58924400 | -0.91054600 |
| H | -5.43200700 | -3.24347200 | -2.41364000 |
| H | -1.17497900 | 1.06955900 | 1.44610700 |
| H | -0.98358800 | -0.46789000 | 2.23303800 |
| C | -3.38868300 | 0.74308800 | 3.01434000 |
| H | -3.61470800 | 0.41592500 | 0.90231700 |
| H | -3.37501200 | -1.05328700 | 1.82631700 |
| H | -2.34542200 | -0.01228700 | -2.00496400 |
| H | -0.67292300 | 0.47030900 | -2.22605400 |
| H | -2.72422300 | 1.86481400 | -0.42725200 |
| H | -1.02883500 | 2.31824700 | -0.49581000 |
| H | -2.90308100 | 2.30876100 | -2.90805500 |
| H | -1.21290300 | 2.78306600 | -2.91624100 |
| H | -3.41587900 | 4.17737100 | -1.28608600 |
| H | -1.70859100 | 4.63247900 | -1.29027700 |
| H | -6.03864200 | -4.33548400 | -1.16673800 |
| H | -2.67613900 | 4.80728900 | -2.76086800 |
| C | -4.86927400 | 0.68738500 | 3.34357200 |
| H | -3.06463100 | 1.78745300 | 2.90958000 |
| H | -2.80203500 | 0.33180200 | 3.84663800 |
| H | -5.10386100 | 1.23996400 | 4.25730700 |
| H | -5.47136700 | 1.11621200 | 2.53526100 |
| H | -5.20600900 | -0.34504100 | 3.48696400 |
| O | 1.40919500 | 0.57446600 | 1.12683200 |
| C | 1.34641500 | 1.73563300 | 0.49963500 |
| C | 0.99233000 | 2.89786500 | 1.22225400 |
| C | 0.87502100 | 4.12427500 | 0.58043600 |
| C | 1.10703800 | 4.24496600 | -0.79180000 |
| C | 1.59702800 | 1.87568900 | -0.88431500 |
| C | 1.47337100 | 3.10963300 | -1.51407800 |
| H | 0.80369000 | 2.80165100 | 2.28884800 |
| H | 0.59163000 | 5.00029500 | 1.15978400 |
| H | 1.00781700 | 5.20682900 | -1.28618200 |
| H | 1.89124300 | 0.98948500 | -1.44942900 |
| H | 1.66598400 | 3.18357300 | -2.58249400 |
| C | 3.28859800 | -1.11111600 | 0.23716500 |
| C | 1.39429800 | -2.54765400 | -0.68906200 |
| O | 1.42307600 | -3.58932800 | -1.18734200 |
| C | 3.86724000 | -0.83386000 | 1.47565300 |
| C | 5.25460400 | -0.82436900 | 1.58749600 |
| C | 6.01459800 | -1.08061500 | 0.44869000 |
| C | 4.14596700 | -1.37126400 | -0.83373300 |
| N | 5.48465200 | -1.34526400 | -0.74874900 |

| | | | |
|---|---|---|---|
| H | 3.24291500 | -0.59788300 | 2.33332300 |
| H | 5.73950700 | -0.61197800 | 2.53693000 |
| H | 7.10311400 | -1.07433800 | 0.49997000 |
| H | 3.74670700 | -1.60064400 | -1.82352200 |

**Ni[P(n-Bu)$_3$](Pyr)(CO)(OPh)[MeOPh-B(OH)$_2$] Iso-1**

| | | | |
|---|---|---|---|
| Ni | 1.23404700 | -0.49006600 | 1.05678100 |
| O | 1.39165100 | -0.98125800 | -0.77641900 |
| B | -1.72558300 | -1.52491400 | -0.97988500 |
| C | -3.26610000 | -1.54502900 | -0.79335600 |
| P | 0.24496700 | 1.54455100 | 0.47139900 |
| C | 2.18753400 | -2.08020400 | 1.66716200 |
| C | 0.99120600 | -0.24724600 | 2.75122800 |
| C | 0.86426900 | 2.87648300 | 1.57701100 |
| C | 0.81533700 | 2.07545500 | -1.18879400 |
| C | -1.58773200 | 1.70879200 | 0.56624000 |
| C | 0.39811600 | 4.29083200 | 1.25500400 |
| C | 1.06550700 | 5.33764300 | 2.13733800 |
| C | 0.59250600 | 6.74817400 | 1.83718500 |
| C | 2.32233900 | 2.31774700 | -1.20494000 |
| C | -2.25121100 | 2.57972000 | -0.49479800 |
| C | 1.62203200 | -3.34541100 | 1.49503800 |
| N | 2.27524000 | -4.49012800 | 1.74560400 |
| C | 3.52824300 | -4.39863700 | 2.20127300 |
| C | 3.48125500 | -2.00948000 | 2.18197900 |
| C | 4.17396100 | -3.18981000 | 2.44113500 |
| C | 2.51526900 | -1.22603100 | -1.45919900 |
| C | 2.42295100 | -1.83319600 | -2.72624700 |
| C | 3.56206500 | -2.04170600 | -3.49440800 |
| C | 4.82049100 | -1.66349000 | -3.02595600 |
| C | 3.78788900 | -0.85549900 | -0.99164300 |
| C | 4.92166900 | -1.07534400 | -1.76658900 |
| C | -4.08755300 | -0.55172600 | -1.35654600 |
| C | -5.46072400 | -0.54593200 | -1.17590600 |
| C | -6.06513100 | -1.55592700 | -0.41518700 |
| C | -3.90090300 | -2.54252000 | -0.04137600 |
| C | -5.27870200 | -2.56305800 | 0.15370400 |
| O | -7.41548300 | -1.47120800 | -0.28603600 |
| O | -0.96728300 | -2.51093800 | -0.39527500 |
| O | -1.14947100 | -0.50211200 | -1.69550600 |
| C | -8.05462400 | -2.47983400 | 0.47503700 |
| C | -3.72737700 | 2.80177300 | -0.19159700 |
| C | -4.45643000 | 3.52412000 | -1.30958500 |
| C | 2.91855600 | 2.25799900 | -2.60379400 |
| C | 4.40420100 | 2.56584000 | -2.61265700 |
| H | 1.96057500 | 2.81841100 | 1.55591300 |
| H | 0.57356000 | 2.60590400 | 2.60018400 |
| H | 0.60096500 | 4.52560600 | 0.19998000 |
| H | -0.69300700 | 4.35639000 | 1.37162500 |
| H | 2.15422100 | 5.27062200 | 2.00707100 |
| H | 0.87632600 | 5.09389400 | 3.19131300 |
| H | 0.80129900 | 7.02411500 | 0.79815100 |
| H | -0.48776000 | 6.84519500 | 1.98850000 |
| H | 0.26578100 | 2.96969800 | -1.50616700 |
| H | 0.55220400 | 1.28355300 | -1.89509800 |
| H | 2.54665400 | 3.29041500 | -0.74266000 |
| H | 2.82671800 | 1.56455100 | -0.58075100 |
| H | -1.79878200 | 2.09437900 | 1.57327600 |
| H | -2.02300200 | 0.70425600 | 0.52405300 |
| H | -1.74665100 | 3.55260700 | -0.57934400 |
| H | -2.13660400 | 2.09087100 | -1.47178500 |
| H | 1.08240700 | 7.48555800 | 2.47859000 |
| H | 0.59965100 | -3.45739500 | 1.13665400 |
| H | 4.03692100 | -5.34282800 | 2.39345900 |
| H | 3.95887800 | -1.04931500 | 2.36966200 |
| H | 5.19018300 | -3.17086700 | 2.82516700 |
| H | 1.44172300 | -2.12899200 | -3.09245000 |
| H | 3.46342000 | -2.50739400 | -4.47198400 |
| H | 5.70703300 | -1.82976200 | -3.63036200 |
| H | 3.86582200 | -0.38458100 | -0.01337700 |
| H | 5.89360300 | -0.77341000 | -1.38251000 |
| H | -3.62706900 | 0.23588000 | -1.95142900 |
| H | -6.09103100 | 0.22601800 | -1.60973100 |
| H | -3.29638300 | -3.32856500 | 0.40613700 |
| H | -5.73023500 | -3.35450000 | 0.74247600 |
| H | -0.17811800 | -0.59160800 | -1.64095700 |
| H | -0.02452800 | -2.34893000 | -0.56845000 |
| H | -9.11675400 | -2.23762800 | 0.46890300 |
| H | -7.90671600 | -3.47323800 | 0.03415000 |
| H | -7.69213300 | -2.49556000 | 1.51025400 |
| H | -3.81815900 | 3.36671500 | 0.74655700 |
| H | -4.20288800 | 1.82974200 | -0.00428100 |
| H | -5.51045300 | 3.68669900 | -1.06760800 |
| H | -4.00898400 | 4.50306500 | -1.51319600 |
| H | -4.41754300 | 2.94923300 | -2.24160300 |
| H | 2.38103800 | 2.95402300 | -3.26227900 |
| H | 2.74556800 | 1.25314400 | -3.01375400 |
| H | 4.83203000 | 2.47549600 | -3.61491300 |
| H | 4.60500300 | 3.58194000 | -2.25525300 |
| H | 4.94570800 | 1.87175500 | -1.96046900 |
| O | 0.83052000 | -0.08862100 | 3.88412800 |

**Ni[P(n-Bu)$_3$](Pyr)(CO)(OPh)[MeOPh-B(OH)$_2$], Iso-2**

| | | | |
|---|---|---|---|
| Ni | 1.37590400 | -0.46404400 | -0.48582800 |
| O | 2.84434600 | -1.68624700 | 0.00873900 |
| B | 0.21123100 | -2.89505200 | 0.85980300 |
| C | -1.33753700 | -2.87252000 | 0.71755300 |
| C | 0.83241800 | -1.54824800 | -1.86339800 |
| C | 2.24666500 | 0.86211400 | 0.59492300 |
| P | -0.36834400 | 0.89401000 | -0.45180000 |
| C | -0.89625500 | 1.08758100 | 1.29718400 |
| C | -0.09739300 | 2.56611700 | -1.15980100 |
| C | -1.80500000 | 0.17350100 | -1.35037400 |
| C | -0.98368600 | 3.69539900 | -0.64141200 |
| C | -0.65062300 | 5.02742200 | -1.30087200 |
| C | -2.96374900 | 1.08270900 | -1.74786200 |
| C | -4.08361600 | 0.29270500 | -2.41476200 |
| C | -2.36498300 | 1.37408500 | 1.58440100 |
| C | -1.50531000 | 6.16795000 | -0.77870600 |
| C | -5.28017200 | 1.15609900 | -2.77013400 |
| C | 2.30854400 | 0.84731400 | 1.99046400 |
| N | 3.13909600 | 1.61294100 | 2.71621800 |
| C | 3.95833200 | 2.43280400 | 2.04942900 |
| C | 3.10538200 | 1.73891200 | -0.07182600 |
| C | 3.98365700 | 2.53382000 | 0.66213500 |
| C | 4.10470900 | -1.35381000 | -0.27323400 |
| C | 5.10610900 | -1.48525100 | 0.70602700 |
| C | 6.42426400 | -1.14979400 | 0.41923000 |
| C | 6.78080200 | -0.66436700 | -0.83914500 |
| C | 4.47471100 | -0.86265200 | -1.53998500 |
| C | 5.79426500 | -0.52273900 | -1.81481600 |
| C | -1.96890600 | -3.41034400 | -0.41994400 |

| | | | |
|---|---|---|---|
| C | -3.32040400 | -3.22524400 | -0.66153600 |
| C | -4.09503200 | -2.48882400 | 0.24513900 |
| C | -2.14657200 | -2.17601700 | 1.62388600 |
| C | -3.50898300 | -1.98087400 | 1.40941700 |
| O | -5.39818400 | -2.30404500 | -0.09954200 |
| O | 0.77888800 | -2.06426600 | 1.80309100 |
| O | 0.94731800 | -3.65464200 | -0.01050300 |
| C | -6.14640700 | -1.39245100 | 0.68498500 |
| C | -2.67029600 | 1.32157000 | 3.07548500 |
| C | -4.13730600 | 1.56683100 | 3.37849200 |
| H | -0.19648300 | 2.46226600 | -2.24826900 |
| H | 0.95278600 | 2.81674100 | -0.97316800 |
| H | -2.04399700 | 3.46429800 | -0.80581400 |
| H | -0.86078000 | 3.78951700 | 0.44626000 |
| H | -2.16398100 | -0.65331200 | -0.72133500 |
| H | -1.40209500 | -0.30307700 | -2.25256000 |
| H | -3.36852200 | 1.60611000 | -0.87284500 |
| H | -2.60835100 | 1.86826900 | -2.42992200 |
| H | -0.24666600 | 1.85400600 | 1.73739200 |
| H | -0.60177100 | 0.14512800 | 1.77923200 |
| H | -2.65596700 | 2.35391200 | 1.18261200 |
| H | -2.99329200 | 0.63224300 | 1.07272200 |
| H | -0.77546100 | 4.93124600 | -2.38782800 |
| H | 0.41272900 | 5.25069200 | -1.14165000 |
| H | -2.56974600 | 5.97858900 | -0.95358400 |
| H | -1.37142000 | 6.30220200 | 0.29981700 |
| H | -4.39526600 | -0.52018300 | -1.74492300 |
| H | -3.69178600 | -0.20115100 | -3.31426400 |
| H | -6.07389400 | 0.57231000 | -3.24398400 |
| H | -5.70582700 | 1.62763100 | -1.87715800 |
| H | -5.00389700 | 1.95985100 | -3.46117200 |
| H | -1.25542100 | 7.11608900 | -1.26212600 |
| H | 1.66170500 | 0.18291900 | 2.56465000 |
| H | 4.62817800 | 3.03913200 | 2.65871400 |
| H | 3.11603500 | 1.78821900 | -1.16049100 |
| H | 4.67262500 | 3.21425500 | 0.16880400 |
| H | 4.82052000 | -1.84567200 | 1.69151300 |
| H | 7.18123400 | -1.25871600 | 1.19243300 |
| H | 7.81096900 | -0.39695900 | -1.05499100 |
| H | 3.69700700 | -0.74096100 | -2.29385700 |
| H | 6.05460400 | -0.14171200 | -2.79998100 |
| H | -1.36876300 | -3.95772700 | -1.14387000 |
| H | -3.79942800 | -3.61447200 | -1.55556700 |
| H | -1.69221400 | -1.76068400 | 2.52243700 |
| H | -4.09645300 | -1.42719800 | 2.13574000 |
| H | 1.87082100 | -3.34629800 | -0.02746100 |
| H | 1.73865900 | -1.99081500 | 1.66209500 |
| H | -7.11999100 | -1.30448000 | 0.20370200 |
| H | -6.28148000 | -1.75104600 | 1.71265800 |
| H | -5.66482200 | -0.40444000 | 0.71410300 |
| H | -2.04744100 | 2.05507900 | 3.60412100 |
| H | -2.36566600 | 0.33994300 | 3.46563100 |
| H | -4.34697000 | 1.51306200 | 4.44997900 |
| H | -4.45782300 | 2.55355800 | 3.02754300 |
| H | -4.77418500 | 0.82528800 | 2.88098100 |
| O | 0.62841300 | -2.11813800 | -2.84232300 |

**TS_(B-C act.), transmetalation, Iso1**

| | | | |
|---|---|---|---|
| Ni | 0.14438300 | 0.05056700 | -0.78101900 |
| O | 0.96100300 | 2.32049500 | -2.36538300 |
| B | 2.16401500 | 1.53431400 | -2.49165200 |
| C | 2.20195600 | 0.08988600 | -1.03868800 |
| C | 0.50901500 | 0.02752100 | 1.11286400 |
| P | -1.94501700 | -0.41437500 | -0.08806300 |
| C | -1.93546500 | -2.16659400 | 0.45582700 |
| C | -2.61478000 | 0.62018100 | 1.27972600 |
| C | -3.27806900 | -0.34369300 | -1.35722300 |
| C | -2.54132700 | 0.02249500 | 2.68109500 |
| C | -2.76520600 | 1.07744700 | 3.75506700 |
| C | -4.62260100 | -0.93025100 | -0.93970600 |
| C | -5.74707400 | -0.54435500 | -1.89230000 |
| C | -1.15404700 | -3.03291100 | -0.52702100 |
| C | -2.59373500 | 0.52645100 | 5.15828600 |
| C | -7.06955300 | -1.19558000 | -1.52999900 |
| C | 0.91898800 | -1.15091300 | 1.75167200 |
| N | 1.26486600 | -1.24261500 | 3.04418000 |
| C | 1.22577100 | -0.11476400 | 3.76573400 |
| C | 0.48091800 | 1.18375600 | 1.89607400 |
| C | 0.84763500 | 1.11697500 | 3.23900300 |
| C | 0.58162100 | 3.03300700 | -1.28846000 |
| C | 1.46138500 | 3.72822500 | -0.44367300 |
| C | 0.95504900 | 4.49080900 | 0.60643200 |
| C | -0.41653000 | 4.57878800 | 0.84338500 |
| C | -0.79982300 | 3.11573000 | -1.04239800 |
| C | -1.29178000 | 3.88487400 | 0.00781300 |
| C | 2.40180200 | -1.24006000 | -1.49193800 |
| C | 3.32011900 | -2.10879900 | -0.91994400 |
| C | 4.12101500 | -1.66383100 | 0.14035700 |
| C | 3.04264900 | 0.48052200 | 0.03182800 |
| C | 4.00523800 | -0.34011900 | 0.59529800 |
| O | 4.98141700 | -2.57398100 | 0.65434000 |
| O | 3.34526600 | 2.25602600 | -2.20891600 |
| O | 2.04375700 | 0.76723100 | -3.67018600 |
| H | -3.64832800 | 0.89096200 | 1.03086000 |
| H | -2.03885600 | 1.55361200 | 1.24696800 |
| H | -3.27866400 | -0.78635600 | 2.78242100 |
| H | -1.55969500 | -0.44023200 | 2.84679900 |
| H | -2.91690900 | -0.84335900 | -2.26249200 |
| H | -3.38426900 | 0.71680900 | -1.62563100 |
| H | -4.54604100 | -2.02505200 | -0.89697800 |
| H | -4.88741900 | -0.61199700 | 0.07930900 |
| H | -2.96299900 | -2.52639700 | 0.58925600 |
| H | -1.45845300 | -2.19796400 | 1.44144200 |
| H | -1.66137400 | -3.04913100 | -1.50359300 |
| H | -0.17604500 | -2.55774200 | -0.71541000 |
| H | -3.76323800 | 1.52086500 | 3.63668300 |
| H | -2.05041900 | 1.89648800 | 3.58665600 |
| H | -3.30659800 | -0.27990800 | 5.36175600 |
| H | -1.58710600 | 0.11397700 | 5.29146500 |
| H | -5.46176700 | -0.81669500 | -2.91685800 |
| H | -5.85448900 | 0.54825600 | -1.89458700 |
| H | -7.87195700 | -0.88607800 | -2.20462200 |
| H | -7.00142200 | -2.28737300 | -1.57650700 |
| H | -7.37436600 | -0.93163100 | -0.51160100 |
| H | -2.74114500 | 1.29672500 | 5.92013100 |
| H | 0.99346200 | -2.08271900 | 1.18351600 |
| H | 1.51117100 | -0.20530900 | 4.81388900 |
| H | 0.18372000 | 2.13740400 | 1.46447200 |
| H | 0.83583200 | 2.00618900 | 3.86595300 |
| H | 2.52676800 | 3.67316800 | -0.63540400 |
| H | 1.64908200 | 5.02927300 | 1.24764100 |
| H | -0.79804800 | 5.18208200 | 1.66190500 |
| H | -1.47146800 | 2.58832200 | -1.71742200 |
| H | -2.36602200 | 3.94206100 | 0.17015500 |

| | | | |
|---|---|---|---|
| H | 1.82090200 | -1.59926600 | -2.34294800 |
| H | 3.44894400 | -3.12764400 | -1.27616000 |
| H | 2.92835500 | 1.48709500 | 0.43373100 |
| H | 4.62300300 | 0.02423400 | 1.41018800 |
| H | 4.11989900 | 1.69131700 | -2.23231200 |
| C | 5.74165400 | -2.18290300 | 1.78679700 |
| H | 6.33086400 | -3.05420400 | 2.06991000 |
| H | 6.41593100 | -1.35076700 | 1.55261800 |
| H | 5.09170600 | -1.89337900 | 2.62107300 |
| C | -0.93130500 | -4.45604800 | -0.03846000 |
| C | -0.05982600 | -5.25606900 | -0.98942400 |
| H | -1.89942800 | -4.95473500 | 0.10344500 |
| H | -0.46334000 | -4.42016000 | 0.95473400 |
| H | 0.12666500 | -6.26835500 | -0.62125000 |
| H | -0.52339200 | -5.34243900 | -1.97806300 |
| H | 0.91193800 | -4.76765400 | -1.12686800 |
| C | -0.33943700 | -0.03435100 | -2.54568900 |
| O | -0.80747900 | -0.12018600 | -3.59442800 |
| H | 2.87613700 | 0.34496500 | -3.88722100 |

**TS_(B-C act.), transmetalation, Iso2**

| | | | |
|---|---|---|---|
| Ni | 0.19001000 | -0.43933200 | -0.60971700 |
| O | 0.94788800 | 1.02651200 | -2.77361800 |
| B | 2.10106500 | 0.14795700 | -2.69645100 |
| C | 2.24118700 | -0.58834600 | -0.84056600 |
| C | 0.51938500 | 0.41179600 | 1.09262000 |
| P | -1.96703100 | -0.25885800 | 0.01905600 |
| C | -2.32839700 | -1.54134300 | 1.27743500 |
| C | -2.50848400 | 1.34680900 | 0.73853200 |
| C | -3.21567500 | -0.55245500 | -1.30601900 |
| C | -2.53884800 | 1.43329200 | 2.26046000 |
| C | -2.65885100 | 2.87446000 | 2.73674400 |
| C | -4.64855400 | -0.77181500 | -0.83357700 |
| C | -5.64234800 | -0.79417800 | -1.98840000 |
| C | -1.90989600 | -2.93537600 | 0.82630800 |
| C | -2.60083300 | 2.99550900 | 4.24792900 |
| C | -7.06663800 | -1.05088800 | -1.53090100 |
| C | 0.71838500 | -0.41389400 | 2.20896900 |
| N | 0.96628800 | 0.02086100 | 3.45365800 |
| C | 1.04465400 | 1.34579100 | 3.63060600 |
| C | 0.60697000 | 1.78925600 | 1.31737500 |
| C | 0.87908000 | 2.26475200 | 2.59858900 |
| C | 0.79022600 | 2.23049700 | -2.18739600 |
| C | 1.84135900 | 3.06405700 | -1.77379100 |
| C | 1.55577500 | 4.29051700 | -1.17826000 |
| C | 0.24204900 | 4.71662700 | -0.98758900 |
| C | -0.53222600 | 2.66564200 | -2.00497100 |
| C | -0.80183100 | 3.89521500 | -1.41345500 |
| C | 2.58031100 | -1.96727200 | -0.83587400 |
| C | 3.65161100 | -2.48394300 | -0.12539900 |
| C | 4.46398900 | -1.62277200 | 0.62289400 |
| C | 3.08747700 | 0.22826400 | -0.04706800 |
| C | 4.18727900 | -0.24577700 | 0.64497900 |
| O | 5.49734100 | -2.20350000 | 1.27442100 |
| O | 3.34152100 | 0.79730100 | -2.86648800 |
| O | 1.87546800 | -1.03105300 | -3.45976400 |
| H | -3.49449700 | 1.58974600 | 0.32148600 |
| H | -1.81607400 | 2.09847300 | 0.34146300 |
| H | -3.37209500 | 0.83563500 | 2.65587700 |
| H | -1.62477000 | 0.99887600 | 2.68602000 |
| H | -2.88860500 | -1.40450200 | -1.91105900 |
| H | -3.15983600 | 0.32107500 | -1.96999100 |
| H | -4.71380500 | -1.72096900 | -0.28373900 |
| H | -4.94425300 | 0.00812000 | -0.11646000 |
| H | -3.39480500 | -1.50937100 | 1.53452800 |
| H | -1.78131500 | -1.25741600 | 2.18376400 |
| H | -2.44690100 | -3.21278500 | -0.09334500 |
| H | -0.84304700 | -2.92106000 | 0.55252900 |
| H | -3.59026700 | 3.31316100 | 2.35315600 |
| H | -1.84369100 | 3.45775800 | 2.28287600 |
| H | -3.40930100 | 2.43150200 | 4.72536100 |
| H | -1.65475000 | 2.59814000 | 4.63293800 |
| H | -5.33576400 | -1.56222200 | -2.71076000 |
| H | -5.58693300 | 0.16062200 | -2.52799500 |
| H | -7.76686400 | -1.05851000 | -2.37045200 |
| H | -7.15206600 | -2.01647400 | -1.02131100 |
| H | -7.40251900 | -0.28123700 | -0.82782600 |
| H | -2.68364700 | 4.03467400 | 4.57790000 |
| H | 0.68199300 | -1.50153900 | 2.09762900 |
| H | 1.24874300 | 1.68335000 | 4.64696500 |
| H | 0.46816200 | 2.49774900 | 0.50363800 |
| H | 0.95204200 | 3.33320700 | 2.79204400 |
| H | 2.86380200 | 2.73890900 | -1.92491100 |
| H | 2.38047600 | 4.92292800 | -0.85842700 |
| H | 0.03462800 | 5.67498700 | -0.52091100 |
| H | -1.33646300 | 2.01362800 | -2.34129500 |
| H | -1.83517100 | 4.20678100 | -1.27585400 |
| H | 2.00807400 | -2.65125800 | -1.45983600 |
| H | 3.89929700 | -3.54137800 | -0.14830500 |
| H | 2.88269600 | 1.29663200 | -0.00168500 |
| H | 4.81180000 | 0.43880000 | 1.20970800 |
| H | 4.01161900 | 0.13146600 | -3.03882500 |
| C | 6.35283300 | -1.36077200 | 2.03071800 |
| H | 7.11004400 | -2.01272000 | 2.46418100 |
| H | 6.83845300 | -0.61016900 | 1.39647100 |
| H | 5.80795100 | -0.85158000 | 2.83375200 |
| C | -2.14402900 | -3.99660400 | 1.89153300 |
| C | -1.72173700 | -5.38211700 | 1.43784600 |
| H | -3.20592500 | -3.99953800 | 2.17215100 |
| H | -1.59554800 | -3.71449800 | 2.80045500 |
| H | -1.89092500 | -6.13335500 | 2.21367400 |
| H | -2.27815100 | -5.69444100 | 0.54777200 |
| H | -0.65751500 | -5.40648700 | 1.18094900 |
| C | -0.24128900 | -1.61374700 | -1.93735900 |
| O | -0.63603800 | -2.46022000 | -2.61673500 |
| H | 1.19451100 | -0.84578200 | -4.11140700 |

**Ni[P(n-Bu)$_3$](Pyr)(CO)(PhOMe)[OPh-B(OH)$_2$], Iso1**

| | | | |
|---|---|---|---|
| Ni | 0.65366500 | 1.66307100 | 0.29195800 |
| O | 0.54968100 | -2.79526600 | 1.58074400 |
| B | 1.61974100 | -1.92477300 | 1.56129800 |
| C | 2.61616500 | 1.73474200 | 0.18436200 |
| C | 0.69503700 | 0.76901400 | -1.41264600 |
| P | -1.61062300 | 1.32867300 | 0.23019100 |
| C | -2.33310800 | 1.57390500 | -1.44422200 |
| C | -1.86993300 | -0.42854600 | 0.71481000 |
| C | -2.67345500 | 2.32353600 | 1.36339200 |
| C | -2.96007300 | -1.23824500 | 0.02120300 |
| C | -2.77895700 | -2.73014300 | 0.27241400 |
| C | -3.99715700 | 1.70783100 | 1.80689800 |
| C | -4.82887100 | 2.67357100 | 2.64123100 |
| C | -3.84231500 | 1.71727500 | -1.59452000 |
| C | -3.88079400 | -3.58152600 | -0.32885900 |

| | | | |
|---|---|---|---|
| C | -6.12964600 | 2.05980600 | 3.12625100 |
| C | 0.71681500 | 1.50055500 | -2.61024500 |
| N | 0.64443900 | 0.97335400 | -3.84078000 |
| C | 0.55220900 | -0.35944100 | -3.93136500 |
| C | 0.63472200 | -0.62143300 | -1.55709400 |
| C | 0.55387900 | -1.19952600 | -2.82263100 |
| C | 0.50133500 | -4.01097600 | 0.95469200 |
| C | 1.05507000 | -4.22121000 | -0.30989500 |
| C | 0.87295300 | -5.44861100 | -0.94089200 |
| C | 0.14663100 | -6.46481100 | -0.32421400 |
| C | -0.22475200 | -5.02425200 | 1.58059900 |
| C | -0.39944500 | -6.24615100 | 0.93924600 |
| C | 3.29373600 | 2.93670400 | 0.46422700 |
| C | 4.67834700 | 3.03620400 | 0.41585200 |
| C | 5.45472200 | 1.91541700 | 0.10405900 |
| C | 3.42207700 | 0.63852900 | -0.14822600 |
| C | 4.81953900 | 0.70490400 | -0.17695500 |
| O | 6.80474100 | 2.10479500 | 0.09547100 |
| O | 2.84978000 | -2.36170900 | 1.16147100 |
| O | 1.30418200 | -0.65990300 | 1.97689200 |
| H | -2.00679600 | -0.43550700 | 1.80355500 |
| H | -0.90623000 | -0.92215700 | 0.56245200 |
| H | -3.95531600 | -0.91352700 | 0.35732300 |
| H | -2.93454800 | -1.06025200 | -1.06357000 |
| H | -2.84435200 | 3.28569600 | 0.86183100 |
| H | -2.07127000 | 2.55827300 | 2.24918900 |
| H | -4.58446600 | 1.38049200 | 0.94036300 |
| H | -3.80127700 | 0.79907800 | 2.39224900 |
| H | -1.96251300 | 0.74614400 | -2.06132500 |
| H | -1.83570400 | 2.46535300 | -1.84915500 |
| H | -4.35669300 | 0.83503600 | -1.19053200 |
| H | -4.20323900 | 2.57315400 | -1.00648200 |
| H | -2.70702500 | -2.91187700 | 1.35380000 |
| H | -1.80603500 | -3.03638900 | -0.13712800 |
| H | -4.85822200 | -3.33557400 | 0.10024300 |
| H | -3.95327500 | -3.43141800 | -1.41151000 |
| H | -5.03745100 | 3.57264000 | 2.04508000 |
| H | -4.23261600 | 3.01664800 | 3.49735000 |
| H | -6.71539700 | 2.76589400 | 3.72091200 |
| H | -6.75408700 | 1.73838800 | 2.28587200 |
| H | -5.94407400 | 1.17835600 | 3.74912200 |
| H | -3.70187800 | -4.64702200 | -0.15632000 |
| H | 0.78936800 | 2.58978000 | -2.57908100 |
| H | 0.48331300 | -0.77043200 | -4.93836200 |
| H | 0.63861600 | -1.27186800 | -0.68145100 |
| H | 0.48905600 | -2.27908500 | -2.94236400 |
| H | 1.62266300 | -3.43045500 | -0.79043300 |
| H | 1.30209900 | -5.60590600 | -1.92682200 |
| H | 0.00657900 | -7.41855600 | -0.82396400 |
| H | -0.65614200 | -4.83101600 | 2.55822000 |
| H | -0.96839300 | -7.03019900 | 1.43121500 |
| H | 2.73247400 | 3.83234100 | 0.72617200 |
| H | 5.18365000 | 3.97471900 | 0.62853300 |
| H | 2.96024200 | -0.30457100 | -0.43904200 |
| H | 5.39154500 | -0.17736100 | -0.44910700 |
| H | 2.00673900 | -0.01279100 | 1.82743000 |
| H | 3.52406100 | -1.67718100 | 1.20033100 |
| C | 7.60392500 | 0.98305600 | -0.22325300 |
| H | 8.63865100 | 1.32175200 | -0.17818200 |
| H | 7.45997800 | 0.16489500 | 0.49428100 |
| H | 7.39102400 | 0.60634600 | -1.23181200 |
| C | -4.24543400 | 1.90630600 | -3.05192500 |
| C | -5.74367100 | 2.07230100 | -3.22951700 |
| H | -3.88994300 | 1.04612500 | -3.63503700 |
| H | -3.71853700 | 2.77878600 | -3.46085500 |
| H | -6.01606500 | 2.20570800 | -4.27994500 |
| H | -6.28694100 | 1.19747000 | -2.85631100 |
| H | -6.11400000 | 2.94399900 | -2.67928100 |
| C | 0.64778900 | 2.53162800 | 1.85007200 |
| O | 0.65478300 | 3.08432100 | 2.86501200 |

**Ni[P(n-Bu)$_3$](Pyr)(CO)(PhOMe)[OPh-B(OH)$_2$], Iso2**

| | | | |
|---|---|---|---|
| Ni | 0.64044100 | -1.61990600 | -0.35721900 |
| O | 0.38083100 | 2.85149000 | -1.71852700 |
| B | 1.41869800 | 2.02307700 | -2.09292600 |
| C | 2.57186300 | -1.75792200 | -0.16283100 |
| C | 0.74442900 | -0.71547600 | 1.33607400 |
| P | -1.62086800 | -1.29075600 | -0.26109200 |
| C | -2.30605300 | -1.66042500 | 1.40750200 |
| C | -1.93363200 | 0.49517900 | -0.60694500 |
| C | -2.69986900 | -2.21992300 | -1.43433200 |
| C | -2.97734400 | 1.24942600 | 0.21080200 |
| C | -2.79291700 | 2.75583600 | 0.07984800 |
| C | -4.04416400 | -1.60019200 | -1.80361000 |
| C | -4.90925700 | -2.54992600 | -2.62214200 |
| C | -3.81094100 | -1.81771700 | 1.58382700 |
| C | -3.85723600 | 3.55872300 | 0.80301700 |
| C | -6.23174200 | -1.93191400 | -3.03769900 |
| C | 0.73673000 | -1.40282600 | 2.55955900 |
| N | 0.70820500 | -0.82914700 | 3.77037100 |
| C | 0.69924400 | 0.50940300 | 3.81164700 |
| C | 0.77563000 | 0.68062000 | 1.42876600 |
| C | 0.74179100 | 1.30760600 | 2.67275700 |
| C | 0.48182100 | 3.97342900 | -0.93337400 |
| C | 1.33993900 | 4.04656100 | 0.16453400 |
| C | 1.30854400 | 5.17524500 | 0.97975900 |
| C | 0.43579700 | 6.22608900 | 0.70852200 |
| C | -0.38935200 | 5.02438900 | -1.21819800 |
| C | -0.41035400 | 6.14601600 | -0.39588100 |
| C | 3.16254000 | -3.01744600 | 0.01183100 |
| C | 4.53855800 | -3.16169700 | 0.15457400 |
| C | 5.37035400 | -2.03907800 | 0.11052700 |
| C | 3.41997300 | -0.65145700 | -0.19153200 |
| C | 4.80676400 | -0.77410200 | -0.06808200 |
| O | 6.70802500 | -2.28034600 | 0.25535600 |
| O | 2.71634100 | 2.35558100 | -1.85487600 |
| O | 1.10342500 | 0.85457700 | -2.72949800 |
| H | -2.15827800 | 0.57777200 | -1.67865600 |
| H | -0.96786700 | 0.99275500 | -0.47117700 |
| H | -3.99088200 | 0.96030100 | -0.10115400 |
| H | -2.89688000 | 0.97687900 | 1.27250500 |
| H | -2.84558500 | -3.21414200 | -0.99048200 |
| H | -2.11339500 | -2.39212000 | -2.34519000 |
| H | -4.59229600 | -1.29737800 | -0.90301400 |
| H | -3.88003600 | -0.67560400 | -2.37410400 |
| H | -1.92051700 | -0.88048000 | 2.07574600 |
| H | -1.79840200 | -2.57776100 | 1.73447500 |
| H | -4.33575700 | -0.91662800 | 1.23992300 |
| H | -4.18303300 | -2.64107500 | 0.95741800 |
| H | -2.76930300 | 3.03036500 | -0.98409400 |
| H | -1.79824800 | 3.01875500 | 0.46808700 |
| H | -4.85574400 | 3.35384000 | 0.40202300 |
| H | -3.87896300 | 3.31784700 | 1.87120200 |

| | | | |
|---|---|---|---|
| H | -5.08915000 | -3.46148300 | -2.03561800 |
| H | -4.34950200 | -2.87411200 | -3.50970100 |
| H | -6.84175700 | -2.62919600 | -3.61812800 |
| H | -6.81878900 | -1.62762300 | -2.16468500 |
| H | -6.07653400 | -1.03893800 | -3.65243200 |
| H | -3.67917400 | 4.63435300 | 0.71219800 |
| H | 0.74654700 | -2.49547500 | 2.56729600 |
| H | 0.66623700 | 0.96042800 | 4.80334200 |
| H | 0.81873900 | 1.29608400 | 0.52804200 |
| H | 0.74807400 | 2.39290700 | 2.75284400 |
| H | 2.02227500 | 3.22947500 | 0.37605200 |
| H | 1.97408000 | 5.22583300 | 1.83734600 |
| H | 0.41549700 | 7.10048700 | 1.35161900 |
| H | -1.05160100 | 4.93832600 | -2.07460500 |
| H | -1.09558900 | 6.95902500 | -0.61982900 |
| H | 2.54485500 | -3.91427400 | 0.03826100 |
| H | 4.99222400 | -4.13931600 | 0.29438300 |
| H | 3.00244300 | 0.34991400 | -0.28433900 |
| H | 5.42709900 | 0.11673900 | -0.09244700 |
| H | 3.30952200 | 1.67080400 | -2.17867200 |
| C | 7.56374700 | -1.15779400 | 0.22209100 |
| H | 8.57613400 | -1.54011500 | 0.35349100 |
| H | 7.50157600 | -0.62535800 | -0.73627500 |
| H | 7.33846500 | -0.44905300 | 1.02972000 |
| C | -4.18118900 | -2.08573900 | 3.03762400 |
| C | -5.67448600 | -2.26665900 | 3.24034100 |
| H | -3.81519500 | -1.25563700 | 3.65681000 |
| H | -3.64265400 | -2.97607500 | 3.38851300 |
| H | -5.92246400 | -2.45091900 | 4.28903300 |
| H | -6.22878200 | -1.37739900 | 2.92160400 |
| H | -6.05460400 | -3.11287300 | 2.65799000 |
| C | 0.75478300 | -2.39590500 | -1.95749600 |
| O | 0.88381900 | -2.84873900 | -3.01355100 |
| H | 0.15360500 | 0.75306500 | -2.83490800 |

**Ni[P(n-Bu)$_3$](Pyr)(CO)(PhOMe), CO-N trans**

| | | | |
|---|---|---|---|
| Ni | 0.92900900 | 0.68660700 | -0.32255900 |
| C | 2.86586000 | 0.57662000 | -0.25761300 |
| C | 1.04525700 | -1.22989500 | -0.19454400 |
| P | -1.34180900 | 0.48760700 | -0.15060300 |
| C | -2.02663100 | -0.90022900 | -1.14815600 |
| C | -1.72364900 | 0.11033400 | 1.61256600 |
| C | -2.35474700 | 1.97429100 | -0.56774300 |
| C | -2.96892900 | -0.70875400 | 1.93299200 |
| C | -3.11225500 | -0.96670000 | 3.42734400 |
| C | -3.72639200 | 2.12996800 | 0.07975300 |
| C | -4.42901700 | 3.40966100 | -0.35663800 |
| C | -3.51332200 | -0.92469800 | -1.47903000 |
| C | -4.33562400 | -1.79941700 | 3.76515300 |
| C | -5.78299300 | 3.58782600 | 0.30609500 |
| C | 1.02595500 | -2.02038300 | -1.35424700 |
| N | 1.00090200 | -3.36039700 | -1.38131100 |
| C | 1.00912100 | -3.99163200 | -0.20096900 |
| C | 1.09518000 | -1.93218200 | 1.01501300 |
| C | 1.06521200 | -3.32539300 | 1.01911600 |
| C | 3.65753300 | 0.38816600 | -1.39701400 |
| C | 5.04361900 | 0.32925700 | -1.31188500 |
| C | 5.67738300 | 0.45698600 | -0.07172600 |
| C | 3.51529500 | 0.70620500 | 0.96953400 |
| C | 4.90820300 | 0.64750800 | 1.07711800 |
| O | 7.04289500 | 0.38085400 | -0.08824000 |
| H | -1.74854200 | 1.07492200 | 2.13683300 |
| H | -0.84336900 | -0.41533800 | 2.00195400 |
| H | -3.87025400 | -0.20266300 | 1.56308500 |
| H | -2.92309800 | -1.67108400 | 1.40337400 |
| H | -2.44036500 | 1.99396000 | -1.66274200 |
| H | -1.73757500 | 2.84444200 | -0.31099800 |
| H | -4.36661900 | 1.27055700 | -0.15424600 |
| H | -3.61904600 | 2.13430900 | 1.17366600 |
| H | -1.72834700 | -1.81955900 | -0.62710900 |
| H | -1.44447000 | -0.91159000 | -2.07893000 |
| H | -4.11464700 | -0.92900500 | -0.56089600 |
| H | -3.79420000 | -0.00880000 | -2.01819100 |
| H | -3.15519900 | -0.00422200 | 3.95518300 |
| H | -2.20482900 | -1.46623500 | 3.79303900 |
| H | -5.25552100 | -1.30540500 | 3.43434300 |
| H | -4.29691400 | -2.77728800 | 3.27356600 |
| H | -4.54397200 | 3.40180700 | -1.44901600 |
| H | -3.78429000 | 4.26989000 | -0.13194400 |
| H | -6.27605600 | 4.50839000 | -0.01742100 |
| H | -6.45272100 | 2.75410400 | 0.06981900 |
| H | -5.68799400 | 3.62961200 | 1.39635600 |
| H | -4.42413800 | -1.97437500 | 4.84073000 |
| H | 1.02041400 | -1.54221700 | -2.33722600 |
| H | 0.98101300 | -5.08064900 | -0.23715100 |
| H | 1.16513000 | -1.39764800 | 1.96310800 |
| H | 1.09398100 | -3.88574600 | 1.95050000 |
| H | 3.19312100 | 0.27312200 | -2.37525900 |
| H | 5.65744300 | 0.17801300 | -2.19610400 |
| H | 2.93697400 | 0.84990400 | 1.88211600 |
| H | 5.37467500 | 0.75116200 | 2.05179600 |
| C | 7.69899900 | 0.48570000 | 1.15718400 |
| H | 8.76522200 | 0.38808400 | 0.95201200 |
| H | 7.51238500 | 1.45576100 | 1.63693100 |
| H | 7.39047500 | -0.30963600 | 1.84848600 |
| C | -3.88416600 | -2.13703300 | -2.32420600 |
| C | -5.35812500 | -2.17289700 | -2.68466100 |
| H | -3.60715100 | -3.04993000 | -1.78006900 |
| H | -3.27333700 | -2.14017900 | -3.23667100 |
| H | -5.60774400 | -3.04967300 | -3.28794100 |
| H | -5.98576100 | -2.20051900 | -1.78754000 |
| H | -5.64867800 | -1.28542200 | -3.25703000 |
| C | 1.06615000 | 2.45144800 | -0.51141500 |
| O | 1.22023800 | 3.58968000 | -0.64584300 |

**Ni[P(n-Bu)$_3$](Pyr)(CO)(PhOMe), CO-P trans**

| | | | |
|---|---|---|---|
| Ni | -0.14755800 | -1.97136600 | -0.00643800 |
| C | 0.15738800 | -3.66310600 | 0.32434600 |
| C | -2.09332500 | -2.17030200 | 0.01197700 |
| P | -0.45801500 | 0.26234000 | -0.19554700 |
| C | -2.03122900 | 0.80380500 | -0.98234500 |
| C | -0.52252700 | 0.79601200 | 1.56808500 |
| C | 0.85355000 | 1.26250900 | -1.01690600 |
| C | -1.27798300 | 2.06882000 | 1.92881500 |
| C | -1.22010300 | 2.36378600 | 3.42196200 |
| C | 1.16474300 | 2.64655600 | -0.45982600 |
| C | 2.34251600 | 3.28952700 | -1.18121500 |
| C | -2.17650600 | 2.26436000 | -1.39241000 |
| C | -1.99857000 | 3.60967000 | 3.80396200 |
| C | 2.68537500 | 4.66582900 | -0.64094500 |
| C | -2.79159600 | -2.41573500 | -1.17939700 |
| N | -4.12576800 | -2.47910100 | -1.30315100 |
| C | -4.84481300 | -2.30434500 | -0.18771900 |
| C | -2.88535200 | -2.02341100 | 1.15700200 |

| | | | |
|---|---|---|---|
| C | -4.27499000 | -2.08361500 | 1.06238400 |
| C | -3.52416000 | 2.53457200 | -2.05038200 |
| C | -3.68467100 | 3.97835600 | -2.49037400 |
| C | 1.78614600 | -1.65303900 | -0.05692200 |
| H | 0.52163300 | 0.85429600 | 1.90275100 |
| H | -0.95849300 | -0.04936700 | 2.11684200 |
| H | -0.87401200 | 2.92536900 | 1.37457200 |
| H | -2.32814400 | 1.97469900 | 1.61815400 |
| H | 0.56991100 | 1.32942900 | -2.07575300 |
| H | 1.76273700 | 0.65384900 | -0.99172600 |
| H | 0.28869000 | 3.30594500 | -0.52984000 |
| H | 1.40168400 | 2.56902300 | 0.61134000 |
| H | -2.84181400 | 0.50201500 | -0.30610600 |
| H | -2.15545000 | 0.16260800 | -1.86502800 |
| H | -2.06030200 | 2.92691800 | -0.52526600 |
| H | -1.37308600 | 2.54044000 | -2.08976000 |
| H | -0.16969900 | 2.46934800 | 3.72596000 |
| H | -1.60402300 | 1.49628600 | 3.97569800 |
| H | -1.61221700 | 4.49354900 | 3.28510400 |
| H | -3.05646800 | 3.51300000 | 3.53741100 |
| H | 2.11623800 | 3.35311800 | -2.25417400 |
| H | 3.21413900 | 2.62461800 | -1.10064800 |
| H | 3.53277900 | 5.11060300 | -1.16967600 |
| H | 1.83844600 | 5.35357900 | -0.73878500 |
| H | 2.94621400 | 4.62115300 | 0.42186300 |
| H | -1.94585300 | 3.80873400 | 4.87768300 |
| H | -2.23946300 | -2.55855800 | -2.11201000 |
| H | -5.92791700 | -2.35109000 | -0.30118200 |
| H | -2.42567500 | -1.85341400 | 2.13176400 |
| H | -4.90557100 | -1.96362800 | 1.94017700 |
| H | -4.32503200 | 2.26385200 | -1.34918400 |
| H | -3.64510400 | 1.86286300 | -2.91063600 |
| H | -4.65836600 | 4.15569800 | -2.95481200 |
| H | -3.59413800 | 4.66454500 | -1.64141100 |
| H | -2.91587200 | 4.25912500 | -3.21828000 |
| O | 0.38265900 | -4.77255400 | 0.55679200 |
| C | 2.49290500 | -1.81716000 | -1.25856700 |
| C | 3.81690500 | -1.41462400 | -1.39137600 |
| C | 4.48631800 | -0.83789100 | -0.30635700 |
| C | 2.49092300 | -1.11505200 | 1.02267300 |
| C | 3.82247000 | -0.69710800 | 0.91355700 |
| H | 1.99651600 | -2.24845100 | -2.12764400 |
| H | 4.35403400 | -1.53304300 | -2.32912800 |
| O | 5.77654300 | -0.45228000 | -0.53724800 |
| H | 1.99853400 | -0.99006900 | 1.98764200 |
| H | 4.32446900 | -0.26981000 | 1.77643600 |
| C | 6.45921500 | 0.15731300 | 0.53833000 |
| H | 7.45374600 | 0.40711200 | 0.16880700 |
| H | 5.95622300 | 1.07563500 | 0.86997600 |
| H | 6.55354600 | -0.51882500 | 1.39792100 |

**TS[Pyr-PhOMe], reductive eliminination**

| | | | |
|---|---|---|---|
| Ni | -1.33141100 | -1.35063500 | -0.45072000 |
| C | -1.31411400 | -3.04354800 | -0.99958600 |
| P | 0.92800100 | -0.92353700 | -0.26207200 |
| C | 2.19945100 | -2.08360200 | -0.92698800 |
| C | 1.34148400 | 0.73097300 | -0.95015400 |
| C | 1.25311400 | -0.84067600 | 1.55680200 |
| C | 3.58715600 | -2.08631500 | -0.29482200 |
| C | 4.52008600 | -3.09317900 | -0.95559100 |
| C | 5.89833400 | -3.12475600 | -0.32026300 |
| C | 2.78274300 | 1.17615800 | -1.15083400 |
| C | 2.24453600 | 0.18809800 | 2.08622700 |
| C | 2.23896900 | 0.26191300 | 3.60734200 |
| C | 3.22689200 | 1.27849400 | 4.15024800 |
| C | 2.83964600 | 2.62367600 | -1.62659600 |
| C | 4.25307700 | 3.11332800 | -1.88219100 |
| C | -1.76759600 | 0.50777000 | -0.09957300 |
| C | -3.16287600 | -0.93700700 | -0.00482000 |
| C | -3.54520100 | -1.28050800 | 1.30616800 |
| N | -4.80211900 | -1.48328900 | 1.71110400 |
| C | -5.77125100 | -1.33503400 | 0.79876000 |
| C | -4.20632000 | -0.78143600 | -0.92981800 |
| C | -5.51910500 | -0.98664100 | -0.52719700 |
| C | -1.52645000 | 1.16544000 | 1.12266100 |
| C | -1.26018400 | 2.52471300 | 1.17730400 |
| C | -1.25880800 | 3.29228000 | 0.00450400 |
| C | -1.83016300 | 1.31078500 | -1.24941300 |
| C | -1.57040700 | 2.68028000 | -1.21248700 |
| O | -0.96240700 | 4.61492100 | 0.15528500 |
| C | -0.93345500 | 5.39765000 | -1.02137400 |
| H | 2.27003000 | -1.87667300 | -2.00367100 |
| H | 1.76518500 | -3.08958900 | -0.85502600 |
| H | 4.03796800 | -1.08689900 | -0.34698900 |
| H | 3.50554200 | -2.31985400 | 0.77623700 |
| H | 4.60540000 | -2.85493600 | -2.02460600 |
| H | 4.06263300 | -4.09074000 | -0.91011000 |
| H | 6.38620300 | -2.14608200 | -0.38267300 |
| H | 5.84018900 | -3.39154500 | 0.74040200 |
| H | 0.80456200 | 1.44626700 | -0.31243400 |
| H | 0.81536800 | 0.78138000 | -1.91245800 |
| H | 3.35920900 | 1.08094500 | -0.22234000 |
| H | 3.28137800 | 0.52596400 | -1.88405200 |
| H | 1.53888700 | -1.85348200 | 1.86984900 |
| H | 0.27654700 | -0.65967100 | 2.02239800 |
| H | 3.25944100 | -0.03992400 | 1.73177000 |
| H | 1.99809900 | 1.18137500 | 1.68324400 |
| H | 2.45653100 | -0.73218500 | 4.02088300 |
| H | 1.22242500 | 0.50893900 | 3.94534400 |
| H | 4.25199100 | 1.03438700 | 3.85138700 |
| H | 3.00894600 | 2.28310500 | 3.77202400 |
| H | 6.55454100 | -3.85141100 | -0.80696500 |
| H | 3.20447800 | 1.32569400 | 5.24232000 |
| H | 2.34723800 | 3.25916400 | -0.87539800 |
| H | 2.23315900 | 2.72658900 | -2.53770900 |
| H | 4.27055700 | 4.15603800 | -2.21073300 |
| H | 4.86649400 | 3.04231100 | -0.97769000 |
| H | 4.74586900 | 2.51530700 | -2.65631600 |
| H | -2.78000000 | -1.40131200 | 2.07654600 |
| H | -6.79048600 | -1.49691000 | 1.14716000 |
| H | -3.99210500 | -0.49710200 | -1.95835900 |
| H | -6.33971000 | -0.87719400 | -1.23172000 |
| H | -1.53793800 | 0.60349900 | 2.05501300 |
| H | -1.05143700 | 3.01787800 | 2.12313000 |
| H | -2.05692400 | 0.85643300 | -2.21330900 |
| H | -1.60332000 | 3.25402800 | -2.13330300 |
| H | -0.65305200 | 6.40447400 | -0.71289700 |
| H | -0.19456700 | 5.01984200 | -1.74081100 |
| H | -1.91456600 | 5.43060400 | -1.51217100 |
| O | -1.21231600 | -4.12591900 | -1.40682200 |

**Ni[P(n-Bu)$_3$](CO)[Pyr-PhOMe]**

| | | | |
|---|---|---|---|
| Ni | 0.55839900 | -1.33451500 | 1.04696300 |
| P | -1.28584200 | -0.20952400 | 0.43098300 |

| | | | |
|---|---|---|---|
| C | 1.10577800 | -1.01015900 | 2.68109400 |
| C | -1.07228300 | 0.75182300 | -1.13525300 |
| C | -2.78511600 | -1.26456700 | 0.17483000 |
| C | -1.78786100 | 1.04427400 | 1.69352100 |
| C | -3.91199000 | -0.77954500 | -0.72887300 |
| C | -5.02821800 | -1.80959700 | -0.84717400 |
| C | -3.22491900 | 1.54893900 | 1.72618200 |
| C | -3.44143900 | 2.60528100 | 2.80244700 |
| C | -1.93333000 | 1.97475600 | -1.42158800 |
| C | -1.55370900 | 2.63823700 | -2.74036400 |
| C | -6.14848600 | -1.36336300 | -1.76932900 |
| C | -2.38594100 | 3.87101700 | -3.04254900 |
| C | -4.87991500 | 3.08463300 | 2.87585500 |
| C | 2.97170800 | -0.59382600 | -0.70711000 |
| C | 1.91285500 | -1.61437800 | -0.81496300 |
| C | 1.01497000 | -1.64236100 | -1.90604200 |
| N | 0.01161300 | -2.50556200 | -2.07797000 |
| C | -0.17402800 | -3.39305000 | -1.08293200 |
| C | 1.80001000 | -2.67560500 | 0.13904800 |
| C | 0.67426200 | -3.52391200 | 0.01266500 |
| C | 2.93632200 | 0.59767600 | -1.45860200 |
| C | 3.92599600 | 1.55799300 | -1.34534900 |
| C | 4.99820800 | 1.36584600 | -0.46612600 |
| C | 4.05161200 | -0.75717800 | 0.17033600 |
| C | 5.05532300 | 0.19611400 | 0.29580800 |
| O | 5.91933400 | 2.36311200 | -0.42043200 |
| C | 7.00419300 | 2.19622400 | 0.47431700 |
| H | -3.16954000 | -1.49679000 | 1.17776000 |
| H | -2.40297900 | -2.21569300 | -0.21479000 |
| H | -4.33136600 | 0.16625400 | -0.36221700 |
| H | -3.51192000 | -0.56521200 | -1.73004600 |
| H | -1.08798400 | 1.88334400 | 1.57461200 |
| H | -1.53621700 | 0.60258600 | 2.66593500 |
| H | -3.51405400 | 1.96462100 | 0.75252700 |
| H | -3.90773200 | 0.70611400 | 1.90482600 |
| H | -1.15923500 | 0.01554600 | -1.94609600 |
| H | -0.01553100 | 1.05278400 | -1.13443400 |
| H | -2.99727600 | 1.70586700 | -1.44705000 |
| H | -1.82689900 | 2.70629600 | -0.60767900 |
| H | -5.42827700 | -2.02118600 | 0.15401700 |
| H | -4.60414600 | -2.75802100 | -1.20371200 |
| H | -6.60789700 | -0.43446400 | -1.41467700 |
| H | -5.77582300 | -1.17529800 | -2.78196300 |
| H | -1.65925100 | 1.90595300 | -3.55252200 |
| H | -0.48695200 | 2.90134800 | -2.71614600 |
| H | -2.10137200 | 4.33261100 | -3.99216400 |
| H | -2.77251200 | 3.45497600 | 2.60829300 |
| H | -3.13013500 | 2.19986800 | 3.77464300 |
| H | -5.01748500 | 3.84811700 | 3.64634800 |
| H | -5.20318900 | 3.51641000 | 1.92247800 |
| H | -5.56150700 | 2.25824200 | 3.10471800 |
| H | -3.45143200 | 3.62437500 | -3.10177300 |
| H | -2.27150800 | 4.62857100 | -2.25991200 |
| H | -6.93963800 | -2.11456600 | -1.84399700 |
| H | 1.14221000 | -0.91305500 | -2.70675600 |
| H | -1.02889900 | -4.06068300 | -1.18811300 |
| H | 2.60411100 | -2.92683400 | 0.82085100 |
| H | 0.51262700 | -4.32169000 | 0.73130300 |
| H | 2.11055200 | 0.78714000 | -2.14020600 |
| H | 3.88586900 | 2.47437100 | -1.92603000 |
| H | 4.12717600 | -1.65556900 | 0.77567000 |
| H | 5.87258200 | 0.01876800 | 0.98624200 |
| H | 7.62421800 | 3.08586800 | 0.36902300 |
| H | 6.66129700 | 2.11418000 | 1.51290600 |
| H | 7.59894400 | 1.30867800 | 0.22615000 |
| O | 1.35420600 | -0.68651800 | 3.77655200 |

**Ni[P(n-Bu)$_3$](CO)**

| | | | |
|---|---|---|---|
| Ni | -2.81130600 | -0.66271400 | -0.04151100 |
| P | -0.61725700 | -0.36310300 | 0.00956800 |
| C | -4.43492500 | -0.13641300 | -0.13964100 |
| C | -0.09533500 | 0.36467900 | 1.63132300 |
| C | 0.49831800 | -1.82127900 | -0.22374700 |
| C | -0.12020600 | 0.87084000 | -1.27732200 |
| C | 1.93175100 | -1.76742600 | 0.29364200 |
| C | 2.72579300 | -3.03339900 | -0.05670800 |
| C | 1.29055000 | 0.82775000 | -1.85256900 |
| C | 1.48894600 | 1.84704500 | -2.96703800 |
| C | 1.13294500 | 1.27265300 | 1.68647800 |
| C | 1.13895200 | 2.19586500 | 2.90444800 |
| C | 3.65134000 | -3.48735700 | 1.05849800 |
| C | 2.07493300 | 3.38608900 | 2.73485500 |
| C | 2.90247700 | 1.84512400 | -3.52060700 |
| H | 0.48584600 | -2.05032500 | -1.29816200 |
| H | -0.01714600 | -2.66576100 | 0.25032500 |
| H | 2.44444200 | -0.88425700 | -0.10280900 |
| H | 1.91905100 | -1.63504200 | 1.38409200 |
| H | -0.31720600 | 1.85763600 | -0.83727000 |
| H | -0.85144400 | 0.77001600 | -2.08783700 |
| H | 2.03123500 | 1.01087500 | -1.06219100 |
| H | 1.50956700 | -0.17805700 | -2.23855400 |
| H | 0.02829700 | -0.49239900 | 2.30676700 |
| H | -0.97059000 | 0.90477900 | 2.01105300 |
| H | 2.04421900 | 0.66100500 | 1.69769600 |
| H | 1.20410100 | 1.88566400 | 0.77797400 |
| H | 3.30979800 | -2.85629000 | -0.96835000 |
| H | 2.02840000 | -3.84252700 | -0.31082500 |
| H | 4.37971400 | -2.70954300 | 1.31226100 |
| H | 3.08719400 | -3.71230600 | 1.96997300 |
| H | 1.41865700 | 1.62267900 | 3.79725200 |
| H | 0.11493300 | 2.54437200 | 3.09155000 |
| H | 2.42407900 | 3.77924500 | 3.69324900 |
| H | 1.23633300 | 2.84649300 | -2.58739100 |
| H | 0.76937700 | 1.64531000 | -3.77177300 |
| H | 3.01310100 | 2.53805200 | -4.35912900 |
| H | 3.62984600 | 2.13530900 | -2.75492700 |
| H | 3.18677600 | 0.84900800 | -3.87711500 |
| H | 2.96335000 | 3.11036200 | 2.15603200 |
| H | 1.58690000 | 4.20690100 | 2.20095400 |
| H | 4.21210500 | -4.38560400 | 0.78496900 |
| O | -5.50109500 | 0.35774100 | -0.22359000 |

**[Pyr-PhOMe]**

| | | | |
|---|---|---|---|
| C | 1.62785200 | 0.01649800 | 0.00511900 |
| C | 0.16058600 | 0.09843500 | -0.01321200 |
| C | -0.49470800 | 1.30814400 | -0.30178500 |
| C | -1.87482100 | 1.39469300 | -0.32278300 |
| C | -2.65398900 | 0.26270500 | -0.05225100 |
| C | -0.63679300 | -1.02037300 | 0.25572600 |
| C | -2.02617700 | -0.95152500 | 0.23881400 |
| H | 0.09192900 | 2.19196800 | -0.53996700 |
| O | -3.99938500 | 0.44379300 | -0.09692900 |
| H | -0.16475100 | -1.96587600 | 0.51146800 |
| H | -2.60565800 | -1.83979300 | 0.46445400 |

| | | | |
|---|---|---|---|
| H | -2.37742000 | 2.32819400 | -0.55664400 |
| C | -4.81097700 | -0.68942500 | 0.15358900 |
| H | -5.84223000 | -0.35012600 | 0.06315500 |
| H | -4.62647600 | -1.48712100 | -0.57632200 |
| H | -4.64829600 | -1.08780900 | 1.16256400 |
| C | 2.43135800 | 1.10816400 | 0.35866300 |
| C | 3.81259900 | 0.97327700 | 0.35410100 |
| C | 4.36269700 | -0.25620900 | 0.00357300 |
| C | 2.30114100 | -1.16599600 | -0.34243100 |
| N | 3.62607700 | -1.31841900 | -0.34373600 |
| H | 1.97281800 | 2.04807200 | 0.65714700 |
| H | 4.45424200 | 1.80436300 | 0.63069500 |
| H | 5.44254000 | -0.39775200 | -0.00046600 |
| H | 1.72323000 | -2.03585400 | -0.65724900 |

**Ni[P(n-Bu)$_3$](PyrCO)[PhO-B(OH)$_2$-PhOMe], iso1**

| | | | |
|---|---|---|---|
| Ni | -0.61661200 | -0.63880600 | -0.40421400 |
| O | -2.19286600 | -1.78989600 | -0.00661800 |
| B | -1.47091600 | -2.22276500 | 1.32900400 |
| C | 0.02149300 | -2.54510700 | 0.73547400 |
| C | -1.53932800 | 0.61502600 | -1.41415000 |
| P | 1.10781200 | 0.69108700 | -0.25539200 |
| C | 2.64119500 | -0.03068300 | -0.97493900 |
| C | 0.96366000 | 2.37613300 | -0.98628400 |
| C | 1.40859400 | 0.92221600 | 1.54963600 |
| C | 2.24200900 | 3.16542300 | -1.24485000 |
| C | 1.95232400 | 4.54424500 | -1.82480300 |
| C | 2.13673600 | 2.17211500 | 2.02580600 |
| C | 2.25569400 | 2.21357300 | 3.54400700 |
| C | 3.97164600 | 0.29670400 | -0.30589000 |
| C | 3.21466000 | 5.33297900 | -2.12203600 |
| C | 2.95500100 | 3.46430600 | 4.04455600 |
| C | -3.50370300 | -1.45126800 | -0.13374400 |
| C | -4.02422700 | -1.35011000 | -1.42907800 |
| C | -5.34454600 | -0.96210200 | -1.62424500 |
| C | -6.16583300 | -0.67540100 | -0.53463800 |
| C | -4.32958500 | -1.18298400 | 0.96208400 |
| C | -5.65012500 | -0.79531400 | 0.75377500 |
| C | 1.16523000 | -2.48359500 | 1.56400300 |
| C | 2.40367300 | -2.95118100 | 1.16064300 |
| C | 2.55855100 | -3.49874000 | -0.12358500 |
| C | 0.22324500 | -3.09055300 | -0.54878900 |
| C | 1.46493700 | -3.55818900 | -0.98873400 |
| O | 3.81329500 | -3.91721100 | -0.43773800 |
| O | -2.24151500 | -3.32731900 | 1.82413100 |
| O | -1.32285500 | -1.10043100 | 2.24566400 |
| C | 4.02634500 | -4.37492400 | -1.76213500 |
| C | 5.12881900 | -0.46805700 | -0.93622500 |
| C | 6.46447600 | -0.14524900 | -0.29097700 |
| H | 0.28423300 | 2.93983900 | -0.33197700 |
| H | 0.42184600 | 2.25122400 | -1.93167900 |
| H | 2.82574400 | 3.27907600 | -0.32305900 |
| H | 2.88541200 | 2.60820800 | -1.94026200 |
| H | 1.93622500 | 0.01697000 | 1.87392700 |
| H | 0.42211700 | 0.84877300 | 2.02497900 |
| H | 3.14069200 | 2.22497700 | 1.58312100 |
| H | 1.60635300 | 3.07106400 | 1.68059500 |
| H | 2.65244800 | 0.25909800 | -2.03376300 |
| H | 2.48790500 | -1.11604200 | -0.96905700 |
| H | 4.17332400 | 1.37543800 | -0.35251100 |
| H | 3.92088400 | 0.04509600 | 0.76304100 |
| H | 1.31837500 | 5.10122200 | -1.12156700 |
| H | 1.35428900 | 4.43233800 | -2.73903600 |
| H | 3.81352900 | 5.47870700 | -1.21662600 |
| H | 3.84625700 | 4.81112600 | -2.84894200 |
| H | 2.79449400 | 1.31917300 | 3.88504400 |
| H | 1.25246800 | 2.14191600 | 3.98470400 |
| H | 3.03612500 | 3.47584500 | 5.13477500 |
| H | 3.96879400 | 3.54342100 | 3.63733900 |
| H | 2.41301500 | 4.36765700 | 3.74488700 |
| H | 2.98970900 | 6.32164700 | -2.53105200 |
| H | -3.37096100 | -1.56835400 | -2.26952700 |
| H | -5.73338100 | -0.88409500 | -2.63643800 |
| H | -7.19644700 | -0.36891300 | -0.68782700 |
| H | -3.94261600 | -1.29265700 | 1.96809600 |
| H | -6.27983800 | -0.58169400 | 1.61364300 |
| H | 1.05403900 | -2.06220600 | 2.56172800 |
| H | 3.27410400 | -2.90463800 | 1.81032100 |
| H | -0.63339900 | -3.18376900 | -1.21446000 |
| H | 1.56447200 | -3.96913900 | -1.98781000 |
| H | -2.10094000 | -1.02922300 | 2.80553500 |
| H | -1.81706700 | -3.68095300 | 2.60811100 |
| H | 5.08506500 | -4.62272300 | -1.83022200 |
| H | 3.42897400 | -5.26712200 | -1.98306000 |
| H | 3.78704600 | -3.59465500 | -2.49632600 |
| H | 5.16644100 | -0.24486200 | -2.01143100 |
| H | 4.92524200 | -1.54449300 | -0.85768800 |
| H | 7.28432800 | -0.70251000 | -0.75220200 |
| H | 6.70102500 | 0.92083800 | -0.37633200 |
| H | 6.45762200 | -0.39234700 | 0.77601200 |
| C | -2.43410200 | 1.55544700 | -0.68692300 |
| O | -1.49154300 | 0.57321400 | -2.63192400 |
| C | -2.39816000 | 1.65736500 | 0.70704900 |
| N | -3.18207100 | 2.47673500 | 1.41411900 |
| C | -4.05308100 | 3.22163500 | 0.72152200 |
| C | -3.35650800 | 2.33896000 | -1.38701400 |
| C | -4.18352600 | 3.18714200 | -0.66713200 |
| H | -1.71371300 | 1.01680600 | 1.27050400 |
| H | -4.68823000 | 3.88140500 | 1.31144000 |
| H | -3.40878800 | 2.25727800 | -2.46958100 |
| H | -4.91970200 | 3.81245800 | -1.16279400 |

**Ni[P(n-Bu)$_3$](PyrCO)[PhO-B(OH)$_2$-PhOMe], iso2**

| | | | |
|---|---|---|---|
| Ni | -0.61384500 | -0.65440100 | -0.42672000 |
| O | -2.17956100 | -1.79389900 | -0.01739500 |
| B | -1.44001200 | -2.24148900 | 1.36879700 |
| C | 0.03275300 | -2.55432200 | 0.73487300 |
| C | -1.54719000 | 0.60024700 | -1.42530700 |
| P | 1.10342700 | 0.68648200 | -0.26477500 |
| C | 2.65337200 | -0.01790900 | -0.96641700 |
| C | 0.95232400 | 2.37112800 | -0.99553800 |
| C | 1.38048500 | 0.91683600 | 1.54426900 |
| C | 2.22735800 | 3.17028100 | -1.23918000 |
| C | 1.93506700 | 4.54654600 | -1.82376400 |
| C | 2.09422300 | 2.17079800 | 2.03149400 |
| C | 2.19713400 | 2.20881000 | 3.55097200 |
| C | 3.97235300 | 0.32002000 | -0.28013200 |
| C | 3.19568600 | 5.34246600 | -2.10897100 |
| C | 2.87998900 | 3.46430600 | 4.06216900 |
| C | -3.48809900 | -1.44996900 | -0.13811200 |
| C | -4.03208300 | -1.37748600 | -1.42668600 |
| C | -5.35630700 | -0.99513200 | -1.60758500 |

| | | | |
|---|---|---|---|
| C | -6.15940300 | -0.68711300 | -0.51008600 |
| C | -4.29579700 | -1.15596700 | 0.96576700 |
| C | -5.62087400 | -0.77637900 | 0.77159400 |
| C | 1.17700000 | -2.50013700 | 1.56446000 |
| C | 2.41713400 | -2.95386600 | 1.15400900 |
| C | 2.57453800 | -3.48432900 | -0.13779700 |
| C | 0.23711900 | -3.08006300 | -0.55752500 |
| C | 1.48180200 | -3.53716600 | -1.00340500 |
| O | 3.83100100 | -3.89238100 | -0.45686600 |
| O | -2.16465000 | -3.31902500 | 1.95455100 |
| O | -1.34747500 | -1.12584100 | 2.27263300 |
| C | 4.04763200 | -4.32906300 | -1.78782800 |
| C | 5.14363900 | -0.43611200 | -0.89478900 |
| C | 6.46842200 | -0.10151000 | -0.23330700 |
| H | 0.26163800 | 2.92897900 | -0.34814900 |
| H | 0.42130800 | 2.24262200 | -1.94666000 |
| H | 2.79831300 | 3.28854800 | -0.30992300 |
| H | 2.88368500 | 2.61758100 | -1.92606200 |
| H | 1.90935900 | 0.01418300 | 1.87409900 |
| H | 0.38864800 | 0.83649300 | 2.00724400 |
| H | 3.10222200 | 2.23287100 | 1.59926100 |
| H | 1.56024600 | 3.06642300 | 1.68320900 |
| H | 2.67542100 | 0.27381100 | -2.02455700 |
| H | 2.51028800 | -1.10448300 | -0.96456400 |
| H | 4.16662300 | 1.40018500 | -0.32474000 |
| H | 3.90957200 | 0.06845900 | 0.78818800 |
| H | 1.29045400 | 5.10053400 | -1.12795100 |
| H | 1.34737300 | 4.42982800 | -2.74412300 |
| H | 3.78462000 | 5.49236400 | -1.19773100 |
| H | 3.83756100 | 4.82365200 | -2.82904200 |
| H | 2.74049900 | 1.31828600 | 3.89485200 |
| H | 1.19016200 | 2.12654900 | 3.98100800 |
| H | 2.95124100 | 3.47270500 | 5.15310500 |
| H | 3.89660600 | 3.55436100 | 3.66432800 |
| H | 2.33228000 | 4.36362100 | 3.76080700 |
| H | 2.96918500 | 6.32949500 | -2.52108400 |
| H | -3.39200800 | -1.60918300 | -2.27396800 |
| H | -5.76237300 | -0.93672800 | -2.61443300 |
| H | -7.19312100 | -0.38545700 | -0.65229300 |
| H | -3.87843700 | -1.21449700 | 1.96326300 |
| H | -6.23554200 | -0.54100900 | 1.63676600 |
| H | 1.06042100 | -2.09593800 | 2.56849600 |
| H | 3.28742500 | -2.91208900 | 1.80410300 |
| H | -0.61631800 | -3.16683600 | -1.22845900 |
| H | 1.58299800 | -3.93316500 | -2.00833200 |
| H | -1.99591200 | -1.23006300 | 2.97385000 |
| H | 5.10760100 | -4.57054100 | -1.85851500 |
| H | 3.45485100 | -5.22081100 | -2.02260400 |
| H | 3.80517500 | -3.53887200 | -2.51027700 |
| H | 5.19302400 | -0.21410300 | -1.96981600 |
| H | 4.94808300 | -1.51410200 | -0.81737600 |
| H | 7.29842500 | -0.65317500 | -0.68302000 |
| H | 6.69755300 | 0.96625900 | -0.31773100 |
| H | 6.44997500 | -0.34667200 | 0.83399300 |
| C | -2.43326800 | 1.54732500 | -0.69789000 |
| O | -1.50163800 | 0.55599200 | -2.64328100 |
| C | -2.41908500 | 1.62811400 | 0.69820800 |
| N | -3.19525700 | 2.45767900 | 1.40232300 |
| C | -4.03811100 | 3.23026500 | 0.70555700 |
| C | -3.32687900 | 2.35965300 | -1.40257200 |
| C | -4.14867300 | 3.21473300 | -0.68514700 |
| H | -1.76786100 | 0.95751400 | 1.26661900 |
| H | -4.66656000 | 3.89815400 | 1.29366000 |
| H | -3.36109100 | 2.29407700 | -2.48703800 |
| H | -4.86339500 | 3.86194700 | -1.18409200 |
| H | -2.19209200 | -4.07091600 | 1.36010100 |

**TS[transmet], iso2**

| | | | |
|---|---|---|---|
| Ni | -0.60129700 | -0.54824000 | -0.39043700 |
| O | -2.11974500 | -1.90525500 | -0.16258300 |
| B | -1.58167100 | -2.52808500 | 1.02048300 |
| C | 0.63179100 | -2.05608000 | 0.14249500 |
| C | -1.73137800 | 0.76629900 | -1.15883000 |
| P | 1.03120600 | 0.87883500 | -0.30003200 |
| C | 2.64886300 | 0.24164100 | -0.92364500 |
| C | 0.79699900 | 2.47654400 | -1.18812800 |
| C | 1.27144800 | 1.30585600 | 1.48135000 |
| C | 2.01430700 | 3.34367800 | -1.49033300 |
| C | 1.63643800 | 4.60285100 | -2.26038900 |
| C | 1.93224600 | 2.62739600 | 1.85327600 |
| C | 2.03404500 | 2.80250700 | 3.36336500 |
| C | 3.92698900 | 0.71896000 | -0.24428000 |
| C | 2.83749000 | 5.46746000 | -2.59889500 |
| C | 2.66678300 | 4.12348600 | 3.76156500 |
| C | -3.47609400 | -1.66821200 | -0.32577100 |
| C | -3.92771900 | -1.45975400 | -1.62693200 |
| C | -5.27397400 | -1.18743500 | -1.84423500 |
| C | -6.16614200 | -1.13310900 | -0.77452000 |
| C | -4.35323200 | -1.61806300 | 0.75548100 |
| C | -5.70024200 | -1.35263800 | 0.51922700 |
| C | 1.47748400 | -2.15369000 | 1.26559900 |
| C | 2.63256500 | -2.92567900 | 1.28116800 |
| C | 2.99112700 | -3.66076000 | 0.14545500 |
| C | 1.00633900 | -2.85019000 | -0.95588300 |
| C | 2.15767800 | -3.64337000 | -0.97643200 |
| O | 4.16406900 | -4.35483400 | 0.22905000 |
| O | -1.46379900 | -3.88744700 | 1.08295500 |
| O | -1.59506500 | -1.78145000 | 2.17091200 |
| C | 4.58104000 | -5.04229700 | -0.93300300 |
| C | 5.15388300 | -0.00592800 | -0.78459500 |
| C | 6.44123200 | 0.43407200 | -0.11149900 |
| H | 0.04437400 | 3.04130000 | -0.61933200 |
| H | 0.30023900 | 2.20934800 | -2.12842700 |
| H | 2.53773400 | 3.62994500 | -0.56948500 |
| H | 2.74105800 | 2.76656800 | -2.07924000 |
| H | 1.82653000 | 0.46633900 | 1.91699400 |
| H | 0.27327100 | 1.25982400 | 1.93557400 |
| H | 2.93647500 | 2.69187000 | 1.41488700 |
| H | 1.36124700 | 3.46570800 | 1.42997700 |
| H | 2.67336100 | 0.47227800 | -1.99712800 |
| H | 2.59750200 | -0.84875000 | -0.85331200 |
| H | 4.05813700 | 1.80287400 | -0.36646900 |
| H | 3.86016900 | 0.54119100 | 0.83888800 |
| H | 0.91304600 | 5.18100800 | -1.66953300 |
| H | 1.10750300 | 4.31746700 | -3.17958200 |
| H | 3.36175100 | 5.79094000 | -1.69314900 |
| H | 3.55958400 | 4.91913900 | -3.21356400 |
| H | 2.61287900 | 1.96780600 | 3.78187500 |
| H | 1.03139800 | 2.71875800 | 3.80385200 |
| H | 2.73601100 | 4.23003400 | 4.84748300 |
| H | 3.67982900 | 4.21745300 | 3.35561000 |
| H | 2.08557900 | 4.97136400 | 3.38352000 |
| H | 2.54990600 | 6.36604800 | -3.15147000 |
| H | -3.21506100 | -1.49192600 | -2.44493800 |

| | | | |
|---|---|---|---|
| H | -5.62528300 | -1.01763200 | -2.85820400 |
| H | -7.21645600 | -0.91998600 | -0.94894700 |
| H | -3.97951600 | -1.75559800 | 1.76457100 |
| H | -6.38516700 | -1.30742600 | 1.36134600 |
| H | 1.22598400 | -1.59858100 | 2.17114700 |
| H | 3.28047100 | -2.97174400 | 2.15350500 |
| H | 0.39134200 | -2.83663300 | -1.85898200 |
| H | 2.40421000 | -4.22411700 | -1.86055500 |
| H | -1.26782400 | -2.29921200 | 2.91241000 |
| H | 5.54453100 | -5.49239400 | -0.69370800 |
| H | 3.87495300 | -5.83352100 | -1.21599500 |
| H | 4.70170700 | -4.35958600 | -1.78460600 |
| H | 5.22174500 | 0.15820100 | -1.86874100 |
| H | 5.01195900 | -1.08871600 | -0.65536500 |
| H | 7.31064400 | -0.09728100 | -0.50842800 |
| H | 6.61710300 | 1.50607400 | -0.25216900 |
| H | 6.40661400 | 0.24935300 | 0.96756600 |
| C | -2.70265900 | 1.48533000 | -0.27178000 |
| O | -1.78530700 | 0.94319200 | -2.37527000 |
| C | -2.62879100 | 1.35290100 | 1.11723700 |
| N | -3.47062400 | 1.93351300 | 1.97765500 |
| C | -4.44566200 | 2.68080100 | 1.44474000 |
| C | -3.73259700 | 2.26922500 | -0.79958600 |
| C | -4.62125800 | 2.87728100 | 0.07415800 |
| H | -1.85007300 | 0.71639000 | 1.54660500 |
| H | -5.13014300 | 3.14572900 | 2.15369700 |
| H | -3.81366000 | 2.37154300 | -1.87940900 |
| H | -5.44072100 | 3.49190000 | -0.28702900 |
| H | -1.41921600 | -4.27746300 | 0.20616500 |

**TS[transmet], iso1**

| | | | |
|---|---|---|---|
| Ni | -0.63525800 | -0.37612200 | -0.36597600 |
| O | -2.23503900 | -1.62436000 | -0.23493300 |
| B | -1.78550800 | -2.39241400 | 0.89022200 |
| C | 0.46333400 | -2.00508500 | 0.15457800 |
| C | -1.70819400 | 1.06105600 | -0.99610100 |
| P | 1.12494300 | 0.89613000 | -0.32338000 |
| C | 2.66480900 | 0.07930000 | -0.93537600 |
| C | 1.04154800 | 2.47791600 | -1.26065800 |
| C | 1.40938200 | 1.37565500 | 1.43699200 |
| C | 2.34373300 | 3.18794500 | -1.61471100 |
| C | 2.09476500 | 4.49170800 | -2.36306500 |
| C | 2.26424400 | 2.59615200 | 1.75715700 |
| C | 2.38832200 | 2.82319500 | 3.25851700 |
| C | 3.98114200 | 0.36979600 | -0.22427200 |
| C | 3.37915200 | 5.19680200 | -2.75987700 |
| C | 3.21806300 | 4.04744000 | 3.60077800 |
| C | -3.56629300 | -1.37322100 | -0.49861400 |
| C | -3.90234600 | -1.05172600 | -1.81128100 |
| C | -5.22311600 | -0.74660800 | -2.12059800 |
| C | -6.20365100 | -0.76868700 | -1.12964800 |
| C | -4.53301900 | -1.40200800 | 0.50426500 |
| C | -5.85265300 | -1.10009800 | 0.17683800 |
| C | 1.29946100 | -2.11710100 | 1.28364000 |
| C | 2.42354300 | -2.93465200 | 1.32053300 |
| C | 2.75216900 | -3.70791100 | 0.20161600 |
| C | 0.80432000 | -2.84141500 | -0.92324800 |
| C | 1.91930700 | -3.68300500 | -0.92159700 |
| O | 3.89622200 | -4.44727300 | 0.30194600 |
| O | -1.86753800 | -3.74664600 | 0.71492500 |
| O | -1.75089700 | -1.71969200 | 2.09280300 |
| C | 4.27617700 | -5.19092700 | -0.83845400 |
| C | 5.09491000 | -0.54743000 | -0.71488800 |
| C | 6.41845100 | -0.29330300 | -0.01653900 |
| H | 0.37778400 | 3.14056500 | -0.68705500 |
| H | 0.48262500 | 2.24794700 | -2.17547100 |
| H | 2.94060400 | 3.39876700 | -0.71813100 |
| H | 2.96728600 | 2.53190700 | -2.23800800 |
| H | 1.80989300 | 0.48304000 | 1.93291900 |
| H | 0.40818300 | 1.51826100 | 1.86404800 |
| H | 3.26736900 | 2.48991000 | 1.32568500 |
| H | 1.82692700 | 3.49030600 | 1.29126700 |
| H | 2.73901800 | 0.32815200 | -2.00211700 |
| H | 2.47725500 | -0.99688700 | -0.89321400 |
| H | 4.27992600 | 1.41857600 | -0.35993000 |
| H | 3.85678300 | 0.22432300 | 0.85885600 |
| H | 1.48225900 | 5.15326900 | -1.73544300 |
| H | 1.48808300 | 4.28428200 | -3.25462000 |
| H | 3.98514200 | 5.44284700 | -1.88123100 |
| H | 3.99337200 | 4.56549100 | -3.41089700 |
| H | 2.83074600 | 1.92962700 | 3.71993600 |
| H | 1.38409400 | 2.91596300 | 3.69353200 |
| H | 3.30183300 | 4.19314900 | 4.68115300 |
| H | 4.23365800 | 3.96268600 | 3.19951000 |
| H | 2.77676700 | 4.95657800 | 3.17847700 |
| H | 3.18299400 | 6.12956000 | -3.29577200 |
| H | -3.11826300 | -1.02195900 | -2.56177000 |
| H | -5.48463700 | -0.48877500 | -3.14301700 |
| H | -7.23362700 | -0.52709600 | -1.37401500 |
| H | -4.24774300 | -1.62422700 | 1.52795600 |
| H | -6.60745000 | -1.11106500 | 0.95865900 |
| H | 1.07011300 | -1.52839400 | 2.17447300 |
| H | 3.06962600 | -2.98586100 | 2.19421300 |
| H | 0.18342600 | -2.83109000 | -1.82129700 |
| H | 2.13972200 | -4.29757200 | -1.78959200 |
| H | -1.45691400 | -2.26121300 | 2.82871500 |
| H | -1.56967000 | -4.25252000 | 1.47353700 |
| H | 5.21930600 | -5.67632600 | -0.58724700 |
| H | 3.53325000 | -5.95776600 | -1.09194900 |
| H | 4.42393800 | -4.54369900 | -1.71290700 |
| H | 5.21279700 | -0.42540800 | -1.80028000 |
| H | 4.78342600 | -1.59112000 | -0.56278500 |
| H | 7.20323400 | -0.96524800 | -0.37479000 |
| H | 6.76375500 | 0.73359300 | -0.17873200 |
| H | 6.32827800 | -0.43916100 | 1.06533500 |
| C | -2.72077700 | 1.56134800 | -0.00710200 |
| O | -1.76049800 | 1.45419000 | -2.15895200 |
| C | -2.55682000 | 1.32789600 | 1.36056200 |
| N | -3.44006300 | 1.67883900 | 2.29975800 |
| C | -4.55821500 | 2.27826400 | 1.87109300 |
| C | -3.89389500 | 2.19741800 | -0.42391200 |
| C | -4.83173200 | 2.55770100 | 0.53169100 |
| H | -1.66355000 | 0.80154800 | 1.70391400 |
| H | -5.28034200 | 2.54602200 | 2.64214200 |
| H | -4.04899500 | 2.37255700 | -1.48601100 |
| H | -5.76593100 | 3.03788400 | 0.25434700 |

**Ni[P(n-Bu)$_3$](PyrCO)[PhOB(OH)$_2$][PhOMe], iso1a**

| | | | |
|---|---|---|---|
| Ni | 0.45916600 | 0.02253300 | -0.43359100 |
| O | 2.24592400 | -0.69100400 | -1.33503700 |
| B | 2.22146600 | -0.77140900 | -2.72185000 |
| C | -0.15968700 | -1.79925400 | -0.05615300 |
| C | 1.16060400 | 1.78552400 | -0.76564200 |

| | | | |
|---|---|---|---|
| P | -1.39849400 | 0.93791700 | 0.09012300 |
| C | -2.67085600 | 0.02619400 | 1.06485400 |
| C | -1.27434500 | 2.58380300 | 0.92707600 |
| C | -2.21004200 | 1.29140800 | -1.51843900 |
| C | -2.47037600 | 3.11566900 | 1.70860100 |
| C | -2.27651500 | 4.56728200 | 2.12711100 |
| C | -3.49531400 | 2.10994200 | -1.48576900 |
| C | -4.18414500 | 2.16676800 | -2.84335800 |
| C | -3.37784900 | -1.10867700 | 0.33136500 |
| C | -3.43167200 | 5.10212800 | 2.95413300 |
| C | -5.44437600 | 3.01262000 | -2.82909800 |
| C | 2.19904300 | 2.19388600 | 0.24783300 |
| O | 0.98797700 | 2.50347300 | -1.75274300 |
| C | 3.37169700 | -0.91090900 | -0.54967000 |
| C | 3.27912100 | -1.85585100 | 0.46566800 |
| C | 4.36848400 | -2.04021700 | 1.31240700 |
| C | 5.52907000 | -1.28828700 | 1.14392900 |
| C | 4.51865100 | -0.14583900 | -0.73183800 |
| C | 5.60000700 | -0.34382100 | 0.12211300 |
| C | -0.44359000 | -2.72465300 | -1.08080100 |
| C | -0.93460600 | -4.00245800 | -0.82861200 |
| C | -1.16163600 | -4.41475700 | 0.48841900 |
| C | -0.36677400 | -2.27017300 | 1.24874700 |
| C | -0.85416500 | -3.54904100 | 1.53962700 |
| O | -1.69197400 | -5.66920100 | 0.64236700 |
| O | 3.35983900 | -1.13840600 | -3.36697200 |
| O | 1.00252200 | -0.46853200 | -3.24637500 |
| C | -2.02711600 | -6.05446300 | 1.95767400 |
| C | -4.16832800 | -2.00024600 | 1.28025500 |
| C | -4.78934000 | -3.19416600 | 0.57799700 |
| H | -0.99219900 | 3.28531300 | 0.12966400 |
| H | -0.39941700 | 2.53767300 | 1.58727200 |
| H | -3.39297800 | 3.03175200 | 1.11871100 |
| H | -2.63230400 | 2.49922900 | 2.60305500 |
| H | -2.38635700 | 0.31261500 | -1.98556000 |
| H | -1.44296400 | 1.78574800 | -2.12904900 |
| H | -4.19717200 | 1.69544300 | -0.74573000 |
| H | -3.27359300 | 3.13246700 | -1.14808700 |
| H | -3.41021800 | 0.74841400 | 1.43216100 |
| H | -2.15844500 | -0.36898000 | 1.95083900 |
| H | -4.05037700 | -0.69352700 | -0.43438200 |
| H | -2.64816600 | -1.72629100 | -0.20326100 |
| H | -2.14283000 | 5.18270300 | 1.22679400 |
| H | -1.33769100 | 4.65799600 | 2.68985100 |
| H | -4.37538200 | 5.04567300 | 2.40082200 |
| H | -3.56053600 | 4.52435100 | 3.87572100 |
| H | -4.42211000 | 1.14309600 | -3.16400800 |
| H | -3.47745600 | 2.55545400 | -3.58864800 |
| H | -5.93040600 | 3.03701000 | -3.80842800 |
| H | -6.17314400 | 2.62560000 | -2.10882900 |
| H | -5.22421100 | 4.04698500 | -2.54425800 |
| H | -3.27881500 | 6.14679100 | 3.23879800 |
| H | 2.35453500 | -2.41382800 | 0.58313300 |
| H | 4.30457300 | -2.77487900 | 2.10989000 |
| H | 6.37417300 | -1.43338800 | 1.81029800 |
| H | 4.55264000 | 0.59617800 | -1.52380300 |
| H | 6.49895700 | 0.25218100 | -0.00985400 |
| H | -0.30595400 | -2.42860600 | -2.12206100 |
| H | -1.17043300 | -4.69493300 | -1.63419800 |
| H | -0.16107700 | -1.61090900 | 2.09648600 |
| H | -1.00619300 | -3.84978600 | 2.57269800 |
| H | 0.94927100 | -0.49443500 | -4.20330300 |
| H | 3.27398300 | -1.20637200 | -4.31948400 |
| H | -2.47764800 | -7.04466300 | 1.88363800 |
| H | -1.14388200 | -6.11038600 | 2.60788100 |
| H | -2.74899300 | -5.36084800 | 2.41125800 |
| H | -4.94359800 | -1.41085400 | 1.79018500 |
| H | -3.48682000 | -2.35320100 | 2.06742800 |
| H | -5.34440600 | -3.83319000 | 1.27153300 |
| H | -5.48458400 | -2.88051800 | -0.20876300 |
| H | -4.01568600 | -3.81041200 | 0.10510700 |
| C | 2.28917500 | 1.52010000 | 1.47083100 |
| N | 3.21758000 | 1.75203900 | 2.40151800 |
| C | 4.11839200 | 2.70230600 | 2.11866300 |
| C | 3.14205000 | 3.19252800 | -0.00917100 |
| C | 4.12129200 | 3.44955700 | 0.94035200 |
| H | 1.55350100 | 0.73885000 | 1.69538700 |
| H | 4.88136600 | 2.87587000 | 2.87680000 |
| H | 3.08784300 | 3.73345400 | -0.95156200 |
| H | 4.88014800 | 4.21019700 | 0.78063900 |

**Ni[P(n-Bu)$_3$](PyrCO)[PhOB(OH)$_2$][PhOMe], iso1b**

| | | | |
|---|---|---|---|
| Ni | -0.25456300 | -0.80161600 | -0.13188000 |
| O | -1.95642000 | -2.16037000 | -0.07251000 |
| B | -2.63937300 | -1.76795500 | -1.23086800 |
| C | -1.46177100 | 0.71850100 | -0.49781000 |
| C | 0.84491800 | -2.35572500 | 0.01438700 |
| P | 1.37773700 | 0.46704000 | 0.44611700 |
| C | 0.90520600 | 2.05872000 | 1.25554900 |
| C | 2.51443200 | -0.35169200 | 1.65089700 |
| C | 2.46288800 | 0.99038500 | -0.94480000 |
| C | 3.29771400 | 0.50487900 | 2.63841500 |
| C | 4.34629100 | -0.30683500 | 3.38800800 |
| C | 3.72257300 | 1.76825100 | -0.58073000 |
| C | 4.41367600 | 2.35676800 | -1.80366100 |
| C | 0.56627400 | 3.20848900 | 0.31163200 |
| C | 5.10968000 | 0.51698200 | 4.40911500 |
| C | 5.69265400 | 3.09762100 | -1.45862900 |
| O | 1.00260000 | -3.03774200 | 1.02597000 |
| C | 1.39104600 | -2.87369500 | -1.28882900 |
| C | -2.55872600 | -2.29909900 | 1.16579200 |
| C | -1.92524400 | -1.73074800 | 2.26712600 |
| C | -2.47858800 | -1.89501100 | 3.53314500 |
| C | -3.65378500 | -2.62435900 | 3.69854300 |
| C | -3.72658600 | -3.04120800 | 1.31610300 |
| C | -4.27065300 | -3.19593400 | 2.58798400 |
| C | -1.54348000 | 1.21777800 | -1.81478900 |
| C | -2.35437800 | 2.29064800 | -2.17292900 |
| C | -3.12575700 | 2.93008400 | -1.19680400 |
| C | -2.26633400 | 1.38054900 | 0.44144500 |
| C | -3.08921800 | 2.46484700 | 0.11905100 |
| O | -3.86713000 | 3.99974200 | -1.62197700 |
| O | -3.94111300 | -1.39515200 | -1.14392800 |
| O | -1.85697700 | -1.88623200 | -2.34276900 |
| C | -4.60934600 | 4.68630400 | -0.63616000 |
| C | -0.11152800 | 4.36617500 | 1.03198700 |
| C | -0.50484200 | 5.48501300 | 0.08440700 |
| H | 3.19971000 | -0.95125300 | 1.03436100 |
| H | 1.91056200 | -1.09169200 | 2.18855600 |
| H | 3.78912800 | 1.34554700 | 2.13031300 |
| H | 2.60715300 | 0.95819400 | 3.36231200 |
| H | 1.82370700 | 1.57901400 | -1.61741300 |
| H | 2.72734500 | 0.08404300 | -1.50389300 |

| | | | |
|---|---|---|---|
| H | 3.48318100 | 2.57893000 | 0.12331200 |
| H | 4.42136100 | 1.10767100 | -0.04836900 |
| H | 1.71283500 | 2.36222500 | 1.93264700 |
| H | 0.03624600 | 1.83030700 | 1.88590200 |
| H | 1.48202500 | 3.56730200 | -0.18150200 |
| H | -0.09472900 | 2.86267900 | -0.49004500 |
| H | 5.04462900 | -0.74474000 | 2.66148900 |
| H | 3.85853200 | -1.15882800 | 3.88024400 |
| H | 5.63016900 | 1.35538200 | 3.93351700 |
| H | 4.43568500 | 0.93881300 | 5.16240700 |
| H | 3.71446800 | 3.03213000 | -2.31572900 |
| H | 4.62695900 | 1.55155700 | -2.51946200 |
| H | 6.17252200 | 3.51615200 | -2.34761100 |
| H | 5.49808500 | 3.92543400 | -0.76819400 |
| H | 6.41703300 | 2.43412200 | -0.97426400 |
| H | 5.85963100 | -0.07985300 | 4.93554800 |
| H | -0.99803100 | -1.17737500 | 2.11285300 |
| H | -1.98442700 | -1.45067700 | 4.39251400 |
| H | -4.08393400 | -2.75081600 | 4.68737800 |
| H | -4.20148600 | -3.48354100 | 0.44689900 |
| H | -5.18390500 | -3.77202300 | 2.70742400 |
| H | -0.93233700 | 0.76317200 | -2.59924300 |
| H | -2.39098100 | 2.66731100 | -3.19298500 |
| H | -2.25643500 | 1.05577800 | 1.48446900 |
| H | -3.68262000 | 2.94314400 | 0.89336500 |
| H | -2.21645500 | -1.47216900 | -3.12985200 |
| H | -4.31745300 | -1.09483000 | -1.97378500 |
| H | -5.11352200 | 5.50699800 | -1.14666200 |
| H | -5.36016700 | 4.03832700 | -0.16519100 |
| H | -3.96048200 | 5.09428200 | 0.15116300 |
| H | 0.54598300 | 4.75053500 | 1.82435200 |
| H | -1.00721500 | 3.97971200 | 1.53856100 |
| H | -1.00947500 | 6.30455900 | 0.60456100 |
| H | 0.37015600 | 5.90590500 | -0.42325000 |
| H | -1.18511900 | 5.11433500 | -0.69142800 |
| C | 1.32775400 | -2.10385000 | -2.45421400 |
| N | 1.78774100 | -2.49329200 | -3.64647400 |
| C | 2.34304300 | -3.71140700 | -3.70300800 |
| C | 1.97665400 | -4.13934100 | -1.38059400 |
| C | 2.46071600 | -4.56721000 | -2.60814700 |
| H | 0.86732000 | -1.11208900 | -2.40567000 |
| H | 2.71314300 | -4.02087900 | -4.67998100 |
| H | 2.03443200 | -4.75413200 | -0.48539400 |
| H | 2.92177800 | -5.54341400 | -2.72686700 |

**Ni[P(n-Bu)$_3$](PyrCO)[PhOMe]**

| | | | |
|---|---|---|---|
| Ni | 1.17131900 | -0.47180400 | -0.00722600 |
| P | -1.07237500 | -0.76154900 | 0.02052200 |
| C | -1.81966500 | -0.44843100 | -1.63964800 |
| C | -1.69855200 | -2.41788800 | 0.54698800 |
| C | -1.85962100 | 0.45801500 | 1.16379100 |
| C | -3.22522700 | 0.13441900 | -1.72367600 |
| C | -3.62523100 | 0.45808800 | -3.15736800 |
| C | -5.01929800 | 1.05019800 | -3.25956200 |
| C | -3.10202100 | -2.82919600 | 0.11756800 |
| C | -3.25652100 | 0.21443700 | 1.71956800 |
| C | -3.70699900 | 1.36550300 | 2.61106800 |
| C | -5.08231200 | 1.14548400 | 3.21394100 |
| C | -3.48500300 | -4.21113100 | 0.63241100 |
| C | -4.87491700 | -4.63436500 | 0.19320000 |
| C | 3.06246700 | -0.39012700 | 0.10053100 |
| C | 1.18238100 | 0.97179700 | -1.11057100 |
| C | 0.61948000 | 2.17002600 | -0.42334700 |
| O | 1.39380200 | 0.93577600 | -2.31026300 |
| C | 0.83093600 | 2.40528500 | 0.93996600 |
| N | 0.22673600 | 3.37332700 | 1.63304300 |
| C | -0.64143200 | 4.14460000 | 0.96285300 |
| C | -0.25331300 | 3.01780500 | -1.11097400 |
| C | -0.90654700 | 4.01723300 | -0.39965400 |
| H | -1.76322400 | -1.39811100 | -2.18827300 |
| H | -1.12150400 | 0.21773300 | -2.16175000 |
| H | -3.95781100 | -0.55790200 | -1.28757400 |
| H | -3.28501600 | 1.05324300 | -1.12183200 |
| H | -3.55838700 | -0.45363600 | -3.76594500 |
| H | -2.88915500 | 1.15363600 | -3.58379200 |
| H | -5.77369400 | 0.35835400 | -2.87001500 |
| H | -5.09897900 | 1.97676600 | -2.68086500 |
| H | -1.60653300 | -2.44659500 | 1.64119100 |
| H | -0.97461200 | -3.15352600 | 0.17546100 |
| H | -3.84100900 | -2.09702500 | 0.46611000 |
| H | -3.16954400 | -2.82123000 | -0.97938400 |
| H | -1.82753200 | 1.42078400 | 0.63531800 |
| H | -1.15611700 | 0.57625200 | 1.99809200 |
| H | -3.98240100 | 0.08270800 | 0.90711000 |
| H | -3.27446400 | -0.72287800 | 2.29409300 |
| H | -3.69718700 | 2.29383000 | 2.02218400 |
| H | -2.96536000 | 1.51550000 | 3.40744400 |
| H | -5.84258200 | 1.02339200 | 2.43509900 |
| H | -5.10396900 | 0.24248500 | 3.83325600 |
| H | -5.28828500 | 1.28305100 | -4.29330000 |
| H | -5.38875900 | 1.98434300 | 3.84455000 |
| H | -3.42063300 | -4.21621800 | 1.72902000 |
| H | -2.74247900 | -4.94349700 | 0.28811300 |
| H | -5.13743300 | -5.62563900 | 0.57258000 |
| H | -5.63452600 | -3.93174700 | 0.55216700 |
| H | -4.95297400 | -4.66733200 | -0.89869400 |
| H | 1.52593300 | 1.76200100 | 1.48380900 |
| H | -1.15204000 | 4.90716200 | 1.54959400 |
| H | -0.42273500 | 2.86295300 | -2.17430700 |
| H | -1.61243800 | 4.68428200 | -0.88507100 |
| C | 3.66586200 | -0.43810600 | 1.37386600 |
| C | 5.03249900 | -0.64131600 | 1.52977500 |
| C | 5.85050000 | -0.77992200 | 0.40349700 |
| C | 3.91414300 | -0.49839900 | -1.00494400 |
| C | 5.28823900 | -0.70346300 | -0.87387400 |
| H | 3.05912500 | -0.33131600 | 2.27388000 |
| H | 5.49111400 | -0.69206900 | 2.51425700 |
| O | 7.17913800 | -0.97472100 | 0.65338900 |
| H | 3.49381000 | -0.41713300 | -2.00718200 |
| H | 5.90787400 | -0.79387000 | -1.76100600 |
| C | 8.02525100 | -1.11570500 | -0.46913100 |
| H | 9.03089200 | -1.26813800 | -0.07698000 |
| H | 7.74330400 | -1.97924200 | -1.08547000 |
| H | 8.01676200 | -0.21839400 | -1.10134700 |

**TS(decarb.), Ni[P(n-Bu)$_3$](PyrCO)[PhOMe]**

| | | | |
|---|---|---|---|
| Ni | 0.62804400 | -1.37460200 | 0.01360600 |
| P | -1.30664100 | -0.42834700 | -0.13666500 |
| C | -2.65799900 | -1.55302100 | -0.70035200 |
| C | -1.82926600 | 0.15235900 | 1.52605100 |
| C | -1.40317800 | 1.07756200 | -1.19926900 |
| C | -3.90115300 | -0.95004900 | -1.34563000 |
| C | -5.00943900 | -1.98055300 | -1.51970300 |
| C | -6.24037700 | -1.41172600 | -2.20194600 |

| | | | |
|---|---|---|---|
| C | -3.25241200 | 0.68397700 | 1.65501400 |
| C | -0.97573500 | 2.37870000 | -0.52229200 |
| C | -0.73631700 | 3.49917300 | -1.52469700 |
| C | -0.28639900 | 4.78453400 | -0.85432200 |
| C | -3.50225200 | 1.34921500 | 3.00199800 |
| C | -4.92603200 | 1.85230100 | 3.15546800 |
| C | 1.62028700 | 0.30233500 | 0.07947500 |
| C | 1.98317200 | -2.95417800 | 0.15642500 |
| C | 0.34499700 | -3.11326200 | -0.04785000 |
| O | -0.17202400 | -4.18707800 | -0.16995400 |
| C | 2.55477500 | -3.07297200 | 1.42823500 |
| C | 3.92999700 | -3.23338200 | 1.52962600 |
| C | 4.68261100 | -3.26134500 | 0.35665200 |
| C | 2.83811800 | -3.00199400 | -0.95329400 |
| N | 4.16213200 | -3.15057300 | -0.87335700 |
| H | -2.93100200 | -2.13459100 | 0.19172400 |
| H | -2.20216700 | -2.28050700 | -1.38087400 |
| H | -4.28247700 | -0.10701500 | -0.75445300 |
| H | -3.64037700 | -0.53129600 | -2.32689200 |
| H | -5.27915100 | -2.38353100 | -0.53371500 |
| H | -4.62185200 | -2.83361500 | -2.09242200 |
| H | -6.66043000 | -0.57716500 | -1.63023200 |
| H | -6.00086500 | -1.03280300 | -3.20131800 |
| H | -1.09747300 | 0.92019700 | 1.81080200 |
| H | -1.66973400 | -0.68192900 | 2.21986500 |
| H | -3.46436600 | 1.40496900 | 0.85150100 |
| H | -3.96846800 | -0.13810300 | 1.51413900 |
| H | -2.42277500 | 1.17659100 | -1.59124100 |
| H | -0.75406700 | 0.88520100 | -2.06295200 |
| H | -1.74349300 | 2.68971200 | 0.20120200 |
| H | -0.05656800 | 2.22810200 | 0.05544200 |
| H | -1.64648000 | 3.67526300 | -2.11515200 |
| H | 0.03051900 | 3.16639100 | -2.23852800 |
| H | -1.04358800 | 5.15798700 | -0.15607000 |
| H | 0.63407600 | 4.62226400 | -0.28111500 |
| H | -7.02675600 | -2.16291500 | -2.31457600 |
| H | -0.08991000 | 5.57786300 | -1.58164800 |
| H | -2.79441500 | 2.18056000 | 3.12349100 |
| H | -3.26568600 | 0.63761800 | 3.80440900 |
| H | -5.08570700 | 2.33035900 | 4.12559200 |
| H | -5.17212300 | 2.58688300 | 2.38121600 |
| H | -5.64818300 | 1.03355200 | 3.06805800 |
| H | 1.92822200 | -3.03638400 | 2.31670200 |
| H | 4.41672300 | -3.32712000 | 2.49538100 |
| H | 5.76415200 | -3.37719400 | 0.40530500 |
| H | 2.42398300 | -2.91098300 | -1.95808100 |
| C | 1.91313000 | 0.98982100 | 1.27423400 |
| C | 2.52034600 | 2.24201300 | 1.29465000 |
| C | 2.86896400 | 2.86614400 | 0.09276000 |
| C | 2.01582600 | 0.95286500 | -1.09940200 |
| C | 2.63148500 | 2.20855500 | -1.11554200 |
| H | 1.63628300 | 0.54698300 | 2.23405700 |
| H | 2.72309500 | 2.76429600 | 2.22698000 |
| O | 3.42254300 | 4.11484300 | 0.20331700 |
| H | 1.81889300 | 0.48367500 | -2.06671000 |
| H | 2.90311400 | 2.66634200 | -2.06269200 |
| C | 3.75400600 | 4.76885600 | -1.00300800 |
| H | 4.15977600 | 5.74156600 | -0.72378700 |
| H | 4.50923700 | 4.21383000 | -1.57495000 |
| H | 2.87238600 | 4.91534300 | -1.64251600 |

**TS(B-C), Ni[P(n-Bu)$_3$]$_2$(PyrCO)[PhOB(OH)$_2$-PhOMe], Iso-1_up**

| | | | |
|---|---|---|---|
| Ni | 0.03529900 | -0.30574000 | -0.60445800 |
| O | -2.47597100 | -1.06978700 | -0.94030700 |
| B | -1.91682300 | -2.32953300 | -0.49853800 |
| C | 0.19537000 | -2.32789200 | -0.26855100 |
| P | -0.89519000 | 0.75999300 | 1.37480100 |
| C | 0.08623200 | -0.38282600 | -2.46586500 |
| P | 1.95956000 | 0.81630700 | -0.76211400 |
| C | -0.59622500 | 2.60172200 | 1.22220300 |
| C | 3.24676700 | -0.30606500 | -1.45493900 |
| C | 2.14526400 | 2.40288200 | -1.69813300 |
| C | 2.56360100 | 1.20116800 | 0.93629500 |
| C | -2.72959900 | 0.71168700 | 1.49090200 |
| C | -0.39966200 | 0.33654900 | 3.11484200 |
| C | -0.16192400 | 3.38059800 | 2.45683200 |
| C | 0.27541100 | 4.79931200 | 2.11894500 |
| C | 0.66159400 | 5.60545500 | 3.34581000 |
| C | -3.39197900 | 1.61656700 | 2.51908100 |
| C | -1.15528500 | -0.88192100 | 3.65729900 |
| C | 3.51983100 | 2.77971300 | -2.24777400 |
| C | 3.59385200 | 4.25351400 | -2.62452100 |
| C | 3.84446000 | 2.00966000 | 1.09198200 |
| C | 4.18857400 | 2.24141100 | 2.55814000 |
| C | 3.82972800 | -1.29176900 | -0.45073700 |
| C | 4.93268900 | 4.64352800 | -3.22358400 |
| C | 5.50402000 | 2.97402000 | 2.74861600 |
| O | 0.70920800 | -1.04669800 | -3.27550200 |
| C | -3.81018300 | -0.97088200 | -1.16421500 |
| C | -4.72158600 | -2.01141300 | -0.93238700 |
| C | -6.07814000 | -1.80977700 | -1.17026000 |
| C | -6.55722000 | -0.58787600 | -1.63787200 |
| C | -4.29441800 | 0.25935000 | -1.62692800 |
| C | -5.65090400 | 0.44626600 | -1.86400300 |
| C | 0.59157700 | -2.54905800 | 1.07345400 |
| C | 1.49793500 | -3.52877400 | 1.45881100 |
| C | 2.06276100 | -4.36806800 | 0.49247200 |
| C | 0.81887800 | -3.17917600 | -1.20120900 |
| C | 1.72056900 | -4.18421800 | -0.85031600 |
| O | 2.92006700 | -5.32652500 | 0.95255200 |
| O | -2.01585600 | -3.30238100 | -1.50739000 |
| O | -2.27226100 | -2.59840000 | 0.84267300 |
| H | -1.51861500 | 3.03350100 | 0.81038100 |
| H | 0.15648000 | 2.74143100 | 0.43523100 |
| H | -0.97405400 | 3.40365700 | 3.19736800 |
| H | 0.67239600 | 2.86638500 | 2.95730500 |
| H | -0.52690600 | 5.30711400 | 1.56728100 |
| H | 1.12626100 | 4.74640900 | 1.42294700 |
| H | -0.18193400 | 5.70358700 | 4.03710400 |
| H | 1.47709900 | 5.12206000 | 3.89550100 |
| H | -3.00513700 | -0.33829000 | 1.65255800 |
| H | -3.10697600 | 0.94267000 | 0.48817200 |
| H | -3.00171800 | 1.41314700 | 3.52762600 |
| H | -3.14810600 | 2.66979400 | 2.31486600 |
| H | -0.54116600 | 1.19557800 | 3.77977700 |
| H | 0.68007900 | 0.13421300 | 3.09949900 |
| H | -2.09548200 | -0.55309200 | 4.12085200 |
| H | -1.46533400 | -1.53667300 | 2.83425300 |
| H | 1.78041300 | 3.18939800 | -1.02016500 |
| H | 1.42274900 | 2.37902600 | -2.52229100 |
| H | 4.31495900 | 2.55071000 | -1.52634800 |
| H | 3.73737100 | 2.16543900 | -3.13130600 |

| | | | |
|---|---|---|---|
| H | 2.65467000 | 0.22638100 | 1.43540900 |
| H | 1.75009800 | 1.71627400 | 1.45780600 |
| H | 4.68196900 | 1.50444200 | 0.58873100 |
| H | 3.73738400 | 2.98298400 | 0.59213600 |
| H | 4.05042600 | 0.28888300 | -1.90550700 |
| H | 2.75732900 | -0.85580600 | -2.26888800 |
| H | 4.37285700 | -0.75140300 | 0.33830000 |
| H | 3.02091800 | -1.83839800 | 0.05176000 |
| H | 3.39212500 | 4.85871500 | -1.72897000 |
| H | 2.78379900 | 4.48777500 | -3.32809600 |
| H | 5.75211200 | 4.45100400 | -2.52268900 |
| H | 5.14231400 | 4.07126000 | -4.13345500 |
| H | 4.21502500 | 1.27404700 | 3.07800500 |
| H | 3.37163400 | 2.80894100 | 3.02754300 |
| H | 5.72231100 | 3.14970000 | 3.80544200 |
| H | 6.33915400 | 2.40359000 | 2.32896300 |
| H | 5.49103000 | 3.94821100 | 2.24838700 |
| H | 0.99324600 | 6.61392600 | 3.08417700 |
| H | 4.96619600 | 5.70406800 | -3.48662100 |
| H | -4.36147000 | -2.96693100 | -0.56925600 |
| H | -6.77092500 | -2.62746400 | -0.98534400 |
| H | -7.61814700 | -0.44434200 | -1.82069000 |
| H | -3.59427100 | 1.06451300 | -1.78563200 |
| H | -5.99732600 | 1.41226600 | -2.22514300 |
| H | 0.14911400 | -1.94110500 | 1.85560200 |
| H | 1.76482600 | -3.67593700 | 2.50351200 |
| H | 0.56843600 | -3.07755100 | -2.25350300 |
| H | 2.14515200 | -4.81722500 | -1.62441300 |
| H | -1.89015700 | -3.42543000 | 1.14686900 |
| H | -1.59126200 | -4.12387800 | -1.25156200 |
| C | 3.47107700 | -6.20491800 | -0.00994700 |
| H | 4.11785100 | -6.89045300 | 0.53757500 |
| H | 2.69105100 | -6.77646000 | -0.52844500 |
| H | 4.06779400 | -5.66592300 | -0.75778500 |
| C | -0.33725300 | -1.69017500 | 4.65533300 |
| C | -1.02027400 | -2.98519900 | 5.05532600 |
| H | -0.12051700 | -1.08031600 | 5.54282400 |
| H | 0.64145600 | -1.91488600 | 4.20599900 |
| H | -0.41575200 | -3.56952900 | 5.75462800 |
| H | -1.98912800 | -2.79797500 | 5.53034700 |
| H | -1.20655000 | -3.61221600 | 4.17496800 |
| C | -4.90632700 | 1.44331700 | 2.51902800 |
| C | -5.61284200 | 2.38191100 | 3.47988000 |
| H | -5.14418900 | 0.39990800 | 2.76769600 |
| H | -5.28178500 | 1.59209500 | 1.49704400 |
| H | -6.69758000 | 2.24499400 | 3.46041400 |
| H | -5.27917000 | 2.22104600 | 4.51104400 |
| H | -5.40846700 | 3.42950200 | 3.23270900 |
| C | 4.77549700 | -2.29219000 | -1.10125100 |
| C | 5.49010900 | -3.15722600 | -0.07828800 |
| H | 5.51173900 | -1.75622300 | -1.71688500 |
| H | 4.20058000 | -2.92136000 | -1.79532100 |
| H | 6.13455800 | -3.90436800 | -0.55164400 |
| H | 6.12145300 | -2.54880300 | 0.57871100 |
| H | 4.77561200 | -3.68816500 | 0.56021000 |
| C | -0.95299900 | 0.59074700 | -2.95062200 |
| C | -1.18121000 | 1.83322000 | -2.34882700 |
| C | -1.69872100 | 0.27718800 | -4.09161200 |
| N | -2.04859300 | 2.74333600 | -2.80312900 |
| H | -0.60912900 | 2.10817900 | -1.46182900 |
| C | -2.61546300 | 1.20313000 | -4.56752000 |
| H | -1.54891300 | -0.68145300 | -4.58060300 |
| C | -2.74938100 | 2.41798100 | -3.89714200 |
| H | -3.21828400 | 0.99248000 | -5.44540100 |
| H | -3.45992800 | 3.16436700 | -4.24933200 |

**TS(B-C), Ni[P(n-Bu)$_3$]$_2$(PyrCO)[PhOB(OH)$_2$-PhOMe], Iso-2_up**

| | | | |
|---|---|---|---|
| Ni | 0.04365700 | -0.28828400 | -0.59523800 |
| O | -2.46671000 | -1.12606500 | -1.04805300 |
| B | -1.98024100 | -2.37672300 | -0.48868700 |
| C | 0.15126700 | -2.32184800 | -0.34563100 |
| P | -0.89533800 | 0.72562500 | 1.39330100 |
| C | 0.11593700 | -0.27756300 | -2.45900600 |
| P | 1.97062100 | 0.83279200 | -0.69867600 |
| C | -0.58619500 | 2.57042600 | 1.27390500 |
| C | 3.26247700 | -0.28281600 | -1.39256800 |
| C | 2.16683900 | 2.43348700 | -1.61008000 |
| C | 2.57355500 | 1.18850200 | 1.00584700 |
| C | -2.73219600 | 0.69071300 | 1.45956200 |
| C | -0.42303500 | 0.28767700 | 3.13419000 |
| C | -0.18360500 | 3.33698200 | 2.52745200 |
| C | 0.28233800 | 4.75225600 | 2.21282600 |
| C | 0.68485400 | 5.53048500 | 3.45233300 |
| C | -3.41211400 | 1.55335100 | 2.51253400 |
| C | -1.16118400 | -0.94849100 | 3.66282600 |
| C | 3.53797500 | 2.80392400 | -2.16956500 |
| C | 3.59391500 | 4.26024700 | -2.61167400 |
| C | 3.87732000 | 1.96106000 | 1.15875400 |
| C | 4.28730100 | 2.09379200 | 2.61974300 |
| C | 3.82193600 | -1.29352800 | -0.39942300 |
| C | 4.93014600 | 4.64006300 | -3.22330600 |
| C | 5.57359000 | 2.87762400 | 2.80496300 |
| O | 0.74586700 | -0.90409900 | -3.29557100 |
| C | -3.80842400 | -0.97189200 | -1.22108700 |
| C | -4.75580600 | -1.97322900 | -0.96167600 |
| C | -6.10942100 | -1.71340500 | -1.16102000 |
| C | -6.54957300 | -0.47300200 | -1.61738100 |
| C | -4.25346000 | 0.27575300 | -1.67516900 |
| C | -5.60656200 | 0.52064700 | -1.87374000 |
| C | 0.52714400 | -2.61191700 | 0.98867300 |
| C | 1.42582300 | -3.61316500 | 1.33565400 |
| C | 2.00524300 | -4.39936200 | 0.33473700 |
| C | 0.79545900 | -3.11818700 | -1.31183000 |
| C | 1.69082200 | -4.13991200 | -1.00267600 |
| O | 2.85253800 | -5.38463400 | 0.75549700 |
| O | -2.10166300 | -3.52050800 | -1.29018500 |
| O | -2.33441900 | -2.54664500 | 0.85585700 |
| H | -1.49424000 | 3.01009200 | 0.83874900 |
| H | 0.19000100 | 2.71546300 | 0.51188600 |
| H | -1.01938500 | 3.36649600 | 3.24071100 |
| H | 0.62799900 | 2.81207600 | 3.05305100 |
| H | -0.50891000 | 5.28623400 | 1.67010500 |
| H | 1.13260400 | 4.69431500 | 1.51570000 |
| H | -0.15384700 | 5.62662800 | 4.14988700 |
| H | 1.49708900 | 5.02739800 | 3.98863000 |
| H | -3.03188000 | -0.35945400 | 1.55832400 |
| H | -3.06716200 | 0.99091400 | 0.45952500 |
| H | -3.06611400 | 1.27919000 | 3.52045600 |
| H | -3.13411200 | 2.60954400 | 2.38027800 |
| H | -0.58708200 | 1.14212000 | 3.79948300 |
| H | 0.66043500 | 0.10661000 | 3.13166200 |
| H | -2.07099800 | -0.63310400 | 4.19117900 |
| H | -1.51980800 | -1.56892000 | 2.83253200 |

| | | | |
|---|---|---|---|
| H | 1.81913000 | 3.21271800 | -0.91507400 |
| H | 1.43578800 | 2.43200800 | -2.42717400 |
| H | 4.32973600 | 2.61909900 | -1.43213400 |
| H | 3.77005800 | 2.15415800 | -3.02352300 |
| H | 2.63961000 | 0.20783800 | 1.49743500 |
| H | 1.77611000 | 1.72165900 | 1.53326400 |
| H | 4.68315800 | 1.47164000 | 0.59225800 |
| H | 3.76694500 | 2.96434700 | 0.72142800 |
| H | 4.07800200 | 0.31885500 | -1.81209000 |
| H | 2.78676200 | -0.81114000 | -2.22867300 |
| H | 4.36667300 | -0.77285900 | 0.40203200 |
| H | 3.00212900 | -1.83708100 | 0.08884500 |
| H | 3.38114200 | 4.90265000 | -1.74542100 |
| H | 2.78394600 | 4.45168300 | -3.32819700 |
| H | 5.74980400 | 4.48506800 | -2.51346400 |
| H | 5.14811100 | 4.03361900 | -4.10874700 |
| H | 4.39096400 | 1.08991200 | 3.05375100 |
| H | 3.46957600 | 2.57518700 | 3.17578300 |
| H | 5.85198500 | 2.95881100 | 3.85904100 |
| H | 6.40713200 | 2.39976600 | 2.27943700 |
| H | 5.47892200 | 3.89426700 | 2.40879000 |
| H | 1.02695100 | 6.53959500 | 3.20717400 |
| H | 4.95301600 | 5.68943600 | -3.52894700 |
| H | -4.42971300 | -2.94466100 | -0.60948500 |
| H | -6.83039100 | -2.50092700 | -0.95462600 |
| H | -7.60831100 | -0.28436400 | -1.76925700 |
| H | -3.51699300 | 1.05078500 | -1.85098900 |
| H | -5.92152000 | 1.50038700 | -2.22686700 |
| H | 0.06881700 | -2.04706300 | 1.79482400 |
| H | 1.67539100 | -3.81822900 | 2.37486900 |
| H | 0.58271300 | -2.95353600 | -2.36572400 |
| H | 2.13256800 | -4.72788100 | -1.80205500 |
| H | -2.09359100 | -3.43318400 | 1.13850700 |
| C | 3.43730600 | -6.19355600 | -0.24697900 |
| H | 4.07284100 | -6.91128000 | 0.27197600 |
| H | 2.67729300 | -6.73297100 | -0.82575500 |
| H | 4.05226600 | -5.60154000 | -0.93829800 |
| C | -0.29916500 | -1.80173000 | 4.58379800 |
| C | -0.97491800 | -3.10222800 | 4.97766900 |
| H | -0.02336900 | -1.22529100 | 5.47727400 |
| H | 0.64878900 | -2.02176100 | 4.07101800 |
| H | -0.34859600 | -3.70657300 | 5.63964100 |
| H | -1.92417800 | -2.92116100 | 5.49326200 |
| H | -1.19781400 | -3.70704600 | 4.09042500 |
| C | -4.92947100 | 1.42379400 | 2.44489900 |
| C | -5.64526900 | 2.27563200 | 3.47711700 |
| H | -5.20274000 | 0.36711900 | 2.57163700 |
| H | -5.26511400 | 1.69196900 | 1.43357400 |
| H | -6.73159300 | 2.16871200 | 3.41209200 |
| H | -5.34745100 | 2.00048200 | 4.49494500 |
| H | -5.41042000 | 3.33780500 | 3.34676600 |
| C | 4.76161300 | -2.29332300 | -1.05954800 |
| C | 5.44945100 | -3.19360700 | -0.04870700 |
| H | 5.51403000 | -1.75326100 | -1.65177400 |
| H | 4.18769800 | -2.89672000 | -1.77695800 |
| H | 6.09118700 | -3.93644000 | -0.53266100 |
| H | 6.07934100 | -2.61128500 | 0.63293000 |
| H | 4.71865700 | -3.73309600 | 0.56370300 |
| C | -0.91225500 | 0.71974500 | -2.92205100 |
| C | -1.16448800 | 1.93072100 | -2.26852900 |
| C | -1.62342100 | 0.45995000 | -4.09817800 |
| N | -2.02995900 | 2.85449600 | -2.69896200 |
| H | -0.61418700 | 2.16850200 | -1.35769800 |
| C | -2.53743900 | 1.39946600 | -4.55165300 |
| H | -1.44647100 | -0.46888200 | -4.63391200 |
| C | -2.70189500 | 2.57718000 | -3.82387000 |
| H | -3.11410800 | 1.22922000 | -5.45566300 |
| H | -3.41159000 | 3.33364000 | -4.15564300 |
| H | -1.91380100 | -3.30915800 | -2.20772600 |

**Ni[P(n-Bu)$_3$]$_2$(PyrCO)[PhOB(OH)$_2$](PhOMe)**

| | | | |
|---|---|---|---|
| Ni | 0.57958700 | -0.96023400 | -0.02835200 |
| O | -1.23583200 | 2.17547100 | 3.57364500 |
| B | -0.59409600 | 0.97305500 | 3.34357100 |
| C | 2.51706800 | -1.04739400 | 0.31595900 |
| P | 0.74051100 | 1.08549000 | -1.07224000 |
| C | 0.63963500 | -2.31158600 | 1.27235600 |
| P | -1.62714700 | -1.26571700 | -0.33506300 |
| C | -0.68766700 | 2.25670400 | -0.85286800 |
| C | -2.22306600 | -3.01347500 | -0.37917800 |
| C | -2.46260100 | -0.55244200 | 1.14742700 |
| C | -2.38055100 | -0.51838300 | -1.85642900 |
| C | 2.15676300 | 2.09196000 | -0.46952800 |
| C | 1.02382400 | 1.00936700 | -2.89745200 |
| C | -1.12095900 | 3.07286100 | -2.06546500 |
| C | -2.36928300 | 3.90173100 | -1.79455800 |
| C | -2.90175100 | 4.59455100 | -3.03539800 |
| C | 2.24995300 | 3.53033600 | -0.95585200 |
| C | 2.05477200 | -0.05644900 | -3.23754500 |
| C | -3.89558800 | -0.90694800 | 1.52450600 |
| C | -4.30898800 | -0.20881400 | 2.81558000 |
| C | -3.74964600 | 0.14241500 | -1.75282900 |
| C | -4.14423300 | 0.84820000 | -3.04406500 |
| C | -3.61917300 | -3.29620300 | -0.92303300 |
| C | -5.72510600 | -0.54946800 | 3.24157300 |
| C | -5.45306300 | 1.60789100 | -2.92984500 |
| O | 0.42895600 | -2.01581000 | 2.46658100 |
| C | -0.98839000 | 3.28760600 | 2.81878700 |
| C | 0.30827800 | 3.75860400 | 2.59508000 |
| C | 0.49787600 | 4.92391500 | 1.85859500 |
| C | -0.59110200 | 5.62636500 | 1.34441700 |
| C | -2.08414600 | 3.98963800 | 2.31511600 |
| C | -1.88117700 | 5.15663000 | 1.58458600 |
| C | 3.36098600 | -1.68880000 | -0.61019900 |
| C | 4.74681700 | -1.65612500 | -0.50539300 |
| C | 5.35262100 | -0.99568200 | 0.56799600 |
| C | 3.15758200 | -0.42994200 | 1.39702400 |
| C | 4.54893600 | -0.38640800 | 1.53177300 |
| O | 6.72023100 | -1.01254900 | 0.58926300 |
| O | -0.90648200 | -0.03768900 | 4.20554600 |
| O | 0.25650300 | 0.85338000 | 2.28021900 |
| H | -0.42529700 | 2.91739200 | -0.01490100 |
| H | -1.54598500 | 1.67543300 | -0.49366600 |
| H | -0.30362200 | 3.72803200 | -2.39931500 |
| H | -1.32511500 | 2.40456600 | -2.91556700 |
| H | -2.14943900 | 4.63638300 | -1.00993200 |
| H | -3.14454200 | 3.24380300 | -1.37046100 |
| H | -2.16042900 | 5.28118400 | -3.45797300 |
| H | -3.15369600 | 3.86852900 | -3.81763400 |
| H | 3.07224800 | 1.53406800 | -0.70646900 |
| H | 2.07728700 | 2.06515400 | 0.62602700 |
| H | 2.33656800 | 3.56459000 | -2.05191600 |
| H | 1.32438000 | 4.07111200 | -0.70860700 |
| H | 1.32548900 | 1.99346900 | -3.28154700 |

| | | | |
|---|---|---|---|
| H | 0.07211200 | 0.76813600 | -3.38819100 |
| H | 2.97639600 | 0.11526300 | -2.66324400 |
| H | 1.67895300 | -1.02852600 | -2.88270700 |
| H | -2.33960300 | 0.53864300 | 1.09204700 |
| H | -1.80669500 | -0.86514700 | 1.96949900 |
| H | -4.59749100 | -0.64023100 | 0.72404800 |
| H | -3.98662700 | -1.99375900 | 1.66018900 |
| H | -2.39009300 | -1.30924100 | -2.61801100 |
| H | -1.66358900 | 0.22156800 | -2.22853900 |
| H | -4.51666600 | -0.59785100 | -1.48857800 |
| H | -3.74529500 | 0.87602700 | -0.93124300 |
| H | -2.12476900 | -3.38981300 | 0.64959500 |
| H | -1.48948800 | -3.58486600 | -0.96196800 |
| H | -4.37310700 | -2.68582700 | -0.41043600 |
| H | -3.67193600 | -3.00491700 | -1.98133800 |
| H | -4.20524300 | 0.87759900 | 2.68351700 |
| H | -3.59702100 | -0.47321100 | 3.60829400 |
| H | -6.45243000 | -0.26234300 | 2.47413800 |
| H | -5.84030000 | -1.62560600 | 3.41103100 |
| H | -4.20234400 | 0.11131600 | -3.85633300 |
| H | -3.33786200 | 1.53995900 | -3.32896900 |
| H | -5.71389300 | 2.11052900 | -3.86556400 |
| H | -6.28138300 | 0.94038600 | -2.66986100 |
| H | -5.39605700 | 2.37551300 | -2.14959000 |
| H | -3.80499800 | 5.17390400 | -2.82317600 |
| H | -6.00454600 | -0.03935000 | 4.16744400 |
| H | 1.15456000 | 3.20601200 | 2.99158300 |
| H | 1.50885200 | 5.28745900 | 1.68892300 |
| H | -0.43470500 | 6.53482100 | 0.76905400 |
| H | -3.08310200 | 3.60882700 | 2.50783200 |
| H | -2.73940800 | 5.69895900 | 1.19610500 |
| H | 2.93699600 | -2.21837900 | -1.46280700 |
| H | 5.37952800 | -2.14178200 | -1.24475600 |
| H | 2.57244200 | 0.06690500 | 2.17018100 |
| H | 4.98793200 | 0.12027500 | 2.38610700 |
| H | 0.53508400 | -0.07767400 | 2.19677900 |
| H | -0.47982800 | -0.85873800 | 3.92345300 |
| C | 7.34552900 | -0.35809400 | 1.67254100 |
| H | 8.41923400 | -0.47423800 | 1.52366300 |
| H | 7.09933500 | 0.71166900 | 1.70035100 |
| H | 7.06516000 | -0.80402400 | 2.63590200 |
| C | 2.39911500 | -0.14939800 | -4.71515900 |
| C | 3.41056600 | -1.24833600 | -4.99223800 |
| H | 2.79022000 | 0.81772900 | -5.05932900 |
| H | 1.48323300 | -0.32433600 | -5.29657600 |
| H | 3.68224400 | -1.30260800 | -6.04995100 |
| H | 4.33139200 | -1.08847800 | -4.41956700 |
| H | 3.01743400 | -2.22842100 | -4.69996500 |
| C | 3.42621000 | 4.27455800 | -0.33665600 |
| C | 3.47538800 | 5.73636300 | -0.74282500 |
| H | 4.36251900 | 3.77207500 | -0.61386200 |
| H | 3.36161000 | 4.19004200 | 0.75793700 |
| H | 4.31711500 | 6.26226300 | -0.28380800 |
| H | 3.57127700 | 5.84365600 | -1.82859900 |
| H | 2.55783400 | 6.25956700 | -0.44741800 |
| C | -3.99446600 | -4.76725500 | -0.79401600 |
| C | -5.36304300 | -5.07882300 | -1.37182200 |
| H | -3.96122700 | -5.05189500 | 0.26643600 |
| H | -3.22838200 | -5.37899600 | -1.28932000 |
| H | -5.61695800 | -6.13736500 | -1.26937700 |
| H | -6.14670200 | -4.50167100 | -0.86934300 |
| H | -5.40955400 | -4.83031900 | -2.43755100 |
| C | 0.92653800 | -3.72959300 | 0.92333700 |
| C | 1.04673700 | -4.10543900 | -0.41732700 |
| C | 1.06008900 | -4.72599300 | 1.89225300 |
| N | 1.27824700 | -5.34950100 | -0.83881300 |
| H | 0.93294000 | -3.33102200 | -1.18338600 |
| C | 1.31151400 | -6.02754200 | 1.47940300 |
| H | 0.96745100 | -4.45881300 | 2.94203100 |
| C | 1.41108600 | -6.28489900 | 0.11270400 |
| H | 1.42882000 | -6.83481200 | 2.19603300 |
| H | 1.60633900 | -7.29702400 | -0.23961600 |

**Ni[P(n-Bu)$_3$]$_2$(PyrCO)[PhOMe]**

| | | | |
|---|---|---|---|
| Ni | -0.35145900 | -0.59068200 | -0.24451800 |
| P | 1.86563300 | -0.38961600 | -0.14251500 |
| P | -0.89755100 | 1.59155600 | 0.17782600 |
| C | 0.16418700 | 2.96417700 | -0.49548500 |
| C | 2.84308500 | -1.88693500 | 0.31752200 |
| C | 2.27534200 | -0.08498600 | -1.91665500 |
| C | 2.63299200 | 0.95842500 | 0.87228800 |
| C | -2.58616900 | 2.10637500 | -0.34821700 |
| C | -0.93466100 | 1.85266800 | 2.00681200 |
| C | 0.53609300 | 4.11407600 | 0.43324500 |
| C | 1.51462700 | 5.08206200 | -0.21949700 |
| C | 1.98555100 | 6.17795400 | 0.71869000 |
| C | -3.02121500 | 3.52383200 | -0.01034900 |
| C | -1.73479800 | 0.74014100 | 2.67145000 |
| C | -1.67492900 | 0.73948900 | 4.19017300 |
| C | 3.66975600 | -0.36649200 | -2.46468400 |
| C | 3.75067800 | -0.09596700 | -3.96191600 |
| C | 3.79359300 | 1.76269900 | 0.29950700 |
| C | 4.18952400 | 2.91281300 | 1.21732400 |
| C | 4.32721900 | -1.72091700 | 0.62161900 |
| C | 5.01431500 | -3.05234100 | 0.89799100 |
| C | -2.40350900 | -0.45384200 | 4.78371200 |
| C | 5.12258600 | -0.38915700 | -4.54178300 |
| C | 6.48462800 | -2.89475100 | 1.24079800 |
| C | 5.24028800 | 3.82609900 | 0.61309700 |
| C | -4.43824700 | 3.83210500 | -0.47956900 |
| C | -4.87653500 | 5.24487500 | -0.13934000 |
| C | -2.20116500 | -1.05882900 | -0.64405700 |
| C | -0.12569000 | -2.39536800 | -0.75777000 |
| C | -0.29808800 | -3.46204400 | 0.28208400 |
| O | 0.18301600 | -2.71986900 | -1.90508000 |
| C | -0.44873100 | -3.12946000 | 1.62948800 |
| N | -0.59473800 | -4.01272600 | 2.62038800 |
| C | -0.60441400 | -5.30587900 | 2.26651100 |
| C | -0.29663600 | -4.81651300 | -0.05394100 |
| C | -0.45933300 | -5.75601800 | 0.95514500 |
| H | -0.34958100 | 3.34779500 | -1.38715800 |
| H | 1.08752200 | 2.50577600 | -0.87393900 |
| H | -0.36450500 | 4.65176200 | 0.76160200 |
| H | 0.99092600 | 3.71960500 | 1.35490000 |
| H | 1.05123400 | 5.52142200 | -1.11314400 |
| H | 2.37984700 | 4.51011500 | -0.58776500 |
| H | 1.14741300 | 6.78652700 | 1.07420900 |
| H | 2.47873400 | 5.75705000 | 1.60294200 |
| H | -3.28127000 | 1.36818600 | 0.07275900 |
| H | -2.62890800 | 1.93624800 | -1.43223800 |
| H | -2.96079400 | 3.69079500 | 1.07512900 |
| H | -2.32929500 | 4.25026600 | -0.46199700 |
| H | -1.32506100 | 2.84527700 | 2.26778800 |
| H | 0.10055300 | 1.82150400 | 2.37105300 |

| | | | |
|---|---|---|---|
| H | -2.78348200 | 0.77972000 | 2.34224400 |
| H | -1.35580100 | -0.22203400 | 2.29370600 |
| H | 1.98080000 | 0.95168900 | -2.13169900 |
| H | 1.54720800 | -0.71472100 | -2.44782100 |
| H | 4.42730200 | 0.23765000 | -1.94765800 |
| H | 3.93724900 | -1.41573000 | -2.27529900 |
| H | 2.91657200 | 0.50226700 | 1.83034700 |
| H | 1.82543400 | 1.65567600 | 1.12114100 |
| H | 4.66357600 | 1.11741500 | 0.11926800 |
| H | 3.51175600 | 2.16847900 | -0.68488900 |
| H | 2.68963800 | -2.60436500 | -0.50045700 |
| H | 2.33838300 | -2.33172300 | 1.18563600 |
| H | 4.83954800 | -1.21764300 | -0.20827000 |
| H | 4.45866100 | -1.06295200 | 1.49251900 |
| H | -2.09676100 | 1.67638300 | 4.57899300 |
| H | -0.62303500 | 0.73103900 | 4.50751100 |
| H | -3.46135600 | -0.45267400 | 4.49909800 |
| H | -1.97328100 | -1.39513200 | 4.42194000 |
| H | 3.47926900 | 0.95159200 | -4.15175800 |
| H | 2.98891800 | -0.69741000 | -4.47555700 |
| H | 5.89508300 | 0.22310700 | -4.06382600 |
| H | 5.40014600 | -1.43788500 | -4.39075500 |
| H | 4.90086700 | -3.70215100 | 0.01960700 |
| H | 4.49075900 | -3.56561000 | 1.71575200 |
| H | 6.96365500 | -3.85845200 | 1.43398300 |
| H | 4.54539500 | 2.50532700 | 2.17301000 |
| H | 3.28814300 | 3.49394200 | 1.46323300 |
| H | 5.50212900 | 4.64642900 | 1.28747600 |
| H | 6.16070100 | 3.27895500 | 0.38318300 |
| H | 4.88463000 | 4.27119500 | -0.32339000 |
| H | 2.70002200 | 6.84898000 | 0.23355100 |
| H | 7.03273800 | -2.41162400 | 0.42474300 |
| H | 6.61889400 | -2.27462200 | 2.13371800 |
| H | -2.35499600 | -0.46470300 | 5.87597500 |
| H | -5.13001800 | 3.10557800 | -0.03278700 |
| H | -4.50047400 | 3.67055600 | -1.56415500 |
| H | -5.89344200 | 5.45107400 | -0.48447900 |
| H | -4.85429200 | 5.41713000 | 0.94224700 |
| H | -4.21511100 | 5.98711300 | -0.59940400 |
| H | 5.16227200 | -0.18970000 | -5.61616600 |
| H | -0.43787300 | -2.06921300 | 1.90560400 |
| H | -0.73357300 | -6.02080300 | 3.07839400 |
| H | -0.17162100 | -5.09813400 | -1.09696300 |
| H | -0.47322100 | -6.82057700 | 0.74026400 |
| C | -3.17199100 | -1.43957300 | 0.29889400 |
| C | -4.51318200 | -1.59487900 | -0.04152800 |
| C | -4.92994800 | -1.38898400 | -1.35960500 |
| C | -2.64732800 | -0.89676600 | -1.96120800 |
| C | -3.98705000 | -1.04533400 | -2.33041700 |
| H | -2.88892100 | -1.61604300 | 1.33820600 |
| H | -5.25578800 | -1.88212700 | 0.69937300 |
| O | -6.26822900 | -1.55920200 | -1.59979900 |
| H | -1.92795700 | -0.64379100 | -2.74184600 |
| H | -4.27950600 | -0.90016700 | -3.36629400 |
| C | -6.70218400 | -1.36074300 | -2.92725400 |
| H | -7.78021800 | -1.52435600 | -2.92691600 |
| H | -6.49233700 | -0.34102000 | -3.27746600 |
| H | -6.23102300 | -2.06868000 | -3.62230300 |

**Decarbonylative transmetalation in the presence of $Na_2CO3$**

**$Ni[P(n\text{-}Bu)_3]_2(PyrCO)[Na_2CO_3](OPh)$, iso_1a**

| | | | |
|---|---|---|---|
| Ni | -0.33021400 | -0.86931300 | 0.26652500 |
| P | -2.34631400 | -0.08344800 | -0.02581400 |
| P | 0.85526400 | 1.13108800 | 0.06313000 |
| C | 0.07056100 | 2.63915200 | 0.81492600 |
| C | -3.59382400 | -1.22159500 | -0.77055900 |
| C | -2.91218300 | 0.19348400 | 1.70600000 |
| C | -2.55936600 | 1.47730700 | -0.99604800 |
| C | 2.56028700 | 1.13340100 | 0.74872100 |
| C | 1.09294700 | 1.43319000 | -1.74379000 |
| C | 0.14840000 | 3.95328100 | 0.04876100 |
| C | -0.69583300 | 5.04688700 | 0.69065200 |
| C | -0.72292400 | 6.33013500 | -0.11942700 |
| C | 3.22798300 | 2.47923200 | 1.00921500 |
| C | 2.28776000 | 2.25265200 | -2.21381000 |
| C | 2.42140300 | 2.20303600 | -3.72907700 |
| C | -4.39911800 | 0.27437000 | 2.03293800 |
| C | -3.54792500 | 2.52030700 | -0.48884500 |
| C | -4.91505800 | -0.62872700 | -1.24813600 |
| C | 3.64430100 | 2.95286200 | -4.22491700 |
| O | 1.31467100 | -1.91474200 | 0.56856400 |
| C | -1.15442600 | -2.55471400 | 0.54129300 |
| C | -0.95633200 | -3.54992400 | -0.55702500 |
| O | -1.68682700 | -2.88661800 | 1.59274000 |
| C | 1.82922200 | -1.88192800 | 1.78679100 |
| C | 3.11703800 | -2.41694100 | 2.02834500 |
| C | 3.69983600 | -2.32571100 | 3.28787700 |
| C | 3.03042000 | -1.71569900 | 4.35187800 |
| C | 1.15098100 | -1.29718000 | 2.88029300 |
| C | 1.74850900 | -1.21160500 | 4.13356600 |
| Na | 2.41615400 | -2.61850200 | -1.3483710 |
| O | 4.52762200 | -1.99760000 | -0.97598700 |
| C | 4.24817700 | -0.85051300 | -1.57020400 |
| O | 4.98720000 | 0.17660200 | -1.30531300 |
| O | 3.22029900 | -0.77975300 | -2.33796700 |
| Na | 5.66313500 | -0.86053800 | 0.55141100 |
| C | 4.69190100 | 2.30036900 | 1.39211000 |
| C | 5.38758500 | 3.60706000 | 1.72703700 |
| H | 0.51218700 | 2.75026800 | 1.81395200 |
| H | -0.98603100 | 2.40430400 | 1.00073300 |
| H | 1.19186000 | 4.28513500 | -0.03335700 |
| H | -0.19107700 | 3.79941500 | -0.98796600 |
| H | -0.32229600 | 5.24618700 | 1.70408200 |
| H | -1.72158600 | 4.66949500 | 0.82427800 |
| H | 0.28285900 | 6.74549100 | -0.24137900 |
| H | -1.12410600 | 6.15418200 | -1.12451400 |
| H | 3.15985000 | 0.56581700 | 0.02466400 |
| H | 2.53722700 | 0.55078700 | 1.67676700 |
| H | 3.17259400 | 3.11838100 | 0.11984400 |
| H | 2.70262200 | 3.02303800 | 1.80807100 |
| H | 0.15261300 | 1.82380200 | -2.15616200 |
| H | 1.22569300 | 0.42482800 | -2.15590400 |
| H | 2.21185400 | 3.29756900 | -1.87741100 |
| H | 3.20335700 | 1.82015200 | -1.78729600 |
| H | -2.38445400 | 1.08511800 | 2.07149600 |
| H | -2.47209500 | -0.65484300 | 2.24935700 |
| H | -4.88074700 | 1.09631100 | 1.48722600 |
| H | -4.89959300 | -0.64642400 | 1.70172300 |
| H | -2.81518200 | 1.16691600 | -2.01727300 |
| H | -1.57475700 | 1.94602400 | -1.08237100 |
| H | -4.56669600 | 2.11174400 | -0.46606600 |
| H | -3.30685200 | 2.78232200 | 0.55356800 |

| | | | |
|---|---|---|---|
| H | -3.77089600 | -2.00822600 | -0.02527300 |
| H | -3.09395400 | -1.72405900 | -1.60952100 |
| H | -5.40920400 | -0.07995200 | -0.43651800 |
| H | -4.73070800 | 0.11194700 | -2.03881500 |
| H | 1.51138400 | 2.60105400 | -4.20270700 |
| H | 2.49446800 | 1.14616200 | -4.01350800 |
| H | 3.60987500 | 4.01185500 | -3.94317000 |
| H | 4.55562600 | 2.52773800 | -3.79058800 |
| H | -1.34336600 | 7.09769800 | 0.35205600 |
| H | 3.74226300 | 2.90528000 | -5.31366500 |
| H | 3.65385100 | -2.86359100 | 1.19325300 |
| H | 4.69161400 | -2.74792200 | 3.44486400 |
| H | 3.49151500 | -1.65129200 | 5.33294600 |
| H | 0.14861300 | -0.89986200 | 2.71051700 |
| H | 1.20310000 | -0.74505500 | 4.95107900 |
| H | 5.19344200 | 1.80477900 | 0.54946100 |
| H | 4.75412100 | 1.61237900 | 2.25119700 |
| H | 6.43835000 | 3.45629200 | 1.99153700 |
| H | 5.35999900 | 4.29566200 | 0.87582400 |
| H | 4.90462900 | 4.11277900 | 2.57045300 |
| C | -0.52521300 | -3.13362400 | -1.81894900 |
| N | -0.27537500 | -3.95606700 | -2.84197700 |
| C | -0.48133900 | -5.26303600 | -2.62317500 |
| C | -1.17850900 | -4.91520400 | -0.36051600 |
| C | -0.93899500 | -5.78672400 | -1.41383700 |
| H | -0.36657300 | -2.06223000 | -1.99467800 |
| H | -0.26769800 | -5.92422900 | -3.46110300 |
| H | -1.52308900 | -5.25980600 | 0.61128500 |
| H | -1.09067300 | -6.85645700 | -1.30710100 |
| C | -5.86205500 | -1.69890100 | -1.77610500 |
| C | -7.17599300 | -1.12710700 | -2.27665300 |
| H | -6.05134700 | -2.43208700 | -0.98030000 |
| H | -5.36319900 | -2.25582100 | -2.58042800 |
| H | -7.84402600 | -1.90903900 | -2.64749100 |
| H | -7.70453600 | -0.59229700 | -1.48043800 |
| H | -7.01465500 | -0.41643400 | -3.09419700 |
| C | -4.63903000 | 0.45852700 | 3.52654300 |
| C | -6.11385400 | 0.51057100 | 3.88151200 |
| H | -4.14016500 | 1.37901100 | 3.85914000 |
| H | -4.14941000 | -0.35919800 | 4.07180900 |
| H | -6.26886700 | 0.64334400 | 4.95557600 |
| H | -6.61654000 | 1.33874700 | 3.37055900 |
| H | -6.62491000 | -0.41192500 | 3.58596200 |
| C | -3.51877800 | 3.78235300 | -1.34222800 |
| C | -4.39221200 | 4.89365600 | -0.79033900 |
| H | -3.82495200 | 3.53194300 | -2.36671000 |
| H | -2.47848200 | 4.13092700 | -1.42249900 |
| H | -4.35011000 | 5.79011600 | -1.41501500 |
| H | -5.44051000 | 4.58351800 | -0.72595100 |
| H | -4.07481000 | 5.18102200 | 0.21885100 |

**Ni[P(n-Bu)$_3$]$_2$(PyrCO)[Na$_2$CO$_3$](OPh), iso_1b**

| | | | |
|---|---|---|---|
| Ni | -0.27639100 | -0.80158400 | -0.31134200 |
| P | -2.16763600 | 0.30987100 | -0.28733500 |
| P | 1.13250200 | 0.98458900 | 0.25442800 |
| C | 0.55468200 | 2.43891600 | 1.25097000 |
| C | -3.63022200 | -0.42000800 | -1.14848100 |
| C | -2.64369400 | 0.38925300 | 1.49797000 |
| C | -2.10059500 | 2.04644600 | -0.92175000 |
| C | 2.75201500 | 0.51994000 | 0.98555100 |
| C | 1.56298700 | 1.59173400 | -1.43582700 |
| C | 0.94732100 | 3.83922700 | 0.79577000 |
| C | 0.21157500 | 4.92093600 | 1.57650900 |
| C | 0.46560200 | 6.31805200 | 1.04107500 |
| C | 3.62893800 | 1.62519800 | 1.56001700 |
| C | 2.89655200 | 2.29303100 | -1.66234900 |
| C | 3.09727600 | 2.61433500 | -3.13640900 |
| C | -4.09151400 | 0.66177400 | 1.89136700 |
| C | -2.90754800 | 3.13301900 | -0.22084800 |
| C | -4.80950000 | 0.49373600 | -1.46225600 |
| C | 4.46783400 | 3.20368000 | -3.41584100 |
| O | 1.19485700 | -2.07722100 | -0.13255600 |
| C | -0.99088700 | -2.21540300 | -1.32653200 |
| O | -0.54767800 | -2.31211300 | -2.47126600 |
| C | -1.91543900 | -3.24845300 | -0.79093300 |
| C | 1.53904700 | -2.49092500 | 1.07136400 |
| C | 2.66240700 | -3.33934300 | 1.22440800 |
| C | 3.07250600 | -3.75435600 | 2.48633000 |
| C | 2.38842300 | -3.35354800 | 3.63759800 |
| C | 0.84752700 | -2.10774100 | 2.24351600 |
| C | 1.27130800 | -2.53095400 | 3.49944700 |
| Na | 1.97924000 | -2.41319300 | -2.2681830 |
| O | 4.15607500 | -2.42819000 | -1.62186900 |
| C | 4.17317000 | -1.13938000 | -1.90487100 |
| O | 5.02798100 | -0.38225400 | -1.29302100 |
| O | 3.31329700 | -0.67102500 | -2.73637200 |
| Na | 5.35264100 | -1.99887000 | 0.19533700 |
| C | 5.00654500 | 1.08860700 | 1.92926800 |
| C | 5.89405600 | 2.12223700 | 2.59813600 |
| H | 0.87872500 | 2.26121900 | 2.28494600 |
| H | -0.54139700 | 2.39156100 | 1.28517600 |
| H | 2.03181200 | 3.98456000 | 0.88472200 |
| H | 0.72199100 | 3.95483900 | -0.27592800 |
| H | 0.49442100 | 4.86294500 | 2.63608100 |
| H | -0.86727700 | 4.70268500 | 1.54838000 |
| H | 1.52836900 | 6.57709300 | 1.08972500 |
| H | 0.16118300 | 6.39869400 | -0.00915300 |
| H | 3.29213600 | 0.00823800 | 0.17566900 |
| H | 2.55465300 | -0.23001800 | 1.75999300 |
| H | 3.75068700 | 2.44089100 | 0.83741700 |
| H | 3.15341800 | 2.07085700 | 2.44663400 |
| H | 0.72484400 | 2.19749100 | -1.80661500 |
| H | 1.58729300 | 0.67979400 | -2.04816600 |
| H | 2.97116900 | 3.21388600 | -1.06596700 |
| H | 3.70408000 | 1.61469400 | -1.35257300 |
| H | -1.97172000 | 1.11866500 | 1.96850700 |
| H | -2.33807400 | -0.57924800 | 1.91536500 |
| H | -4.43477800 | 1.61910000 | 1.47833200 |
| H | -4.74516200 | -0.10836800 | 1.45789200 |
| H | -2.36790400 | 1.99172100 | -1.98489400 |
| H | -1.04624800 | 2.33932200 | -0.91187500 |
| H | -3.98365300 | 2.92903800 | -0.29323100 |
| H | -2.67415600 | 3.13780700 | 0.85585700 |
| H | -3.96140600 | -1.27390300 | -0.54189000 |
| H | -3.24946600 | -0.85788900 | -2.08130000 |
| H | -5.19454200 | 0.95358300 | -0.54373900 |
| H | -4.47852300 | 1.32740100 | -2.09714900 |
| H | 2.30877000 | 3.29925200 | -3.48338600 |
| H | 2.97956500 | 1.67617900 | -3.69142800 |
| H | 4.63355400 | 4.12916100 | -2.85132900 |
| H | 5.25276000 | 2.49711300 | -3.12531900 |
| H | -0.08654600 | 7.07694700 | 1.60283900 |
| H | 4.60692500 | 3.43585100 | -4.47623200 |
| H | 3.19324800 | -3.63469800 | 0.32073900 |

| | | | |
|---|---|---|---|
| H | 3.93413800 | -4.41468200 | 2.57381900 |
| H | 2.71153100 | -3.68834300 | 4.61866300 |
| H | -0.01277500 | -1.44253200 | 2.13735200 |
| H | 0.71928300 | -2.21335000 | 4.38145400 |
| H | 5.47658300 | 0.72237100 | 1.00611500 |
| H | 4.88464700 | 0.21409700 | 2.58839400 |
| H | 6.88236400 | 1.71976500 | 2.83887100 |
| H | 6.04347300 | 2.99086400 | 1.94806800 |
| H | 5.45010500 | 2.48649600 | 3.53124500 |
| C | -2.23592900 | -3.28668900 | 0.56815000 |
| N | -3.08892900 | -4.15545300 | 1.11629000 |
| C | -3.66080900 | -5.03713300 | 0.28578200 |
| C | -2.51290400 | -4.19108300 | -1.63286600 |
| C | -3.40448900 | -5.10080000 | -1.08437700 |
| H | -1.75300800 | -2.57566300 | 1.24264200 |
| H | -4.36537400 | -5.73138400 | 0.74121000 |
| H | -2.27239300 | -4.18234600 | -2.69294300 |
| H | -3.90099900 | -5.84616300 | -1.69799300 |
| C | -5.94050900 | -0.25032300 | -2.16053700 |
| C | -7.11531900 | 0.65039300 | -2.49604700 |
| H | -6.27332800 | -1.07835100 | -1.51990900 |
| H | -5.55254400 | -0.71932900 | -3.07471500 |
| H | -7.91931900 | 0.10158400 | -2.99333300 |
| H | -7.53608500 | 1.10564300 | -1.59308400 |
| H | -6.81261000 | 1.46634800 | -3.16091100 |
| C | -2.60514000 | 4.51054100 | -0.79894700 |
| C | -3.31354400 | 5.63263600 | -0.06280300 |
| H | -2.87796800 | 4.52327500 | -1.86241700 |
| H | -1.51726200 | 4.67433600 | -0.77161600 |
| H | -3.07051600 | 6.61118000 | -0.48607300 |
| H | -4.40133600 | 5.51410300 | -0.10533200 |
| H | -3.02976000 | 5.65262500 | 0.99574500 |
| C | -4.27143300 | 0.67561200 | 3.40421000 |
| C | -5.70883700 | 0.92626600 | 3.82226900 |
| H | -3.61567100 | 1.44430800 | 3.83540000 |
| H | -3.92142600 | -0.28026100 | 3.81648300 |
| H | -5.81929000 | 0.93306800 | 4.90984900 |
| H | -6.06930200 | 1.89046000 | 3.44810200 |
| H | -6.37715300 | 0.15359000 | 3.42773100 |

**Ni[P(n-Bu)$_3$]$_2$(PyrCO)[Na$_2$CO$_3$OPh], iso_3a**

| | | | |
|---|---|---|---|
| Ni | 0.12947700 | -0.24948100 | -0.89960100 |
| P | -1.94533500 | -1.22011500 | -0.27805300 |
| P | -0.47075400 | 1.83002000 | -0.50002300 |
| C | 1.93194700 | 0.27945800 | -1.10777600 |
| O | 0.67561900 | -1.91924300 | -1.70089300 |
| C | 1.03128500 | -2.85419700 | -0.82513400 |
| O | 0.78347200 | -2.68886200 | 0.41691400 |
| O | 1.67797000 | -3.85388600 | -1.29189600 |
| Na | 2.91907100 | -3.64118000 | 0.64181900 |
| Na | 2.89857900 | -2.14750000 | -2.4835090 |
| O | 4.16307600 | -2.26386400 | -0.61466200 |
| C | 5.18413400 | -1.55927400 | -0.22257500 |
| C | 5.62202400 | -1.54627100 | 1.13155100 |
| C | 6.70102700 | -0.78018000 | 1.54974500 |
| C | 7.41360500 | 0.01407300 | 0.64743600 |
| C | 5.93139100 | -0.74219200 | -1.11733300 |
| C | 7.01287600 | 0.01882200 | -0.68984400 |
| C | -2.14234900 | -2.87679200 | -1.05871500 |
| C | -1.87905800 | -1.49720800 | 1.54873800 |
| C | -3.57554000 | -0.37945600 | -0.56699100 |
| C | 0.79155300 | 3.17564400 | -0.53475200 |

| | | | |
|---|---|---|---|
| C | -1.70441600 | 2.31891900 | -1.78972700 |
| C | -1.28085700 | 1.99974700 | 1.15042700 |
| C | -4.75370900 | -0.67526200 | 0.35372100 |
| C | -5.94627500 | 0.22393000 | 0.05123700 |
| C | -3.51598800 | -3.53322900 | -1.10916700 |
| C | -3.47686900 | -4.86769700 | -1.84322800 |
| C | -1.49765300 | 3.36937000 | 1.78115200 |
| C | -2.84941300 | 3.24439000 | -1.39172600 |
| C | 0.33525300 | 4.59887200 | -0.83482600 |
| C | -4.83572300 | -5.54009800 | -1.92112600 |
| C | -7.13937400 | -0.05022600 | 0.94835100 |
| O | 2.53015000 | 0.32763800 | -2.18208000 |
| C | 2.64104300 | 0.61924800 | 0.15940900 |
| C | -2.55068600 | -2.73985200 | 2.11734100 |
| C | -2.32225700 | -2.88142500 | 3.61653200 |
| C | -2.97543400 | -4.12229200 | 4.19811900 |
| H | 5.06674300 | -2.14156900 | 1.85902200 |
| H | 6.99063000 | -0.79689300 | 2.59980900 |
| H | 8.26070400 | 0.60905200 | 0.97713600 |
| H | 5.62273100 | -0.71876500 | -2.16279600 |
| H | 7.55215300 | 0.62970300 | -1.41302500 |
| H | -3.84754600 | -0.57677500 | -1.61371700 |
| H | -3.38564700 | 0.69948500 | -0.52743100 |
| H | -5.05343700 | -1.72821600 | 0.27668000 |
| H | -4.45346800 | -0.52508800 | 1.40100300 |
| H | -1.42095700 | -3.52309600 | -0.54592100 |
| H | -1.74260900 | -2.76252500 | -2.07338500 |
| H | -3.90870200 | -3.69428200 | -0.09625500 |
| H | -4.23712500 | -2.86885300 | -1.60763300 |
| H | -0.66863300 | 1.38423000 | 1.82422700 |
| H | -2.24039800 | 1.47045300 | 1.07668000 |
| H | -0.52806800 | 3.85155200 | 1.96741300 |
| H | -2.04028300 | 4.03356300 | 1.09678900 |
| H | -2.10959200 | 1.38021500 | -2.18585000 |
| H | -1.12837100 | 2.75163500 | -2.61770000 |
| H | -3.32764800 | 2.87935200 | -0.46971700 |
| H | -2.46274300 | 4.24305000 | -1.14731000 |
| H | 1.54071600 | 2.87488000 | -1.27863900 |
| H | 1.31272800 | 3.13768600 | 0.43186900 |
| H | -0.13609700 | 4.64056500 | -1.82664700 |
| H | -0.43431800 | 4.92375000 | -0.12339900 |
| H | -2.75863200 | -5.52983300 | -1.34119100 |
| H | -3.07621500 | -4.71063200 | -2.85374400 |
| H | -4.78861500 | -6.49601000 | -2.45027700 |
| H | -5.23940000 | -5.73638100 | -0.92181100 |
| H | -5.56157500 | -4.90871900 | -2.44540900 |
| H | -6.23247600 | 0.10423000 | -1.00318400 |
| H | -5.63174500 | 1.27352400 | 0.15493800 |
| H | -7.98125800 | 0.60965100 | 0.72169700 |
| H | -7.48980300 | -1.08184300 | 0.83689200 |
| H | -6.88252300 | 0.09288700 | 2.00350700 |
| H | -2.27545300 | -0.59420100 | 2.03272600 |
| H | -0.80807500 | -1.53527200 | 1.77529600 |
| H | -3.63066700 | -2.72392400 | 1.91562500 |
| H | -2.15961700 | -3.63112700 | 1.60672800 |
| H | -2.70250200 | -1.98456800 | 4.12537400 |
| H | -1.24176600 | -2.89831200 | 3.81312900 |
| H | -2.80123900 | -4.20957000 | 5.27424700 |
| H | -4.05933400 | -4.11184100 | 4.03941700 |
| H | -2.58767800 | -5.03161200 | 3.72626700 |
| C | 2.10301300 | 0.25526200 | 1.39775300 |
| N | 2.63930800 | 0.58905900 | 2.57596000 |

| | | | |
|---|---|---|---|
| C | 3.75956200 | 1.32684000 | 2.53575000 |
| C | 3.82898900 | 1.35632900 | 0.14767500 |
| C | 4.39224600 | 1.72650400 | 1.36085600 |
| H | 1.20234100 | -0.36629500 | 1.41454900 |
| H | 4.17624300 | 1.60948600 | 3.50219500 |
| H | 4.27274800 | 1.63401400 | -0.80440000 |
| H | 5.31289500 | 2.30021900 | 1.40130600 |
| C | 1.49765700 | 5.58375700 | -0.79569100 |
| C | 1.07715600 | 7.00334400 | -1.12961400 |
| H | 2.27623100 | 5.24766500 | -1.49371500 |
| H | 1.95902400 | 5.55213500 | 0.20072400 |
| H | 1.92212800 | 7.69639100 | -1.09610500 |
| H | 0.64211600 | 7.06226100 | -2.13310500 |
| H | 0.32200100 | 7.36999200 | -0.42608100 |
| C | -3.90868200 | 3.35178700 | -2.48069900 |
| C | -5.06284100 | 4.25702300 | -2.09025800 |
| H | -4.28444000 | 2.34370400 | -2.70880100 |
| H | -3.44219500 | 3.71193200 | -3.40726400 |
| H | -5.81567200 | 4.32048200 | -2.88041400 |
| H | -5.56298700 | 3.89166600 | -1.18648100 |
| H | -4.71637200 | 5.27470400 | -1.88112400 |
| C | -2.27296500 | 3.25968400 | 3.08836600 |
| C | -2.47930000 | 4.60081600 | 3.76804100 |
| H | -1.74610600 | 2.57162100 | 3.76332800 |
| H | -3.24509400 | 2.78766800 | 2.88260700 |
| H | -3.04427800 | 4.50280300 | 4.69892200 |
| H | -1.52119100 | 5.07209000 | 4.01162300 |
| H | -3.02743900 | 5.29358400 | 3.12047300 |

**Ni[P(n-Bu)$_3$]$_2$(PyrCO)[Na$_2$CO$_3$OPh], iso_3b**

| | | | |
|---|---|---|---|
| Ni | 0.21266300 | -0.18031800 | -0.18821400 |
| P | -1.88689100 | -1.25411400 | -0.21119300 |
| P | -0.52739000 | 1.85044600 | 0.09639700 |
| C | 1.96737700 | 0.42178100 | 0.15304100 |
| O | 1.10846600 | -1.77381200 | -0.92068800 |
| C | 1.15808800 | -2.71084900 | 0.01911200 |
| O | 0.61471700 | -2.49274700 | 1.15356800 |
| O | 1.82167000 | -3.77234100 | -0.25181200 |
| Na | 2.70698900 | -3.43005800 | 1.87038900 |
| Na | 3.39594200 | -2.46645700 | -1.3002090 |
| O | 4.37393100 | -2.47889000 | 0.72944600 |
| C | 5.31462000 | -1.59602900 | 0.91078700 |
| C | 5.36085900 | -0.75528900 | 2.05808400 |
| C | 6.33337700 | 0.22375600 | 2.20806000 |
| C | 7.32386900 | 0.41465000 | 1.23893700 |
| C | 6.35133700 | -1.39766400 | -0.04400500 |
| C | 7.32276000 | -0.41616900 | 0.11759400 |
| C | -1.78306500 | -2.89063400 | -1.05477400 |
| C | -2.37922900 | -1.59956800 | 1.53689600 |
| C | -3.37772100 | -0.44124600 | -0.96364200 |
| C | 0.66192800 | 3.20628400 | 0.48606000 |
| C | -1.34128000 | 2.39652000 | -1.47444200 |
| C | -1.79111200 | 1.93225100 | 1.44126900 |
| C | -4.77101700 | -0.79085800 | -0.45446200 |
| C | -5.84021900 | 0.10487600 | -1.06803600 |
| C | -3.05384500 | -3.58155500 | -1.53306200 |
| C | -2.75325100 | -4.89484900 | -2.24465600 |
| C | -2.22694300 | 3.25657600 | 2.05451500 |
| C | -2.58628300 | 3.27432900 | -1.39382400 |
| C | 0.25476000 | 4.63913500 | 0.16137700 |
| C | -4.00363200 | -5.59258400 | -2.74888800 |
| C | -7.24093000 | -0.22475300 | -0.58552300 |
| C | 2.85534100 | 0.69933300 | -1.01792100 |
| O | 2.39168100 | 0.50230200 | 1.29586100 |
| C | -3.17432100 | -2.86357800 | 1.83553300 |
| C | -3.45235500 | -3.02673600 | 3.32433700 |
| C | -4.23064000 | -4.29014100 | 3.64505500 |
| H | 4.57765300 | -0.86980800 | 2.80554200 |
| H | 6.31864200 | 0.85606800 | 3.09457100 |
| H | 8.08289100 | 1.18158900 | 1.36307000 |
| H | 6.36325000 | -2.03893000 | -0.92689700 |
| H | 8.08992900 | -0.29472300 | -0.64635000 |
| H | -3.31264500 | -0.60874600 | -2.04833500 |
| H | -3.23842400 | 0.64016800 | -0.84158700 |
| H | -5.00444000 | -1.84374200 | -0.65844300 |
| H | -4.80599000 | -0.68316400 | 0.63952600 |
| H | -1.22335100 | -3.53878900 | -0.36920200 |
| H | -1.10552900 | -2.73113800 | -1.90246400 |
| H | -3.73370200 | -3.77810100 | -0.69318400 |
| H | -3.60714800 | -2.92289000 | -2.21816700 |
| H | -1.38805500 | 1.28465200 | 2.23133200 |
| H | -2.67129200 | 1.39707500 | 1.06076300 |
| H | -1.36486800 | 3.75747200 | 2.51613900 |
| H | -2.60018800 | 3.94219100 | 1.28324700 |
| H | -1.57708200 | 1.47591200 | -2.02255700 |
| H | -0.55860300 | 2.88846200 | -2.06723300 |
| H | -3.30144100 | 2.85164900 | -0.67072600 |
| H | -2.32871300 | 4.26978500 | -1.00654900 |
| H | 1.58679500 | 2.96775700 | -0.05448900 |
| H | 0.91783600 | 3.10030600 | 1.54874100 |
| H | 0.09877200 | 4.74264900 | -0.92176000 |
| H | -0.70719900 | 4.89076500 | 0.62438000 |
| H | -2.20460500 | -5.55566100 | -1.56010800 |
| H | -2.06761300 | -4.70174000 | -3.08078200 |
| H | -3.76971800 | -6.53132600 | -3.25887900 |
| H | -4.68735000 | -5.82696400 | -1.92558800 |
| H | -4.55278300 | -4.96117700 | -3.45612600 |
| H | -5.79154500 | 0.03068500 | -2.16342300 |
| H | -5.59977300 | 1.15249200 | -0.83179900 |
| H | -7.99110100 | 0.43630500 | -1.02801600 |
| H | -7.51634500 | -1.25361700 | -0.84024900 |
| H | -7.31747200 | -0.12871200 | 0.50286200 |
| H | -2.92556100 | -0.71826300 | 1.90009600 |
| H | -1.43030500 | -1.62705200 | 2.08292900 |
| H | -4.12720000 | -2.85955400 | 1.28898200 |
| H | -2.62123000 | -3.74189800 | 1.47323800 |
| H | -4.00261800 | -2.14639200 | 3.68530800 |
| H | -2.49841500 | -3.02393700 | 3.86863000 |
| H | -4.42284100 | -4.39100500 | 4.71696100 |
| H | -5.19947600 | -4.29923700 | 3.13383300 |
| H | -3.68529100 | -5.18342100 | 3.32209500 |
| C | 2.33800800 | 0.66320000 | -2.31809100 |
| N | 3.04853300 | 0.91476100 | -3.42013500 |
| C | 4.34498900 | 1.21781700 | -3.23847300 |
| C | 4.20836600 | 1.01622200 | -0.85653900 |
| C | 4.96474700 | 1.27828400 | -1.99303600 |
| H | 1.28301800 | 0.40603700 | -2.45011400 |
| H | 4.91517900 | 1.42193100 | -4.14408000 |
| H | 4.64258700 | 1.03913100 | 0.14144900 |
| H | 6.02145100 | 1.51668000 | -1.91370800 |
| C | 1.30437300 | 5.64792700 | 0.61076800 |
| C | 0.93729200 | 7.07672200 | 0.25307400 |
| H | 2.27188200 | 5.38501500 | 0.16252300 |
| H | 1.44605300 | 5.55680800 | 1.69622800 |

| | | | |
|---|---|---|---|
| H | 1.69709000 | 7.78836900 | 0.58722400 |
| H | 0.82526200 | 7.19795000 | -0.82969500 |
| H | -0.01307000 | 7.36896800 | 0.71275200 |
| C | -3.28084700 | 3.40985900 | -2.74252500 |
| C | -4.53367800 | 4.26399500 | -2.67600000 |
| H | -3.53092200 | 2.40493400 | -3.11265000 |
| H | -2.57591800 | 3.82824100 | -3.47318900 |
| H | -5.02316000 | 4.34544600 | -3.65019100 |
| H | -5.26226600 | 3.84275700 | -1.97452200 |
| H | -4.30491700 | 5.27959400 | -2.33582600 |
| C | -3.31542100 | 3.04695100 | 3.10052400 |
| C | -3.76811800 | 4.34082100 | 3.75185400 |
| H | -2.95061400 | 2.34713700 | 3.86457000 |
| H | -4.17053300 | 2.54550100 | 2.62357900 |
| H | -4.55284600 | 4.17081600 | 4.49382800 |
| H | -2.93657800 | 4.84041000 | 4.25998100 |
| H | -4.16270900 | 5.04170800 | 3.00856000 |

**Ni[P(n-Bu)$_3$]$_2$(PyrCO)[NaCO$_3$], iso_3a**

| | | | |
|---|---|---|---|
| Ni | -0.36589100 | -1.12021800 | -0.50636400 |
| P | 1.72393200 | -0.13950000 | -0.01017900 |
| P | -1.54814800 | 0.70059400 | -0.36872100 |
| C | -1.88325600 | -2.10241400 | -1.01789100 |
| O | 0.34560300 | -2.93653400 | -0.30909200 |
| C | 1.15754200 | -3.13454800 | -1.38175700 |
| O | 1.37739400 | -2.14358400 | -2.13275000 |
| O | 1.62226600 | -4.31153700 | -1.50606000 |
| Na | 0.25874000 | -5.13038500 | 0.04560300 |
| C | 2.91563700 | -1.43383700 | 0.54196300 |
| C | 2.47518300 | 0.64160400 | -1.50933300 |
| C | 1.82683900 | 1.16881300 | 1.30632500 |
| C | -3.28026000 | 0.71821300 | -1.00875300 |
| C | -1.72011800 | 1.13436900 | 1.42558200 |
| C | -0.72302000 | 2.13158000 | -1.19919100 |
| C | 2.94828600 | 2.20134800 | 1.28430700 |
| C | 2.73630500 | 3.28490900 | 2.33408000 |
| C | 4.13789500 | -1.03458400 | 1.35583300 |
| C | -1.46896200 | 3.42303100 | -1.50935300 |
| C | -0.52968200 | 4.47124200 | -2.09433400 |
| C | -1.70938300 | 2.60302500 | 1.83427000 |
| C | -1.60702100 | 2.77979800 | 3.34373200 |
| C | -4.24887200 | 1.75573700 | -0.45243800 |
| C | -5.63189700 | 1.64248700 | -1.08189400 |
| C | -6.61844400 | 2.64930100 | -0.51854400 |
| C | -1.23134500 | 5.77292100 | -2.43578400 |
| C | 3.84701000 | 4.31917000 | 2.35952100 |
| C | -1.56669200 | 4.23745300 | 3.76465200 |
| C | -2.60748600 | -2.84662200 | 0.06792700 |
| O | -2.25545100 | -2.20997400 | -2.17738200 |
| C | 3.99115800 | 0.61238700 | -1.65590900 |
| H | 1.81032700 | 0.64374200 | 2.27261000 |
| H | 0.87386100 | 1.71160300 | 1.27402000 |
| H | 3.92336400 | 1.72050000 | 1.43765900 |
| H | 3.00112200 | 2.67383700 | 0.29236900 |
| H | 3.20928700 | -1.96214200 | -0.37292000 |
| H | 2.30927000 | -2.15869100 | 1.09955900 |
| H | 4.75659800 | -0.31831400 | 0.79951000 |
| H | 3.83074200 | -0.51368500 | 2.27433200 |
| H | -0.32108100 | 1.71268500 | -2.13096700 |
| H | 0.16226700 | 2.35947100 | -0.59046200 |
| H | -2.28657900 | 3.22534600 | -2.21644600 |
| H | -1.94005100 | 3.83082500 | -0.60603200 |
| H | -0.90459500 | 0.60758300 | 1.93629600 |
| H | -2.63795800 | 0.64240800 | 1.77530500 |
| H | -0.85945100 | 3.11972900 | 1.36194800 |
| H | -2.60822000 | 3.11209300 | 1.46001000 |
| H | -3.68800500 | -0.28315900 | -0.82328800 |
| H | -3.20570700 | 0.79660200 | -2.10145300 |
| H | -4.33967800 | 1.63083800 | 0.63589500 |
| H | -3.86774100 | 2.77302100 | -0.60378800 |
| H | -6.01101500 | 0.62196600 | -0.93543900 |
| H | -5.54246400 | 1.77210900 | -2.16884900 |
| H | -7.60567800 | 2.55402100 | -0.97870100 |
| H | -6.74409000 | 2.51974000 | 0.56182000 |
| H | -6.27518000 | 3.67621400 | -0.68435300 |
| H | -0.04128200 | 4.05992700 | -2.98829200 |
| H | 0.28077400 | 4.65718700 | -1.37391100 |
| H | -0.53950500 | 6.51261500 | -2.84740700 |
| H | -2.02266700 | 5.61390400 | -3.17590600 |
| H | -1.69928700 | 6.21518600 | -1.54961000 |
| H | 2.63611800 | 2.81580500 | 3.32299100 |
| H | 1.77063400 | 3.77697600 | 2.14239100 |
| H | 3.66870600 | 5.09225100 | 3.11201600 |
| H | 4.81389800 | 3.85650800 | 2.58469500 |
| H | 3.94557700 | 4.81900300 | 1.38984500 |
| H | -0.70405300 | 2.26181400 | 3.69829200 |
| H | -2.45052300 | 2.26936800 | 3.82766400 |
| H | -1.48627700 | 4.34616900 | 4.84952100 |
| H | -0.71072800 | 4.75450900 | 3.31681200 |
| H | -2.46977300 | 4.76861700 | 3.44551200 |
| H | 2.11202100 | 1.67716700 | -1.56020800 |
| H | 2.01911200 | 0.10116400 | -2.34559400 |
| H | 4.47208000 | 1.16804000 | -0.83829500 |
| H | 4.34804900 | -0.42359200 | -1.56826900 |
| C | -2.10673500 | -2.90773600 | 1.37301500 |
| N | -2.71610900 | -3.53464200 | 2.38467300 |
| C | -3.89826100 | -4.10927300 | 2.11278300 |
| C | -3.83621100 | -3.46003700 | -0.18695100 |
| C | -4.49921400 | -4.09516600 | 0.85615300 |
| H | -1.14843500 | -2.42773200 | 1.58679800 |
| H | -4.39020400 | -4.60439800 | 2.94918800 |
| H | -4.24361800 | -3.42126000 | -1.19492400 |
| H | -5.46274900 | -4.57332500 | 0.70607500 |
| C | 4.99246500 | -2.23923700 | 1.73101000 |
| C | 6.21137700 | -1.86457600 | 2.55463800 |
| H | 5.30344100 | -2.75607700 | 0.81308600 |
| H | 4.37385800 | -2.96139200 | 2.28173200 |
| H | 6.81473000 | -2.73889800 | 2.81439200 |
| H | 6.85795700 | -1.16840000 | 2.00948900 |
| H | 5.92260900 | -1.37312300 | 3.49038000 |
| C | 4.45210700 | 1.18749900 | -2.98885600 |
| C | 5.96015200 | 1.14420400 | -3.15841300 |
| H | 4.09456000 | 2.22307400 | -3.07768700 |
| H | 3.96652400 | 0.63413100 | -3.80337100 |
| H | 6.27650700 | 1.56140200 | -4.11859400 |
| H | 6.46516600 | 1.71236400 | -2.36940500 |
| H | 6.33317900 | 0.11574900 | -3.10675000 |

**Ni[P(n-Bu)$_3$]$_2$(PyrCO)[NaCO$_3$], iso_3b**

| | | | |
|---|---|---|---|
| Ni | 0.17409100 | -1.18831800 | -0.76989600 |
| P | -1.79507600 | -0.01571500 | -0.23039600 |
| P | 1.55042800 | 0.49554000 | -0.45988000 |
| C | 1.58793300 | -2.43762600 | -0.90032600 |
| O | -0.88963000 | -2.62144100 | -1.46579300 |

| | | | |
|---|---|---|---|
| C | -1.29146600 | -3.44052300 | -0.45253900 |
| O | -1.41560600 | -2.94179000 | 0.70001500 |
| O | -1.47673400 | -4.65118100 | -0.79477600 |
| Na | -0.07445500 | -4.38780400 | -2.5481600 |
| C | -3.26247800 | -0.87757300 | -0.93173200 |
| C | -1.97993300 | -0.03899500 | 1.60778000 |
| C | -2.03776600 | 1.76671500 | -0.69232000 |
| C | 3.37213600 | 0.18606800 | -0.46588800 |
| C | 1.27947500 | 1.69196500 | -1.84643200 |
| C | 1.24208900 | 1.39594900 | 1.12376700 |
| C | -2.97487100 | 2.64842500 | 0.12460200 |
| C | -2.93427300 | 4.09829500 | -0.34321700 |
| C | -4.58788600 | -0.13960600 | -1.05363500 |
| C | 2.27480900 | 2.36427900 | 1.68640900 |
| C | 1.78577600 | 3.01171500 | 2.97624800 |
| C | 1.40479900 | 3.18545100 | -1.56452600 |
| C | 0.92088600 | 4.03158600 | -2.73472700 |
| C | 4.30420400 | 1.32597900 | -0.86188500 |
| C | 5.76851800 | 0.91041700 | -0.79693000 |
| C | 6.71486800 | 2.02342500 | -1.20899600 |
| C | 2.79267000 | 3.97911700 | 3.57115000 |
| C | -3.85450800 | 5.00640900 | 0.45188300 |
| C | 1.00878800 | 5.52160400 | -2.45958700 |
| O | 1.92444400 | -3.05416500 | -1.91506800 |
| C | 2.27735000 | -2.75004500 | 0.39198700 |
| C | -3.38445500 | -0.15487300 | 2.18399300 |
| H | -2.32978000 | 1.77919600 | -1.75238100 |
| H | -1.04412200 | 2.23155500 | -0.67269100 |
| H | -4.00516800 | 2.27389600 | 0.07605800 |
| H | -2.69176200 | 2.60825100 | 1.18647700 |
| H | -3.36365200 | -1.78802400 | -0.33001800 |
| H | -2.93604200 | -1.23300900 | -1.91571500 |
| H | -4.93946500 | 0.19796000 | -0.06972100 |
| H | -4.46361900 | 0.77200800 | -1.65686800 |
| H | 1.04945500 | 0.60561800 | 1.86238100 |
| H | 0.27872100 | 1.90964900 | 1.00151400 |
| H | 3.21837900 | 1.83471100 | 1.87889600 |
| H | 2.51039200 | 3.14934700 | 0.95646200 |
| H | 0.27751300 | 1.47605200 | -2.23600900 |
| H | 1.96732300 | 1.39217400 | -2.64764800 |
| H | 0.82019500 | 3.45321100 | -0.67064500 |
| H | 2.44547800 | 3.44250800 | -1.32426500 |
| H | 3.53676600 | -0.66196800 | -1.14362300 |
| H | 3.62967500 | -0.18900500 | 0.53474500 |
| H | 4.06911700 | 1.66276100 | -1.88141600 |
| H | 4.15148100 | 2.20034200 | -0.21731000 |
| H | 5.92147300 | 0.03026700 | -1.43593400 |
| H | 6.00032000 | 0.57907700 | 0.22457700 |
| H | 7.76070500 | 1.70927800 | -1.15612100 |
| H | 6.52000600 | 2.34950600 | -2.23633300 |
| H | 6.60087400 | 2.90032500 | -0.56268200 |
| H | 1.54295400 | 2.22557800 | 3.70372700 |
| H | 0.83834400 | 3.53192000 | 2.77439800 |
| H | 2.42250400 | 4.43415100 | 4.49375200 |
| H | 3.73536900 | 3.47438400 | 3.80791400 |
| H | 3.02386900 | 4.79042900 | 2.87252700 |
| H | -3.19406000 | 4.14027900 | -1.41036400 |
| H | -1.89804900 | 4.46329700 | -0.27809500 |
| H | -3.80902600 | 6.04163200 | 0.10255500 |
| H | -4.89653700 | 4.67809100 | 0.37597600 |
| H | -3.59082100 | 5.00459400 | 1.51489300 |
| H | -0.11808800 | 3.75454800 | -2.96518400 |
| H | 1.50200700 | 3.77690300 | -3.63113900 |
| H | 0.65985400 | 6.11354200 | -3.30987000 |
| H | 0.39995800 | 5.79925000 | -1.59204500 |
| H | 2.03869100 | 5.82637100 | -2.24538300 |
| H | -1.48381000 | 0.86009800 | 1.99859100 |
| H | -1.38723200 | -0.90210100 | 1.92872800 |
| H | -4.00246400 | 0.69899000 | 1.87236000 |
| H | -3.87268300 | -1.05039100 | 1.77553000 |
| C | 1.72476800 | -2.36400100 | 1.61910500 |
| N | 2.33440400 | -2.52717100 | 2.79739800 |
| C | 3.54431500 | -3.10292500 | 2.77604800 |
| C | 3.52395100 | -3.38038200 | 0.40335900 |
| C | 4.17275700 | -3.55740900 | 1.61732500 |
| H | 0.72047200 | -1.93176200 | 1.63137600 |
| H | 4.03772000 | -3.20785600 | 3.74165400 |
| H | 3.96261500 | -3.69749000 | -0.54010400 |
| H | 5.15014600 | -4.02731800 | 1.67452200 |
| C | -5.66280600 | -1.01165900 | -1.69137800 |
| C | -6.98953200 | -0.29200500 | -1.85341900 |
| H | -5.79671100 | -1.91532600 | -1.08209500 |
| H | -5.30550900 | -1.36491400 | -2.66831400 |
| H | -7.74836000 | -0.93230500 | -2.31198700 |
| H | -7.38087500 | 0.04178600 | -0.88627600 |
| H | -6.88359400 | 0.59744200 | -2.48436500 |
| C | -3.37780900 | -0.23968600 | 3.70467600 |
| C | -4.77143500 | -0.37149600 | 4.29218400 |
| H | -2.88172900 | 0.65204900 | 4.11336600 |
| H | -2.75857700 | -1.09274600 | 4.01169700 |
| H | -4.75087200 | -0.43061800 | 5.38399000 |
| H | -5.39996300 | 0.48348800 | 4.01962500 |
| H | -5.27148500 | -1.27305900 | 3.92259700 |

**Ni[P(n-Bu)$_3$](PyrCO)[Na$_2$CO$_3$OPh], iso1**

| | | | |
|---|---|---|---|
| Ni | 0.66633900 | 1.31838800 | -0.39284600 |
| P | -0.76788200 | -0.31610900 | -0.27301300 |
| C | -0.09934400 | 2.31323400 | 0.97255800 |
| O | 2.17745800 | 2.48078500 | -0.81008800 |
| C | 2.60887600 | 1.61456600 | -1.70376400 |
| O | 1.80179300 | 0.60371600 | -1.90695300 |
| O | 3.74589200 | 1.70608100 | -2.23161700 |
| Na | 3.79365100 | -0.67343000 | -1.8780490 |
| Na | 4.36031900 | 2.21667000 | 0.08056200 |
| O | 4.32872900 | -0.02897600 | 0.20815000 |
| C | 3.51836500 | -0.69784800 | 0.98469200 |
| C | 3.56320900 | -2.11800100 | 1.08093900 |
| C | 2.68862700 | -2.82655500 | 1.90092300 |
| C | 1.72093500 | -2.16753900 | 2.66089900 |
| C | 2.51871400 | -0.05463600 | 1.76847300 |
| C | 1.65399400 | -0.77277400 | 2.58380300 |
| C | -1.66381400 | -0.59323300 | 1.31086700 |
| C | -2.05621000 | -0.21861600 | -1.59255500 |
| C | 0.19031900 | -1.84821700 | -0.62430100 |
| C | -0.34867600 | -3.18721900 | -0.13971000 |
| C | 0.56419500 | -4.33705900 | -0.54619600 |
| C | -2.66156800 | -1.51334300 | -2.12358800 |
| C | -3.69184500 | -1.25748600 | -3.21630600 |
| C | -2.94779900 | -1.41291700 | 1.30396800 |
| C | -3.54905100 | -1.54333000 | 2.69764600 |
| C | -4.83529000 | -2.34915300 | 2.70865100 |
| C | 0.18314400 | -5.65159600 | 0.10910600 |
| C | -4.28213300 | -2.53834000 | -3.77791800 |
| C | -1.37558300 | 3.04841900 | 0.68244400 |

| | | | |
|---|---|---|---|
| O | 0.41752700 | 2.42646200 | 2.07915800 |
| H | 4.32696000 | -2.64680800 | 0.50913500 |
| H | 2.76518600 | -3.91244900 | 1.94741700 |
| H | 1.04150100 | -2.72488900 | 3.30139300 |
| H | 2.41604700 | 1.02885700 | 1.71983400 |
| H | 0.91050300 | -0.22532000 | 3.16187600 |
| H | 1.18052200 | -1.67955100 | -0.18009600 |
| H | 0.34720800 | -1.85340900 | -1.71179300 |
| H | -0.43174000 | -3.16345700 | 0.95649000 |
| H | -1.36075700 | -3.37258300 | -0.52351200 |
| H | -1.58183900 | 0.32270800 | -2.42182600 |
| H | -2.84092700 | 0.45346100 | -1.21787100 |
| H | -1.86444100 | -2.15727100 | -2.52181100 |
| H | -3.12939700 | -2.08519700 | -1.31241400 |
| H | -1.87327400 | 0.40342300 | 1.72141700 |
| H | -0.92479100 | -1.03802500 | 1.99088200 |
| H | -3.68581600 | -0.94900800 | 0.63393600 |
| H | -2.76149300 | -2.41592500 | 0.89931800 |
| H | -3.72991900 | -0.54054000 | 3.10787200 |
| H | -2.80845800 | -2.00716000 | 3.36365600 |
| H | -5.25264600 | -2.43786400 | 3.71533600 |
| H | -5.59891100 | -1.88570000 | 2.07466600 |
| H | -4.67069300 | -3.36337900 | 2.32900500 |
| H | 1.59679500 | -4.07340700 | -0.27999200 |
| H | 0.54889300 | -4.43871200 | -1.64059700 |
| H | 0.83373900 | -6.47179500 | -0.20742400 |
| H | 0.25295600 | -5.57652200 | 1.20041400 |
| H | -0.84714900 | -5.93607600 | -0.13133300 |
| H | -3.22558100 | -0.67384800 | -4.02140800 |
| H | -4.49085500 | -0.62080800 | -2.81309100 |
| H | -5.02084200 | -2.33851200 | -4.55878400 |
| H | -3.50469500 | -3.17482400 | -4.21412500 |
| H | -4.77862300 | -3.12234200 | -2.99551200 |
| C | -1.90414000 | 3.10602600 | -0.60945800 |
| N | -3.04476600 | 3.72264400 | -0.93254600 |
| C | -3.70784300 | 4.31773100 | 0.06751800 |
| C | -2.08159400 | 3.69148100 | 1.70343900 |
| C | -3.27075200 | 4.33433000 | 1.39206600 |
| H | -1.35720400 | 2.62221200 | -1.42305600 |
| H | -4.64064600 | 4.80959700 | -0.20611500 |
| H | -1.68240800 | 3.66575300 | 2.71466400 |
| H | -3.85635600 | 4.83987200 | 2.15416300 |

**Ni[P(n-Bu)$_3$](PyrCO)[Na$_2$CO$_3$OPh], iso2**

| | | | |
|---|---|---|---|
| Ni | 0.90631000 | 0.79214700 | -1.43494800 |
| P | -1.12153200 | 0.62069400 | -0.69538700 |
| C | 1.13545600 | 2.38280800 | -0.50070300 |
| O | 2.78659500 | 0.51835600 | -1.91131900 |
| C | 2.46771900 | -0.62058300 | -2.48403600 |
| O | 1.18172500 | -0.81641400 | -2.62392500 |
| O | 3.33441800 | -1.49282500 | -2.75954000 |
| Na | 1.78117800 | -3.05146600 | -2.0953900 |
| Na | 4.05824300 | -1.01263400 | -0.4656480 |
| O | 2.29463100 | -2.32799900 | -0.00231900 |
| C | 1.37563800 | -2.47404200 | 0.91261600 |
| C | 0.15759000 | -3.16891400 | 0.66103500 |
| C | -0.81230400 | -3.34518200 | 1.64272400 |
| C | -0.63702300 | -2.82522900 | 2.92543900 |
| C | 1.52262400 | -1.95512400 | 2.23109800 |
| C | 0.54315700 | -2.12754300 | 3.20016000 |
| C | -1.13554100 | 0.45537800 | 1.13660000 |
| C | -2.23098700 | 2.02998200 | -1.10992800 |
| C | -1.91166900 | -0.91017100 | -1.34645100 |
| C | -3.00376600 | -1.57543100 | -0.51877000 |
| C | -3.40597400 | -2.92920300 | -1.08834000 |
| C | -3.73269900 | 1.77263700 | -1.14896400 |
| C | -4.52155300 | 3.02687400 | -1.50239200 |
| C | -2.40931800 | 0.79884800 | 1.90041300 |
| C | -2.24224600 | 0.59146600 | 3.40000000 |
| C | -3.48289700 | 0.96833100 | 4.18887200 |
| C | -4.49760100 | -3.60609100 | -0.27929200 |
| C | -6.01738600 | 2.77887100 | -1.57555300 |
| O | 0.62115200 | 3.45949600 | -0.77900100 |
| C | 2.19636200 | 2.30579700 | 0.55673600 |
| H | -0.01708400 | -3.59403000 | -0.32927900 |
| H | -1.71843100 | -3.89909500 | 1.39821700 |
| H | -1.39435600 | -2.96402100 | 3.69150600 |
| H | 2.43636900 | -1.41125900 | 2.46742300 |
| H | 0.70000900 | -1.70724400 | 4.19263200 |
| H | -1.07748700 | -1.61125100 | -1.47674700 |
| H | -2.26177800 | -0.68174700 | -2.36211700 |
| H | -2.64368800 | -1.72384400 | 0.50883000 |
| H | -3.88735300 | -0.92707000 | -0.44594700 |
| H | -1.89240400 | 2.40657600 | -2.08268700 |
| H | -1.98575800 | 2.82962700 | -0.40005700 |
| H | -3.95652700 | 0.98672100 | -1.88441600 |
| H | -4.08520300 | 1.38583200 | -0.18383600 |
| H | -0.31502300 | 1.08639400 | 1.50374700 |
| H | -0.81797300 | -0.57607300 | 1.34668200 |
| H | -2.69284300 | 1.84316100 | 1.70358300 |
| H | -3.24960000 | 0.18864500 | 1.54437900 |
| H | -1.38007700 | 1.17629300 | 3.74981800 |
| H | -1.97778600 | -0.45920800 | 3.58048900 |
| H | -3.34741600 | 0.81037900 | 5.26246900 |
| H | -3.74355100 | 2.02212300 | 4.04101100 |
| H | -4.34803600 | 0.37391700 | 3.87536900 |
| H | -2.51358800 | -3.57158300 | -1.12519100 |
| H | -3.72995100 | -2.80884600 | -2.13128100 |
| H | -4.75873700 | -4.58742700 | -0.68512000 |
| H | -4.18651500 | -3.75027300 | 0.76158900 |
| H | -5.41080400 | -3.00182700 | -0.26232400 |
| H | -4.15875000 | 3.42226100 | -2.46056700 |
| H | -4.30587500 | 3.80646900 | -0.75936800 |
| H | -6.56976000 | 3.68802200 | -1.82826700 |
| H | -6.25754600 | 2.02580700 | -2.33392400 |
| H | -6.40599000 | 2.41175300 | -0.61956500 |
| C | 2.61107900 | 1.06925400 | 1.05561100 |
| N | 3.63059100 | 0.89956500 | 1.90356200 |
| C | 4.26064800 | 2.01122800 | 2.31511200 |
| C | 2.84947900 | 3.44800400 | 1.02278900 |
| C | 3.89595300 | 3.29790200 | 1.92414900 |
| H | 2.08430400 | 0.16406900 | 0.73477300 |
| H | 5.09881600 | 1.86071300 | 2.99363600 |
| H | 2.53450200 | 4.42246200 | 0.65781600 |
| H | 4.43781900 | 4.15708200 | 2.30785800 |

**TS(decarb.)**
**Ni[P(n-Bu)$_3$](PyrCO)[Na$_2$CO$_3$OPh],**

| | | | |
|---|---|---|---|
| Ni | -0.06053300 | 0.93686500 | -0.21398500 |
| P | -2.19790500 | 0.39364300 | -0.03003500 |
| C | 0.07131100 | 2.44183700 | 0.56074900 |
| C | 1.67421600 | 1.94271700 | -0.12243900 |
| O | 0.43762000 | -0.26168700 | -1.76915300 |
| C | 0.56815900 | -1.35512800 | -1.05700100 |

| | | | |
|---|---|---|---|
| O | 0.28886400 | -1.26005300 | 0.21159900 |
| O | 1.02974100 | -2.41763600 | -1.57609200 |
| Na | 1.94030600 | -2.87838000 | 0.47121200 |
| Na | 2.82653300 | -0.89331800 | -2.1952120 |
| O | 3.71238600 | -1.76272300 | -0.32820100 |
| C | 4.77831600 | -1.30221900 | 0.26282800 |
| C | 4.89858600 | -1.25458600 | 1.67973200 |
| C | 6.00631300 | -0.69498800 | 2.30438000 |
| C | 7.06142800 | -0.16272200 | 1.55884700 |
| C | 5.88381000 | -0.78484100 | -0.46762700 |
| C | 6.98559500 | -0.22427500 | 0.16524200 |
| C | -2.63732400 | -0.93105100 | -1.22243500 |
| C | -2.45371100 | -0.28180900 | 1.66023100 |
| C | -3.42163300 | 1.75156500 | -0.24468300 |
| C | -4.79373700 | 1.60238000 | 0.40490500 |
| C | -5.70166700 | 2.79029000 | 0.11417700 |
| C | -4.10409400 | -1.17806800 | -1.55056700 |
| C | -4.27860200 | -2.29879800 | -2.56798700 |
| C | -5.73362100 | -2.55261100 | -2.91824700 |
| C | -7.06040100 | 2.66661500 | 0.77974100 |
| C | 2.06425800 | 2.49409100 | -1.34919000 |
| N | 3.33919000 | 2.65083400 | -1.72220100 |
| C | 4.27862800 | 2.29465100 | -0.83221800 |
| C | 2.67250700 | 1.57839900 | 0.78508400 |
| C | 4.00232800 | 1.77028000 | 0.42811400 |
| C | -3.57693700 | -1.28748800 | 1.88602400 |
| C | -3.63679100 | -1.75848900 | 3.33349700 |
| C | -4.72473100 | -2.78959600 | 3.57347200 |
| H | 4.07021200 | -1.63821400 | 2.27751400 |
| H | 6.04565000 | -0.66454500 | 3.39223700 |
| H | 7.92372300 | 0.27795900 | 2.05051600 |
| H | 5.83522500 | -0.81271800 | -1.55720600 |
| H | 7.79924900 | 0.17654700 | -0.43790000 |
| H | -3.52173400 | 1.90813700 | -1.32727600 |
| H | -2.93561500 | 2.65837400 | 0.13713400 |
| H | -5.28473700 | 0.68120300 | 0.06580900 |
| H | -4.67663800 | 1.49605700 | 1.49264300 |
| H | -2.15850100 | -1.83885600 | -0.83221300 |
| H | -2.08122600 | -0.68996400 | -2.13621400 |
| H | -4.67040200 | -1.42599800 | -0.64303800 |
| H | -4.55850400 | -0.25823200 | -1.94634700 |
| H | -3.82235700 | -3.21600700 | -2.17174000 |
| H | -3.70992700 | -2.05310900 | -3.47458000 |
| H | -5.84199500 | -3.35877900 | -3.64885300 |
| H | -6.31247900 | -2.83152300 | -2.03119700 |
| H | -6.20146500 | -1.65768000 | -3.34272000 |
| H | -5.82411300 | 2.89005400 | -0.97288600 |
| H | -5.20453500 | 3.71272700 | 0.44310200 |
| H | -7.70083700 | 3.52487000 | 0.55971900 |
| H | -7.58669600 | 1.76710200 | 0.44259900 |
| H | -6.96363600 | 2.59756600 | 1.86849400 |
| H | 1.30849800 | 2.80182800 | -2.07153600 |
| H | 5.31205100 | 2.42774600 | -1.14976500 |
| H | 2.41531100 | 1.14182000 | 1.74799500 |
| H | 4.81487200 | 1.48231200 | 1.09036100 |
| H | -2.56687300 | 0.58442200 | 2.32625300 |
| H | -1.49295700 | -0.74222500 | 1.91633100 |
| H | -4.54849800 | -0.85992300 | 1.60534300 |
| H | -3.42925800 | -2.15582500 | 1.22827200 |
| H | -3.79341000 | -0.88924400 | 3.98671600 |
| H | -2.65912800 | -2.17059600 | 3.61803700 |
| H | -4.75609400 | -3.11259700 | 4.61754500 |
| H | -5.71286500 | -2.38946900 | 3.32199600 |
| H | -4.56875400 | -3.68109600 | 2.95661600 |
| O | -0.09621600 | 3.48421400 | 1.09584200 |

**Ni[P(n-Bu)$_3$](Pyr)(CO)[Na$_2$CO$_3$OPh], trans O**

| | | | |
|---|---|---|---|
| Ni | -0.00149000 | 1.17534000 | -0.15513800 |
| P | -2.19324400 | 0.44198300 | -0.00451900 |
| C | -0.22265200 | 2.45048700 | 0.98328900 |
| C | 1.82632100 | 1.79891400 | -0.31209900 |
| O | 0.38099500 | 0.02841800 | -1.67203900 |
| C | 0.51767300 | -1.15638900 | -1.09400200 |
| O | 0.26454600 | -1.23584300 | 0.16583100 |
| O | 0.95868400 | -2.12324400 | -1.78845600 |
| Na | 1.90084000 | -2.90358800 | 0.15991300 |
| Na | 2.79122600 | -0.62035100 | -2.2344360 |
| O | 3.69596300 | -1.74134100 | -0.52301900 |
| C | 4.70107800 | -1.36322200 | 0.21787200 |
| C | 4.61417500 | -1.30595000 | 1.63693800 |
| C | 5.66711200 | -0.85016800 | 2.42057300 |
| C | 6.86879900 | -0.43722500 | 1.84007000 |
| C | 5.94382500 | -0.95860500 | -0.34126600 |
| C | 6.99120000 | -0.50509700 | 0.44992800 |
| C | -2.57307500 | -0.83454400 | -1.26755200 |
| C | -2.40350300 | -0.33218100 | 1.64868300 |
| C | -3.50118600 | 1.73425200 | -0.14143200 |
| C | -4.86049400 | 1.46876300 | 0.49782900 |
| C | -5.83575800 | 2.61662100 | 0.27265300 |
| C | -4.02832200 | -1.10452200 | -1.62750100 |
| C | -4.15563800 | -2.16858600 | -2.71056300 |
| C | -5.59769700 | -2.44280900 | -3.09716500 |
| C | -7.18364100 | 2.37855400 | 0.92946300 |
| C | 2.22261400 | 2.43518500 | -1.49227400 |
| N | 3.49680500 | 2.73951400 | -1.78925600 |
| C | 4.42929400 | 2.42395500 | -0.87796200 |
| C | 2.81490200 | 1.48897300 | 0.61894400 |
| C | 4.14393800 | 1.80201900 | 0.33236600 |
| C | -3.46457400 | -1.41507900 | 1.80665500 |
| C | -3.48063100 | -2.00467800 | 3.21121400 |
| C | -4.51620500 | -3.10245300 | 3.37488200 |
| H | 3.67049700 | -1.59208000 | 2.10602500 |
| H | 5.54768600 | -0.80884400 | 3.50233800 |
| H | 7.69057900 | -0.08173400 | 2.45492000 |
| H | 6.05144500 | -0.99556600 | -1.42537700 |
| H | 7.92062200 | -0.19372700 | -0.02510900 |
| H | -3.61470600 | 1.94538800 | -1.21344400 |
| H | -3.07642000 | 2.65038200 | 0.28865100 |
| H | -5.29815100 | 0.54177700 | 0.10664200 |
| H | -4.73360400 | 1.30692300 | 1.57766500 |
| H | -2.08033100 | -1.74829300 | -0.91278100 |
| H | -2.00955100 | -0.53587100 | -2.15987100 |
| H | -4.59321900 | -1.42275800 | -0.74108000 |
| H | -4.50933400 | -0.17857600 | -1.97399600 |
| H | -3.67774400 | -3.09379200 | -2.36145800 |
| H | -3.58233400 | -1.85398800 | -3.59256800 |
| H | -5.67215500 | -3.21020500 | -3.87228600 |
| H | -6.18097800 | -2.78603100 | -2.23595000 |
| H | -6.08460400 | -1.53919200 | -3.47975500 |
| H | -5.96679600 | 2.76791100 | -0.80748200 |
| H | -5.39200100 | 3.54716800 | 0.65132100 |
| H | -7.87405300 | 3.20791200 | 0.75421900 |
| H | -7.65643400 | 1.46855100 | 0.54475700 |
| H | -7.08085500 | 2.25794200 | 2.01315400 |

| | | | |
|---|---|---|---|
| H | 1.48644000 | 2.69101400 | -2.25483200 |
| H | 5.45768300 | 2.67135200 | -1.13954100 |
| H | 2.57118300 | 0.97746000 | 1.54876800 |
| H | 4.94321700 | 1.53348700 | 1.01995400 |
| H | -2.57444600 | 0.48861800 | 2.35929200 |
| H | -1.41683900 | -0.74650400 | 1.88261600 |
| H | -4.46151400 | -1.02127200 | 1.56791800 |
| H | -3.27729700 | -2.21955400 | 1.08137200 |
| H | -3.66714600 | -1.20137800 | 3.93681200 |
| H | -2.48082500 | -2.39246400 | 3.44881700 |
| H | -4.51514100 | -3.51597900 | 4.38707700 |
| H | -5.52570600 | -2.72893100 | 3.17219400 |
| H | -4.33071900 | -3.92886800 | 2.68037900 |
| O | -0.38290700 | 3.33324700 | 1.71637700 |

**Ni[P(n-Bu)$_3$](Pyr)(CO)[Na$_2$CO$_3$OPh], trans N**

| | | | |
|---|---|---|---|
| Ni | -0.16850300 | -1.50174900 | -0.42224200 |
| C | -0.82495600 | -3.13044700 | 0.11892300 |
| P | -1.95913200 | -0.29571300 | 0.05977600 |
| C | 0.63481500 | 0.12298700 | -1.04079000 |
| O | 1.41175900 | -2.36923100 | -1.14419400 |
| C | 2.12544200 | -2.84296400 | -0.12348800 |
| O | 1.65975900 | -2.74631300 | 1.06288000 |
| O | 3.28648000 | -3.28719100 | -0.40057900 |
| Na | 3.93960500 | -2.42019900 | 1.62395000 |
| Na | 3.52226200 | -1.18555700 | -1.5578500 |
| O | 4.47382200 | -0.63306800 | 0.40724400 |
| C | 4.71245200 | 0.62015700 | 0.67255500 |
| C | 4.26560100 | 1.23934700 | 1.87322500 |
| C | 4.46656000 | 2.59184000 | 2.11883700 |
| C | 5.13183300 | 3.40122000 | 1.19411400 |
| C | 5.40855300 | 1.46352600 | -0.23763700 |
| C | 5.60501100 | 2.81470600 | 0.01789800 |
| C | -1.70827400 | 1.21708500 | 1.07350600 |
| C | -2.69196500 | 0.22869900 | -1.54473500 |
| C | -3.24425000 | -1.27125300 | 0.95234700 |
| C | -4.38447900 | -0.53473400 | 1.64717900 |
| C | -5.36386200 | -1.49695600 | 2.30763400 |
| C | -4.18681300 | 0.51608000 | -1.62326600 |
| C | -4.60883600 | 0.92826600 | -3.02791300 |
| C | -2.68083700 | 2.37595100 | 0.87620200 |
| C | -2.31759000 | 3.57267300 | 1.74608500 |
| C | -3.25677000 | 4.74799200 | 1.54530500 |
| C | -6.48976600 | -0.77981700 | 3.03040200 |
| C | -6.09832300 | 1.19947600 | -3.13640700 |
| C | 0.62247700 | 0.47155000 | -2.39722800 |
| N | 1.30895000 | 1.49701100 | -2.92907600 |
| C | 2.04601800 | 2.23770400 | -2.09037000 |
| C | 1.43652500 | 0.90322000 | -0.20132900 |
| C | 2.14476600 | 1.98554600 | -0.72477200 |
| H | 3.72380400 | 0.62432600 | 2.59319000 |
| H | 4.09369000 | 3.02583300 | 3.04563200 |
| H | 5.28720400 | 4.45814400 | 1.39034000 |
| H | 5.77738500 | 1.02054500 | -1.16342700 |
| H | 6.13366200 | 3.42383300 | -0.71410400 |
| H | -2.69799900 | -1.86517400 | 1.69601600 |
| H | -3.63513200 | -2.00044300 | 0.22908500 |
| H | -3.97631100 | 0.14284500 | 2.40986700 |
| H | -4.92841600 | 0.10223100 | 0.93900500 |
| H | -2.43586900 | -0.56671600 | -2.25648600 |
| H | -2.10906900 | 1.10032700 | -1.86983600 |
| H | -4.75466400 | -0.37547300 | -1.32260700 |
| H | -4.46738300 | 1.30672400 | -0.91549600 |
| H | -0.69208500 | 1.56633000 | 0.86260600 |
| H | -1.70234800 | 0.88853500 | 2.12098400 |
| H | -2.67844200 | 2.68419100 | -0.17853800 |
| H | -3.71003100 | 2.06545200 | 1.09718100 |
| H | -1.28492700 | 3.87480100 | 1.52681600 |
| H | -2.32041200 | 3.26494600 | 2.80039000 |
| H | -2.98301300 | 5.59855700 | 2.17500400 |
| H | -3.24539500 | 5.09077900 | 0.50527600 |
| H | -4.28997300 | 4.47697400 | 1.78708700 |
| H | -4.81832300 | -2.14275300 | 3.00857700 |
| H | -5.77656500 | -2.16927600 | 1.54335400 |
| H | -7.18719600 | -1.48263400 | 3.49358700 |
| H | -6.10266800 | -0.12921900 | 3.82190800 |
| H | -7.06422500 | -0.14948600 | 2.34331800 |
| H | -4.31730300 | 0.14164400 | -3.73670100 |
| H | -4.04177600 | 1.82071200 | -3.32449100 |
| H | -6.38374000 | 1.49417100 | -4.14965800 |
| H | -6.68375600 | 0.31202100 | -2.87364200 |
| H | -6.40555200 | 2.00417700 | -2.45989200 |
| H | 0.03589700 | -0.11285000 | -3.10881700 |
| H | 2.59350200 | 3.06754900 | -2.53718000 |
| H | 1.52605700 | 0.67025300 | 0.86050200 |
| H | 2.77877200 | 2.59971300 | -0.08940800 |
| O | -1.18875900 | -4.17092000 | 0.44908200 |

**Ni[P(nBu)$_3$](Pyr)(CO)[Na$_2$CO$_3$OPhB(OH)$_2$] (PhOMe), CO-P trans, iso2**

| | | | |
|---|---|---|---|
| Ni | 0.55085900 | 1.43910200 | -1.04966200 |
| P | 0.00481200 | 0.51913200 | 0.97292500 |
| C | -1.36786500 | 1.78174600 | -1.34584100 |
| O | -0.30207900 | -2.24520600 | -2.13575400 |
| C | 0.45394500 | -3.02018300 | -1.50320700 |
| O | 1.80976700 | -2.76824300 | -1.73374400 |
| O | 0.12639800 | -3.93767300 | -0.72006900 |
| Na | -2.17891400 | -3.31405900 | -1.0308070 |
| C | -1.02301200 | -0.99319200 | 1.06526000 |
| C | 1.40551900 | 0.14982700 | 2.10688500 |
| C | -0.95711500 | 1.80128000 | 1.86363900 |
| C | -0.26253100 | 3.15750900 | 1.82435900 |
| C | -1.45905700 | -1.39077900 | 2.47380600 |
| C | -1.80364100 | 3.10974700 | -1.21940400 |
| N | -3.08021000 | 3.51519100 | -1.28163700 |
| C | -4.00147900 | 2.57269200 | -1.51771600 |
| C | -2.35885100 | 0.83378600 | -1.62150500 |
| C | -3.69258600 | 1.23023400 | -1.71137800 |
| C | 2.07975700 | -1.19509800 | 1.85426200 |
| B | 2.84685000 | -3.47226300 | -1.09591300 |
| C | 2.48764400 | 1.37254300 | -0.65187600 |
| O | 4.08722200 | -3.03292200 | -1.45974900 |
| O | 2.65084300 | -4.47213100 | -0.21269000 |
| C | 3.02321000 | 2.53437500 | -0.06722100 |
| C | 4.34204600 | 2.60312600 | 0.36573900 |
| C | 5.19187400 | 1.50245800 | 0.20366500 |
| C | 3.36963800 | 0.30423700 | -0.82918200 |
| C | 4.70484000 | 0.34814400 | -0.40976000 |
| O | 6.46368800 | 1.65435900 | 0.67464300 |
| C | 7.32236500 | 0.53884000 | 0.55537900 |
| O | -3.81925100 | -2.15023700 | -0.43876800 |
| H | -1.13660700 | 1.47824500 | 2.89671000 |
| H | -1.93587600 | 1.85863600 | 1.36800700 |
| H | 0.65458900 | 3.12778400 | 2.43067600 |

| | | | |
|---|---|---|---|
| H | 0.07368700 | 3.35754700 | 0.79434000 |
| H | -0.42622600 | -1.80124400 | 0.62320100 |
| H | -1.90700800 | -0.87468300 | 0.42295200 |
| H | -0.60165800 | -1.39918400 | 3.16271300 |
| H | -2.15406600 | -0.63840000 | 2.87121300 |
| H | -1.07855600 | 3.90932600 | -1.04178800 |
| H | -5.03784200 | 2.90841200 | -1.55369000 |
| H | -2.11320600 | -0.22165100 | -1.73885400 |
| H | -4.48195400 | 0.50562500 | -1.89191700 |
| H | 1.04822000 | 0.21349200 | 3.14326900 |
| H | 2.13684800 | 0.95572900 | 1.97172500 |
| H | 1.41490200 | -2.01885700 | 2.15554900 |
| H | 2.24926200 | -1.32791100 | 0.77553300 |
| H | 2.39206900 | 3.41278600 | 0.07600700 |
| H | 4.73845800 | 3.50173400 | 0.83148400 |
| H | 3.03520300 | -0.63951500 | -1.26002700 |
| H | 5.33182700 | -0.52721300 | -0.55266500 |
| H | 4.77092100 | -3.54478900 | -1.01831400 |
| H | 8.27453000 | 0.83674300 | 0.99388400 |
| H | 6.93216500 | -0.33327300 | 1.09764800 |
| H | 7.48032400 | 0.25594800 | -0.49339100 |
| H | 1.68503000 | -4.59740900 | -0.12073900 |
| C | -4.84642300 | -1.42448500 | -0.13067300 |
| C | -6.03334400 | -1.41419100 | -0.91958000 |
| C | -7.10793900 | -0.58637200 | -0.61871200 |
| C | -7.07671600 | 0.26624700 | 0.48754200 |
| C | -4.85616400 | -0.56293500 | 1.00503500 |
| C | -5.93777500 | 0.25782400 | 1.29634100 |
| H | -6.06823800 | -2.06618900 | -1.79174200 |
| H | -7.98831800 | -0.60382600 | -1.26036300 |
| H | -7.91915800 | 0.91241700 | 0.71756500 |
| H | -3.96985300 | -0.54661300 | 1.63944600 |
| H | -5.89178400 | 0.90892800 | 2.16891600 |
| Na | 1.23943100 | -0.87999000 | -3.1233190 |
| C | 3.41199400 | -1.31082300 | 2.58426000 |
| C | 4.17085700 | -2.57993600 | 2.24663800 |
| H | 3.23779200 | -1.24727200 | 3.66695000 |
| H | 4.02515400 | -0.43561300 | 2.32633400 |
| H | 5.11289700 | -2.64744900 | 2.79848200 |
| H | 3.58541600 | -3.47731700 | 2.47079700 |
| H | 4.40700000 | -2.61263400 | 1.17708300 |
| C | -1.15357900 | 4.30236500 | 2.28020100 |
| C | -0.45719700 | 5.64824100 | 2.19767800 |
| H | -1.49435900 | 4.11311800 | 3.30701800 |
| H | -2.05887100 | 4.31019300 | 1.65799200 |
| H | -1.10788300 | 6.46507500 | 2.52077800 |
| H | 0.44112700 | 5.67251300 | 2.82416100 |
| H | -0.14348200 | 5.86488800 | 1.17000100 |
| C | -2.13542400 | -2.75590900 | 2.48264000 |
| C | -2.70187400 | -3.12253900 | 3.84167900 |
| H | -1.40350600 | -3.51248400 | 2.15977500 |
| H | -2.93132500 | -2.74956500 | 1.72479900 |
| H | -3.16197100 | -4.11461700 | 3.83605200 |
| H | -1.92642500 | -3.12209600 | 4.61612800 |
| H | -3.47175300 | -2.40602900 | 4.14898600 |
| C | 0.92548400 | 2.23180900 | -2.56280500 |
| O | 1.18443200 | 2.77216000 | -3.55343200 |

**[$Na_2CO_3OPhB(OH)_2$]**

| | | | |
|---|---|---|---|
| O | -2.39246100 | -0.26204400 | 0.67775600 |
| C | -1.50997100 | -1.21952500 | 0.20138700 |
| O | -1.34593900 | -2.20660200 | 0.92593600 |
| O | -0.93315600 | -0.99255000 | -0.91648900 |
| Na | 0.26086000 | 0.99965300 | -1.13482800 |
| B | -2.82692500 | 0.83189700 | -0.05429600 |
| O | -3.57903800 | 1.73216500 | 0.63445800 |
| O | -2.51886700 | 1.02642500 | -1.37788500 |
| H | -3.87965900 | 2.43850800 | 0.05738200 |
| H | -2.05400000 | 0.21377500 | -1.66198200 |
| Na | 0.49484300 | -2.90842300 | -0.2528160 |
| O | 2.35641900 | -1.74787300 | -0.09430900 |
| C | 2.27510700 | -0.49286300 | 0.15148300 |
| C | 2.96643000 | 0.49355800 | -0.63193300 |
| C | 1.45452300 | 0.04282700 | 1.20437600 |
| C | 2.85161700 | 1.85744200 | -0.37640800 |
| H | 3.61247600 | 0.13835800 | -1.43266900 |
| C | 1.34431200 | 1.40811800 | 1.44084400 |
| H | 0.90683400 | -0.66160900 | 1.82905900 |
| C | 2.02965400 | 2.34210300 | 0.64996700 |
| H | 3.41063900 | 2.56068600 | -0.99253500 |
| H | 0.70707500 | 1.75462000 | 2.25333000 |
| H | 1.95283000 | 3.40650900 | 0.84894600 |

**TS (Transm.), iso2_down**
**Ni[P(n-Bu)$_3$](PyrCO)[$Na_2CO_3OPh$][$B(OH)_2PhOMe$]**

| | | | |
|---|---|---|---|
| Ni | 0.87095300 | 0.24394800 | 0.16107100 |
| P | -1.23162800 | 1.02954900 | 0.13752200 |
| C | 0.96252600 | 0.75765500 | 1.92545100 |
| O | 1.77860800 | -2.29031100 | -0.65470400 |
| C | 1.29364100 | -1.72858800 | -1.80976900 |
| O | 0.73449300 | -0.60155500 | -1.72433200 |
| O | 1.39886500 | -2.41276000 | -2.85709200 |
| Na | 3.15657500 | -3.70461600 | -2.1894910 |
| C | -2.51525300 | 0.29169600 | 1.22578900 |
| C | -1.13206600 | 2.81699400 | 0.58245200 |
| C | -1.93792400 | 0.92163500 | -1.55487100 |
| C | -3.43141500 | 1.13664900 | -1.77899700 |
| C | -3.81532400 | 0.82825200 | -3.22097800 |
| C | -2.13847800 | 3.77809300 | -0.03373800 |
| C | -1.92016400 | 5.21310500 | 0.42743900 |
| C | -3.69792600 | 1.15467800 | 1.65238700 |
| C | -4.70721700 | 0.35746100 | 2.46908800 |
| C | -5.88364800 | 1.19613800 | 2.93393000 |
| C | -5.30263300 | 0.98767900 | -3.47902100 |
| C | -2.89738500 | 6.18920400 | -0.20258400 |
| O | 1.13837800 | 1.86415700 | 2.41569200 |
| C | 0.89120400 | -0.45062400 | 2.81766800 |
| B | 2.97930500 | -1.80494600 | -0.04778400 |
| C | 2.88323300 | 0.42222600 | 0.00816100 |
| O | 3.20795200 | -2.16747000 | 1.26315200 |
| O | 4.06678400 | -1.91451500 | -0.94449200 |
| C | 3.82493800 | 0.69141100 | 1.03047700 |
| C | 4.96589300 | 1.45132100 | 0.83210700 |
| C | 5.21024900 | 2.02760600 | -0.42136400 |
| C | 3.16818600 | 1.03787600 | -1.23012700 |
| C | 4.29837000 | 1.82265400 | -1.46038400 |
| O | 6.35525300 | 2.75759200 | -0.52761000 |
| C | 6.60944600 | 3.37625800 | -1.77354900 |
| Na | -0.47407200 | -1.29426400 | -3.7637860 |
| O | -2.19781000 | -2.18617700 | -2.82918900 |
| C | -2.66285800 | -2.45922700 | -1.65263300 |
| C | -1.83678900 | -2.93523600 | -0.58763600 |
| C | -4.04428300 | -2.30221800 | -1.32539900 |

| | | | |
|---|---|---|---|
| C | -2.35291800 | -3.26481500 | 0.66164900 |
| C | -4.54605500 | -2.62539400 | -0.07038700 |
| C | -3.71321000 | -3.11465800 | 0.94114300 |
| H | -1.70219400 | -0.10071700 | -1.87032800 |
| H | -1.33822700 | 1.58978000 | -2.18987700 |
| H | -3.99327900 | 0.45689000 | -1.12393400 |
| H | -3.73986700 | 2.15779600 | -1.51744300 |
| H | -0.11082100 | 3.13170500 | 0.33368500 |
| H | -1.17059500 | 2.86305500 | 1.67758500 |
| H | -2.07087500 | 3.73695700 | -1.13033700 |
| H | -3.16292800 | 3.47072700 | 0.21284600 |
| H | -2.00204800 | -0.08222200 | 2.12139800 |
| H | -2.86143900 | -0.60528000 | 0.69367900 |
| H | -3.34533600 | 2.01488400 | 2.24021800 |
| H | -4.20518400 | 1.57939800 | 0.77630200 |
| H | -4.19818900 | -0.09172800 | 3.33340700 |
| H | -5.05670900 | -0.48956600 | 1.86305000 |
| H | -6.59982500 | 0.60783600 | 3.51451500 |
| H | -5.55433800 | 2.03004300 | 3.56362900 |
| H | -6.42392000 | 1.62645400 | 2.08346700 |
| H | -3.49734600 | -0.20143900 | -3.43853600 |
| H | -3.24588000 | 1.48458000 | -3.89647000 |
| H | -5.56595600 | 0.75675600 | -4.51541000 |
| H | -5.88510800 | 0.31891000 | -2.83526100 |
| H | -5.63916800 | 2.01016600 | -3.27282500 |
| H | -0.88877900 | 5.51262600 | 0.19696800 |
| H | -2.00205500 | 5.25407100 | 1.52205200 |
| H | -2.72972500 | 7.21380300 | 0.14098900 |
| H | -2.80942600 | 6.18905800 | -1.29451700 |
| H | -3.93281200 | 5.92577100 | 0.03883700 |
| H | 3.67488300 | 0.24931500 | 2.01525300 |
| H | 5.68321600 | 1.62407500 | 1.63055900 |
| H | 2.48739400 | 0.88449200 | -2.06597400 |
| H | 4.46055100 | 2.26491000 | -2.43892500 |
| H | 7.54500000 | 3.92332900 | -1.65724100 |
| H | 6.72009900 | 2.63862300 | -2.57876300 |
| H | 5.81145700 | 4.07757200 | -2.04772600 |
| H | 4.87027200 | -1.55264300 | -0.56160000 |
| H | -0.77429200 | -3.07293500 | -0.78846200 |
| H | -4.70935400 | -1.93528400 | -2.10730600 |
| H | -1.67825300 | -3.63118600 | 1.43620100 |
| H | -5.61076300 | -2.49521800 | 0.12516000 |
| H | -4.11308100 | -3.36167100 | 1.92109500 |
| H | 2.38949300 | -2.15869100 | 1.76886300 |
| C | 0.22593800 | -1.62561600 | 2.44072000 |
| N | 0.20660800 | -2.74238400 | 3.17869700 |
| C | 0.87236000 | -2.71436400 | 4.34198900 |
| C | 1.55462700 | -0.44153000 | 4.04831800 |
| C | 1.55321800 | -1.59594400 | 4.82022400 |
| H | -0.31638100 | -1.66299800 | 1.49035700 |
| H | 0.86134500 | -3.63768100 | 4.91927900 |
| H | 2.06640300 | 0.46489200 | 4.36263700 |
| H | 2.07155900 | -1.63787600 | 5.77343900 |

**TS (Transm.), iso2_up**
**Ni[P(n-Bu)$_3$](PyrCO)[$Na_2CO_3$OPh][B(OH)$_2$PhOMe]**

| | | | |
|---|---|---|---|
| Ni | 1.04268400 | 0.05328600 | 0.09432500 |
| P | -0.97856400 | 1.01551200 | -0.07978200 |
| C | 1.57587300 | 1.51289300 | 1.07525500 |
| O | 0.62133600 | -2.35803700 | 1.21763300 |
| C | 0.47196800 | -2.62558400 | -0.11042800 |
| O | 0.40322100 | -1.63249900 | -0.89496800 |
| O | 0.35641000 | -3.82673400 | -0.45336200 |
| Na | 1.31170200 | -4.71884100 | 1.45639700 |
| C | -1.82917100 | 0.55229200 | 1.47889900 |
| C | -0.96671400 | 2.85782800 | -0.14724900 |
| C | -2.00464600 | 0.43289500 | -1.48551400 |
| C | -3.52109800 | 0.60252900 | -1.42939200 |
| C | -4.15926600 | 0.27054100 | -2.77196500 |
| C | -2.20197800 | 3.63190400 | -0.58836200 |
| C | -1.96625500 | 5.13609600 | -0.52304000 |
| C | -3.06978300 | 1.31526500 | 1.93179300 |
| C | -3.62364500 | 0.75648600 | 3.23605700 |
| C | -4.92257400 | 1.41945800 | 3.65650000 |
| C | -5.66011800 | 0.49531300 | -2.77484300 |
| C | -3.16265900 | 5.94256500 | -0.99404800 |
| C | 2.24465800 | 2.64862700 | 0.37588800 |
| O | 1.36822700 | 1.58480800 | 2.29319600 |
| B | 1.88344700 | -1.88595000 | 1.76486100 |
| C | 2.92263100 | -0.68079000 | 0.26309400 |
| O | 1.85141400 | -1.10054900 | 2.89120000 |
| O | 2.78288900 | -2.98830500 | 1.78012800 |
| C | 3.97438900 | -0.13758700 | 1.04291900 |
| C | 5.30792000 | -0.22272800 | 0.68194000 |
| C | 5.66612200 | -0.84324300 | -0.52259900 |
| C | 3.33324200 | -1.28493200 | -0.94438200 |
| C | 4.66730800 | -1.37498600 | -1.34220600 |
| O | 6.99660400 | -0.87900300 | -0.80080600 |
| C | 7.38411700 | -1.48113700 | -2.02172800 |
| Na | -0.73971300 | -3.14747100 | -2.4683440 |
| O | -2.87880300 | -2.95806700 | -2.22274200 |
| C | -3.62697700 | -2.77544100 | -1.18338200 |
| C | -3.10396300 | -2.73656500 | 0.14532200 |
| C | -5.03811800 | -2.58059100 | -1.28200000 |
| C | -3.90512300 | -2.47684300 | 1.25103200 |
| C | -5.82534100 | -2.30927400 | -0.17065900 |
| C | -5.27440300 | -2.24042300 | 1.11364600 |
| H | -1.77673300 | -0.63326900 | -1.60825700 |
| H | -1.59443600 | 0.93727400 | -2.37292100 |
| H | -3.93155100 | -0.06673900 | -0.66119400 |
| H | -3.79859900 | 1.62253300 | -1.13718700 |
| H | -0.13044400 | 3.12960200 | -0.80465700 |
| H | -0.65416200 | 3.18053800 | 0.85680500 |
| H | -2.46828100 | 3.35165200 | -1.61702700 |
| H | -3.07175500 | 3.37315700 | 0.02911900 |
| H | -1.05200500 | 0.61616500 | 2.25352100 |
| H | -2.06166600 | -0.51543600 | 1.36499000 |
| H | -2.83111800 | 2.38063700 | 2.06335500 |
| H | -3.85076500 | 1.26993500 | 1.16240700 |
| H | -2.86737000 | 0.86269200 | 4.02561100 |
| H | -3.78327600 | -0.32297500 | 3.11362800 |
| H | -5.29700000 | 1.02039300 | 4.60341500 |
| H | -4.79978800 | 2.50123800 | 3.77985600 |
| H | -5.70172300 | 1.26335800 | 2.90204700 |
| H | -3.92520300 | -0.77451600 | -3.01643000 |
| H | -3.68844000 | 0.88692300 | -3.55297100 |
| H | -6.11203900 | 0.21741300 | -3.73205900 |
| H | -6.14059900 | -0.10426400 | -1.99442200 |
| H | -5.90647300 | 1.54665500 | -2.58547000 |
| H | -1.08149300 | 5.38747400 | -1.12487100 |
| H | -1.70893000 | 5.41230600 | 0.50892400 |
| H | -2.97627200 | 7.01866600 | -0.93949600 |
| H | -3.41733000 | 5.70378900 | -2.03206100 |

| | | | |
|---|---|---|---|
| H | -4.04810500 | 5.72956600 | -0.38577300 |
| H | 3.72975400 | 0.33992400 | 1.99060900 |
| H | 6.09504400 | 0.19123400 | 1.30707700 |
| H | 2.58499900 | -1.70790400 | -1.61130400 |
| H | 4.91722300 | -1.85205200 | -2.28515800 |
| H | 8.46981000 | -1.40295100 | -2.06847300 |
| H | 7.09576000 | -2.53894000 | -2.06079200 |
| H | 6.94581200 | -0.96385600 | -2.88386000 |
| H | 3.62652600 | -2.72767800 | 2.15844000 |
| H | -2.03763300 | -2.91366000 | 0.28092400 |
| H | -5.48470800 | -2.61559900 | -2.27529200 |
| H | -3.44769300 | -2.45155300 | 2.24139200 |
| H | -6.89423300 | -2.14455700 | -0.30725700 |
| H | -5.89420100 | -2.02522000 | 1.98022300 |
| H | 1.43126200 | -0.23233600 | 2.76256700 |
| C | 2.73520200 | 2.51049000 | -0.92647700 |
| N | 3.30005600 | 3.49966300 | -1.62412200 |
| C | 3.38610000 | 4.69152200 | -1.01729100 |
| C | 2.36317600 | 3.89798300 | 0.99066900 |
| C | 2.93984000 | 4.94035000 | 0.27952400 |
| H | 2.66665200 | 1.53534900 | -1.41471500 |
| H | 3.83720200 | 5.49179000 | -1.60262300 |
| H | 1.98988700 | 4.02738300 | 2.00378400 |
| H | 3.04322500 | 5.93034000 | 0.71308500 |

**Ni[P(n-Bu)$_3$](PyrCO)[Na$_2$CO$_3$OPhB(OH)$_2$](PhOMe) Iso2_down**

| | | | |
|---|---|---|---|
| Ni | 0.62675000 | 0.84033800 | 0.00795800 |
| P | -1.61231000 | 1.01360700 | -0.16747300 |
| C | 0.55781000 | 1.81692500 | 1.56533800 |
| O | 1.91197800 | -1.77665400 | -0.03748300 |
| C | 1.66177800 | -1.44952400 | -1.34687800 |
| O | 0.77604500 | -0.57121900 | -1.52030600 |
| O | 2.26281500 | -2.06441000 | -2.26118700 |
| Na | 4.37058600 | -1.21257400 | -2.2542050 |
| C | -2.67803800 | 0.25410700 | 1.12685300 |
| C | -2.03004500 | 2.81123600 | -0.18504800 |
| C | -2.20748800 | 0.28728400 | -1.74733100 |
| C | -3.69408200 | 0.02473800 | -1.95272800 |
| C | -3.94356500 | -0.74186700 | -3.24507900 |
| C | -3.24104700 | 3.28896800 | -0.97469800 |
| C | -3.40407100 | 4.80224800 | -0.90680200 |
| C | -4.05693800 | 0.85296800 | 1.38063700 |
| C | -4.81387300 | 0.09346200 | 2.46226500 |
| C | -6.18137500 | 0.68481600 | 2.75210800 |
| C | -5.40504100 | -1.10295200 | -3.43878800 |
| C | -4.59431800 | 5.30558500 | -1.70316200 |
| O | 0.42809800 | 3.01607700 | 1.77671300 |
| C | 0.78229000 | 0.86553500 | 2.71426600 |
| B | 3.08747100 | -2.25206100 | 0.50641500 |
| C | 2.57205600 | 1.13126900 | -0.10403400 |
| O | 3.12816700 | -2.66195700 | 1.79159500 |
| O | 4.25233800 | -2.28734800 | -0.24017300 |
| C | 3.56021500 | 0.92272900 | 0.87989900 |
| C | 4.92282800 | 1.06642700 | 0.63498500 |
| C | 5.37109100 | 1.45694900 | -0.63103400 |
| C | 3.06593400 | 1.50456900 | -1.36636000 |
| C | 4.43333800 | 1.67698800 | -1.64473000 |
| O | 6.72732800 | 1.59307000 | -0.77918900 |
| C | 7.18268800 | 2.08692800 | -2.02027100 |
| Na | -0.02224800 | -2.01992000 | -3.3053000 |
| O | -1.46348600 | -3.01217400 | -2.08026300 |
| C | -2.00270300 | -3.10640600 | -0.90609000 |
| C | -1.23356200 | -3.12829500 | 0.29766600 |
| C | -3.41579300 | -3.19711200 | -0.72485500 |
| C | -1.82351200 | -3.25225800 | 1.55086300 |
| C | -3.99391200 | -3.30914700 | 0.53414100 |
| C | -3.21028200 | -3.34242800 | 1.69177300 |
| H | -1.68969600 | -0.67812400 | -1.80024100 |
| H | -1.79601700 | 0.90710700 | -2.55763400 |
| H | -4.06497000 | -0.58569500 | -1.11866000 |
| H | -4.27474000 | 0.95640300 | -1.94657900 |
| H | -1.12742400 | 3.32040800 | -0.54553600 |
| H | -2.10995400 | 3.11579200 | 0.86615200 |
| H | -3.14676500 | 2.98202700 | -2.02653300 |
| H | -4.15928300 | 2.81186500 | -0.60645500 |
| H | -2.09953700 | 0.27177200 | 2.06104800 |
| H | -2.75898800 | -0.80844400 | 0.85681300 |
| H | -3.96077600 | 1.90944300 | 1.67194300 |
| H | -4.65424900 | 0.85197900 | 0.45906300 |
| H | -4.20919000 | 0.07495900 | 3.37977200 |
| H | -4.90879000 | -0.95605400 | 2.15237000 |
| H | -6.71228800 | 0.12609200 | 3.52809300 |
| H | -6.10267500 | 1.72418800 | 3.09017200 |
| H | -6.81171000 | 0.68322100 | 1.85606000 |
| H | -3.32633000 | -1.65207100 | -3.21833100 |
| H | -3.58696700 | -0.14671400 | -4.09923900 |
| H | -5.57053200 | -1.66956100 | -4.35995100 |
| H | -5.76765100 | -1.71537000 | -2.60494600 |
| H | -6.03732700 | -0.20902800 | -3.48254300 |
| H | -2.48218700 | 5.27972900 | -1.26599700 |
| H | -3.50169900 | 5.10375400 | 0.14505300 |
| H | -4.69711600 | 6.39257100 | -1.64147300 |
| H | -4.50259800 | 5.04250800 | -2.76273300 |
| H | -5.52918400 | 4.86495000 | -1.34013500 |
| H | 3.26547400 | 0.62059800 | 1.88687600 |
| H | 5.66052800 | 0.89579400 | 1.41593700 |
| H | 2.36589200 | 1.67919400 | -2.18500300 |
| H | 4.74692000 | 2.01034400 | -2.63157600 |
| H | 8.26567100 | 2.17690200 | -1.93839600 |
| H | 6.94308600 | 1.40380300 | -2.84820500 |
| H | 6.75318600 | 3.07042400 | -2.24990000 |
| H | 4.98240700 | -2.54562200 | 0.33074000 |
| H | -0.14990900 | -3.06591400 | 0.21641900 |
| H | -4.04137900 | -3.18898900 | -1.61693700 |
| H | -1.18614800 | -3.26712600 | 2.43493200 |
| H | -5.07910900 | -3.37521000 | 0.61614300 |
| H | -3.66930200 | -3.43138800 | 2.67277900 |
| H | 2.29841700 | -2.52048200 | 2.28720500 |
| C | 0.67537400 | -0.51740100 | 2.53221400 |
| N | 1.09568600 | -1.42479500 | 3.42077100 |
| C | 1.59334200 | -0.96043300 | 4.57930100 |
| C | 1.26921300 | 1.32179100 | 3.94050700 |
| C | 1.66628400 | 0.39245300 | 4.89627300 |
| H | 0.26217000 | -0.91304800 | 1.60100900 |
| H | 1.95630900 | -1.71220100 | 5.27760600 |
| H | 1.35578700 | 2.39324300 | 4.10449400 |
| H | 2.06158700 | 0.70572700 | 5.85782400 |

**Ni[P(n-Bu)$_3$](PyrCO)[Na$_2$CO$_3$OPhB(OH)$_2$](PhOMe) Iso2_up**

| | | | |
|---|---|---|---|
| Ni | 1.07310700 | 0.47920500 | -0.13383800 |

| | | | |
|---|---|---|---|
| P | -1.14399100 | 0.95227000 | -0.12638900 |
| C | 1.38513000 | 1.91761600 | 0.94016700 |
| O | 0.92025000 | -1.85867500 | 1.13606900 |
| C | 0.97200800 | -2.27446600 | -0.18330400 |
| O | 0.69329200 | -1.38148600 | -1.02652600 |
| O | 1.20570600 | -3.47614900 | -0.43503400 |
| Na | 2.23023400 | -4.65530800 | 1.16021900 |
| C | -1.85356500 | 0.44558000 | 1.49166700 |
| C | -1.51211100 | 2.75463900 | -0.29086200 |
| C | -2.09543400 | 0.08885400 | -1.44154800 |
| C | -3.61283900 | -0.04514100 | -1.35206500 |
| C | -4.18411900 | -0.62747600 | -2.63892500 |
| C | -2.87445100 | 3.23009100 | -0.77696600 |
| C | -2.95245400 | 4.75099300 | -0.82532600 |
| C | -3.21028700 | 0.99046400 | 1.92255400 |
| C | -3.63086200 | 0.46411100 | 3.28925600 |
| C | -5.02655900 | 0.91110100 | 3.68357000 |
| C | -5.69567600 | -0.75510400 | -2.60305700 |
| C | -4.27684300 | 5.25858300 | -1.36608300 |
| C | 1.68695500 | 3.25285400 | 0.35251800 |
| O | 1.29437100 | 1.80885200 | 2.17632000 |
| B | 2.02594500 | -1.67412100 | 1.95453300 |
| C | 3.01487700 | 0.13558300 | -0.18064500 |
| O | 2.05854000 | -0.73083500 | 2.91224600 |
| O | 3.03896900 | -2.62082900 | 1.86865800 |
| C | 3.98718000 | 0.46148700 | 0.78809300 |
| C | 5.31707700 | 0.06731400 | 0.68836000 |
| C | 5.74923800 | -0.66238600 | -0.42279600 |
| C | 3.49854800 | -0.58091300 | -1.29008100 |
| C | 4.83272900 | -0.98075200 | -1.42581700 |
| O | 7.07370700 | -1.00948400 | -0.43787000 |
| C | 7.53436400 | -1.70433400 | -1.57633200 |
| Na | -0.11250000 | -3.10936100 | -2.4990230 |
| O | -2.13359600 | -3.50094100 | -1.90710800 |
| C | -2.92088300 | -3.39624100 | -0.88659100 |
| C | -2.44321600 | -3.10786200 | 0.42739100 |
| C | -4.33628200 | -3.54693200 | -0.99614800 |
| C | -3.30203000 | -2.94102000 | 1.50777600 |
| C | -5.18376500 | -3.36769900 | 0.08889300 |
| C | -4.68596500 | -3.05247100 | 1.35760000 |
| H | -1.66456500 | -0.91906100 | -1.49692600 |
| H | -1.81125500 | 0.59074800 | -2.37876300 |
| H | -3.87039900 | -0.70559200 | -0.51246900 |
| H | -4.09158100 | 0.92061800 | -1.14860900 |
| H | -0.74225200 | 3.15875000 | -0.96112600 |
| H | -1.28430300 | 3.19143900 | 0.69307500 |
| H | -3.06926500 | 2.82536200 | -1.78015100 |
| H | -3.67796700 | 2.84358100 | -0.13660800 |
| H | -1.08793300 | 0.71485900 | 2.23316500 |
| H | -1.87708200 | -0.65199700 | 1.46529700 |
| H | -3.18477500 | 2.08956800 | 1.94935500 |
| H | -3.98061300 | 0.72223600 | 1.18795500 |
| H | -2.89974500 | 0.78514200 | 4.04372400 |
| H | -3.58515000 | -0.63302500 | 3.27288500 |
| H | -5.31140600 | 0.54093000 | 4.67261600 |
| H | -5.10605500 | 2.00370600 | 3.70416000 |
| H | -5.76983800 | 0.54387700 | 2.96704800 |
| H | -3.72076700 | -1.60856400 | -2.81054800 |
| H | -3.88286200 | 0.01067700 | -3.48370400 |
| H | -6.08958800 | -1.20281100 | -3.52076300 |
| H | -6.00775500 | -1.38671500 | -1.76459300 |
| H | -6.17540000 | 0.22286500 | -2.47997900 |
| H | -2.12396100 | 5.13321500 | -1.43835900 |
| H | -2.78011600 | 5.15012400 | 0.18387500 |
| H | -4.31638500 | 6.35108700 | -1.39146000 |
| H | -4.45126700 | 4.89800900 | -2.38551200 |
| H | -5.11511700 | 4.91197700 | -0.75215800 |
| H | 3.69697600 | 1.02157900 | 1.67566100 |
| H | 6.04370200 | 0.32370600 | 1.45636700 |
| H | 2.80977500 | -0.86112000 | -2.08613000 |
| H | 5.14317200 | -1.53152200 | -2.30948800 |
| H | 8.59973800 | -1.87727300 | -1.42162100 |
| H | 7.02742700 | -2.67131300 | -1.69975500 |
| H | 7.39360100 | -1.12082100 | -2.49551400 |
| H | 3.81106600 | -2.31664700 | 2.35593500 |
| H | -1.36832800 | -3.00400500 | 0.56882200 |
| H | -4.74556800 | -3.77546500 | -1.97984500 |
| H | -2.88069400 | -2.71234700 | 2.48802800 |
| H | -6.25905100 | -3.47246100 | -0.05634000 |
| H | -5.35353300 | -2.90976600 | 2.20351500 |
| H | 1.56929700 | 0.09252100 | 2.69023500 |
| C | 1.97429700 | 3.38477700 | -1.00922300 |
| N | 2.17020200 | 4.55210000 | -1.62785300 |
| C | 2.08182000 | 5.65405800 | -0.86979800 |
| C | 1.61957300 | 4.41680000 | 1.12219800 |
| C | 1.81815200 | 5.64085100 | 0.49920300 |
| H | 2.03849300 | 2.47714000 | -1.61465200 |
| H | 2.22893400 | 6.60118800 | -1.38781400 |
| H | 1.39703000 | 4.33685000 | 2.18352400 |
| H | 1.76446600 | 6.57285900 | 1.05396500 |

**Ni[P(n-Bu)$_3$](Pyr)[Na$_2$CO$_3$OPh]**

| | | | |
|---|---|---|---|
| Ni | 0.88220700 | 0.41811300 | -1.22760500 |
| P | -1.10985800 | 0.00757500 | -0.50359400 |
| C | 0.74940900 | 2.21005500 | -0.68162900 |
| O | 2.70628800 | 0.43298300 | -1.93024000 |
| C | 2.60666400 | -0.85082800 | -2.18436400 |
| O | 1.41795400 | -1.35006300 | -1.94052400 |
| O | 3.58931400 | -1.55441200 | -2.52959500 |
| Na | 2.71485000 | -3.10809300 | -0.9528450 |
| Na | 4.70127000 | -0.20645400 | -0.8462050 |
| O | 3.69358800 | -1.71911100 | 0.50985300 |
| C | 3.05244300 | -1.14403900 | 1.48884100 |
| C | 1.77769200 | -1.59403600 | 1.93867300 |
| C | 1.10326200 | -0.97083800 | 2.98231400 |
| C | 1.64505600 | 0.14073600 | 3.63139200 |
| C | 3.58470600 | -0.02122000 | 2.18407900 |
| C | 2.89718500 | 0.60010700 | 3.22031600 |
| C | -1.54382600 | 0.74158200 | 1.12268300 |
| C | -2.42029700 | 0.56329400 | -1.67701000 |
| C | -1.29168400 | -1.82005200 | -0.34138800 |
| C | -2.31387300 | -2.39433500 | 0.63035800 |
| C | -2.23444500 | -3.91381000 | 0.70721400 |
| C | -3.74883900 | -0.18485000 | -1.68189300 |
| C | -4.71925100 | 0.37593400 | -2.71360800 |
| C | -3.00684700 | 0.84938900 | 1.53307600 |
| C | -3.15181200 | 1.40909200 | 2.94282100 |
| C | -4.59967300 | 1.54197600 | 3.37829600 |
| C | -3.23922700 | -4.50931000 | 1.67667600 |
| C | -6.03882600 | -0.37358400 | -2.74744800 |
| H | 1.33769200 | -2.46848400 | 1.45530400 |
| H | 0.13619800 | -1.36330900 | 3.29869600 |
| H | 1.11017500 | 0.62705100 | 4.44169200 |
| H | 4.57502900 | 0.34050700 | 1.90188400 |

| | | | |
|---|---|---|---|
| H | 3.34683600 | 1.45886100 | 3.71655400 |
| H | -0.29026700 | -2.16935400 | -0.06161500 |
| H | -1.45361400 | -2.20407500 | -1.35750000 |
| H | -2.14586500 | -1.97898600 | 1.63441600 |
| H | -3.33242900 | -2.09661300 | 0.34688000 |
| H | -1.97189300 | 0.51659300 | -2.67726300 |
| H | -2.57690300 | 1.63205200 | -1.47706900 |
| H | -3.57268200 | -1.24916800 | -1.89297700 |
| H | -4.21654000 | -0.15071000 | -0.68946700 |
| H | -1.09127800 | 1.74053200 | 1.11929100 |
| H | -0.96978400 | 0.17395300 | 1.86606200 |
| H | -3.54458600 | 1.49732600 | 0.82626000 |
| H | -3.50441300 | -0.12742500 | 1.48401900 |
| H | -2.65040400 | 2.38453600 | 2.99644100 |
| H | -2.60672500 | 0.75714300 | 3.64052400 |
| H | -4.68585900 | 1.94090500 | 4.39267800 |
| H | -5.15384000 | 2.21199400 | 2.71222900 |
| H | -5.10916100 | 0.57229800 | 3.36035700 |
| H | -1.21467000 | -4.20177400 | 1.00053100 |
| H | -2.38606800 | -4.33453400 | -0.29632900 |
| H | -3.16562400 | -5.59922800 | 1.72200800 |
| H | -3.08609000 | -4.12542700 | 2.69091000 |
| H | -4.26529900 | -4.25963500 | 1.38650500 |
| H | -4.24623300 | 0.34824600 | -3.70442900 |
| H | -4.89659200 | 1.43839400 | -2.49896900 |
| H | -6.72377200 | 0.04097900 | -3.49196500 |
| H | -5.88786700 | -1.43101200 | -2.99025800 |
| H | -6.54310300 | -0.33204800 | -1.77602000 |
| C | 0.01844800 | 3.19197200 | -1.36470200 |
| N | -0.03624600 | 4.49042900 | -1.02405300 |
| C | 0.66035100 | 4.86428500 | 0.05514300 |
| C | 1.46821200 | 2.64251800 | 0.44141800 |
| C | 1.42663300 | 3.98369600 | 0.81549200 |
| H | -0.56444000 | 2.92340200 | -2.24904400 |
| H | 0.59966900 | 5.91894900 | 0.32475900 |
| H | 2.05279100 | 1.93228800 | 1.02761300 |
| H | 1.97414900 | 4.34043000 | 1.68561700 |

**Ni[P(n-Bu)$_3$](Pyr)[Na$_2$CO$_3$OPh-B(OH)$_2$PhOMe]**

| | | | |
|---|---|---|---|
| Ni | 0.70458300 | -0.72390700 | 0.48636300 |
| P | -1.02108900 | 0.54014100 | 0.31179500 |
| C | 0.90710600 | -0.14603500 | 2.25387200 |
| O | 2.38656500 | -1.83128400 | 0.23874800 |
| C | 1.80685300 | -2.41747800 | -0.84732500 |
| O | 0.73145300 | -1.82738300 | -1.21791300 |
| O | 2.29713800 | -3.45077400 | -1.34284400 |
| Na | 4.16088800 | -3.48275700 | 0.29734300 |
| C | -2.26339500 | 0.59248700 | 1.65824900 |
| C | -0.41182400 | 2.26968400 | 0.15108600 |
| C | -1.89176800 | 0.05399900 | -1.22870400 |
| C | -3.31577500 | 0.54199000 | -1.47041200 |
| C | -3.86020000 | 0.03569400 | -2.79960600 |
| C | -1.19255600 | 3.24198300 | -0.72449300 |
| C | -0.63179300 | 4.65539600 | -0.62742000 |
| C | -3.16170800 | 1.82130700 | 1.75842500 |
| C | -4.16580200 | 1.68079600 | 2.89566300 |
| C | -5.06640400 | 2.89379700 | 3.04202500 |
| C | -5.30531900 | 0.43793200 | -3.03140900 |
| C | -1.32815700 | 5.63577700 | -1.55408000 |
| H | -1.88700600 | -1.04547300 | -1.20783300 |
| H | -1.22435400 | 0.34060200 | -2.05460800 |
| H | -3.96080900 | 0.17599900 | -0.65851200 |
| H | -3.37204700 | 1.63866900 | -1.44751000 |
| H | 0.62614800 | 2.20587200 | -0.20059500 |
| H | -0.34597000 | 2.65179800 | 1.17867500 |
| H | -1.16569000 | 2.90135700 | -1.77097400 |
| H | -2.25332800 | 3.25729600 | -0.44251900 |
| H | -1.70413300 | 0.46623900 | 2.59375100 |
| H | -2.86828100 | -0.31329900 | 1.53703300 |
| H | -2.55693400 | 2.72582100 | 1.91718900 |
| H | -3.70575900 | 1.98209300 | 0.81864300 |
| H | -3.62340600 | 1.49985100 | 3.83344100 |
| H | -4.77196000 | 0.78061900 | 2.72425500 |
| H | -5.78110000 | 2.77392500 | 3.86091800 |
| H | -4.48289200 | 3.79866300 | 3.24314800 |
| H | -5.64093300 | 3.07396200 | 2.12696400 |
| H | -3.76556600 | -1.05820500 | -2.81957400 |
| H | -3.22848600 | 0.41429300 | -3.61574400 |
| H | -5.68188800 | 0.07348300 | -3.99131400 |
| H | -5.95545200 | 0.03382900 | -2.24758000 |
| H | -5.42395600 | 1.52724800 | -3.02239200 |
| H | 0.44576800 | 4.63447700 | -0.83735800 |
| H | -0.72123500 | 4.99808000 | 0.41271900 |
| H | -0.89715900 | 6.63844800 | -1.48104500 |
| H | -1.25014700 | 5.32002700 | -2.60090600 |
| H | -2.39534500 | 5.71639800 | -1.32078800 |
| C | 1.46921000 | 1.05878700 | 2.69358500 |
| N | 1.71126900 | 1.36303600 | 3.97876800 |
| C | 1.38292400 | 0.44753800 | 4.89708900 |
| C | 0.56347700 | -1.07644000 | 3.24417300 |
| C | 0.81206300 | -0.78330900 | 4.58299500 |
| H | 1.75752600 | 1.82433700 | 1.97228100 |
| H | 1.58253300 | 0.71457300 | 5.93468000 |
| H | 0.10481900 | -2.02825200 | 2.97404400 |
| H | 0.56197000 | -1.49184400 | 5.36899900 |
| B | 3.76475700 | -0.91065500 | -0.01034900 |
| C | 3.33214700 | 0.54701400 | -0.50696600 |
| O | 4.34843700 | -1.09303300 | 1.28991600 |
| O | 4.55849600 | -1.64714000 | -0.98214200 |
| C | 3.68538900 | 1.71222400 | 0.19313000 |
| C | 3.43077400 | 2.98532500 | -0.30158900 |
| C | 2.80565500 | 3.13912800 | -1.54330200 |
| C | 2.68807000 | 0.74258400 | -1.73901600 |
| C | 2.41675100 | 2.00640700 | -2.26383700 |
| H | 4.16911600 | 1.61928900 | 1.16461900 |
| H | 3.71256800 | 3.87625800 | 0.25269900 |
| O | 2.61276300 | 4.42456300 | -1.96121200 |
| H | 2.37141600 | -0.12234800 | -2.32263200 |
| H | 1.91460900 | 2.09794400 | -3.22185900 |
| C | 1.93089000 | 4.60382100 | -3.18722500 |
| H | 1.83617400 | 5.68071700 | -3.32890400 |
| H | 2.48771500 | 4.17280400 | -4.02845200 |
| H | 0.92772300 | 4.15645400 | -3.16110000 |
| H | 5.18097700 | -0.61787400 | 1.33516400 |
| H | 4.32762400 | -1.42306000 | -1.88635400 |
| Na | 0.30345900 | -3.77456300 | -2.6310620 |
| O | -1.48699600 | -4.59118100 | -1.80387600 |
| C | -2.24597800 | -3.91665500 | -1.00821600 |
| C | -1.84761300 | -3.55581300 | 0.31801100 |
| C | -3.55071800 | -3.47581100 | -1.39190700 |
| C | -2.68534900 | -2.85085100 | 1.17145000 |
| H | -0.85293700 | -3.85758500 | 0.65036300 |
| C | -4.37523600 | -2.76567800 | -0.52954500 |
| H | -3.89196500 | -3.72656400 | -2.39584900 |

| | | | |
|---|---|---|---|
| C | -3.96275300 | -2.44652700 | 0.76672200 |
| H | -2.33733700 | -2.60462800 | 2.17614100 |
| H | -5.36239400 | -2.45446800 | -0.87330800 |
| H | -4.61730700 | -1.90256000 | 1.44421200 |

**TS(transmet), from Ni[P(n-Bu)$_3$](Pyr)[Na$_2$CO$_3$OPh-B(OH)$_2$PhOMe]**

| | | | |
|---|---|---|---|
| Ni | 1.07784100 | 0.35904900 | 0.01774300 |
| P | -1.06131700 | 1.00375300 | -0.27367000 |
| C | 1.42442500 | 2.09056600 | 0.67420500 |
| O | 1.31062100 | -2.04647100 | 1.36772000 |
| C | 1.05335600 | -2.40233900 | 0.07618300 |
| O | 0.68231200 | -1.48251300 | -0.71286800 |
| O | 1.15445500 | -3.61414300 | -0.22332300 |
| Na | 2.49357300 | -4.20045600 | 1.56022800 |
| C | -2.02324400 | 0.85824500 | 1.28679300 |
| C | -1.20115400 | 2.75219300 | -0.84618600 |
| C | -1.97469000 | -0.02557100 | -1.49486900 |
| C | -3.50151400 | -0.02891700 | -1.47392000 |
| C | -4.07181700 | -0.82153700 | -2.64315700 |
| C | -2.46015000 | 3.21445900 | -1.56707500 |
| C | -2.36899600 | 4.67622500 | -1.98692700 |
| C | -3.27031000 | 1.71332500 | 1.48299000 |
| C | -3.99602000 | 1.35013400 | 2.77217600 |
| C | -5.22369600 | 2.20710000 | 3.02119800 |
| C | -5.58910500 | -0.85193400 | -2.63989000 |
| C | -3.60682700 | 5.15382900 | -2.72444700 |
| H | -1.62921300 | -1.05455700 | -1.33353100 |
| H | -1.60036300 | 0.27438800 | -2.48483900 |
| H | -3.84755700 | -0.47953300 | -0.53173400 |
| H | -3.90513500 | 0.99082200 | -1.49959100 |
| H | -0.33024800 | 2.91629600 | -1.49450700 |
| H | -1.01089900 | 3.37882500 | 0.03573900 |
| H | -2.63085800 | 2.59357400 | -2.45801700 |
| H | -3.34406600 | 3.07661000 | -0.93104700 |
| H | -1.32538700 | 1.04759300 | 2.11184000 |
| H | -2.28132000 | -0.20728400 | 1.35140500 |
| H | -2.99637800 | 2.77867000 | 1.49507100 |
| H | -3.96199300 | 1.58753900 | 0.64062300 |
| H | -3.29852500 | 1.43661400 | 3.61720900 |
| H | -4.27898200 | 0.28897500 | 2.72430300 |
| H | -5.73512100 | 1.92775800 | 3.94668300 |
| H | -4.96043800 | 3.26791300 | 3.09634500 |
| H | -5.94634400 | 2.10914800 | 2.20387800 |
| H | -3.66727300 | -1.84166300 | -2.60129300 |
| H | -3.70863400 | -0.37906000 | -3.58337100 |
| H | -5.98665300 | -1.44632800 | -3.46842000 |
| H | -5.96314800 | -1.29105700 | -1.70862500 |
| H | -6.01094000 | 0.15640000 | -2.72492700 |
| H | -1.47933100 | 4.81568800 | -2.61594300 |
| H | -2.20068700 | 5.29577400 | -1.09558600 |
| H | -3.52701100 | 6.20400700 | -3.01858900 |
| H | -3.77697000 | 4.56813900 | -3.63444800 |
| H | -4.50260100 | 5.05275300 | -2.10216900 |
| C | 2.08699900 | 3.02334400 | -0.14600200 |
| N | 2.37847400 | 4.28478400 | 0.19138400 |
| C | 2.02605500 | 4.68656500 | 1.42094000 |
| C | 1.06853300 | 2.55720400 | 1.94572100 |
| C | 1.37924900 | 3.86180900 | 2.33273900 |
| H | 2.41005700 | 2.72318100 | -1.14522200 |
| H | 2.27593400 | 5.71443500 | 1.68228400 |
| H | 0.52811100 | 1.92270700 | 2.65088600 |
| H | 1.11301400 | 4.23099900 | 3.32013300 |
| B | 2.48938600 | -1.29833900 | 1.73655900 |
| C | 3.06402900 | -0.05199900 | 0.03141000 |
| O | 2.38481100 | -0.40765100 | 2.78717100 |
| O | 3.60118700 | -2.17298800 | 1.74357100 |
| C | 4.07637200 | 0.72020700 | 0.65656100 |
| C | 5.38928000 | 0.74749700 | 0.22125100 |
| C | 5.76307200 | 0.01706600 | -0.91563000 |
| C | 3.48737100 | -0.75119400 | -1.11851300 |
| C | 4.79983100 | -0.73164600 | -1.59483200 |
| H | 3.82202300 | 1.29468800 | 1.54498700 |
| H | 6.14791000 | 1.33395000 | 0.73344600 |
| O | 7.07240600 | 0.10268700 | -1.27407300 |
| H | 2.76936400 | -1.35038800 | -1.67484700 |
| H | 5.05964600 | -1.29151600 | -2.48829000 |
| C | 7.47131200 | -0.60894100 | -2.43010100 |
| H | 8.53762000 | -0.42024800 | -2.55152300 |
| H | 7.30496600 | -1.68794500 | -2.31894000 |
| H | 6.93795900 | -0.26141900 | -3.32348300 |
| H | 4.40933200 | -1.70339400 | 1.96636200 |
| Na | -0.25169900 | -3.24654300 | -2.1410050 |
| O | -2.24343000 | -3.49334900 | -1.40946300 |
| C | -3.02093900 | -3.24154600 | -0.40781600 |
| C | -2.53404300 | -2.76614600 | 0.84723100 |
| C | -4.43703300 | -3.40439300 | -0.48793800 |
| C | -3.38648900 | -2.47029800 | 1.90610400 |
| H | -1.45886300 | -2.62123800 | 0.95782200 |
| C | -5.27715500 | -3.09685100 | 0.57313600 |
| H | -4.85016000 | -3.76478400 | -1.42942800 |
| C | -4.76970100 | -2.62087600 | 1.78659100 |
| H | -2.96333900 | -2.10219600 | 2.84183100 |
| H | -6.35251000 | -3.22824000 | 0.45347800 |
| H | -5.43008600 | -2.38401000 | 2.61638100 |
| H | 1.66754400 | 0.21491800 | 2.62456400 |

**Ni[P(n-Bu)$_3$](Pyr)[Na$_2$CO$_3$OPh-B(OH)$_2$](PhOMe), Iso1**

| | | | |
|---|---|---|---|
| Ni | 0.98799400 | 0.77124400 | -0.37735000 |
| P | -1.27097800 | 1.00140700 | -0.37547300 |
| C | 1.17700000 | 2.57440800 | 0.07080600 |
| O | 1.28169700 | -1.45003400 | 1.36469900 |
| C | 1.35322400 | -1.94954800 | 0.07916700 |
| O | 0.78066300 | -1.24040500 | -0.79032200 |
| O | 1.90529100 | -3.05266300 | -0.12120900 |
| Na | 4.16021700 | -2.83410300 | -0.0260130 |
| C | -1.88291300 | 0.65235600 | 1.32640200 |
| C | -1.88248800 | 2.69069400 | -0.79425900 |
| C | -2.13321700 | -0.16665800 | -1.50769800 |
| C | -3.60862500 | -0.50873800 | -1.32117900 |
| C | -4.12386700 | -1.35446700 | -2.47916800 |
| C | -3.31603300 | 2.88135900 | -1.27085200 |
| C | -3.64117300 | 4.34582600 | -1.53489100 |
| C | -3.28609900 | 1.08499400 | 1.73307400 |
| C | -3.61247900 | 0.69297500 | 3.16927100 |
| C | -5.04002200 | 1.02981600 | 3.55876600 |
| C | -5.60094700 | -1.67930400 | -2.35385300 |
| C | -5.05828000 | 4.55347000 | -2.03807800 |
| H | -1.55690000 | -1.09963800 | -1.45729600 |
| H | -1.96374400 | 0.23853500 | -2.51687700 |
| H | -3.73752900 | -1.06816000 | -0.38397600 |
| H | -4.22421100 | 0.39510400 | -1.23395600 |

| | | | |
|---|---|---|---|
| H | -1.19333800 | 3.07493100 | -1.55771500 |
| H | -1.68142900 | 3.31114300 | 0.09116400 |
| H | -3.47893500 | 2.30353000 | -2.19187000 |
| H | -4.02723000 | 2.47904600 | -0.53805700 |
| H | -1.14658200 | 1.11493300 | 2.00028800 |
| H | -1.77363900 | -0.43314100 | 1.45441100 |
| H | -3.39361600 | 2.17327900 | 1.61937200 |
| H | -4.03029100 | 0.63368200 | 1.06471700 |
| H | -2.90598500 | 1.18710300 | 3.85092400 |
| H | -3.44140200 | -0.38569700 | 3.28601800 |
| H | -5.25549600 | 0.76145100 | 4.59686800 |
| H | -5.24361200 | 2.10040100 | 3.44499100 |
| H | -5.75386900 | 0.49282100 | 2.92425100 |
| H | -3.53098000 | -2.27814200 | -2.52445400 |
| H | -3.93837800 | -0.81889000 | -3.42287300 |
| H | -5.95113600 | -2.31363900 | -3.17419200 |
| H | -5.79907200 | -2.20923400 | -1.41605000 |
| H | -6.21075300 | -0.76839700 | -2.35650200 |
| H | -2.92174000 | 4.75005100 | -2.25988100 |
| H | -3.48317900 | 4.91971700 | -0.61137100 |
| H | -5.27644700 | 5.60939400 | -2.22065800 |
| H | -5.22887100 | 4.01458100 | -2.97652400 |
| H | -5.79382700 | 4.18467500 | -1.31508100 |
| C | 1.32841200 | 3.60541000 | -0.87180800 |
| N | 1.38789600 | 4.91453800 | -0.58672400 |
| C | 1.30748700 | 5.26379200 | 0.70342600 |
| C | 1.12036700 | 2.98544700 | 1.41082800 |
| C | 1.18107100 | 4.33923700 | 1.73575100 |
| H | 1.39704800 | 3.36049200 | -1.93418700 |
| H | 1.35281700 | 6.33158800 | 0.91789200 |
| H | 1.02428600 | 2.24831800 | 2.20992100 |
| H | 1.13068800 | 4.67170500 | 2.77011200 |
| B | 2.34192400 | -1.31003900 | 2.22623200 |
| C | 2.92706500 | 0.57408400 | -0.37181400 |
| O | 2.17353200 | -0.61733200 | 3.38262500 |
| O | 3.57655900 | -1.86171600 | 1.95707300 |
| C | 3.83159800 | 1.10176000 | 0.57319400 |
| C | 5.19820300 | 0.84727800 | 0.54288000 |
| C | 5.74615400 | 0.07005400 | -0.48396300 |
| C | 3.51543600 | -0.21086900 | -1.37917800 |
| C | 4.89744300 | -0.45995000 | -1.46087000 |
| H | 3.45379800 | 1.73887600 | 1.37380800 |
| H | 5.87011100 | 1.26404800 | 1.28995400 |
| O | 7.10340400 | -0.10817800 | -0.45048400 |
| H | 2.88163500 | -0.65059800 | -2.15084800 |
| H | 5.29684900 | -1.02705300 | -2.29950800 |
| C | 7.67870700 | -0.81087000 | -1.53083500 |
| H | 8.75400200 | -0.82243100 | -1.35413400 |
| H | 7.31470000 | -1.84724500 | -1.58705400 |
| H | 7.47378900 | -0.32082500 | -2.49128700 |
| H | 4.21946000 | -1.53842800 | 2.59617700 |
| Na | 0.31586200 | -3.24785500 | -2.0453350 |
| O | -1.66196500 | -3.78960700 | -1.41282900 |
| C | -2.43575300 | -3.62473700 | -0.38931400 |
| C | -1.97146700 | -3.08258400 | 0.84657900 |
| C | -3.82273100 | -3.96097200 | -0.41859800 |
| C | -2.82081000 | -2.85907000 | 1.92380600 |
| H | -0.91516500 | -2.83002100 | 0.92991200 |
| C | -4.66353300 | -3.72030500 | 0.66006900 |
| H | -4.22091100 | -4.38630100 | -1.33926300 |
| C | -4.18325500 | -3.15594000 | 1.84628300 |
| H | -2.40972300 | -2.43979600 | 2.84344500 |
| H | -5.71977400 | -3.97633800 | 0.57524800 |
| H | -4.84495700 | -2.96912800 | 2.68828700 |
| H | 1.27830000 | -0.27647400 | 3.46442700 |

**Ni[P(n-Bu)$_3$](Pyr)[Na$_2$CO$_3$OPh-B(OH)$_2$](PhOMe), Iso2**

| | | | |
|---|---|---|---|
| Ni | 1.06935300 | 0.78177900 | -0.39560700 |
| P | -1.19814700 | 1.00611600 | -0.36044500 |
| C | 1.26487300 | 2.57881200 | 0.07382300 |
| O | 1.25913000 | -1.57365600 | 1.25719300 |
| C | 1.24537300 | -2.02898300 | -0.05223900 |
| O | 0.85367900 | -1.20049100 | -0.91342600 |
| O | 1.54929300 | -3.22200500 | -0.26924100 |
| Na | 3.11345800 | -3.99949900 | 1.11678300 |
| C | -1.79272800 | 0.65627200 | 1.34846600 |
| C | -1.77939200 | 2.70924300 | -0.77061700 |
| C | -2.11960900 | -0.14015700 | -1.46877500 |
| C | -3.60488400 | -0.41499200 | -1.24717200 |
| C | -4.19704200 | -1.20784800 | -2.40532900 |
| C | -3.21533200 | 2.95144000 | -1.21467400 |
| C | -3.48380800 | 4.42527900 | -1.49064500 |
| C | -3.15654200 | 1.17125100 | 1.79140900 |
| C | -3.48099100 | 0.77659600 | 3.22703600 |
| C | -4.85775100 | 1.24055800 | 3.66624500 |
| C | -5.68163100 | -1.47194400 | -2.23440300 |
| C | -4.90188200 | 4.68871400 | -1.96352700 |
| H | -1.58601500 | -1.09785000 | -1.41352200 |
| H | -1.95133300 | 0.24333700 | -2.48656500 |
| H | -3.73359800 | -0.98695500 | -0.31721400 |
| H | -4.17126300 | 0.51563500 | -1.12152900 |
| H | -1.09712400 | 3.06834000 | -1.55245400 |
| H | -1.53392900 | 3.32686700 | 0.10536300 |
| H | -3.42507600 | 2.37028900 | -2.12420000 |
| H | -3.92531800 | 2.58921500 | -0.45988100 |
| H | -1.01516100 | 1.04916300 | 2.01922900 |
| H | -1.75077400 | -0.43730500 | 1.44815500 |
| H | -3.19332100 | 2.26647800 | 1.69898500 |
| H | -3.94180300 | 0.78345300 | 1.12982700 |
| H | -2.71206100 | 1.18438200 | 3.89780200 |
| H | -3.41005100 | -0.31583100 | 3.31673300 |
| H | -5.07186300 | 0.96392800 | 4.70249700 |
| H | -4.95555900 | 2.32898300 | 3.58792900 |
| H | -5.63927200 | 0.79801000 | 3.03869200 |
| H | -3.64820300 | -2.15530500 | -2.49434400 |
| H | -4.02124300 | -0.65612800 | -3.34151300 |
| H | -6.08877600 | -2.06719400 | -3.05787200 |
| H | -5.86940500 | -2.01950200 | -1.30457200 |
| H | -6.25052800 | -0.53594800 | -2.19041600 |
| H | -2.76477600 | 4.78867000 | -2.23743800 |
| H | -3.27858500 | 5.00348200 | -0.57916800 |
| H | -5.07823900 | 5.75103300 | -2.15414600 |
| H | -5.11822400 | 4.14696500 | -2.89088700 |
| H | -5.63578300 | 4.36083000 | -1.21925400 |
| C | 1.41705600 | 3.61305000 | -0.86444800 |
| N | 1.47167600 | 4.92132000 | -0.57328400 |
| C | 1.38547300 | 5.26328700 | 0.71854800 |
| C | 1.20353200 | 2.97980800 | 1.41667900 |
| C | 1.25948300 | 4.33298600 | 1.74605000 |
| H | 1.49051000 | 3.37294300 | -1.92760600 |
| H | 1.42647700 | 6.33035000 | 0.93832600 |
| H | 1.11429000 | 2.23219900 | 2.20545800 |
| H | 1.20522300 | 4.66177300 | 2.78167500 |

| | | | |
|---|---|---|---|
| B | 2.41300800 | -1.10333800 | 1.84518000 |
| C | 3.00895800 | 0.59028500 | -0.42612700 |
| O | 2.24944200 | -0.17127500 | 2.82005600 |
| O | 3.59746200 | -1.76344500 | 1.54054300 |
| C | 3.92419200 | 1.14999400 | 0.48965300 |
| C | 5.28491300 | 0.84575700 | 0.48729100 |
| C | 5.80949900 | -0.00434100 | -0.49502900 |
| C | 3.58379600 | -0.24783500 | -1.39855000 |
| C | 4.94997200 | -0.54515100 | -1.45365900 |
| H | 3.55777500 | 1.83690800 | 1.25303300 |
| H | 5.96852100 | 1.28615400 | 1.21024700 |
| O | 7.15728700 | -0.23253000 | -0.43695600 |
| H | 2.94261600 | -0.71390500 | -2.14620300 |
| H | 5.33079400 | -1.19030700 | -2.24100500 |
| C | 7.71011100 | -1.03198500 | -1.46139700 |
| H | 8.78199600 | -1.08008900 | -1.26910900 |
| H | 7.29711400 | -2.05005000 | -1.45398000 |
| H | 7.54007800 | -0.59630900 | -2.45425400 |
| H | 3.07038600 | 0.08068000 | 3.24898400 |
| H | 4.36727300 | -1.17823200 | 1.52610500 |
| Na | 0.11446900 | -3.10672900 | -2.2461250 |
| O | -1.79013600 | -3.72913900 | -1.47543200 |
| C | -2.54010200 | -3.58492100 | -0.43137600 |
| C | -2.04680500 | -3.07486700 | 0.80733400 |
| C | -3.92977200 | -3.91093800 | -0.43964100 |
| C | -2.87398900 | -2.86374500 | 1.90463400 |
| H | -0.98724300 | -2.83583600 | 0.87700600 |
| C | -4.74702700 | -3.68704300 | 0.66028000 |
| H | -4.34944000 | -4.31190800 | -1.36174300 |
| C | -4.23941300 | -3.15010200 | 1.84787000 |
| H | -2.44247300 | -2.46164800 | 2.82249700 |
| H | -5.80687200 | -3.93260100 | 0.59066200 |
| H | -4.88384400 | -2.97331000 | 2.70501800 |

**Ni[P(n-Bu)$_3$](Pyr)(PhOMe), iso-1**

| | | | |
|---|---|---|---|
| Ni | -1.06092300 | -0.45259900 | -0.00481300 |
| P | 1.20203400 | -0.53735000 | 0.01130500 |
| C | 1.89692200 | 0.69774000 | -1.17001400 |
| C | 1.90218100 | -0.08339500 | 1.65757800 |
| C | 1.95620000 | -2.16335200 | -0.44055000 |
| C | 3.30461800 | 0.50744300 | 1.74791300 |
| C | 3.69217500 | 0.85084100 | 3.18069400 |
| C | 3.37853800 | -2.49114900 | -0.00012200 |
| C | 3.81238700 | -3.87714800 | -0.45962400 |
| C | 3.31060000 | 0.51155900 | -1.70497900 |
| C | 3.71567400 | 1.62724800 | -2.65967100 |
| C | 5.07730200 | 1.46374800 | 3.28380100 |
| C | 5.10989100 | 1.43789000 | -3.22918000 |
| C | 5.21830200 | -4.23402300 | -0.01185600 |
| C | -2.93686600 | -0.46065900 | -0.07090200 |
| C | -1.14142100 | 1.39756400 | 0.03502700 |
| H | 1.82082800 | -0.98057300 | 2.28631400 |
| H | 1.18618700 | 0.63492400 | 2.07981800 |
| H | 4.04625200 | -0.18557600 | 1.33010200 |
| H | 3.36273800 | 1.41711000 | 1.13365600 |
| H | 1.86646400 | -2.24265300 | -1.53262000 |
| H | 1.27603300 | -2.92779000 | -0.04306300 |
| H | 4.08487600 | -1.74492400 | -0.38511800 |
| H | 3.44870300 | -2.43424000 | 1.09523000 |
| H | 1.79073200 | 1.67372500 | -0.67503600 |
| H | 1.19005900 | 0.73660000 | -2.00913300 |
| H | 4.03285300 | 0.46277100 | -0.88000900 |
| H | 3.38688200 | -0.45410900 | -2.22569300 |
| H | 3.63732100 | -0.05778400 | 3.79563600 |
| H | 2.94559900 | 1.53866700 | 3.60019400 |
| H | 5.84250400 | 0.78115600 | 2.89885300 |
| H | 5.14475500 | 2.38883400 | 2.70146400 |
| H | 3.65192800 | 2.58898300 | -2.13286000 |
| H | 2.98206300 | 1.68830300 | -3.47478200 |
| H | 5.38477500 | 2.24800800 | -3.91006800 |
| H | 3.74326100 | -3.92887700 | -1.55475000 |
| H | 3.09799100 | -4.62136400 | -0.08232500 |
| H | 5.51342300 | -5.23056700 | -0.35159400 |
| H | 5.95211300 | -3.52154400 | -0.40337900 |
| H | 5.30245700 | -4.21949500 | 1.08006200 |
| H | 5.86304700 | 1.40559500 | -2.43442200 |
| H | 5.18637800 | 0.49831600 | -3.78713200 |
| H | 5.34054100 | 1.70492400 | 4.31715300 |
| C | -1.17903700 | 2.14137600 | -1.15493500 |
| N | -1.04955500 | 3.47286400 | -1.22367800 |
| C | -0.90098100 | 4.13542800 | -0.06974100 |
| C | -1.02497500 | 2.12391500 | 1.22651900 |
| C | -0.89129400 | 3.51050200 | 1.17381100 |
| H | -1.30795600 | 1.63352200 | -2.11280600 |
| H | -0.79696000 | 5.21735900 | -0.14639000 |
| H | -1.03134200 | 1.61567300 | 2.19019100 |
| H | -0.78841200 | 4.09697500 | 2.08384300 |
| C | -3.60967300 | -0.45629500 | -1.30753900 |
| C | -4.97332500 | -0.69869800 | -1.40275700 |
| C | -5.72437800 | -0.92420900 | -0.24313300 |
| C | -3.72264100 | -0.64869100 | 1.07456400 |
| C | -5.09679100 | -0.89377200 | 1.00451800 |
| H | -3.05516300 | -0.26560800 | -2.22635700 |
| H | -5.48454000 | -0.71089900 | -2.36217800 |
| O | -7.05748100 | -1.15040600 | -0.43321600 |
| H | -3.26170600 | -0.61290800 | 2.06196200 |
| H | -5.66307800 | -1.05088800 | 1.91760700 |
| C | -7.84241300 | -1.35448200 | 0.72358500 |
| H | -8.86368200 | -1.51184900 | 0.37664100 |
| H | -7.51381000 | -2.23651300 | 1.28863100 |
| H | -7.81803300 | -0.48335900 | 1.39099000 |

**TS(decarb.) from Ni[P(n-Bu)$_3$](Pyr)[$Na_2CO_3$OPh-B(OH)$_2$](PhOMe), Iso1**

| | | | |
|---|---|---|---|
| Ni | 1.43738900 | 0.22085600 | -0.12995200 |
| P | -0.65948400 | 1.00338300 | -0.11021700 |
| C | 2.40429100 | 1.82090200 | 0.04778700 |
| O | 0.51029000 | -2.31278700 | 1.51514700 |
| C | 0.50610800 | -2.65399000 | 0.18385500 |
| O | 0.93464100 | -1.79241000 | -0.63417000 |
| O | 0.00338600 | -3.75851000 | -0.12119100 |
| Na | -1.93964500 | -4.83388300 | -0.0302980 |
| C | -1.38198000 | 0.84903100 | 1.58548100 |
| C | -0.64954800 | 2.81982200 | -0.45424900 |
| C | -1.87691300 | 0.27328400 | -1.29506900 |
| C | -3.36881400 | 0.61069000 | -1.28428300 |
| C | -4.01577100 | 0.31804300 | -2.63215100 |
| C | -1.86295000 | 3.59142300 | -0.95380900 |
| C | -1.52593500 | 5.06273300 | -1.16780500 |
| C | -2.72361400 | 1.47418200 | 1.94403300 |
| C | -3.03777200 | 1.34365000 | 3.42913500 |
| C | -4.42749000 | 1.84138700 | 3.78203000 |
| C | -5.50257800 | 0.61927200 | -2.65049700 |

| | | | |
|---|---|---|---|
| C | -2.69351500 | 5.86756100 | -1.70883500 |
| H | -1.76967700 | -0.81318500 | -1.18090800 |
| H | -1.45078100 | 0.51833200 | -2.28086200 |
| H | -3.87262100 | 0.01760000 | -0.51132200 |
| H | -3.53896300 | 1.66070200 | -1.02327300 |
| H | 0.16138500 | 2.95563700 | -1.18076600 |
| H | -0.25974000 | 3.27885500 | 0.46564300 |
| H | -2.21659800 | 3.16357600 | -1.90310700 |
| H | -2.70849000 | 3.51075600 | -0.25731800 |
| H | -0.60155800 | 1.27692600 | 2.23574800 |
| H | -1.40204400 | -0.22955400 | 1.80389800 |
| H | -2.73401700 | 2.53675500 | 1.66541600 |
| H | -3.52339600 | 0.99527500 | 1.36629900 |
| H | -2.28019200 | 1.88694600 | 4.01070100 |
| H | -2.93504300 | 0.28953000 | 3.72533100 |
| H | -4.63712200 | 1.75150800 | 4.85162300 |
| H | -4.55338200 | 2.89453600 | 3.50830000 |
| H | -5.19459300 | 1.27284900 | 3.24407900 |
| H | -3.84420100 | -0.73833800 | -2.88512400 |
| H | -3.50405000 | 0.90371500 | -3.41089000 |
| H | -5.95426400 | 0.40124900 | -3.62290300 |
| H | -6.02651900 | 0.02180800 | -1.89643600 |
| H | -5.69460700 | 1.67439200 | -2.42556900 |
| H | -0.66721700 | 5.13688000 | -1.84963800 |
| H | -1.18008700 | 5.49218100 | -0.21722700 |
| H | -2.43109300 | 6.91862200 | -1.85888800 |
| H | -3.03478800 | 5.47267400 | -2.67216700 |
| H | -3.54908200 | 5.83586200 | -1.02519100 |
| C | 2.64425200 | 2.68236800 | -1.05587000 |
| N | 2.74841300 | 4.01031200 | -0.99906200 |
| C | 2.64559900 | 4.58889800 | 0.20846800 |
| C | 2.35484500 | 2.47397200 | 1.30709400 |
| C | 2.46400000 | 3.85437600 | 1.38176500 |
| H | 2.75486400 | 2.24410700 | -2.05074000 |
| H | 2.72478400 | 5.67483900 | 0.23435600 |
| H | 2.20341700 | 1.88735800 | 2.21323000 |
| H | 2.39604200 | 4.36470600 | 2.34043700 |
| B | 1.70139700 | -1.80426300 | 2.04187100 |
| C | 3.38020800 | 0.23021400 | -0.06467100 |
| O | 1.66085100 | -0.77704900 | 2.94336900 |
| O | 2.84599600 | -2.46111200 | 1.72436800 |
| C | 4.25850000 | 0.10799700 | 1.03438800 |
| C | 5.49786200 | -0.50370800 | 0.92728600 |
| C | 5.92613800 | -1.01490400 | -0.30558400 |
| C | 3.86705500 | -0.24361900 | -1.29948900 |
| C | 5.10186800 | -0.87844800 | -1.42565200 |
| H | 3.95980500 | 0.50894600 | 2.00180500 |
| H | 6.16353500 | -0.58437800 | 1.78317100 |
| O | 7.15757600 | -1.60176800 | -0.31409700 |
| H | 3.25298300 | -0.13644800 | -2.19344700 |
| H | 5.41428900 | -1.25573200 | -2.39462600 |
| C | 7.61328700 | -2.10867200 | -1.55170400 |
| H | 8.59992400 | -2.53233500 | -1.36487700 |
| H | 6.95060600 | -2.89407700 | -1.93766400 |
| H | 7.69671300 | -1.31801400 | -2.30859400 |
| H | 3.63301800 | -1.95200100 | 1.94925400 |
| Na | -0.59754200 | -2.73273900 | -2.2241330 |
| O | -2.66735900 | -3.27261900 | -1.53243100 |
| C | -3.52084900 | -2.75117500 | -0.68745200 |
| C | -3.12727200 | -2.34242000 | 0.62203100 |
| C | -4.89840600 | -2.58540900 | -0.99920200 |
| C | -4.03726600 | -1.80975900 | 1.53182400 |
| H | -2.07206200 | -2.41416500 | 0.89387000 |
| C | -5.79394400 | -2.04983700 | -0.08253600 |
| H | -5.23460700 | -2.87685700 | -1.99292600 |
| C | -5.38019100 | -1.65237700 | 1.19299600 |
| H | -3.68471600 | -1.49935700 | 2.51553300 |
| H | -6.83881200 | -1.93637600 | -0.36804000 |
| H | -6.08779900 | -1.23400900 | 1.90259400 |
| H | 0.78100500 | -0.38791100 | 2.97209700 |

**TS(reductive elimination) from Ni[P(n-Bu)$_3$](Pyr)[Na$_2$CO$_3$OPh-B(OH)$_2$](PhOMe), Iso2**

| | | | |
|---|---|---|---|
| Ni | 1.58572600 | 0.01223600 | -0.66138900 |
| P | -0.31954300 | 0.32636600 | 0.43022400 |
| C | 1.62963900 | 2.70119600 | -1.14121500 |
| O | 1.70515000 | -3.07413800 | 0.75144600 |
| C | 1.28774300 | -2.94202100 | -0.55777000 |
| O | 1.78926400 | -2.01427200 | -1.24931400 |
| O | 0.41782900 | -3.76335500 | -0.92307000 |
| Na | -0.33665300 | -4.41440000 | 1.0950690 |
| C | -1.15133400 | -1.14915300 | 1.18009600 |
| C | -0.00300500 | 1.41271300 | 1.90844200 |
| C | -1.61750600 | 1.17299300 | -0.57616400 |
| C | -3.04359800 | 1.35118800 | -0.06049600 |
| C | -3.82390700 | 2.35667100 | -0.89627500 |
| C | -0.97746700 | 2.52073500 | 2.28620000 |
| C | -0.38503200 | 3.48525200 | 3.30496000 |
| C | -2.27795800 | -0.98185900 | 2.19336700 |
| C | -2.69429600 | -2.30603700 | 2.82177400 |
| C | -3.85601000 | -2.16819200 | 3.78903100 |
| C | -5.23671600 | 2.56505200 | -0.38246900 |
| C | -1.32854000 | 4.61936000 | 3.66280700 |
| H | -1.65062500 | 0.61480800 | -1.52195000 |
| H | -1.17970100 | 2.15002000 | -0.82329400 |
| H | -3.56753200 | 0.38707000 | -0.09013900 |
| H | -3.05201900 | 1.67755200 | 0.98605600 |
| H | 0.97814400 | 1.86517400 | 1.71606000 |
| H | 0.16136400 | 0.73527400 | 2.75779900 |
| H | -1.26526300 | 3.08727400 | 1.38826500 |
| H | -1.90970800 | 2.09801000 | 2.68520000 |
| H | -0.32032800 | -1.69711500 | 1.65109400 |
| H | -1.49741900 | -1.74441800 | 0.32165300 |
| H | -1.97498300 | -0.28504200 | 2.98899900 |
| H | -3.15675300 | -0.53887400 | 1.71378900 |
| H | -1.82913300 | -2.75495700 | 3.33692500 |
| H | -2.97551900 | -2.99888300 | 2.01297900 |
| H | -4.14616800 | -3.12828500 | 4.22585300 |
| H | -3.60831500 | -1.49061100 | 4.61319600 |
| H | -4.73452000 | -1.75670800 | 3.28086000 |
| H | -3.85204800 | 2.00603800 | -1.93743300 |
| H | -3.27968800 | 3.31346300 | -0.91226300 |
| H | -5.80537000 | 3.26058600 | -1.00731800 |
| H | -5.78026700 | 1.61386500 | -0.35970500 |
| H | -5.23197500 | 2.96522400 | 0.63809300 |
| H | 0.55359000 | 3.89444700 | 2.90181700 |
| H | -0.09923100 | 2.93126400 | 4.20996200 |
| H | -0.88749700 | 5.30745900 | 4.38941200 |
| H | -1.59624200 | 5.20300100 | 2.77483400 |
| H | -2.26169900 | 4.23904000 | 4.09218100 |
| C | 1.05408600 | 2.61342600 | -2.42903500 |
| N | 0.10477700 | 3.41580400 | -2.90683800 |
| C | -0.35246800 | 4.38670500 | -2.09987500 |

| | | | |
|---|---|---|---|
| C | 1.14387900 | 3.75003100 | -0.33985100 |
| C | 0.13942700 | 4.58530900 | -0.81463300 |
| H | 1.38161700 | 1.82702500 | -3.10867200 |
| H | -1.13772300 | 5.02325200 | -2.50450500 |
| H | 1.53876400 | 3.91502500 | 0.65881100 |
| H | -0.25048400 | 5.38588700 | -0.19016100 |
| B | 2.75018300 | -2.20186000 | 1.16251900 |
| C | 2.63865500 | 1.73408800 | -0.66891100 |
| O | 2.36314500 | -1.13598100 | 1.91700900 |
| O | 4.00806600 | -2.60921300 | 0.87477400 |
| C | 3.31002500 | 1.91870200 | 0.58899200 |
| C | 4.40609200 | 1.17439600 | 0.96366200 |
| C | 4.93558400 | 0.19565100 | 0.08580200 |
| C | 3.21361600 | 0.72888100 | -1.55050200 |
| C | 4.37279500 | 0.00180400 | -1.15301900 |
| H | 2.96514900 | 2.68899400 | 1.27431800 |
| H | 4.90369100 | 1.35029200 | 1.91418300 |
| O | 5.98142800 | -0.55315000 | 0.60302200 |
| H | 2.98788200 | 0.74001000 | -2.61664000 |
| H | 4.77536500 | -0.73669600 | -1.83921400 |
| C | 6.72112500 | -1.31659000 | -0.33851000 |
| H | 7.57570600 | -1.72477700 | 0.20136500 |
| H | 6.12458900 | -2.14186100 | -0.74752900 |
| H | 7.07075800 | -0.68764100 | -1.16547600 |
| H | 3.03863800 | -0.44786300 | 1.99615800 |
| H | 4.69992300 | -1.98368500 | 1.13651000 |
| Na | -0.08371200 | -2.14685000 | -2.7205270 |
| O | -2.12430800 | -1.82496400 | -2.33697600 |
| C | -3.31965600 | -1.72629500 | -1.86365200 |
| C | -3.76448300 | -2.49723100 | -0.74643400 |
| C | -4.28986500 | -0.83559900 | -2.41255300 |
| C | -5.02806700 | -2.33750400 | -0.19029400 |
| H | -3.06498000 | -3.22338300 | -0.32695600 |
| C | -5.55317700 | -0.69307700 | -1.85593400 |
| H | -3.99829600 | -0.24124900 | -3.27757600 |
| C | -5.94022800 | -1.42683800 | -0.72931700 |
| H | -5.30974000 | -2.93834700 | 0.67551900 |
| H | -6.25170700 | 0.01533900 | -2.30115100 |
| H | -6.92847200 | -1.30264000 | -0.29556700 |

**Prod: (reductive elimination) from Ni[P(n-Bu)$_3$](Pyr)[Na$_2$CO$_3$OPh-B(OH)$_2$](PhOMe), Iso1**

| | | | |
|---|---|---|---|
| Ni | 1.58572600 | 0.01223600 | -0.66138900 |
| P | -0.31954300 | 0.32636600 | 0.43022400 |
| C | 1.62963900 | 2.70119600 | -1.14121500 |
| O | 1.70515000 | -3.07413800 | 0.75144600 |
| C | 1.28774300 | -2.94202100 | -0.55777000 |
| O | 1.78926400 | -2.01427200 | -1.24931400 |
| O | 0.41782900 | -3.76335500 | -0.92307000 |
| Na | -0.33665300 | -4.41440000 | 1.0950690 |
| C | -1.15133400 | -1.14915300 | 1.18009600 |
| C | -0.00300500 | 1.41271300 | 1.90844200 |
| C | -1.61750600 | 1.17299300 | -0.57616400 |
| C | -3.04359800 | 1.35118800 | -0.06049600 |
| C | -3.82390700 | 2.35667100 | -0.89627500 |
| C | -0.97746700 | 2.52073500 | 2.28620000 |
| C | -0.38503200 | 3.48525200 | 3.30496000 |
| C | -2.27795800 | -0.98185900 | 2.19336700 |
| C | -2.69429600 | -2.30603700 | 2.82177400 |
| C | -3.85601000 | -2.16819200 | 3.78903100 |
| C | -5.23671600 | 2.56505200 | -0.38246900 |
| C | -1.32854000 | 4.61936000 | 3.66280700 |
| H | -1.65062500 | 0.61480800 | -1.52195000 |
| H | -1.17970100 | 2.15002000 | -0.82329400 |
| H | -3.56753200 | 0.38707000 | -0.09013900 |
| H | -3.05201900 | 1.67755200 | 0.98605600 |
| H | 0.97814400 | 1.86517400 | 1.71606000 |
| H | 0.16136400 | 0.73527400 | 2.75779900 |
| H | -1.26526300 | 3.08727400 | 1.38826500 |
| H | -1.90970800 | 2.09801000 | 2.68520000 |
| H | -0.32032800 | -1.69711500 | 1.65109400 |
| H | -1.49741900 | -1.74441800 | 0.32165300 |
| H | -1.97498300 | -0.28504200 | 2.98899900 |
| H | -3.15675300 | -0.53887400 | 1.71378900 |
| H | -1.82913300 | -2.75495700 | 3.33692500 |
| H | -2.97551900 | -2.99888300 | 2.01297900 |
| H | -4.14616800 | -3.12828500 | 4.22585300 |
| H | -3.60831500 | -1.49061100 | 4.61319600 |
| H | -4.73452000 | -1.75670800 | 3.28086000 |
| H | -3.85204800 | 2.00603800 | -1.93743300 |
| H | -3.27968800 | 3.31346300 | -0.91226300 |
| H | -5.80537000 | 3.26058600 | -1.00731800 |
| H | -5.78026700 | 1.61386500 | -0.35970500 |
| H | -5.23197500 | 2.96522400 | 0.63809300 |
| H | 0.55359000 | 3.89444700 | 2.90181700 |
| H | -0.09923100 | 2.93126400 | 4.20996200 |
| H | -0.88749700 | 5.30745900 | 4.38941200 |
| H | -1.59624200 | 5.20300100 | 2.77483400 |
| H | -2.26169900 | 4.23904000 | 4.09218100 |
| C | 1.05408600 | 2.61342600 | -2.42903500 |
| N | 0.10477700 | 3.41580400 | -2.90683800 |
| C | -0.35246800 | 4.38670500 | -2.09987500 |
| C | 1.14387900 | 3.75003100 | -0.33985100 |
| C | 0.13942700 | 4.58530900 | -0.81463300 |
| H | 1.38161700 | 1.82702500 | -3.10867200 |
| H | -1.13772300 | 5.02325200 | -2.50450500 |
| H | 1.53876400 | 3.91502500 | 0.65881100 |
| H | -0.25048400 | 5.38588700 | -0.19016100 |
| B | 2.75018300 | -2.20186000 | 1.16251900 |
| C | 2.63865500 | 1.73408800 | -0.66891100 |
| O | 2.36314500 | -1.13598100 | 1.91700900 |
| O | 4.00806600 | -2.60921300 | 0.87477400 |
| C | 3.31002500 | 1.91870200 | 0.58899200 |
| C | 4.40609200 | 1.17439600 | 0.96366200 |
| C | 4.93558400 | 0.19565100 | 0.08580200 |
| C | 3.21361600 | 0.72888100 | -1.55050200 |
| C | 4.37279500 | 0.00180400 | -1.15301900 |
| H | 2.96514900 | 2.68899400 | 1.27431800 |
| H | 4.90369100 | 1.35029200 | 1.91418300 |
| O | 5.98142800 | -0.55315000 | 0.60302200 |
| H | 2.98788200 | 0.74001000 | -2.61664000 |
| H | 4.77536500 | -0.73669600 | -1.83921400 |
| C | 6.72112500 | -1.31659000 | -0.33851000 |
| H | 7.57570600 | -1.72477700 | 0.20136500 |
| H | 6.12458900 | -2.14186100 | -0.74752900 |
| H | 7.07075800 | -0.68764100 | -1.16547600 |
| H | 3.03863800 | -0.44786300 | 1.99615800 |
| H | 4.69992300 | -1.98368500 | 1.13651000 |
| Na | -0.08371200 | -2.14685000 | -2.7205270 |
| O | -2.12430800 | -1.82496400 | -2.33697600 |
| C | -3.31965600 | -1.72629500 | -1.86365200 |
| C | -3.76448300 | -2.49723100 | -0.74643400 |
| C | -4.28986500 | -0.83559900 | -2.41255300 |

| | | | |
|---|---|---|---|
| C | -5.02806700 | -2.33750400 | -0.19029400 |
| H | -3.06498000 | -3.22338300 | -0.32695600 |
| C | -5.55317700 | -0.69307700 | -1.85593400 |
| H | -3.99829600 | -0.24124900 | -3.27757600 |
| C | -5.94022800 | -1.42683800 | -0.72931700 |
| H | -5.30974000 | -2.93834700 | 0.67551900 |
| H | -6.25170700 | 0.01533900 | -2.30115100 |
| H | -6.92847200 | -1.30264000 | -0.29556700 |

**Prod: (reductive elimination) from Ni[P(n-Bu)$_3$](Pyr)[Na$_2$CO$_3$OPh-B(OH)$_2$](PhOMe), Iso2**

| | | | |
|---|---|---|---|
| Ni | 1.54535300 | 0.01447500 | -0.53247000 |
| P | -0.29849200 | 0.34547000 | 0.59689200 |
| C | 1.49842600 | 2.66733400 | -1.27736000 |
| O | 2.31135500 | -3.21823800 | 0.81029000 |
| C | 1.58702800 | -3.01945100 | -0.31842100 |
| O | 1.86362400 | -2.00985500 | -1.03799600 |
| O | 0.70515000 | -3.86797000 | -0.57918200 |
| Na | -1.33958500 | -4.42646200 | 0.0887130 |
| C | -1.13578100 | -1.07067000 | 1.45371800 |
| C | -0.03240900 | 1.58942700 | 1.95738700 |
| C | -1.57451000 | 1.09762400 | -0.51265700 |
| C | -3.03713500 | 1.25740500 | -0.10891300 |
| C | -3.75055000 | 2.22746300 | -1.04319300 |
| C | -1.07165400 | 2.67426400 | 2.21377200 |
| C | -0.55682900 | 3.76832100 | 3.13975600 |
| C | -2.27136100 | -0.82697100 | 2.44060400 |
| C | -2.64120300 | -2.09711400 | 3.19782800 |
| C | -3.89218100 | -1.94629900 | 4.04314300 |
| C | -5.20053000 | 2.46280900 | -0.66391300 |
| C | -1.57106100 | 4.87723700 | 3.35571300 |
| H | -1.52954300 | 0.53354600 | -1.45552300 |
| H | -1.15070200 | 2.07849400 | -0.76196300 |
| H | -3.54224600 | 0.28204200 | -0.13613200 |
| H | -3.12726000 | 1.62448700 | 0.92060900 |
| H | 0.92396600 | 2.07009700 | 1.70978600 |
| H | 0.16206900 | 1.01563000 | 2.87506400 |
| H | -1.36871600 | 3.13529500 | 1.26035900 |
| H | -1.98928700 | 2.24064600 | 2.63522300 |
| H | -0.32520800 | -1.61229600 | 1.96703700 |
| H | -1.48089200 | -1.73353400 | 0.64525000 |
| H | -1.99508600 | -0.04449100 | 3.16237200 |
| H | -3.15727200 | -0.45743800 | 1.91035900 |
| H | -1.79242900 | -2.40827100 | 3.82254900 |
| H | -2.78773300 | -2.90994800 | 2.46895200 |
| H | -4.12988600 | -2.86047600 | 4.59455200 |
| H | -3.78145300 | -1.13940700 | 4.77547100 |
| H | -4.75833600 | -1.70069600 | 3.41838600 |
| H | -3.68594200 | 1.84288900 | -2.07141600 |
| H | -3.20188600 | 3.18167800 | -1.04780500 |
| H | -5.69874500 | 3.14724900 | -1.35715400 |
| H | -5.75816300 | 1.52022300 | -0.66439400 |
| H | -5.28182400 | 2.89370800 | 0.34050000 |
| H | 0.36815000 | 4.18632900 | 2.71539500 |
| H | -0.26617200 | 3.32817600 | 4.10341600 |
| H | -1.18677300 | 5.66294900 | 4.01201000 |
| H | -1.84845700 | 5.34631400 | 2.40482500 |
| H | -2.49207400 | 4.49160100 | 3.80620200 |
| C | 0.84617500 | 2.46923900 | -2.51538400 |
| N | -0.14866100 | 3.21500200 | -2.99381000 |
| C | -0.57093400 | 4.24363200 | -2.24157700 |
| C | 1.04274800 | 3.77153900 | -0.53442600 |
| C | -0.00251300 | 4.55326200 | -1.01043600 |
| H | 1.14774600 | 1.63798700 | -3.15249200 |
| H | -1.39109600 | 4.83472800 | -2.64599800 |
| H | 1.49617700 | 4.02081600 | 0.42080500 |
| H | -0.36488700 | 5.39903800 | -0.43090500 |
| B | 3.24911400 | -2.26560300 | 1.25747600 |
| C | 2.56550400 | 1.76955400 | -0.79564400 |
| O | 2.89038600 | -1.07605500 | 1.81335400 |
| O | 4.54947700 | -2.65694400 | 1.25377600 |
| C | 3.32581600 | 2.08574900 | 0.38257000 |
| C | 4.46871100 | 1.41081000 | 0.72356000 |
| C | 4.95598200 | 0.35925800 | -0.09013100 |
| C | 3.09355300 | 0.69925600 | -1.61485900 |
| C | 4.30147400 | 0.03501300 | -1.25516700 |
| H | 3.00269500 | 2.89732500 | 1.03009400 |
| H | 5.01387200 | 1.65927300 | 1.62943400 |
| O | 6.07916700 | -0.27647700 | 0.38892600 |
| H | 2.76117300 | 0.58220000 | -2.64592000 |
| H | 4.66702000 | -0.75404200 | -1.90438700 |
| C | 6.72041200 | -1.16401100 | -0.51197700 |
| H | 7.63052800 | -1.50042200 | -0.01441600 |
| H | 6.08923300 | -2.03264400 | -0.73687600 |
| H | 6.97903500 | -0.65521600 | -1.44896000 |
| H | 5.11364200 | -1.91352400 | 1.50570000 |
| Na | -0.17966500 | -2.20454800 | -2.1687440 |
| O | -2.11019700 | -2.87969400 | -1.29134000 |
| C | -3.27775900 | -2.28993400 | -1.33481200 |
| C | -4.23716400 | -2.44076100 | -0.29769400 |
| C | -3.65867500 | -1.45649700 | -2.41987700 |
| C | -5.48327900 | -1.82725800 | -0.35666300 |
| H | -3.97468600 | -3.05824900 | 0.56348900 |
| C | -4.90593200 | -0.84707900 | -2.47025600 |
| H | -2.94329800 | -1.30738400 | -3.22968600 |
| C | -5.83751900 | -1.03016100 | -1.44618500 |
| H | -6.19014600 | -1.97561000 | 0.45830800 |
| H | -5.15787200 | -0.21773800 | -3.32237500 |
| H | -6.81455600 | -0.55823900 | -1.49493500 |
| H | 2.03884200 | -0.73238200 | 1.47696800 |

**Ni[P(n-Bu)$_3$][Na$_2$CO$_3$OPh-B(OH)$_2$],**

| | | | |
|---|---|---|---|
| Ni | -0.60913300 | -0.22766200 | 0.73784200 |
| P | 1.47469400 | 0.12149200 | 0.32159700 |
| O | -3.56991500 | 1.74967000 | -0.94972500 |
| C | -4.53700700 | 1.72546900 | 0.07455200 |
| O | -4.16180900 | 1.94632000 | 1.25259400 |
| O | -5.67587000 | 1.41648100 | -0.32592300 |
| Na | -4.70473700 | -0.22078800 | -1.8309740 |
| C | 1.98446100 | -0.60934900 | -1.31417900 |
| C | 2.14773400 | 1.85620700 | 0.26564800 |
| C | 2.55457600 | -0.75368100 | 1.55586300 |
| C | 3.94828700 | -1.23506000 | 1.17117000 |
| C | 4.64054000 | -1.97026300 | 2.31202200 |
| C | 3.61943200 | 2.13352400 | 0.54876000 |
| C | 3.95060300 | 3.61957600 | 0.49720800 |
| C | 3.25834000 | -0.15074900 | -2.01382900 |
| C | 3.46060300 | -0.84560400 | -3.35458300 |
| C | 4.71756100 | -0.39003800 | -4.07410800 |
| C | 6.02065100 | -2.47982800 | 1.93699200 |
| C | 5.40538800 | 3.91416000 | 0.81687700 |
| H | 1.95885700 | -1.61851400 | 1.87769600 |
| H | 2.60773600 | -0.09717100 | 2.43572500 |

| | | | |
|---|---|---|---|
| H | 3.88019600 | -1.90553800 | 0.30230300 |
| H | 4.57813400 | -0.39492200 | 0.85189100 |
| H | 1.53662900 | 2.42370200 | 0.97990300 |
| H | 1.87149300 | 2.25924500 | -0.72064600 |
| H | 3.88457800 | 1.74103600 | 1.54122800 |
| H | 4.25985100 | 1.59733000 | -0.16362600 |
| H | 1.13315800 | -0.44701500 | -1.99090100 |
| H | 1.99563300 | -1.69733500 | -1.15922800 |
| H | 3.22513200 | 0.93666600 | -2.17580600 |
| H | 4.13806700 | -0.32941500 | -1.38218900 |
| H | 2.58047300 | -0.66842400 | -3.98770200 |
| H | 3.49433600 | -1.93186400 | -3.19290900 |
| H | 4.84741900 | -0.89775900 | -5.03412100 |
| H | 4.69284600 | 0.68720400 | -4.27198500 |
| H | 5.61163000 | -0.58794700 | -3.47288500 |
| H | 4.00750800 | -2.80723300 | 2.63731600 |
| H | 4.71262200 | -1.29960900 | 3.17951400 |
| H | 6.50301900 | -3.00485600 | 2.76626000 |
| H | 5.96996800 | -3.17495000 | 1.09187800 |
| H | 6.67980200 | -1.65673000 | 1.64001400 |
| H | 3.29700800 | 4.15832200 | 1.19700900 |
| H | 3.69952500 | 4.00795100 | -0.49957300 |
| H | 5.62636600 | 4.98440600 | 0.77331000 |
| H | 5.66827600 | 3.56408000 | 1.82113700 |
| H | 6.07613300 | 3.40992500 | 0.11266400 |
| B | -2.21199500 | 1.59463800 | -0.68408300 |
| O | -1.43405400 | 1.32671800 | -1.77015600 |
| O | -1.67780100 | 1.69633000 | 0.57385400 |
| H | -0.55211200 | 1.07968600 | -1.45019700 |
| H | -2.44423400 | 1.82631600 | 1.18203100 |
| Na | -5.40525500 | -0.03293700 | 1.7024440 |
| O | -4.22600400 | -1.10399400 | 0.16536600 |
| C | -3.03614800 | -1.64760000 | 0.09150400 |
| C | -2.62675300 | -2.40385300 | -1.02371300 |
| C | -2.08926100 | -1.49420800 | 1.17841900 |
| C | -1.38107100 | -3.07483400 | -1.05021300 |
| H | -3.34688200 | -2.57773100 | -1.82587800 |
| C | -0.80480500 | -2.15802700 | 1.12470700 |
| H | -2.50335700 | -1.17261400 | 2.13977300 |
| C | -0.49505700 | -2.99013000 | -0.00053500 |
| H | -1.13667000 | -3.69326900 | -1.91320500 |
| H | -0.25747900 | -2.32970000 | 2.05612100 |
| H | 0.44474100 | -3.53978800 | -0.01769100 |

## Supplementary Methods

### 1. General

Unless otherwise noted, all materials including dry solvents were obtained from commercial suppliers and used as received. $Ni(OAc)_2 \cdot 4H_2O$ and $P(n\text{-}Bu)_3$ were obtained from Kanto Chemicals. $Na_2CO_3$ was obtained from Wako Chemicals. Toluene was purified by Glass Contour. Anisole and 1,4-dioxane were distilled with Na/ketyl before the use. Phenyl nicotinate (**1A**)[1], phenyl 2-naphthoate (**1B**)[1], phenyl thiophene-2-carboxylate (**1K**)[1], phenyl thiophene-3-carboxylate (**1L**)[1], phenyl furan-3-carboxylate (**1N**)[1], phenyl furan-2-carboxylate (**1O**)[1], phenyl 2-phenylthiazole-4-carboxylate (**1R**)[1], phenyl isonicotinate (**1T**)[1], phenyl 2-phenylquinoline-4-carboxylate (**1V**)[1], phenyl picolinatephenyl (**1AC**)[1], pyrazine-2-carboxylate (**1AD**)[1], 4-oxo-4*H*-chromene-2-carboxylic acid (**1AB**)[2], 5-(methoxycarbonyl)nicotinic acid (**1AA**)[3], and 4-methylpiperazine-1-carbothioamide (**1S**)[4] were synthesized according to procedures reported in the literature. Phenyl 3-methylbenzoate (**1J**)[5], phenyl 4-fluorobenzoate (**1G**)[6], methyl phenyl terephthalate (**1F**)[7], phenyl 4-methylbenzoate (**1H**)[7], phenyl 1-naphthoate (**1C**)[8], phenyl 2-methoxybenzoate (**1E**)[8], and phenyl quinoline-3-carboxylate (**1W**)[8] were synthesized by known procedures using the corresponding carboxylic acids, phenol, EDC·HCl, and DMAP, and the spectra matched with those of reported compounds in the literature. Unless otherwise noted, all reactions were performed with dry solvents under an atmosphere of $N_2$ gas in dried glassware using standard vacuum-line techniques. All coupling reactions were performed in 20-mL glass vessel tubes equipped with J. Young® O-ring tap and heated in an 8-well reaction block (heater + magnetic stirrer) unless otherwise noted. All work-up and purification procedures were carried out with reagent-grade solvents in air.

Analytical thin-layer chromatography (TLC) was performed using E. Merck silica gel 60 $F_{254}$ precoated plates (0.25 mm). The developed chromatogram was analyzed by UV lamp (254 nm). Flash column chromatography was performed with E. Merck silica gel 60 (230–400 mesh) or Biotage Isolera® equipped with Biotage SNAP Cartridge KP-Sil columns using hexane/EtOAc as an eluent. Preparative thin-layer chromatography (PTLC) was performed using Wakogel B5-F silica coated plates (0.75 mm) prepared in our laboratory. Preparative high performance liquid chromatography (preparative HPLC) was performed with a Biotage Isolera®, one equipped with Biotage SNAP Cartridge KP-C18-HS columns using acetonitrile/water as an eluent. Preparative gel permeation chromatography (GPC) was performed with a JAI LC-9204 instrument equipped with JAIGEL-1H/JAIGEL-2H columns using chloroform as an eluent. Gas chromatography (GC) analysis was conducted on a Shimadzu GC-2010 instrument equipped with a HP-5 column (30 m × 0.25 mm, Hewlett-Packard) with dodecane as an internal standard. GCMS analysis was conducted on a Shimadzu GCMS-QP2010 instrument equipped with a HP-5 column (30 m × 0.25 mm, Hewlett-Packard). High-resolution mass spectra (HRMS) were obtained from a JEOL JMS-T100TD instrument (DART), Thermo Scientific Exactive Plus Orbitrap MS (ESI) and MSI.TOKYO Inc. MULTUM-FAB (FAB). Nuclear magnetic resonance (NMR) spectra were recorded on a JEOL JNM-ECA-400 ($^1$H 400 MHz, $^{13}$C 100 MHz) and JNM-ECA-600 ($^1$H 600 MHz, $^{13}$C 150 MHz) spectrometer. Chemical shifts for $^1$H NMR are expressed in parts per million (ppm) relative to tetramethylsilane (δ 0.00 ppm). Chemical shifts for $^{13}$C NMR are expressed in ppm relative to $CDCl_3$ (δ 77.0 ppm). Data are reported as follows: chemical shift, multiplicity (s = singlet, d = doublet, dd = doublet of doublets, t = triplet, td = triplet of doublets, q = quartet, m = multiplet, br = broad signal), coupling constant (Hz), and integration.

## 2. Preparation of Arenecarboxylic Acid Phenyl Esters 1

### 2-1. Method A

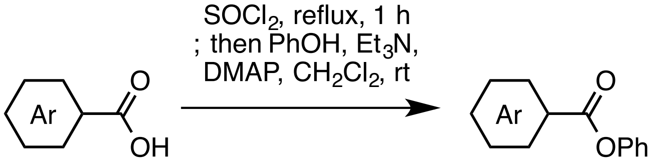


Thionyl chloride (0.5 M) was added to the carboxylic acid (1.0 equiv) and the mixture was refluxed for 1 h. The solution was concentrated *in vacuo*. To the residue were added $CH_2Cl_2$ (0.5 M), phenol (1.0 equiv), and then *N*,*N*-dimethyl-4-aminopyridine (DMAP: 1 mol%). After cooling the mixture to 0 °C, triethylamine ($Et_3N$: 1.2 equiv) was slowly added and then the reaction mixture was warmed to room temperature. After stirring the mixture for 1 h, $NaHCO_3$aq was added to the resulting mixture to quench the reaction. The mixture was extracted three times with $CH_2Cl_2$. The combined organic layer was dried over $Na_2SO_4$, and then filtrated. The filtrate was concentrated *in vacuo* and the residue was purified by recrystallization or flash column chromatography to afford the corresponding phenyl ester **1**.

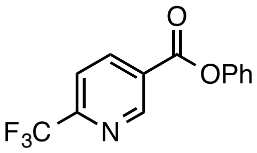


**1X**

**Phenyl 6-(trifluoromethyl)nicotinate (1X)**

Following the method A, purification by recrystallization (hexane) afforded **1X** as a white solid (741 mg, 88% yield; 3.15 mmol scale). $^1$H NMR ($CDCl_3$, 400 MHz) δ 9.48 (s, 1H), 8.64 (d, *J* = 8.0 Hz, 1H), 7.86 (d, *J* = 8.0 Hz, 1H), 7.47 (t, *J* = 8.0 Hz, 2H), 7.33 (t, *J* = 8.0 Hz, 1H), 7.24 (d, *J* = 8.0 Hz, 2H); $^{13}$C NMR ($CDCl_3$, 100 MHz) δ 162.6, 151.7 (q, $J_{C–F}$ = 35 Hz), 151.4, 150.2, 139.3, 129.7, 128.1, 126.5, 121.4, 121.0 (q, $J_{C–F}$ = 279 Hz), 120.3 (q, $J_{C–F}$ = 3.0 Hz); HRMS (DART) *m*/*z* calcd for $C_{13}H_9F_3NO_2$ $[M+H]^+$: 268.0585, found: 268.0585.

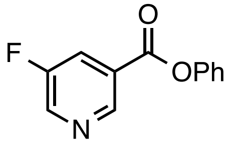


**1Y**

**Phenyl 5-fluoronicotinate (1Y)**

Following the method A, purification by flash column chromatography (hexane/EtOAc = 5:1) afforded **1Y** as a white solid (470 mg, 77% yield; 2.65 mmol scale). $^1$H NMR ($CDCl_3$, 400 MHz) δ 9.22 (s, 1H), 8.72 (d, *J* = 2.8 Hz, 1H), 8.16–8.11 (m, 1H), 7.45 (t, *J* = 8.0 Hz, 2H), 7.30 (t, *J* = 8.0 Hz, 1H), 7.23 (d, *J* = 8.0 Hz, 2H); $^{13}$C NMR ($CDCl_3$, 100 MHz) δ 161.5 (d, $J_{C–F}$ = 238 Hz), 157.7, 150.3, 147.0 (d, $J_{C–F}$ = 4.0 Hz), 142.6 (d, $J_{C–F}$ = 24 Hz), 129.6, 126.9 (d, $J_{C–F}$ = 3.0 Hz), 126.4, 124.0 (d, $J_{C–F}$ = 20 Hz), 121.3; HRMS (DART) *m*/*z* calcd for $C_{12}H_9FNO_2$ $[M+H]^+$: 218.0617, found: 218.0620.

### 2-2. Method B

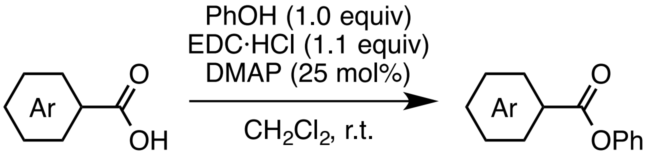

To a round-bottomed flask with the carboxylic acid (1.0 equiv) were added phenol (1.0 equiv), 1-(3-dimethylaminopropyl)-3-ethylcarbodiimide hydrochloride (EDC·HCl: 1.1 equiv), *N,N*-dimethyl-4-aminopyridine (DMAP: 0.25 equiv) and $CH_2Cl_2$ (0.5 M). After stirring the mixture for several hours with monitoring reaction progress with TLC, the reaction was quenched with saturated $NaHCO_3$aq and extracted three times with $CH_2Cl_2$. The combined organic layer was dried over $Na_2SO_4$, filtrated, and concentrated *in vacuo*. The residue was purified by recrystallization or flash column chromatography to afford the corresponding phenyl ester **1**.

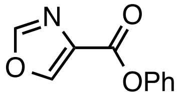

**1Q**

**Phenyl oxazole-4-carboxylate (1Q)**

Following the method B, purification by flash column chromatography (hexane/EtOAc = 5:1) afforded **1Q** as a white solid (800 mg, 96% yield; 4.4 mmol scale). $^1$H NMR ($CDCl_3$, 400 MHz) δ 8.45 (s, 1H), 8.02 (s, 1H), 7.43 (t, *J* = 7.6 Hz, 2H), 7.28 (t, *J* = 7.6 Hz, 1H), 7.22 (d, *J* = 7.6 Hz, 2H); $^{13}$C NMR ($CDCl_3$, 100 MHz) δ 159.2, 151.6, 150.1, 145.1, 132.7, 129.5, 126.2, 121.5; HRMS (DART) *m*/*z* calcd for $C_{10}H_8NO_3$ $[M+H]^+$: 190.0504, found: 190.0505.

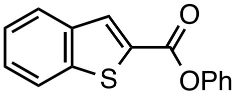

**1P**

**Phenyl benzo[*b*]thiophene-2-carboxylate (1P)**

Following the method B, purification by recrystallization (hexane) afforded **1P** as a white solid (708 mg, 93% yield; 3.0 mmol scale). $^1$H NMR ($CDCl_3$, 400 MHz) δ 8.25 (s, 1H), 7.93 (d, *J* = 8.0 Hz, 1H), 7.91 (d, *J* = 8.4 Hz, 1H), 7.50 (dd, *J* = 8.4, 7.2 Hz, 1H), 7.47–7.40 (m, 3H), 7.31–7.23 (m, 3H); $^{13}$C NMR ($CDCl_3$, 100 MHz) δ 161.2, 150.6, 142.6, 138.6, 132.7, 131.9, 129.5, 127.3, 126.1, 125.7, 125.1, 122.8, 121.6; HRMS (DART) *m*/*z* calcd for $C_{15}H_{11}O_2S$ $[M+H]^+$: 255.0480, found: 255.0477.

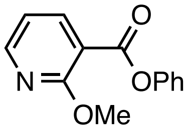

**1AA**

**Phenyl 2-methoxynicotinate (1AA)**

Following the method B, purification by flash column chromatography (hexane/EtOAc = 7:1) afforded **1AA** as colorless liquid (1.22 g, 91% yield; 6.0 mmol scale). $^1$H NMR ($CDCl_3$, 400 MHz) δ 8.38 (d, *J* = 5.2 Hz, 1H), 8.35 (d, *J* = 7.6 Hz, 1H), 7.42 (t, *J* = 8.0 Hz, 2H), 7.27 (t, *J* = 8.0 Hz, 1H), 7.23 (d, *J* = 8.0 Hz, 2H), 7.01 (dd, *J* = 7.6, 5.2 Hz, 1H), 4.08 (s, 3H); $^{13}$C NMR

(CDCl$_3$, 100 MHz) δ 163.4, 163.0, 151.6, 150.8, 141.8, 129.5, 126.0, 121.8, 116.5, 113.4, 54.3; HRMS (DART) *m*/*z* calcd for $C_{13}H_{12}NO_3$ [M+H]$^+$: 230.0817, found: 230.0819.

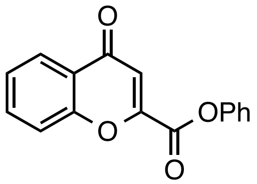

**1AB**

**Phenyl 4-oxo-4*H*-chromene-2-carboxylate (1AB)**

Following the method B, purification by flash column chromatography (hexane/EtOAc = 10:1) afforded **1AB** as a yellow solid (462 mg, 30% yield; 5.8 mmol scale). $^1$H NMR (CDCl$_3$, 400 MHz) δ 8.23 (d, *J* = 8.4 Hz, 1H), 7.78 (t, *J* = 8.8 Hz, 1H), 7.65 (d, *J* = 8.8 Hz, 1H), 7.51–7.43 (m, 3H), 7.33 (t, *J* = 7.6 Hz, 1H), 7.31 (s, 1H), 7.25 (d, *J* = 7.6 Hz, 2H); $^{13}$C NMR (CDCl$_3$, 100 MHz) δ 178.2, 159.1, 156.0, 151.6, 150.0, 134.9, 129.7, 126.7, 126.1, 125.8, 124.4, 121.1, 118.8, 115.7; HRMS (DART) *m*/*z* calcd for $C_{16}H_{11}O_4$ [M+H]$^+$: 267.0657, found: 267,0654.

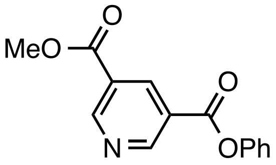

**1Z**

**3-Methyl 5-phenyl pyridine-3,5-dicarboxylate (1Z)**

Following the method B, purification by recrystallization from hexane afforded **1Z** as a white solid (570 mg, 44% yield; 5.0 mmol scale). $^1$H NMR (CDCl$_3$, 400 MHz) δ 9.53 (d, *J* = 0.8 Hz, 1H), 9.44 (d, *J* = 2.0 Hz, 1H), 9.03 (dd, *J* = 2.0, 0.8 Hz, 1H), 7.46 (t, *J* = 8.0 Hz, 2H), 7.32 (t, *J* = 8.0 Hz, 1H), 7.24 (d, *J* = 8.0 Hz, 2H), 4.02 (s, 3H); $^{13}$C NMR (CDCl$_3$, 100 MHz) δ 164.8, 163.0, 154.7, 154.6, 150.3, 138.5, 129.6, 126.4, 126.1, 125.6, 121.4, 52.8; HRMS (DART) *m*/*z* calcd for $C_{14}H_{12}NO_4$ [M+H]$^+$: 258.0766, found: 258.0770.

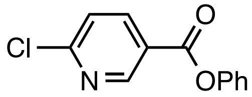

**S1**

**Phenyl 6-chloronicotinate (S1)**

Following the method B, purification by recrystallization (hexane) afforded **S1** as a white solid (1.82 g, 79% yield; 10 mmol scale). $^1$H NMR (CDCl$_3$, 400 MHz) δ 9.17 (d, *J* = 2.8 Hz, 1H), 8.39 (dd, *J* = 8.4, 2.8 Hz, 1H), 7.50 (d, *J* = 8.4 Hz, 1H), 7.46 (dd, *J* = 8.0, 7.6 Hz, 2H), 7.31 (t, *J* = 8.0 Hz, 1H), 7.22 (d, *J* = 7.6 Hz, 2H); $^{13}$C NMR (CDCl$_3$, 100 MHz) δ 163.0, 156.3, 151.6, 150.3, 140.0, 129.6, 126.4, 124.6, 124.4, 121.4; HRMS (DART) *m*/*z* calcd for $C_{12}H_9ClNO_2$ [M+H]$^+$: 234.0322, found: 234.0318.

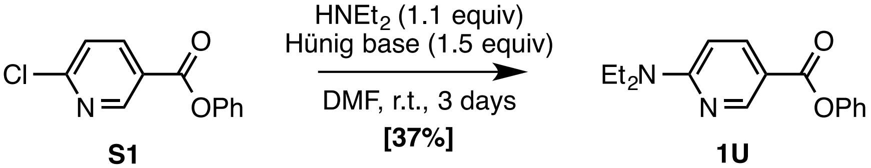


To a solution of phenyl 6-chloronicotinate (**S1**: 467 mg, 2.0 mmol) in DMF (4.0 mL) were added diethylamine (160.9 mg, 2.2 mmol, 1.1 equiv) and *N,N*-diisopropylethylamine (Hünig base: 510 mL, 3.0 mmol, 1.5 equiv). The mixture was stirred for 3 days at room temperature. The reaction was quenched with $NH_4Cl$aq and extracted four times with EtOAc. The combined organic layers were washed with brine, and then dried over $Na_2SO_4$. After filtration of the organic layer, the mixture was concentrated *in vacuo*. Purification by flash column chromatography (hexane/EtOAc = 15:1 to 10:1) afforded **1U** as a white solid (198 mg, 37% yield). $^1$H NMR ($CDCl_3$, 400 MHz) δ 8.96 (d, *J* = 2.0 Hz, 1H), 8.08 (dd, *J* = 9.2, 2.0 Hz, 1H), 7.40 (t, *J* = 8.0 Hz, 2H), 7.24 (t, *J* = 8.0 Hz, 1H), 7.19 (d, *J* = 8.0 Hz, 2H), 6.48 (d, *J* = 9.2 Hz, 1H), 3.59 (q, *J* = 7.2 Hz, 4H), 1.22 (t, *J* = 7.2 Hz, 6H); $^{13}$C NMR ($CDCl_3$, 100 MHz) δ 164.8, 159.5, 152.3, 151.0, 138.4, 129.3, 125.5, 121.8, 112.2, 104.4, 42.9, 12.8; HRMS (DART) *m/z* calcd for $C_{16}H_{19}N_2O_2$ $[M+H]^+$: 271.1447, found: 271.1451.

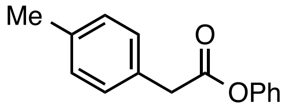


**1AG**

**Phenyl 2-(*p*-tolyl)acetate (1AG)**

Following the method B, purification by flash column chromatography (hexane/EtOAc = 20:1) afforded **1AG** as colorless oil (1.12 g, 99% yield; 5 mmol scale). $^1$H NMR ($CDCl_3$, 400 MHz) δ 7.34 (t, *J* = 7.6 Hz, 2H), 7.27 (d, *J* = 8.0 Hz, 2H), 7.21 (t, *J* = 7.6 Hz, 1H), 7.16 (d, *J* = 8.0 Hz, 2H), 7.05 (d, *J* = 7.6 Hz, 2H), 3.81 (s, 2H), 2.35 (s, 3H); $^{13}$C NMR ($CDCl_3$, 100 MHz) δ 170.2, 150.8, 137.0, 130.4, 129.4, 129.3, 129.1, 125.8, 121.4, 41.0, 21.1; HRMS (ESI) *m/z* calcd for $C_{15}H_{14}O_2Na$ $[M+Na]^+$: 249.0886, found: 249.0876.

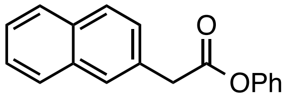


**1AH**

**Phenyl 2-(naphthalen-2-yl)acetate (1AH)**

Following the method B, purification by recrystallization (hexane) afforded **1AH** as a white solid (1.10 g, 76% yield; 5 mmol scale). $^1$H NMR ($CDCl_3$, 400 MHz) δ 7.88–7.80 (m, 4H), 7.54–7.44 (m, 3H), 7.35 (dd, *J* = 8.0, 7.6 Hz, 2H), 7.21 (t, *J* = 7.6 Hz, 1H), 7.06 (d, *J* = 8.0 Hz, 2H), 4.02 (s, 2H); $^{13}$C NMR ($CDCl_3$, 100 MHz) δ 170.0, 150.7, 133.5, 132.6, 130.9, 129.4, 128.4, 128.1, 127.71, 127.67, 127.2, 126.3, 126.0, 125.9, 121.4, 41.6; HRMS (ESI) *m/z* calcd for $C_{18}H_{14}O_2Na$ $[M+Na]^+$: 285.0886, found: 285.0876.

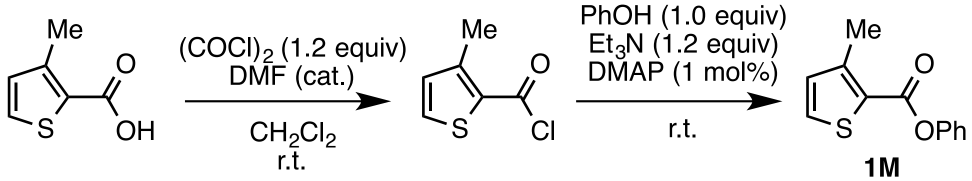


**1M**

**Phenyl 3-methylthiophene-2-carboxylate (1M)**

To a solution of 3-methylthiophene-2-carboxylic acid (713 mg, 5.0 mmol, 1.0 equiv) in $CH_2Cl_2$ (10 mL) were added oxalyl chloride (762 mg, 6.0 mmol, 1.2 equiv) and a portion of DMF. After stirring for 1.5 h at room temperature, phenol (470 mg, 5.0 mmol, 1.0 equiv) and *N,N*-dimethyl-4-aminopyridine (DMAP: 6.1 mg, 0.05 mmol, 1 mol%) were added to the mixture. Triethylamine ($Et_3N$: 470 mg, 6.0 mmol, 1.2 equiv) was slowly added to the mixture at 0 °C. After stirring the solution overnight, the reaction mixture was quenched with saturated $NaHCO_3$aq and extracted three times with $CH_2Cl_2$. The combined organic layer was dried over $Na_2SO_4$, filtrated, and concentrated *in vacuo*. Purification by flash column chromatography (hexane/EtOAc = 20:1) afforded **1M** as brown oil (990 mg, 91% yield). $^1$H NMR ($CDCl_3$, 400 MHz) δ 7.49 (d, $J$ = 5.2 Hz, 1H), 7.42 (t, $J$ = 8.0 Hz, 2H), 7.26 (t, $J$ = 8.0 Hz, 1H), 7.21 (d, $J$ = 8.0 Hz, 2H), 6.99 (d, $J$ = 5.2 Hz, 1H), 2.61 (s, 3H); $^{13}$C NMR ($CDCl_3$, 100 MHz) δ 161.1, 150.5, 148.0, 131.9, 131.1, 129.4, 125.9, 125.6, 121.8, 16.1; HRMS (DART) *m*/*z* calcd for $C_{12}H_{11}O_2S$ $[M+H]^+$: 219.0480, found: 219.0483.

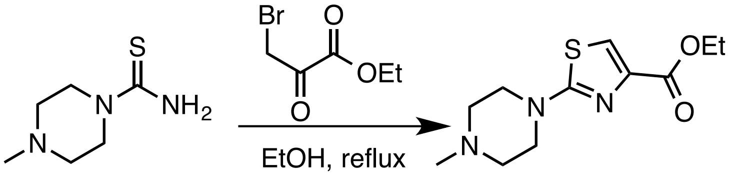


**S2** **S3**

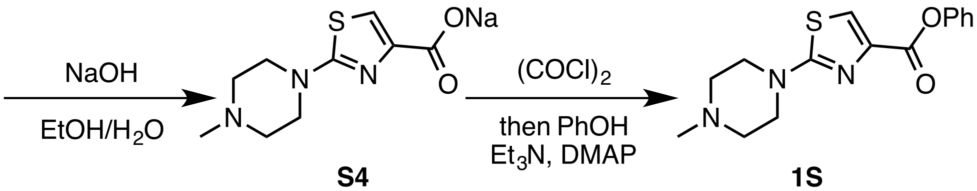


**S4** **1S**

[69% 3 steps]

**Phenyl 2-(4-methylpiperazin-1-yl)thiazole-4-carboxylate (1S)**

A mixture of 4-methylpiperazine-1-carbothioamide (1.1 g, 6.7 mmol, 1.0 equiv) and ethyl 3-bromo-2-oxopropanoate (935 mL, 7.4 mmol, 1.1 equiv) in EtOH (10 mL) was refluxed overnight. After cooling to room temperature, the mixture was concentrated *in vacuo*. $NaHCO_3$aq was added to the resulted solid and the mixture was extracted with $Et_2O$. The combined organic layer was dried over $MgSO_4$, filtrated, and concentrated *in vacuo* to afford ethyl 2-(4-methylpiperazin-1-yl)thiazole-4-carboxylate (**S3**: 1.57 g, 91% yield) as orange oil. **S3** was used without further purification.

The obtained oil **S3** (1.57 g, 6.1 mmol) was treated with NaOH (270 mg, 6.7 mmol, 1.1 equiv) in $EtOH/H_2O$ (10 mL/5 mL) at room temperature. After stirring overnight, the mixture was concentrated *in vacuo* to afford sodium 2-(4-methylpiperazin-1-yl)thiazole-4-carboxylate **S4** (1.53 g, quant.) as a yellow solid.

To a solution of the sodium carboxylate **S4** (1.53 g, 6.14 mmol) in $CH_2Cl_2$ (20 mL) were added oxalyl chloride (791 mL, 9.2 mmol, 1.5 equiv) and a portion of DMF. After stirring for 1.5

h, phenol (866 mg, 9.2 mmol, 1.5 equiv) and *N,N*-dimethyl-4-aminopyridine (DMAP: 10.0 mg, 0.05 mmol, 1 mol%) were added to the mixture. Then, triethylamine ($Et_3N$: 1.72 mL, 12 mmol, 2.0 equiv) was slowly added to the mixture at 0 °C. After stirring the solution overnight, the reaction mixture was quenched with saturated $NaHCO_3$aq and extracted three times with $CH_2Cl_2$. The combined organic layer was dried over $Na_2SO_4$, filtrated, and concentrated *in vacuo*. Purification by flash column chromatography ($CHCl_3$/MeOH = 20:1) and then preparative HPLC ($H_2O$/MeCN) afforded phenyl 2-(4-methylpiperazin-1-yl)thiazole-4-carboxylate **1S** as a brown solid (1.38 g, 74% yield). $^1$H NMR ($CDCl_3$, 400 MHz) δ 7.67 (s, 1H), 7.40 (dd, *J* = 8.0, 7.6 Hz, 2H), 7.24 (t, *J* = 8.0 Hz, 1H), 7.19 (d, *J* = 7.6 Hz, 2H), 3.59 (t, *J* = 4.8 Hz, 4H), 2.53 (t, *J* = 4.8 Hz, 4H), 2.34 (s, 3H); $^{13}$C NMR ($CDCl_3$, 100 MHz) δ 170.9, 159.9, 150.7, 143.0, 129.4, 125.8, 121.7, 118.2, 54.1, 48.3, 46.1; HRMS (DART) *m*/*z* calcd for $C_{15}H_{18}N_3O_2S$ $[M+H]^+$: 304.1120, found: 304.1125.

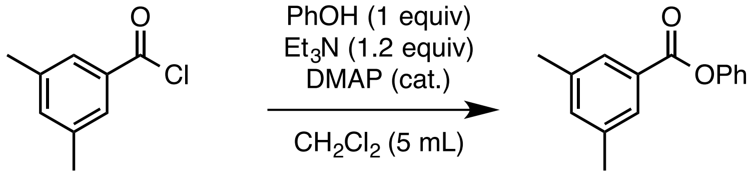


**1D**

To a solution of phenol (470 mg, 5.0 mmol, 1.0 equiv), DMAP (6.0 mg, 0.05 mmol, 1 mol%) and $Et_3N$ (607 mg, 6.0 mmol, 1.2 equiv) in $CH_2Cl_2$ (5 mL) was slowly added 3,5-dimethylbenzoyl chloride (0.74 mL, 5.0 mmol, 1.0 equiv) at 0 °C. After stirring for 3 h, the reaction mixture was quenched with saturated $NaHCO_3$aq and extracted three times with $CH_2Cl_2$. The combined organic layer was dried over $Na_2SO_4$, filtrated, and concentrated *in vacuo*. Purification by flash column chromatography (hexane/EtOAc = 50:1) afforded **1D** as colorless liquid (1.13 g, quant). $^1$H NMR ($CDCl_3$, 400 MHz) δ 7.83 (s, 2H), 7.43 (t, *J* = 8.4 Hz, 2H), 7.30–7.24 (m, 2H), 7.21 (d, *J* = 8.4 Hz, 2H), 2.41 (s, 6H); $^{13}$C NMR ($CDCl_3$, 100 MHz) δ 165.5, 151.0, 138.3, 135.2, 129.5, 129.4, 127.9, 125.8, 121.7, 21.2; HRMS (DART) *m*/*z* calcd for $C_{15}H_{15}O_2$ $[M+H]^+$: 227.1072, found: 227.1075.

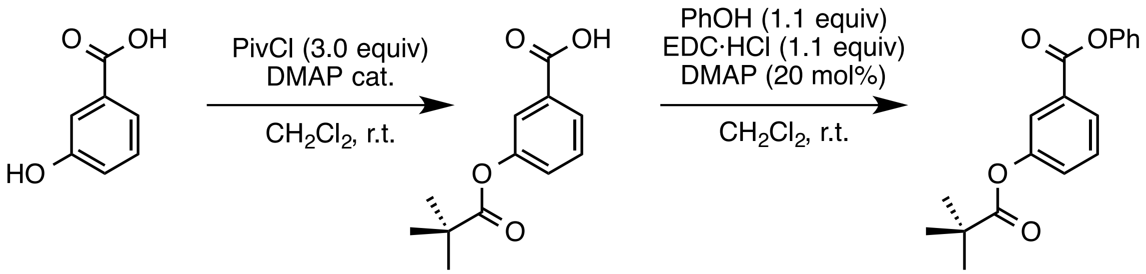


**S5** **4**

To a solution of 3-hydroxybenzoic acid (1.38 g, 10 mmol) and DMAP (12.0 mg, 0.1 mmol, 1 mol%) in pyridine (12 mL) was added pivaloyl chloride (3.62 g, 30 mmol, 3.0 equiv) at 0 °C. This solution was allowed to warm to room temperature and stirred for 1 h. To the mixture was carefully added water (30 mL) and stirred overnight at room temperature. This solution was extracted three times with $CH_2Cl_2$. The combined organic layer was washed with 2.0M $H_2SO_4$ (20 mL) three times. The organic layer was dried over $Na_2SO_4$ and filtrated. The filtrate was concentrated *in vacuo* to afford 3-(pivaloyloxy)benzoic acid (**S5**: 2.15 g, 97% yield) as a white solid.

A mixture of **S5** (2.15 g, 9.7 mmol, 1.0 equiv), phenol (1.0 g, 10.7 mmol, 1.1 equiv), 1-(3-dimethylaminopropyl)-3-ethylcarbodiimide hydrochloride (EDC·HCl: 2.0 g, 10.7 mmol, 1.1 equiv), and *N,N*-dimethylaminopyridine (DMAP: 237 mg, 1.9 mmol, 0.2 equiv) in $CH_2Cl_2$ (20 mL) was stirred for 3 h at room temperature. After quenching the reaction with $NaHCO_3$aq, the mixture was extracted three times with $CH_2Cl_2$. The combined organic layer was dried over $Na_2SO_4$, filtrated, and then concentrated *in vacuo*. The residue was purified by flash column chromatography (hexane/EtOAc = 20:1) to afford phenyl 3-(pivaloyloxy)benzoate **4** as a white solid (2.56 g, 88% yield). $^1$H NMR ($CDCl_3$, 400 MHz) δ 8.07 (d, *J* = 8.0 Hz, 1H), 7.88 (s, 1H), 7.52 (t, *J* = 8.0 Hz, 1H), 7.43 (t, *J* = 8.0 Hz, 2H), 7.34 (d, *J* = 8.0 Hz, 1H), 7.27 (t, *J* = 8.0 Hz, 1H), 7.20 (d, *J* = 8.0 Hz, 2H), 1.36 (s, 9H); $^{13}$C NMR ($CDCl_3$, 100 MHz) δ 176.9, 164.3, 151.2, 150.8, 131.0, 129.54, 129.50, 127.4, 127.0, 126.0, 123.3, 121.6, 39.1, 27.1; HRMS (DART) *m*/*z* calcd for $C_{18}H_{19}O_4$ $[M+H]^+$: 299.1283, found: 299.1285.

## 3. Procedure for the Ni-Catalyzed Decarbonylative Cross-Coupling of 1 and 2

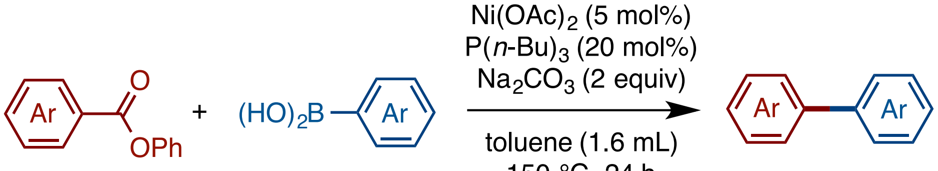


**1** (0.40 mmol) **2** (1.5 equiv) **3**

**General Procedure:** A 20-mL glass vessel equipped with J. Young® O-ring tap containing a magnetic stirring bar and $Ni(OAc)_2 \cdot 4H_2O$ (5.0 mg, 0.020 mmol, 5 mol%) was dried with a heatgun under reduced pressure and filled with $N_2$ gas after cooling to room temperature. To this vessel was added phenyl arenecarboxylic acid phenyl ester **1** (0.40 mmol, 1.0 equiv), arylboronic acid **2** (0.60 mmol, 1.5 equiv), and $Na_2CO_3$ (84.8 mg, 0.8 mmol, 2.0 equiv). The vessel was vacuumed and refilled $N_2$ gas three times. To this was added P(*n*-Bu)$_3$ (19.0 mL, 0.08 mmol, 20 mol%) and toluene (1.6 mL). The vessel was sealed with O-ring tap and then heated at 150 °C for 24 h in an 8-well reaction block with stirring. After cooling the reaction mixture to room temperature, the mixture was passed through a short silica gel pad with EtOAc. The filtrate was concentrated and the residue was purified by flash column chromatography or PTLC to afford the corresponding cross-coupling product **3**.

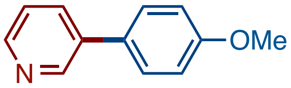


**3Aa**

### 3-(4-Methoxyphenyl)pyridine (3Aa)[9]

Purification by flash column chromatography (hexane/$Et_2O$ = 2:1) afforded **3Aa** as a white solid (using 5 mol% catalyst, for 24 h; 69.7 mg, 95% yield: using 3 mol% catalyst, for 48 h; 73.4 mg, >99% yield). $^1$H NMR ($CDCl_3$, 400 MHz) δ 8.82 (d, *J* = 2.4 Hz, 1H), 8.55 (d, *J* = 4.8 Hz, 1H), 7.83 (dd, *J* = 8.0, 2.4 Hz, 1H), 7.53 (d, *J* = 9.2 Hz, 2H), 7.34 (dd, *J* = 8.0, 4.8 Hz, 1H), 7.02 (d, *J* = 9.2 Hz, 2H), 3.87 (s, 3H); $^{13}$C NMR ($CDCl_3$, 100 MHz) δ 159.7, 147.9, 147.8, 136.2, 133.8, 130.2, 128.1, 123.4, 114.5, 55.3; HRMS (ESI) *m*/*z* calcd for $C_{12}H_{12}NO$ $[M+H]^+$: 186.0913, found: 186.0912.

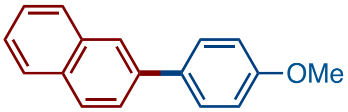


**3Ba**

**2-(4-Methoxyphenyl)naphthalene (3Ba)**[10]

Purification by flash column chromatography (hexane/$CHCl_3$ = 5:1) afforded **3Ba** as a white solid (84.9 mg, 91% yield). $^1$H NMR ($CDCl_3$, 400 MHz) δ 7.98 (s, 1H), 7.91–7.82 (m, 3H), 7.71 (dd, $J$ = 8.4, 1.6 Hz, 1H), 7.65 (d, $J$ = 8.8 Hz, 2H), 7.52–7.42 (m, 2H), 7.01 (d, $J$ = 8.8 Hz, 2H), 3.86 (s, 3H); $^{13}$C NMR ($CDCl_3$, 100 MHz) δ 159.2, 138.1, 133.7, 133.6, 132.3, 128.4, 128.3, 128.0, 127.6, 126.2, 125.6, 125.4, 125.0, 114.3, 55.4; HRMS (DART) $m/z$ calcd for $C_{17}H_{15}O$ $[M+H]^+$: 235.1123, found: 235.1120.

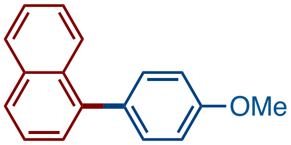


**3Ca**

**1-(4-Methoxyphenyl)naphthalene (3Ca)**[11]

Purification by flash column chromatography (hexane/EtOAc = 100:1) afforded **3Ca** as a white solid (90.2 mg, 96% yield). $^1$H NMR ($CDCl_3$, 400 MHz) δ 7.92 (d, $J$ = 8.4 Hz, 1H), 7.86 (d, $J$ = 8.0 Hz, 1H), 7.80 (d, $J$ = 8.0 Hz, 1H), 7.85–7.35 (m, 6H), 6.99 (d, $J$ = 8.8 Hz, 2H), 3.83 (s, 3H); $^{13}$C NMR ($CDCl_3$, 100 MHz) δ 158.9, 139.9, 133.8, 133.1, 131.8, 131.1, 128.2, 127.3, 126.9, 126.0, 125.9, 125.7, 125.4, 113.7, 55.3; HRMS (DART) $m/z$ calcd for $C_{17}H_{15}O$ $[M+H]^+$: 235.1123, found: 235.1122.

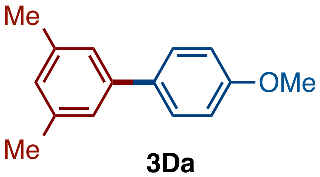


**3Da**

**4'-Methoxy-3,5-dimethyl-1,1'-biphenyl (3Da)** [12]

Purification by flash column chromatography (hexane/EtOAc = 100:1) afforded **3Da** as colorless liquid (63.8 mg, 75% yield). $^1$H NMR ($CDCl_3$, 400 MHz) δ 7.49 (d, $J$ = 9.2 Hz, 2H), 7.16 (s, 2H), 6.97–6.91 (m, 3H), 3.81 (s, 3H), 2.35 (s, 6H); $^{13}$C NMR ($CDCl_3$, 100 MHz) δ 159.0, 140.8, 138.1, 133.9, 128.3, 128.1, 124.6, 114.0, 55.2, 21.4; HRMS (DART) $m/z$ calcd for $C_{15}H_{17}O$ $[M+H]^+$: 213.1279, found: 213.1277.

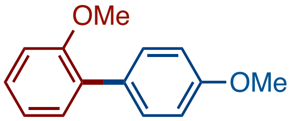


**3Ea**

**2,4'-Dimethoxy-1,1'-biphenyl (3Ea)**[13]

Purification by PTLC (hexane/EtOAc= 20:1) afforded **3Ea** as colorless oil (66.1 mg, 77% yield). $^1$H NMR ($CDCl_3$, 400 MHz) δ 7.46 (d, $J$ = 8.8 Hz, 2H), 7.32–7.25 (m, 2H), 7.00 (dd, $J$ =

8.0, 7.2 Hz, 1H), 6.97–6.91 (m, 3H), 3.82 (s, 3H), 3.79 (s, 3H); $^{13}C$ NMR ($CDCl_3$, 100 MHz) δ 158.6, 156.4, 130.8, 130.63, 130.55, 130.2, 128.1, 120.8, 113.4, 111.1, 55.5, 55.2; HRMS (DART) *m/z* calcd for $C_{14}H_{15}O_2$ $[M+H]^+$: 215.1072, found: 215.1076.

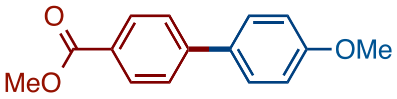


**3Fa**

**Methyl 4'-methoxy-[1,1'-biphenyl]-4-carboxylate (3Fa)**[14]

Purification by flash column chromatography (hexane/$CHCl_3$ = 4:1 to 1:1) afforded **3Fa** as a white solid (85.0 mg, 88% yield). $^1H$ NMR ($CDCl_3$, 400 MHz) δ 8.07 (d, *J* = 8.4 Hz, 2H), 7.61 (d, *J* = 8.4 Hz, 2H), 7.56 (d, *J* = 8.4 Hz, 2H), 6.98 (d, *J* = 8.4 Hz, 2H), 3.92 (s, 3H), 3.85 (s, 3H); $^{13}C$ NMR ($CDCl_3$, 100 MHz) δ 167.0, 159.8, 145.1, 132.3, 130.0, 128.3, 128.2, 126.4, 114.3, 55.3, 52.0; HRMS (DART) *m/z* calcd for $C_{15}H_{15}O_3$ $[M+H]^+$: 243.1021, found: 243.1019.

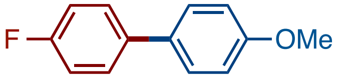


**3Ga**

**4-Fluoro-4'-methoxy-1,1'-biphenyl (3Ga)**[9]

Purification by flash column chromatography (hexane/$CHCl_3$ = 10:1 to 4:1) afforded **3Ga** as a white solid (68.9 mg, 85% yield). $^1H$ NMR ($CDCl_3$, 400 MHz) δ 7.51–7.43 (m, 4H), 7.12–7.06 (m, 2H), 6.97 (d, *J* = 8.8 Hz, 2H), 3.86 (s, 3H); $^{13}C$ NMR ($CDCl_3$, 100 MHz) δ 162.1 (d, $J_{C\text{–}F}$ = 249 Hz), 159.1, 136.9 (d, $J_{C\text{–}F}$ = 4.0 Hz), 132.8, 128.2 (d, $J_{C\text{–}F}$ = 8.0 Hz), 128.0, 115.5 (d, $J_{C\text{–}F}$ = 21 Hz), 114.2, 55.3; HRMS (DART) *m/z* calcd for $C_{13}H_{12}FO$ $[M+H]^+$: 203.0872, found: 203.0870.

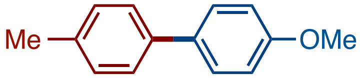


**3Ha**

**4-Methoxy-4'-methyl-1,1'-biphenyl (3Ha)**[15]

Purification by flash column chromatography (hexane/$CHCl_3$ = 10:1 to 4:1) afforded **3Ha** as a white solid (70.3 mg, 89% yield). $^1H$ NMR ($CDCl_3$, 400 MHz) δ 7.50 (d, *J* = 8.8 Hz, 2H), 7.44 (d, *J* = 8.0 Hz, 2H), 7.22 (d, *J* = 8.0 Hz, 2H), 6.95 (d, *J* = 8.8 Hz, 2H), 3.84 (s, 3H), 2.38 (s, 3H); $^{13}C$ NMR ($CDCl_3$, 100 MHz) δ 158.9, 137.9, 136.3, 133.7, 129.4, 127.9, 126.6, 114.1, 55.3, 21.0; HRMS (DART) *m/z* calcd for $C_{14}H_{15}O$ $[M+H]^+$: 199.1123, found: 199.1121.

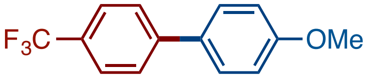


**3Ia**

**4-Methoxy-4'-(trifluoromethyl)-1,1'-biphenyl (3Ia)**[9]

The reaction was conducted at 160 °C. Purification by flash column chromatography (hexane/$CHCl_3$ = 4:1) afforded **3Ia** as a white solid (99.5 mg, 99% yield). $^1H$ NMR ($CDCl_3$, 400 MHz) δ 7.67–7.62 (m, 4H), 7.53 (d, *J* = 8.8 Hz, 2H), 6.99 (d, *J* = 8.8 Hz, 2H), 3.85 (s, 3H); $^{13}C$

NMR ($CDCl_3$, 100 MHz) δ 159.8, 144.3, 132.1, 128.7 (q, $J_{C\text{–}F}$ = 33 Hz), 128.3, 126.8, 125.7 (q, $J_{C\text{–}F}$ = 4 Hz), 124.4 (q, $J_{C\text{–}F}$ = 276 Hz), 114.4, 55.3; HRMS (DART) *m*/*z* calcd for $C_{14}H_{12}F_3O$ $[M+H]^+$: 253.0840, found: 253.0838.

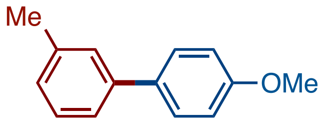

**3Ja**

**4'-Methoxy-3-methyl-1,1'-biphenyl (3Ja)**[16]

Purification by PTLC (hexane/EtOAc = 20:1) afforded **3Ja** as a white solid (57.8 mg, 73% yield). $^1$H NMR ($CDCl_3$, 400 MHz) δ 7.51 (d, *J* = 9.2 Hz, 2H), 7.38–7.25 (m, 3H), 7.10 (d, *J* = 7.6 Hz, 1H), 6.94 (d, *J* = 9.2 Hz, 2H), 3.80 (s, 3H), 2.39 (s, 3H); $^{13}$C NMR ($CDCl_3$, 100 MHz) δ 159.0, 140.7, 138.2, 133.8, 128.6, 128.1, 127.5, 127.4, 123.8, 114.1, 55.2, 21.5; HRMS (DART) *m*/*z* calcd for $C_{14}H_{15}O$ $[M+H]^+$: 199.1123, found: 199.1120.

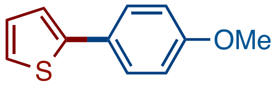

**3Ka**

**2-(4-Methoxyphenyl)thiophene (3Ka)**[12]

Purification by PTLC (hexane/EtOAc = 30:1) afforded **3Ka** as a white solid (59.3 mg, 78% yield). $^1$H NMR ($CDCl_3$, 400 MHz) δ 7.53 (d, *J* = 8.4 Hz, 2H), 7.21–7.16 (m, 2H), 7.03 (d, *J* = 4.4 Hz, 1H), 6.89 (d, *J* = 8.4 Hz, 2H), 3.81 (s, 3H); $^{13}$C NMR ($CDCl_3$, 100 MHz) δ 159.1, 144.3, 127.9, 127.23, 127.16, 123.8, 122.0, 114.2, 55.3; HRMS (ESI) *m*/*z* calcd for $C_{11}H_{11}OS$ $[M+H]^+$: 191.0525, found: 191.0522.

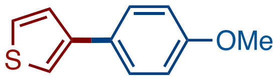

**3La**

**3-(4-Methoxyphenyl)thiophene (3La)**[17]

Purification by flash column chromatography (hexane/EtOAc = 100:1) afforded **3La** as a white solid (50.6 mg, 66% yield). $^1$H NMR ($CDCl_3$, 400 MHz) δ 7.52 (d, *J* = 7.6 Hz, 2H), 7.38–7.30 (m, 3H), 6.92 (d, *J* = 7.6 Hz, 2H), 3.84 (s, 3H); $^{13}$C NMR ($CDCl_3$, 100 MHz) δ 158.8, 142.0, 128.7, 127.5, 126.2, 126.0, 118.9, 114.1, 55.3; HRMS (DART) *m*/*z* calcd for $C_{11}H_{10}OS$ $[M+H]^+$: 191.0531, found: 191.0533.

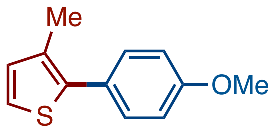

**3Ma**

**2-(4-Methoxyphenyl)-3-methylthiophene (3Ma)**

Purification by flash column chromatography (hexane/EtOAc = 100:1) afforded **3Ma** as colorless liquid (49.4 mg, 60% yield). $^1$H NMR ($CDCl_3$, 400 MHz) δ 7.37 (d, *J* = 7.6 Hz, 2H),

7.13 (d, $J$ = 5.6 Hz, 1H), 6.93 (d, $J$ = 7.6 Hz, 2H), 6.88 (d, $J$ = 5.6 Hz, 1H), 3.81 (s, 3H), 2.28 (s, 3H); $^{13}$C NMR ($CDCl_3$, 100 MHz) δ 158.8, 137.6, 132.5, 130.9, 130.2, 127.1, 122.6, 113.9, 55.2, 14.8; HRMS (ESI) $m/z$ calcd for $C_{12}H_{13}OS$ $[M+H]^+$: 205.0682, found: 205.0680

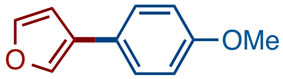

**3Na**

**3-(4-Methoxyphenyl)furan (3Na)**[12]

Purification by flash column chromatography (hexane/EtOAc = 100:1) afforded **3Na** as a white solid (46.9 mg, 67% yield). $^1$H NMR ($CDCl_3$, 400 MHz) δ 7.65 (d, $J$ = 1.6 Hz, 1H), 7.44 (t, $J$ = 1.6 Hz, 1H), 7.40 (d, $J$ = 8.8 Hz, 2H), 6.91 (d, $J$ = 8.8 Hz, 2H), 6.64 (d, $J$ = 1.6 Hz, 1H), 3.81 (s, 3H); $^{13}$C NMR ($CDCl_3$, 100 MHz) δ 158.7, 143.5, 137.6, 127.0, 126.0, 125.0, 114.2, 108.8, 55.3; HRMS (ESI) $m/z$ calcd for $C_{11}H_{11}O_2$ $[M+H]^+$: 175.0754, found: 175.0752.

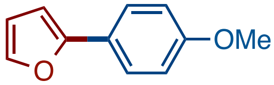

**3Oa**

**2-(4-Methoxyphenyl)furan (3Oa)**[18]

The reaction was conducted using 1.2 equiv of $Na_2CO_3$. Purification by flash column chromatography (hexane/EtOAc = 100:1) afforded **3Oa** as a white solid (51.0 mg, 73% yield). $^1$H NMR ($CDCl_3$, 400 MHz) δ 7.60 (d, $J$ = 8.8 Hz, 2H), 7.41 (d, $J$ = 1.6 Hz, 1H), 6.91 (d, $J$ = 8.8 Hz, 2H), 6.50 (d, $J$ = 3.2 Hz, 1H), 6.43 (dd, $J$ = 3.2, 1.6 Hz, 1H), 3.81 (s, 3H); $^{13}$C NMR ($CDCl_3$, 100 MHz) δ 159.0, 154.0, 141.3, 125.2, 124.0, 114.0, 111.5, 103.3, 55.3; HRMS (ESI) $m/z$ calcd for $C_{11}H_{11}O_2$ $[M+H]^+$: 175.0754, found: 175.0752.

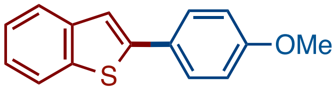

**3Pa**

**2-(4-Methoxyphenyl)benzo[*b*]thiophene (3Pa)**[19]

Purification by precipitation of the crude mixture in $Et_2O$ and filtration of the suspension afforded **3Pa** as a white solid (91.7 mg, 95% yield). $^1$H NMR ($CDCl_3$, 400 MHz) δ 7.80 (d, $J$ = 8.0 Hz, 1H), 7.74 (d, $J$ = 7.2 Hz, 1H), 7.64 (d, $J$ = 8.8 Hz, 2H), 7.42 (s, 1H), 7.36–7.25 (m, 2H), 6.96 (d, $J$ = 8.8 Hz, 2H), 3.85 (s, 3H); $^{13}$C NMR ($CDCl_3$, 100 MHz) δ 159.8, 144.1, 140.9, 139.1, 127.7, 127.0, 124.4, 123.9, 123.2, 122.2, 118.2, 114.3, 55.4; HRMS (ESI) $m/z$ calcd for $C_{15}H_{13}OS$ $[M+H]^+$: 241.0682, found: 241.0678.

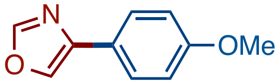

**3Qa**

**4-(4-Methoxyphenyl)oxazole (3Qa)**[20]

Purification by PTLC (hexane/EtOAc = 5:1) afforded **3Qa** as a white solid (35.5 mg, 50% yield). $^1$H NMR ($CDCl_3$, 400 MHz) δ 7.91 (s, 1H), 7.86 (s, 1H), 7.68 (d, $J$ = 8.4 Hz, 2H), 6.95 (d,

*J* = 8.4 Hz, 2H), 3.85 (s, 3H); $^{13}$C NMR ($CDCl_3$, 100 MHz) δ 159.6, 151.2, 140.2, 132.6, 126.9, 123.4, 114.2, 55.3; HRMS (ESI) *m*/*z* calcd for $C_{10}H_{10}NO_2$ $[M+H]^+$: 176.0706, found: 176.0704.

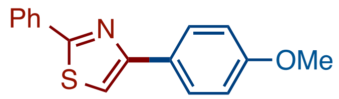

**3Ra**

**4-(4-Methoxyphenyl)-2-phenylthiazole (3Ra)**[21]

The reaction was conducted at 160 °C. Purification by PTLC (hexane/$CHCl_3$ = 1:1) afforded **3Ra** as a white solid (86.8 mg, 81% yield). $^1$H NMR ($CDCl_3$, 400 MHz) δ 8.03 (dd, *J* = 8.4, 2.0 Hz, 2H), 7.92 (d, *J* = 8.4 Hz, 2H), 7.47–7.38 (m, 3H), 7.31 (s, 1H), 6.96 (d, *J* = 7.2 Hz, 2H), 3.84 (s, 3H); $^{13}$C NMR ($CDCl_3$, 100 MHz) δ 167.6, 159.6, 156.0, 133.8, 129.9, 128.9, 127.7, 127.5, 126.5, 114.0, 110.9, 55.3; HRMS (DART) *m*/*z* calcd for $C_{16}H_{14}NOS$ $[M+H]^+$: 268.0796, found: 268.0795.

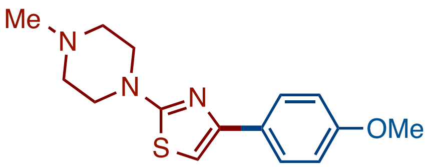

**3Sa**

**4-(4-Methoxyphenyl)-2-(4-methylpiperazin-1-yl)thiazole (3Sa)**

Purification by PTLC ($CHCl_3$/MeOH = 50:1) afforded **3Sa** as a yellow solid (54.5 mg, 47% yield). $^1$H NMR ($CDCl_3$, 400 MHz) δ 7.76 (d, *J* = 8.4 Hz, 2H), 6.90 (d, *J* = 8.4 Hz, 2H), 6.62 (s, 1H), 3.82 (s, 3H), 3.56 (t, *J* = 5.2 Hz, 4H), 2.54 (t, *J* = 5.2 Hz, 4H), 2.35 (s, 3H); $^{13}$C NMR ($CDCl_3$, 100 MHz) δ 170.9, 159.2, 151.6, 128.1, 127.3, 113.8, 99.6, 55.2, 54.5, 48.2, 46.2; HRMS (DART) *m*/*z* calcd for $C_{15}H_{20}N_3OS$ $[M+H]^+$: 290.1327, found: 290.1329.

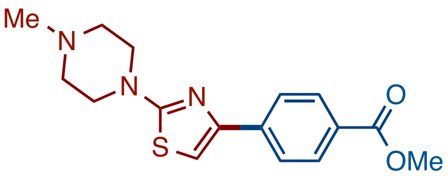

**3Si**

**Methyl 4-(2-(4-methylpiperazin-1-yl)thiazol-4-yl)benzoate (3Si)**

The reaction was conducted at 160 °C. Purification by PTLC ($CHCl_3$/MeOH = 50:1) afforded **3Si** as a brown solid (63.2 mg, 51% yield). $^1$H NMR ($CDCl_3$, 400 MHz) δ 8.03 (d, *J* = 8.4 Hz, 2H), 7.90 (d, *J* = 8.4 Hz, 2H), 6.91 (s, 1H), 3.92 (s, 3H), 3.59 (t, *J* = 5.2 Hz, 4H), 2.55 (t, *J* = 5.2 Hz, 4H), 2.36 (s, 3H); $^{13}$C NMR ($CDCl_3$, 100 MHz) δ 171.0, 167.0, 150.8, 139.1, 129.9, 128.8, 125.8, 103.7, 54.2, 52.0, 48.3, 46.2; HRMS (DART) *m*/*z* calcd for $C_{16}H_{20}N_3O_2S$ $[M+H]^+$: 318.1276, found: 318.1275.

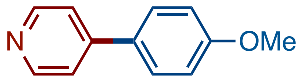

**3Ta**

**4-(4-Methoxyphenyl)pyridine (3Ta)**[22]

The reaction was conducted using 1.5 equiv of $Et_3N$ instead of $Na_2CO_3$. Purification by flash column chromatography (hexane/$Et_2O$ = 1:1) afforded **3Ta** as a white solid (69.7 mg, 94% yield). $^1$H NMR ($CDCl_3$, 600 MHz) δ 8.62 (d, *J* = 6.4 Hz, 2H), 7.59 (d, *J* = 8.4 Hz, 2H), 7.46 (d, *J* = 6.4 Hz, 2H), 7.00 (d, *J* = 8.4 Hz, 2H), 3.85 (s, 3H); $^{13}$C NMR ($CDCl_3$, 150 MHz) δ 160.5, 150.1, 147.7, 130.3, 128.1, 121.0, 114.5, 55.3; HRMS (ESI) *m*/*z* calcd for $C_{12}H_{12}NO$ $[M+H]^+$: 186.0913, found: 186.0910.

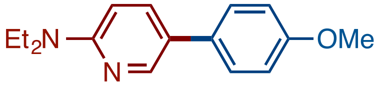


**3Ua**

***N*,*N*-Diethyl-5-(4-methoxyphenyl)pyridin-2-amine (3Ua)**

Purification by PTLC (hexane/EtOAc = 10:1) and then GPC afforded **3Ua** as a white solid (64.3 mg, 63% yield). $^1$H NMR ($CDCl_3$, 400 MHz) δ 8.37 (d, *J* = 2.4 Hz, 1H), 7.60 (dd, *J* = 9.2, 2.4 Hz, 1H), 7.41 (d, *J* = 9.2 Hz, 2H), 6.95 (d, *J* = 9.2 Hz, 2H), 6.51 (d, *J* = 9.2 Hz, 1H), 3.82 (s, 3H), 3.52 (q, *J* = 7.2 Hz, 4H), 1.20 (t, *J* =7.2 Hz, 6H); $^{13}$C NMR ($CDCl_3$, 100 MHz) δ 158.4, 156.4, 145.9, 135.5, 131.4, 126.9, 123.6, 114.2, 105.3, 55.3, 42.5, 13.0; HRMS (DART) *m*/*z* calcd for $C_{16}H_{21}N_2O$ $[M+H]^+$: 257.1654, found: 257.1658.

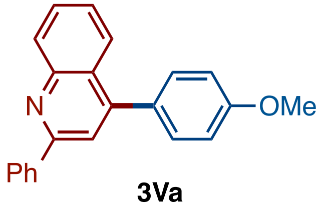


**3Va**

**4-(4-Methoxyphenyl)-2-phenylquinoline (3Va)**[23]

The reaction was conducted using 1.5 equiv of $Et_3N$ instead of $Na_2CO_3$. Purification by flash column chromatography (hexane/EtOAc = 30:1) afforded **3Va** as a white solid (125.6 mg, >99% yield). $^1$H NMR ($CDCl_3$, 600 MHz) δ 8.23 (d, *J* = 8.4 Hz, 1H), 8.18 (d, *J* = 7.2 Hz, 2H), 7.94 (d, *J* = 8.4 Hz, 1H), 7.79 (s, 1H), 7.71 (td, *J* = 8.4, 1.2 Hz, 1H), 7.54–7.42 (m, 6H), 7.07, (d, *J* = 8.4 Hz, 2H), 3.90 (s, 3H); $^{13}$C NMR ($CDCl_3$, 150 MHz) δ 159.8, 156.9, 148.9, 148.8, 139.7, 130.8, 130.6, 130.1, 129.4, 129.2, 128.8, 127.5, 126.2, 125.9, 125.6, 119.3, 114.0, 55.4; HRMS (DART) *m*/*z* calcd for $C_{22}H_{18}NO$ $[M+H]^+$: 312.1388, found: 312.1389.

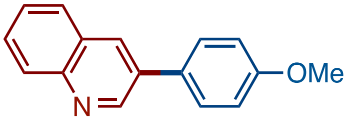


**3Wa**

**3-(4-Methoxyphenyl)quinoline (3Wa)**[13]

Purification by PTLC (hexane/EtOAc = 5:1) afforded **3Wa** as a white solid (98.7 mg, >99% yield). $^1$H NMR ($CDCl_3$, 400 MHz) δ 9.16 (d, *J* = 2.4 Hz, 1H), 8.21 (d, *J* = 2.4 Hz, 1H), 8.12 (d, *J* = 8.4 Hz, 1H), 7.83 (d, *J* = 8.4 Hz, 1H), 7.68 (dd, *J* = 8.4, 7.8 Hz, 1H), 7.63 (d, *J* = 9.2 Hz, 2H), 7.54 (dd, *J* = 8.4, 7.8 Hz, 1H), 7.03 (d, *J* = 9.2 Hz, 2H), 3.85 (s, 3H); $^{13}$C NMR ($CDCl_3$, 100 MHz) δ 159.7, 149.8, 146.9, 133.3, 132.3, 130.2, 129.1, 129.0, 128.4, 128.0, 127.8, 126.8, 114.6, 55.3; HRMS (ESI) *m*/*z* calcd for $C_{16}H_{14}NO$ $[M+H]^+$: 236.1070, found: 236.1073.

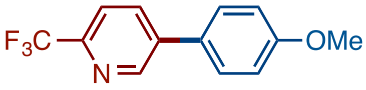

**3Xa**

**5-(4-Methoxyphenyl)-2-(trifluoromethyl)pyridine (3Xa)**

The reaction was conducted using 1.5 equiv of $Et_3N$ instead of $Na_2CO_3$. Purification by flash column chromatography (hexane/EtOAc = 10:1) afforded **3Xa** as a white solid (89 mg, 88% yield). $^1H$ NMR ($CDCl_3$, 400 MHz) δ 8.90 (s, 1H), 7.98 (d, $J$ = 8.4 Hz, 1H), 7.71 (d, $J$ = 8.4 Hz, 1H), 7.54 (d, $J$ = 9.0 Hz, 2H), 7.03 (d, $J$ = 9.0 Hz, 2H), 3.87 (s, 3H); $^{13}C$ NMR ($CDCl_3$, 100 MHz) δ 160.4, 148.0, 146.0 (q, $J_{C-F}$ = 35 Hz), 139.0, 134.7, 128.6, 128.4, 121.7 (q, $J_{C-F}$ = 275 Hz), 120.3, 114.8, 55.3; HRMS (DART) $m/z$ calcd for $C_{13}H_{11}F_3NO$ $[M+H]^+$: 254.0793, found: 254.0790.

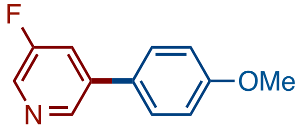

**3Ya**

**3-Fluoro-5-(4-methoxyphenyl)pyridine (3Ya)**

Purification by PTLC (hexane/EtOAc = 4:1) afforded **3Ya** as a white solid (75.2 mg, 92% yield). $^1H$ NMR ($CDCl_3$, 400 MHz) δ 8.62 (s, 1H), 8.39 (s, 1H), 7.54–7.47 (m, 3H), 6.99 (d, $J$ = 8.8 Hz, 2H), 3.84 (s, 3H); $^{13}C$ NMR ($CDCl_3$, 100 MHz) δ 160.0, 159.6 (d, $J_{C-F}$ = 260 Hz), 143.6 (d, $J_{C-F}$ = 4 Hz), 137.7 (d, $J_{C-F}$ = 4 Hz), 135.7 (d, $J_{C-F}$ = 24 Hz), 128.5, 128.2, 120.2 (d, $J_{C-F}$ = 19 Hz), 114.5, 55.2; HRMS (ESI) $m/z$ calcd for $C_{12}H_{11}FNO$ $[M+H]^+$: 204.0819, found: 204.0820.

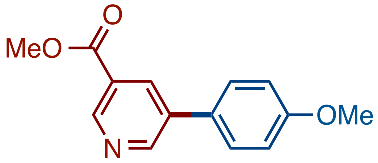

**3Za**

**Methyl 5-(4-methoxyphenyl)nicotinate (3Za)**

The reaction was conducted using 1.2 equiv of $Na_2CO_3$. Purification by flash column chromatography (hexane/EtOAc = 4:1) afforded **3Za** as a white solid (85.8 mg, 88% yield). $^1H$ NMR ($CDCl_3$, 400 MHz) δ 9.14 (s, 1H), 8.96 (d, $J$ = 2.0 Hz, 1H), 8.43 (d, $J$ = 2.0 Hz, 1H), 7.56 (d, $J$ = 8.0 Hz, 2H), 7.02 (d, $J$ = 8.0 Hz, 2H), 3.98 (s, 3H), 3.86 (s, 3H); $^{13}C$ NMR ($CDCl_3$, 100 MHz) δ 165.8, 160.0, 151.3, 148.6, 135.9, 134.5, 128.8, 128.2, 125.8, 114.6, 55.3, 52.4; HRMS (DART) $m/z$ calcd for $C_{14}H_{14}NO_3$ $[M+H]^+$: 244.0974, found: 244.0971.

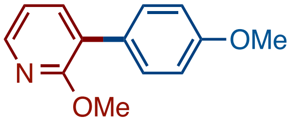

**3AAa**

**2-Methoxy-3-(4-methoxyphenyl)pyridine (3AAa)**[24]

Purification by PTLC (hexane/EtOAc = 10:1) afforded **3AAa** as a white solid (72.4 mg, 84% yield). $^1H$ NMR ($CDCl_3$, 400 MHz) δ 8.12 (dd, $J$ = 4.8, 1.2 Hz, 1H), 7.57 (dd, $J$ = 8.0, 1.2

Hz, 1H), 7.50 (d, $J$ = 8.0 Hz, 2H), 6.99–6.92 (m, 3H), 3.97 (s, 3H), 3.83 (s, 3H); $^{13}$C NMR ($CDCl_3$, 100 MHz) δ 160.8, 159.0, 145.1, 138.1, 130.2, 129.0, 124.3, 117.1, 113.6, 55.2, 53.5; HRMS (ESI) $m/z$ calcd for $C_{13}H_{14}NO_2$ $[M+H]^+$: 216.1019, found: 216.1020.

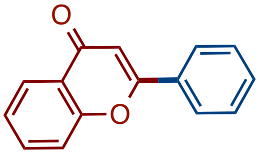


**3ABh**

**2-Phenyl-4*H*-chromen-4-one (3ABh)**[25]

Purification by flash column chromatography (hexane/EtOAc = 6:1) afforded **3ABh** as a white solid (80.0 mg, 90% yield). $^1$H NMR ($CDCl_3$, 400 MHz) δ 8.22 (d, $J$ = 8.0 Hz, 1H), 7.91 (d, $J$ = 7.6 Hz, 2H), 7.68 (dd, $J$ = 8.0, 7.6 Hz, 1H), 7.60–7.46 (m, 4H), 7.40 (dd, $J$ = 8.0, 7.6 Hz, 1H), 6.81 (s, 1H); $^{13}$C NMR ($CDCl_3$, 100 MHz) δ 178.3, 163.2, 156.1, 133.7, 131.6, 131.5, 128.9, 126.2, 125.6, 125.1, 123.8, 118.0, 107.5; HRMS (ESI) $m/z$ calcd for $C_{15}H_{11}O$ $[M+H]^+$: 223.0754, found: 223.0756.

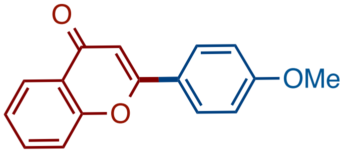


**3ABa**

**2-(4-Methoxyphenyl)-4*H*-chromen-4-one (3ABa)**[25]

Purification by PTLC (hexane/$Et_2O$ = 1:1, hexane/EtOAc= 3:1, and then $CHCl_3$) afforded **3ABa** as a white solid (100.8 mg, >99% yield). $^1$H NMR ($CDCl_3$, 400 MHz) δ 8.21 (d, $J$ = 7.6 Hz, 1H), 7.86 (d, $J$ = 8.0 Hz, 2H), 7.66 (dd, $J$ = 7.6, 7.2 Hz, 1H), 7.52 (d, $J$ = 7.6 Hz, 1H), 7.39 (dd, $J$ = 7.6, 7.2 Hz, 1H), 6.99 (d, $J$ = 8.0 Hz, 2H), 6.72 (s, 1H), 3.86 (s, 3H); $^{13}$C NMR ($CDCl_3$, 100 MHz) δ 178.2, 163.3, 162.3, 156.1, 133.5, 127.9, 125.5, 125.0, 123.9, 117.9, 114.4, 106.1, 55.4; HRMS (DART) $m/z$ calcd for $C_{16}H_{13}O_3$ $[M+H]^+$: 253.0865, found: 253.0861.

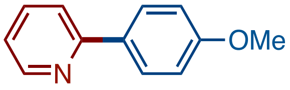


**3ACa**

**2-(4-Methoxyphenyl)pyridine (3ACa)**[26]

Purification by flash column chromatography (hexane/$Et_2O$ = 5:1 to 7:3) afforded **3ACa** as a white solid (9.0 mg, 12% yield). $^1$H NMR ($CDCl_3$, 400 MHz) δ 8.66 (d, $J$ = 4.4 Hz, 1H), 7.95 (d, $J$ = 8.4 Hz, 2H), 7.75–7.64 (m, 2H), 7.17 (dd, $J$ = 7.2, 4.4 Hz, 1H), 7.00 (d, $J$ = 8.4 Hz, 2H), 3.87 (s, 3H); $^{13}$C NMR ($CDCl_3$, 100 MHz) δ 160.5, 157.1, 149.5, 136.6, 132.0, 128.1, 121.4, 119.8, 114.1, 55.3; HRMS (ESI) $m/z$ calcd for $C_{12}H_{12}NO$ $[M+H]^+$: 186.0913, found: 186.0909.

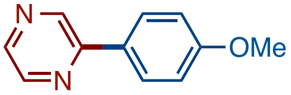


**3ADa**

**2-(4-Methoxyphenyl)pyrazine (3ADa)**[27]

Purification by flash column chromatography (hexane/$Et_2O$ = 1:1) afforded **3ADa** as a white solid (17.0 mg, 23% yield). $^1H$ NMR ($CDCl_3$, 400 MHz) δ 8.98 (s, 1H), 8.58 (d, $J$ = 2.4 Hz, 1H), 8.44 (d, $J$ = 2.4 Hz, 1H), 7.98 (d, $J$ = 7.2 Hz, 2H), 7.03 (d, $J$ = 7.2 Hz, 2H), 3.88 (s, 3H); $^{13}C$ NMR ($CDCl_3$, 100 MHz) δ 161.1, 152.5, 144.0, 142.1, 141.6, 128.8, 128.3, 114.4, 55.4; HRMS (ESI) $m/z$ calcd for $C_{11}H_{11}N_2O$ [M+H]$^+$: 187.0866, found: 187.0864.

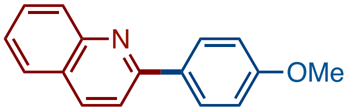


**3AEa**

**2-(4-Methoxyphenyl)quinoline (3AEa)**[28]

Purification by flash column chromatography (hexane/EtOAc = 20:1) afforded **3AEa** as a white solid (34.3 mg, 36% yield). $^1H$ NMR ($CDCl_3$, 400 MHz) δ 8.20–8.11 (m, 4H), 7.83 (d, $J$ = 8.8 Hz, 1H), 7.80 (d, $J$ = 8.8 Hz, 1H), 7.71 (td, $J$ = 7.2, 1.2 Hz, 1H), 7.50 (td, $J$ = 7.2, 1.2 Hz, 1H), 7.05 (d, $J$ = 9.2 Hz, 2H), 3.89 (s, 3H); $^{13}C$ NMR ($CDCl_3$, 100 MHz) δ 160.8, 156.9, 148.3, 136.6, 132.2, 129.6, 129.5, 128.9, 127.4, 126.9, 125.9, 118.5, 114.2, 55.4; HRMS (ESI) $m/z$ calcd for $C_{16}H_{14}NO$ [M+H]$^+$: 236.1070, found: 236.1078.

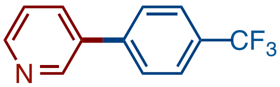


**3Ab**

**3-(4-(Trifluoromethyl)phenyl)pyridine (3Ab)**[29]

Purification by flash column chromatography (hexane/$Et_2O$ = 2:1) afforded **3Ab** as a white solid (64.5 mg, 72% yield). $^1H$ NMR ($CDCl_3$, 400 MHz) δ 8.87 (s, 1H), 8.66 (d, $J$ = 4.0 Hz, 1H), 7.89 (d, $J$ = 8.0 Hz, 1H), 7.74 (d, $J$ = 8.8 Hz, 2H), 7.68 (d, $J$ = 8.8 Hz, 2H), 7.41 (dd, $J$ = 8.0, 4.0 Hz, 1H); $^{13}C$ NMR ($CDCl_3$, 100 MHz) δ 149.3, 148.3, 141.3, 135.2, 134.4, 130.2 (q, $J_{C–F}$ = 32 Hz), 127.4, 126.0 (q, $J_{C–F}$ = 4.0 Hz), 124.0 (q, $J_{C–F}$ = 277 Hz), 123.6; HRMS (ESI) $m/z$ calcd for $C_{12}H_9F_3N$ [M+H]$^+$: 224.0682, found: 224.0684.

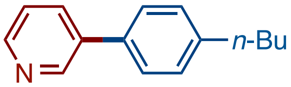


**3Ac**

**3-(4-Butylphenyl)pyridine (3Ac)**[30]

Purification by PTLC (hexane/EtOAc = 4:1) afforded **3Ac** as colorless liquid (63.0 mg, 75% yield). $^1H$ NMR ($CDCl_3$, 400 MHz) δ 8.84 (d, $J$ = 2.4 Hz, 1H), 8.56 (dd, $J$ = 4.8, 2.4 Hz, 1H), 7.87 (dd, $J$ = 8.0, 4.8 Hz, 1H), 7.50 (d, $J$ = 8.0 Hz, 2H), 7.35 (dd, $J$ = 8.0, 4.8 Hz, 1H), 7.29 (d, $J$ = 8.0 Hz, 2H), 2.67 (t, $J$ = 8.0 Hz, 2H), 1.70–1.59 (m, 2H), 1.46–1.32 (m, 2H), 0.95 (t, $J$ = 7.8 Hz, 3H); $^{13}C$ NMR ($CDCl_3$, 100 MHz) δ 148.24, 148.19, 143.0, 136.6, 135.1, 134.1, 129.2, 127.0, 123.5, 35.3, 33.6, 22.4, 13.9; HRMS (ESI) $m/z$ calcd for $C_{15}H_{18}N$ [M+H]$^+$: 212.1434, found: 212.1433.

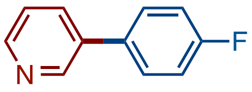

**3Ad**

**3-(4-Fluorophenyl)pyridine (3Ad)**[29]

Purification by flash column chromatography (hexane/EtOAc = 6:1) afforded **3Ad** as a white solid (62.1 mg, 90% yield). $^1$H NMR ($CDCl_3$, 400 MHz) δ 8.81 (s, 1H), 8.59 (d, $J$ = 4.8 Hz, 1H), 7.82 (d, $J$ = 8.0 Hz, 1H), 7.59–7.50 (m, 2H), 7.36 (dd, $J$ = 8.0, 4.8 Hz, 1H), 7.20–7.11 (m, 2H); $^{13}$C NMR ($CDCl_3$, 100 MHz) δ 162.8 (d, $J_{C–F}$ = 249 Hz), 148.4, 148.1, 135.6, 134.1, 133.8, 128.7 (d, $J_{C–F}$ = 8.0 Hz), 123.9, 116.0 (d, $J_{C–F}$ = 21 Hz); HRMS (ESI) *m*/*z* calcd for $C_{11}H_9FN$ $[M+H]^+$: 174.0714, found: 174.0711.

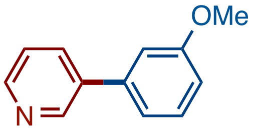

**3Ae**

**3-(3-Methoxyphenyl)pyridine (3Ae)**[31]

Purification by PTLC (hexane/$Et_2O$ = 1:1) afforded **3Ae** as colorless liquid (65.2 mg, 88% yield). $^1$H NMR ($CDCl_3$, 400 MHz) δ 8.85 (s, 1H), 8.59 (d, $J$ = 4.8 Hz, 1H), 7.86 (dd, $J$ = 8.0, 2.4 Hz, 1H), 7.42–7.32 (m, 2H), 7.16 (dd, $J$ = 8.0, 4.8 Hz, 1H), 7.10 (s, 1H), 6.94 (dd, $J$ = 8.0, 2.4 Hz, 1H), 3.86 (s, 3H); $^{13}$C NMR ($CDCl_3$, 100 MHz) δ 160.0, 148.6, 148.3, 139.2, 136.4, 134.3, 130.1, 123.4, 119.5, 113.3, 112.9, 55.3; HRMS (ESI) *m*/*z* calcd for $C_{12}H_{12}ON$ $[M+H]^+$: 186.0913, found: 186.0910.

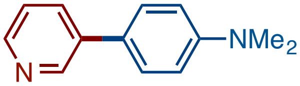

**3Af**

***N*,*N*-Dimethyl-4-(pyridin-3-yl)aniline (3Af)**[32]

The reaction was conducted using 1.5 equiv of $Et_3N$ instead of $Na_2CO_3$. Purification by flash column chromatography (hexane/EtOAc = 4:1) afforded **3Af** as a white solid (39.3 mg, 50% yield). $^1$H NMR ($CDCl_3$, 400 MHz) δ 8.82 (s, 1H), 8.48 (d, $J$ = 4.8 Hz, 1H), 7.83 (d, $J$ = 8.0 Hz, 1H), 7.49 (d. $J$ = 8.8 Hz, 2H), 7.30 (dd, $J$ = 8.0, 4.8 Hz, 1H), 6.82 (d, $J$ = 8.8 Hz, 2H), 3.01 (s, 6H); $^{13}$C NMR ($CDCl_3$, 100 MHz) δ 150.4, 147.7, 147.1, 136.6, 133.2, 127.7, 125.4, 123.4, 112.8, 40.4; HRMS (ESI) *m*/*z* calcd for $C_{13}H_{15}N_2$ $[M+H]^+$: 199.1230, found: 199.1228.

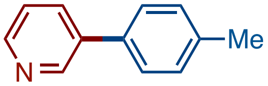

**3Ag**

**3-(4-Methyl)pyridine (3Ag)**[29]

Purification by flash column chromatography (hexane/EtOAc = 6:1) afforded **3Ag** as a white solid (64.7 mg, 96% yield). $^1$H NMR ($CDCl_3$, 400 MHz) δ 8.84 (d, $J$ = 2.0 Hz, 1H), 8.56 (dd, $J$ = 4.8, 2.0 Hz, 1H), 7.85 (dd, $J$ = 8.0, 2.0 Hz, 1H), 7.47 (d, $J$ = 8.4 Hz, 2H), 7.34 (dd, $J$ = 8.0, 4.8 Hz, 1H), 7.28 (d, $J$ = 8.4 Hz, 2H), 2.41 (s, 3H); $^{13}$C NMR ($CDCl_3$, 100 MHz) δ 148.1

(one carbon overlap), 138.0, 136.5, 134.9, 134.1, 129.7, 126.9, 123.5, 21.1; HRMS (ESI) *m*/*z* calcd for $C_{12}H_{12}N$ $[M+H]^+$: 170.0964, found: 170.0961.

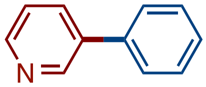


**3Ah**

**3-Phenylpyridine (3Ah)**[29]

Purification by flash column chromatography (hexane/EtOAc = 4:1) afforded **3Ah** as colorless oil (50.1 mg, 81% yield). $^1H$ NMR ($CDCl_3$, 400 MHz) δ 8.86 (d, *J* = 2.4 Hz, 1H), 8.59 (d, *J* = 5.2 Hz, 1H), 7.87 (dd, *J* = 8.0, 2.4 Hz, 1H), 7.58 (d, *J* = 8.0 Hz, 2H), 7.48 (dd, *J* = 8.0, 2.4 Hz, 2H), 7.41 (dd, *J* = 8.0, 5.2 Hz, 1H), 7.37 (d, *J* = 8.0 Hz, 1H); $^{13}C$ NMR ($CDCl_3$, 100 MHz) δ 148.4, 148.3, 137.8, 136.6, 134.3, 129.0, 128.1, 127.1, 123.5; HRMS (ESI) *m*/*z* calcd for $C_{11}H_{10}N$ $[M+H]^+$: 156.0808, found: 156.0805.

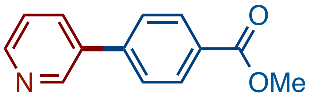


**3Ai**

**Methyl 4-(pyridin-3-yl)benzoate (3Ai)**[33]

Purification by flash column chromatography (hexane/EtOAc = 5:1) afforded **3Ai** as a white solid (61.1 mg, 72% yield). $^1H$ NMR ($CDCl_3$, 400 MHz) δ 8.89 (d, *J* = 2.4 Hz, 1H), 8.65 (dd, *J* = 4.8, 2.4 Hz, 1H), 8.15 (d, *J* = 8.8 Hz, 2H), 7.92 (dd, *J* = 8.0, 2.4 Hz, 1H), 7.66 (d, *J* = 8.8 Hz, 2H), 7.40 (dd, *J* = 8.0, 4.8 Hz, 1H), 3.96 (s, 3H); $^{13}C$ NMR ($CDCl_3$, 100 MHz) δ 166.6, 149.2, 148.3, 142.1, 135.4, 134.4, 130.3, 129.6, 127.0, 123.6, 52.2; HRMS (ESI) *m*/*z* calcd for $C_{13}H_{11}NO_2$ $[M+H]^+$: 214.0863, found: 214.0863.

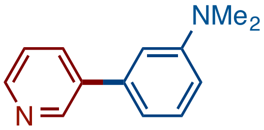


**3Aj**

***N*,*N*-Dimethyl-3-(pyridin-3-yl)aniline (3Aj)**

Purification by PTLC (hexane/EtOAc = 3:1) afforded **3Aj** as yellow oil (62.6 mg, 79% yield). $^1H$ NMR ($CDCl_3$, 400 MHz) δ 8.85 (d, *J* = 1.6 Hz, 1H), 8.57 (dd, *J* = 4.8, 1.6 Hz, 1H), 7.87 (dd, *J* = 8.0, 1.6 Hz, 1H), 7.36–7.31 (m, 2H), 6.91 (d, *J* = 7.6 Hz, 1H), 6.88 (d, *J* = 2.8 Hz, 1H), 6.77 (dd, *J* = 8.4, 2.8 Hz, 1H), 3.00 (s, 6H); $^{13}C$ NMR ($CDCl_3$, 100 MHz) δ 150.9, 148.4, 148.2, 138.7, 137.5, 134.4, 129.7, 123.3, 115.4, 112.1, 111.1, 40.5; HRMS (ESI) *m*/*z* calcd for $C_{13}H_{15}N_2$ $[M+H]^+$: 199.1230, found: 199.1227.

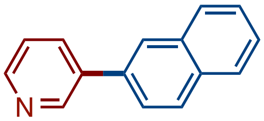


**3Ak**

**3-(Naphthalen-2-yl)pyridine (3Ak)**[34]

Purification by flash column chromatography (hexane/EtOAc = 5:1 to 4:1) afforded **3Ak** as a white solid (74.9 mg, 91% yield). $^1$H NMR (CDCl$_3$, 400 MHz) δ 8.99 (d, *J* = 2.4 Hz, 1H), 8.63 (d, *J* = 4.8 Hz, 1H), 8.05 (d, *J* = 2.0 Hz, 1H), 8.00 (dd, *J* = 8.0, 2.4 Hz, 1H), 7.96 (d, *J* = 8.8 Hz, 1H), 7.94–7.87 (m, 2H), 7.72 (dd, *J* = 8.8, 2.0 Hz, 1H), 7.58–7.49 (m, 2H), 7.40 (dd, *J* = 8.0, 4.8 Hz, 1H); $^{13}$C NMR (CDCl$_3$, 100 MHz) δ 148.6, 148.5, 136.6, 135.2, 134.6, 133.6, 132.9, 128.9, 128.2, 127.7, 126.6, 126.4, 126.2, 125.0, 123.6; HRMS (ESI) *m*/*z* calcd for $C_{15}H_{12}N$ $[M+H]^+$: 206.0964, found: 206.0964.

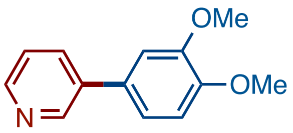


**3Al**

**3-(3,4-Dimethoxyphenyl)pyridine (3Al)**[35]

Purification by flash column chromatography (hexane/EtOAc = 1:1) afforded **3Al** as a white solid (62.6 mg, 73% yield). $^1$H NMR (CDCl$_3$, 400 MHz) δ 8.82 (d, *J* = 2.0 Hz, 1H), 8.56 (dd, *J* = 4.8, 2.0 Hz, 1H), 7.84 (dd, *J* = 8.0, 2.0 Hz, 1H), 7.34 (dd, *J* = 8.0, 4.8 Hz, 1H), 7.14 (dd, *J* = 8.4, 2.0 Hz, 1H), 7.08 (d, *J* = 2.0 Hz, 1H), 6.97 (d, *J* = 8.4 Hz, 1H), 3.97 (s, 3H), 3.93 (s, 3H); $^{13}$C NMR (CDCl$_3$, 100 MHz) δ 149.3, 149.1, 148.0, 147.9, 136.4, 133.9, 130.6, 123.4, 119.5, 111.6, 110.1, 55.9 (one peak overlap); HRMS (ESI) *m*/*z* calcd for $C_{13}H_{14}NO_2$ $[M+H]^+$: 216.1019, found: 216.1020.

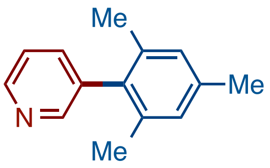


**3Am**

**3-Mesitylpyridine (3Am)**[30]

Purification by PTLC (hexane/EtOAc = 4:1) afforded **3Am** as a white solid (52.0 mg, 66% yield). $^1$H NMR (CDCl$_3$, 400 MHz) δ 8.59 (dd, *J* = 4.8, 1.6 Hz, 1H), 8.43 (d, *J* = 1.6 Hz, 1H), 7.50 (dd, *J* = 8.0, 1.6 Hz, 1H), 7.36 (dd, *J* = 8.0, 4.8 Hz, 1H), 6.97 (s, 2H), 2.34 (s, 3H), 2.00 (s, 6H); $^{13}$C NMR (CDCl$_3$, 100 MHz) δ 150.3, 148.0, 137.5, 136.9, 136.6, 136.2, 135.0, 128.3, 123.3, 21.0, 20.8; HRMS (ESI) *m*/*z* calcd for $C_{14}H_{16}N$ $[M+H]^+$: 198.1277, found: 198.1273.

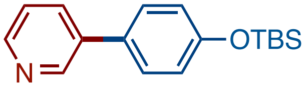


**3An**

**3-(4-((*tert*-Butyldimethylsilyl)oxy)phenyl)pyridine (3An)**

Purification by flash column chromatography (hexane/EtOAc = 5:1) afforded **3An** as colorless oil (82.4 mg, 72% yield). $^1$H NMR (CDCl$_3$, 400 MHz) δ 8.81 (d, *J* = 2.4 Hz, 1H), 8.54 (d, *J* = 4.8 Hz, 1H), 7.82 (dd, *J* = 8.0, 2.4 Hz, 1H), 7.46 (d, *J* = 6.8 Hz, 2H), 7.32 (dd, *J* = 8.0, 4.8 Hz, 1H), 6.94 (d, *J* = 6.8 Hz, 2H), 1.01 (s, 9H), 0.24 (s, 6H); $^{13}$C NMR (CDCl$_3$, 100 MHz) δ

156.0, 148.0, 147.8, 136.3, 133.8, 130.8, 128.1, 123.4, 120.7, 25.6, 18.2, –4.4; HRMS (DART) *m*/*z* calcd for $C_{17}H_{24}NOSi$ $[M+H]^+$: 286.1627, found: 286.1621.

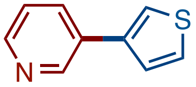


**3Ao**

**3-(Thiophen-3-yl)pyridine (3Ao)**[36]

The reaction was conducted using 1.2 equiv of $Na_2CO_3$. Purification by PTLC (hexane/$Et_2O$ = 1:1) and then GPC afforded **3Ao** as a white solid (41.3 mg, 64% yield). $^1H$ NMR ($CDCl_3$, 400 MHz) δ 8.88 (s, 1H), 8.53 (d, *J* = 4.4 Hz, 1H), 7.85 (d, *J* = 8.0 Hz, 1H), 7.52 (dd, *J* = 2.8, 1.2 Hz, 1H), 7.44 (dd, *J* = 5.2, 2.8 Hz, 1H), 7.39 (dd, *J* = 5.2, 1.2 Hz, 1H), 7.31 (dd, *J* = 8.0, 4.4 Hz, 1H); $^{13}C$ NMR ($CDCl_3$, 100 MHz) δ 148.2, 147.6, 138.7, 133.4, 131.4, 126.9, 125.8, 123.6, 121.3; HRMS (ESI) *m*/*z* calcd for $C_9H_8NS$ $[M+H]^+$: 162.0372, found: 162.0369.

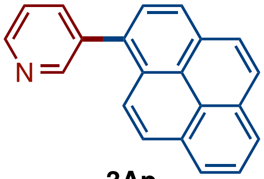


**3Ap**

**3-(Pyren-1-yl)pyridine (3Ap)**

Purification by PTLC (hexane/$Et_2O$ = 1:1) afforded **3Aq** as a yellow solid (61.7 mg, 55% yield). $^1H$ NMR ($CDCl_3$, 400 MHz) δ 8.88 (s, 1H), 8.72 (d, *J* = 4.8 Hz, 1H), 8.24–8.10 (m, 3H), 8.10–7.95 (m, 5H), 7.94–7.79 (m, 2H), 7.46 (dd, *J* = 8.8, 4.8 Hz, 1H); $^{13}C$ NMR ($CDCl_3$, 100 MHz) δ 151.0, 148.4, 137.6, 136.7, 133.4, 131.3, 131.0, 130.7, 128.5, 128.0, 127.8, 127.4, 127.2, 126.1, 125.4, 125.0, 124.8, 124.64, 124.61, 124.2, 123.2; HRMS (DART) *m*/*z* calcd for $C_{21}H_{14}N$ $[M+H]^+$: 280.1126, found: 280.1122.

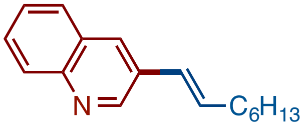


**3Wq**

**(*E*)-3-(Oct-1-en-1-yl)quinoline (3Wq)**

The reaction was conducted using NaCl (1.0 equiv) as additive. Purification by PTLC (hexane/EtOAc = 8:1) and then GPC afforded **3Wq** as colorless oil (57.8 mg, 57% yield). $^1H$ NMR ($CDCl_3$, 600 MHz) δ 8.97 (s, 1H), 8.06 (d, *J* = 8.8 Hz, 1H), 7.98 (s, 1H), 7.76 (d, *J* = 8.8 Hz, 1H), 7.64 (t, *J* = 8.8 Hz, 1H), 7.51 (t, *J* = 8.8 Hz, 1H), 6.54–6.43 (m, 2H), 2.28 (q, *J* = 8.0 Hz, 2H), 1.56–1.46 (m, 2H), 1.42–1.27 (m, 6H), 0.91 (q, *J* = 6.8 Hz, 3H); $^{13}C$ NMR ($CDCl_3$, 150 MHz) δ 149.4, 147.1, 134.1, 131.5, 130.8, 129.2, 128.7, 128.2, 127.6, 126.8, 126.5, 33.3, 31.7, 29.2, 28.9, 22.6, 14.1; HRMS (ESI) *m*/*z* calcd for $C_{17}H_{22}N$ $[M+H]^+$: 240.1747, found: 240.1752.

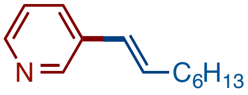


**3Aq**

**(*E*)-3-(Oct-1-en-1-yl)pyridine (3Aq)**[37]

The reaction was conducted by using 1.0 equiv of NaCl as additive. Purification by PTLC (hexane/EtOAc = 5:1) afforded **3Aq** as colorless oil (35.0 mg, 46% yield). $^1$H NMR ($CDCl_3$, 400 MHz) δ 8.56 (s, 1H), 8.42 (d, *J* = 4.4 Hz, 1H), 7.65 (d, *J* = 8.0 Hz, 1H), 7.21 (dd, *J* = 8.0, 4.4 Hz, 1H), 6.40–6.25 (m, 2H), 2.23 (q, *J* = 8.0 Hz, 2H), 1.52–1.43 (m, 2H), 1.40–1.32 (m, 6H), 0.88 (t, *J* = 6.8 Hz, 3H); $^{13}$C NMR ($CDCl_3$, 100 MHz) δ 147.94, 147.86, 133.7, 133.5, 132.3, 126.2, 123.3, 33.1, 31.7, 29.1, 28.9, 22.6, 14.1; HRMS (ESI) *m*/*z* calcd for $C_{13}H_{20}N$ $[M+H]^+$: 190.1590, found: 190.1586.

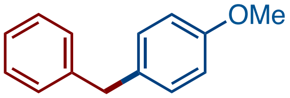


**3AFa**

**1-Benzyl-4-methoxybenzene (3AFa)**[38]

The reaction was conducted using *N*,*N*-dimethyl-4-aminopyridine (DMAP: 14.7 mg, 0.12 mmol, 30 mol%) as additive. Purification by PTLC (hexane/EtOAc = 20:1) afforded **3AFa** as colorless oil (36.5 mg, 46% yield). $^1$H NMR ($CDCl_3$, 400 MHz) δ 7.27 (dd, *J* = 7.6, 7.2 Hz, 2H), 7.21–7.15 (m, 3H), 7.10 (d, *J* = 8.4 Hz, 2H), 6.82 (d, *J* = 8.4 Hz, 2H), 3.92 (s, 2H), 3.78 (s, 3H); $^{13}$C NMR ($CDCl_3$, 100 MHz) δ 157.9, 141.6, 133.2, 129.8, 128.8, 128.4, 126.0, 113.9, 55.2, 41.0; HRMS (DART) *m*/*z* calcd for $C_{14}H_{15}O$ $[M+H]^+$: 199.1123, found: 199.1122.

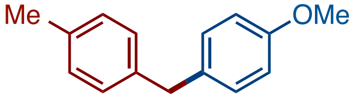


**3AGa**

**1-Methoxy-4-(4-methylbenzyl)benzene (3AGa)**[38]

The reaction was conducted using *N*,*N*-dimethyl-4-aminopyridine (DMAP: 14.7 mg, 0.12 mmol, 30 mol%) as additive. Purification by PTLC (hexane/EtOAc = 20:1) afforded **3AGa** as colorless oil (30.0 mg, 35% yield). $^1$H NMR ($CDCl_3$, 400 MHz) δ 7.12–7.03 (m, 6H), 6.81 (d, *J* = 8.4 Hz, 2H), 3.88 (s, 2H), 3.77 (s, 3H), 2.31 (s, 3H); $^{13}$C NMR ($CDCl_3$, 100 MHz) δ 157.9, 138.5, 135.4, 133.5, 129.8, 129.1, 128.7, 113.8, 55.2, 40.6, 21.0; HRMS (FAB) *m*/*z* calcd for $C_{15}H_{16}O$ [M]: 212.1201, found: 212.1203

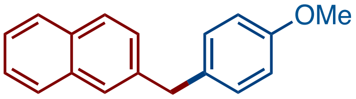


**3AHa**

**2-(4-Methoxybenzyl)naphthalene (3AHa)**[38]

The reaction was conducted using *N*,*N*-dimethyl-4-aminopyridine (DMAP: 14.7 mg, 0.12 mmol, 30 mol%) as additive. Purification by PTLC (hexane/EtOAc = 20:1) afforded **3AHa** as a

white solid (45.8 mg, 46% yield). $^1H$ NMR ($CDCl_3$, 400 MHz) δ 7.81–7.77 (m, 3H), 7.60 (s, 1H), 7.47–7.35 (m, 2H), 7.29 (dd, $J$ = 8.4, 1.6 Hz, 1H), 7.12 (dd, $J$ = 8.8, 1.6 Hz, 2H), 6.82 (dd, $J$ = 8.8, 1.6 Hz, 2H), 4.07 (s, 2H), 3.77 (s, 3H); $^{13}C$ NMR ($CDCl_3$, 100 MHz) δ 158.0, 139.0, 133.6, 133.0, 132.0, 129.9, 128.0, 127.6, 127.54, 127.50, 126.9, 125.9, 125.3, 113.9, 55.2, 41.2; HRMS (FAB) $m/z$ calcd for $C_{18}H_{16}O$ $[M]^+$: 248.1201, found: 248.1207.

## 3. Gram-scale Reaction

**[Gram-Scale]**

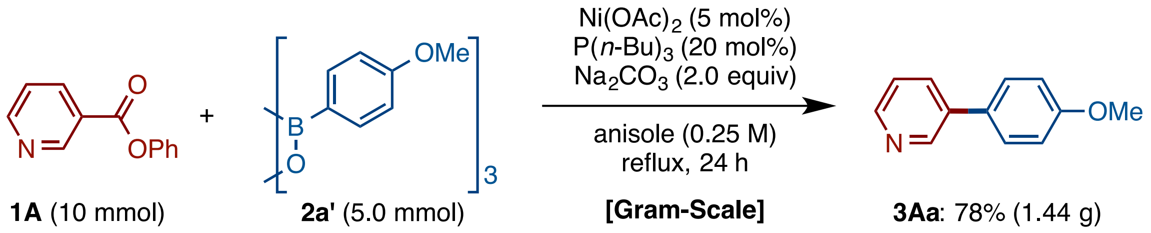


A 100-mL Schlenk tube equipped with a condenser and balloon containing a magnetic stirring bar and $Ni(OAc)_2·4H_2O$ (124 mg, 0.5 mmol, 5 mol%) was dried with a heatgun *in vacuo* and filled with $N_2$ gas after cooling to room temperature. To this was added phenyl nicotinate (**1A**: 1.99 g, 10 mmol, 1.0 equiv), tri(*p*-methoxyphenyl)boroxine (**2a'**: 2.00 g, 5.0 mmol, 0.5 equiv), and $Na_2CO_3$ (2.12 g, 20 mmol, 2.0 equiv). The tube was vacuumed and refilled $N_2$ gas three times. To this was added P(*n*-Bu)$_3$ (0.49 mL, 2.0 mmol, 20 mol%) and anisole (40 mL). The Schlenk cock was closed and then this mixture was refluxed for 24 h in an oil bath (oil bath temperature was 160 °C) with stirring. After cooling the reaction mixture to room temperature, the mixture was passed through a short silica gel pad with EtOAc. The filtrate was concentrated *in vacuo* and the residue was purified by flash column chromatography (hexane/$Et_2O$ = 3:2 to 2:3) to afford 3-(4-methoxyphenyl)pyridine (**3Aa**: 1.44 g, 78% yield) as a white solid.

## 4. One-pot Transformation of Thiophene-2-carboxylic Acid to Biaryl 3Ka

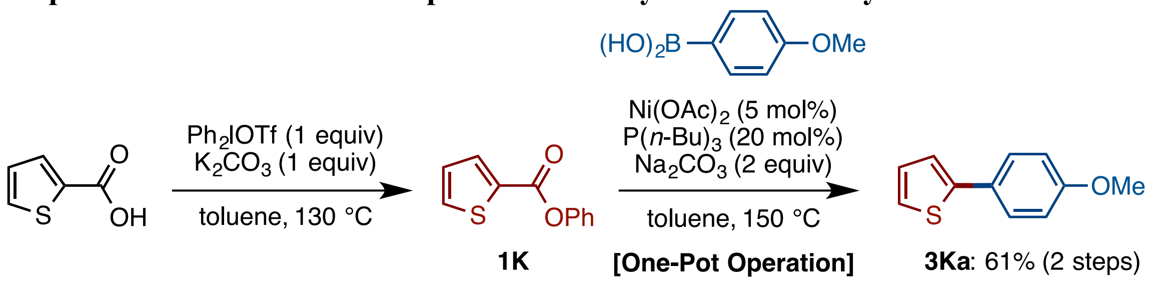


A 20-mL glass vessel equipped with J. Young® O-ring tap containing a magnetic stirring bar was dried with heatgun under reduced pressure and filled with $N_2$ gas after cooling to room temperature. To this was added thiophene-2-carboxylic acid (51.3 mg, 0.40 mmol), diphenyliodonium triflate (172.0 mg, 0.40 mmol, 1.0 equiv), $K_2CO_3$ (55.2 mg, 0.40 mmol, 1.0 equiv), and then toluene (2.0 mL). The vessel was sealed with O-ring tap and then heated at 130 °C for 2 h in an 8-well reaction block with stirring. After cooling the reaction mixture to room temperature, this mixture was concentrated *in vacuo* to remove toluene and iodobenzene.

To the same tube containing obtained crude product **1K** were added $Ni(OAc)_2$ (3.5 mg, 0.02 mmol, 5 mol%), *p*-methoxyphenylboronic acid **3** (0.60 mmol, 91.9 mg, 1.5 equiv), and $Na_2CO_3$

(84.8 mg, 0.8 mmol, 2.0 equiv). The vessel was vacuumed and refilled $N_2$ gas three times. To this was added P(*n*-Bu)$_3$ (19.0 mL, 0.08 mmol, 20 mol%) and toluene (1.6 mL). The vessel was sealed with O-ring tap and then heated at 150 °C for 24 h in an 8-well reaction block with stirring. After cooling the reaction mixture to room temperature, the mixture was passed through a short silica gel pad with EtOAc. The filtrate was concentrated and the residue was purified by flash column chromatography (hexane/EtOAc = 100:1) to afford 2-(4-methoxyphenyl)thiophene (**3Ka**: 46.2 mg, 61% yield over 2 steps) as a white solid.

**5. Application to the Synthesis of Telmisartan Derivtives**

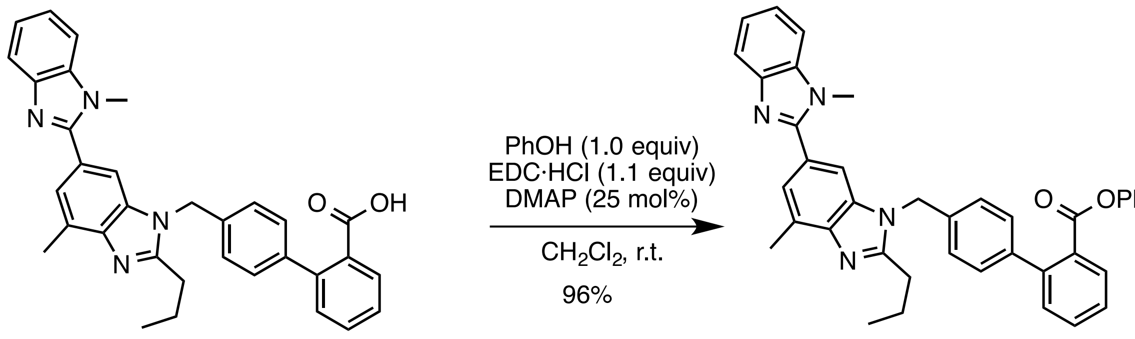


telmisartan　　　　**4** (telmisartan Ph-ester)

To a test tube equipped with screw cap containing a magnetic stirring bar were added telmisartan (772 mg, 1.5 mmol, 1.0 equiv), phenol (155 mg, 1.65 mmol, 1.1 equiv), 1-(3-dimethylaminopropyl)-3- ethylcarbodiimide hydrochloride (EDC·HCl: 316 mg, 1.65 mmol, 1.1 equiv), *N,N*-dimethyl-4-aminopyridine (DMAP: 18.3 mg, 0.15 mmol, 0.1 equiv) and $CH_2Cl_2$ (3.0 mL). After stirring for 6 h, the reaction mixture was quenched with saturated $NaHCO_3$aq and extracted three times with $CH_2Cl_2$. The combined organic layer was dried over $Na_2SO_4$, filtrated, and concentrated *in vacuo*. The residue was purified by flash column chromatography (hexane/EtOAc = 2:1 to EtOAc) to afford telmisartan Ph-ester (**4**: 850 mg, 96% yield) as a white solid. $^1$H NMR ($CDCl_3$, 400 MHz) δ 8.03 (dd, *J* = 8.0, 1.6 Hz, 1H), 7.83–7.80 (m, 1H), 7.60 (td, *J* = 8.0, 1.6 Hz, 1H), 7.52–7.44 (m, 3H), 7.40–7.35 (m, 3H), 7.32–7.28 (m, 3H), 7.21 (t, *J* = 8.0 Hz, 2H), 7.12 (d, *J* = 8.0 Hz, 2H), 7.04 (t, *J* = 8.0 Hz, 1H), 6.85 (d, *J* = 8.0 Hz, 2H), 5.45 (s, 2H), 3.71 (s, 3H), 2.91 (t, *J* = 8.0 Hz, 2H), 2.79 (s, 3H), 1.91–1.81 (m, 2H), 1.03 (t, *J* = 8.0 Hz, 3H); $^{13}$C NMR ($CDCl_3$, 100 MHz) δ 166.6, 156.4, 154.7, 150.6, 143.2, 142.9, 142.3, 141.1, 136.7, 135.0, 131.9, 130.9, 130.4, 129.9, 129.5, 129.25, 129.20, 127.6, 125.9, 125.7, 124.0, 123.9, 122.4, 122.3, 121.1, 119.6, 109.5, 108.8, 46.9, 31.7, 29.8, 21.8, 16.9, 14.1; HRMS (DART) *m*/*z* calcd for $C_{39}H_{35}N_4O_2$ [M+H]$^+$: 591.2760, found: 591.2770.

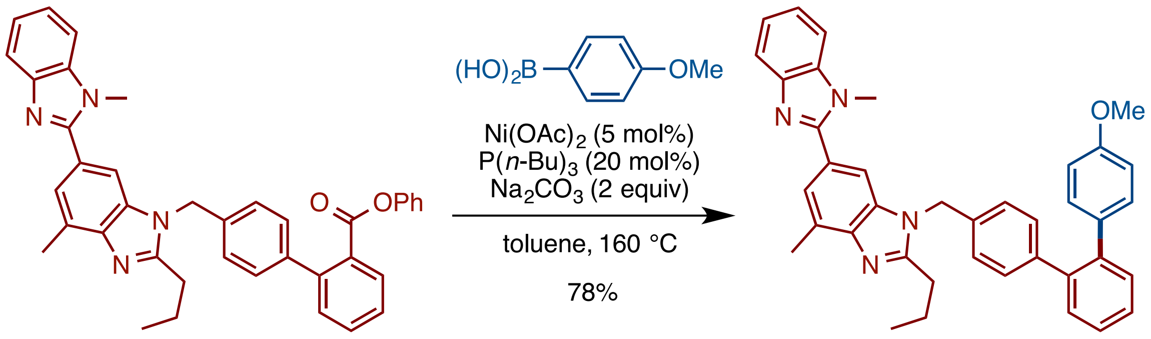


**4** (telmisartan Ph-ester) **5**

A 20-mL glass vessel equipped with J. Young® O-ring tap containing a magnetic stirring bar and $Ni(OAc)_2 \cdot 4H_2O$ (3.5 mg, 0.0125 mmol, 5 mol%) was dried with a heatgun under reduced pressure and filled with $N_2$ gas after cooling to room temperature. To this vessel was added telmisartan Ph-ester **4** (145 mg, 0.25 mmol, 1.0 equiv), *p*-methoxyphenylboronic acid (**2a**: 58.0 mg, 0.375 mmol, 1.5 equiv), and $Na_2CO_3$ (53 mg, 0.50 mmol, 2.0 equiv). The vessel was vacuumed and refilled $N_2$ gas three times. To this were added P(*n*-Bu)$_3$ (12.5 mL, 0.05 mmol, 20 mol%) and toluene (1.0 mL). The vessel was sealed with O-ring tap and then heated at 160 °C for 24 h in an 8-well reaction block with stirring. After cooling the reaction mixture to room temperature, the mixture was passed through Celite® with EtOAc. The filtrate was concentrated and the residue was purified by flash column chromatography (hexane/EtOAc = 1:2 to EtOAc), followed by reverse-phase HPLC (Isolera®; $H_2O$/MeCN) to afford a coupling product **5** as a white solid (111 mg, 78% yield). $^1$H NMR ($CDCl_3$, 400 MHz) δ 7.84–7.81 (m, 1H), 7.51 (s, 1H), 7.44 (s, 1H), 7.40–7.28 (m, 7H), 7.10 (d, *J* = 8.0 Hz, 2H), 7.01 (d, *J* = 8.8 Hz, 2H), 6.94 (d, *J* = 8.0 Hz, 2H), 6.71 (d, *J* = 8.8 Hz, 2H), 5.36 (s, 2H), 3.80 (s, 3H), 3.72 (s, 3H), 2.87 (t, *J* = 7.6 Hz, 2H), 2.78 (s, 3H), 1.85–1.76 (m, 2H), 1.03 (t, *J* = 7.6 Hz, 3H); $^{13}$C NMR ($CDCl_3$, 100 MHz) δ 158.3, 156.4, 154.7, 143.1, 142.9, 141.5, 140.1, 139.5, 136.7, 135.2, 133.9, 133.6, 130.8, 130.6, 130.4, 127.6, 125.8, 123.9, 123.7, 122.4, 122.3, 119.5, 113.3, 109.5, 108.7, 55.1, 47.0, 31.8, 29.8, 21.7, 16.8, 14.0; HRMS (DART) *m*/*z* calcd for $C_{39}H_{37}N_4O$ $[M+H]^+$: 577.2967, found: 577.2969.

## 6. Orthogonal Coupling of 6, 1Q, and 10

### 6-1. Orthogonal Coupling of 6

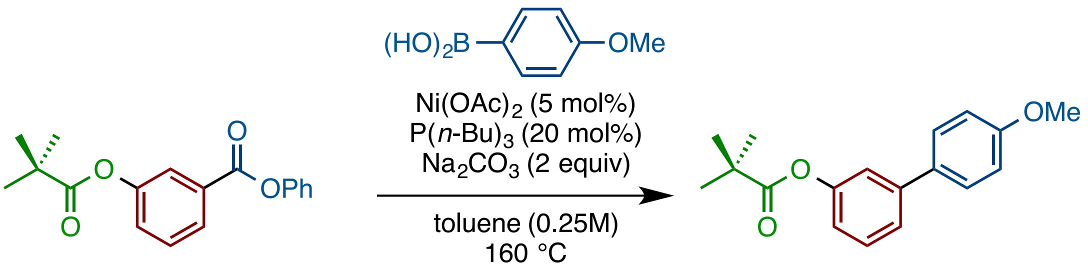


**6** (0.40 mmol) **7** (74%)

A 20-mL glass vessel equipped with J. Young® O-ring tap containing a magnetic stirring bar and $Ni(OAc)_2 \cdot 4H_2O$ (5.0 mg, 0.020 mmol, 5 mol%) was dried with a heatgun under reduced pressure and filled with $N_2$ gas after cooling to room temperature. To this vessel was added phenyl 3-(pivaloyloxy)benzoate (**6**: 119.2 mg, 0.40 mmol, 1.0 equiv), *p*-methoxyphenylboronic

acid (**2a**: 91.8 mg, 0.60 mmol, 1.5 equiv), and $Na_2CO_3$ (85.0 mg, 0.8 mmol, 2.0 equiv). The vessel was vacuumed and refilled $N_2$ gas three times. To this were added P(*n*-Bu)$_3$ (20 mL, 0.08 mmol, 20 mol%) and toluene (1.6 mL). The vessel was sealed with O-ring tap and then heated at 150 °C for 24 h in an 8-well reaction block with stirring. After cooling the reaction mixture to room temperature, the mixture was passed through a short silica gel pad with EtOAc. The filtrate was concentrated *in vacuo* and the residue was purified by flash column chromatography (hexane/EtOAc = 100:1), and then GPC to afford 4'-methoxy-[1,1'-biphenyl]-3-yl pivalate **7** (84.2 mg, 74% yield) as colorless oil. $^1$H NMR ($CDCl_3$, 400 MHz) δ 7.51 (d, *J* = 8.8 Hz, 2H), 7.41–7.37 (m, 2H), 7.22 (s, 1H), 7.01–6.98 (m, 1H), 6.96 (d, *J* = 8.8 Hz, 2H), 3.83 (s, 3H), 1.39 (s, 9H); $^{13}$C NMR ($CDCl_3$, 100 MHz) δ 177.1, 159.3, 151.4, 142.4, 132.7, 129.5, 128.2, 123.9, 119.7, 119.6, 114.1, 55.3, 39.1, 27.1; HRMS (DART) *m*/*z* calcd for $C_{18}H_{21}O_3$ [M+H]$^+$: 285.1491, found: 285.1494.

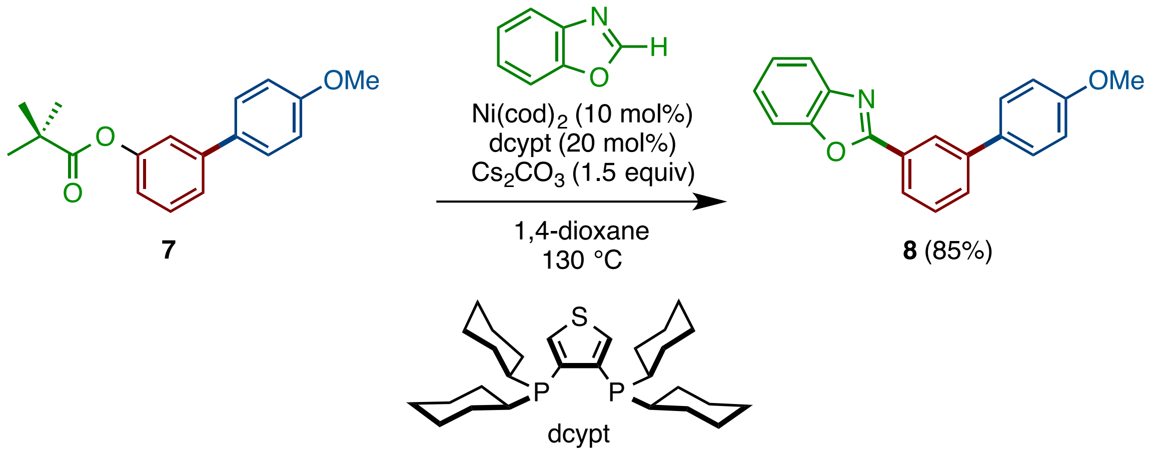


**7**

**8** (85%)

A 20-mL glass vessel equipped with J. Young® O-ring tap containing a magnetic stirring bar and $Cs_2CO_3$ (146.6 mg, 0.45 mmol, 1.5 equiv) was dried with a heatgun under reduced pressure and filled with $N_2$ gas after cooling to room temperature. To this vessel was added 4'-methoxy-[1,1'-biphenyl]-3-yl pivalate (**7**: 85.3 mg, 0.30 mmol, 1.0 equiv) and 2,3-bis(dicyclohexylphosphine)thiophene (dcypt: 28.3 mg, 0.06 mmol, 20 mol%), and then introduced into an argon-atmosphere glovebox. To the reaction vessel was added Ni(cod)$_2$ (8.3 mg, 0.03 mmol, 10 mol%), and then taken out of the glovebox. To this tube were added benzoxazole (47.6 mg, 0.40 mmol, 1.3 equiv) and 1,4-dioxane (1.5 mL) under a stream of $N_2$. The vessel was sealed with O-ring tap and then heated at 130 °C for 12 h in an 8-well reaction block with stirring. After cooling the reaction mixture to room temperature, the mixture was passed through a short silica gel pad with EtOAc. The filtrate was concentrated *in vacuo* and the residue was purified by flash column chromatography (hexane/EtOAc = 100:1) to afford 2-(4'-methoxy-[1,1'-biphenyl]-3-yl)benzo[*d*]oxazole (**8**: 76.8 mg, 85% yield) as a white solid. $^1$H NMR ($CDCl_3$, 400 MHz) δ 8.46 (s, 1H), 8.18 (d, *J* = 8.0 Hz, 1H), 7.83–7.77 (m, 1H), 7.72 (d, *J* = 8.0 Hz, 1H), 7.65–7.53 (m, 4H), 7.40–7.33 (m, 2H), 7.01 (d, *J* = 8.8 Hz, 2H), 3.87 (s, 3H); $^{13}$C NMR ($CDCl_3$, 100 MHz) δ 163.0, 159.5, 150.7, 142.1, 141.5, 132.5, 129.7, 129.3, 128.2, 127.5, 125.7, 125.1, 124.6, 120.0, 114.3, 110.6, 55.3 (one peak overlap); HRMS (DART) *m*/*z* calcd for $C_{20}H_{16}NO_2$ [M+H]$^+$: 302.1181, found: 302.1178.

## 6-2. Orthogonal Coupling of 1Q

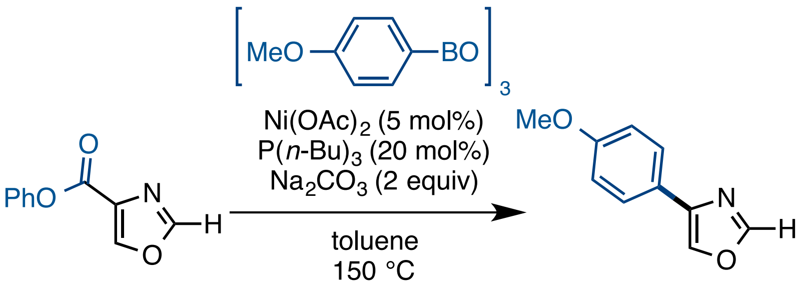


**1Q** **3Qa** (57%)

A 20-mL glass vessel equipped with J. Young® O-ring tap containing a magnetic stirring bar and $Ni(OAc)_2 \cdot 4H_2O$ (5.0 mg, 0.020 mmol, 5 mol%) was dried with a heatgun for 1 min under reduced pressure and filled with $N_2$ gas after cooling to room temperature. To this vessel was added phenyl oxazole-4-carboxylate (**1Q**: 75.7 mg, 0.40 mmol, 1.0 equiv), *p*-methoxyphenylboroxine (**2a'**: 80.3 mg, 0.20 mmol, 0.50 equiv), and $Na_2CO_3$ (85.0 mg, 0.8 mmol, 2.0 equiv). The vessel was vacuumed and refilled with $N_2$ gas three times. To this were added P(*n*-Bu)$_3$ (20 mL, 0.08 mmol, 20 mol%) and toluene (1.6 mL). The vessel was sealed with O-ring tap and then heated at 150 °C for 24 h in an 8-well reaction block with stirring. After cooling the reaction mixture to room temperature, the mixture was passed through a short silica gel pad with EtOAc. The filtrate was concentrated *in vacuo* and the residue was purified by flash column chromatography by using Isolera® (hexane/EtOAc = 95:5 to 4:1), and then GPC to afford 4-(4-methoxyphenyl)oxazole (**3Qa**: 40.7 mg, 57% yield) as a white solid.

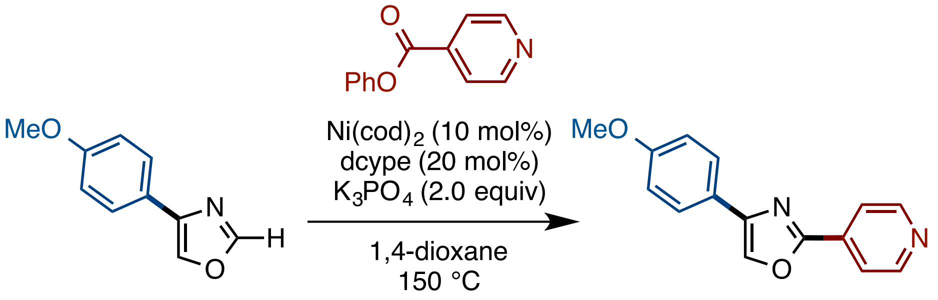


**3Qa** **9** (99%)

A 20-mL glass vessel equipped with J. Young® O-ring tap containing a magnetic stirring bar and $K_3PO_4$ (169.8 mg, 0.80 mmol, 2.0 equiv) was dried with a heatgun for 3 minutes *in vacuo* and filled with $N_2$ gas after cooling to room temperature. To this vessel was added 4-(4-methoxyphenyl)oxazole (**3Qa**: 70.7 mg, 0.40 mmol, 1.0 equiv) and phenyl 4-nicotinate (119.5 mg, 0.60 mmol, 1.5 equiv), and then introduced into an argon-atmosphere glovebox. To the reaction vessel was added Ni(cod)$_2$ (11.0 mg, 0.04 mmol, 10 mol%) and 1,2-bis(dicyclohexylphosphino)ethane (dcype: 33.8 mg, 0.08 mmol, 20 mol%), and then taken out of the glovebox. To this tube was added 1,4-dioxane (1.6 mL) under a stream of $N_2$. The vessel was sealed with O-ring tap and then heated at 150 °C for 24 h in an 8-well reaction block with stirring. After cooling the reaction mixture to room temperature, the mixture was passed through a Celite® pad with EtOAc as an eluent. The filtrate was concentrated *in vacuo* and the residue was purified by PTLC (hexane/EtOAc = 1:1) to afford 4-(4-methoxyphenyl)-2-(pyridin-4-yl)oxazole (**9**: 100.5 mg, 99% yield) as a white solid. $^1H$ NMR ($CDCl_3$, 400 MHz) δ 8.76 (dd, *J* = 4.4, 1.2 Hz, 2H), 7.96–7.92 (m, 3H), 7.75 (dd, *J* = 7.2, 2.4 Hz, 2H), 6.98 (dd, *J* = 7.2, 2.4 Hz, 2H), 3.86 (s, 3H) ;

$^{13}C$ NMR ($CDCl_3$, 100 MHz) δ 159.8, 159.4, 150.5, 142.5, 134.3, 133.5, 127.0, 123.1, 120.0, 114.2, 55.3; HRMS (DART) *m/z* calcd for $C_{15}H_{13}N_2O_2$ $[M+H]^+$: 253.0977, found: 253.0972.

## 6-3. Orthogonal Coupling of 10

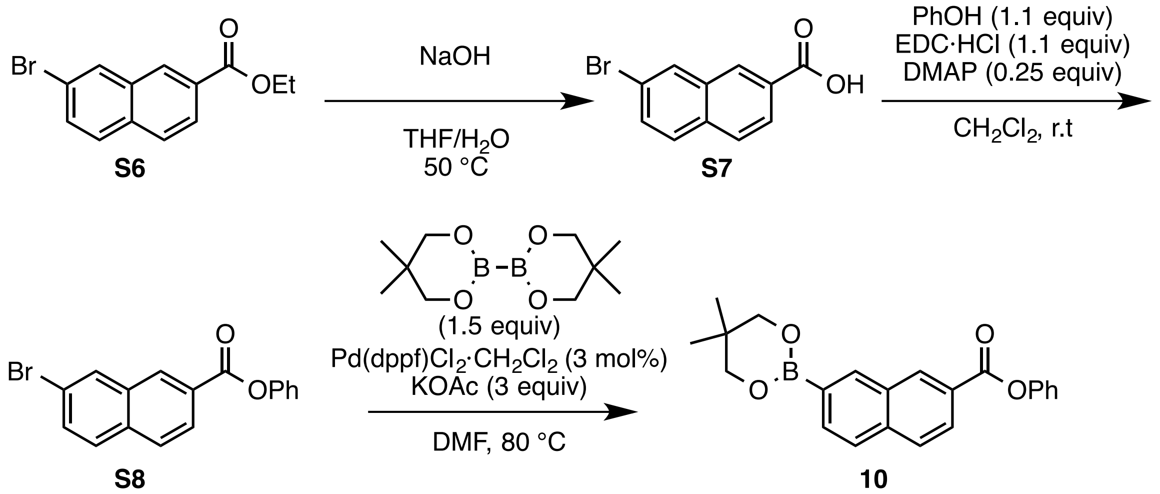


The solution of **S6** (1.95 g, 7.0 mmol, 1.0 equiv) in THF (28 mL) was treated with 1M NaOHaq (14 mL, 2.0 equiv) at 50 °C. After stirring overnight, the mixture was diluted with $Et_2O$ and washed two times with $Et_2O$. 1 M HClaq was added to the combined water phase to adjust the pH to 2, and then resulted solution was extracted three times with $Et_2O$. The combined organic phase was concentrated *in vacuo* to afford 7-bromo-2-naphthoic acid (**S7:** 1.76 g) as a white solid. This was used without further purification.

To a round-bottomed flask with the carboxylic acid **S7** (1.76 g, 7.0 mmol, 1.0 equiv) were added phenol (725 mg, 7.7 mmol, 1.1 equiv), 1-(3-dimethylaminopropyl)-3-ethylcarbodiimide hydrochloride (EDC·HCl: 1.48 g, 7.7 mmol, 1.1 equiv), *N,N*-dimethyl-4-aminopyridine (DMAP: 213.8 mg, 1.75 mmol, 0.25 equiv) and $CH_2Cl_2$ (28 mL). After stirring the mixture for several hours with monitoring reaction progress on TLC, the reaction was quenched with saturated $NaHCO_3$aq and extracted three times with $CH_2Cl_2$. The combined organic layer was dried over $Na_2SO_4$, passed a short silica gel pad, and concentrated *in vacuo*. The residue was purified by recrystallization (hexane/$CH_2Cl_2$) to afford phenyl 7-bromo-2-naphthoate (**S8**: 2.15 g, 94% yield) as a white solid.

To a dried 50-mL Schlenk tube were added dehydrated KOAc (883 mg, 9.0 mmol, 3.0 equiv), phenyl 7-bromo-2-naphthoate (**S8**: 982 mg, 3.0 mmol, 1.0 equiv), Pd(dppf)$Cl_2$·$CH_2Cl_2$ (73.5 mg, 0.090 mmol, 3 mol%), and bis(neopentyl glycolato)diboron (1.01 g, 4.5 mmol, 1.5 equiv). The tube was vacuumed and filled with $N_2$ gas three times. DMF (12 mL) was added under a stream of $N_2$ gas and the mixture was heated at 80 °C for 12 h in an oil bath. After cooling the mixture to room temperature, the mixture was diluted with EtOAc and passed a silica-gel pad with EtOAc as an eluent. The mixture was washed with brine, dried over $Na_2SO_4$, filtered, and then concentrated *in vacuo*. The resulted crude product was purified by silica-gel column chromatography by using Isolera® (hexane/EtOAc = 10:1 to 1:1) to afford phenyl 7-(5,5-dimethyl-1,3,2-dioxaborinan-2-yl)-2-naphthoate (**10**: 940 mg, 87% yield) as a white solid. $^1H$ NMR ($CDCl_3$, 400 MHz) δ 8.77 (s, 1H), 8.41 (s, 1H), 8.19 (d, *J* = 8.4 Hz, 1H), 8.01–7.91 (m, 3H), 7.45 (d, *J* = 8.4 Hz, 2H), 7.32–7.22 (m, 3H), 3.84 (s, 4H), 1.07 (s, 6H); $^{13}C$ NMR ($CDCl_3$,

100 MHz) δ 165.4, 151.1, 135.2, 134.7, 133.8, 131.7, 130.9, 129.5, 129.1, 128.3, 127.3, 125.9, 125.2, 121.8, 72.5, 32.0, 21.9; HRMS (DART) *m/z* calcd for $C_{22}H_{22}BO_4$ $[M+H]^+$: 361.1611, found: 361.1611.

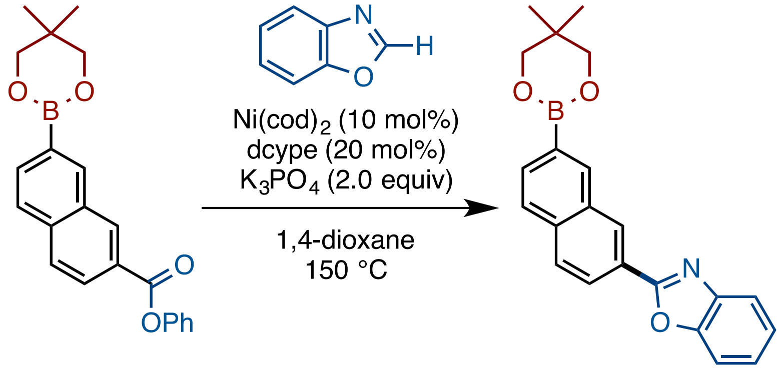


**10** **11** (50%)

A 20-mL glass vessel equipped with J. Young® O-ring tap containing a magnetic stirring bar and $K_3PO_4$ (169.8 mg, 0.80 mmol, 2.0 equiv) was dried with a heatgun for 3 min *in vacuo* and filled with $N_2$ gas after cooling to room temperature. To this vessel was added phenyl 7-(5,5-dimethyl-1,3,2-dioxaborinan-2-yl)-2-naphthoate (**10**: 144.1 mg, 0.40 mmol, 1.0 equiv), and then introduced into an argon-atmosphere glovebox. To the reaction vessel was added $Ni(cod)_2$ (11.0 mg, 0.04 mmol, 10 mol%) and 1,2-bis(dicyclohexylphosphino)ethane (dcype: 33.8 mg, 0.08 mmol, 20 mol%), and then taken out of the glovebox. To this tube were added benzoxazole (60 μL, 71.4 mg, 0.60 mmol, 1.5 equiv) and 1,4-dioxane (1.6 mL) under a stream of $N_2$. The vessel was sealed with O-ring tap and then heated at 150 °C for 12 h in an 8-well reaction block with stirring. After cooling the reaction mixture to room temperature, the mixture was passed through a silica-gel pad with EtOAc as an eluent. The filtrate was concentrated *in vacuo* and the residue was purified by silica-gel column chromatography by using Isolera® (hexane/EtOAc = 100:1 to EtOAc) to afford a pale yellow solid (82 mg). This solid was further purified by silica-gel column chromatography by using Isolera® (hexane/$CHCl_3$ = 4:1 to $CHCl_3$) to afford 2-(7-(5,5-dimethyl-1,3,2-dioxaborinan-2-yl)naphthalen-2-yl)benzo[*d*]oxazole (**11**: 71.3 mg, 50% yield) as a white solid. $^1H$ NMR ($CDCl_3$, 400 MHz) δ 8.77 (s, 1H), 8.40 (s, 1H), 8.31 (dd, *J* = 9.0, 1.2 Hz, 1H), 8.01 (d, *J* = 9.0 Hz, 1H), 7.98–7.92 (m, 2H), 7.83–7.80 (m, 1H), 7.65–7.62 (m, 1H), 7.40–7.35 (m, 2H), 3.85 (s, 4H), 1.08 (s, 6H); $^{13}C$ NMR ($CDCl_3$, 100 MHz) δ 163.2, 150.8, 142.2, 134.8, 134.3, 134.1, 131.0, 129.5, 127.9, 127.7, 125.1, 124.9, 124.6, 123.7, 120.0, 110.6, 72.4, 31.9, 21.9; HRMS (DART) *m/z* calcd for $C_{22}H_{21}BNO_3$ $[M+H]^+$: 358.1615, found: 358.1618.

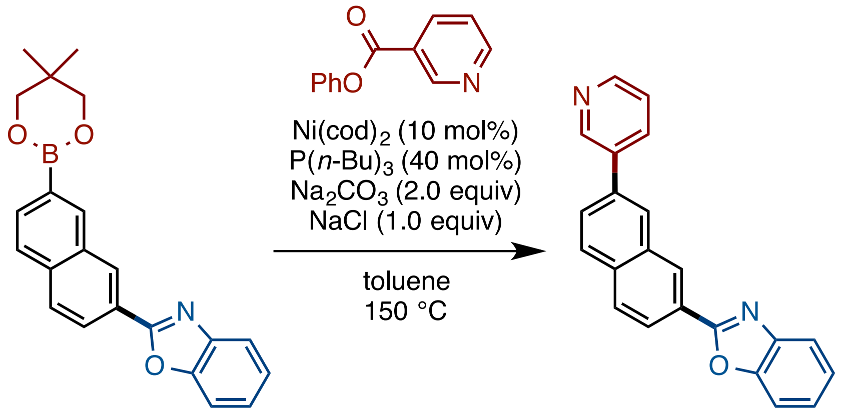


A 20-mL glass vessel equipped with J. Young® O-ring tap containing a magnetic stirring bar was dried with a heatgun *in vacuo* and filled with $N_2$ gas after cooling to room temperature. To this vessel was added a phenyl 3-nicotinate (**1A**: 59.8 mg, 0.30 mmol, 1.5 equiv), 2-(7-(5,5-dimethyl-1,3,2-dioxaborinan-2-yl)naphthalen-2-yl)benzo[*d*]oxazole (**11**: 71.4 mg, 0.20 mmol, 1.0 equiv), $Na_2CO_3$ (42.4 mg, 0.4 mmol, 2.0 equiv), and NaCl (11.9 mg, 0.20 mmol, 1.0 equiv). The vessel was introduced into an argon-atmosphere glovebox. To the reaction vessel was added Ni(cod)$_2$ (5.5 mg, 0.020 mmol, 10 mol%), and then taken out of the glovebox. To this were added P(*n*-Bu)$_3$ (10 mL, 0.08 mmol, 40 mol%) and toluene (0.8 mL) under a stream of $N_2$. The vessel was sealed with O-ring tap and then heated at 150 °C for 48 h in an oil bath with stirring. After cooling the reaction mixture to room temperature, the mixture was passed through a short silica gel pad with EtOAc. The filtrate was concentrated *in vacuo* and the residue was purified by silica-gel column chromatography by using Isolera® (hexane/EtOAc = 4:1 to EtOAc) to afford 2-(7-(pyridin-3-yl)naphthalen-2-yl)benzo[*d*]oxazole (**12**: 33.8 mg, 52% yield) as a white solid. $^{1}$H NMR ($CDCl_3$, 600 MHz) δ 9.00 (s, 1H), 8.79 (s, 1H), 8.66 (d, $J$ = 4.2 Hz, 1H), 8.36 (d, $J$ = 9.0 Hz, 1H), 8.10–8.07 (m, 2H), 8.05–7.98 (m, 2H), 7.84–7.75 (m, 2H), 7.64–7.60 (m, 1H), 7.45–7.41 (m, 1H), 7.40–7.34 (m, 2H); $^{13}$C NMR ($CDCl_3$, 150 MHz) δ 162.9, 150.8, 148.9, 148.5, 142.2, 137.0, 136.1, 134.9, 134.6, 132.4, 129.9, 129.1, 127.8, 126.1, 125.3, 124.9, 124.7, 123.7, 120.1, 110.6; HRMS (DART) *m*/*z* calcd for $C_{22}H_{15}N_2O$ $[M+H]^+$: 323.1184, found: 323.1181.

## 7. Computational Details

Calculations were performed by Gaussian 09 quantum chemical package.[39] The geometries of all reported structures were optimized without symmetry constraints in toluene at the M06L level of density functional theory[40] in conjunction with the Lanl2dz basis set and corresponding Hay–Wadt ECP for Ni[41,42]. Standard 6-31G(d) basis sets were used for all other atoms. Below, this approach will be called as M06L/{Lanl2dz + [6-31G(d)]} or M06L/BS1. Solvent effects were estimated by using the PCM solvation method[43–45]. Previously, we have shown that this approach reasonable describes geometries and energetics of the Ni-complexes and Ni-catalyzed coupling reactions[46]. In order to incorporate disperse interactions into calculations we also performed geometry optimization and energy calculations of selected important intermediates and transition states at the M06/BS1 level of theory[47].

The nature of each stationary point was characterized by performing normal mode analysis at the appropriate (i.e. same as a geometry optimization) levels of theory. Relative free

energies and enthalpies of all reported structures were calculated under standard conditions (1 atm and 298.15 K). In the presented figures and tables, we give both relative Gibbs free energies and enthalpies (in kcal/mol) as ΔG/ΔH. Cartesian coordinates and total energies of all reported structures are also presented below. In this paper, we mainly discussed the M06/BS1 calculated free energies (*i.e.* ΔG values) at 298.15 K unless otherwise specified.

**8. NBO Analysis of the Intermediate Complex B**

NBO and molecular orbital analysis on the pre-reaction complex **B** shows the existence of a bonding between the Ni-center and C=O fragment of the substrate (called as Ni-(C=O) bond, see Supplementary Figure 159). Close examination shows that this orbital interacts with the bonding π-orbitals of the pyridine fragment and C(acyl)-O bond of substrate. The strength of these interactions are 9.1 and 1.1 kcal/mol, respectively. In addition, there exist a donor-acceptor interaction between the π-orbital of pyridine and empty orbital of Ni ($Ni_{LP}*$), *i.e.* E[Pyr → $Ni_{LP}*$], as well as between bonding C(acyl)–O orbital and empty orbital of Ni ($Ni_{LP}*$), *i.e.* E[(C-O)→ $Ni_{LP}*$]. Again, the Pyr → $Ni_{LP}*$ interaction is calculated to be significantly larger than the (C–O)→ $Ni_{LP}*$. It is clear that these π-interactions collectively play a stabilization role in the C(acyl)–O activation transition state.

**Supplementary References**


1. Amaike, K., Muto, K., Yamaguchi, J. & Itami, K. Decarbonylative C–H coupling of azoles and aryl esters: unprecedented nickel catalysis and application to the synthesis of muscoride A. *J. Am. Chem. Soc.* **134**, 13573–13576 (2012).
2. Ladouceur, G. H., Connell, R. D., Baryza, J., Campbell, A.-M., Lease, T. G. & Cook, J. H. U. S. Patent 1998024627.
3. Eickmeier, C., Fuchs, K., Peters, S., Dorner-Ciossek, C., Heine, N., Handschuh, S., Klinder, K. & Kostka, M. Eur. Patent 2006002769.
4. Palmer, J. T. *et al.* Design and synthesis of tri-ring $P_3$ benzamide-containing aminonitriles as potent, selective, orally effective inhibitors of cathepsin K. *J. Med. Chem.* **48**, 7520–7534 (2005).
5. Roy, H. N. & Al Mamun, A. H. Regiospecific phenyl esterification to some organic acids catalyzed by combined Lewis acids. *Synth. Commun.* **36**, 2975–2981 (2006).
6. Bottalico, D., Fiandanese, V., Marchese, G. & Punzi, A. A new versatile synthesis of esters from Grignard reagents and chloroformates. *Synlett* **2007**, 974–976 (2007).
7. Qin, C. *et al.* Palladium-catalyzed aromatic esterification of aldehydes with organoboronic acids and molecular oxygen. *Org. Lett.* **10**, 1537–1540 (2008).
8. Ueda, T., Konishi, H. & Manabe, K. Palladium-catalyzed carbonylation of aryl, alkenyl, and allyl halides with phenyl formate. *Org. Lett.* **14**, 3100–3103 (2012).
9. Ackermann, L. & Althammer, A. Air-stable PinP(O)H as preligand for palladium-catalyzed Kumada couplings of unactivated tosylates. *Org. Lett.* **8**, 3457–3460 (2006).
10. Guan, B.-T., Wang, Y., Li, B.-J., Yu, D.-G. & Shi, Z.-J. Biaryl construction via Ni-catalyzed C−O activation of phenolic carboxylates. *J. Am. Chem. Soc.* **130,** 14468–14470 (2008).
11. Shen, H.-C., Pal, S., Lian, J.-J. & Liu, R.-S. Ruthenium-catalyzed aromatization of aromatic enynes via the 1,2-migration of halo and aryl groups: a new process involving electrocyclization and skeletal rearrangement. *J. Am. Chem. Soc.* **125**, 15762–15763 (2003).

12. Tang, Y.-Q., Lv, H., Lu, J.-M. & Shao, L.-X. Palladium(II)-N-heterocyclic carbene complex derived from proline towards Suzuki–Miyaura coupling reaction in water at room temperature. *J. Organomet. Chem.* **696**, 2576–2579 (2011).
13. Denmark, S. E., Smith, R. C., Chang, W.-T. T. & Muhuhi, J. M. Cross-coupling reactions of aromatic and heteroaromatic silanolates with aromatic and heteroaromatic halides. *J. Am. Chem. Soc.* **131**, 3104–3118 (2009).
14. Kwong, F. Y., Chan, K. S., Yeung, C. H. & Chan, A. S. C. An active ferrocenyl triarylphosphine for palladium-catalyzed Suzuki–Miyaura cross-coupling of aryl halides. *Chem. Commun.* 2336–2337 (2004).
15. Hoshiya, N. *et al.* Sulfur modification of Au via treatment with piranha solution provides low-Pd releasing and recyclable Pd material, SAPd. *J. Am. Chem. Soc.* **132**, 7270–7272 (2010).
16. Leowanawat, P., Zhang, N., Resmerita, A.-M., Rosen, B. M. & Percec, V. $Ni(COD)_2/PCy_3$ catalyzed cross-coupling of aryl and heteroaryl neopentylglycolboronates with aryl and heteroaryl mesylates and sulfamates in THF at room temperature. *J. Org. Chem.* **76**, 9946–9955 (2011).
17. Molander, G. A., Trice, S. L. J. & Dreher, S. D. Palladium-catalyzed, direct boronic acid synthesis from aryl chlorides: a simplified route to diverse boronate ester derivatives. *J. Am. Chem. Soc.* **132**, 17701–17703 (2010).
18. Molander, G. A., Canturk, B. & Kennedy, L. E. Scope of the Suzuki–Miyaura cross-coupling reactions of potassium heteroaryltrifluoroborates. *J. Org. Chem.* **74**, 973–980 (2009).
19. Tamba, S., Okubo, Y., Tanaka, S., Monguchi, D. & Mori, A. Palladium-catalyzed C–H functionalization of heteroarenes with aryl bromides and chlorides. *J. Org. Chem.* **75**, 6998–7001 (2010).
20. Whitney, S. E., Winters, M. & Rickborn, B. Benzyne-oxazole cycloadducts: isolation and retro-Diels-Alder reactions. *J. Org. Chem.* **55**, 929–935 (1990).
21. Kirchberg, S. *et al.* Oxidative biaryl coupling of thiophenes and thiazoles with arylboronic acids through palladium catalysis: otherwise difficult C4-selective C–H arylation enabled by boronic acids. *Angew. Chem. Int. Ed.* **50**, 2387–2391 (2011).
22. Kobayashi, O., Uraguchi, D. & Yamakawa, T. $Cp_2Ni$-KO*t*-Bu-$BEt_3$ (or $PPh_3$) catalyst system for direct C–H arylation of benzene, naphthalene, and pyridine. *Org. Lett.* **11**, 2679–2682 (2009).
23. Cao, K., Zhang, F.-M., Tu, Y.-Q., Zhuo, X.-T. & Fan, C.-A. Iron(III)-catalyzed and air-mediated tandem reaction of aldehydes, alkynes and amines: an efficient approach to substituted quinolones. *Chem. Eur. J.* **15**, 6332–6334 (2009).
24. Nguyen, T. T., Marquise, N., Chevallier, F. & Mongin, F. Deprotonative metalation of aromatic compounds by using an amino-based lithium cuprate. *Chem. Eur. J.* **17**, 10405–10416 (2011).
25. Zhao, J., Zhao, Y. & Fu, H. Transition-metal-free intramolecular Ullmann-type O-arylation: synthesis of chromone derivatives. *Angew. Chem. Int. Ed.* **50**, 3769–3773 (2011).
26. Parmentier, M., Gros, P. & Fort, Y. Pyridino-directed lithiation of anisylpyridines: new access to functional pyridylphenols. *Tetrahedron* **61**, 3261–3269 (2005).
27. Saito, R., Tokita, M., Uda, K., Ishikawa, C. & Satoh, M. Synthesis and in vitro evaluation of botryllazine B analogues as a new class of inhibitor against human aldose reductase. *Tetrahedron* **65**, 3019–3026 (2009).

28. Tobisu, M., Hyodo, I. & Chatani, N. Nickel-catalyzed reaction of arylzinc reagents with N-aromatic heterocycles: a straightforward approach to C–H bond arylation of electron-deficient heteroaromatic compounds. *J. Am. Chem. Soc.* **131**, 12070–12071 (2009).
29. Chiba, S., Xu, Y.-J. & Wang, Y.-F. A Pd(II)-catalyzed ring-expansion reaction of cyclic 2-azidoalcohol derivatives: synthesis of azaheterocycles. *J. Am. Chem. Soc.* **131**, 12886–12887 (2009).
30. Barder, T. E., Walker, S. D., Martinelli, J. R. & Buchwald, S. L. Catalysts for Suzuki−Miyaura coupling processes: scope and studies of the effect of ligand structure. *J. Am. Chem. Soc.* **127**, 4685–4696 (2005).
31. Molander, G. A. & Iannazzo, L. Palladium-catalyzed Hiyama cross-coupling of aryltrifluorosilanes with aryl and heteroaryl chlorides. *J. Org. Chem.* **76**, 9182–9187 (2011).
32. Vella, S. J., Tiburcio, J., Gauld, J. W. & Loeb, S. J. Push-pull [2]pseudorotaxanes. Electronic control of threading by switching ON/OFF an intramolecular charge transfer. *Org. Lett.* **8**, 3421–3424 (2006).
33. Chen, G.-J., Huang, J., Gao, L.-X. & Han, F.-S. Nickel-catalyzed cross-coupling of phenols and arylboronic acids through an in situ phenol activation mediated by PyBroP. *Chem. Eur. J.* **17**, 4038–4042 (2011).
34. Gooßen, L. J., Rodríguez, N., Lange, P. P. & Linder, C. Decarboxylative cross-coupling of aryl tosylates with aromatic carboxylate salts. *Angew. Chem. Int. Ed.* **49**, 1111–1114 (2010).
35. Hiraoka, S., Sakata, Y. & Shionoya, M. Ti(IV)-centered dynamic interconversion between Pd(II), Ti(IV)-containing ring and cage molecules. *J. Am. Chem. Soc.* **130**, 10058–10059 (2008).
36. Billingsley, K. & Buchwald, S. L. Highly efficient monophosphine-based catalyst for the palladium-catalyzed Suzuki–Miyaura reaction of heteroaryl halides and heteroaryl boronic acids and esters. *J. Am. Chem. Soc.* **129**, 3358–3366 (2007).
37. Nakao, Y., Imanaka, H., Sahoo, A. K., Yada, A. & Hiyama, T. Alkenyl- and aryl[2-(hydroxymethyl)phenyl]dimethylsilanes: an entry to tetraorganosilicon reagents for the silicon-based cross-coupling reaction. *J. Am. Chem. Soc.* **127**, 6952–6953 (2005).
38. Inés, B., Moreno, I., SanMartin, R. & Domínguez, E. A Nonsymmetric pincer-catalyzed Suzuki–Miyaura arylation of benzyl halides and other nonactivated unusual coupling partners. *J. Org. Chem.* **73**, 8448–8451 (2008).
39. *Gaussian 09, Revision D.01,* Frisch, M. J., Trucks, G. W., Schlegel, H. B., Scuseria, G. E., Robb, M. A., Cheeseman, J. R., Scalmani, G., Barone, V., Mennucci, B., Petersson, G. A., Nakatsuji, H., Caricato, M., Li, X., Hratchian, H. P., Izmaylov, A. F. Bloino, J., Zheng, G., Sonnenberg, J. L., Hada, M., Ehara, M., Toyota, K., Fukuda, R., Hasegawa, J., Ishida, M., Nakajima, T., Honda, Y., Kitao, O., Nakai, H., Vreven, T., Montgomery, Jr., J. A., Peralta, J. E., Ogliaro, F., Bearpark, M., Heyd, J. J., Brothers, E., Kudin, K. N., Staroverov, V. N., Keith, T., Kobayashi, R., Normand, J., Raghavachari, K., Rendell, A., Burant, J. C., Iyengar, S. S., Tomasi, J., Cossi, M., Rega, N., Millam, J. M., Klene, M., Knox, J. E., Cross, J. B., Bakken, V., Adamo, C., Jaramillo, J., Gomperts, R., Stratmann, R. E., Yazyev, O., Austin, A. J., Cammi, R., Pomelli, C., Ochterski, J. W., Martin, R. L., Morokuma, K., Zakrzewski, V. G., Voth, G. A., Salvador, P., Dannenberg, J. J., Dapprich, S., Daniels, A. D., Farkas, O., Foresman, J. B., Ortiz, J. V., Cioslowski, J. & Fox, D. J. Gaussian, Inc., Wallingford CT, **2013.**
40. Zhao, Y. & Truhlar, D. G. A new local density functional for main-group thermochemistry, transition metal bonding, thermochemical kinetics, and noncovalent interactions. *J. Chem. Phys.* **125**, 194101 (2006).

41. Wadt, W. R. & Hay, P. J. *Ab initio* effective core potentials for molecular calculations. Potentials for main group elements Na to Bi. *J. Chem. Phys.* **82**, 284–298 (1985).
42. Hay, P. J. & Wadt, W. R. *Ab initio* effective core potentials for molecular calculations. Potentials for K to Au including the outermost core orbitals. *J. Chem. Phys.* **82**, 299–310 (1985).
43. Mennucci, B. & Tomasi, J. A new integral equation formalism for the polarizable continuum model:Theoretical background and applications to isotropic and anisotropicdielectrics. *J. Chem. Phys.* **106**, 3032–3041 (1997).
44. Mennucci, B. & Tomasi, J. Continuum solvation models: A new approach to the problem of solute's charge distribution and cavity boundaries. *J. Chem. Phys.* **106**, 5151–5158 (1997).
45. Scalmani, G. & Frisch, M. J. Continuous surface charge polarizable continuum models of solvation. I. General formalism. *J. Chem. Phys.* **132**, 114110 (2010).
46. Xu, H., Muto, K., Yamaguchi, J., Zhao, C., Itami, K. & Musaev, D. G. Key mechanistic features of Ni-catalyzed C–H/C–O biaryl coupling of azoles and naphthalen-2-yl pivalates. *J. Am. Chem. Soc.* **136**, 14834–14844 (2014)
47. For M06 method see: (a) Zhao, Y. & Truhlar, D. G. The M06 suite of density functionals for main group thermochemistry, thermochemical kinetics, noncovalent interactions, excited states, and transition elements: two new functionals and systematic testing of four M06-class functionals and 12 other functionals. *Theor. Chem. Acc.* **120**, 215–241 (2008). (b) Zhao, Y. & Truhlar, D. G. Density functionals with broad applicability in chemistry. *Acc. Chem. Res.* **41**, 157–167 (2008). (c) Zhao, Y. & Truhlar, D. G. Benchmark energetic data in a model system for Grubbs II metathesis catalysis and their use for the development, assessment, and validation of electronic structure methods. *J. Chem. Theory Comput.* **5**, 324–333 (2008).
